# Enzymatic Control over Reactive Intermediates Enables Direct Oxidation of Alkenes to Carbonyls by a P450 Iron-Oxo Species

**DOI:** 10.1021/jacs.2c02567

**Published:** 2022-08-23

**Authors:** Jordi Soler, Sebastian Gergel, Cindy Klaus, Stephan C. Hammer, Marc Garcia-Borràs

**Affiliations:** † Institut de Química Computacional i Catàlisi (IQCC) and Departament de Química, Universitat de Girona, Carrer Maria Aurèlia Capmany 69, Girona 17003, Catalonia, Spain; ‡ Chair of Organic Chemistry and Biocatalysis, Faculty of Chemistry, Bielefeld University, Universitätsstraße 25, 33615 Bielefeld, Germany

## Abstract

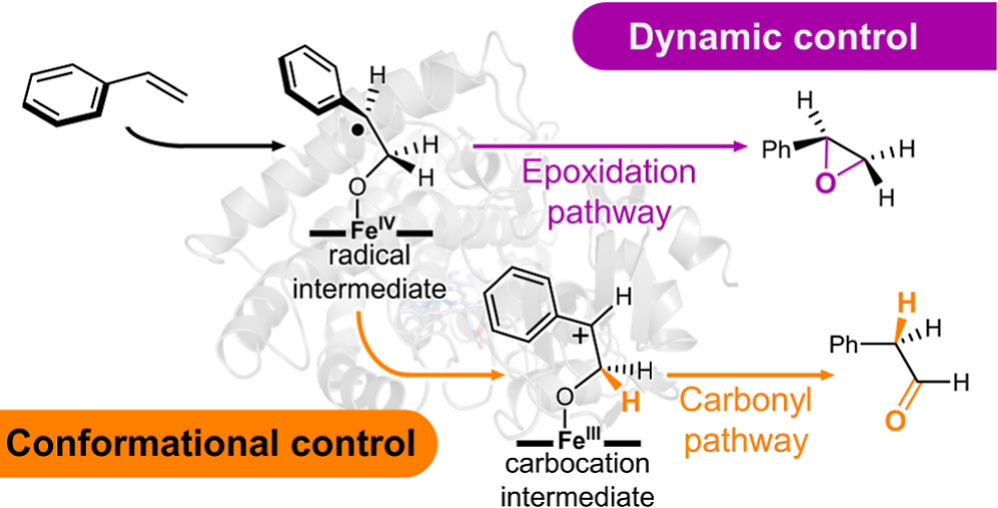

The aerobic oxidation of alkenes to carbonyls is an important and challenging transformation in synthesis. Recently, a new P450-based enzyme (aMOx) has been evolved in the laboratory to directly oxidize styrenes to their corresponding aldehydes with high activity and selectivity. The enzyme utilizes a heme-based, high-valent iron-oxo species as a catalytic oxidant that normally epoxidizes alkenes, similar to other catalysts. How the evolved aMOx enzyme suppresses the commonly preferred epoxidation and catalyzes direct carbonyl formation is currently not well understood. Here, we combine computational modelling together with mechanistic experiments to study the reaction mechanism and unravel the molecular basis behind the selectivity achieved by aMOx. Our results describe that although both pathways are energetically accessible diverging from a common covalent radical intermediate, intrinsic *dynamic effects* determine the strong preference for epoxidation. We discovered that aMOx overrides these intrinsic preferences by controlling the accessible conformations of the covalent radical intermediate. This disfavors epoxidation and facilitates the formation of a carbocation intermediate that generates the aldehyde product through a fast 1,2-hydride migration. Electrostatic preorganization of the enzyme active site also contributes to the stabilization of the carbocation intermediate. Computations predicted that the hydride migration is stereoselective due to the enzymatic conformational control over the intermediate species. These predictions were corroborated by experiments using deuterated styrene substrates, which proved that the hydride migration is *cis*- and enantioselective. Our results demonstrate that directed evolution tailored a highly specific active site that imposes strong steric control over key fleeting biocatalytic intermediates, which is essential for accessing the carbonyl forming pathway and preventing competing epoxidation.

## Introduction

The aerobic oxidation of alkenes to the corresponding carbonyl compounds is an important but often challenging transformation in organic chemistry.^1−3^ The value of this oxidation reaction results from the easy accessibility of alkenes from petroleum, renewable resources, or well-established synthetic methods such as carbonyl olefination and olefin metathesis. In addition, aldehydes and ketones are very relevant functional groups in valuable molecules and crucial intermediates in synthesis. Alkene to carbonyl oxidation is established by the palladium(II)-catalyzed Wacker-Tsuji oxidation, yet, this reaction is in part limited to oxidizing ethylene to acetaldehyde and 1-alkenes to methyl ketones.^1^ Although significant efforts have been made to extend Wacker-type oxidation to yield other types of carbonyl compounds,^1−3^ efficient and selective aerobic alkene to carbonyl oxidations are rare and sought after.

**Scheme 1:**
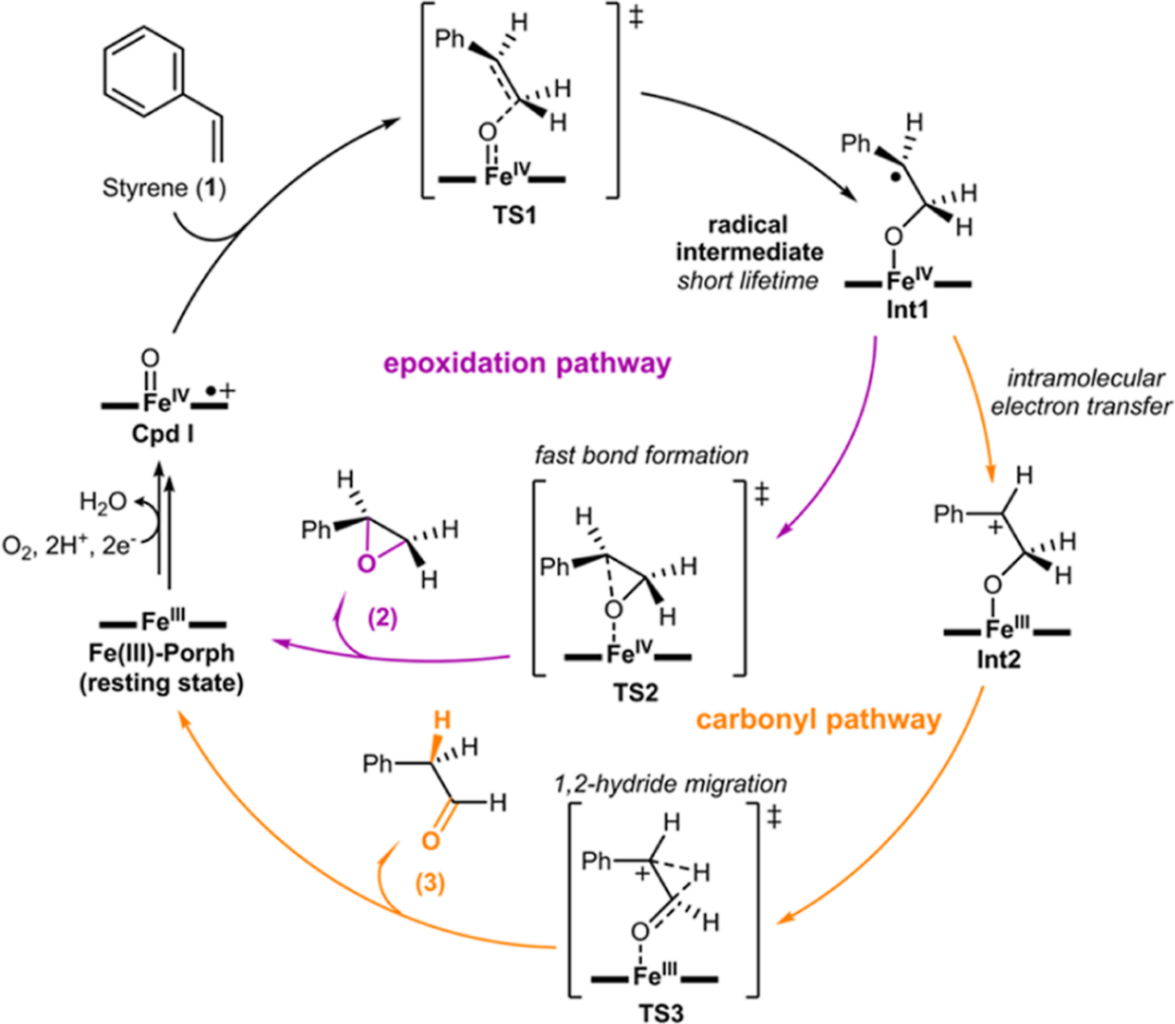
Iron-oxo-Mediated Alkene Oxidation by an Iron Porphyrin-Type Catalyst Iron-oxo-mediated alkene oxidation generally leads to the corresponding epoxide product. The proposed catalytic cycles for P450-catalyzed alkene epoxidation (epoxide pathway, purple) and anti-Markovnikov oxidation (carbonyl pathway, orange) with styrene (**1**) as the model substrate start with the formation of an iron-oxo complex, termed compound I (**Cpd I**). The first C–O bond formation (**TS1**) leads to a short-lived radical intermediate (**Int1**) that directly converts to the epoxide product (**2**) by a very fast second C–O bond-forming step (**TS2**). These two C–O bond-forming steps proceed most often in a stereospecific manner and might occur stepwise (without epimerization when the shallow reactive radical intermediate is formed) or in a concerted fashion. The alternative stepwise anti-Markovnikov oxidation (carbonyl pathway) is proposed to occur via an intramolecular electron transfer, yielding a highly reactive carbocation intermediate (**Int2**). Subsequent 1,2-hydride migration (**TS3**) produces the carbonyl product, aldehyde **3**.

Experiments and theory suggest that high-valent metal-oxo species can be exploited as catalytic oxidants to convert alkenes directly to carbonyl compounds.^4−6^ High-valent metal-oxo complexes are known in chemistry and biology for oxidation chemistry;^7−10^ however, these species typically epoxidize alkenes. Established catalysts for alkene epoxidation via high-valent metal-oxo complexes include Jacobsen’s manganese(III)salen complexes,^11^ synthetic metalloporphyrins,^12^ biomimetic non-heme iron complexes,^13−15^ and enzymes such as heme-dependent cytochrome P450 monooxygenases^16^ and peroxygenases^17^ or non-heme α-ketoglutarate-dependent dioxygenases.^18^ The exact mechanism of metal-oxo-mediated alkene epoxidation has long been controversial.^5,6,10,19−22^ A currently widely accepted view is that epoxide formation proceeds via an almost concerted reaction mechanism, potentially through an extremely short lived radical intermediate (Scheme 1). Support for a concerted nature of the oxo transfer or a two-step mechanism in which the second step occurs very fast comes from the retention of the stereochemistry in epoxidation of *cis*-alkenes.^11,12,16,17^ It is believed that metal-oxo-mediated alkene to carbonyl oxidation proceeds from the radical intermediate via an electron/hydride transfer process including the formation of a carbocation species (Scheme 1).^5,23^ This proposed electron transfer process is related to the carbocation formation in metal-oxo-mediated oxidative rearrangements, for example, in pentalenolactone biosynthesis, catalyzed by a P450 enzyme that includes an intramolecular electron transfer.^24,25^ Although direct alkene to carbonyl oxidation is theoretically accessible through oxo transfer chemistry, carbonyl products are only observed as minor by-products, if at all.^6,23^ High-valent metal-oxo species have never been exploited for carbonyl synthesis due to the lack of catalysts that suppress the strongly favored epoxide formation.^26^ The origin of high chemoselectivity that favors epoxidation over carbonyl formation is not completely understood. Recently, however, directed evolution has been harnessed to generate a biocatalyst that outcompetes epoxidation. A laboratory-evolved iron-heme P450 enzyme called anti-Markovnikov oxygenase (aMOx) directly oxidizes alkenes to carbonyls with high selectivity and activity (up to 94% carbonyl selectivity and up to 4500 total turnover numbers, TTN).^26^ This aMOx enzyme was evolved from a P450 monooxygenase of *Labrenzia aggregata* (P450_LA1_), which possessed initial carbonyl forming activity.^27^ The physiological substrate of P450_LA1_ is unknown, and this enzyme has been shown to catalyze various reactions such as asymmetric sulfoxidation, sp^3^ C–H bond hydroxylation, and alkene epoxidation on diverse compounds. A homolog of P450_LA1_ within the CYP116B subfamily was found to catalyze aromatic C–H bond hydroxylation in 2-hydroxyphenylacetic acid catabolism, which is the first demonstration of a physiological substrate for an enzyme from the CYP116B subfamily.^28^ The evolved aMOx enzyme is the first catalyst to use metal-oxo species for selective alkene to carbonyl oxidation and thus a good opportunity to study the origin of chemoselectivity in this reaction.

**Figure 1:**
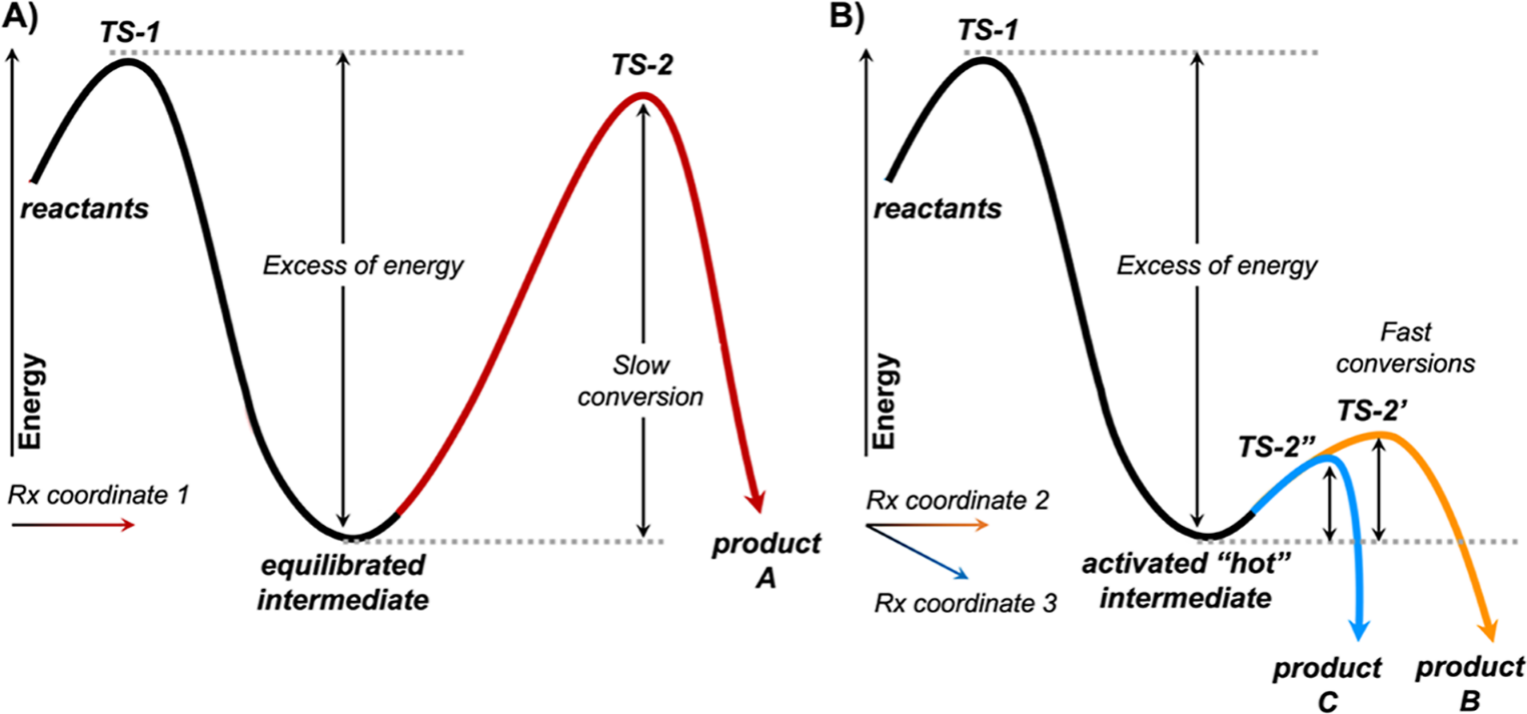
Activated “hot” intermediates. Schematic comparison of the behavior of two intermediates that are generated from the first transition state (**TS-1**) with a large excess of the kinetic energy. (A) High-energy second transition state, **TS-2**, implies a slow conversion of the intermediate. This scenario allows thermal equilibration of the intermediate, loss of excess energy to the surrounding solvent molecules, and intramolecular vibrational energy redistribution. Transition state theory can be successfully applied to predict reaction rates, intermediate lifetimes, and product selectivities. (B) Low-energy second transition state, **TS-2′** or **TS-2″**, implies a fast conversion of the intermediate. At these short timescales, the reactivity of the intermediate can compete with its excess energy redistribution and thermal equilibration. Consequently, the intermediate is not thermally equilibrated and can be termed as an activated “hot” intermediate. In such a scenario, the dynamic behavior of the activated “hot” intermediate influences its reactivity. Under dynamic control, the reaction can lead to product B through **TS-2′** (non-IRC pathway) if there exists a coupling between the kinetic energy of the intermediate and the modes that promote its subsequent reaction, although a lower-energy pathway to product C through **TS-2″** exists (lowest-energy IRC pathway).

As we will describe below, iron-oxo-mediated alkene oxidation can involve a short-lived radical intermediate that is generated with a large excess of the kinetic energy (Figure 1). This is not unusual and, in fact, common to any intermediate or product derived from a high-energy transition state (TS).^29^ This excess energy originates from potential energy that is in part transformed into vibrational energy during the intermediate or product formation (Figure 1A). Because energy redistribution via intramolecular and intermolecular processes is extremely fast in solution,^30^ excess vibrational energy does not typically affect subsequent reactions of an intermediate. Consequently, statistical thermodynamic treatments, such as transition state theory, can be successfully applied to rationalize and predict experimental observations. However, this is not the case if the lifetime of a reactive intermediate is very short and reaches the femtosecond to picosecond timescale. At this timescale, the reactivity of the intermediate can compete with its excess energy redistribution and thermal equilibration.^29^ For reactive intermediates with very short lifetimes, non-statistic distribution of energy can affect their dynamic behavior and thus control the reactivity of these species (Figure 1B). Changes in reactivity can, for example, take place via selective vibrational activation of one part of a molecule or by specific vibrations with a displacement (momentum) that correlates with the geometry of a subsequent transition state and its displacement vector corresponding to the imaginary frequency (*dynamic match*). The reactivity of energetically non-equilibrated intermediates (kinetically activated, “*hot*” intermediates) has received significant attention recently.^29,31^ It has been shown that dynamic effects and the amount of excess energy and how it is distributed can control the reactivity of intermediates in solution,^32,33^ in organometallic reactions,^34,35^ and in enzymes.^36−38^

Here, we show that the high chemoselectivity in metal-oxo-mediated alkene oxidation by iron porphyrin-type catalysts is a consequence of the radical’s dynamic behavior in its kinetically (vibrational) activated state. We further reveal how an evolved heme-dependent enzyme overwrites this dynamically controlled process to generate carbonyls instead of epoxides. Directed evolution of aMOx not only steered oxidation activity toward acceptance of a non-natural substrate but enabled control over the accessible conformations of the reactive intermediates by preorganizing them to favor stereoselective carbonyl formation, while disfavoring epoxidation. This conformational control is achieved by confinement^39^ in the active site of the enzyme. Experiments with isotopically labeled substrates support the computational findings and proposed reaction mechanism.

## Results and Discussion

### Exploration of the Intrinsic Oxidation Reaction Mechanism Using Density Functional Theory Calculations on an Enzyme-free Computational Truncated Model

**Figure 2:**
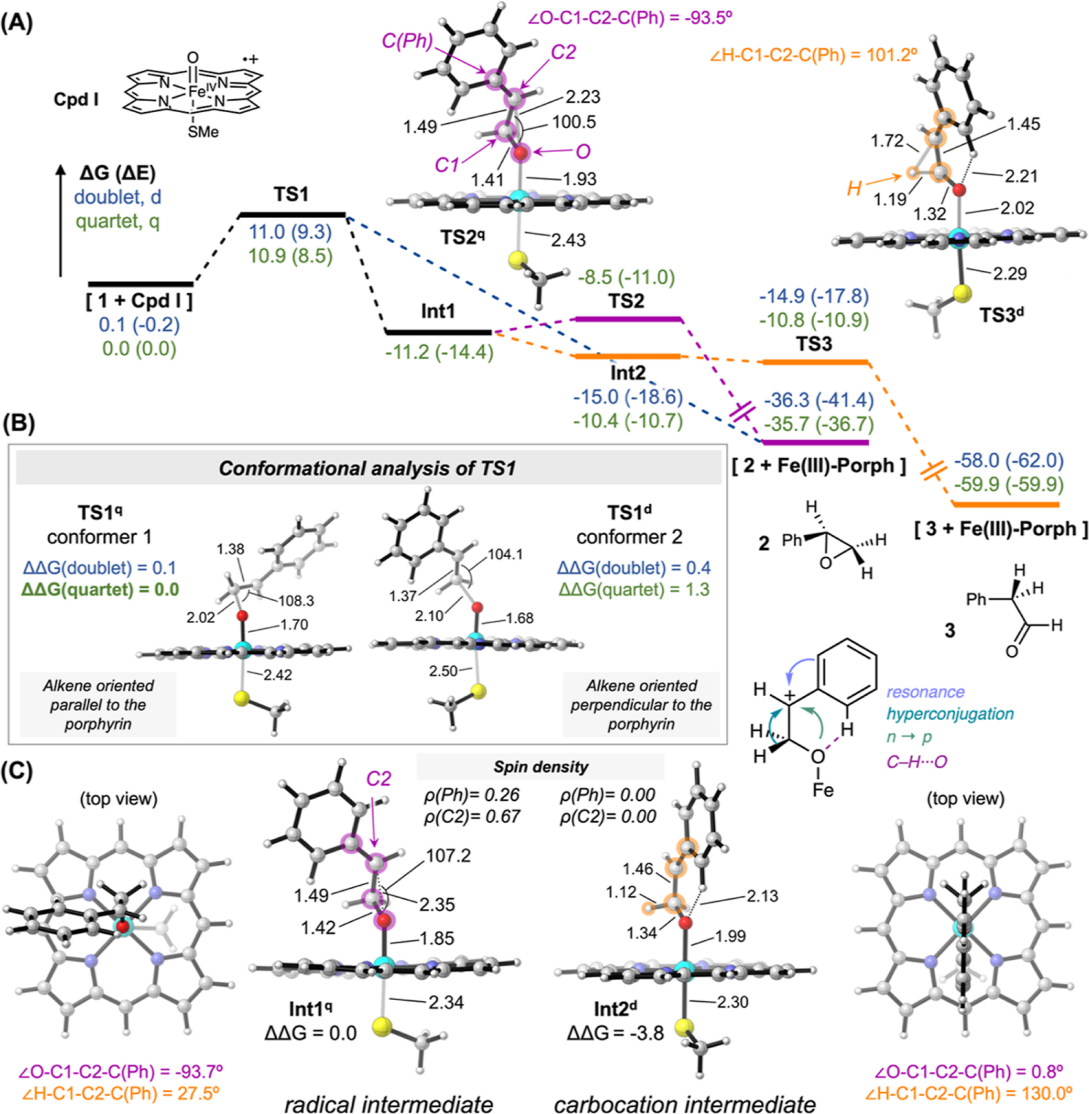
Enzyme-free model DFT calculations. (A) DFT-calculated competing reaction pathways for styrene (**1**) epoxidation (purple pathway) and carbonyl formation (orange pathway) catalyzed by the iron-oxo heme enzyme-free computational model. Relative Gibbs energies (Δ*G*) and electronic energies (Δ*E*, in parenthesis) are reported, in blue for the doublet electronic state (d) and in green for the quartet electronic state (q). Lowest-energy DFT-optimized key transition state geometries are shown. See Figure S1 for additional details on the intrinsic mechanisms. (B) Conformational analysis of **TS1**. (C) Lowest-energy DFT-optimized structures of key covalent radical and carbocation intermediates (**Int1** and **Int2**, respectively). Energies, distances, angles, and spin density values are given in kcal·mol^–1^, angstroms (Å), degrees (°), and atomic units (a.u.), respectively.

To understand the origin of chemoselectivity in iron-oxo-mediated alkene oxidation by iron porphyrin-type catalysts, we first studied the intrinsic reaction mechanism using density functional theory (DFT) calculations and a truncated model (Figure 2, see Supporting Information for computational details). This simplified model includes the high-valent iron-oxo species coordinated to the porphyrin pyrrole core and a methanethiolate moiety that mimics the axial cysteine ligand in compound I (Cpd I). We used styrene as the model substrate because this alkene is typically used in epoxidation reactions, and it was also the substrate for evolving the aMOx enzyme that performs chemoselective carbonyl formation. The energetically accessible spin states (doublet, d; and quartet, q) of the catalytic iron species have been considered and are discussed if necessary (see SI for details).^10,40^

As styrenes are generally epoxidized by high-valent iron-oxo species,^11−17^ one might expect to find significantly lower-energy barriers for epoxidation than for the competing carbonyl pathway. This would be in line with transition state theory and relate product selectivities to differences in activation barriers. However, the calculated reaction mechanism for iron-oxo-mediated styrene oxidation revealed that the carbonyl pathway is not only energetically accessible but even slightly preferred over epoxidation (Figures 2A, and S1), although exhibiting small energy differences that are in the accuracy limit of the DFT methodology used.^41^ Calculations describe that the formation of the first C–O bond is the shared rate-limiting step for both epoxidation and carbonyl pathways (**TS1**, Δ*G*^‡^ = 10.9 and 11.0 kcal·mol^–1^ in the quartet and doublet states, respectively). Different conformers evaluated for **TS1** revealed no significant energy differences (see Figure 2B). Intrinsic reaction coordinate (IRC) calculations (see Supporting Information) describe that **TS1** in the doublet electronic state directly leads to the epoxide product via formation of the second C–O bond in an asynchronous but concerted manner, while quartet **TS1** forms a covalent radical intermediate (**Int1**). The generated radical intermediate can either lead to the epoxide product via a low-energy transition state (**TS2**, Δ*G*^‡^ = 2.7 kcal·mol^–1^) or to the carbonyl product in an almost barrierless pathway. The latter involves the formation of a covalent carbocation intermediate (**Int2**), that is, ΔΔ*G* = 0.8 kcal·mol^–1^ higher in energy (quartet) and −3.8 kcal·mol^–1^ more stable (doublet) with respect to the radical intermediate (Figures 2C and S1). The formation of this key carbocation intermediate is triggered by a conformational change, the rotation of the benzyl group of the substrate, that enables stabilization of the benzylic carbocation via resonance with the aromatic ring and hyperconjugation with the former vinylic α-C–H bonds (Figures 2C, and S1–S3). Within this conformation, the lone pairs of the oxygen atom (2p orbitals) can stabilize the empty p orbital of the carbocation through a *n* → *p* interaction. Additionally, a C–H···O interaction can also be established that stabilizes the positive charge partially delocalized on the aromatic ring by resonance. In this optimal conformation, the α-hydrogen is well aligned with the empty p-orbital of the benzylic carbocation for an effective 1,2-migration. The carbonyl product **3** can be generated in either spin state by barrierless hydride migration (**TS3**) from carbocation **Int2**.

Relaxed scan calculations along the benzyl rotation coordinate were used to study the geometric distortion required to convert **Int1** to an **Int2**-like geometry (from ∠O–C1–C2–C(Ph) ca. −90° to ca. 0°, respectively; see Figure S2). These calculations indicated that this conformational change is energetically feasible (ca. 3.0 kcal·mol^–1^ in electronic energy, Δ*E*) and that the electron reorganization is thermodynamically favorable, once the rotation has occurred (∠O–C1–C2–C(Ph) ca. 0°, Δ*E* ca. −0.7 kcal·mol^–1^ from radical to carbocation). Consequently, the proposed intramolecular electron transfer from the radical intermediate **Int1** to generate the carbocation **Int2** can be described as a conformationally-gated process and can energetically outcompete the epoxide forming **TS2** (Δ*E*^‡^ = 3.4 kcal·mol^–1^). This process is assumed to be a fast inner sphere intramolecular electron rearrangement, once the geometry for the carbocation formation is reached, in analogy to the intermolecular electron transfer in pentalenolactone biosynthesis.^24,25^ This process is found to involve an inversion of the frontier molecular orbitals, where the electron from the α-HOMO (highest occupied molecular orbital) in the quartet radical intermediate moves to the energetically accessible α-LUMO+1 (lowest unoccupied molecular orbital, see Figure S3). Once formed, the carbocation intermediate **Int2** in the quartet electronic state can access the lower-energy doublet **Int2** through a minimum energy crossing point (MECP)^42^ that is energetically and geometrically very similar to the quartet **Int2** optimized structure (Figure S4). Our calculations showed that polar effects are rather important in order to stabilize the carbocation intermediate **Int2**. Gas phase DFT model calculations indicated that the optimized doublet **Int2**^**d**^(gas phase) is Δ*G* = +0.2 kcal·mol^–1^ higher in energy than radical intermediate **Int1**^**q**^(gas phase) and that the carbocation intermediate in the quartet electronic state cannot be optimized as a minimum on the gas phase potential energy surface (PES) (see Figure S27 in the Supporting Information). Finally, DFT calculations also showed that the presence of a precisely positioned water molecule hydrogen-bonding with the O-atom of the intermediate favors the relative stabilization of the carbocation with respect to the radical intermediate (Figures S8 and S9).

DFT calculations described a flat region of the PES, with the carbonyl formation being slightly energetically favored when diverging from quartet **TS1**. The lifetime of the radical intermediate predicted by transition state theory for the epoxidation pathway (**TS2**, Δ*G*^‡^ = 2.7 kcal·mol^–1^) is estimated to be *t*_1/2_ ≈ 1.06 × 10^–11^ s (10 600 fs). Considering a first-order reaction, a rate constant of *K* = 6.52 × 10^10^ s^–1^ can be derived from the Eyring equation. This is more than one order of magnitude slower than the predicted rate constant for carbonyl formation (*K* = 2.67 × 10^12^ s^–1^) via **TS3** from **Int1** (quartet electronic state, Δ*G*^‡^ = 0.5 kcal·mol^–1^). According to transition state theory, such reactions should preferentially lead to carbonyl products instead of the experimentally observed epoxides (Figure 2A). This reveals that thermostatistical methods such as transition state theory cannot completely explain why metal-oxo-mediated iron porphyrin- or P450-catalyzed alkene oxidations favor epoxidation, while carbonyl formation is rarely observed as a side product, if at all.^4,23^

**Figure 3:**
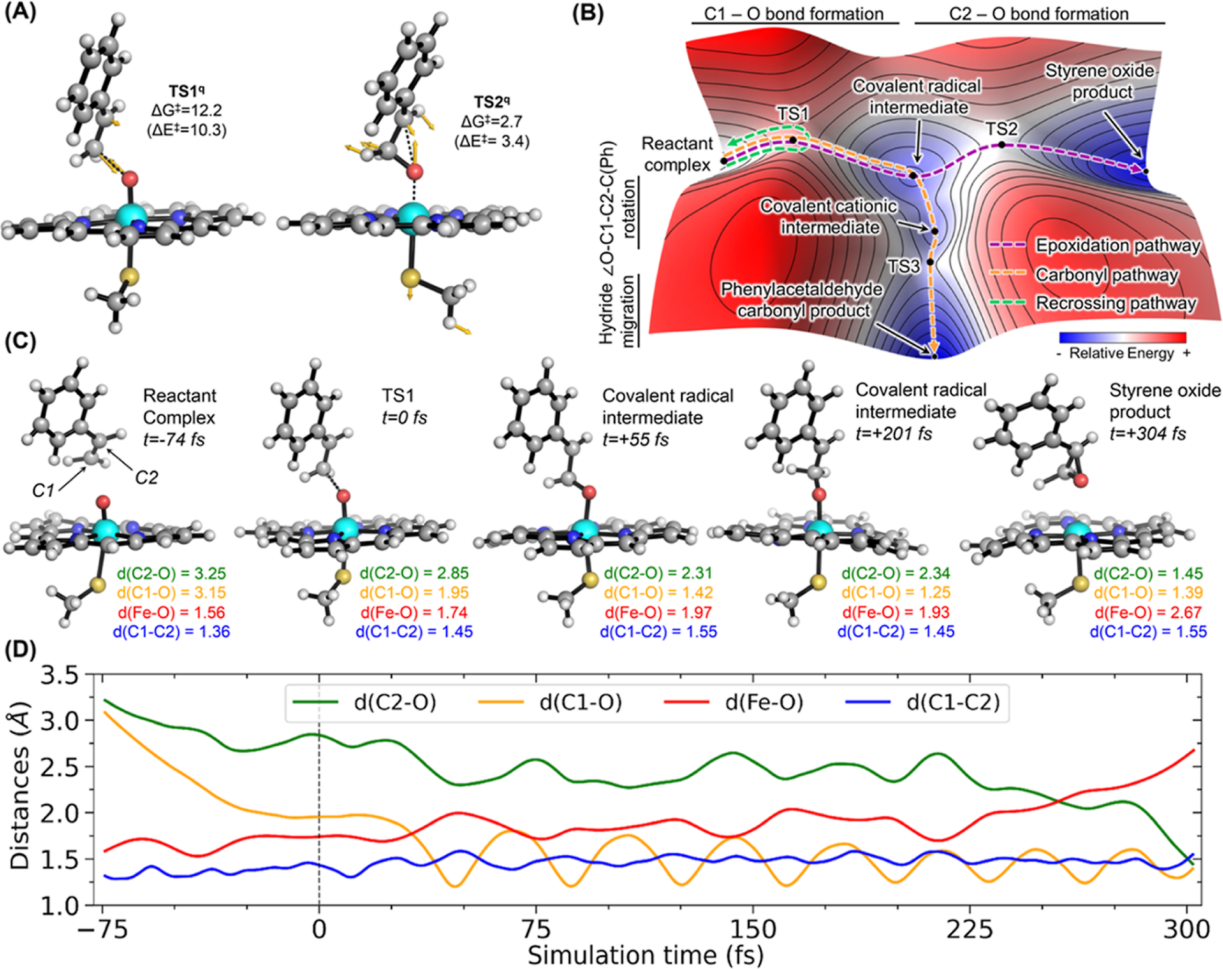
Quasiclassical direct dynamic trajectories (A) Enzyme-free DFT-optimized transition state structures **TS1**-q (used as the starting point for direct QCT simulations) and **TS2**-q. Yellow arrows are the displacement vectors associated with the transition-state structure imaginary frequency. (B) Qualitative three-dimensional energy landscape where different possible pathways are highlighted: dynamically preferred epoxidation pathway highlighted as a purple dashed line, the thermodynamically preferred carbonyl pathway as an orange dashed line, and an example of recrossing as a green dashed line. (C) Selected snapshots along a quartet quasiclassical trajectory from the reactant complex to styrene oxide formation. (D) Time evolution of most relevant distances along the quasiclassical trajectory shown in (C). Energies, distances, and time are given in kcal·mol^–1^, Angstroms (Å), and femtoseconds (fs), respectively. See also Figure S6 for additional details.

We hypothesized that non-statistical effects and the dynamic behavior of the radical intermediate could determine the chemoselectivity in this oxo transfer reaction. This idea is supported by the very short lifetime predicted for this radical (in the picosecond timescale), which can thus compete with redistribution of an excess of the kinetic energy. In this particular case, the radical intermediate is generated from **TS1** with a high amount of kinetic energy (ΔΔ*G* = 22.1 kcal·mol^–1^, see Figure 2A), rendering it a kinetically activated (“hot”) intermediate (Figure 1). Analysis of the momentum vectors corresponding to the negative eigenvalues of **TS1** and **TS2** already indicate a strong coupling and “*dynamic match*” between both transition states (Figures 3A, and S5), suggesting that kinetic activation and resulting non-statistic dynamic behavior of the radical intermediate could strongly favor three-membered ring formation.

### Quasiclassical Direct Dynamic Trajectory Simulations

To investigate the influence of a non-equally distributed excess of the kinetic energy on the chemoselectivity of the reaction (i.e., nonstatistical effects), we used direct quasiclassical trajectories (QCTs) to study the reaction dynamics.^29,35,43^ QCTs allow us to explicitly consider the atomic motions during a chemical reaction, which could describe the formation of nonstatistical (activated, “hot”) intermediates without vibrational energy redistribution and the possible existence of non-intrinsic reaction pathways (non-IRC, Figure 1B). Non-IRC pathways can lead to products that do not correspond to those expected from the lowest-energy pathway (i.e., IRC). QCTs have been previously employed to comprehend selectivities in competing reactions that bifurcate after a shared transition state, to characterize the formation of shallow entropic intermediates along reaction pathways or to study the dynamic behavior of reactive intermeditates.^29,35,38,43−47^ Here, QCTs were applied in order to identify potential non-statistical dynamic effects that might influence the lifetime and/or the behavior of the reactive radical intermediate.

Using our computational truncated model, QCTs were obtained (Figure 3). Direct QCTs were propagated forward and backward for 1200 fs (600 fs in each direction), starting from optimized **TS1** structures for each energetically accessible spin state (20 trajectories for each electronic state, a total of 40 trajectories. See SI for computational details). Please note that a larger number of QCTs would be required in order to statistically and quantitatively predict the product distributions.^35^ None of the 40 trajectories accessed the carbonyl product or the carbocation intermediate (Figure S6). Instead, 7 resulted in non-reactive recrossing events (5 doublet + 2 quartet), 7 lead to the formation of the radical intermediate (quartet), and 26 trajectories (15 doublet + 11 quartet) generated the epoxide product (see Figure S6).

An example of a reactive trajectory on the quartet electronic state for epoxidation is depicted in Figure 3C,D (see Figure S6 for doublet electronic state results). From QCT simulations, the lifetime of the intermediate, defined as the time gap between C1–O and C2–O bond formation (see Table S2), is estimated to be ca. 265 fs for the quartet electronic state (ca. 92 fs in the doublet state).

Houk and co-workers proposed a timing criterion to distinguish between dynamically concerted and dynamically stepwise mechanisms.^43,48^ According to this criterion, a mechanism is considered as dynamically concerted if all the bonding changes are completed within less than a 60 fs timegap, while it is considered as dynamically stepwise otherwise. This dynamic criterion is complementary to the common definition of concerted versus stepwise mechanisms, based on the shape of the PES (having one or two potential energy barriers). In this framework, QCT simulations suggest that the epoxidation pathway (and the carbonyl formation pathway) in both the quartet and doublet electronic states can be classified as dynamically stepwise mechanisms, although they are potential-energy stepwise and concerted, respectively.

Geometrically concerted and asynchronous but dynamically stepwise reactions often involve the formation of entropic intermediates.^43^ Entropic intermediates are an ensemble of dynamical structures that reside in a shallow free-energy well, which correspond to a relatively flat region on the PES but not to local minima and thus cannot be optimized using DFT calculations when considering only the potential energy and not the free energy.^49^ These entropic intermediates must decrease their entropy to exit from this region of the PES. Considering this, QCT simulations are suggesting the existence of an entropic intermediate formed along the doublet PES, after **TS1** and prior epoxide product formation, although an intermediate structure could not be optimized as a minimum on the PES (Figure 2A). This indicates that the carbonyl forming pathway could also be accessible from the doublet electronic state by a non-intrinsic motion where the reaction occurs through geometries not part of the IRC pathway.^35^ When this entropic intermediate is formed, it could give access to the carbocation intermediate if properly stabilized by a strong geometric control (see the results for the calculated enzymatic reaction in next sections).

While statistical transition state theory, based on static DFT calculations, failed to sufficiently explain the experimentally observed chemoselectivity, QCTs indicated the strong preference for epoxidation due to dynamic effects. These simulations revealed that the direction of the atomic motions and momentum acquired right after surpassing **TS1** (formation of the first C1–O bond) are strongly coupled to the atomic motions required to overcome the low-energy barrier in **TS2** (epoxide formation) (Figure 3C,D). QCTs showed that the kinetic energy acquired by the radical intermediate formed from **TS1** largely influences its lifetime and dynamically primes the reaction for three-membered ring formation, explaining the origin of the observed high chemoselectivity for epoxidation. Simulations also indicated that this dynamic preference for epoxidation is present, whether there is a small potential energy barrier (quartet electronic state, **TS2**) or not (doublet electronic state).

This “*dynamic match*” between the **TS1** and **TS2** reaction coordinates favors epoxidation and is illustrated in Figure 3B through a qualitative PES. The preference for epoxidation caused by the intrinsic dynamic behavior following **TS1** could in fact explain the preferred alkene epoxidation for many catalysts^11−18^ that utilize high-valent iron-oxo (or metal-oxo) species as the oxidant. In contrast, the carbonyl pathway requires a specific C1–C2 bond rotation to efficiently stabilize the carbocation intermediate and to allow a 1,2-hydride migration. Due to stereoelectronic effects described above, carbonyl formation depends on very specific conformations of the reactive intermediates that are not dynamically supported. However, how did the directed enzyme evolution reverse the chemoselectivity in this reaction toward carbonyl formation?

An important conclusion from DFT calculations and QCTs using a truncated model is that many natural enzymes, including cytochrome P450 monooxygenases^16^ and peroxygenases,^17^ achieve selective alkene epoxidation because they exploit the intrinsic preference dictated by the existing “*dynamic match*” between the two consecutive, dynamically stepwise, C–O bond formations. Consequently, an enzyme (or a catalyst) that aims to produce carbonyls must overwrite this intrinsic dynamic preference, potentially by allowing the kinetically activated radical intermediate to reach thermal equilibration or by imposing geometric constraints to control the accessible conformations of the intermediate species and transition states.

### Computational Modelling of the Enzyme–Substrate Bound Complex

**Figure 4:**
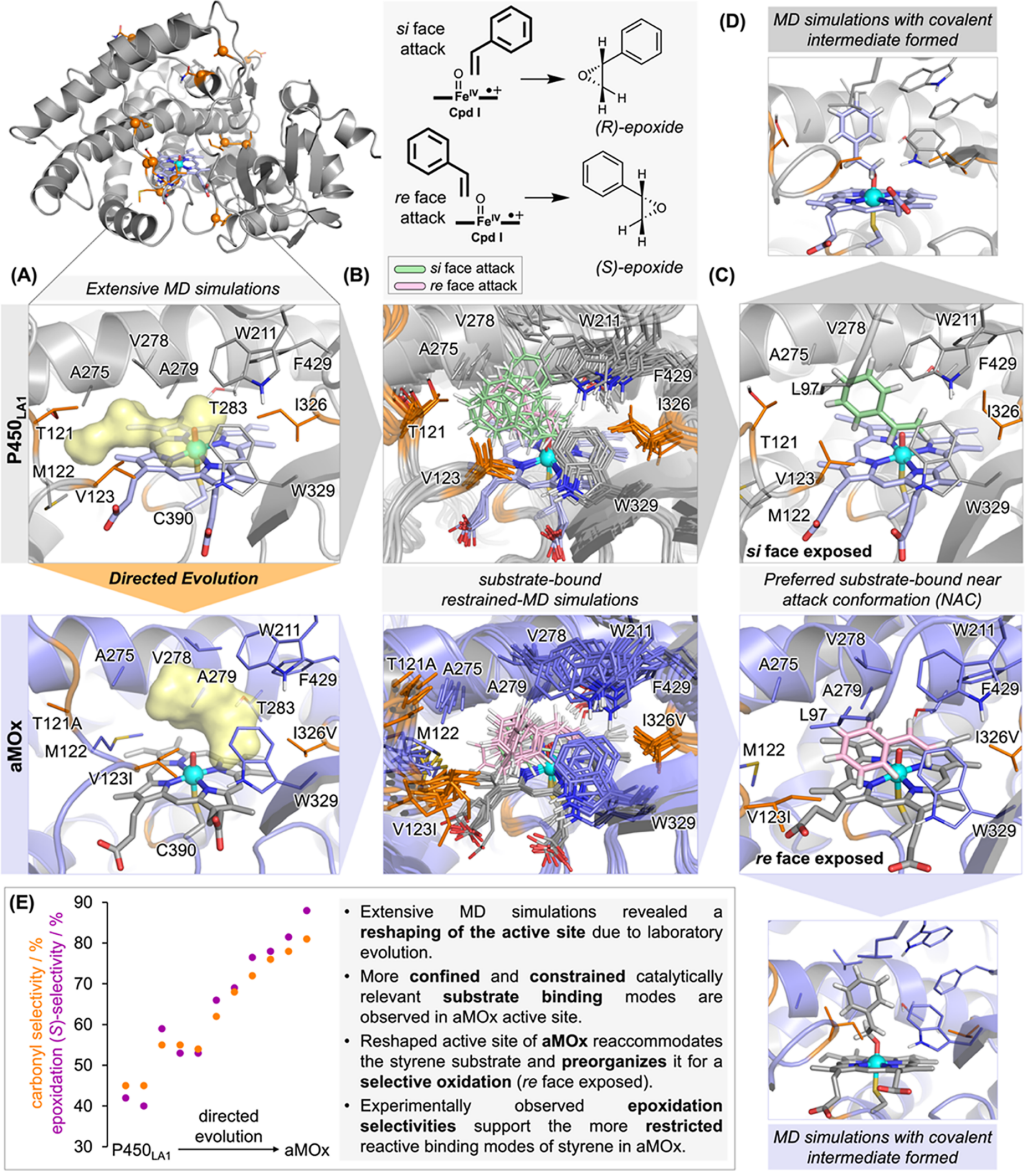
Impact of the directed evolution experiment on the enzyme active site. (A) Homology model was generated for wildtype P450_LA1_ (56% sequence identity with the template, PDB: 6GII) and aMOx (12 mutations with respect to the wildtype). The homology model was refined by extensive molecular dynamics (MD) simulations (5.0 μs of the accumulated simulation time). Positions mutated along directed evolution are shown as orange balls and stick representation. The active site accessible volume is represented by a yellow surface in the most populated cluster characterized from *holo* state MD simulations of wildtype P450_LA1_ and aMOx enzymes (see also Figure S7). (B) Overlay of 10 snapshots of reactive binding modes characterized from restrained-MD simulations of the bound styrene in wildtype P450_LA1_ and aMOx enzymes (snapshots were arbitrarily selected from intervals of 50 ns from 500 ns restrained-MD trajectories). Green- and pink-colored styrene substrates represent *si* face (*R*-epoxide) and *re* face (*S*-epoxide) binding poses, respectively (see also Figures S10 and S11). Relevant active site residues are shown in sticks, and mutated positions are highlighted in orange. (C) Preferential NACs of styrene bound in wildtype P450_LA1_ and aMOx characterized from restrained-MD simulations. (D) Representative binding mode explored by the covalent intermediate **Int1** when formed in wildtype P450_LA1_ and aMOx active sites observed from MD simulations (see also Figure S13). (E) Enantioselectivity in the epoxidation as a function of directed evolution. Interestingly, the enantioselectivity of the epoxidation changes during evolution, stepwise in analogy to the aMOx selectivity. While wildtype P450_LA1_ generates preferably the (*R*)-enantiomer (60:40 of *R*/*S*), epoxidation gets more and more (*S*)-selective in the course of directed evolution and reaches a ratio of 14:86 (*R*/*S*) for aMOx.

The carbonyl-selective biocatalyst (aMOx) was evolved in ten rounds of directed evolution from a cytochrome P450 enzyme (P450_LA1_).^26^ While P450_LA1_ oxidized styrene with low activity (100 TTN) and 55% epoxide selectivity, aMOx oxidized styrenes with high activity (up to 4500 TTN) and chemoselectivity (up to 94% carbonyl product). During laboratory evolution, 12 mutations were introduced. Most of these mutations contribute to change chemoselectivity (Figure 4A), and the mutations scatter over the heme domain of the protein.

To understand how aMOx controls chemoselectivity, we computationally modeled both wildtype P450_LA1_, the evolved aMOx variant and also the intermediate variant P7 obtained in the 4^th^ round of the laboratory evolution.^26^ Since no protein crystal structure is available for P450_LA1_, computational homology models were generated for all the studied enzymes based on a recently solved structure of P450_TT_ (PDB: 6GII) that has 56% of sequence identity for the heme domain (see SI for details). The models were further refined by performing long-timescale molecular dynamics (MD) simulations in the *holo* state (5 replicas of 1.0 μs each, a total of 5.0 μs of the accumulated simulation time). Analysis and comparison of wildtype P450_LA1_ and aMOx active sites revealed that the introduced mutations completely reshaped and narrowed the substrate binding pocket (Figures 4A, and S7). The introduced mutations generated a confined environment that largely reduced the accessible styrene substrate conformations, as confirmed by substrate-bound simulations (Figures 4B, and S10 and S11). Docking calculations were performed on the most representative structures obtained from *holo* state MD simulations after clustering analysis based on protein backbone RMSD, for wildtype P450_LA1_, aMOx and P7 enzymes. Docking predictions were then used as starting points for extensive restrained-MD simulations, in which the distance between the center of mass of the alkene in styrene (defined by C1 and C2 atoms) and the oxygen atom from Cpd I was kept restrained during the MD simulation (5 replicas of 500 ns each, a total of 2,500 ns of the restrained-MD simulation time for each system, see computational details). This allowed us to explore catalytically relevant binding poses, where the substrate arranges in near attack conformations (NACs) to carry out the oxidation reaction, largely refining the docking predictions and preventing undesired unbinding events during the simulations. These simulations showed that P450_LA1_ can orient styrene toward the iron-oxo species, exposing both enantiotopic faces (*re* and *si* face), with a preference for *si* face exposure (Figures 4B,C and S10). In contrast, aMOx binds styrene in a reactive mode that preferentially exposes the *re* face of the alkene (Figures 4B,C and S11). Consequently, restrained-MD simulations qualitatively predict an (*R*)-selective epoxide formation from the P450_LA1_ wildtype, while the epoxide formation as a side product in the aMOx variant is predicted to be (*S*)-selective due to the reshaped active site that reaccommodates the styrene substrate and preorganizes it for a selective oxidation.

An intermediate scenario is found for the P7 variant, in which styrene can effectively explore catalytically relevant binding modes where the *re* face of the alkene is exposed to the iron-oxo species more frequently than in the wildtype (Figure S12). This observation is in line with the shift in the epoxidation selectivity observed along the evolutionary pathway due to the active site reshaping.

To find support for the computationally identified reshaping of the active site and its impact on the preferential substrate binding and enzyme selectivity, we experimentally analyzed the enantioselectivity in the alkene epoxidation reaction along the course of the directed evolution pathway (Figure 4E). The P450_LA1_ wildtype exhibits a slight preference for the formation of the (*R*)-epoxide enantiomer (60:40 *R*/*S*), which qualitatively agrees with the reactive binding modes characterized from computational modeling. Interestingly, the enantiomeric ratio gradually increased toward (*S*)-epoxide formation during evolution. Although the evolved aMOx enzyme generated the epoxide only in low amounts, this byproduct is obtained with high (*S*)-enantioselectivity (Figure 4E). Formation of the (*S*)-epoxide with aMOx is consistent with the preferred *re* face attack observed computationally. This gradual increase in enantioselectivity during directed evolution supports the more restricted reactive binding modes of styrene observed in the computational models for the final evolved aMOx variant. Optimized substrate binding is also supported by UV/Vis spectroscopy, which revealed a 1.4-fold smaller dissociation constant between styrene and aMOx, compared to the P450_LA1_ wildtype (Figure S36). We also found an increase in the coupling efficiency from 6.5 to 26% during enzyme evolution, which supports an optimized substrate binding (Figure S38). As hydrogen peroxide formation is not observed in significant amounts, uncoupling might proceed via the oxidase shunt rather than the peroxide shunt. The kinetic parameters for both enzyme variants showed a 1.6-fold decrease in *K*_M_ and 6.8-fold increase in *k*_cat_, as a result of directed evolution (Figure S37). The evolved aMOx enzyme operates with a catalytic efficiency of 2.0 × 10^3^ M^–1^ s^–1^, which is an order of magnitude higher than that of the P450_LA1_ wildtype and in the range of the average efficiencies of natural enzymes (10^3^ to 10^6^ M^–1^ s^–1^).^50^ Taking together, experimental observations and computational modeling describe that directed evolution increased the confinement in the active site and restricted the accessible, catalytically relevant binding modes of the substrate. This suggests that the accessible conformations of the radical and carbocation intermediates involved in the reaction may also be limited in the more evolved active sites (Figure 4D).

### Computational Modeling of the Enzymatic Reaction Mechanisms Using QM/MM Calculations

We hypothesized that restricting the accessible conformations of the intermediates could strongly influence their reactivity and the chemoselectivity in the oxo–transfer reaction. Thus, we used hybrid quantum mechanics/molecular mechanics calculations (QM/MM)^51^ to model the competing oxidation pathways occurring in both enzymes. Representative structures of the covalent intermediates formed in wildtype P450_LA1_ and aMOx enzymes have been used as the starting point for QM/MM calculations (see Supporting Information for details). These structures were obtained from short MD trajectories (100 ns) with the covalent intermediate bound in wildtype P450_LA1_ and aMOx actives sites (Figure 4D). Initial models for intermediate-bound MD simulations were built from styrene-bound preferential NACs characterized from restrained-MD simulations (Figure 4C).

**Figure 5:**
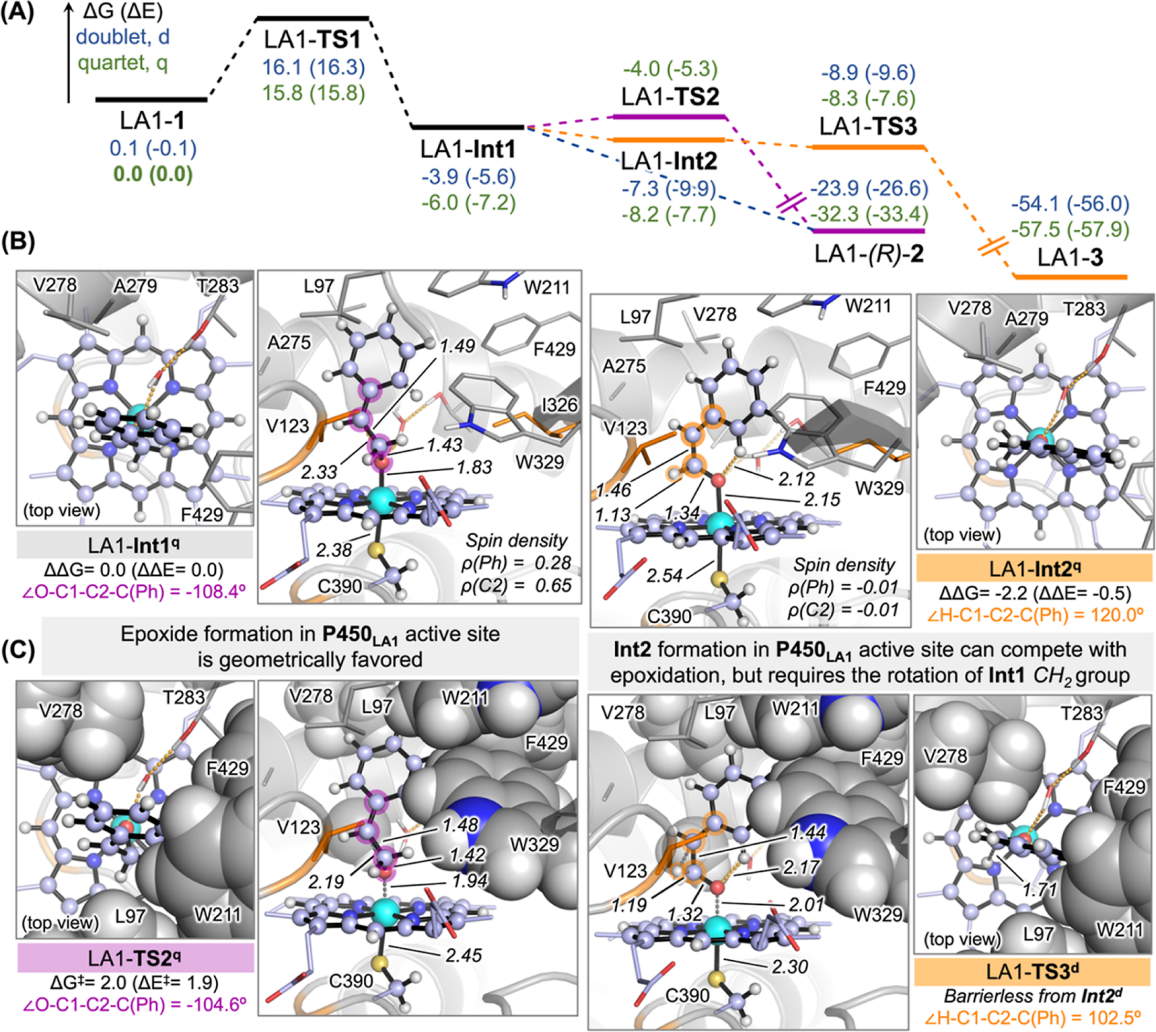
QM/MM calculations on the P450_LA1_-catalyzed reaction pathways. (A) QM/MM-calculated reaction mechanism for the (*S*)-selective epoxidation and carbonyl formation pathways of styrene catalyzed by P450_LA1_ (see also Figure S15). A representative snapshot from intermediate-bound MD simulations with styrene in the preferred reactive *si* face binding pose is used as the starting point (see Figure 4D). Relative Gibbs energies and electronic energies (Δ*G* and Δ*E*, respectively) are shown in blue for the doublet (d) electronic state and green for the quartet (q). Lowest-energy QM/MM-optimized structures for key (B) intermediates and (C) transition states are shown. Space-filling representations for key residues are used to highlight important steric constraints occurring in the active site. Energies, distances, angles, and spin density values are given in kcal·mol^–1^, angstroms (Å), degrees (°), and a.u., respectively. The QM/MM-calculated mechanism based on a *re* face NAC of styrene in the P450_LA1_ active site is reported in Figure S14.

In the P450_LA1_-catalyzed system, the preferential NAC of the substrate corresponds to the *si* face ((*R*)-epoxide forming) binding mode of the styrene substrate (Figure 4C). The alternative *re* face binding mode of styrene, which is accessible in wildtype P450_LA1_, has also been considered in the QM/MM modeling, finding similar trends (see SI Figure S14).^52^ The computed QM/MM reaction profile for the P450_LA1_-catalyzed reaction (Figure 5) compares well to the DFT-modeled enzyme-free truncated model system (Figure 2), with the formation of the first C1–O bond (LA1-**TS1**) being the rate-limiting step in both doublet and quartet electronic states (Figure 5A). Interestingly and in contrast to the truncated model system, the radical intermediate (LA1-**Int1**) can be optimized as a minimum on the PES in both electronic states. This indicates that confinement favors the stabilization of the radical species from a potential energy perspective (Figure 5B). Once formed, the radical intermediate LA1-**Int1** can lead to the epoxide product through a low-energy transition state (LA1-**TS2**, Δ*G*^‡^ = 2.0 kcal·mol^–1^) in the quartet state and through a barrierless step in the doublet state (Figure 5C). In comparison, the carbonyl pathway is also accessible from radical LA1-**Int1** in both electronic states, where carbocation intermediate LA1-**Int2** can be formed slightly downhill after a geometric rearrangement, similar to the enzyme-free model, leading to the generation of the carbonyl product (**3**) through a barrierless transition state (LA1-**TS3**, see Figure 5B,C). Due to the steric constraints imposed by the active site cavity, the optimized radical LA1-**Int1** and carbocation LA1-**Int2** intermediates have the phenyl ring occupying the same position in the enzyme active site. The main geometric difference between these two intermediates is the rotation of the methylene group covalently attached to the *O*-atom, which ensures effective stabilization of the carbocation intermediate, as described above (Figure 5B). The geometric change required to distort LA1-**Int1** into a LA1-**Int2**-like geometry has been explored using relaxed scan calculations (Figure S18), which indicated that this geometric rearrangement is very low in energy (Δ*E* ca. 3.0 kcal·mol^–1^), similar to what is observed for the enzyme-free model.

Based on these results, QM/MM calculations suggest that aldehyde side product formation observed in P450_LA1_ may be attributed to the confinement in the enzyme active site that helps to stabilize the radical intermediate LA1-**Int1**. However, although the carbonyl pathway is energetically downhill and highly favorable, the epoxide (**2**) can still be formed as the major product, favored by the existing “dynamic match” between the two C–O bond forming coordinates. The carbonyl pathway is disfavored by the geometric rearrangement required to form carbocation LA1-**Int2** from LA1-**Int1** and the fact that epoxide formation from LA1-**Int1** is barrierless (doublet) or has a very low-energy barrier (quartet LA1-**TS2**). Consequently, a higher control over the accessible conformations of the nascent radical intermediate would be required to selectively promote the carbonyl pathway.

**Figure 6:**
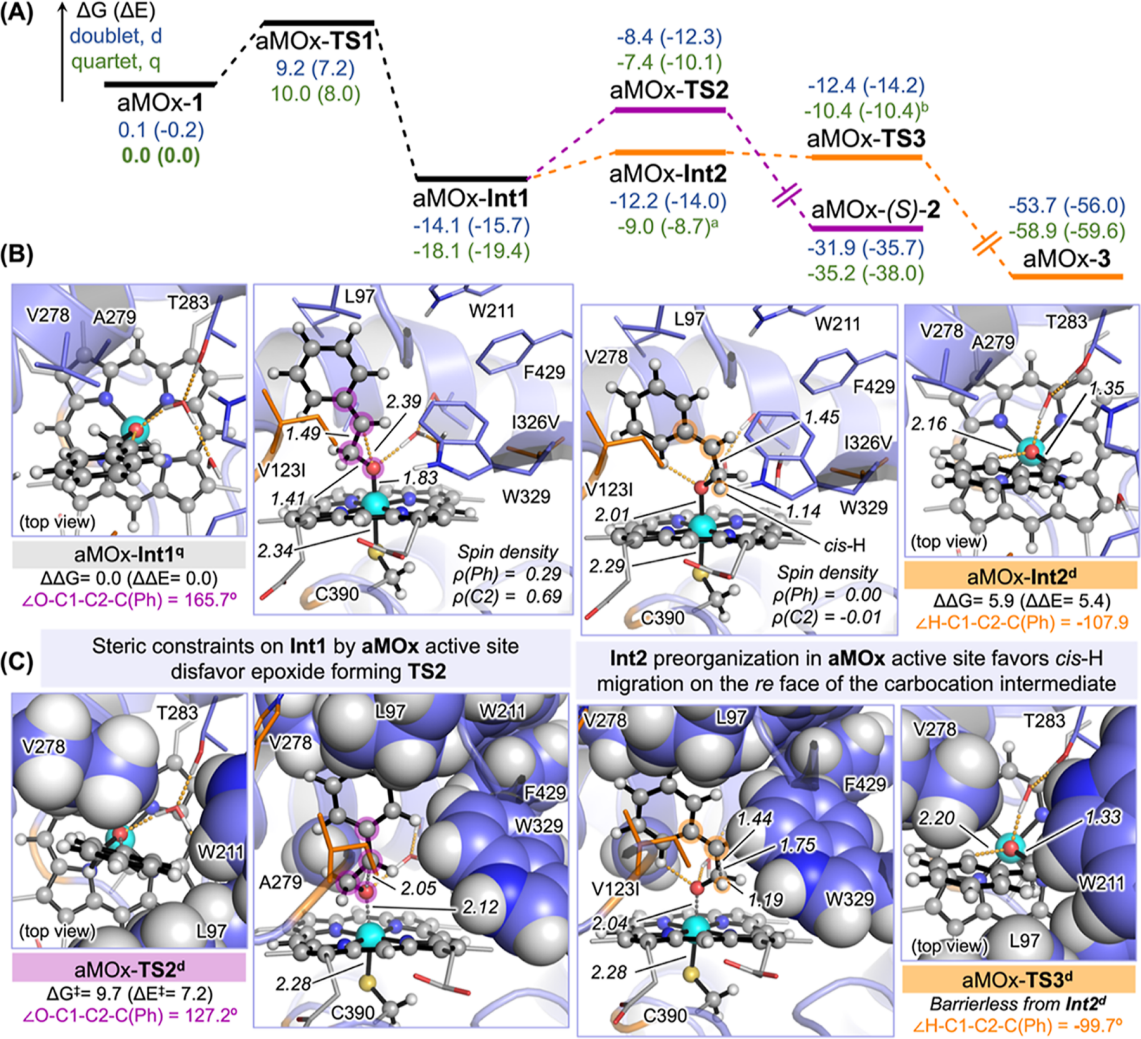
QM/MM calculations on aMOx-catalyzed reaction pathways. (A) QM/MM-calculated reaction mechanism for the (*S*)-selective epoxidation and carbonyl formation pathways of styrene catalyzed by the aMOx variant (see also Figure S19). A representative snapshot from intermediate-bound MD simulations with styrene in the preferred reactive *re* face binding pose is used as the starting point (see Figure 4D). Relative Gibbs energies and electronic energies (Δ*G* and Δ*E*, respectively) are shown in blue for doublet (d) electronic state and green for quartet (q). Lowest-energy QM/MM-optimized structures for key (B) intermediates and (C) transition states are shown. Space-filling representations for key residues are used to highlight important steric constraints occurring in the active site. Energies, distances, angles, and spin density values are given in kcal·mol^–1^, angstroms (Å), degrees (°), and a.u., respectively. The QM/MM-calculated mechanism based on the minor explored *si* face NAC of styrene in the aMOx active site is reported in Figure S23. ^a^ Structure optimized with C2–*cis*-H, C2–*trans*-H, and *cis*-H–*trans*-H distances frozen. Distance values are taken from the optimized structure in the doublet state. Frequency calculations showed that the optimized structure has all positive frequencies. ^b^ The TS structure optimized with C2–*cis*-H, C1–*cis*-H, and O–C1 distances frozen. Distance values are taken from the optimized structure in the doublet state. Frequency calculations showed that all frequencies of the optimized structure are positive except one, which corresponds to the H-migration coordinate.

In the next step, we calculated the competing carbonyl forming and epoxidation reaction pathways catalyzed by the evolved aMOx biocatalyst (Figure 6). These QM/MM calculations revealed significant differences compared to the truncated model (Figure 2) and P450_LA1_ (Figure 5). QM/MM calculations were carried out considering the preferred reactive binding mode of styrene in the aMOx active site (Figure 4), where the *re* face of the alkene is exposed to the iron-oxo active species (see Supporting InformationFigure S23 for calculations involving the alternative minor *si* face binding mode,^52^ which exhibit significantly higher reaction barriers and thus making it significantly less reactive). These calculations showed that similar to the wildtype P450_LA1_ system, both doublet and quartet radical intermediates aMOx-**Int1** can be optimized and characterized as minima on the PES (Figure 6A). Additionally, calculations described a significant barrier for the second C2–O bond formation (aMOx-**TS2**, Figure 6A) from the radical intermediate aMOx-**Int1** in both doublet or quartet electronic states. The estimated barrier for three-membered ring formation (aMOx-**TS2**^**q**^ Δ*G*^‡^ = 10.7 kcal·mol^–1^ and aMOx-**TS2**^**d**^ Δ*G*^‡^ = 9.7 kcal·mol^–1^, from the lowest-energy quartet aMOx-**Int1**^**q**^, Figure 6) is significantly higher as compared to the ones estimated for the enzyme-free model (Figure 2) and wildtype enzyme (Figure 5). This observation presents a scenario in which the activated radical intermediate is stabilized by the active site of aMOx due to confinement and steric interactions increasing its lifetime (Figure 6A,B). This leads to an effective thermal equilibration, loss of the excess of kinetic energy, and vibrational energy redistribution. The equilibrated radical intermediate (aMOx-**Int1**^**q**^) can thus follow the lowest-energy reaction pathway to generate the carbonyl product, through a rotation along the ∠O–C1–C2–C(Ph) dihedral angle (aMOx-**TS-rotation**^**q**^, Δ*G*^‡^ = 7.2 kcal·mol^–1^, Figure S22). This is a significant rotational barrier as compared to the enzyme-free system and wildtype P450_LA1_, which is attributed to the higher confinement and packing in the evolved aMOx active site. Although higher, this rotational barrier that converts aMOx-**Int1** into an aMOx-**Int2**-like geometry is significantly lower than the epoxide-forming barrier aMOx-**TS2** (ΔΔG ca. −2.5 and −3.5 kcal·mol^–1^ for doublet and quartet, respectively; Figure S22). QM/MM calculations described that the carbocation intermediate formation is uphill (aMOx-**Int2**^**d**^ ΔΔ*G* = 5.9 and aMOx-**Int2**^**q**^ ΔΔ*G* = 9.1 kcal·mol^–1^, from the lowest-energy aMOx-**Int1**^**q**^, Figures 6 and S22), but these carbocation intermediates are still lower in energy than the corresponding epoxide-forming transition states (Figure 6A). 1,2-Hydride migration from carbocation aMOx-**Int2** is found to be barrierless in both electronic states (Figure 6A,C), with the doublet electronic state pathway being lower in energy than the quartet electronic state pathway. Tight active site packing in the aMOx variant stabilizes the radical intermediate by holding the aromatic ring in a specific position, establishing hydrophobic interactions with active site residues L97, V123I, V278, and A279 (Figure 6B,C). Similar to wildtype P450_LA1_, formation of the carbocation involves the rotation of the methylene–oxygen group to ensure stereoelectronic stabilization of the carbocation, while the aromatic ring is held at the same position (Figure 6B).

Thus, aMOx can override the intrinsic dynamic preferences for epoxidation by imposing steric constraints to the formed radical intermediate, allowing its thermal equilibration. These steric constraints disfavor the epoxidation **TS2** with respect to the carbocation formation and hydride migration transition state **TS3**. Carbonyl formation is thus energetically more favorable than epoxidation, which explains the chemoselectivity achieved by the evolved aMOx enzyme.

Finally, we evaluated the role of the local electric field (LEF) generated in the active site cavity of wildtype P450_LA1_ and aMOx in the stabilization of the carbocation intermediate using the QM/MM optimized structures of LA1-**Int1**^**q**^ and aMOx-**Int1**^**q**^, respectively. Recently, it was shown that LEFs generated by enzyme scaffolds could also play a significant role in dictating the reactivity of heme^53−57^ and non-heme^58^ iron proteins, in addition to the steric control that the protein exerts by constraining the accessible conformations for the substrate. Interestingly, our calculations indicated that the LEF generated in the active site cavity in both P450_LA1_ and aMOx variants are equivalent in both the direction and strength (Figure S25). The LEF is oriented along the Fe–O axis and follows this direction (from Fe to O direction). Therefore, these calculations showed that evolution did not significantly altered the LEF exerted by the protein scaffold.

In order to assess the impact that this LEF generated by the protein might have on the stabilization of the carbocation intermediate, we carried out additional DFT calculations using computational truncated models (see Figures S26 and S27). Our calculations showed that the generated LEF in the aMOx active site largely stabilizes the carbocation **Int2** as compared to the radical intermediate **Int1**. Additionally, calculations indicated that the generated LEF has a suitable orientation and direction to favor the formation and stabilization of the carbocation **Int2** (Figures S28 and S29). Other orientations and directions of the LEF would lead to much less or insignificant stabilization of the carbocation intermediate **Int2** as compared to the radical.

These results support the conclusion that the existing electrostatic preorganization of the wildtype P450_LA1_ active site favors the formation of the carbocation intermediate. This electrostatic preorganization is required, together with the strong conformational control achieved by the aMOx variant active site upon evolution, to direct the reaction toward the carbonyl formation pathway.

### Selective Hydride Migration: Synergies between Computational Modeling and Enzymatic Conversion of Deuterium-Labeled Styrenes

QM/MM calculations do not only reveal that the protein environment offers a high degree of conformational control over the reactive intermediates but also propose high stereoselectivity in the hydride migration. The tight conformational control of aMOx primes the *cis*-H atom of the styrene substrate for selective migration on the *re* face of the carbocation intermediate through the lowest-energy transition state aMOx-**TS3** (Figure 6B,C and Figures S19–S21). This can be rationalized by the oxygen attack from the *re* face and rotation of the C1–C2 bond to generate a stabilized carbocation intermediate. In this conformation, the C–H bond (*cis*-H) and the empty p-orbital of the benzylic carbocation intermediate are geometrically preorganized for selective hydride migration on the *re* face (Figures 6B, and S20 and S22).

**Figure 7:**
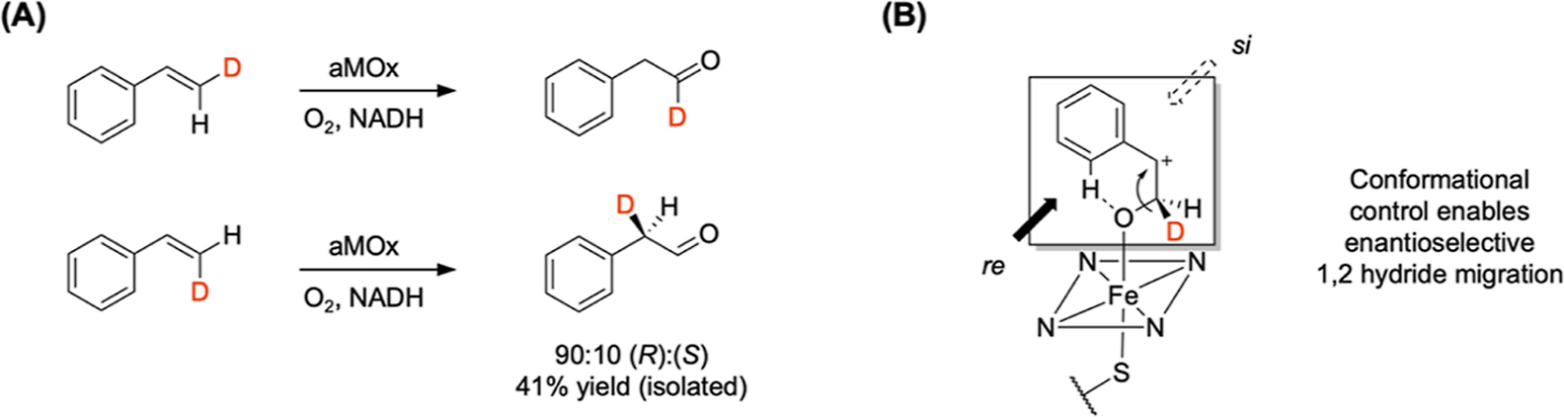
(A) Determination of the *cis*/*trans*- and stereoselectivity of the 1,2-hydride migration with deuterium-labeled styrenes as substrates. (B) Conformational control of the carbocation intermediate allows an enantioselective 1,2-migration, yielding the aldehyde as the (*R*)-enantiomer predominantly.

To validate the predicted *cis*-selective and enantioselective hydride migration obtained from QM/MM calculations and to further support the *modus operandi* of the carbonyl-selective aMOx enzyme, we synthesized deuterium-labeled *cis*- and *trans*-styrene-(β)-*d*, according to the literature.^59,60^ Both substrates were independently converted by the aMOx enzyme. To avoid loss of the deuterium label by keto–enol tautomerism of the aldehyde product, the reactions were performed in an enzyme cascade, together with a phenylacetaldehyde reductase (PAR). We used mass spectrometry to characterize the position of the deuterium label in the products (Figures S30 and S31). While hydride migration could be observed with the deuterium label at the *trans*-position, deuteride migration occurred with the *cis*-labeled styrene (Figure 7). The experimental data are in full agreement with the computational investigations and demonstrate that aMOx catalyzes a *cis*-selective 1,2-migration.

Finally, we aimed to analyze the enantioselectivity during the 1,2-hydride migration. Computational and recent experimental studies^26^ suggested that aMOx can control the enantioselectivity in the 1,2-hydride migration. According to our QM/MM calculations, the *cis*-hydride migrates selectively on the *re* face of the prochiral substrate due to conformational restraints imposed by the active site of the enzyme and the reactive binding pose that the substrate and intermediates adopt (see above). To support this finding with experimental evidences, we performed a preparative scale reaction with *cis*-styrene-(β)-d as a substrate and aMOx as a catalyst (see SI for details). The upscaling was performed in a cascade reaction with PAR to avoid racemization of the aldehyde via keto–enol tautomerism. The deuterium-labeled alcohol was isolated with a yield of 41% and converted to the Mosher ester. ^1^H NMR analysis of the ester revealed that the formed stereocenter is predominantly (*R*)-configurated with a selectivity of (*R*):(*S*) > 90:10 (Figures S33 and S34). Consequently, the 1,2-hydride/deuteride migration occurs selectively on the *re* side, which is in full agreement with computational models and predictions. It is currently not clear whether the <10% (*S*)-enantiomer derives from racemization of the aldehyde or by unselective 1,2-migration.

Overall, this strengthens our findings that aMOx is the first catalyst capable of performing enantioselective alkene to carbonyl oxidation. aMOx achieves this by a catalytic enantioselective 1,2-hydride migration, which has to the best of our knowledge not been reported so far. Catalytic enantioselective 1,2-migrations on prochiral carbocations are very challenging and up to now limited to migration of alkyl or phenyl groups, often in ring-strain releasing processes.^61−63^ Stereoselective 1,2-migrations of hydrides are known but typically restricted to chiral, cyclic molecules. In these substrate-controlled processes, the stereochemical information is already encoded in the structure of the starting material.^64^ In contrast, aMOx achieves enantioselectivity by catalyst-control, a feature that can potentially be used to control many other molecular rearrangement reactions^65^ involving reactive intermediates. Overall, this *cis*-selective and enantioselective 1,2-hydride migration is not only a remarkable catalyst-controlled process, but also supports the computational predictions on the enzymatic mechanism.

## Conclusions

A combination of multiscale computational methods has been employed to model the oxidation of styrene by heme iron-oxo active species in the absence of any enzyme scaffold and in an enzymatic environment specifically evolved to carry out a carbonyl-selective oxidation reaction. DFT calculations for styrene as a substrate indicated that the epoxidation pathway and the direct oxidation to an aldehyde (carbonyl forming pathway) are energetically accessible, exhibiting low intrinsic barriers, and diverge after the first C1–O bond formation that leads to a covalent radical intermediate. DFT calculations and direct QCTs showed that the *fleeting* radical intermediate is formed with a large excess of kinetic energy. In addition, these calculations revealed a strong coupling between the reaction coordinate that describes this first C1–O bond formation with the coordinate that promotes the second C2–O bond formation. These factors intrinsically favor the epoxide product formation over the aldehyde formation in the enzyme-free model system. However, when the reaction takes place in an enzymatic framework, confinement helps in dissipating the excess of energy of the radical intermediate, allowing its thermal equilibration. Furthermore, electrostatic preorganization of the enzyme active site also stabilizes the formation of the carbocation intermediate. In addition, QM/MM calculations described that the evolved aMOx enzyme imposes steric constraints to the reactive intermediates when formed in its active site, which limits their accessible conformations. This translates into disfavoring the transition state that leads to the epoxide product via second C2–O bond formation, while allowing a *cis*- and enantioselective 1,2-hydride migration to preferentially generate the aldehyde product. Consequently, intrinsic dynamic preferences and the important “dynamic match” that strongly favors epoxidation are overridden by the evolved aMOx enzyme. These computational predictions were further supported by experimental investigations using deuterated styrene substrates, demonstrating that confinement achieved by the evolved aMOx enzyme also controls the stereoselectivity in the hydride-migration step.

Our results demonstrate that confinement^39^ is essential to alter and control the intrinsic dynamic behavior of these highly reactive *fleeting* intermediates formed over the course of these diverging oxidation pathways. The dynamic behavior of such intermediates which indeed can be (pro-)chiral^52^ has a large impact on the chemoselectivity of the reaction. Mechanistic insights obtained in this work provide useful guidance that may help to further expand this new biocatalytic direct oxidation of alkenes to carbonyl compounds toward more challenging substrates, including internal alkenes^66^ or unactivated, aliphatic alkenes. We envision that other biological or abiological enzyme-catalyzed reactions might use similar mechanisms to outcompete intrinsic dynamic effects of reactive intermediates to access alternative catalytic cycles that are challenging to achieve otherwise. We expect that our study paves the way toward the design of new enzyme-catalyzed reactions that exploit these features.

## Supporting Information Available

Complete description of the computational methods and protocols, experimental methods and additional computational and experimental results, and energies and cartesian coordinates of all optimized species (PDF)
Movies of exemplary direct QCT (ZIP)
