# Supplementary material for: Enzymatic Control over Reactive Intermediates Enables Direct Oxidation of Alkenes to Carbonyls by a P450 Iron-Oxo Species
Source: J Am Chem Soc. 2022 Aug 23;144(35):15954–68. doi: 10.1021/jacs.2c02567 (PMC9460782; doi:10.1021/jacs.2c02567)
Supplement: Supplementary file 1 — ja2c02567_si_001.pdf [file ja2c02567_si_001.pdf]

Supplementary information

Enzymatic control over reactive intermediates enables direct oxidation of alkenes to carbonyls by a P450 iron-oxo species

Authors: Jordi Soler,[a,‡] Sebastian Gergel,[b,‡] Cindy Klaus,[b] Stephan C. Hammer,[b,*] Marc Garcia-Borràs[a,*]

Affiliations:

[a] Institut de Química Computacional i Catàlisi (IQCC) and Departament de Química, Universitat de Girona, Carrer Maria Aurèlia Capmany 69, Girona 17003, Catalonia, Spain
[b] Chair of Organic Chemistry and Biocatalysis, Faculty of Chemistry, Bielefeld University, Universitätsstraße 25, 33615 Bielefeld, Germany

‡ both authors contributed equally
* Email: marc.garcia@udg.edu, stephan.hammer@uni-bielefeld.de

Table of Contents

A. Computational Modelling Section 3

I. Computational Methods 3

II. Exploration of the intrinsic oxidation mechanisms using DFT calculations on an enzyme-free computational trucated model. 8

III. Quasiclassical direct dynamics trajectory simulations 22

IV. Computational modelling of the enzyme-substrate bound complexes 26

V. Computational modelling of the enzymatic reaction mechanisms using QM/MM calculations 43

VI. Local Electric Field characterization in the active site cavity and its impact on the reactive intermediates 99

VII. Absolute energies of the characterized stationary points 122

B. Experimental Section 129

I. Materials and Methods 129

II. General procedures 133

III. Chemical synthesis of *d*-labeled styrenes 136

IV. Determination of the migration tendency (*cis* vs. *trans*) 139

V. MS spectra from isotopic labeling experiment 142

VI. Chemical and enzymatic synthesis of isotopically labeled 2-phenylethan-2-*d*-1-ol 144

VII. NMR-analysis to determine the enantioselectivity 147

VIII. Enantioselectivity in epoxidation as a function of evolution 153

IX. UV/Vis spectroscopic analysis of substrate binding 154
X. Michealis-Menten kinetics 155
XI. Coupling efficiencies 156
C. Optimized structures (cartesian coordinates) of characterized stationary points 157
D. References 211

A. Computational Modelling Section

I. Computational Methods

Density Functional Theory (DFT) calculations

All Density Functional Theory (DFT) calculations were performed using Gaussian09 software package.[1] A computational truncated model has been used [Fe=O(Por)(SCH_3)(**1**)], which includes: the iron-oxo active species of Cpd I (Fe=O), a porphyrin pyrrole core (Por), a methanethiolate group to mimic the Cys axial ligand, and the styrene (**1**) substrate. The resulting model has a neutral total charge and two different electronic states have been considered: doublet and quartet, both energetically accessible. The unrestricted hybrid (U)B3LYP[2–4] functional was used with an *ultrafine* integration grid,[5] and including the CPCM polarizable conductor model (dichloromethane, ε = 8.9)[6,7] to have an estimation of the dielectric permittivity in the enzyme active site.[8–10] 6-31G(d) basis set was used for all atoms but Fe, where SDD basis set and related SDD pseudopotential were employed. All optimized stationary points were characterized as minima using frequency calculations, including transition states which show a single imaginary frequency that describes the corresponding reaction coordinate. IRC calculations were performed to ensure that optimized transition states connect the expected reactants and products. Enthalpies and entropies were obtained at 1 atm and 298.15 K. Enthalpy calculations were corrected using the harmonic oscillator approximation, as discussed by Truhlar and co-workers,[11,12] by increasing all frequencies below 100 cm^{-1} to 100 cm^{-1} using Goodvibes v.3.0.1 python script.[13] Single point energy calculations were carried out with the previously described DFT functional (U)B3LYP, with an ultrafine grid and CPCM dichloromethane conductor model), Def2TZVP basis set for all atoms, and including Empirical Grimme D3 dispersion corrections with Becke-Johnson (GD3BJ) damping.[14] The Shaik group and others have demonstrated that DFT calculations based on B3LYP functional provide qualitative good descriptions and relative energy differences of species involving thiolate model of cytochrome P450 compound I as compared to CASSCF and CASPT2 higher level methods.[15–18] Calculations in the presence of an oriented electric field (optimizations and/or single point calculations) were performed using the Gaussian09 IOp(3/14= -6) to define the components of the electric field vector, including the *nosymm* keyword. Gaussian09 in combination with the code developed by Harvey et al.[21] and the easyMECP python script.[22]
Optimized structures showed in figures were rendered using CYLview,[19] and molecular orbitals and spin density isosurfaces were represented using PyMOL,[20] using Gaussian09 generated cube files. The minimum energy crossing points (MECP) were optimized and evaluated using

Direct quasiclassical dynamics trajectory simulations

Direct quasiclassical trajectory (QCT) simulations were performed in vacuum at 298.15 K using the same truncated model early described, [Fe=O(Por)(SCH3)(**1**)], which includes: the iron-oxo active species of Cpd I (Fe=O), a porphyrin pyrrole core (Por), a methanethiolate group to mimic the Cys axial ligand (SCH_3), and the styrene (**1**) substrate. Quantum mechanical forces and normal-mode sampling for DFT optimized **TS1** structures were obtained with Gaussian09[1] using the unrestricted hybrid (U)B3LYP[2–4] functional with an *ultrafine* integration grid.[5] 6-31G(d) basis set was used for all atoms but Fe, where SDD basis set and related SDD pseudopotential were employed. QCT were initialized in the vicinity of the rate-limiting transition state **TS1** leading to O-C1 bond formation with the normal mode sampling method. The sampled **TS1** geometries constitute a quantum mechanical Boltzmann distribution.[23,24] Zero-point energy and thermal energy were added in real normal vibration modes on each sampled **TS1** geometry. Both doublet (d) and quartet (q) electronic states were considered by performing 20 independent trajectories for each electronic state (40 trajectories in total). The QCT were carried out using the velocity-Verlet algorithm as implemented in Singleton's Progdyn program,[25–27] and were propagated forward and reverse for 600 fs with a 1 fs step size in each direction. A trajectory was terminated when either the simulation reaches 600 fs in one direction or the geometric criterion for reactant (**1**), epoxide (**2**), or carbonyl (**3**) formation is fulfilled.

Homology modelling

The initial homology model for the heme domain of $P450_{LA1}$ enzyme was generated using Swiss-Model[28] and taking as a template the recently solved structure of $P450_{TT}$, which has 56% of sequence identity for the heme domain (PDB: 6GII) and 60% for the full protein chain (PDB: 6KBH). The initial homology model was further refined by performing extensive MD simulations, 5 independent replicas of 1,000 ns (1 μs) each, accumulating a total of 5 μs of simulation time. Analysis of the conformational landscape of the enzyme explored during the accumulated simulation time was carried out performing clustering in terms of protein backbone Cα RMSD, using Cpptraj[29] module from Ambertools. A representative structure of the most populated cluster was selected as starting point for further modelling. This structure was also used as a starting point to generate the intermediate P7 variant and the evolved aMOx variant. To do so, 5 (T121A, N201K, N209S, Y385H, E418G) and 12 (A103L, M118L, R120H, T121A, V123I, N201K, N209S, I326V, V327M, Y385V, M391L, E418G) additional mutations were introduced using RosettaDesign[30] for P7 and aMOx variants, respectively. The models obtained from RosettaDesign were refined by extensive MD simulations, 5 independent replicas of 1,000 ns (1 μs) each, accumulating a total of 5 μs of simulation time per variant. Equivalent clustering analysis as described for WT $P450_{LA1}$ system was carried out to obtaining an accurate P7 and aMOx structure that were used for further modelling.

Molecular Dynamics simulations

Molecular Dynamics (MD) simulations in explicit water were performed using the AMBER18 package.[31,32] Parameters for the heme compound I (Cpd I) and the axial Cys were taken from reference[33]. The enzyme variants ($P450_{LA1}$, P7, and aMOx) were solvated in a pre-equilibrated cubic box with a 10-Å buffer of TIP3P[34] water molecules using the AMBER18 leap module, resulting in the addition of ~16,500 solvent molecules. Explicit counterions (Na^+ or Cl^-) were introduced to neutralize the system. All subsequent calculations were done using the Stony Brook modification of the Amber14 force field (*ff*14SB).[35] A two-stage geometry optimization approach was used. The first stage minimizes the positions of solvent molecules and ions imposing positional restraints on solute by a harmonic potential with a force constant of 500 kcal mol^{-1} $Å^{-2}$, and the second stage is an unrestrained minimization of all the atoms in the simulation cell. The system was gently heated using six 50 ps steps, incrementing the temperature by 50 K for each step (0–300 K) under constant-volume and periodic-boundary conditions. Water molecules were treated using the SHAKE algorithm, where the angle between the hydrogen atoms was kept fixed. Long-range electrostatic effects were modelled using the particle-mesh-Ewald method.[36] An 8 Å cutoff was applied to Lennard–Jones and electrostatic interactions. Harmonic restraints of 30 kcal·mol^{-1} were applied to the solute, and the Langevin scheme was used to control and equalize the temperature. The time step was kept at 1 fs during the heating stages, allowing potential inhomogeneities to self-adjust. Each system was then equilibrated for 2 ns with a 2 fs time step at a constant pressure of 1 atm and temperature of 300 K without restraints. Once the systems were equilibrated in the NPT ensemble, production trajectories were then run under the NVT ensemble and periodic-boundary conditions. In particular, a total of 5,000 ns (5.0 μs) in the *holo* state were accumulated for $P450_{LA1}$, P7, and aMOx variants: 5 independent replicas of 1,000 ns each (5 x 1 μs for each system). Cpptraj[29] module from Ambertools utilities was used to process and analyze the trajectories, including clusterization analyses. POVME3.0 was used to analyze active site volumes.[37] VMD software was used to visualize MD simulations.[38] Protein structures were rendered using PyMOL.[20]

Docking, substrate-bound and intermediate-bound MD simulations

Parameters for styrene (**1**) substrate were generated within the Antechamber[39] module in AMBER18 package using the general AMBER force field (gaff2),[40] with partial charges set to fit the electrostatic potential generated at the HF/6-31G(d) level by the RESP model.[41] The charges were calculated according to the Merz–Singh–Kollman scheme[42,43] using the Gaussian09 package.[1] The most representative structures from the previous *holo* state simulations were characterized by clustering of the accumulated simulation time, considering the protein backbone RMSD. These structures were used for docking calculations with substrate **1**, which were performed using AutoDock Vina.[44] Docking results were used as starting points for substrate-bound restrained-MD simulations, in which the distance between the center of mass of the alkene in styrene (**1**) substrate (defined by C1 and C2 atoms) and the oxygen atom from Cpd I was kept restrained during the MD simulation (2.8-3.0 and 3.2-3.4 Å, using a 100 kcal·mol^{-1}·$Å^{-2}$ force constant). This allowed to explore catalytically relevant binding poses of the substrate, where it is in a near attack conformation to make the oxidation reaction happen,

largely refining the docking predictions and preventing undesired unbinding events during the simulations. The same protocol previously described for MD simulations was applied. A total of 5 independent replicas of 500 ns of production trajectories were performed for $P450_{LA1}$, P7, and aMOx, accumulating a total of 2.5 μs of substrate restrained-MD simulation time for each system.

Parameters for the covalent radical intermediate-bound structure were generated using the Metal Center Parameter Builder (MCPB.py)[45] in AMBER18 package. The coordination sphere of the metal center includes Cys390, heme cofactor, and the covalent radical intermediate in quartet electronic state (**Int1^{q}**). The same protocol previously described for MD simulations was applied. A total of 2 independent replicas of 500 ns of production trajectory each were accumulated for $P450_{LA1}$ and aMOx, accumulating a total of 1.0 μs of intermediate-bonded MD simulation time for each system. Intermediate-bound MD simulations were used to explore the conformational flexibility of such intermediate when formed in the enzymatic active sites. Representative structures obtained from intermediate-bound simulations that describe the catalytically relevant binding modes characterized from substrate-bound restrained-MD simulations, were used as a starting point for subsequent QM/MM calculations.

Quantum Mechanics / Molecular Mechanics (QM/MM) calculations

Initial structures for QM/MM modelling were selected from intermediate-bound MD simulations on $P450_{LA1}$ and aMOx variant, which describe the catalytically binding modes characterized from substrate-bound restrained-MD simulations. All water molecules and counterions beyond 3 Å from any residue of the protein, cofactors or substrates were removed and the 4 resulting structures had *ca.* 10350 atoms each, respectively.

The QM region included the heme porphyrin pyrrole core, the C390 cysteine sidechain, the iron center, and the styrene molecule (50 QM atoms and 9 H-link atoms). The resulting QM region has a neutral charge and both doublet and quartet energetically accessible electronic states are considered. All residues and water molecules inside a 12 Å shell around the QM region were considered as active (active region), thus resulting in more than 2,600 MM atoms free to move during the optimization procedures.

QM/MM calculations were carried out using the ONIOM[46] approach as implemented in Gaussian09.[1] Geometry optimizations were performed with the hybrid (U)B3LYP[2–4] functional using an *ultrafine* integration grid and with 6-31G(d) basis set on all atoms except for iron, where an SDD basis set and related SDD pseudopotential was used. The MM parameters and MM charges were identical to those used in the intermediate-bound MD simulations. A two-step sequential optimization protocol using QuadMacro[47] optimization algorithm has been used: *i*) a first optimization using a Mechanical Embedding (ME) scheme was initially performed, and once optimized, *ii*) all MM water molecules were kept frozen and a second optimization was performed within the Electrostatic Embedding scheme (EE). This protocol permits to optimize the solvent molecules by including polarization on the QM region without losing control on the microiteration cycles performed during EE optimizations.[48] The use of an electrostatic embedding scheme allows to account for the polarizing effect of the enzyme environment on the QM region. Stationary points were verified as minima or saddle point (transition state) geometries after vibrational frequency analysis, having all frequencies positive

(minima) or only one imaginary frequency (transition states). Thermal corrections were obtained at 1 atm and 298.15 K. Single point energy calculations on the optimized structures were performed at the (U)B3LYP/Def2TZVP theory level within the EE scheme. MolUP VMD extension[49] was used for input preparation and output visualization and PyMOL was used for image rendering.[20]

Local Electric Field (LEF) calculations

QM/MM optimized quartet covalent radical intermediates in both $P450_{LA1}$ and aMOx variants (LA1-**Int1^q^** and aMOx-**Int1^q^**) were used as starting points for calculating the local electric field (LEF) generated in the enzyme active site using TITAN 2.0 software.[50] All 'non-residue' molecules including solvent molecules and counterions, the heme cofactor, the axial C390 sidechain, the Fe-oxo moiety, and the former substrate were removed. This protocol is equivalent to the one used by Alexandrova and co-workers,[51] and accounts for the LEF generated by the protein scaffold in the P450 active site cavity. The atomic charges used were obtained from the *ff*14SB forcefield[35]. The local electric field was calculated at the position where the catalytic oxygen atom (bound to Fe) was placed, since it is centered in the active site cavity. The electric field convention used by TITAN 2.0 is that the direction of the field is defined from positive to negative (i.e. a free positive charge will follow the direction of the electric field vector). This is the general convention for electric field vector representation, despite Gaussian09 package uses the opposite (i.e. negative to positive).[52]

For visual representation of the electric field orientation and direction in the enzyme active site, the units of the computed electric field vector were transformed to Å. To do so, the atomic units of the field were first converted to $V·Å^{-1}$ (1 a.u. = 51.4 $V·Å^{-1}$) and the resulting value was then multiplied by the arbitrary value of 10 $Å^{2}·V^{-1}$ to directly obtain Å. This transformation results into a scaled electric field vector that can be properly represented in the active site of the enzymes. PyMOL software was used for image rendering.[20]

II. Exploration of the intrinsic oxidation mechanisms using DFT calculations on an enzyme-free computational truncated model.

Figure S1: Intrinsic reaction mechanism studies by means of DFT truncated model.

A) Proposed catalytic cycle for epoxidation and anti-Markovnikov oxidation mechanisms catalyzed by P450 enzymes.

B) DFT calculated reaction mechanism for styrene (**1**) oxidation using a truncated computational model based on P450 active site (iron-oxo species coordinated to the porphyrin pyrrole core and a methanethiolate as axial ligand). Quasi-harmonic corrected Gibbs energies (ΔG) and relative electronic energies (ΔE, in parenthesis) of the lowest in energy electronic state (doublet (d) or quartet (q)) are reported. Energy values were obtained at the (U)B3LYP/Def2TZVP/PCM(dichloromethane)//(U)B3LYP/6-31G(d)+SDD(Fe)/PCM(dichloromethane) level. All energies are referred considering the lowest in energy reactant complex in the quartet electronic state $\mathbf{1^q}$ as zero.

C) Optimized geometries for the stationary points reported in B). Mulliken charges (*q*) and spin density (ρ) for the phenyl group (sum of all C and H atoms), C2 benzylic position, C1 and O, are reported for the optimized transition states and intermediates. Values are given in a.u.

D) Intrinsic Reaction Coordinate calculations (IRC) starting from $\mathbf{TS1^d}$ (conformer 1 and conformer 2) followed by an optimization starting from the last structure of the IRC calculation, at the (U)B3LYP/6-31G(d)+SDD(Fe)/PCM(dichloromethane) level. The structure of the last point of the IRC and the optimization are provided as insets.

Distances, angles, and energy values are given in Angstrom (Å), degrees (º), and kcal·mol^{-1}, respectively.

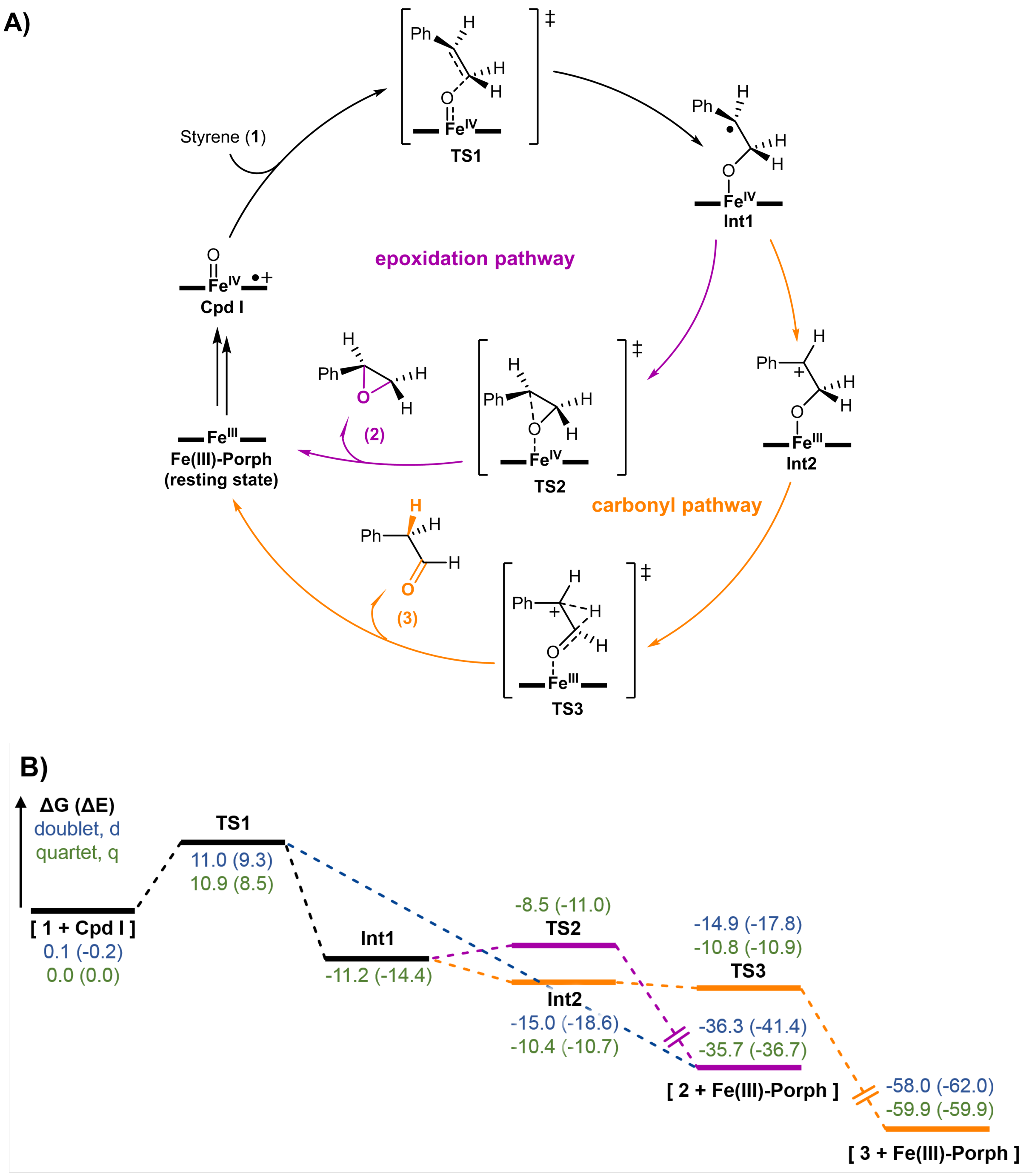

A)
Styrene (1)
TS1
Int1
Cpd I
epoxidation pathway
Fe(III)-Porph
(resting state)
(2)
TS2
Int2
carbonyl pathway
(3)
TS3
B)
ΔG (ΔE)
doublet, d
quartet, q
[1 + Cpd I]
0.1 (-0.2)
0.0 (0.0)
TS1
11.0 (9.3)
10.9 (8.5)
Int1
-11.2 (-14.4)
-8.5 (-11.0)
TS2
Int2
-15.0 (-18.6)
-10.4 (-10.7)
-14.9 (-17.8)
-10.8 (-10.9)
TS3
-36.3 (-41.4)
-35.7 (-36.7)
[2 + Fe(III)-Porph]
-58.0 (-62.0)
-59.9 (-59.9)
[3 + Fe(III)-Porph]

C)

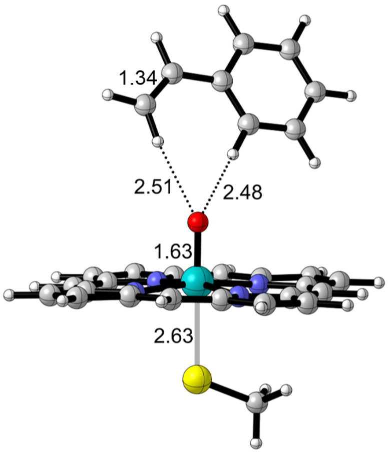

[1 + Cpd I]^{d}
(reactant complex)
ΔG= 0.1 (ΔE= -0.2)

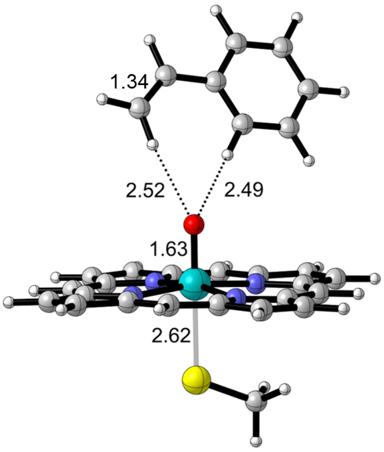

[1 + Cpd I]^{q}
(reactant complex)
ΔG= 0.0 (ΔE= 0.0)

q(Ph) = 0.11
q(C2) = -0.12
q(C1) = -0.16
q(O) = -0.47

ρ(Ph) = -0.10
ρ(C2) = -0.21
ρ(C1) = -0.02
ρ(O) = 0.30

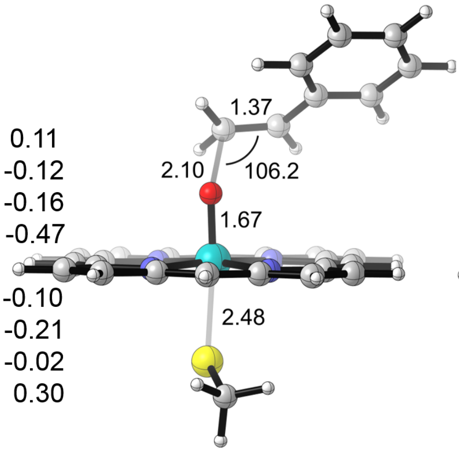

TS1^{d}
(conformer 1)
ΔG‡= 10.9 (ΔE‡= 9.5)
|∠Fe-O-C1-C2| = 77.0°

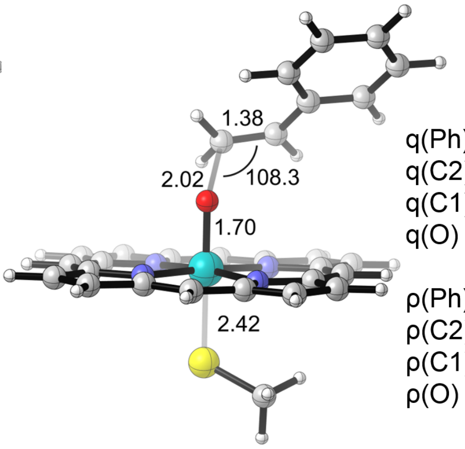

q(Ph) = 0.07
q(C2) = -0.14
q(C1) = -0.16
q(O) = -0.44

ρ(Ph) = 0.13
ρ(C2) = 0.33
ρ(C1) = -0.06
ρ(O) = 0.75

TS1^{q}
(conformer 1)
ΔG‡= 10.9 (ΔE‡= 8.5)
|∠Fe-O-C1-C2| = 85.0°

q(Ph) = 0.14
q(C2) = -0.11
q(C1) = -0.20
q(O) = -0.47

ρ(Ph) = -0.10
ρ(C2) = -0.22
ρ(C1) = -0.00
ρ(O) = 0.30

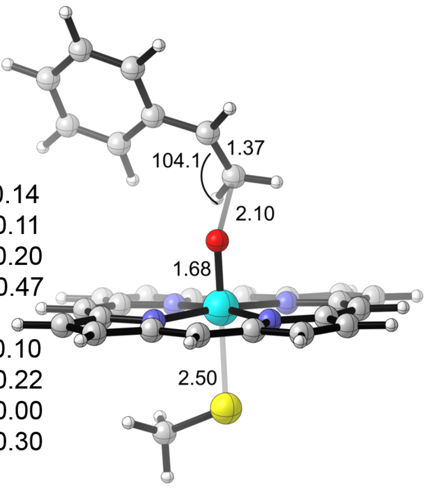

TS1^{d}
(conformer 2)
ΔG‡ = 11.2 (ΔE‡= 10.4)
|∠Fe-O-C1-C2| = 160.0°

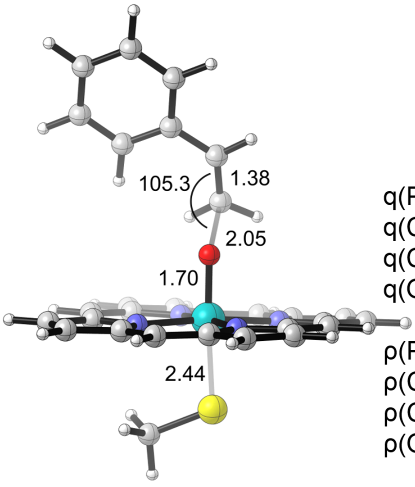

q(Ph) = 0.11
q(C2) = -0.12
q(C1) = -0.19
q(O) = -0.44

ρ(Ph) = 0.13
ρ(C2) = 0.33
ρ(C1) = -0.06
ρ(O) = 0.73

TS1^{q}
(conformer 2)
ΔG‡ = 12.2 (ΔE‡= 10.3)
|∠Fe-O-C1-C2| = 178.8°

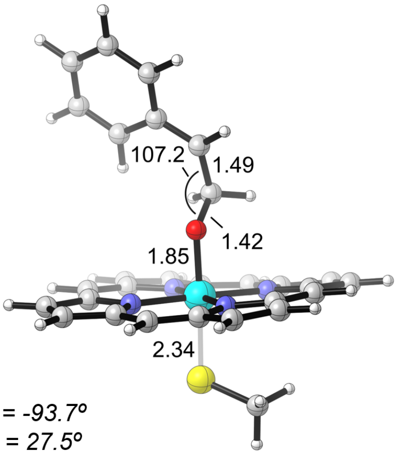

q(Ph) = 0.05
q(C2) = -0.17
q(C1) = 0.01
q(O) = -0.42

ρ(Ph) = 0.26
ρ(C2) = 0.67
ρ(C1) = 0.00
ρ(O) = 0.19

∠O-C1-C2-C(Ph) = -93.7º
∠H-C1-C2-C(Ph) = 27.5º

Int1^{q}
$\Delta G_r(\Delta E_r)$= -11.2 (-14.4)
ΔΔG= 0.0 (ΔΔE= 0.0)

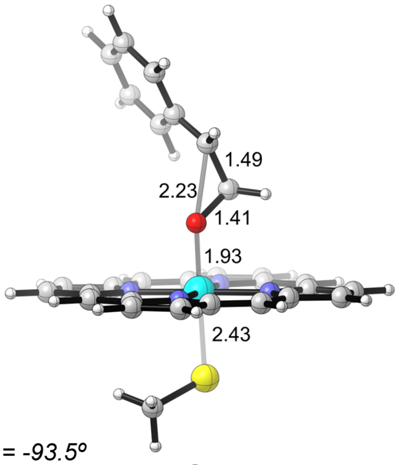

q(Ph) = 0.10
q(C2) = -0.12
q(C1) = 0.01
q(O) = -0.44

ρ(Ph) = 0.22
ρ(C2) = 0.56
ρ(C1) = 0.00
ρ(O) = 0.02

∠O-C1-C2-C(Ph) = -93.5º

TS2^{q}
$\Delta G^{‡}$= 2.7 ($\Delta E^{‡}$= 3.4)

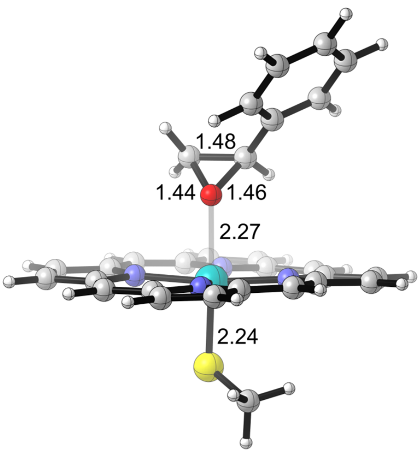

[2 + Fe(III)-Porph]^{d}
(product complex)
ΔG_r= -25.1 (ΔE_r= -27.0)

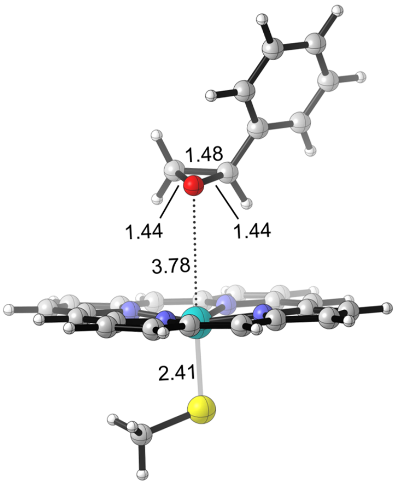

[2 + Fe(III)-Porph]^{q}
(product complex)
ΔG_r= -24.5 (ΔE_r= -22.3)

q(Ph) = 0.39
q(C2) = -0.05
q(C1) = 0.06
q(O) = -0.54

ρ(Ph) = 0.00
ρ(C2) = 0.00
ρ(C1) = 0.00
ρ(O) = 0.01

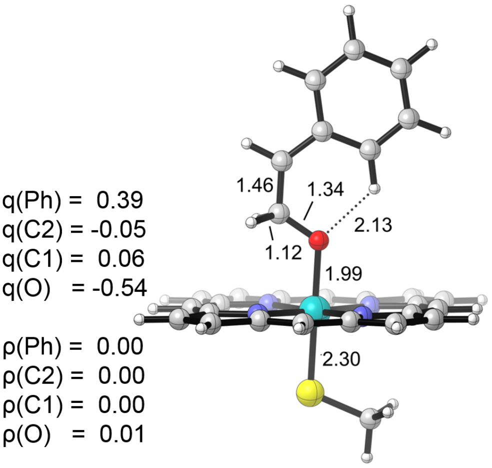

Int2^{d}
ΔΔG= -3.8 (ΔΔE= -4.2)
∠O-C1-C2-C(Ph) = 0.8º
∠H-C1-C2-C(Ph) = 130.0º

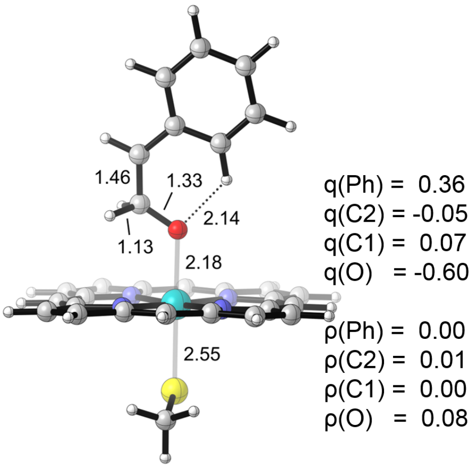

q(Ph) = 0.36
q(C2) = -0.05
q(C1) = 0.07
q(O) = -0.60

ρ(Ph) = 0.00
ρ(C2) = 0.01
ρ(C1) = 0.00
ρ(O) = 0.08

Int2^{q}
ΔΔG= 0.8 (ΔΔE= 3.7)
∠O-C1-C2-C(Ph) = -2.9º
∠H-C1-C2-C(Ph) = 124.1º

q(Ph) = 0.31
q(C2) = -0.08
q(C1) = 0.07
q(O) = -0.52

ρ(Ph) = 0.00
ρ(C2) = 0.01
ρ(C1) = 0.00
ρ(O) = 0.00

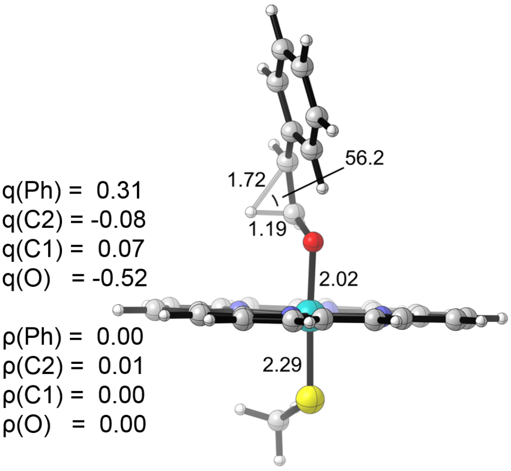

TS3^{d}
ΔG‡= 0.0 (ΔE‡= 0.8)
∠H-C1-C2-C(Ph) = 101.2º

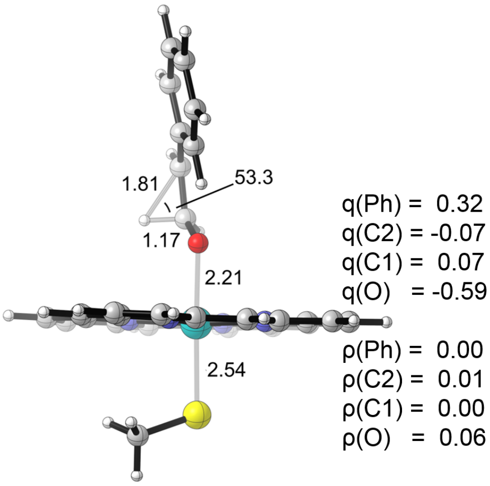

q(Ph) = 0.32
q(C2) = -0.07
q(C1) = 0.07
q(O) = -0.59

ρ(Ph) = 0.00
ρ(C2) = 0.01
ρ(C1) = 0.00
ρ(O) = 0.06

TS3^{q}
ΔG‡= -0.4 (ΔE‡= -0.1)
∠H-C1-C2-C(Ph) = 106.7º

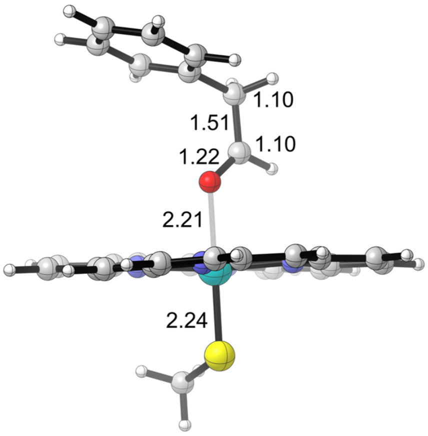

[3 + Fe(III)-Porph]^{d}
(product complex)
ΔG_r= -43.1 (ΔE_r= -43.5)

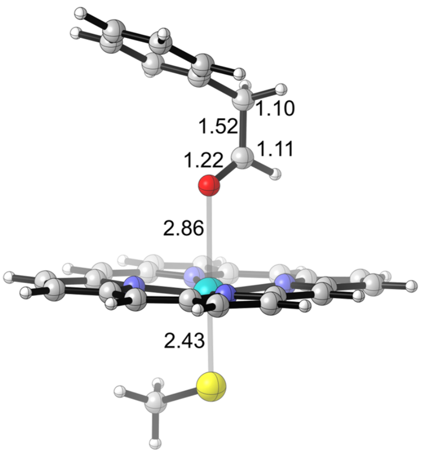

[3 + Fe(III)-Porph]^{q}
(product complex)
ΔG_r= -49.5 (ΔE_r= -49.2)

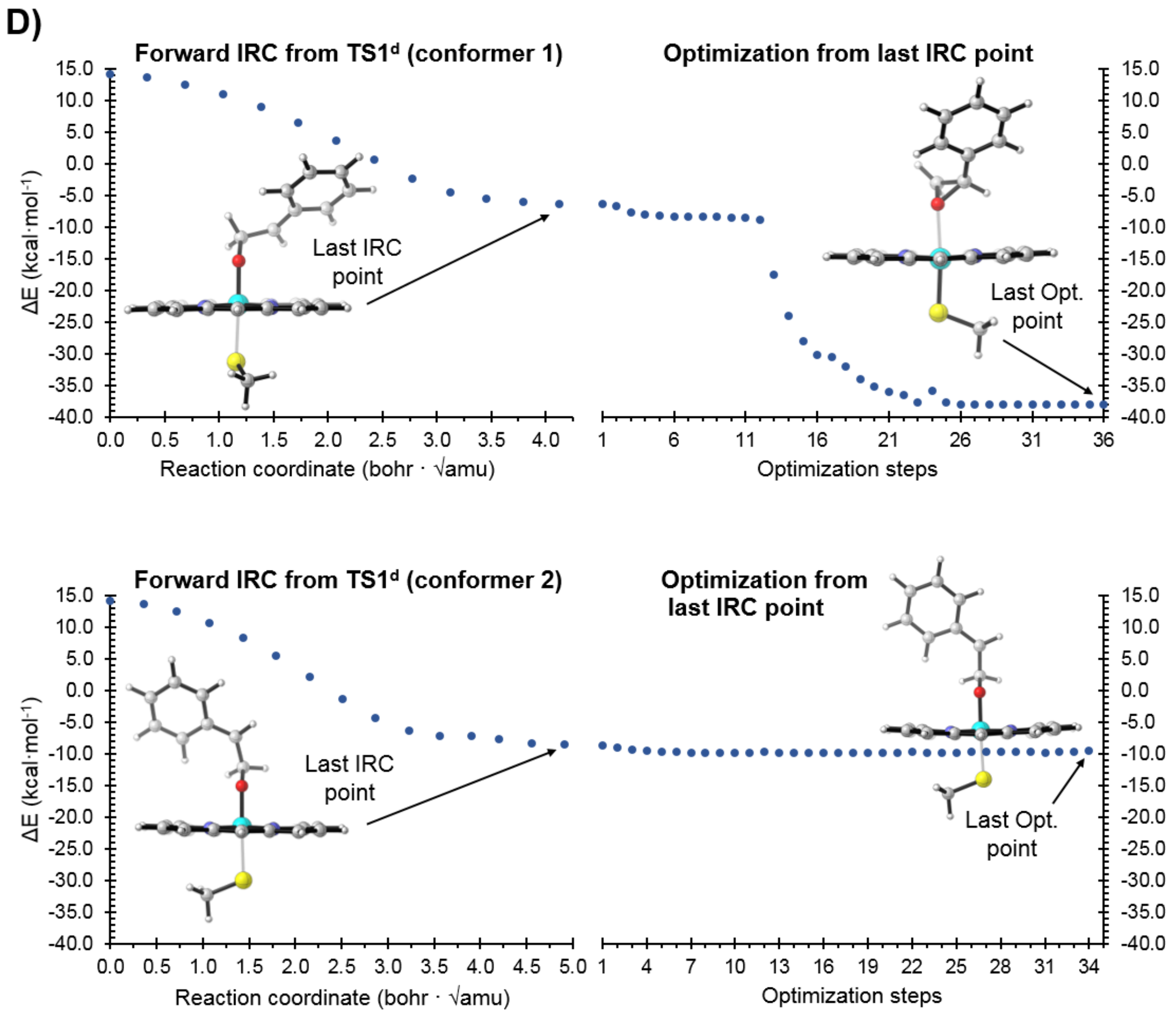

The two lowest in energy conformers optimized for **TS1** are found with a low energy difference (ca. 0.4 kcal·mol^{-1}) between them. The major geometric difference between these two lowest in energy conformers is the relative orientation of the styrene with respect to the iron-oxo species, defined by the ∠(Fe-O-C1-C2) dihedral angle. This geometric parameter describes the major remarkable structural difference between the two conformers, being ∠(Fe-O-C1-C2) = 85.0º for conformer 1 and ∠(Fe-O-C1-C2) = 160.0º for conformer 2.

IRC calculations carried out for **TS1** in the doublet electronic state, reported in **Figure S1-D** describe the potential formation of a radical intermediates. Subsequent full optimizations starting form the last IRC converged structure lead to the direct formation of styrene oxide product (**2**), from **TS1** - conformer 1, or do not reach convergency after 35 cycles of optimization, **TS1** - conformer 2. Consequently, a radical intermediate in the doublet state could not be optimized as a stationary point and minimum on the potential energy surface (PES). However, these IRC calculations are strongly suggesting that this corresponds to a flat region on the doublet PES, and that small perturbations (interactions with surrounding active site residues, for instance) would stabilize the formation of a radical intermediate in the doublet electronic state (as discovered after in our modelling of the enzymatic reaction).

Table S1: DFT computed relative energies for all the stationary points described in **Figure S1** in doublet (d) and quartet (q) electronic states in terms of electronic energy (ΔE), enthalpy (ΔH), and quasi-harmonic corrected Gibbs energy (ΔG). Energy values were obtained at the (U)B3LYP/Def2TZVP/PCM(dichloromethane)//(U)B3LYP/6-31G(d)+SDD(Fe)/PCM(dichloromethane) level. Energies are referred considering the lowest in energy quartet **1**^{q} (reactant complex) in the quartet electronic state as zero. All energies are given in kcal·mol^{-1}.

Structure	Electronic State	ΔE	ΔH	ΔG
**[1 + Cpd I]**	doublet (d)	-0.2	-0.2	0.1
(reactant complex)	quartet (q)	0.0	0.0	0.0
**TS1**	doublet (d)	9.3	8.2	11.0
(conformer 1)	quartet (q)	8.5	7.5	10.9
**TS1**	doublet (d)	10.1	9.0	11.3
(conformer 2)	quartet (q)	10.3	9.3	12.2
**Int1**	quartet (q)	-14.4	-14.2	-11.2
**TS2**	quartet (q)	-11.0	-11.5	-8.5
**[2 + Fe(III)-Porph]**	doublet (d)	-41.4	-39.9	-36.3
(product complex)	quartet (q)	-36.7	-35.4	-35.7
**Int2**	doublet (d)	-18.6	-18.9	-15.0
	quartet (q)	-10.7	-11.6	-10.4
**TS3**	doublet (d)	-17.8	-19.2	-14.9
	quartet (q)	-10.9	-12.7	-10.8
**[3 + Fe(III)-Porph]**	doublet (d)	-62.0	-60.9	-58.0
(product complex)	quartet (q)	-59.9	-59.1	-59.9

Figure S2: DFT calculations on the truncated model were carried out to study the rotation of the C1–C2 bond in the covalent radical intermediate in the quartet electronic state (**Int1^q**, **Figure S1**). which enables the formation of the covalent carbocation intermediate (**Int2^q**, **Figure S1**).

A) Two independent relaxed scan calculations were carried out along the rotation of the ∠(O-C1-C2-C(Ph)) dihedral angle starting from each optimized intermediate species (**Int1^q** and **Int2^q**). Results for the radical intermediate (**Int1^q**, structure on the right) and the carbocation intermediate (**Int2^q**, structure on the left) are shown with black markers and blue markers, respectively. Results for the calculations starting from the carbocation intermediate are also highlighted in an inset graph. Electronic energies were obtained at the (U)B3LYP/6-31G(d)+SDD(Fe)/PCM (dichloro-methane) level.

B) DFT optimized rotation transition state (**TS-rotation^q**). It was optimized starting from the highest in energy point on the relaxed scan coordinate. Mulliken charges (*q*) and spin densities (ρ) for the phenyl group (sum of all C and H atoms), C2 benzylic position, C1 and O are given in a.u. This transition state resembles the geometry of **Int2^q**, but possessing a radical character at the benzylic position. The **TS-rotation^q** connects two equivalent enantiomeric **Int1^q** structures.

C) DFT computed relative energies for all relevant stationary points in doublet (d) and quartet (q) electronic states in terms of electronic energy (ΔE), enthalpy (ΔH), and quasi-harmonic corrected Gibbs energy (ΔG). Energy values were obtained at the (U)B3LYP/Def2TZVP/PCM(dichloro-methane)//(U)B3LYP/6-31G(d)+SDD(Fe)/PCM (dichloromethane) level.

Energies are referred considering the optimized **Int1^q** as zero. Energies and dihedrals are given in kcal·mol^{-1} and degrees (º), respectively.

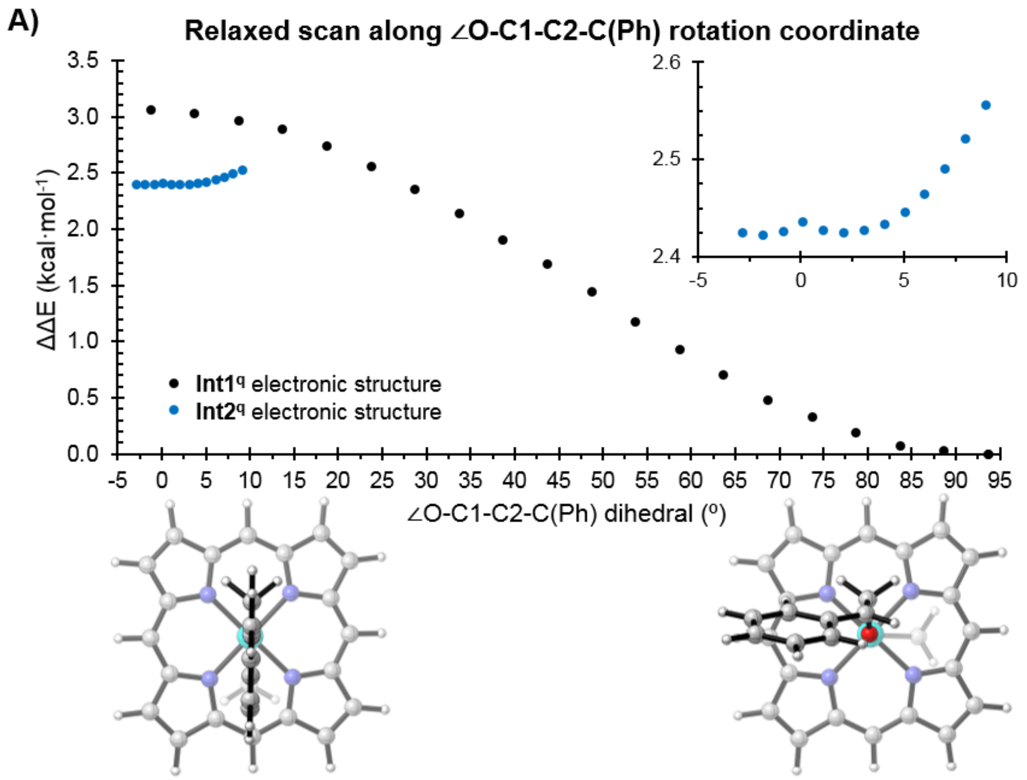

B)

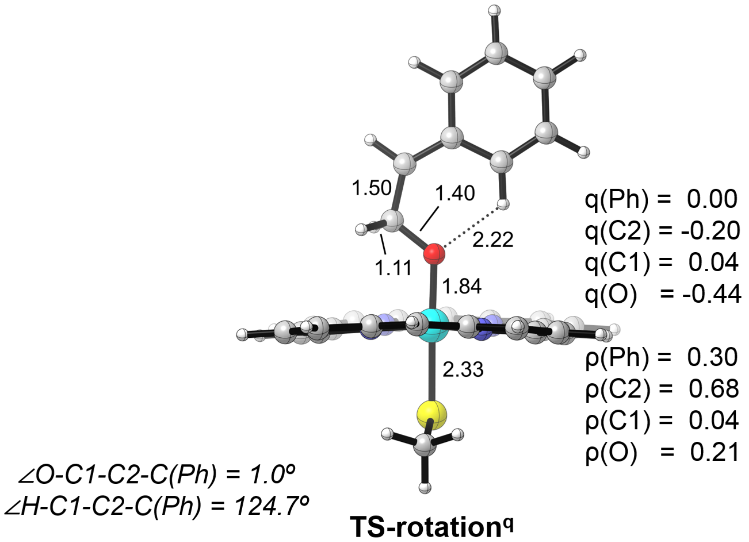

TS-rotation[q]
$\Delta G^{\ddagger}$= 2.0 ($\Delta E^{\ddagger}$= 1.9)

C)

Structure	Electronic State	ΔE	ΔH	ΔG
**Int1** [a]	quartet (q)	0.0	0.0	0.0
**TS2** [a]	quartet (q)	3.4	2.7	2.7
**TS-rotation**	quartet (q)	1.9	0.9	2.0
**Int2** [a]	doublet (d)	-4.2	-4.7	-3.8
	quartet (q)	3.7	2.6	0.8

[a] From **Figure S1**

Relaxed scan calculation starting from the optimized radical **Int1**[q] geometry (black dots) showed that rotation along C1-C2 bond is energetically feasible and that it can bend to reach carbocation-like geometries (with ∠(O-C1-C2-C(Ph) ca. 0º) with low energy requirements (3.1 kcal·mol^{-1}). This conformation corresponds to a transition state (**TS-rotation**[q]) for the phenyl rotation in the radical electronic structure, with $\Delta G^{\ddagger}$ = 2.0 kcal·mol^{-1}. This optimized **TS-rotation**[q] is ca. 0.7 kcal·mol^{-1} lower in energy than the optimized **TS2**[q], and ca. 1.2 kcal·mol^{-1} higher in energy than the carbocation intermediate in the quartet electronic state (**Int2**[q]). Geometrically, both **TS-rotation** and **Int2**[q] exhibit a similar orientation of the aromatic ring.

The relaxed scan calculations starting from the carbocation intermediate **Int2**[q] showed that distortions of the dihedral angle larger than 10º induce the spontaneous 1,2-hydride migration. This is in line with the high reactivity of **Int2**, and its stabilization due to stereoelectronic effects occurring in the optimal geometry with ∠(O-C1-C2-C(Ph) angle near to zero. Additionally, the minimum energy crossing point (MECP) between doublet (d) and quartet (q) carbocation intermediates **Int2** was also studied (see **Figure S4**).

Figure S3: **A-C)** Frontier Molecular Orbitals (FMO) and **D)** spin density (ρ) distribution corresponding to the key intermediates **Int1**$^{\mathbf{q}}$ (quartet), **Int2**$^{\mathbf{d}}$ (doublet), and **Int2**$^{\mathbf{q}}$ (quartet) described in **Figure S1**. Molecular orbitals (isovalue = +0.02 and -0.02) and spin density (isovalue = 0.008) were obtained at the (U)B3LYP/Def2TZVP/PCM(dichloromethane)// (U)B3LYP/6-31G(d)+SDD(Fe)/PCM(dichloromethane) level. Molecular Orbital energies are given in eV. Mulliken spin densities (ρ) of key atoms and groups (Ph = sum of all atoms on the aromatic ring) are reported in a.u.

A)

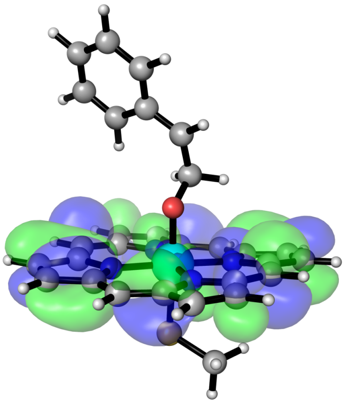

Int1$^{\mathbf{q}}$
α-LUMO+1 (-2.7 eV)

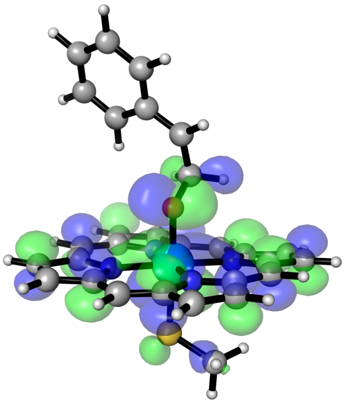

Int1$^{\mathbf{q}}$
β-LUMO+1 (-3.3 eV)

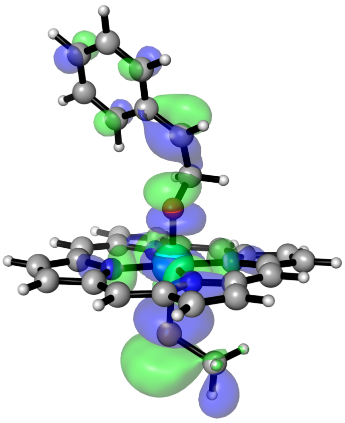

Int1$^{\mathbf{q}}$
α-LUMO (-3.0 eV)

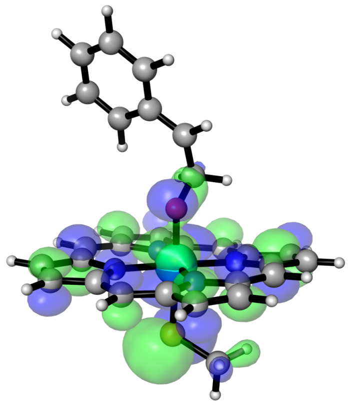

Int1$^{\mathbf{q}}$
β-LUMO (-3.4 eV)

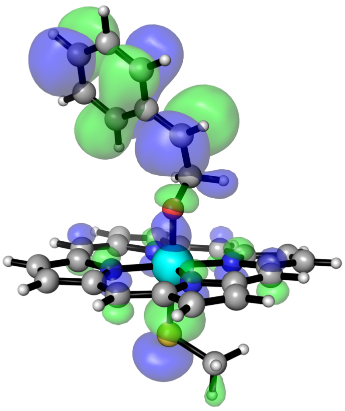

Int1$^{\mathbf{q}}$
α-HOMO (-5.1 eV)

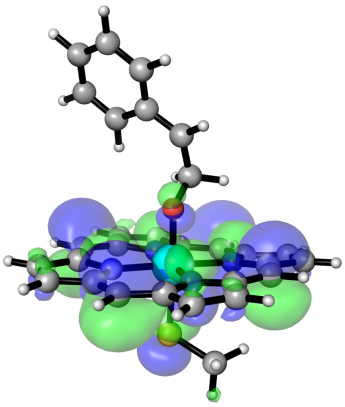

Int1$^{\mathbf{q}}$
β-HOMO (-5.6 eV)

B)

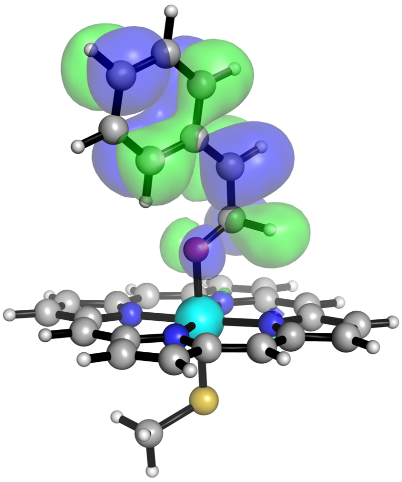

Int2^d
α-LUMO (-3.9 eV)

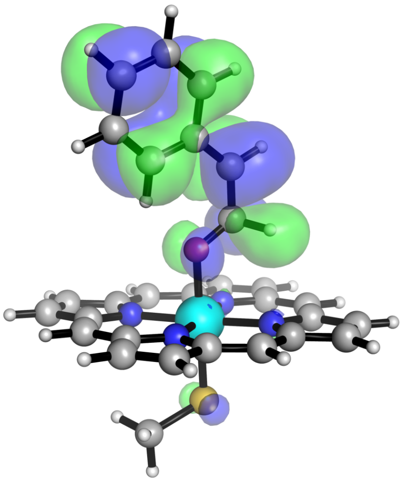

Int2^d
β-LUMO (-3.9 eV)

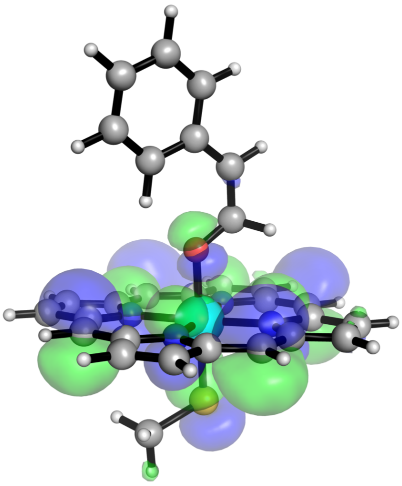

Int2^d
α-HOMO (-5.2 eV)

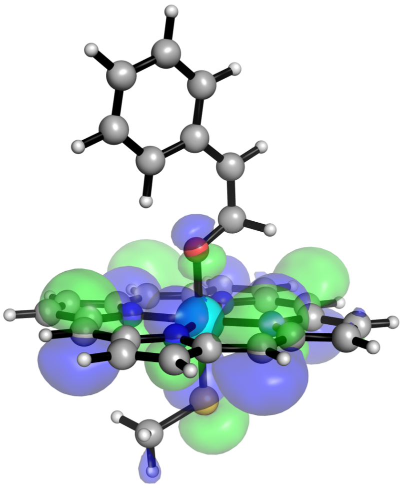

Int2^d
β-HOMO (-5.2 eV)

C)

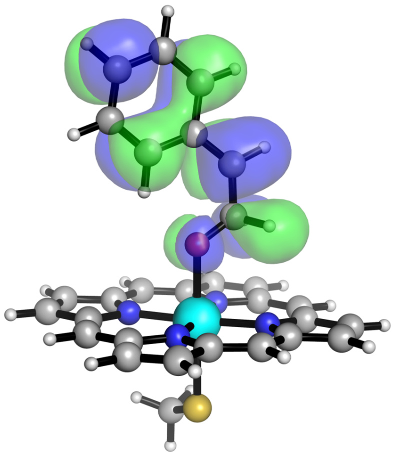

Int2^{q}
α-LUMO (-3.8 eV)

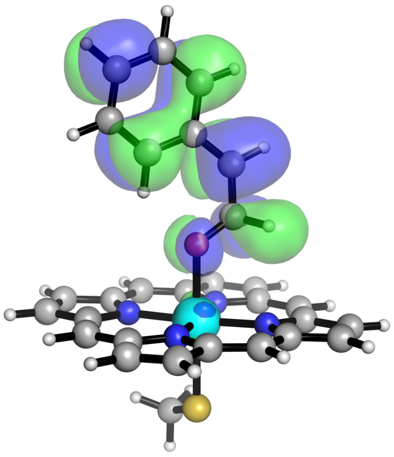

Int2^{q}
β-LUMO (-3.8 eV)

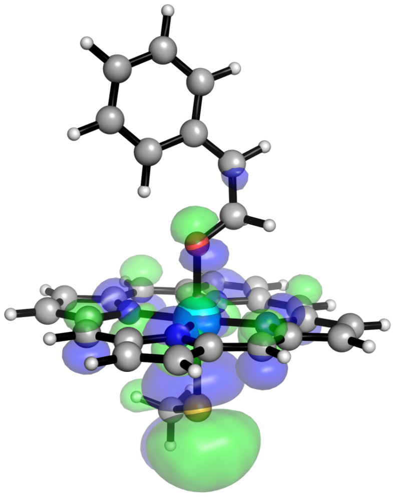

Int2^{q}
α-HOMO (-4.8 eV)

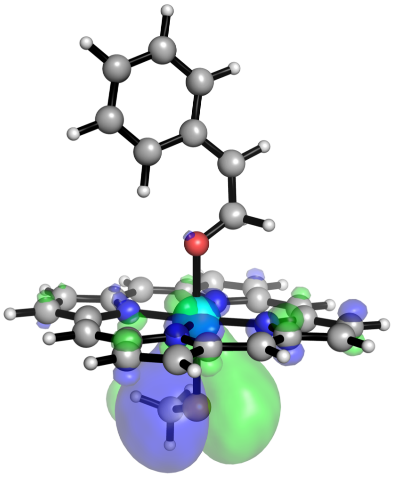

Int2^{q}
β-HOMO (-4.9 eV)

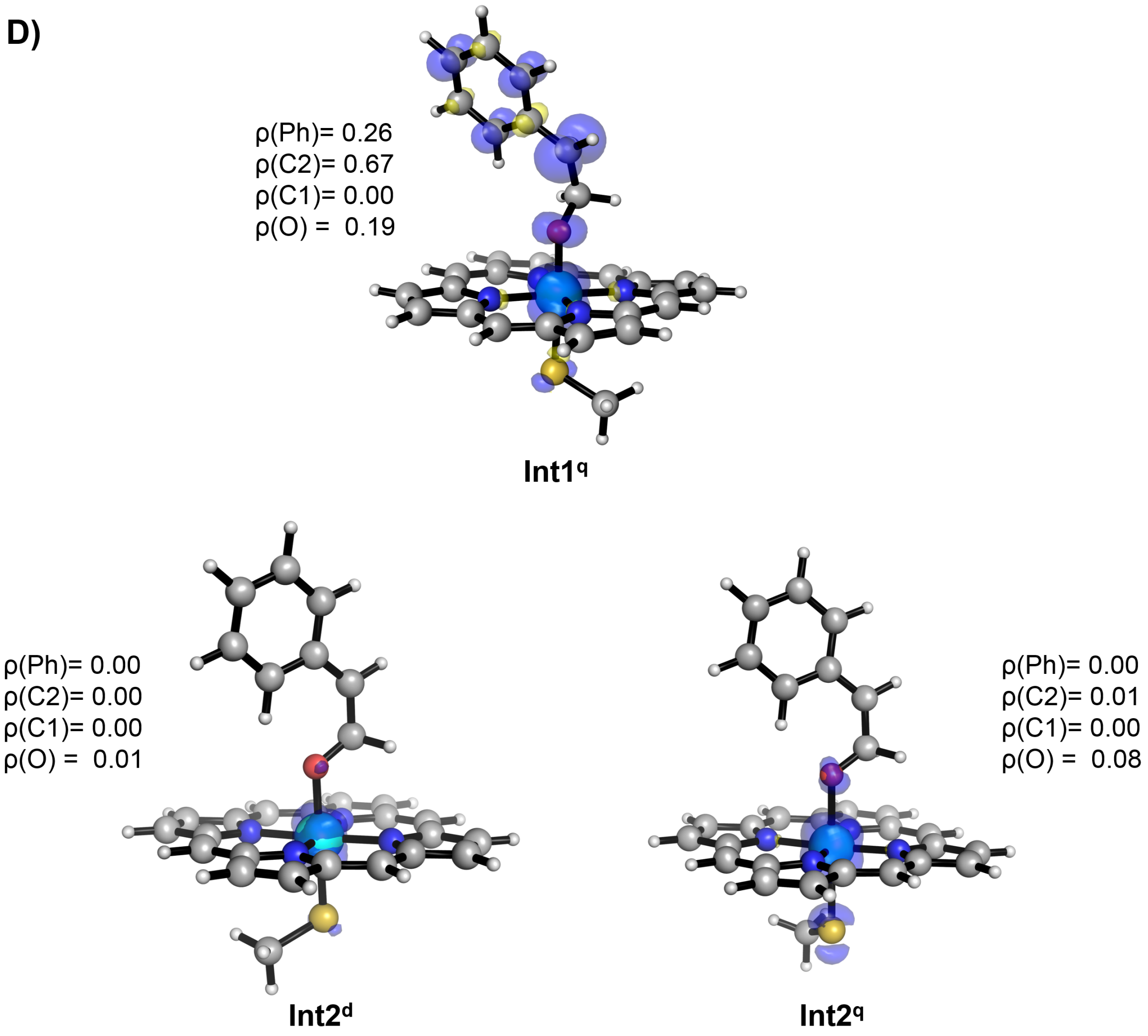

Analysis of the Frontier Molecular Orbitals of optimized radical intermediate **Int1^{q}** (**A**) describe that the highest in energy occupied orbital, α-HOMO, is mainly localized on the former substrate moiety, while the α-LUMO and α-LUMO+1, and β-LUMO and β-LUMO+1, are mainly localized on the iron-oxygen, porphyrin and axial methanethiolate ligands.

On the other hand, covalent carbocation intermediate (**Int2**) in doublet (d) and quartet (q) electronic states (**B** and **C**, respectively) have both the α- and β-HOMO orbitals mainly localized on the iron-oxygen, porphyrin and axial methanethiolate ligands. In contrast, both the α and β-LUMO orbitals localized on the former substrate moiety.

The inverted occupancy of the FMOs in the radical and carbocation intermediates is describing an intramolecular electronic rearrangement that involves an electron transfer from the substrate moiety to the iron-heme. This is also reflected by the changes observed on the spin density distribution (**D**).

Figure S4: Minimum energy crossing point (MECP) between doublet and quartet potential energy surfaces (PES) for carbocation intermediate (**Int2**). **A)** Relative energies (E, electronic energy; H enthalpy; G, Gibbs free energy) for the carbocation intermediate (**Int2**) in the doublet and quartet electronic states, and the optimized MECP at the (U)B3LYP/6-31G(d)+SDD(Fe)/PCM(dichloromethane) level of theory. Single point (SP) energies at the (U)B3LYP/Def2TZVP/PCM(dichloromethane) level are also reported. **B)** Optimized structures for the studied species. Relative energies are referred considering the lowest in energy **Int2^d^** as zero. Energies and distances are given in kcal·mol^{-1} and Angstroms (Å), respectively.

A)

Structure	Electronic State	ΔΔE	ΔΔH	ΔΔG
**Int2** (optimization)	doublet (d)	0.0	0.0	0.0
	MECP	8.1	5.5	4.9
	quartet (q)	8.1	7.5	4.8
**Int2** (SP)	doublet (d) [a]	0.0	0.0	0.0
	MECP [b]	6.8	4.6	5.6
	quartet (q) [a]	7.8	7.3	4.6

[a] From **Figure S1**.

[b] Energy values obtained considering the quartet electronic state of the optimized MECP geometry.

B)

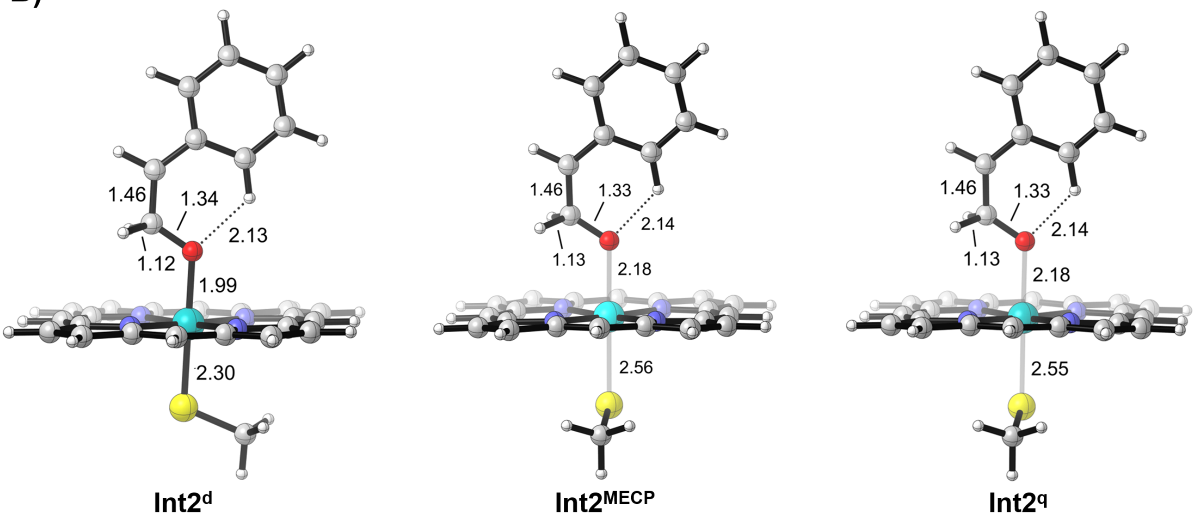

MECP between doublet and quartet potential energy surfaces (PES) for carbocation intermediate (**Int2**) is very close in energy to the quartet **Int2^q**. This is indicating that the energetic cost of crossing from the higher in energy quartet state **Int2^q** to the most stable doublet state **Int2^d** can compete with the 1,2-hydride migration transition state (**TS3^q**, see **Figure S1**).

III. Quasiclassical direct dynamics trajectory simulations

Figure S5: Displacement vectors (represented as yellow arrows) associated to the imaginary frequency of optimized **TS1**. The two relevant optimized conformers for **TS1** in the doublet (d) and quartet (q) electronic states are shown (see **Figure S1**). Energy values were obtained at the (U)B3LYP/Def2TZVP/PCM(dichloromethane)//(U)B3LYP/6-31G(d)+SDD(Fe)/PCM(dichloromethane) level. All energies are referred considering the reactant complex (**1**^{q}) as zero (see **Table S1**). Energy values are given in kcal·mol^{-1}.

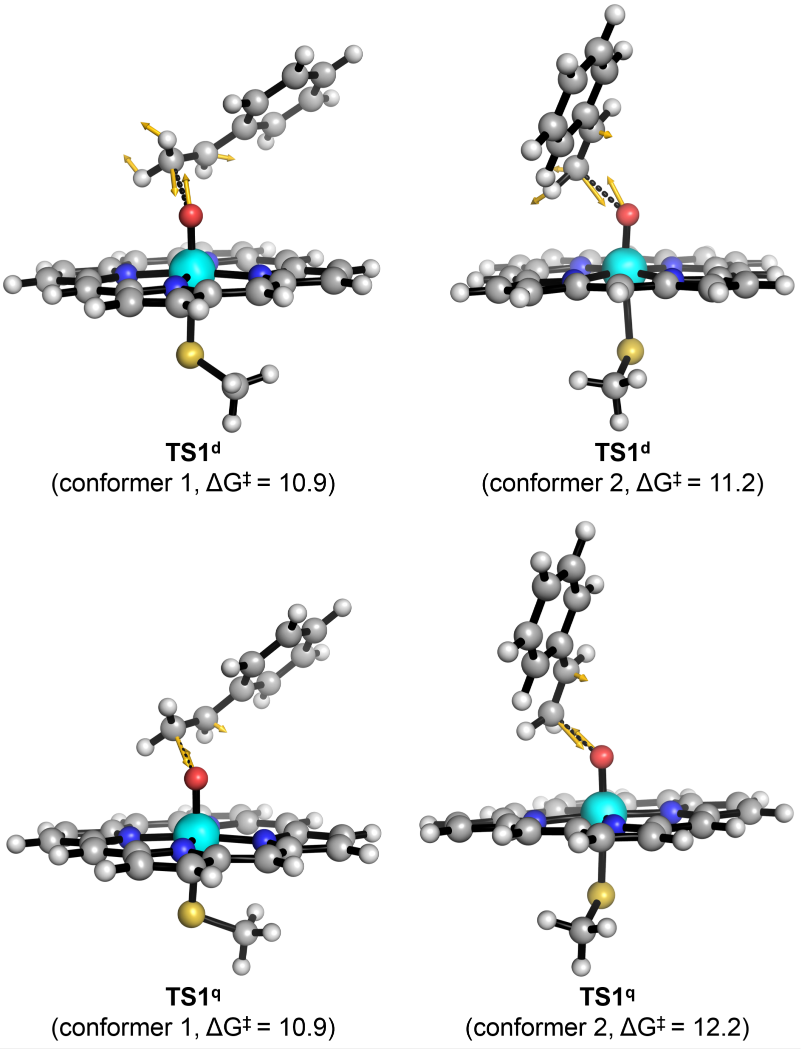

The displacement vectors associated to the imaginary frequencies of optimized **TS1** are very similar for both spin sates (doublet and quartet) and relevant conformers. In all cases, the imaginary frequency involves also the motion of C2.

Figure S6: Direct quasiclassical dynamics trajectories (QCT) were carried out at the (U)B3LYP/6-31G(d)+ SDD(Fe) level. **A)** Summary of the trajectories carried out at the doublet (total of 20 trajectories) and quartet (total of 20 trajectories) electronic states trajectories that lead to epoxide formation, aldehyde formation, recrossing or radical intermediate that remained unreacted after reaching the trajectory time limit of 600 fs. Average time gap between C1–O and C2–O bond formation for epoxidations observed from direct QCT are reported. Distance vs time analysis for representative trajectories are reported for each electronic state: **B**) doublet, and **C**) quartet.

A)

**Electronic State**	**Observed event**	**Number of trajectories**
doublet (d)	Epoxide formation	15 *(average time gap: 92 fs)*
	Aldehyde formation	0
	Recrossing	5
	Unreacted radical intermediate	0
quartet (q)	Epoxide formation	11 *(average timegap: 265 fs)*
	Aldehyde formation	0
	Recrossing	2
	Unreacted radical intermediate	7

B)

Epoxide formation (d):

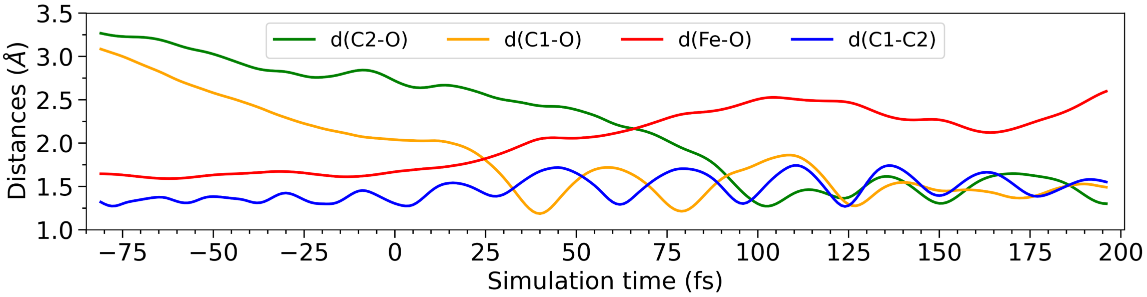

Recrossing (d):

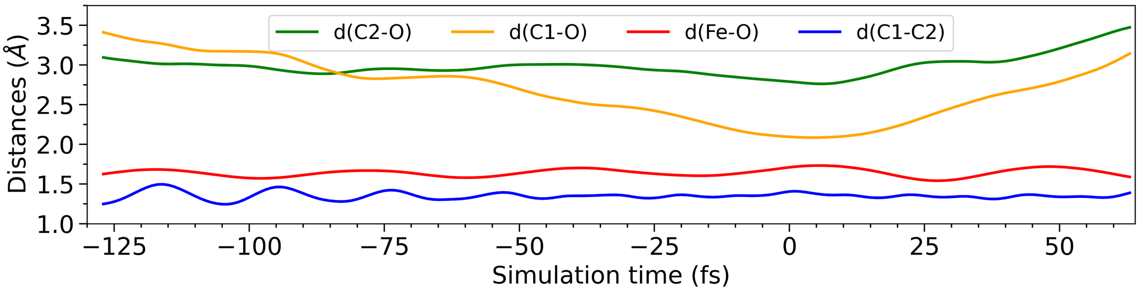

C)

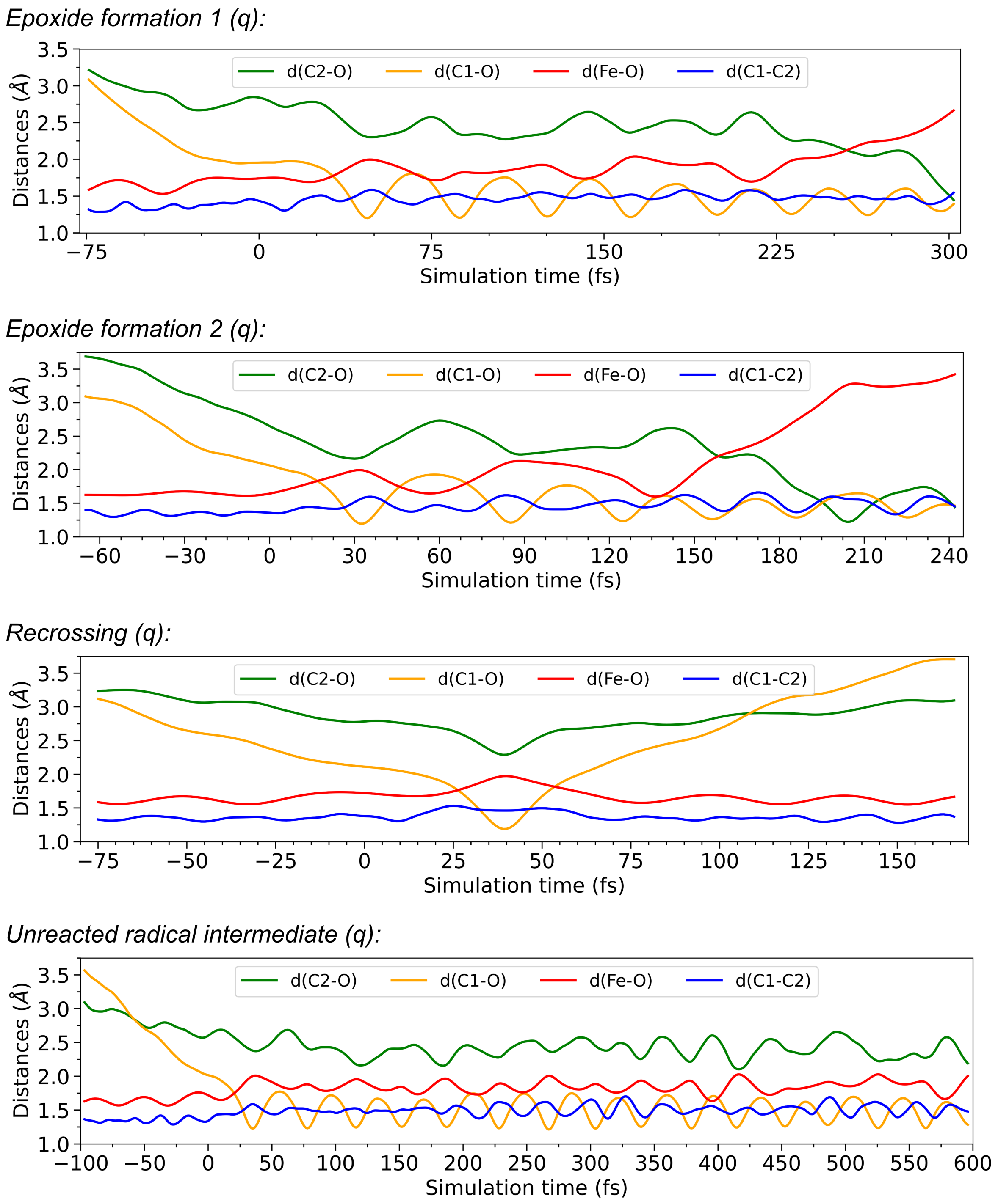

None of the 40 trajectories lead to the direct formation of the aldehyde product. Instead, 65% of these trajectories (15 doublet + 11 quartet) directly generate styrene oxide (**2**) and the 17.5% (5 doublet + 2 quartet) exhibited non-reactive recrossing events. In the quartet electronic state 35% of the trajectories (7 of 20) formed the covalent radical intermediate that remained unreacted after reaching the trajectory time limit of 600 fs. This is not observed in the doublet electronic state.

For reactive trajectories leading to the epoxide product (styrene oxide), an average time gap between the formation of C1–O and C2–O is estimated to be ca. 265 fs for the quartet electronic state and ca. 92 fs for the doublet (see also representative distance vs. time plots in **B** and **C**).

From these time gap values, the epoxidation pathway can be classified as dynamically concerted in both electronic states according to Houk and co-workers time gap criterion (see discussion in the main text).

The lifetime estimated for the quartet radical intermediate from epoxide reactive direct QCT is significantly lower than the one predicted from transition state theory (see main text for discussion), demonstrating the existence of a strong "*dynamic match*" coupling between the coordinates for C1–O and C2–O bond formation. On the other hand, for the doublet electronic state, the existence of a time gap > 60 fs, indicates the potential formation of an entropic intermediate prior epoxide formation. The formation of such entropic intermediate suggests that proper stabilization via conformational restraints or non-covalent interactions in an enzyme active site, for example, could lead to the formation of a covalent intermediate as a minimum on the doublet potential energy surface. This is indeed in line with the QM/MM modelling carried out in this study (see results in the main text), which allow the access to the carbonyl pathway also from the doublet electronic state.

IV. Computational modelling of the enzyme-substrate bound complexes

Figure S7: Representative structures of the most populated conformational state characterized from MD simulations (5 replicas of 1,000 ns each, accumulation a total of 5,000 ns) for P450$_{LA1}$ WT (structure in gray) and the laboratory evolved aMOx variant (structure in blue) in their *holo* states. The most populated conformational states were characterized by clusterization of the accumulated simulation time, based on the protein backbone RMSD. **A)** P450$_{LA1}$ and **B)** aMOx representative structures. **C)** Active sites of P450$_{LA1}$ and aMOx. Yellow surfaces describe the accessible active site volume estimated from volume calculations using POVME3.0 software. Relevant active site residues are shown as sticks and mutated positions are highlighted in orange.

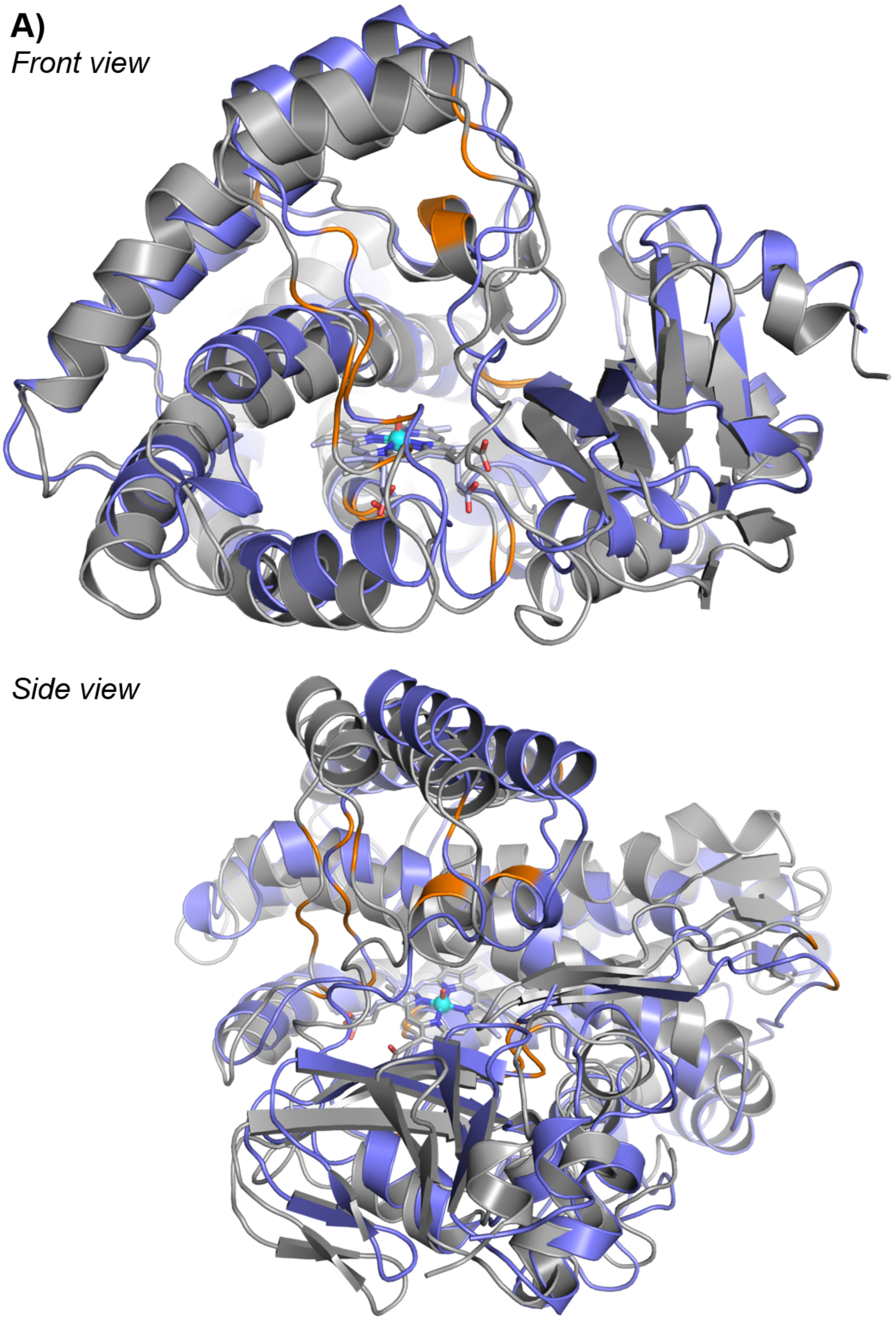

B)

P450$_{LA1}$

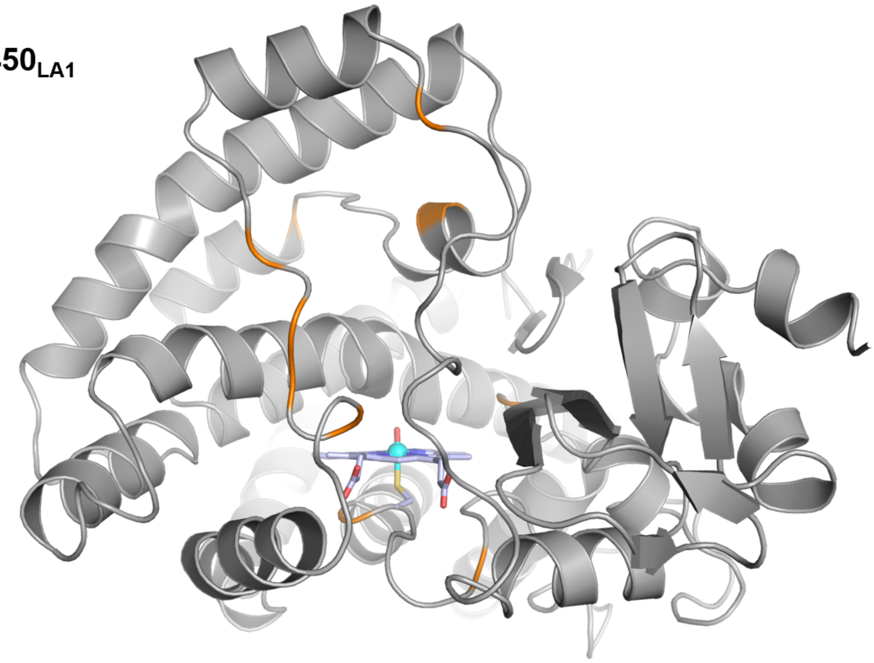

aMOx

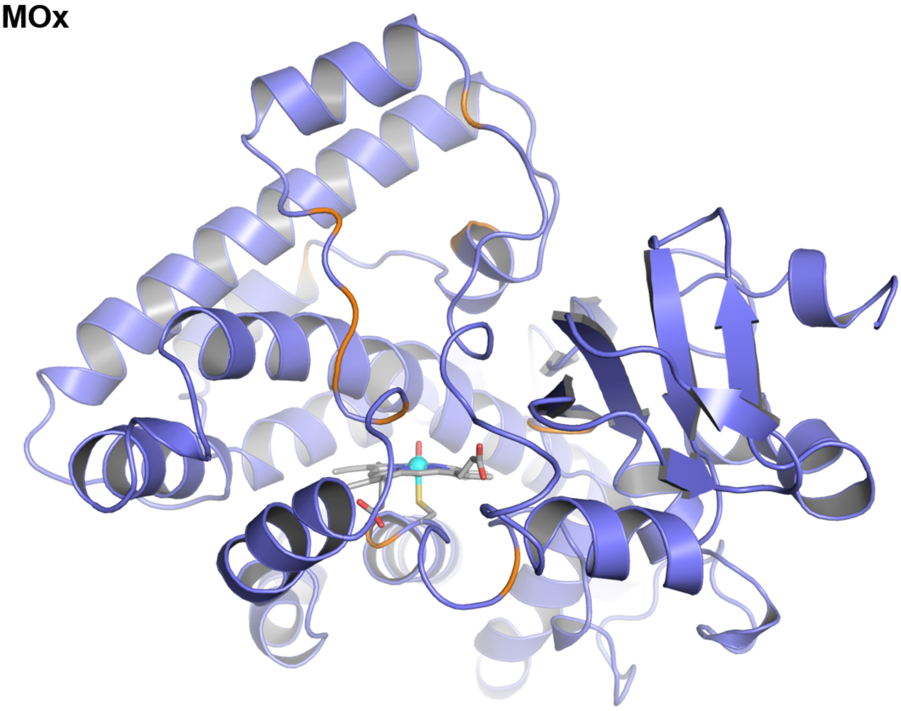

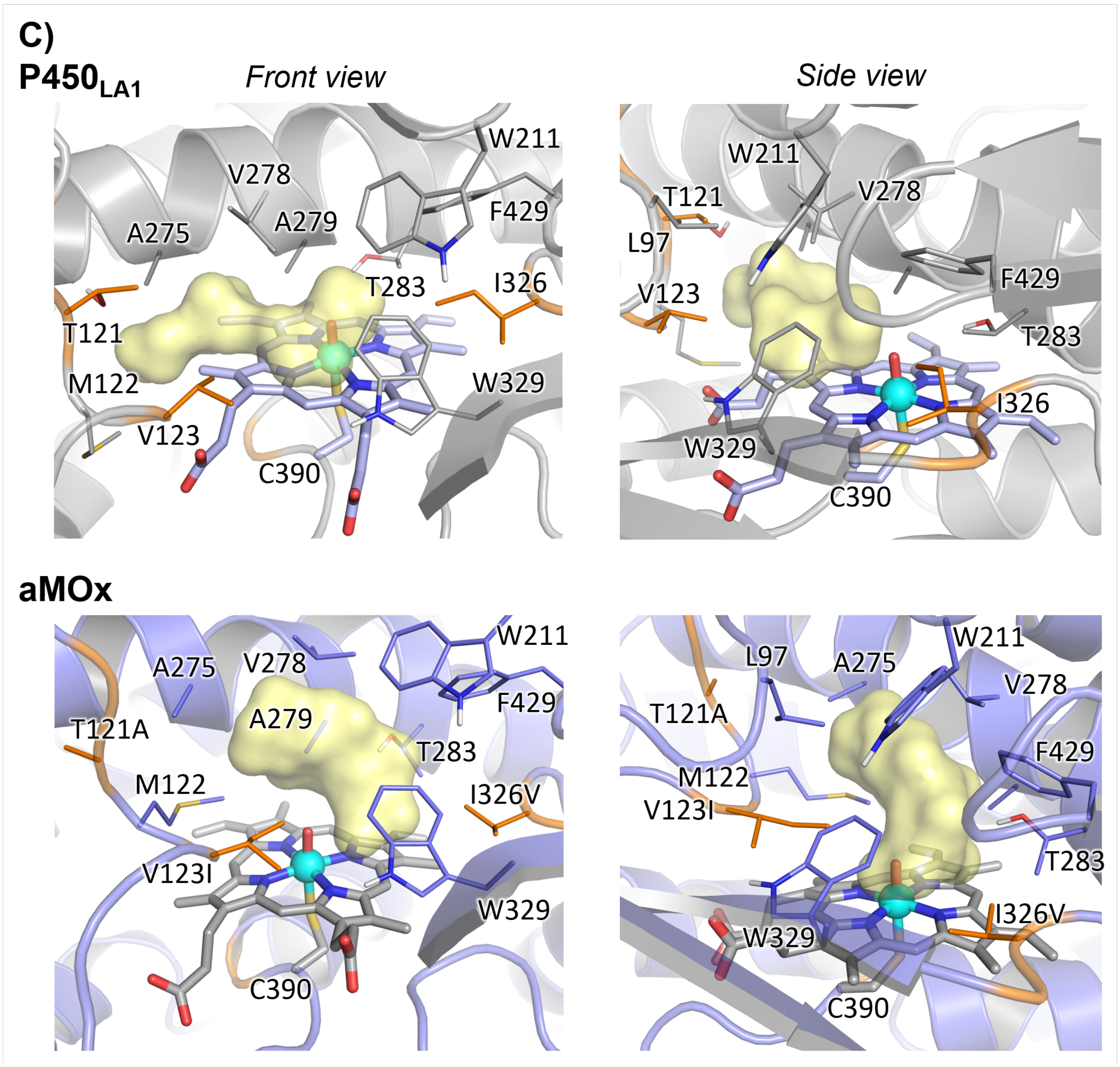

The active site reshaping observed in the laboratory evolved aMOx variant compared to the parent WT P450$_{LA1}$ is mainly caused by two major factors. First, I326V and V123I active site mutations directly modify the shape of the active site by displacing the accessible volume towards the right side (from the front view perspective). Second, the global impact of the mutations introduced by directed evolution at more distal positions from the active site (see **Figure 4A** in the main text), which modify the position of the two vertical loops (loops containing V123I and L97, respectively) in the substrate entrance channel. These conformational changes also reorient M122 sidechain inwards to the aMOx variant active site, contributing to the active site reshaping. The loop containing L97 is displaced closer to the heme cofactor as compared to the WT enzyme, making the accessible volume in aMOx narrower.

Figure S8: Analysis of the presence of ordered water molecules in P450$_{LA1}$ and aMOx active sites from styrene (**1**) bound restrained-MD simulations (5 replicas of 500 ns each, accumulating a total of 2,500 ns for each variant). Analysis were performed using *cpptraj* from Ambertools.

A) Schematic representation of the different interactions and water molecules analyzed: In blue, bridging water molecules between Thr283 and Fe-oxo (O atom); In green, O-acceptor water molecule interacting with the hydroxyl group of Thr283; In orange, H-donor water molecule hydrogen bonded with Thr283; In red, H-donor water molecule interacting with Fe-oxo (O atom); In black (but also including the previously analyzed water molecules), solvent molecules around 5.0 Å and 3.4 Å of styrene substrate.

B) Average statistics for the presence of each specific H-bond interaction as observed from MD simulations. Values bigger than 1.00 indicate that more than one water molecule can be simultaneously found performing that particular interaction.

A)

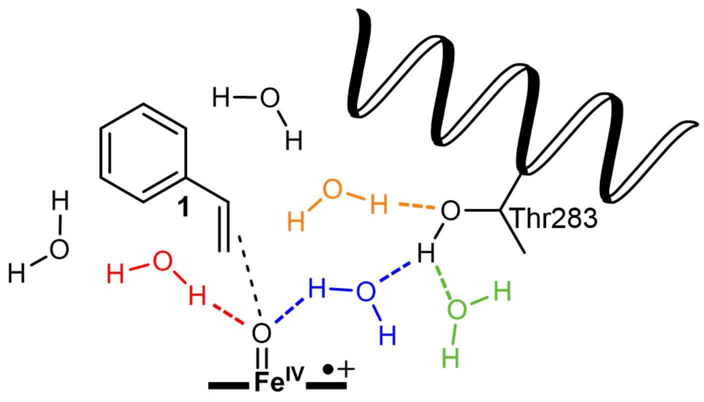

B)

Water analysis in MD simulations	LA1	aMOx
Bridging H_2O (Fe=O, HO-Thr283)	0.49	0.24
H_2O (HO-Thr283)	0.34	0.29
H_2O (OH-Thr283)	0.04	0.09
H_2O (Fe=O)	0.07	0.10
H_2O at 5.0 Å from styrene (**1**)	2.22	4.26
H_2O at 3.4 Å from styrene (**1**)	1.55	2.11

Simulations describe the significant presence of a bridging water molecule between the activated oxygen in Cpd I and the H-donor atom of Thr283 hydroxyl group. The potential impact of this bridging water molecule is evaluated in **Figure S9**.

Figure S9: DFT truncated model calculations to study the impact of a hydrogen bonding water molecule on the relative stability of the radical intermediate (**Int1**) and the carbocation intermediate (**Int2**). Doublet (d) and quartet (q) electronic states are considered. **A)** Relative electronic energy (ΔΔE), enthalpy (ΔΔH), and quasi-harmonic corrected Gibbs energy (ΔΔG) are given for each model system with (**Int1-wat** and **Int2-wat**) or without the water molecule (**Int1** and **Int2**). Energy values were obtained at the (U)B3LYP/Def2TZVP/PCM (dichloromethane)//(U)B3LYP/6-11G(d)+SDD(Fe)/PCM(dichloromethane) level. Energies are referred considering quartet radical intermediate (**Int1^q** or **Int1^q-wat**) as zero. **B)** Optimized structures for the intermediate models with a hydrogen bonded water molecule (**Int1-wat** and **Int2-wat**). Mulliken charges (q) and spin densities (ρ) of key atoms and groups are reported in a.u. Distances and energies are given in Angstroms (Å) and kcal·mol^{-1}, respectively.

A)

Structure	Electronic State	ΔΔE	ΔΔH	ΔΔG
**Int1** [a]	quartet (q)	0.0	0.0	0.0
**Int2** [a]	doublet (d)	-4.2	-4.7	-3.8
	quartet (q)	3.7	2.6	0.8
**Int1-wat**	quartet (q)	0.0	0.0	0.0
**Int2-wat**	doublet (d)	-4.5	-4.9	-3.3
	quartet (q)	-0.4	-1.2	-2.4

[a] From **Figure S1**.

B)

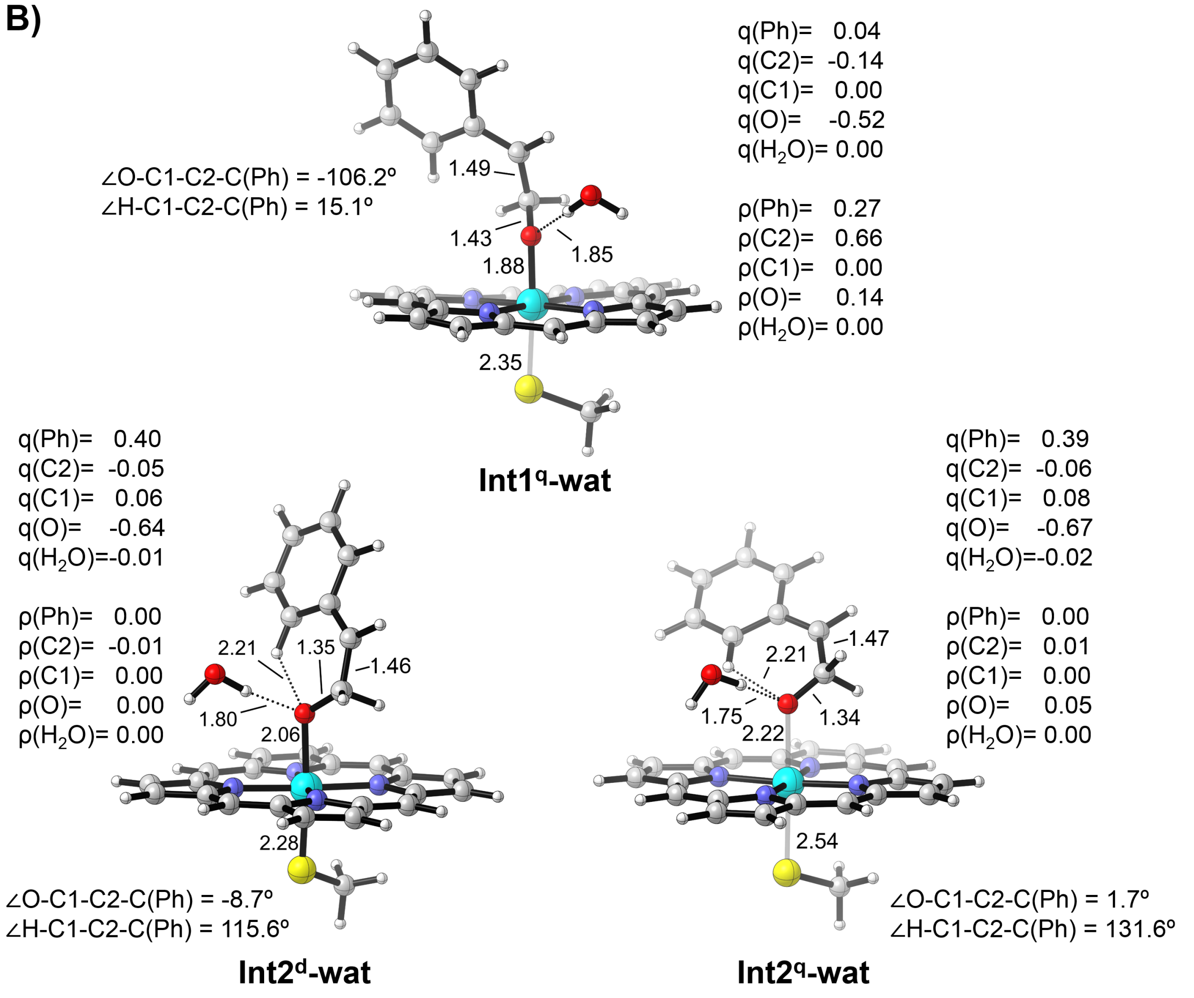

Carbocation intermediate in the quartet electronic state (**Int2^{q}-wat**) is ca. 3 kcal·mol^{-1} stabilized over the radical intermediate (**Int1^{q}-wat**) by the presence of the hydrogen bonding water molecule. The electronic structure of both intermediates is maintained in the presence of the water molecule, having a Mulliken charges and spin density (ρ) distribution similar to those reported in **Figure S1**. Attempts to optimize **Int1^{d}-wat** in the doublet state were unsuccessful, leading to epoxide formation.

Figure S10: Analysis of catalytically relevant near attack conformations (NAC) of styrene (**1**) accessible in P450$_{LA1}$ WT active site, from restrained-MD simulations. Five independent substrate-bound restrained-MD replicas of 500 ns each (2,500 ns total) are carried out.

A) Two different geometric parameters that describe the relative orientation of the substrate in the active site are analyzed: |∠Fe-O-C1-C2| absolute dihedral angle that describes the relative orientation of the C1-C2 double bond with respect to the Fe-oxo; ∠O-C1-C2-C(Ph) dihedral angle describes which substrate enantioface is exposed to the Fe-oxo (*si* face that would lead to *R*-epoxide is characterized by negative values; *re* face that would lead to *S*-epoxide face is characterized by positive values. Dihedral angles and simulation time are given in deg., and ns, respectively.

B) Relative NAC conformations explored by styrene in P450$_{LA1}$ active site along the MD replicas as defined by |∠Fe-O-C1-C2| and ∠O-C1-C2-C(Ph) dihedral angles.

C) Representative snapshots describing the major NAC conformations explored by styrene (**1**) in P450$_{LA1}$ active site as characterized from restrained-MD simulations. Star markers in B) describe each snapshot on the respective MD replica plot.

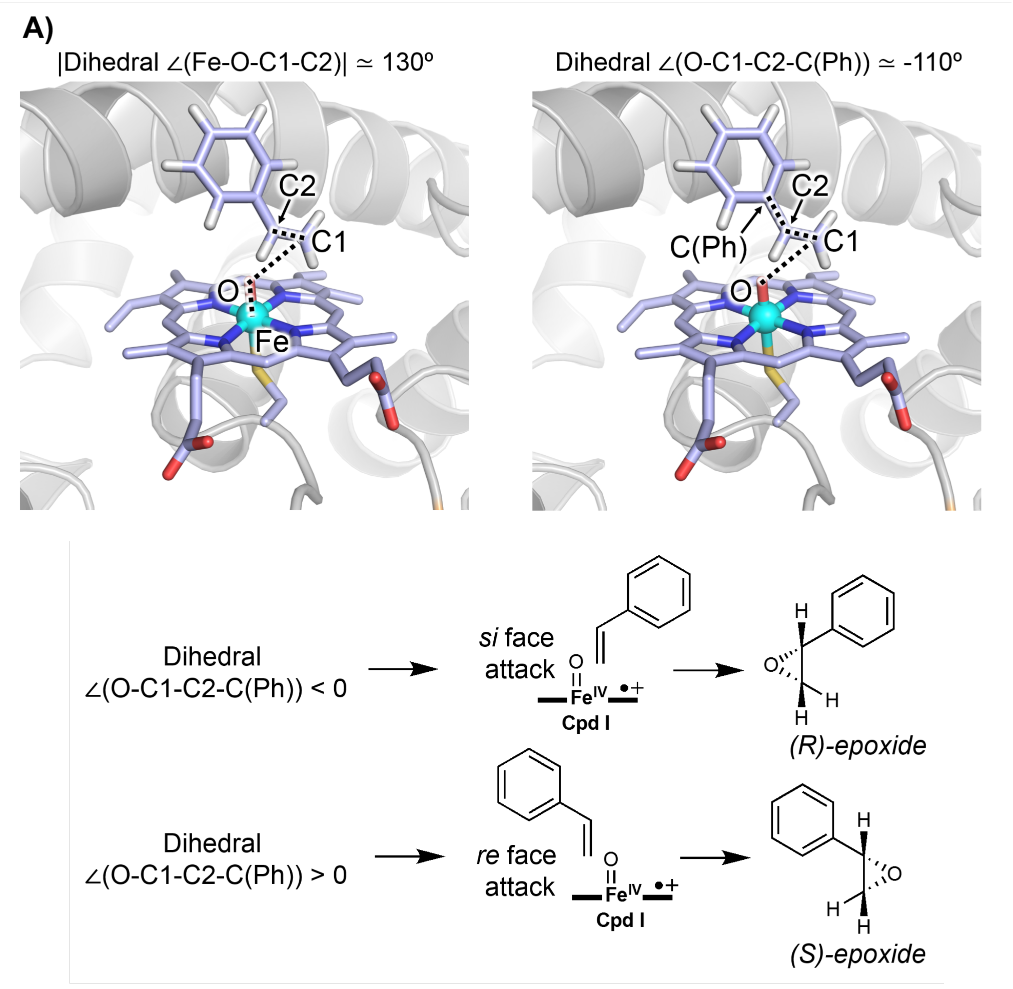

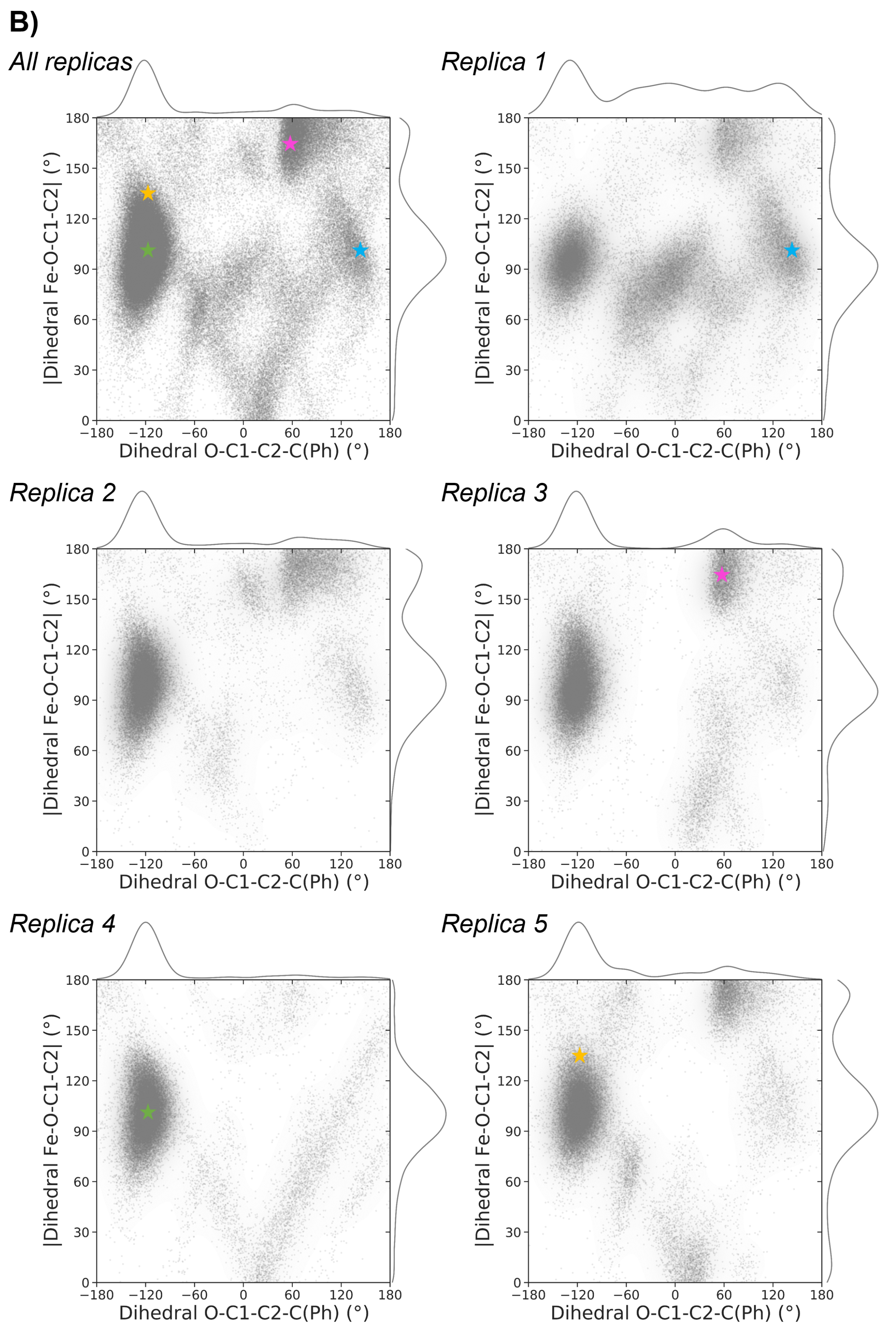
B)
All replicas
Replica 1
Replica 2
Replica 3
Replica 4
Replica 5
|Dihedral Fe-O-C1-C2| (°)
Dihedral O-C1-C2-C(Ph) (°)
−180 −120 −60 0 60 120 180
0 30 60 90 120 150 180

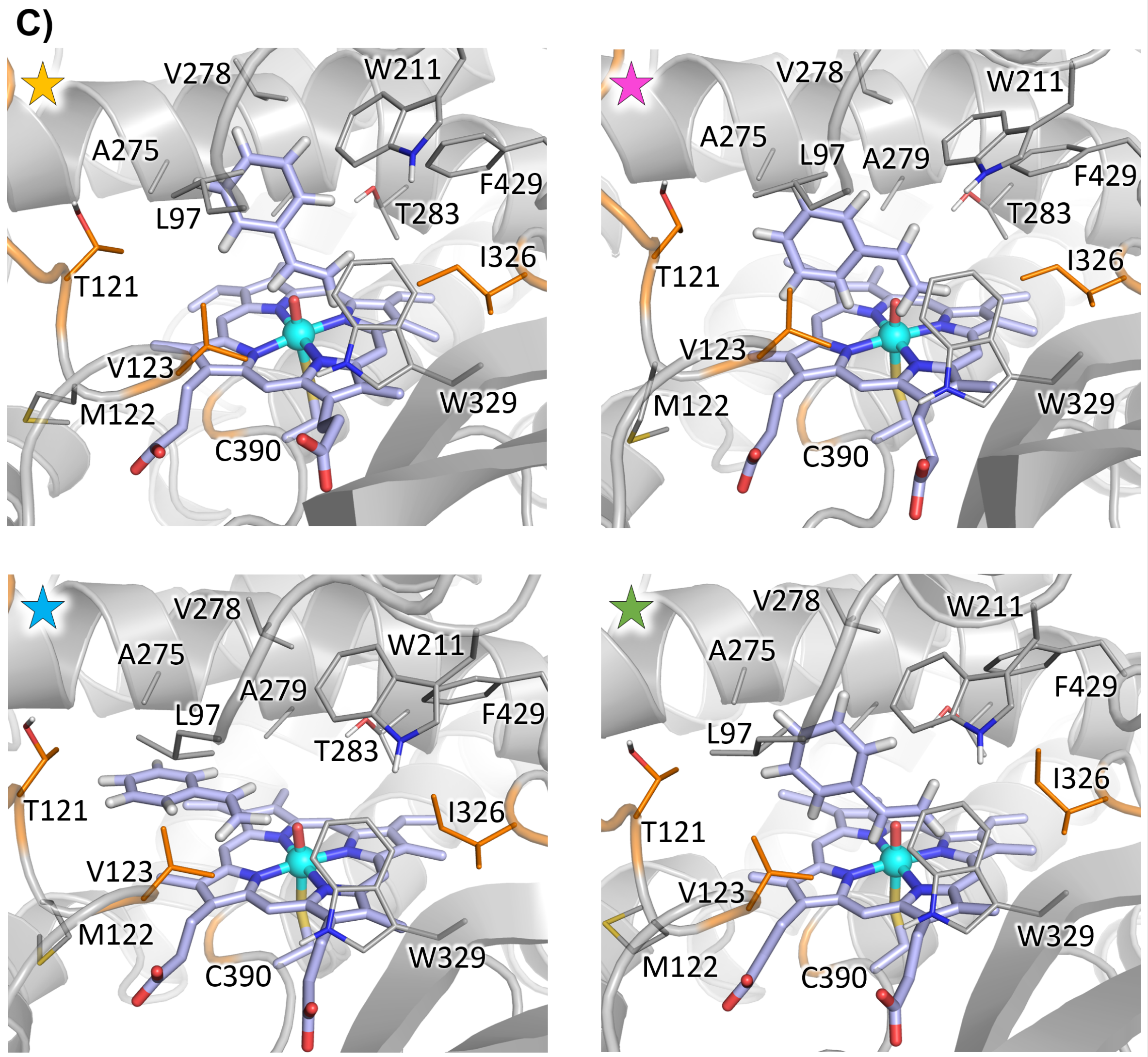

Styrene-bound restrained-MD simulations show that the substrate preferentially explores near-attack conformations (NAC) in P450$_{LA1}$ enzyme that expose the *si* enantioface (negative values for the ∠O-C1-C2-C(Ph) dihedral) as shown in **B**. Catalytically relevant binding poses explored by the substate along restrained-MD simulations are in agreement with the accessible active site volume characterized from holo state simulations in **Figure S7**. Most of the binding poses explored by the substrate have the phenyl group of styrene oriented to the left-handed side of the active site (front view), opposite from the bulky residues W211, F429, I236 and W329 located on the right side of the active site. Binding poses exhibiting *si* enantioface exposed to the iron-oxo species have the substrate C1-C2 double bond orthogonal to the iron oxo moiety (see green and yellow star marker snapshots in **C**) with dihedral angle |∠Fe-O-C1-C2| values comprised between 75º and 135º. Minor binding poses with *re* enantioface exposed to the iron-oxo species (see pink star marker snapshot in **C**) have the C1-C2 double bond in a more parallel orientation with respect to the Fe=O axis, described by dihedral angle |∠Fe-O-C1-C2| values larger than 150º. Binding poses corresponding to the blue star marker are considered as non-reactive due to bad alignment of the *pi* system of the alkene and the iron-oxo species.

Figure S11: Analysis of catalytically relevant near attack conformations (NAC) of styrene (**1**) accessible in aMOx variant active site, from restrained-MD simulations. Five independent substrate-bound restrained-MD replicas of 500 ns each (2,500 ns total) are carried out.

A) Two different geometric parameters that describe the relative orientation of the substrate in the active site are analyzed: |∠Fe-O-C1-C2| absolute dihedral angle that describes the relative orientation of the C1-C2 double bond with respect to the Fe-oxo; ∠O-C1-C2-C(Ph) dihedral angle describes which substrate enantioface is exposed to the Fe-oxo (*si* face that would lead to *R*-epoxide is characterized by negative values; *re* face that would lead to *S*-epoxide face is characterized by positive values. Dihedral angles and simulation time are given in deg., and ns, respectively.

B) Relative NAC conformations explored by styrene in aMOx variant active site along the MD replicas as defined by |∠Fe-O-C1-C2| and ∠O-C1-C2-C(Ph) dihedral angles.

C) Representative snapshots describing the major NAC conformations explored by styrene (**1**) in aMOx variant active site as characterized from restrained-MD simulations. Star markers in B) describe each snapshot on the respective MD replica plot.

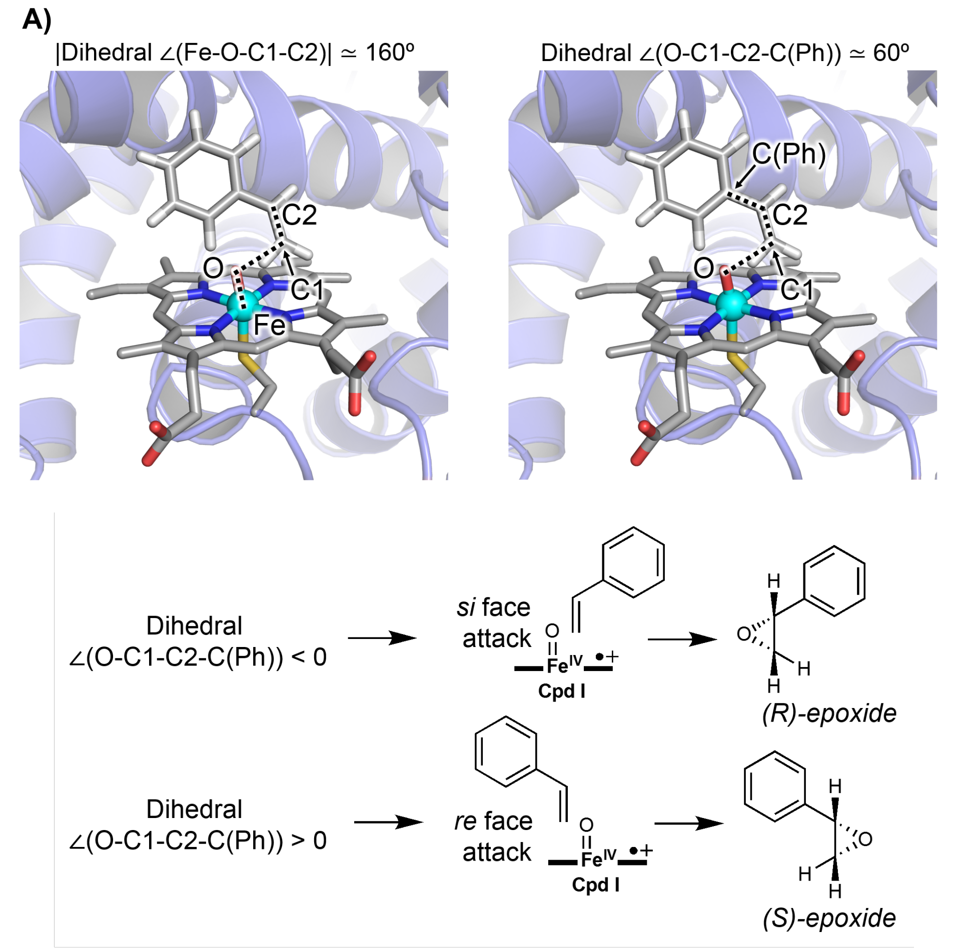

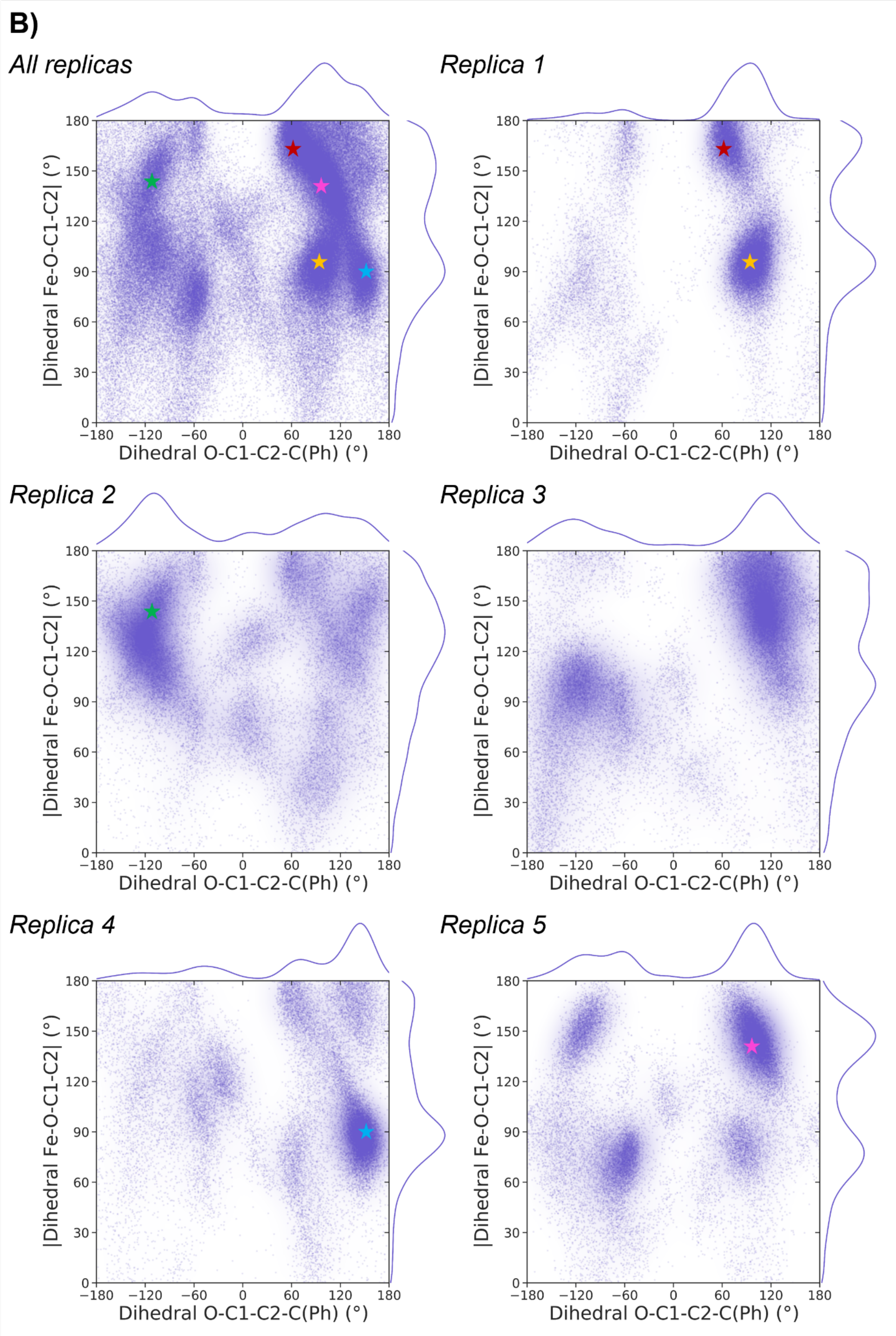
B)
All replicas
Replica 1
Replica 2
Replica 3
Replica 4
Replica 5
|Dihedral Fe-O-C1-C2| (°)
Dihedral O-C1-C2-C(Ph) (°)
−180 −120 −60 0 60 120 180
0 30 60 90 120 150 180

C)

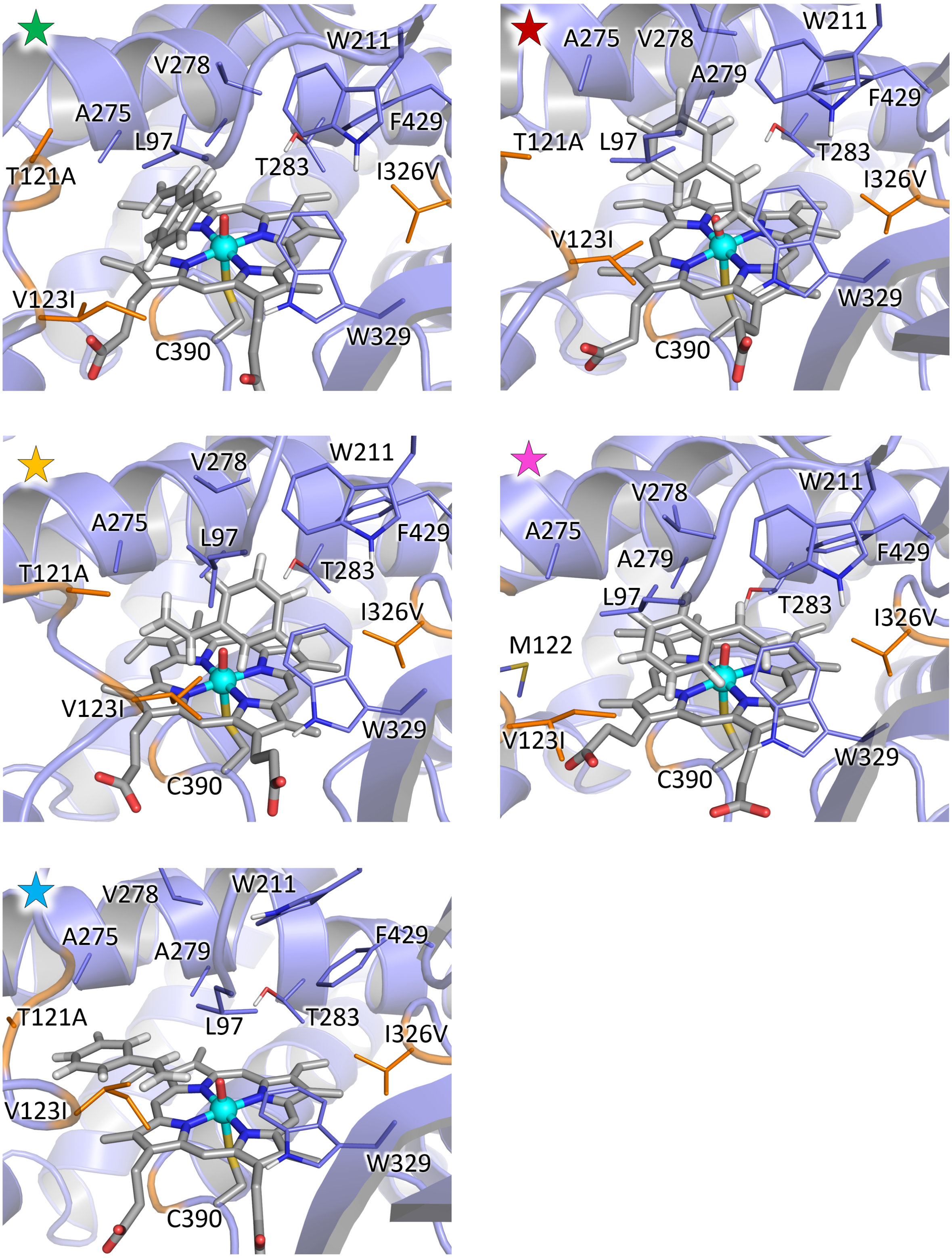
W211
V278
A275
L97
F429
T121A
T283
I326V
V123I
C390
W329
A275
V278
W211
A279
F429
T121A
L97
T283
I326V
V123I
W329
C390
V278
W211
A275
L97
F429
T121A
T283
I326V
V123I
W329
C390
W211
V278
A275
A279
F429
L97
T283
I326V
M122
V123I
C390
W329
V278
W211
A275
A279
F429
T121A
T283
L97
I326V
V123I
W329
C390

Styrene bound restrained-MD simulations describe that, in aMOx evolved active site, the substrate preferentially explores catalytically relevant binding poses (near attack conformations, NAC) exposing its *re* face to the iron-oxo species. This is defined by the preferential exploration of positive values of the ∠O-C1-C2-C(Ph) dihedral angle (**B**) and would lead to the formation of the *S*-epoxide product. These different NAC binding modes observed in aMOx as compared to $P450_{LA1}$ WT enzyme described in **Figure S10**, can be attributed to the reshaping of the active site cavity caused by protein evolution (**Figure S7**).

Preferential, catalytically relevant, NAC binding poses of styrene in aMOx active site are described by the red and pink star marker representative snapshots (shown in **C**), both exposing the *re* enantioface to the iron-oxo, and having the C1-C2 double bond oriented parallel to the Fe=O axis (defined by a large value of the |∠Fe-O-C1-C2| dihedral angle). This parallel relative orientation would facilitate O-C1 bond formation, as opposite to alternative NAC binding poses described by the yellow star marker representative snapshot. I326V mutation facilitates the positioning of the terminal methylidene group (CH_2=) in this region of the active site. The position that styrene occupies in the aMOx active site in these two catalytically relevant binding poses (red and pink star marker representative snapshots), matches the accessible active site volume characterized from *holo* state aMOx MD simulations (**Figure S7**). Within these NAC binding poses, the phenyl ring of the substrate is oriented to the left-handed of the active site (from a front view), opposite to the bulky residues (W211, W329, F429) that are located on the right-handed side of the active site pocket. Styrene phenyl ring is stabilized in these catalytically relevant binding modes by hydrophobic interactions with L97, V123I, V278 and A279.

Minor NAC binding modes orienting the *si* face of styrene to the iron-oxo species, are described by the green star marker representative snapshot (**C**). There, the phenyl group of styrene still occupies a similar position in the active site than in the re face oriented binding poses (see pink marker snapshot), but with the terminal olefin oriented to the opposite side of the active site pocket. Binding poses corresponding to the blue star marker are considered as non-reactive due to bad alignment of the *pi* system of the alkene and the iron-oxo species.

Figure S12: Analysis of catalytically relevant near attack conformations (NAC) of styrene (**1**) accessible in P7 active site (T121A, N201K, N209S, Y385H, E418G) from restrained-MD simulations. Five independent substrate-bound restrained-MD replicas of 500 ns each (2,500 ns total) are carried out.

A) Two different geometric parameters that describe the relative orientation of the substrate in the active site are analyzed: |∠Fe-O-C1-C2| absolute dihedral angle that describes the relative orientation of the C1-C2 double bond with respect to the Fe-oxo; ∠O-C1-C2-C(Ph) dihedral angle describes which substrate enantioface is exposed to the Fe-oxo (*si* face that would lead to *R*-epoxide is characterized by negative values; *re* face that would lead to *S*-epoxide face is characterized by positive values). Dihedral angles and simulation time are given in deg., and ns, respectively.

B) Relative NAC conformations explored by styrene in P7 active site along the MD replicas as defined by |∠Fe-O-C1-C2| and ∠O-C1-C2-C(Ph) dihedral angles.

C) Representative snapshots describing the major NAC conformations explored by styrene (**1**) in P7 active site as characterized from restrained-MD simulations. Star markers in B) describe each snapshot on the respective MD replica plot.

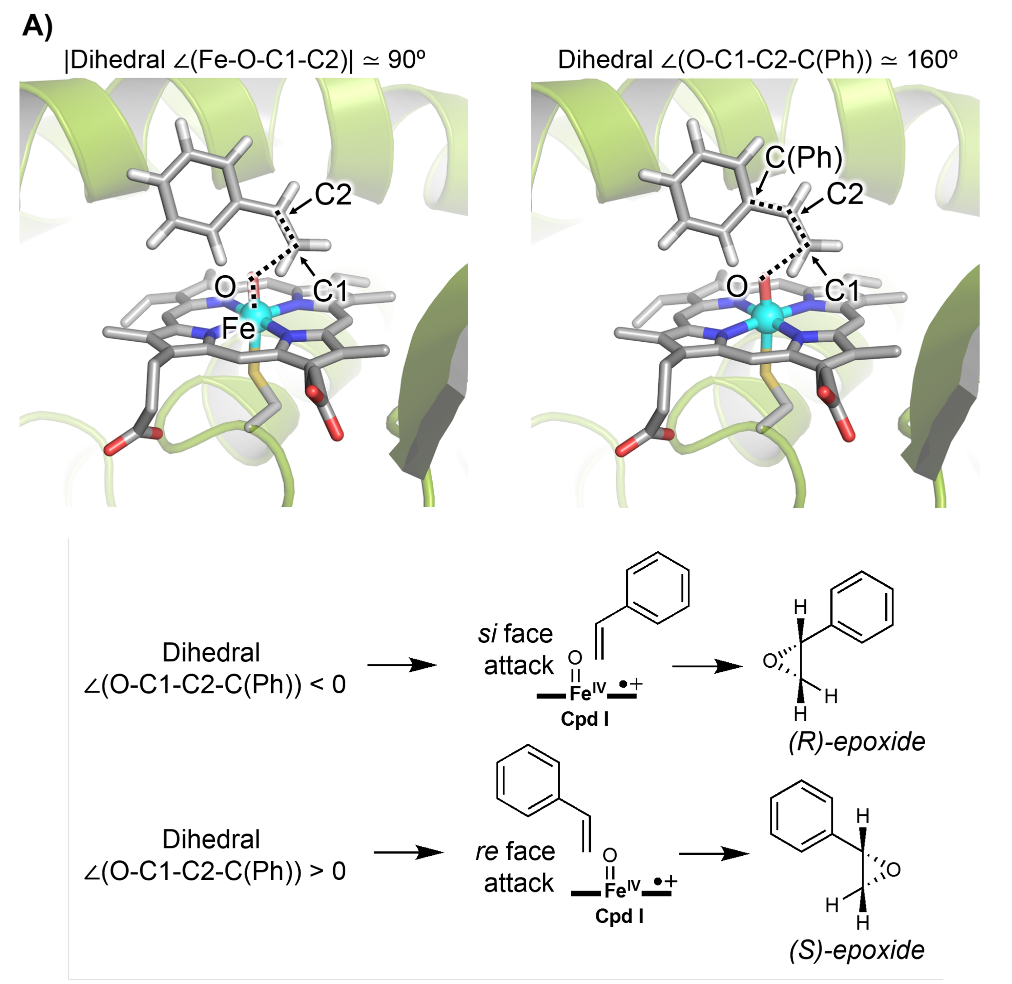

B)

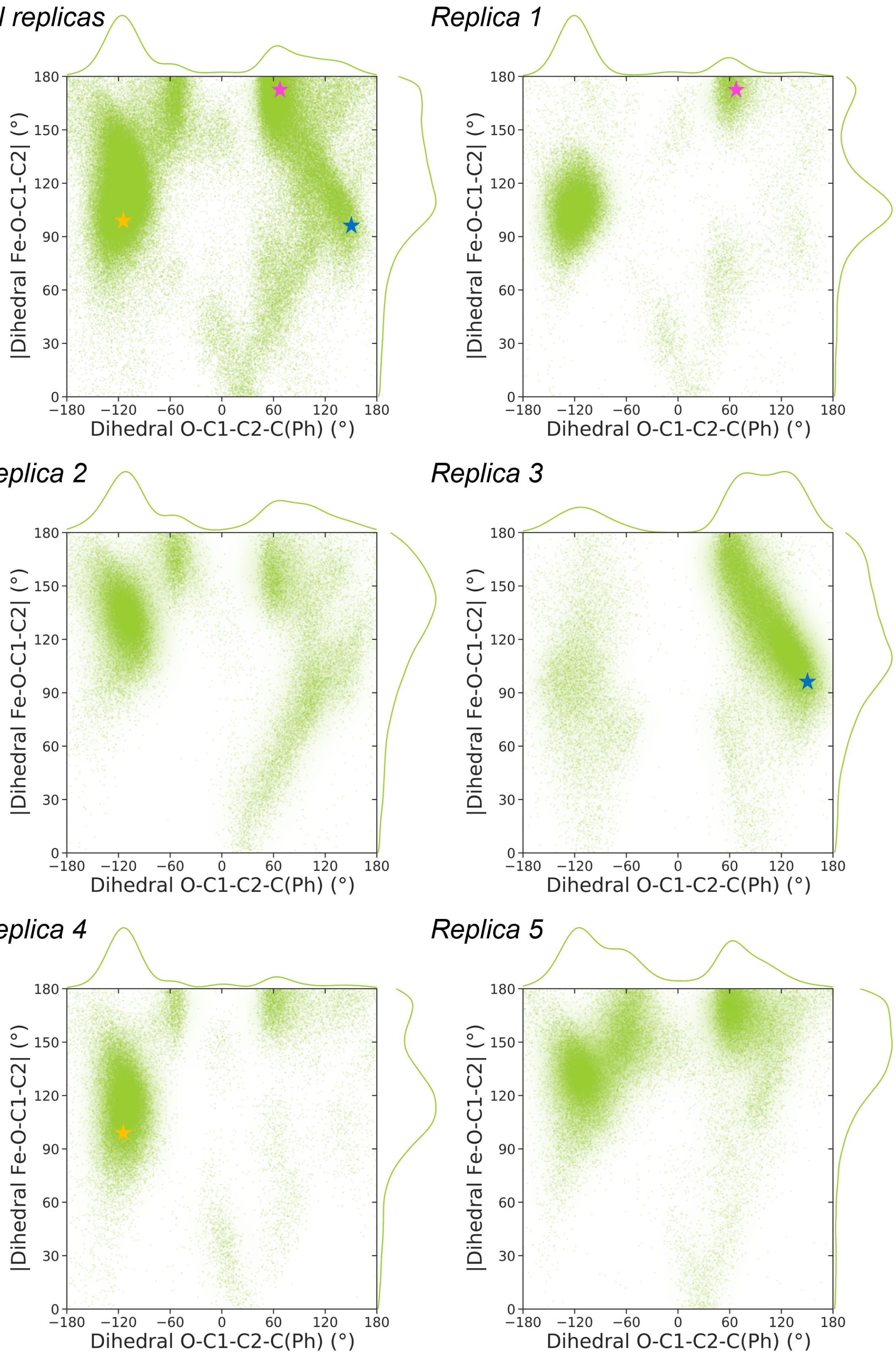
All replicas
Replica 1
Replica 2
Replica 3
Replica 4
Replica 5
|Dihedral Fe-O-C1-C2| (°)
Dihedral O-C1-C2-C(Ph) (°)
0
30
60
90
120
150
180
−180
−120
−60
0
60
120
180

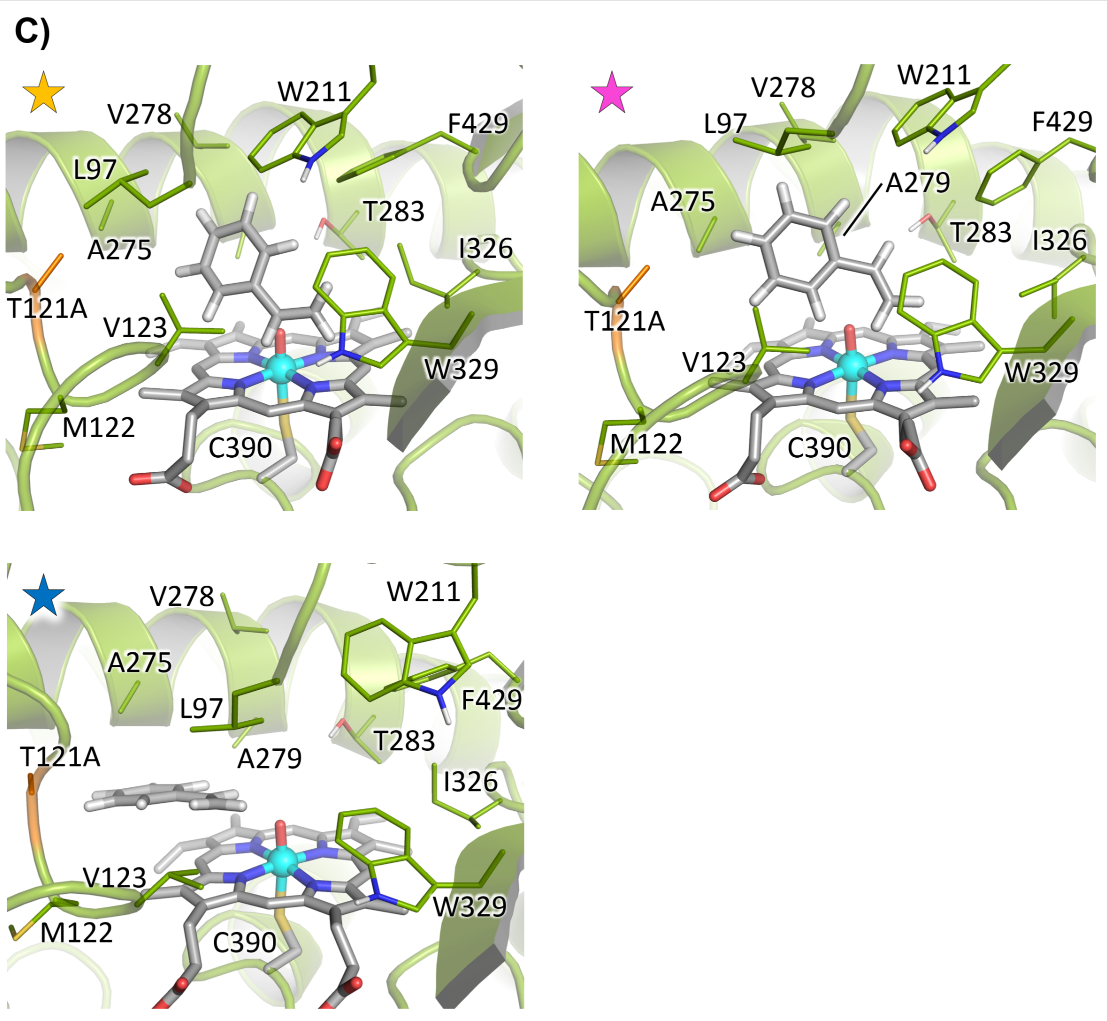

Styrene-bound restrained-MD simulations showed that in P7 variant styrene (**1**) substrate can effectively explore catalytically relevant binding modes in which the *re* face (pro-*S* epoxidation, pink star in **C**) is exposed to the iron-oxo species more frequently than in the WT (see **Figure S10**). However, *si* face catalytically active binding modes are still easily explored (negative values for the ∠O-C1-C2-C(Ph) dihedral as shown in **B**, see yellow star in **C**). This is in line with the higher epoxide (*S*)-selectivity exhibited by P7 variant (ca. 50% (*S*)-epoxide, **Figure 4E** from the main text) as compared to WT $P450_{LA1}$ (40% (*S*)-epoxide), and the lower selectivity as compared to aMOx variant (ca. 86% (S)-epoxide).

Consequently, these new simulations support the computationally identified reshaping of the active site during evolution and its impact on the preferential substrate binding mode and enzyme selectivity experimentally observed.

Figure S13: Conformational analysis based on MD simulations of the covalent radical intermediate (**Int1**) formed in **A**) P450$_{LA1}$ WT enzyme and **B**) aMOx variant. Two independent MD trajectories (500 ns each) were carried out for each system.

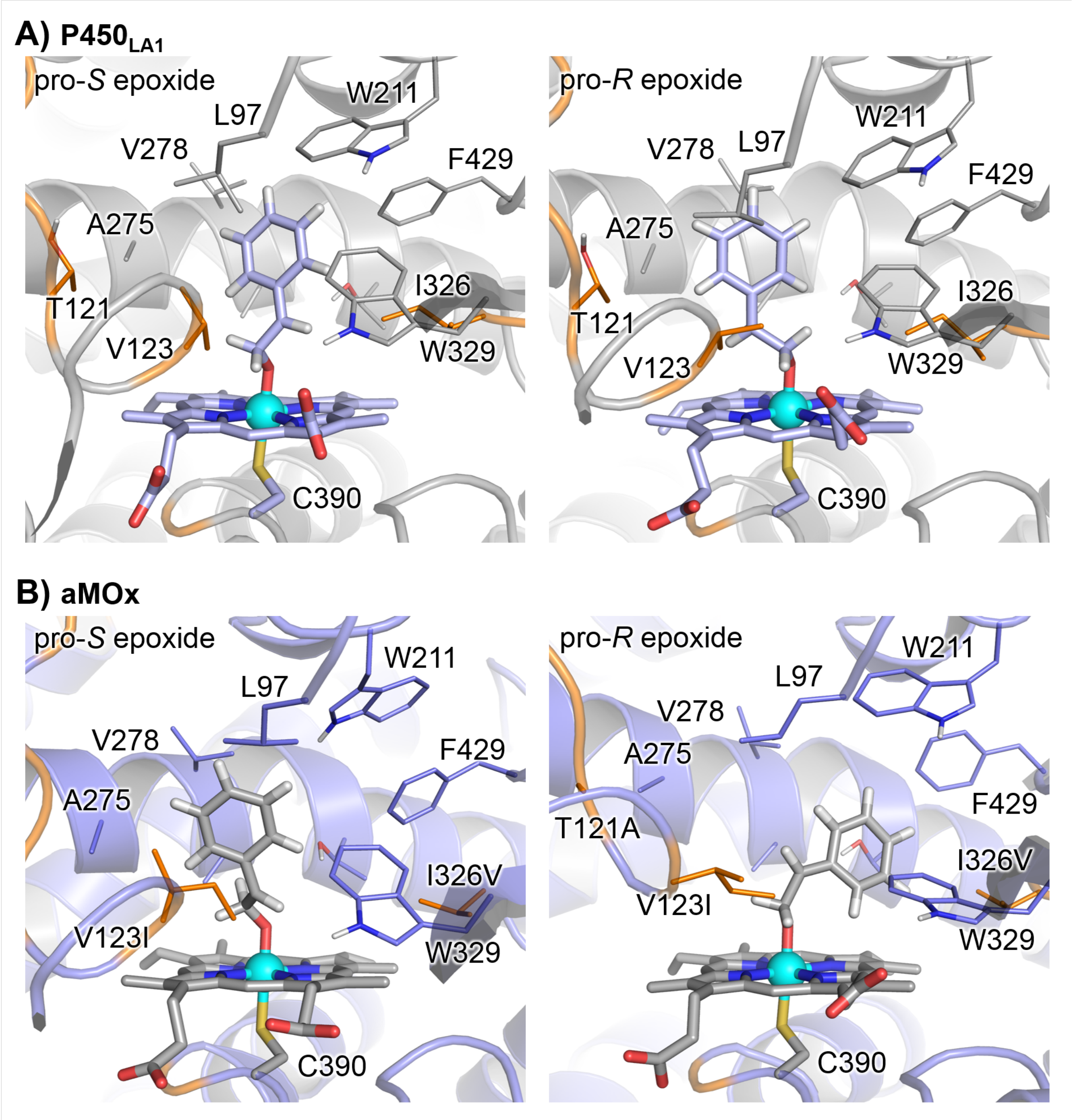

MD simulations were used to perform a conformational analysis of the covalent radical intermediate (**Int1**) when formed in P450$_{LA1}$ and aMOx active sites. Two representative snapshots for each variant, with the intermediate exploring pro-*S* and pro-*R* epoxidation conformations, were selected based on clustering analyses. These conformations are very similar to the major near attack conformations characterized from substrate-bound restrained-MD simulations (**Figures S10** and **S11**). The selected intermediate conformations were used as a starting point for QM/MM calculations.

v. Computational modelling of the enzymatic reaction mechanisms using QM/MM calculations

Figure S14: QM/MM exploration of P450$_{LA1}$ WT catalyzed oxidation of styrene (**1**) substrate. QM/MM calculations were carried out starting from a MD-relaxed structure of the covalent intermediate (**Int1**) formed in P450$_{LA1}$ active site (**Figure S13-A**), that mimics the minor explored, catalytically relevant, binding pose of styrene substrate characterized from restrained-MD simulations (pink marker selected snapshot in **Figure S10)**. This corresponds to a styrene binding pose with its *re* face exposed to the iron-oxo species (pro-*S* epoxidation).

A) QM/MM computed relative stabilities in terms of electronic energy at the QM region (ΔE_{QM}), QM/MM ONIOM electronic energy (ΔE), enthalpy (ΔH), and Gibbs energy (ΔG) for the different optimized species. Energy values were obtained at the (U)B3LYP/Def2TZVP: AmberFF14SB//(U)B3LYP/6- 31G(d)+SDD(Fe):AmberFF14SB level, with the same MM parameters used in MD simulations. An Electrostatic Embedding was used (see computational details). Doublet (d) and quartet (q) electronic states were considered, and all energies are referred considering the lowest in energy LA1-**1**^{q} structure as zero.

B) QM/MM calculated Gibbs free energy profile. Relative Gibbs free energies (ΔG) and electronic energies (ΔE, in parenthesis) are reported.

C) QM/MM optimized structures. Atoms included in the QM region are shown in ball-and-stick representation, and residues in the MM region are shown in sticks. Mutated residues are highlighted in orange. Mulliken charges (*q*) and spin density (ρ) values for the phenyl group (Ph, sum of all C and H atoms), C2 benzylic position, C1 and O, are reported.

Energies, distances, and Mulliken charges and spin density values are given in kcal·mol^{-1}, Angstrom (Å), and a.u., respectively.

A)

Structure	Electronic State	ΔE_{QM}	ΔE	ΔH	ΔG
LA1-**1**	doublet (d)	0.0	0.0	0.0	0.2
	quartet (q)	0.0	0.0	0.0	0.0
LA1-**TS1**	doublet (d)	13.5	17.5	16.9	19.3
	quartet (q)	15.0	18.8	17.5	19.1
LA1-**Int1**	doublet (d)	-8.7	-3.4	-3.3	-1.2
	quartet (q)	-6.0	-4.7	-4.1	-3.6
LA1-**TS2**	quartet (q)	-7.3	-2.8	-2.6	-1.6
LA1-**2-S**	doublet (d)	-27.6	-20.6	-18.0	-14.9
	quartet (q)	-35.9	-23.0	-20.5	-19.2
LA1-**Int2**	doublet (d)	-9.0	-4.0	-3.8	-1.0
	quartet (q)	-15.3	-3.2	-3.6	-2.4
LA1-**TS3-cis-re**	doublet (d)	-5.2	0.3	-0.6	2.3
	quartet (q)	-14.0	-3.6	-4.9	-3.3
LA1-**TS3-trans-si**	doublet (d)	-7.2	-2.7	-3.3	0.6
	quartet (q)	-13.5	-3.3	-4.4	-2.8
LA1-**3**	doublet (d)	-54.4	-52.7	-51.2	-50.0
	quartet (q)	-64.1	-57.3	-55.7	-55.9

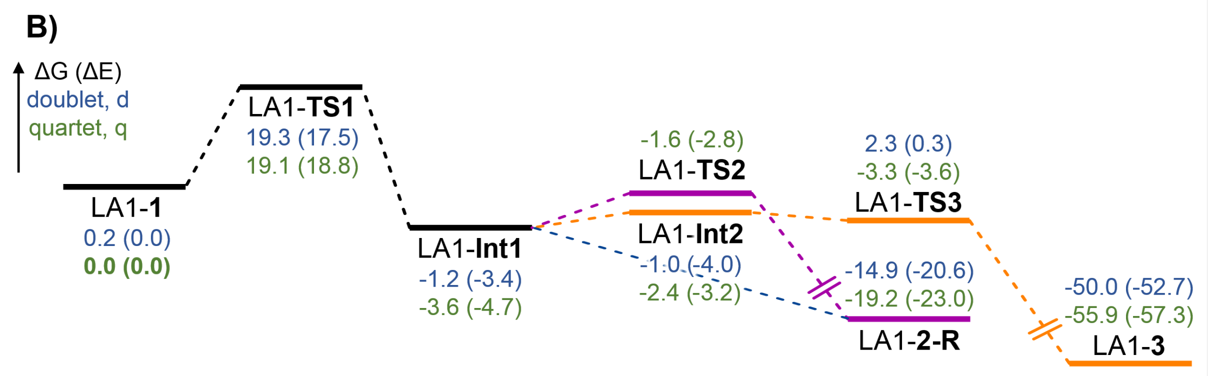

C)

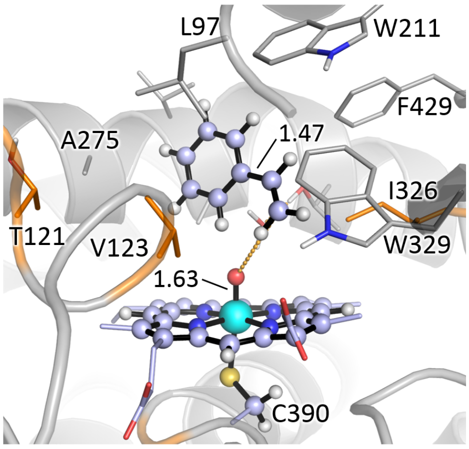

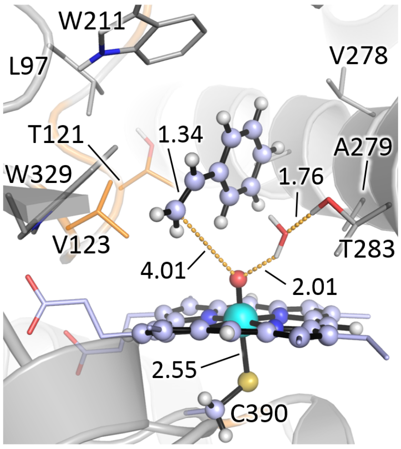

View 1 View 2

LA1-1[d]

ΔG= 0.2 (ΔE= 0.0)

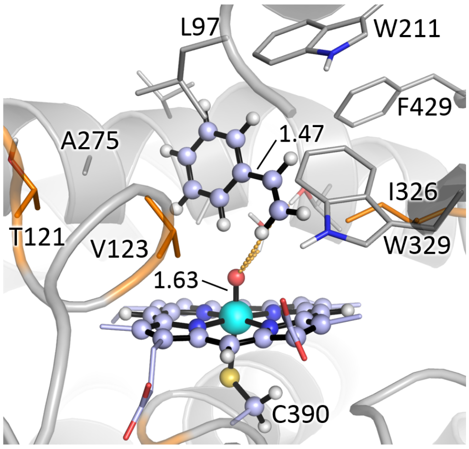

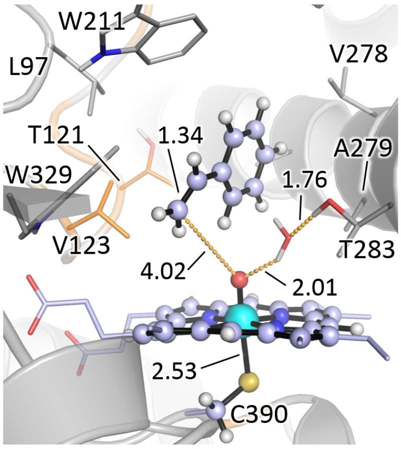

View 1 View 2

LA1-1[q]

ΔG= 0.0 (ΔE= 0.0)

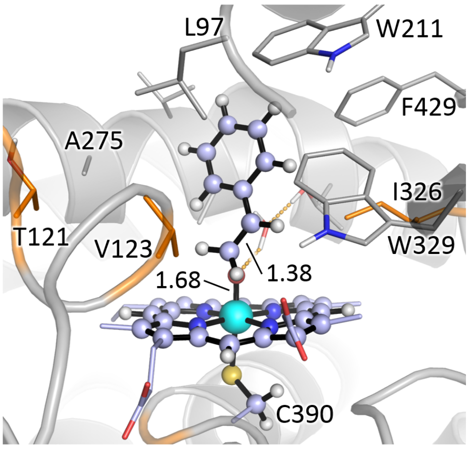

View 1

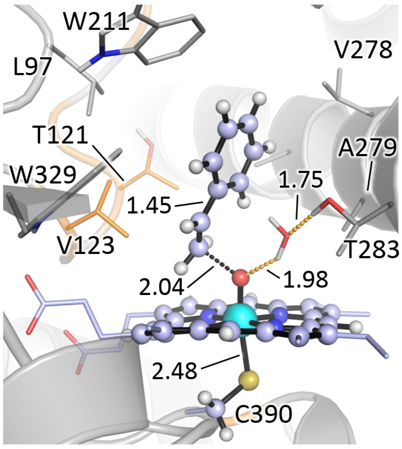

View 2

q(Ph) = 0.05
q(C2) = -0.06
q(C1) = -0.16
q(O) = -0.51

ρ(Ph) = -0.11
ρ(C2) = -0.27
ρ(C1) = 0.00
ρ(O) = 0.25

LA1-**TS1**[d]
$\Delta G^{\ddagger}$= 19.1 ($\Delta E^{\ddagger}$= 17.5)

|∠Fe-O-C1-C2| = 142.4º

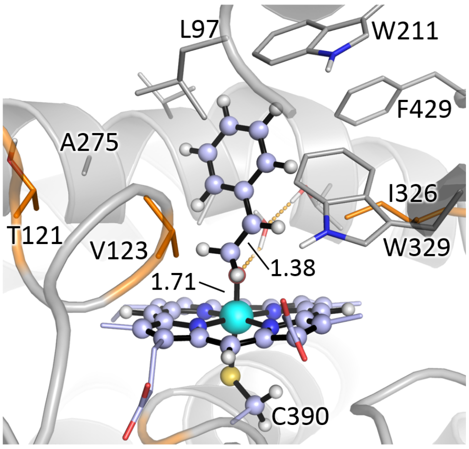

View 1

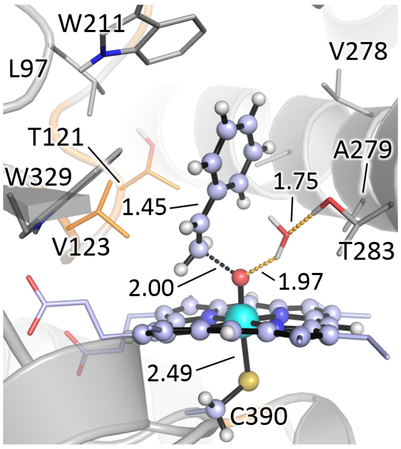

View 2

q(Ph) = -0.01
q(C2) = -0.08
q(C1) = -0.16
q(O) = -0.49

ρ(Ph) = 0.11
ρ(C2) = 0.34
ρ(C1) = -0.09
ρ(O) = 0.65

LA1-**TS1**[q]
$\Delta G^{\ddagger}$= 19.1 ($\Delta E^{\ddagger}$= 18.8)

|∠Fe-O-C1-C2| = 143.1º

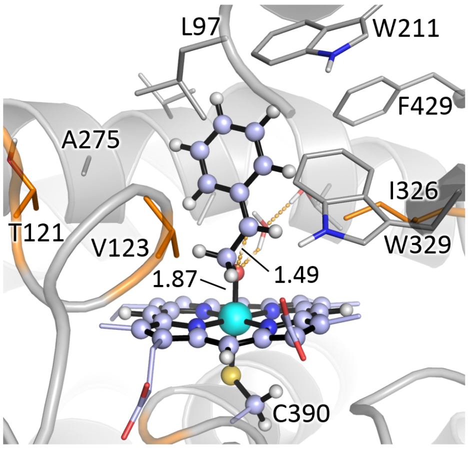

View 1

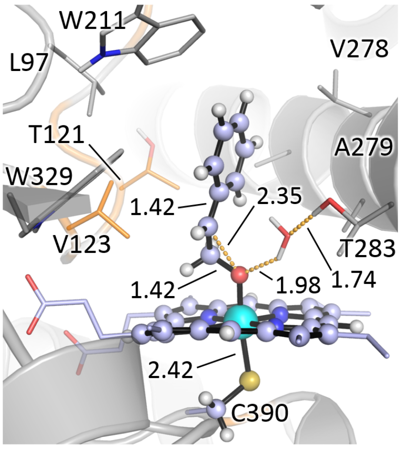

View 2

q(Ph) = -0.02
q(C2) = -0.05
q(C1) = 0.01
q(O) = -0.52

ρ(Ph) = 0.23
ρ(C2) = 0.56
ρ(C1) = -0.01
ρ(O) = 0.05

LA1-**Int1**[d]
ΔG_r= -1.4 (ΔE_r= -3.4)
$\Delta\Delta G$= 2.4 ($\Delta\Delta E$= 1.3)

∠O-C1-C2-C(Ph) = 98.9º
∠H-C1-C2-C(Ph) = -22.6º

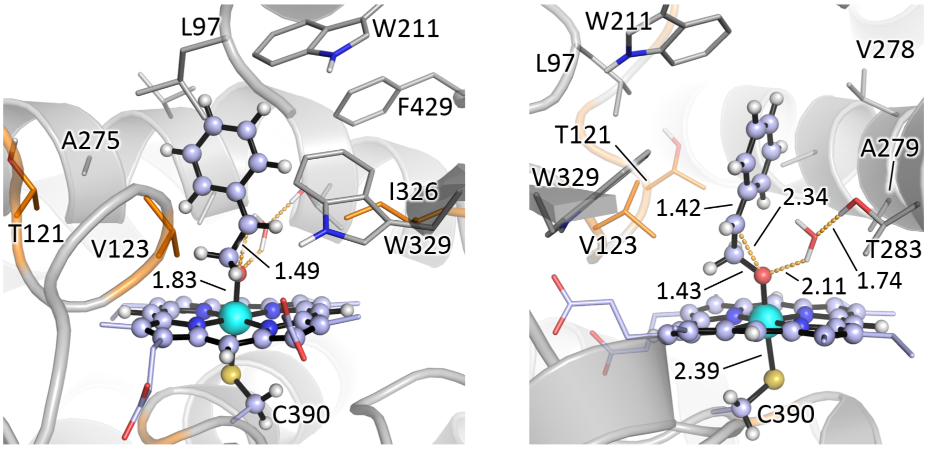

View 1 View 2

q(Ph) = -0.04
q(C2) = -0.09
q(C1) = -0.01
q(O) = -0.44

ρ(Ph) = 0.27
ρ(C2) = 0.65
ρ(C1) = 0.00
ρ(O) = 0.14

LA1-**Int1**[q]
ΔG_r= -3.6 (ΔE_r= -4.7)
$\Delta\Delta G$= 0.0 ($\Delta\Delta E$= 0.0)

∠O-C1-C2-C(Ph) = 100.2°
∠H-C1-C2-C(Ph) = -20.5°

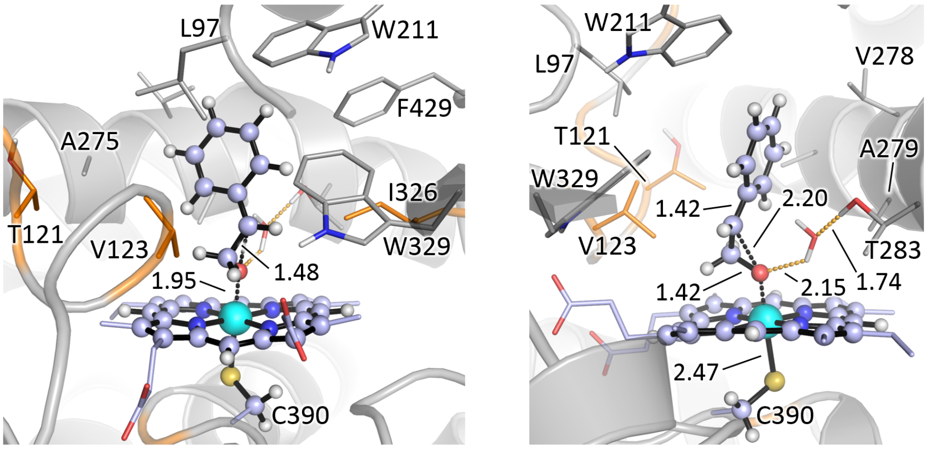

View 1 View 2

q(Ph) = 0.00
q(C2) = 0.00
q(C1) = 0.00
q(O) = 0.00

ρ(Ph) = 0.00
ρ(C2) = 0.00
ρ(C1) = 0.00
ρ(O) = 0.00

LA1-**TS2**[q]
$\Delta G^‡$= 2.0 ($\Delta E^‡$= 1.8)

∠O-C1-C2-C(Ph) = 97.4°

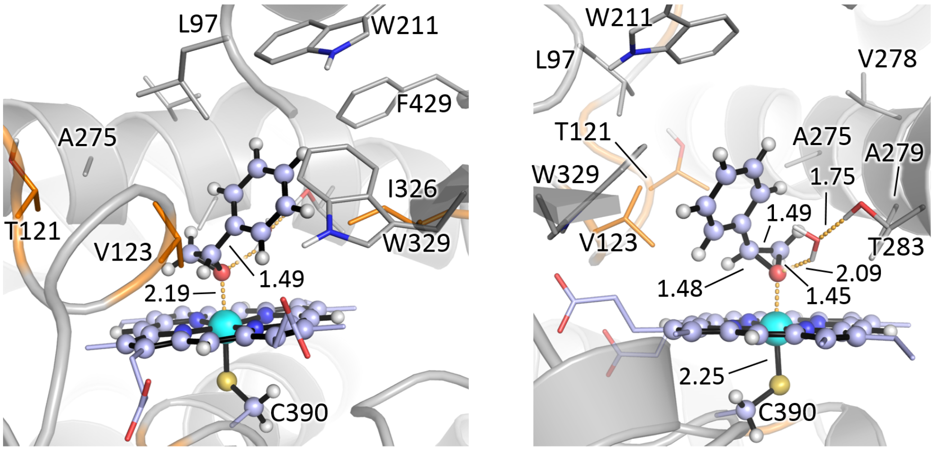

View 1 View 2

LA1-**2-S**[d]
ΔG_r= -13.7 (ΔE_r= -17.2)

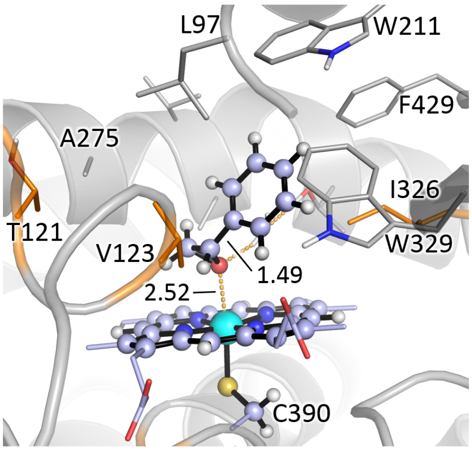

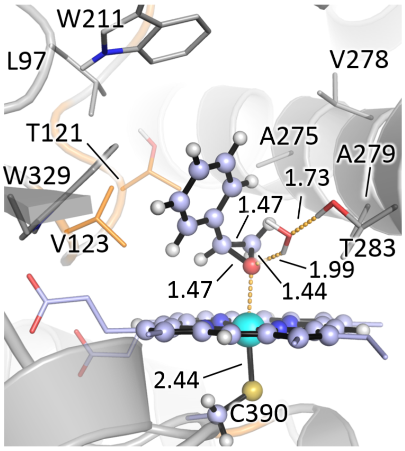

View 1 View 2

LA1-**2-S**[q]

ΔG_r= -15.6 (ΔE_r= -18.3)

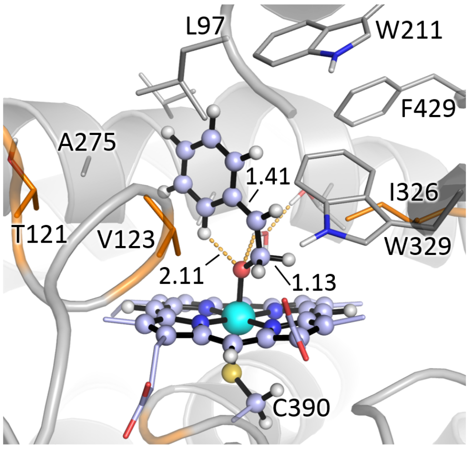

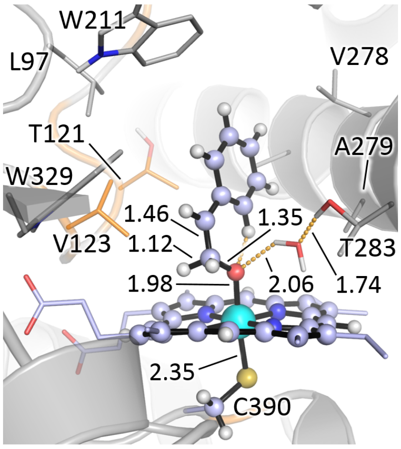

q(Ph) = 0.27
q(C2) = -0.07
q(C1) = 0.03
q(O) = -0.58

ρ(Ph) = -0.03
ρ(C2) = -0.08
ρ(C1) = 0.01
ρ(O) = 0.03

View 1 View 2

LA1-**Int2**[d]

ΔΔG= 0.2 (ΔΔE= -0.6)

∠O-C1-C2-C(Ph) = 7.1°
∠H-C1-C2-C(Ph) = -123.1°

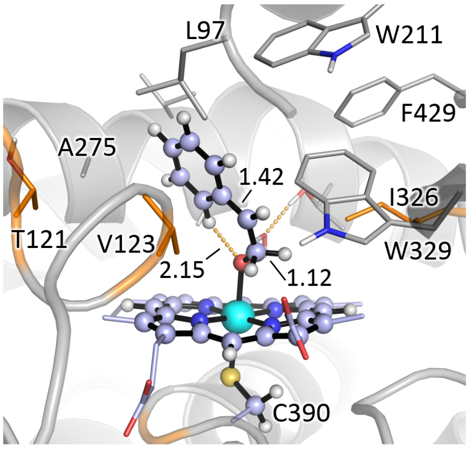

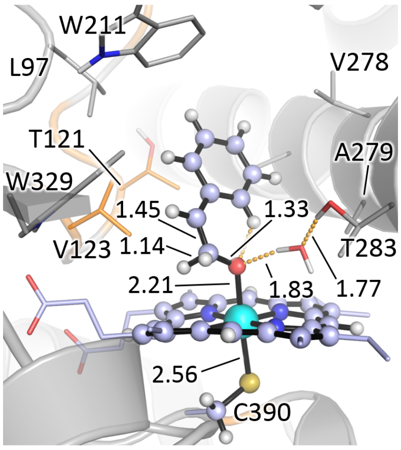

q(Ph) = 0.29
q(C2) = -0.08
q(C1) = 0.07
q(O) = -0.66

ρ(Ph) = 0.00
ρ(C2) = 0.00
ρ(C1) = 0.01
ρ(O) = 0.06

View 1 View 2

LA1-**Int2**[q]

ΔΔG= 1.2 (ΔΔE= 1.5)

∠O-C1-C2-C(Ph) = 4.8°
∠H-C1-C2-C(Ph) = -120.2°

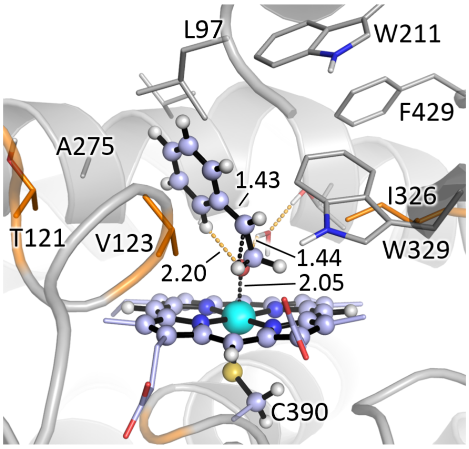

View 1

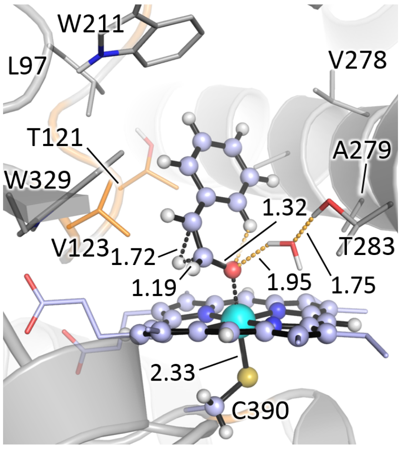

View 2

q(Ph) = 0.00
q(C2) = 0.00
q(C1) = 0.00
q(O) = 0.00

ρ(Ph) = 0.00
ρ(C2) = 0.00
ρ(C1) = 0.00
ρ(O) = 0.00

LA1^S-**TS3-cis-re**^d
ΔG^‡= 3.3 (ΔE^‡= 4.3)

∠H-C1-C2-C(Ph) = -105.6º

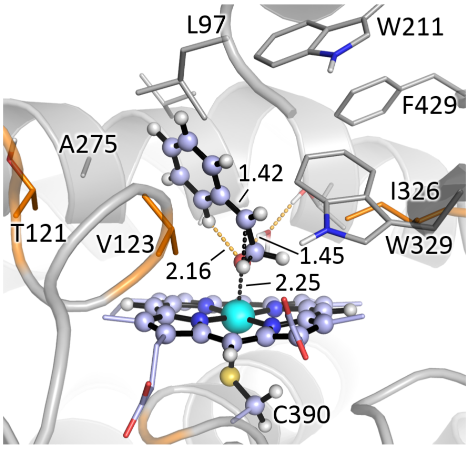

View 1

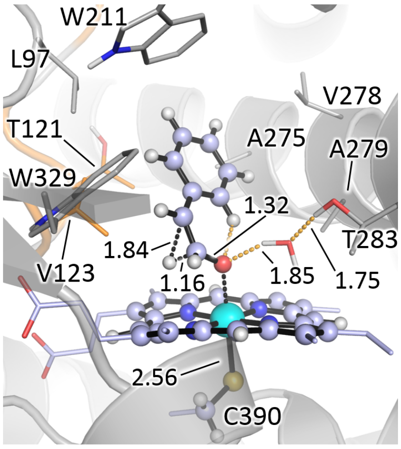

View 2

q(Ph) = 0.27
q(C2) = -0.08
q(C1) = 0.06
q(O) = -0.66

ρ(Ph) = 0.00
ρ(C2) = 0.00
ρ(C1) = 0.01
ρ(O) = 0.05

LA1^S-**TS3-cis-re**^q
ΔG^‡= -0.9 (ΔE^‡= -0.4)

∠H-C1-C2-C(Ph) = -109.7º

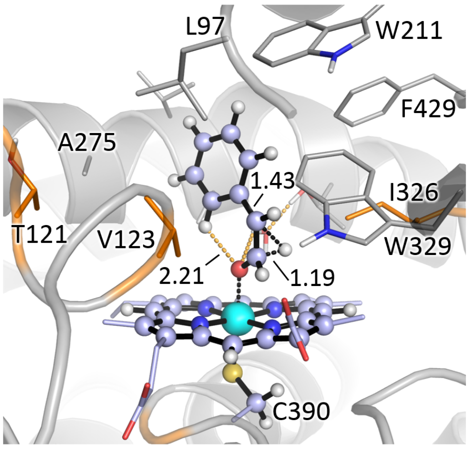

View 1

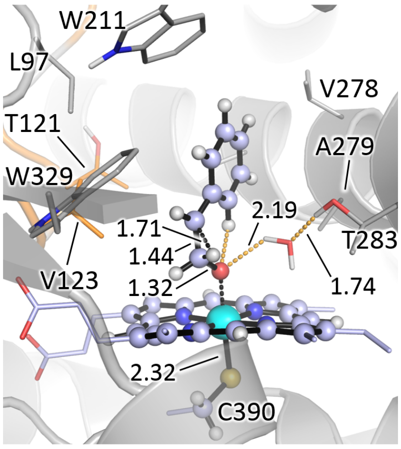

View 2

q(Ph) = 0.23
q(C2) = -0.09
q(C1) = 0.04
q(O) = -0.53

ρ(Ph) = 0.00
ρ(C2) = 0.00
ρ(C1) = 0.00
ρ(O) = 0.00

LA1^S-**TS3-trans-si**^d
ΔG^‡= 1.6 (ΔE^‡= 1.3)

∠H-C1-C2-C(Ph) = 108.4º

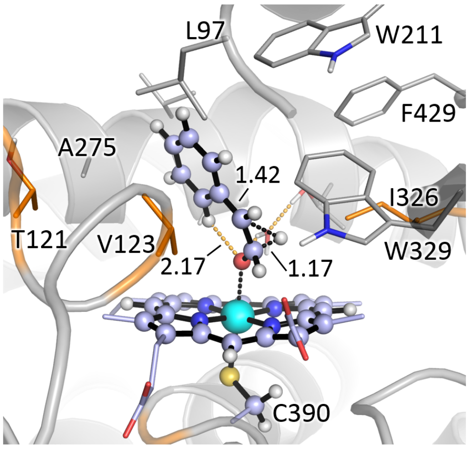

View 1

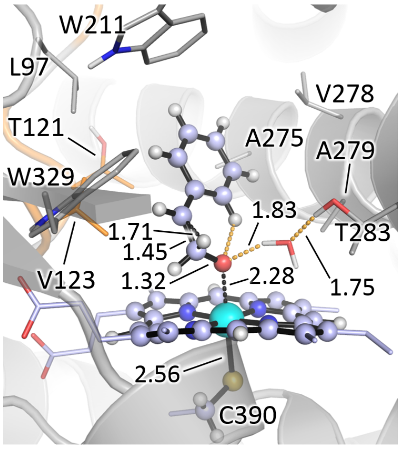

View 2

q(Ph) = 0.27
q(C2) = -0.09
q(C1) = 0.06
q(O) = -0.65

ρ(Ph) = 0.00
ρ(C2) = 0.00
ρ(C1) = 0.01
ρ(O) = 0.05

LA1^{S}-**TS3-trans-si**^{q}
$\Delta G^{\ddagger}$= -0.4 ($\Delta E^{\ddagger}$= -0.1)

∠H-C1-C2-C(Ph) = 110.7º

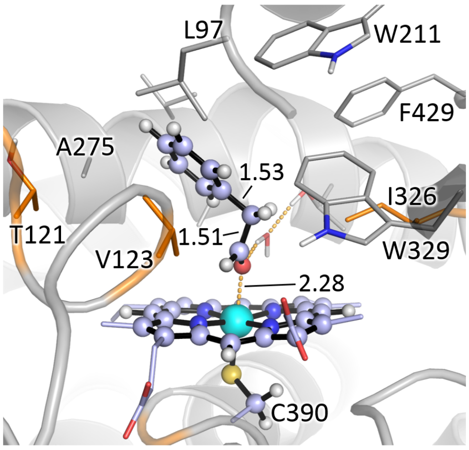

View 1

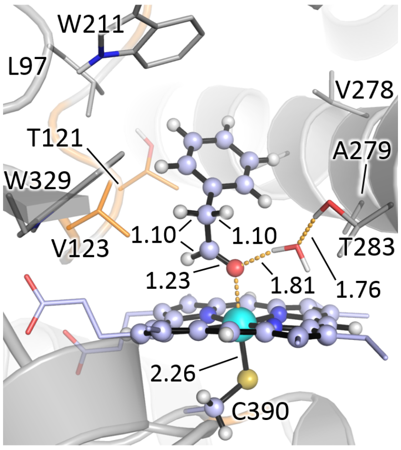

View 2

LA1^{S}-**3**^{d}
ΔG_r= -49.0 (ΔE_r= -48.7)

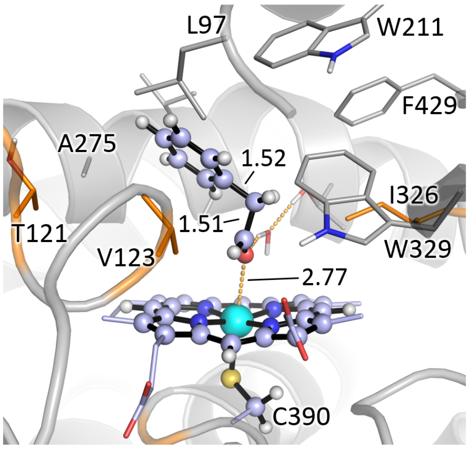

View 1

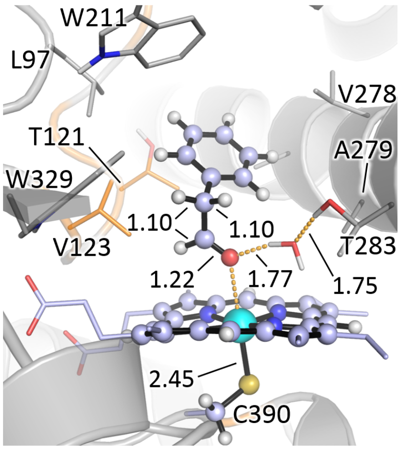

View 2

LA1^{S}-**3**^{q}
ΔG_r= -53.6 (ΔE_r= -54.1)

The particular substrate bound conformation in a catalytically relevant pose explored here, exposing the *re* face to the iron-oxo species, corresponds to the minor NAC binding pose characterized from MD simulations (see **Figure S10**). This particular binding mode leads to the formation of the *S*-epoxide product.

The calculated QM/MM pathways for the styrene epoxidation and carbonyl formation for this minor NAC binding mode are qualitatively equivalent to the ones calculated in **Figure S15** for the major NAC (*si* face exposed to the iron-oxo species) characterized from MD simulations (see **Figure S10**).

For the reaction occurring in $P450_{LA1}$ active site, the covalent radical intermediate LA1-**Int1** in the quartet and doublet electronic states can be optimized as a minimum on the potential energy surface (PES). This is indicating that the enzyme active site can stabilize these reactive intermediates due to confinement, as compared to the free-enzyme model system (see **Figure S1**).

Epoxidation pathway and carbonyl formation pathway are both energetically accessible and both pathways can compete, similar to enzyme-free system (see **Figure S1**). Epoxidation is slightly favored in the doublet state, being barrierless from LA1-**Int1^{d}** (LA1-**TS2^{d}** could not be optimized), and carbonyl formation is slightly favored in the quartet state after a required conformational change of LA1-**Int1^{q}** to form LA1-**Int2^{q}**.

QM/MM calculations for the aldehyde formation pathway describe a barrierless 1,2-hydride migration from LA1-**Int2^{q}** intermediate, being the LA1-**TS3-cis-re** migration transition state favored over LA1-**TS3-trans-si** one.

Figure S15: QM/MM exploration of P450$_{LA1}$ WT catalyzed oxidation of styrene (**1**) substrate. QM/MM calculations were carried out starting from a MD-relaxed structure of the covalent intermediate (**Int1**) formed in P450$_{LA1}$ active site (**Figure S13-A**), that mimics the most populated, catalytically relevant, binding pose of styrene substrate characterized from restrained-MD simulations (green marker selected snapshot in **Figure S10)**. This corresponds to a styrene binding pose with its *si* face exposed to the iron-oxo species (pro-*R* epoxidation).

A) QM/MM computed relative stabilities in terms of electronic energy at the QM region (ΔE_{QM}), QM/MM ONIOM electronic energy (ΔE), enthalpy (ΔH), and Gibbs energy (ΔG) for the different optimized species. Energy values were obtained at the (U)B3LYP/Def2TZVP: AmberFF14SB//(U)B3LYP/6- 31G(d)+SDD(Fe):AmberFF14SB level, with the same MM parameters used in MD simulations. An Electrostatic Embedding was used (see computational details). Doublet (d) and quartet (q) electronic states were considered, and all energies are referred considering LA1-**1**^{q} structure as zero.

B) QM/MM calculated Gibbs free energy profile. Relative Gibbs free energies (ΔG) and electronic energies (ΔE, in parenthesis) are reported.

C) QM/MM optimized structures. Atoms included in the QM region are shown in ball-and-stick representation, and residues in the MM region are shown in sticks. Mutated residues are highlighted in orange. Mulliken charges (*q*) and spin density (ρ) values for the phenyl group (Ph, sum of all C and H atoms), C2 benzylic position, C1 and O, are reported.

Energies, distances, and Mulliken charges and spin density values are given in kcal·mol^{-1}, Angstrom (Å), and a.u., respectively.

A)

Structure	Electronic State	ΔE_{QM}	ΔE	ΔH	ΔG
LA1-**1**	doublet (d)	-0.1	-0.1	-0.2	0.1
	quartet (q)	0.0	0.0	0.0	0.0
LA1-**TS1**	doublet (d)	11.2	16.3	14.8	16.1
	quartet (q)	13.6	15.8	14.7	15.8
LA1-**Int1**	doublet (d)	-11.0	-5.6	-5.8	-3.9
	quartet (q)	-9.6	-7.2	-7.0	-6.0
LA1-**TS2**	quartet (q)	-10.8	-5.3	-5.5	-4.0
LA1-**2-R**	doublet (d)	-33.1	-26.6	-25.0	-23.9
	quartet (q)	-41.5	-33.4	-31.9	-32.3
LA1-**Int2**	doublet (d)	-20.0	-9.9	-10.4	-7.3
	quartet (q)	-24.5	-7.7	-8.8	-8.2
LA1-**TS3-cis-si**	doublet (d)	-19.6	-9.6	-11.2	-8.9
	quartet (q)	-24.0	-7.6	-9.5	-8.3
LA1-**TS3-trans-re**	doublet (d)	-18.2	-9.3	-10.8	-9.0
	quartet (q)	-23.2	-7.2	-8.9	-7.8
LA1-**3**	doublet (d)	-64.4	-56.0	-55.0	-54.1
	quartet (q)	-72.6	-57.9	-57.1	-57.5

B)

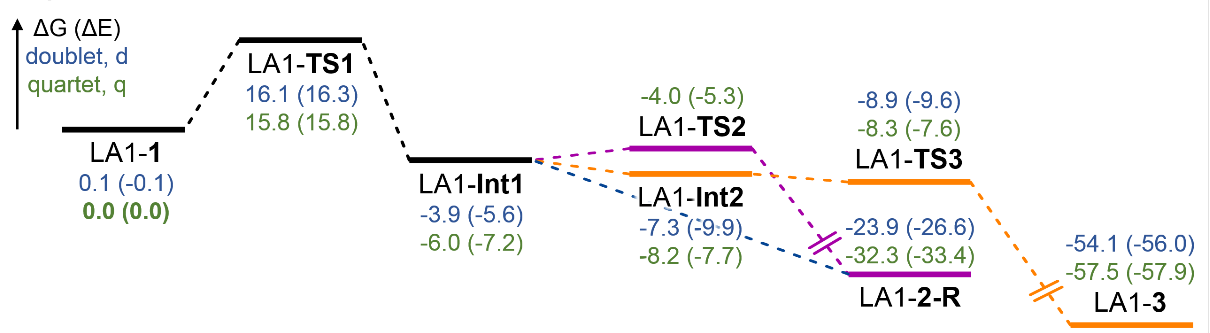

ΔG (ΔE)
doublet, d
quartet, q
LA1-1
0.1 (-0.1)
0.0 (0.0)
LA1-TS1
16.1 (16.3)
15.8 (15.8)
LA1-Int1
-3.9 (-5.6)
-6.0 (-7.2)
-4.0 (-5.3)
LA1-TS2
LA1-Int2
-7.3 (-9.9)
-8.2 (-7.7)
-8.9 (-9.6)
-8.3 (-7.6)
LA1-TS3
-23.9 (-26.6)
-32.3 (-33.4)
LA1-2-R
-54.1 (-56.0)
-57.5 (-57.9)
LA1-3

C)

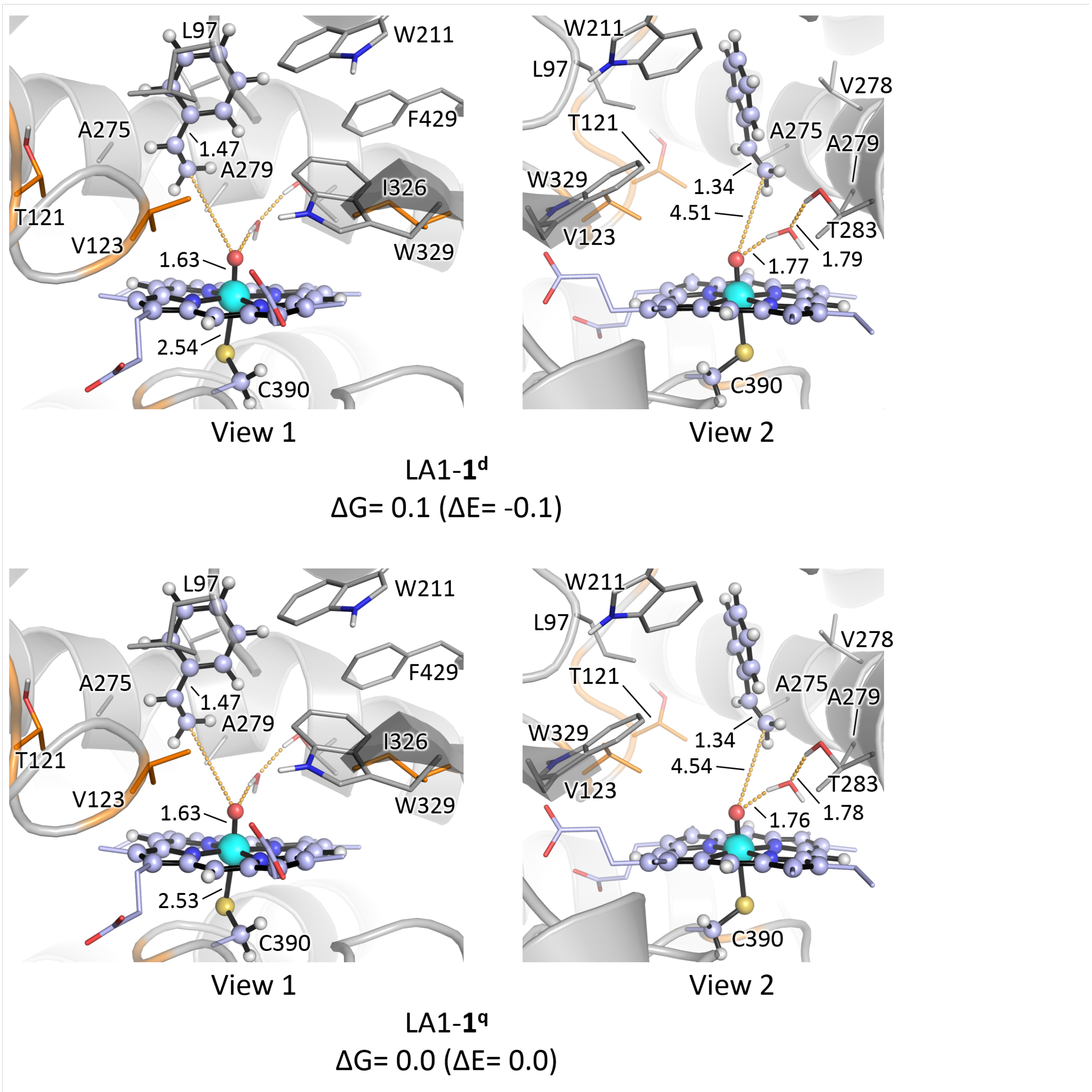

L97
W211
F429
A275
1.47
A279
I326
T121
V123
W329
1.63
2.54
C390
View 1
W211
L97
V278
T121
A275
A279
W329
1.34
4.51
V123
T283
1.77
1.79
C390
View 2
LA1-1ᵈ
ΔG= 0.1 (ΔE= -0.1)
L97
W211
F429
A275
1.47
A279
I326
T121
V123
W329
1.63
2.53
C390
View 1
W211
L97
V278
T121
A275
A279
W329
1.34
4.54
V123
T283
1.76
1.78
C390
View 2
LA1-1ᑫ
ΔG= 0.0 (ΔE= 0.0)

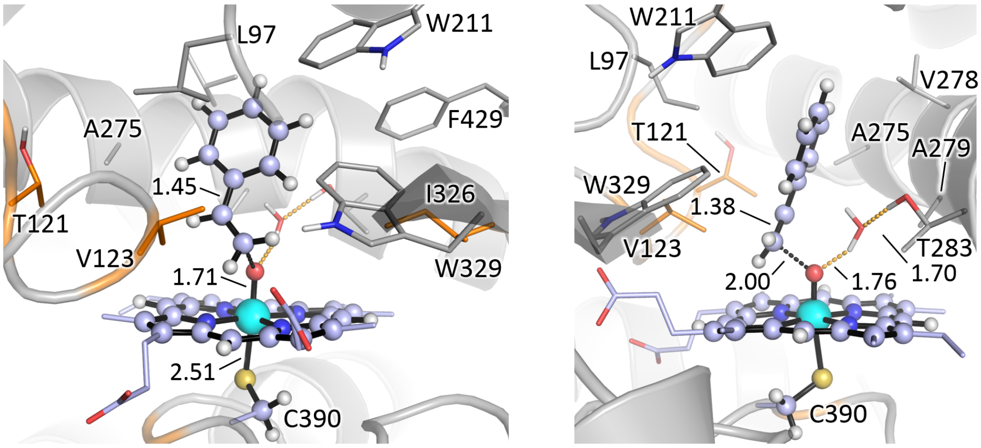

View 1 View 2

q(Ph) = 0.06
q(C2) = -0.03
q(C1) = -0.14
q(O) = -0.55

ρ(Ph) = -0.14
ρ(C2) = -0.35
ρ(C1) = 0.02
ρ(O) = 0.14

LA1-**TS1**[d]
$\Delta G^{\ddagger}$= 16.0 ($\Delta E^{\ddagger}$= 16.4)

|∠Fe-O-C1-C2| = *133.3°*

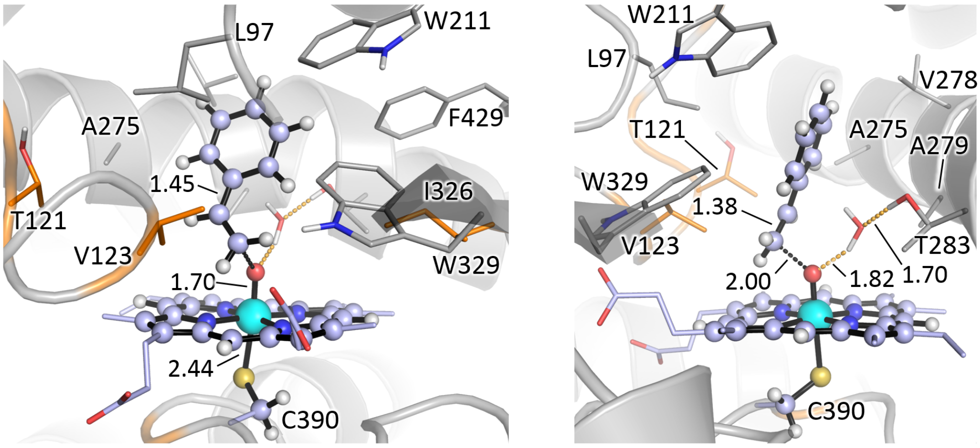

View 1 View 2

q(Ph) = 0.02
q(C2) = -0.07
q(C1) = -0.13
q(O) = -0.52

ρ(Ph) = 0.13
ρ(C2) = 0.35
ρ(C1) = -0.05
ρ(O) = 0.64

LA1-**TS1**[q]
$\Delta G^{\ddagger}$= 15.8 ($\Delta E^{\ddagger}$= 15.8)

|∠Fe-O-C1-C2| = 131.7°

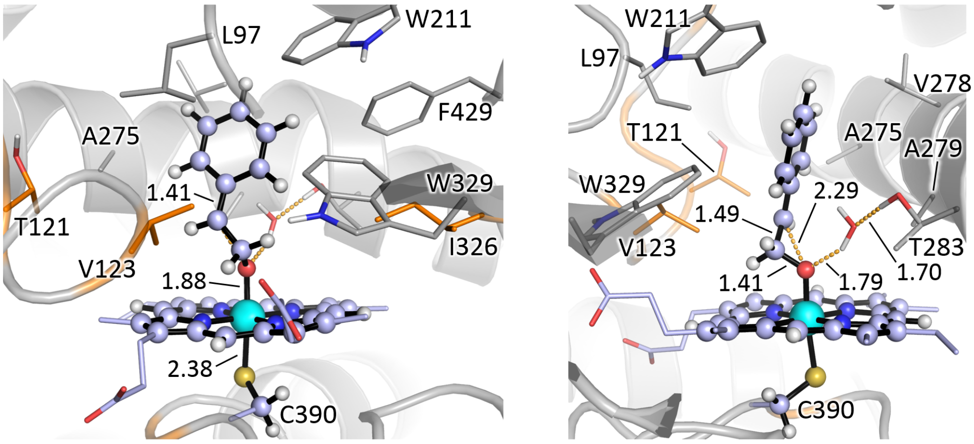

View 1 View 2

q(Ph) = 0.02
q(C2) = 0.00
q(C1) = -0.03
q(O) = -0.54

ρ(Ph) = 0.21
ρ(C2) = 0.50
ρ(C1) = -0.01
ρ(O) = 0.01

LA1-**Int1**[d]
ΔG_r= -4.0 (ΔE_r= -5.5)
$\Delta\Delta G$= 2.1 ($\Delta\Delta E$= 1.6)

∠O-C1-C2-C(Ph) = -107.0°
∠H-C1-C2-C(Ph) = 13.7°

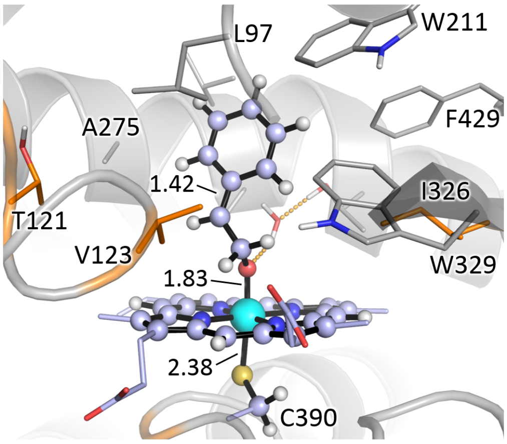

View 1

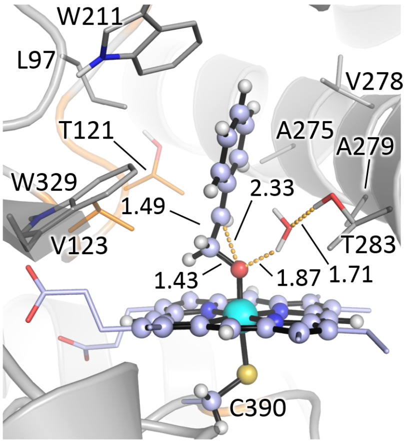

View 2

q(Ph) = -0.03
q(C2) = -0.07
q(C1) = -0.03
q(O) = -0.48

ρ(Ph) = 0.28
ρ(C2) = 0.65
ρ(C1) = 0.00
ρ(O) = 0.15

LA1-**Int1**q
ΔG_r= -6.0 (ΔE_r= -7.2)
$\Delta\Delta G$= 0.0 ($\Delta\Delta E$= 0.0)

∠O-C1-C2-C(Ph) = -108.4°
∠H-C1-C2-C(Ph) = 11.5°

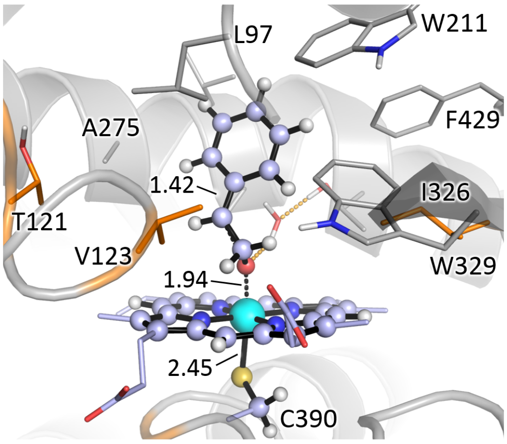

View 1

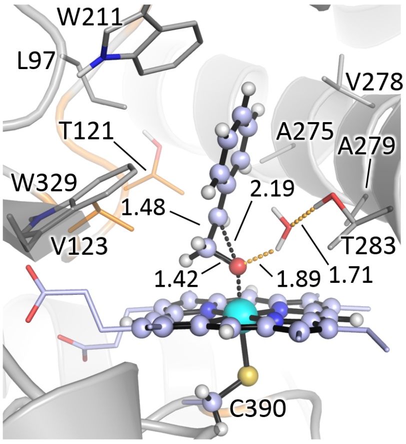

View 2

q(Ph) = 0.02
q(C2) = 0.00
q(C1) = -0.04
q(O) = -0.49

ρ(Ph) = 0.21
ρ(C2) = 0.49
ρ(C1) = 0.00
ρ(O) = -0.05

LA1-**TS2**q
$\Delta G^‡$= 2.0 ($\Delta E^‡$= 1.9)

∠O-C1-C2-C(Ph) = -104.6°

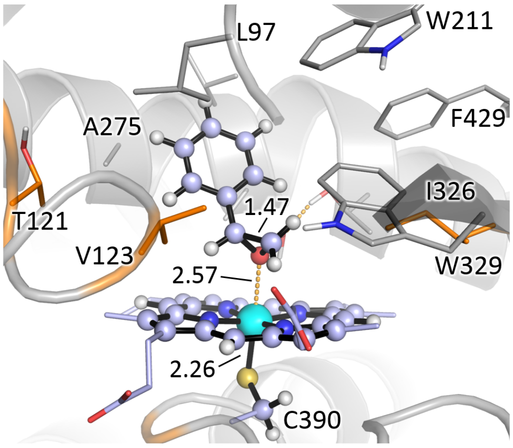

View 1

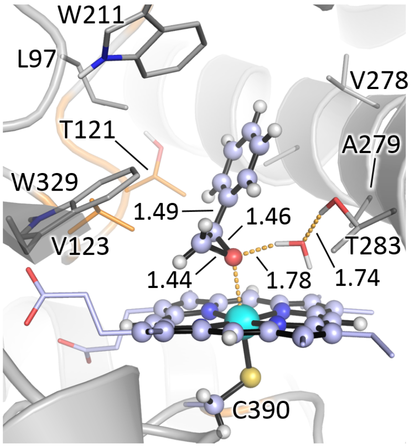

View 2

LA1-**2-R**d
ΔG_r= -20.0 (ΔE_r= -21.0)

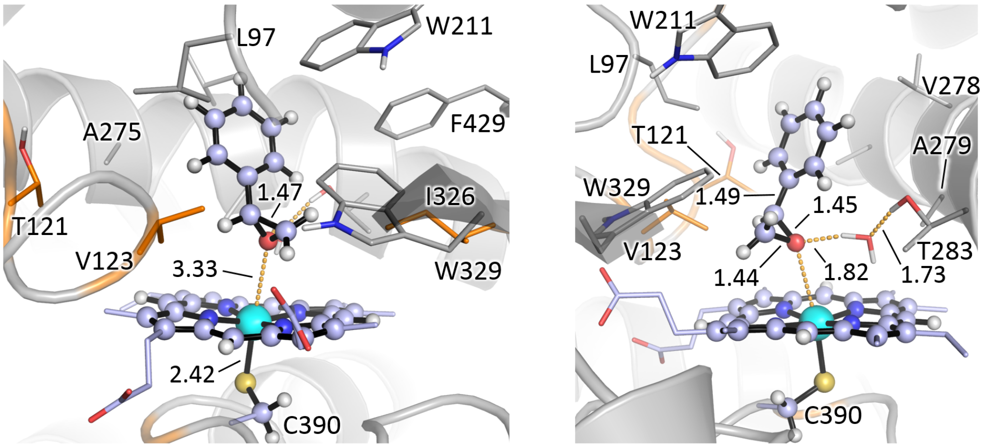

View 1 View 2

LA1-**2-R**[q]

ΔG_r= -26.3 (ΔE_r= -26.2)

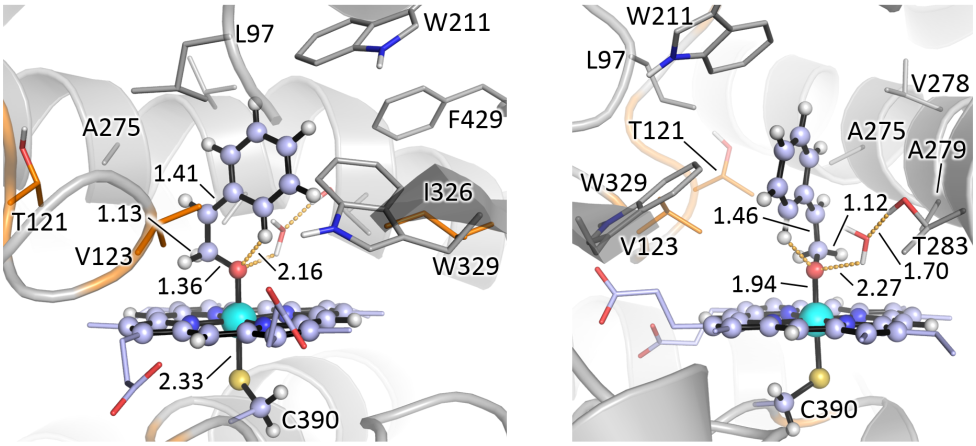

q(Ph) = 0.20
q(C2) = 0.00
q(C1) = 0.04
q(O) = -0.55

ρ(Ph) = 0.09
ρ(C2) = 0.21
ρ(C1) = -0.01
ρ(O) = -0.02

View 1 View 2

LA1-**Int2**[d]

ΔΔG= -3.4 (ΔΔE= -4.3)

∠O-C1-C2-C(Ph) = -11.0º
∠H-C1-C2-C(Ph) = 114.6º

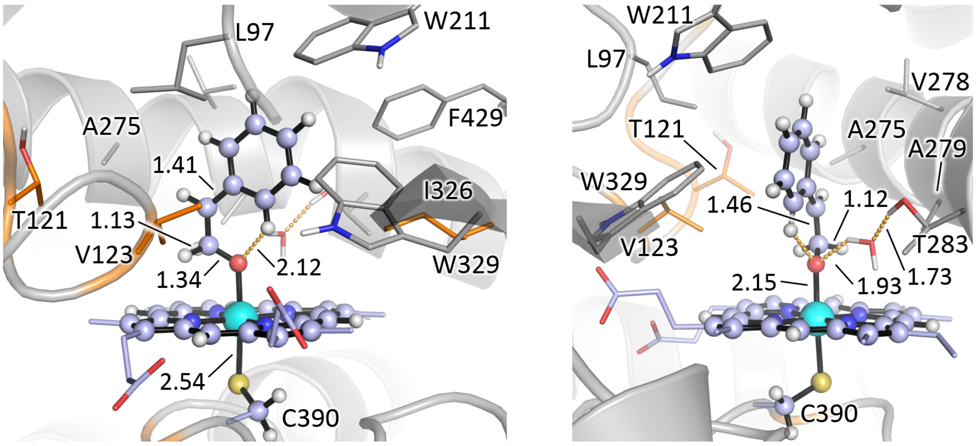

q(Ph) = 0.33
q(C2) = -0.03
q(C1) = 0.06
q(O) = -0.65

ρ(Ph) = -0.01
ρ(C2) = -0.01
ρ(C1) = 0.01
ρ(O) = 0.08

View 1 View 2

LA1-**Int-2**[q]

ΔΔG= -2.2 (ΔΔE= -0.5)

∠O-C1-C2-C(Ph) = -7.5º
∠H-C1-C2-C(Ph) = 120.0º

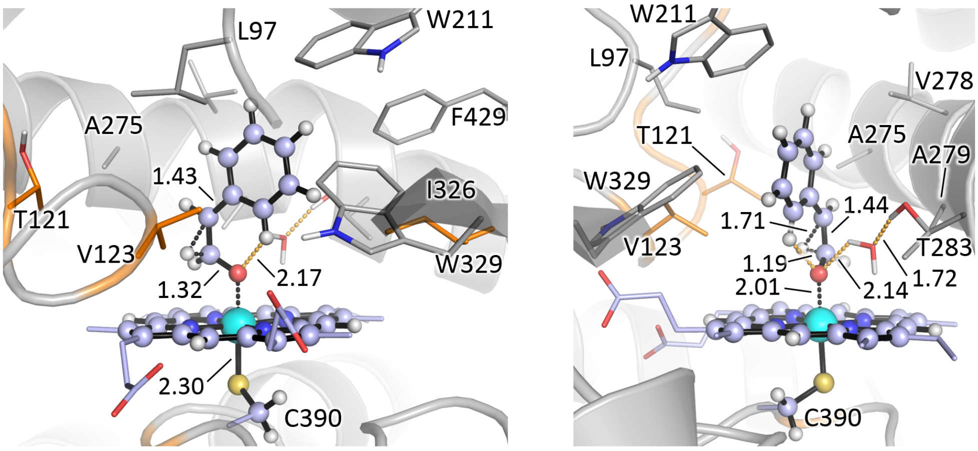

View 1 View 2

q(Ph) = 0.31
q(C2) = -0.08
q(C1) = 0.06
q(O) = -0.54

ρ(Ph) = 0.00
ρ(C2) = 0.01
ρ(C1) = 0.00
ρ(O) = -0.01

LA1-**TS3-cis-si**^{d}
$\Delta G^{\ddagger}$= -1.6 ($\Delta E^{\ddagger}$= 0.4)

∠H-C1-C2-C(Ph) = 102.5°

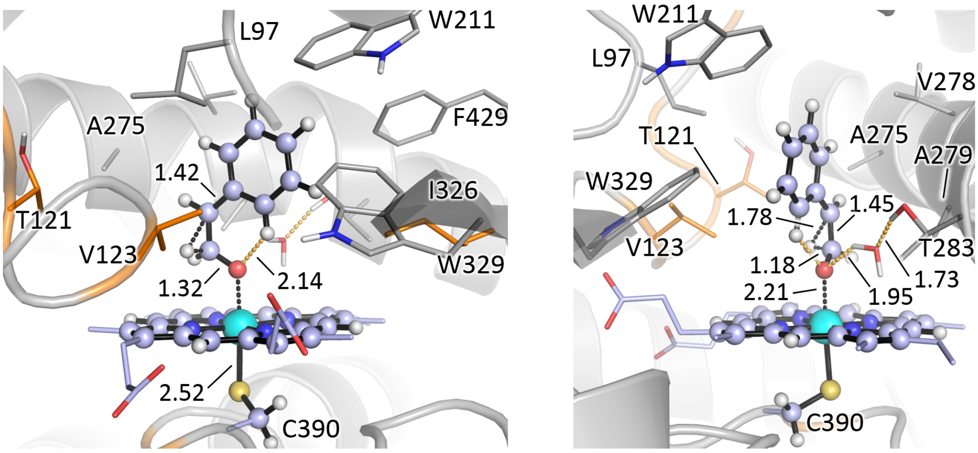

View 1 View 2

q(Ph) = 0.32
q(C2) = -0.07
q(C1) = 0.06
q(O) = -0.63

ρ(Ph) = 0.00
ρ(C2) = 0.01
ρ(C1) = 0.00
ρ(O) = 0.05

LA1-**TS3-cis-si**^{q}
$\Delta G^{\ddagger}$= -0.1 ($\Delta E^{\ddagger}$= 0.1)

∠H-C1-C2-C(Ph) = 104.9°

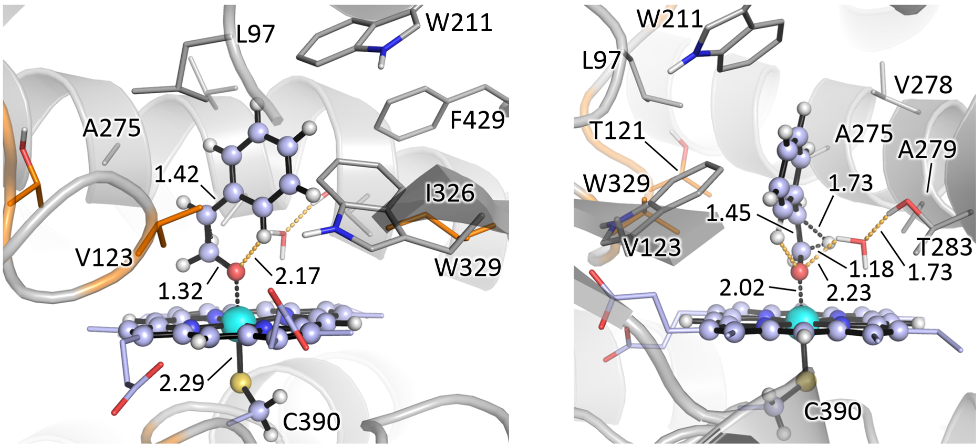

View 1 View 2

q(Ph) = 0.27
q(C2) = -0.01
q(C1) = 0.00
q(O) = -0.52

ρ(Ph) = 0.00
ρ(C2) = 0.01
ρ(C1) = 0.00
ρ(O) = 0.00

LA1-**TS3-trans-re**^{d}
$\Delta G^{\ddagger}$= -1.7 ($\Delta E^{\ddagger}$= 0.6)

∠H-C1-C2-C(Ph) = -104.4°

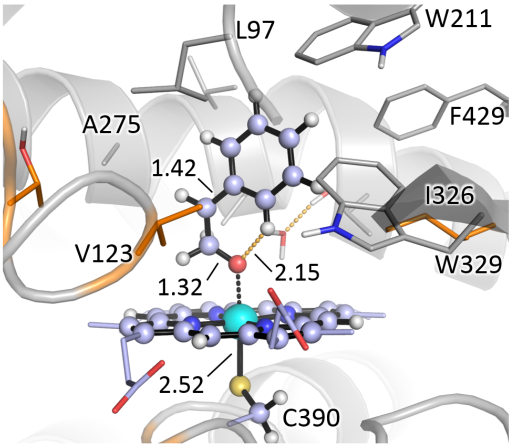

View 1

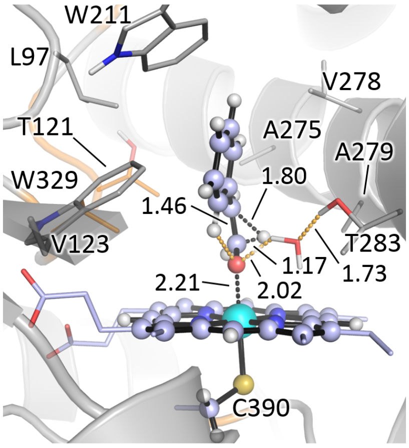

View 2

q(Ph) = 0.29
q(C2) = -0.03
q(C1) = 0.04
q(O) = -0.61

ρ(Ph) = 0.00
ρ(C2) = 0.00
ρ(C1) = 0.01
ρ(O) = 0.05

LA1-**TS3-trans-re**[q]
ΔG‡= 0.4 (ΔE‡= 0.5)

∠H-C1-C2-C(Ph) = -108.9º

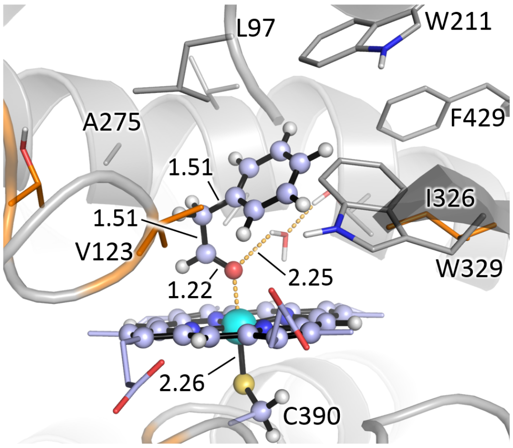

View 1

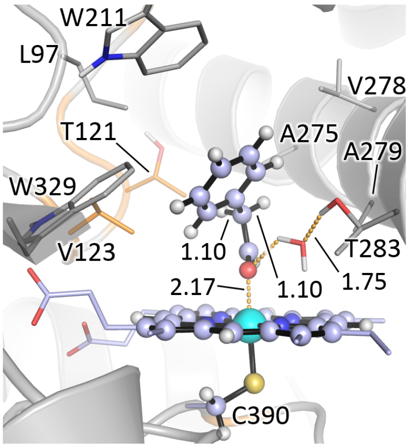

View 2

LA1-**3**[d]
ΔG_r= -46.8 (ΔE_r= -46.1)

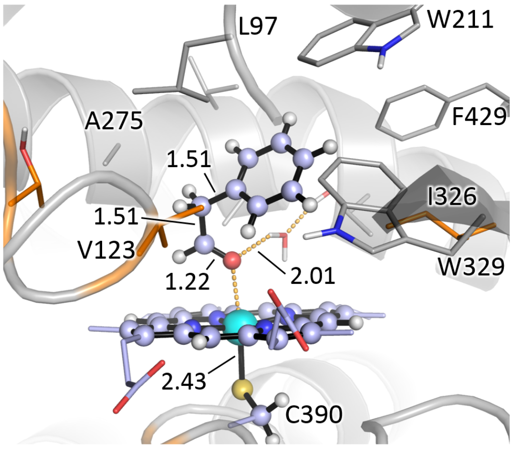

View 1

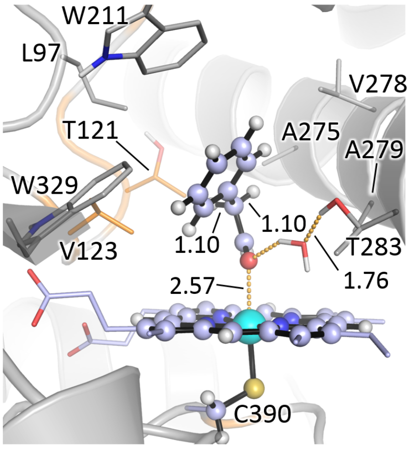

View 2

LA1-**3**[q]
ΔG_r= -49.3 (ΔE_r= -50.2)

The particular substrate bound conformation in a catalytically relevant pose explored here, exposing the *si* face to the iron-oxo species, corresponds to the major NAC binding pose characterized from MD simulations (see **Figure S10**). This particular binding mode leads to the preferential formation of the *R*-epoxide product.

For the reaction occurring in $P450_{LA1}$ active site, the covalent radical intermediate LA1-**Int1** formed from a *si* face NAC in the quartet and doublet electronic states can be optimized as a minimum on the potential energy surface (PES). This is indicating that the enzyme active site can stabilize these reactive intermediates due to confinement, as compared to the free-enzyme model system (see **Figure S1**).

The calculated rate-limiting TS1 is 3.3 kcal·mol^{-1} lower than the equivalent TS1 with the substrate oriented with the less explored binding mode exposing the *re* enantioface to the iron-oxo species, as described in the previous **Figure S14**.

Epoxidation pathway and carbonyl formation pathway are both energetically accessible and both pathways can compete, similar to enzyme-free system (see **Figure S1**). Epoxidation is slightly favored in the doublet state, being barrierless from LA1-**Int1^{d}** (LA1-**TS2^{d}** could not be optimized) although carbocation LA1-**Int2^{d}** leading to the carbonyl pathway can also be formed after a required conformational change that involves the rotation of the methylene-oxygen group (see **Figure S18**). Carbonyl formation is slightly favored in the quartet state after the required conformational change of LA1-**Int1^{q}** to stabilize LA1-**Int2^{q}** due to stereoelectronic effects (see **Figure S15-B** and **Figure S16**).

QM/MM calculations for the aldehyde formation pathway describe that the carbocation intermediate (LA1-**Int2**) is slightly more stabilized than the radical intermediate (LA1-**Int1**) for both electronic states, and that 1,2-hydride migration from LA1-**Int2** is barrierless.

Figure S16: QM/MM optimized structures of key intermediates formed in P450$_{LA1}$ WT active site: **A**) covalent radical intermediate (LA1-**Int1^{q}**); and **B**) covalent carbocation intermediate (LA1-**Int2^{q}**). Lowest in energy electronic states are considered (see **Figure S15**).

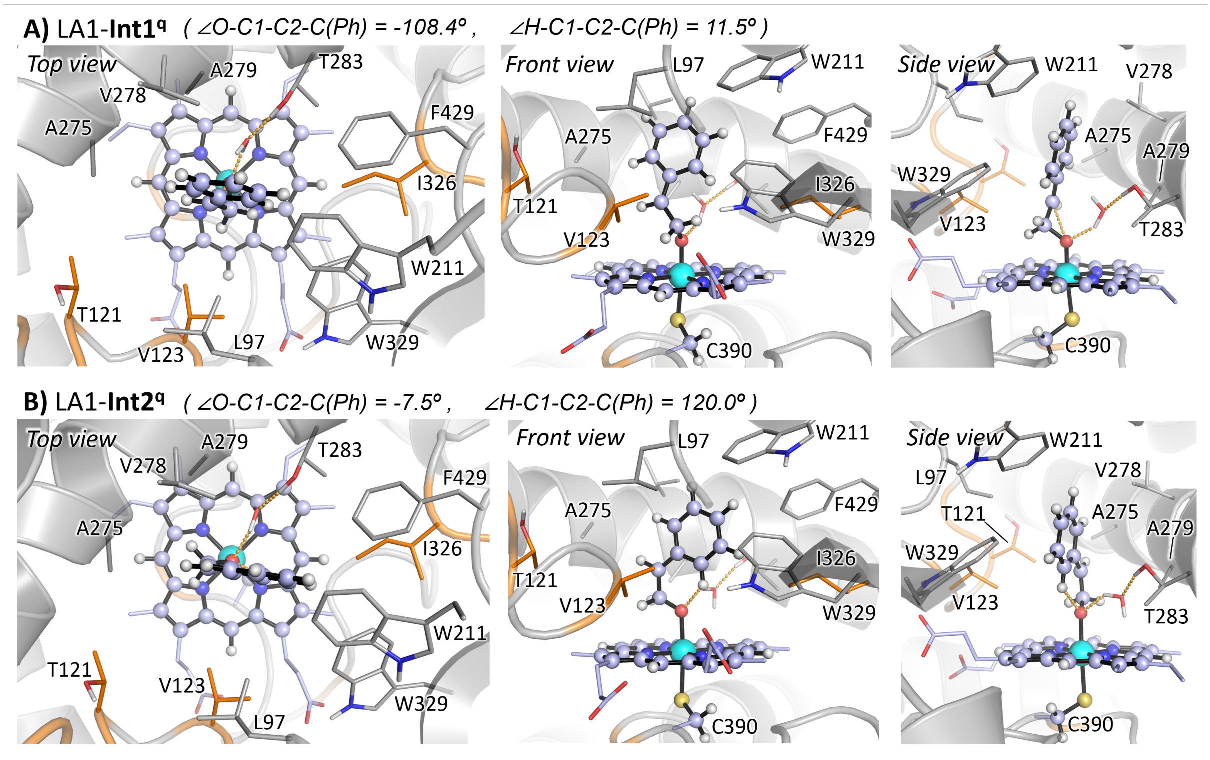

Figure S17: Active site packing occurring in P450$_{LA1}$ QM/MM optimized key transition states: **A)** LA1-**TS2** and **B)** LA1-**TS3**. Only lowest in energy electronic states are considered here (see **Figure S15**). For each QM/MM optimized structure two different orientations (side and top view) are shown, using two different representation models with and without space-filling model for key active site residues (L97, W211, V278, A279, W329, and F429).

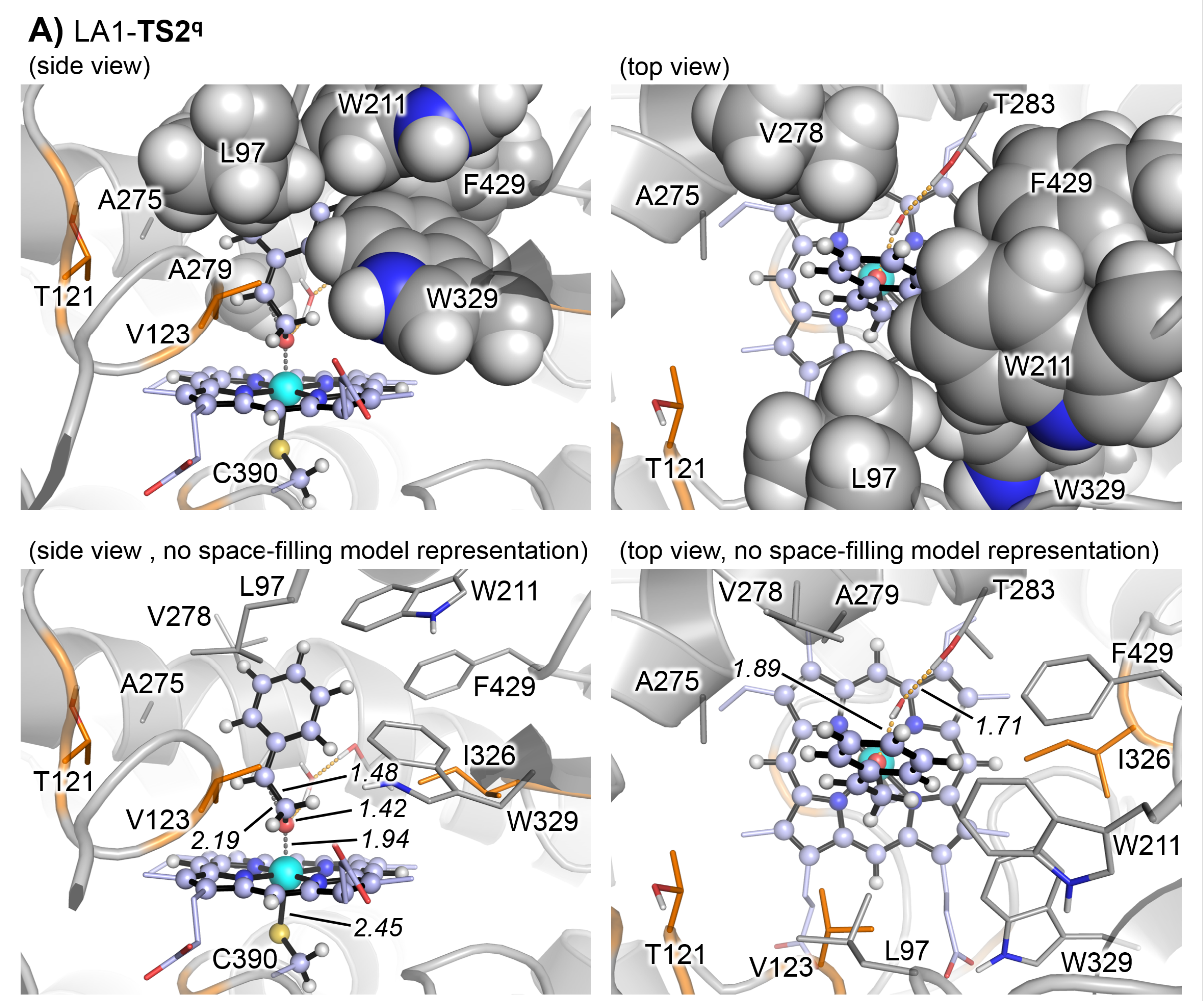

B) LA1-**TS3**[d]

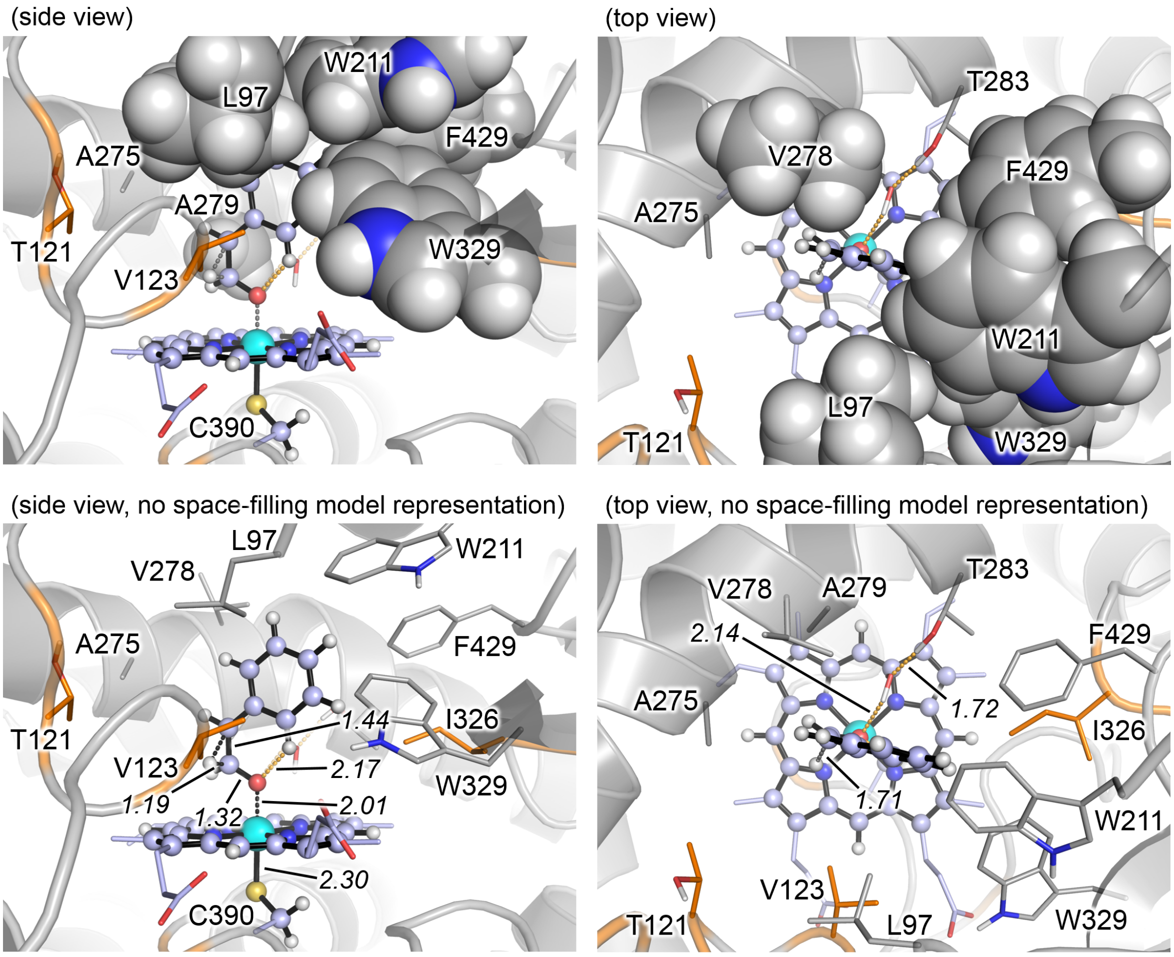

Active site packing in P450$_{LA1}$ impose steric restrictions that directly impact on the transition state structures. Active site residues V278, F429, W211, and W329 force LA1-**TS2**[q] structure to slightly modify its ∠(Fe-O-C1-C2) dihedral angle as compared to the free-enzyme model **TS2** (147.5° vs 179.1°, respectively). However, these interactions do not have a large influence on the energy barrier (LA1-**TS2**[q], $\Delta G^{\ddagger}$=2.0 kcal·mol^{-1}) and do not prevent the formation of the *R*-epoxide product since the energy barrier is still very low. In LA1-**TS3**, the phenyl ring of styrene is also stacked between V278, W329, W211, and F429 residues.

Figure S18: QM/MM calculations were used to study the rotation of the C1–C2 bond in the quartet covalent radical intermediate formed in the $P450_{LA1}$ WT system (LA1-**Int1^q**, **Figure S15**).

A) QM/MM relaxed scan calculations were carried out along the rotation of ∠(O-C1-C2-C(Ph)) dihedral angle starting from the optimized radical intermediate species (LA1-**Int1^q**, ∠(O-C1-C2-C(Ph) ca. -108°). Calculations were carried out using the mechanical embedding (ME) approach and at the (U)B3LYP/6-31G(d)+SDD(Fe):Amber FF14SB level.

B) Single point calculations were performed on the optimized points along the relaxed scan coordinate reported in **A**), but using an electrostatic embedding (EE) scheme and at (U)B3LYP/Def2TZVP:Amber FF14SB level.

C) Geometries of key points on the scan coordinate reported in **A**).

Energies are referred considering the optimized LA1-**Int1^q** as zero. Energies and dihedrals are given in kcal·mol^{-1} and degrees (°), respectively.

A)

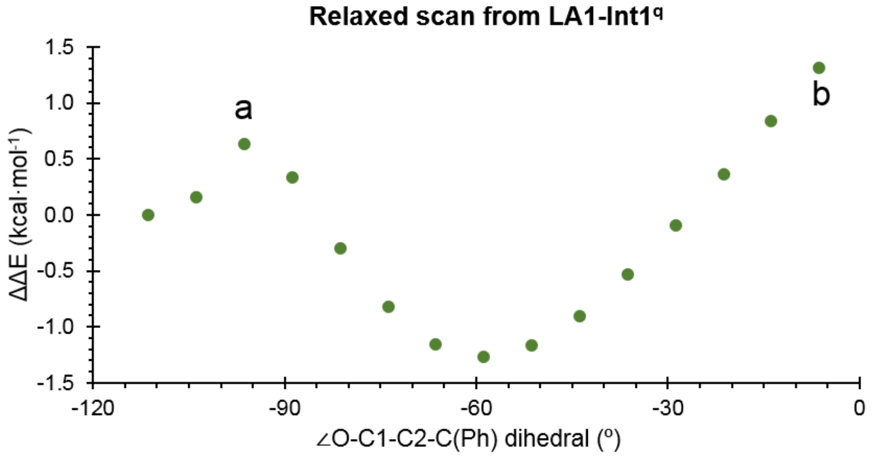

B)

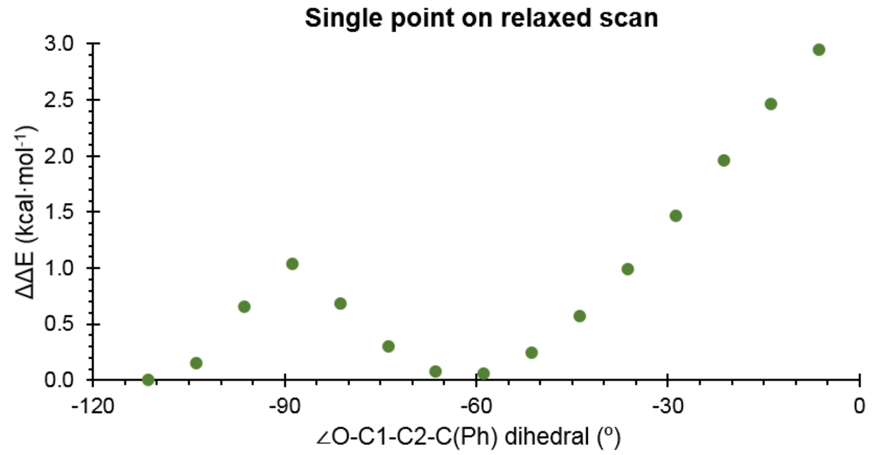

C)

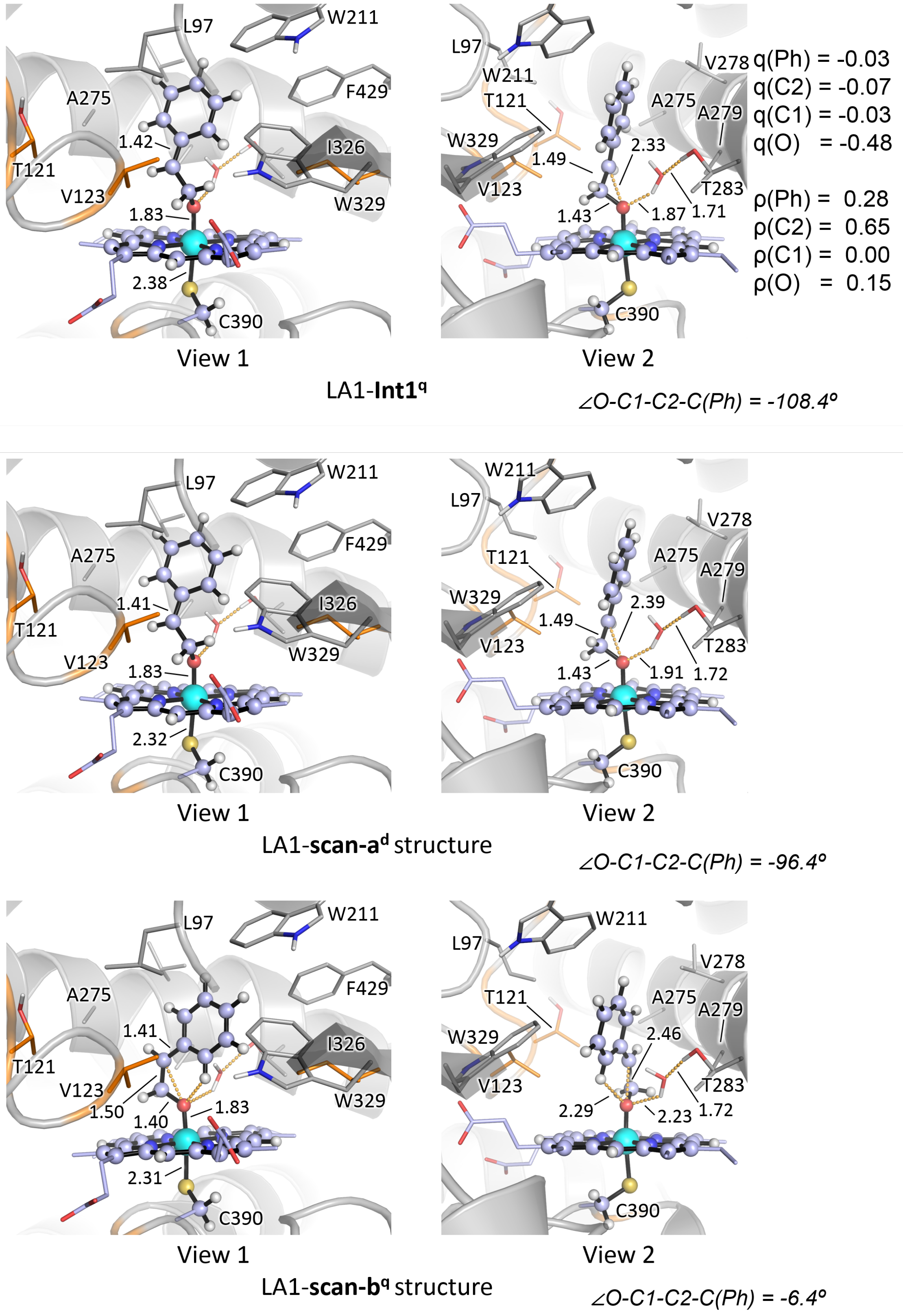

QM/MM relaxed scan calculations indicated that rotation along the O-C1-C2-C(Ph) dihedral from **LA1-Int1** to **LA1-Int2**-like geometry requires a low energy ΔE = ca. +1.5 kcal·mol^{-1} (ΔE = ca. 3.0 kcal·mol^{-1} from Single Point calculations at higher level) on the lower in energy quartet

electronic state. This electronic energy value is similar to the electronic energy barrier calculated for the formation of the epoxide product (LA1-**TS2^q** $\Delta E^{\ddagger}$ = +1.9 kcal·mol^{-1}, from Single Point calculations at higher level, see **Figure S15**).

All attempts to optimize a rotational transition state starting from the highest in energy point on the relaxed scan coordinate were unsuccessful, suggesting that this conformational change can occur slightly uphill but in a barrierless manner, , and thus LA1-**Int2**-like geometry can be easily accessed from LA1-**Int1^q**. This is similar to what is found for the enzyme-free model (see **Figure S2**).

Figure S19: QM/MM exploration of aMOx variant catalyzed oxidation of styrene (**1**) substrate. QM/MM calculations were carried out starting from a MD-relaxed structure of the covalent intermediate (**Int1**) formed in aMOx active site (**Figure S13-B**), that mimics the major explored (preferred), catalytically relevant, binding pose of styrene substrate characterized from restrained-MD simulations (red and pink marker selected snapshots in **Figure S11)**. This corresponds to a styrene binding pose with its *re* face exposed to the iron-oxo species (pro-*S* epoxidation).

A) QM/MM computed relative stabilities in terms of electronic energy at the QM region (ΔE_{QM}), QM/MM ONIOM electronic energy (ΔE), enthalpy (ΔH), and Gibbs energy (ΔG) for the different optimized species. Energy values were obtained at the (U)B3LYP/Def2TZVP: AmberFF14SB//(U)B3LYP/6-31G(d)+SDD(Fe):AmberFF14SB level, with the same MM parameters used in MD simulations. An Electrostatic Embedding was used (see computational details). Doublet (d) and quartet (q) electronic states were considered, and all energies are referred considering the lowest in energy aMOx-**1**$^{\mathbf{d}}$ structure as zero.

B) QM/MM calculated Gibbs free energy profile. Relative Gibbs free energies (ΔG) and electronic energies (ΔE, in parenthesis) are reported.

C) QM/MM optimized structures. Atoms included in the QM region are shown in ball-and-stick representation, and residues in the MM region are shown in sticks. Mutated residues are highlighted in orange. Mulliken charges (*q*) and spin density (ρ) values for the phenyl group (Ph, sum of all C and H atoms), C2 benzylic position, C1 and O, are reported.

D) Intrinsic Reaction Coordinate calculations (IRC) starting from aMOx-**TS1**$^{\mathbf{d}}$ and aMOx-**TS1**$^{\mathbf{q}}$.

E) Reverse Intrinsic Reaction Coordinate calculation (IRC) starting from aMOx-**TS3-trans-si**$^{\mathbf{q}}$

Energies, distances, and Mulliken charges and spin density values are given in kcal·mol^{-1}, Angstrom (Å), and a.u., respectively.

A)

Structure	Electronic State	ΔE_{QM}	ΔE	ΔH	ΔG
aMOx-**1**	doublet (d)	0.0	0.0	0.0	0.0
	quartet (q)	0.2	0.1	0.1	0.0
aMOx-**TS1**	doublet (d)	13.1	7.2	6.4	9.2
	quartet (q)	15.6	8.0	7.2	10.0
aMOx-**Int1**	doublet (d)	-9.5	-15.7	-16.3	-14.1
	quartet (q)	-8.9	-19.4	-19.6	-18.1
aMOx-**TS2 pro-*S***	doublet (d)	-4.7	-12.3	-12.0	-8.4
	quartet (q)	-5.1	-10.1	-10.2	-7.4
aMOx-**TS2 pro-*R***	doublet (d)	-5.2	-5.8	-4.9	0.8
	quartet (q)	-11.9	1.3	1.9	6.6
aMOx-**2-S**	doublet (d)	-31.6	-35.7	-34.0	-31.9
	quartet (q)	-38.9	-38.0	-36.1	-35.2
aMOx-**2-R**	doublet (d)	-24.5	-28.5	-25.9	-22.5
	quartet (q)	-35.1	-37.9	-36.4	-34.7
aMOx-**Int2**	doublet (d)	-18.9	-14.0	-14.9	-12.2
	quartet (q)	-16.8	-8.7	-10.0	-9.0 [a]
aMOx-**TS3-cis-re**	doublet (d)	-18.5	-14.2	-15.9	-12.4
	quartet (q)	-17.7	-10.4	-12.6	-10.4 [b]
aMOx-**TS3-trans-si**	doublet (d)	-17.2	-11.9	-13.4	-10.0
	quartet (q)	-16.4	-8.1	-10.0	-8.1
aMOx-**3**	doublet (d)	-57.9	-56.0	-55.1	-53.7
	quartet (q)	-61.4	-59.6	-58.7	-58.9

[a] aMOx-**Int2^q** was optimized with C2–*cis*-H, C2–*trans*-H, and *cis*-H–*trans*-H distances frozen. Distance values are taken from optimized structure in the doublet state. Frequency calculation showed that the optimized structure has all frequencies positive.

[b] aMOx-**TS3-cis-re^q** was optimized with C2–*cis*-H, C1–*cis*-H and O–C1 distances frozen. Distance values are taken from optimized structure in the doublet state. Frequency calculation showed that all frequencies of the optimized structure are positive except one, which corresponds to the H-migration coordinate.

B)

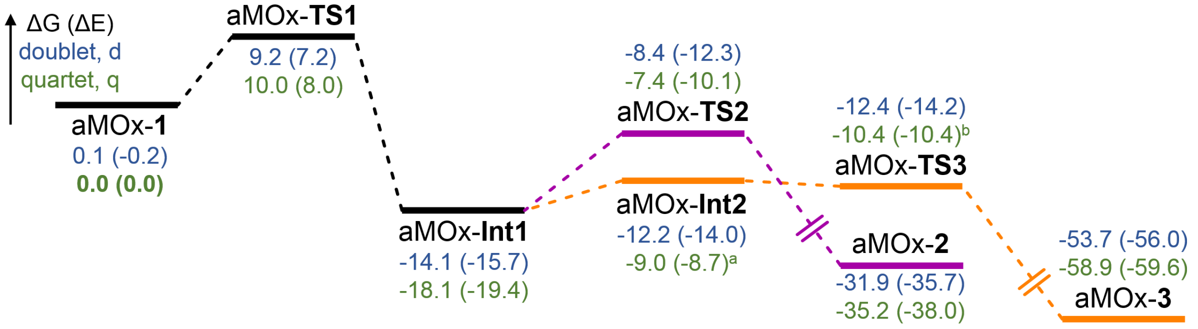

C)

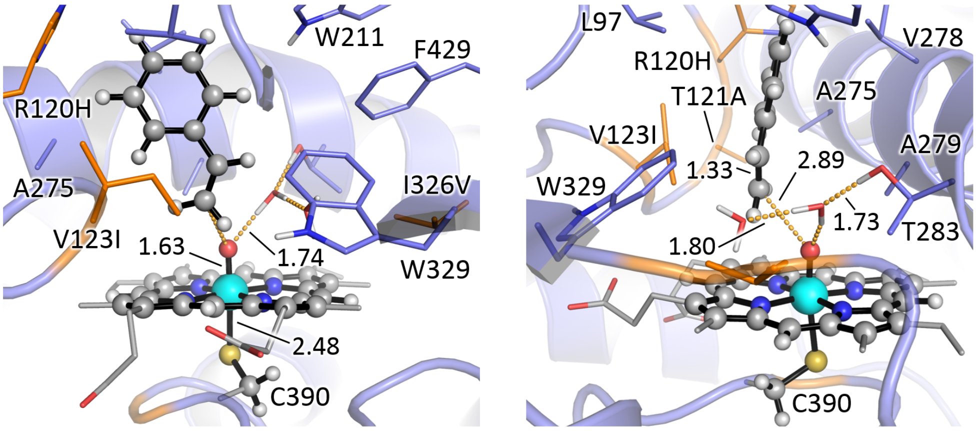

View 1 View 2

aMOx-**1**^{d}

ΔG= 0.0 (ΔE= 0.0)

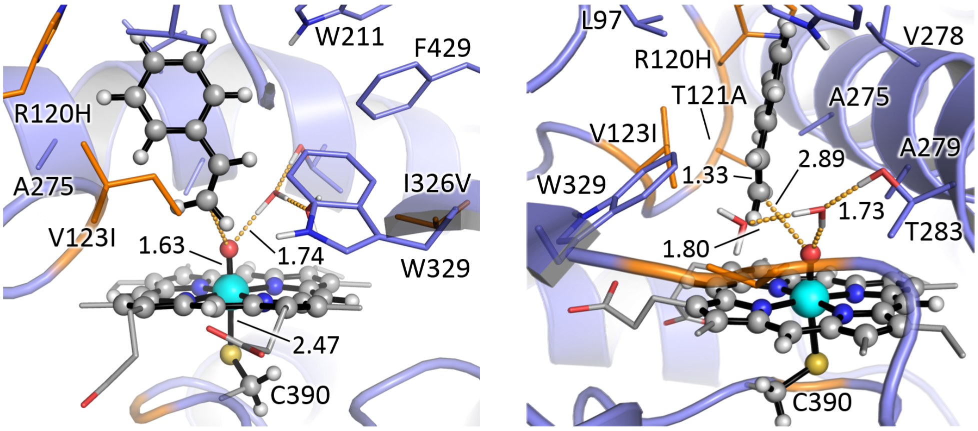

View 1 View 2

aMOx-**1**^{q}

ΔG= 0.0 (ΔE= 0.1)

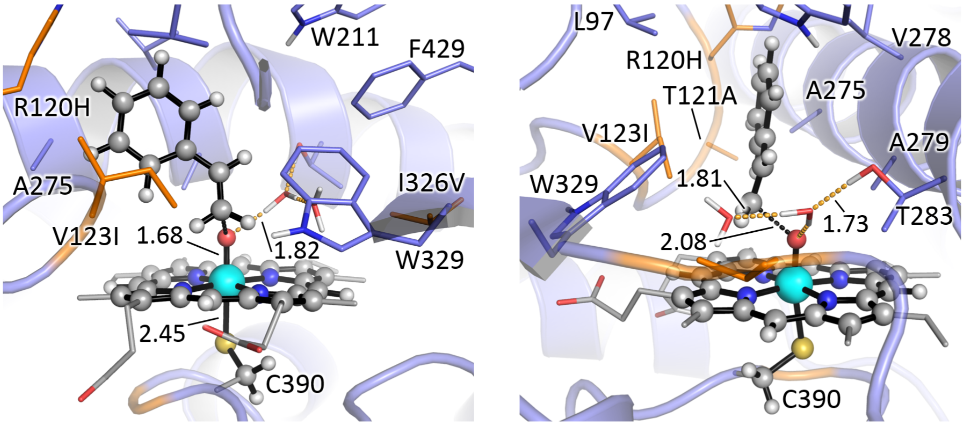

View 1 View 2

q(Ph) = 0.14
q(C2) = -0.06
q(C1) = -0.25
q(O) = -0.52

ρ(Ph) = -0.13
ρ(C2) = -0.27
ρ(C1) = 0.00
ρ(O) = 0.25

aMOx-**TS1**^{d}

ΔG‡= 9.2 (ΔE‡= 7.2)

|∠Fe-O-C1-C2| = 175.2º

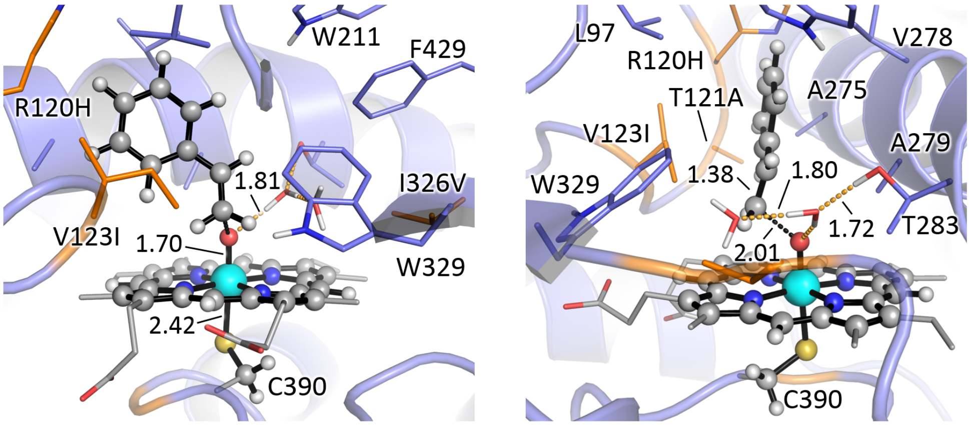

q(Ph) = 0.11
q(C2) = -0.08
q(C1) = -0.22
q(O) = -0.51

ρ(Ph) = 0.15
ρ(C2) = 0.35
ρ(C1) = -0.03
ρ(O) = 0.66

View 1 View 2

aMOx-**TS1[q]**
$\Delta G^{\ddagger}$= 9.9 ($\Delta E^{\ddagger}$= 7.9)

|∠Fe-O-C1-C2| = 174.1°

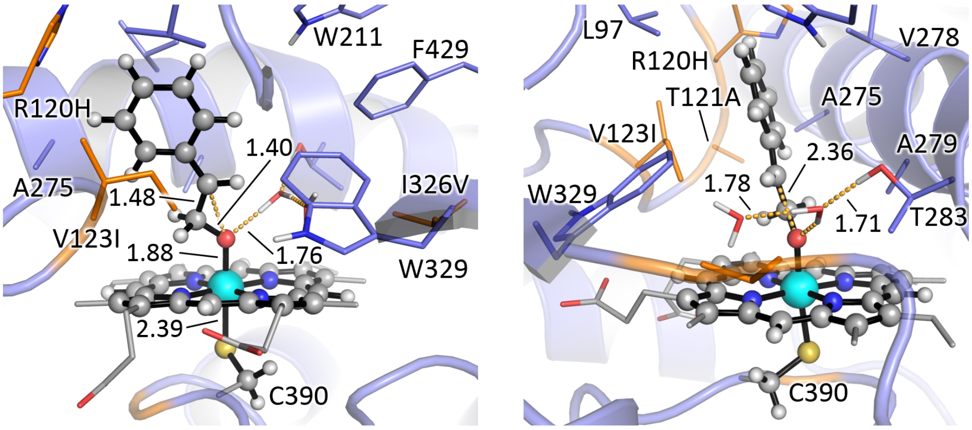

q(Ph) = 0.11
q(C2) = -0.09
q(C1) = 0.02
q(O) = -0.59

ρ(Ph) = -0.19
ρ(C2) = -0.47
ρ(C1) = 0.00
ρ(O) = 0.08

View 1 View 2

aMOx-**Int1[d]**
ΔG_r= -14.1 (ΔE_r= -15.7)
$\Delta\Delta G$= 4.0 ($\Delta\Delta E$= 3.7)

∠O-C1-C2-C(Ph) = 166.1°
∠H-C1-C2-C(Ph) = 40.1°

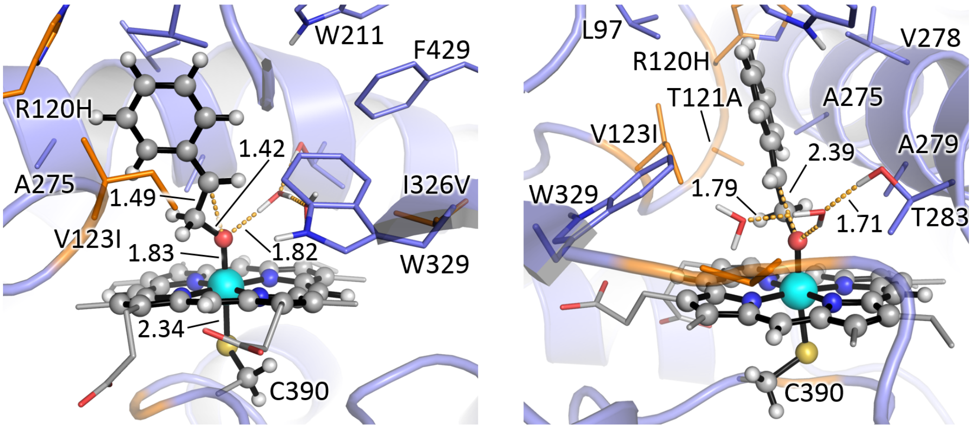

q(Ph) = -0.02
q(C2) = -0.16
q(C1) = 0.01
q(O) = -0.50

ρ(Ph) = 0.29
ρ(C2) = 0.69
ρ(C1) = 0.04
ρ(O) = 0.20

View 1 View 2

aMOx-**Int1[q]**
ΔG_r= -18.1 (ΔE_r= -19.5)
$\Delta\Delta G$= 0.0 ($\Delta\Delta E$= 0.0)

∠O-C1-C2-C(Ph) = 165.7°
∠H-C1-C2-C(Ph) = 43.3°

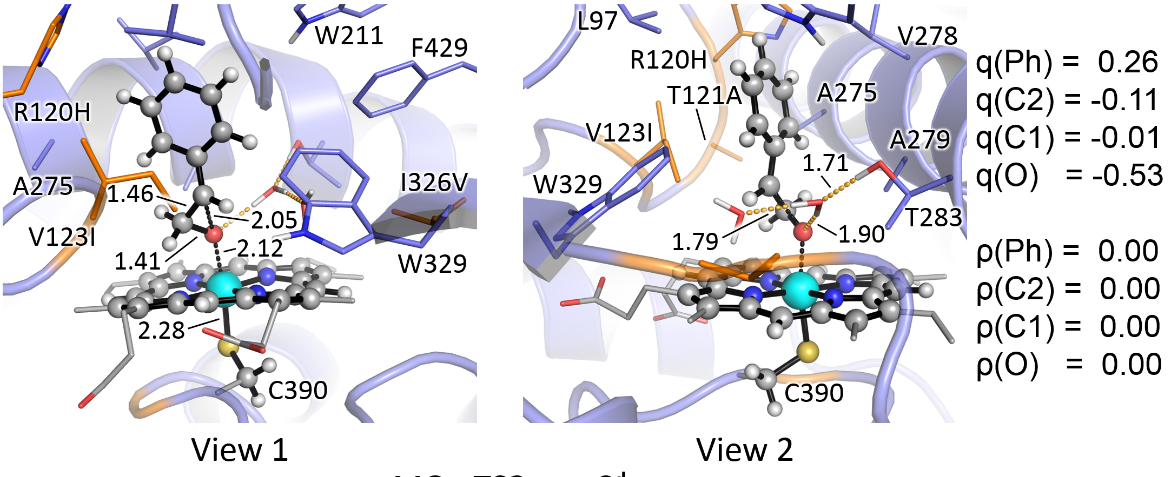

aMOx-**TS2 pro-S**[d]
ΔG‡= 5.7 (ΔE‡= 3.5)

∠O-C1-C2-C(Ph) = 121.9°

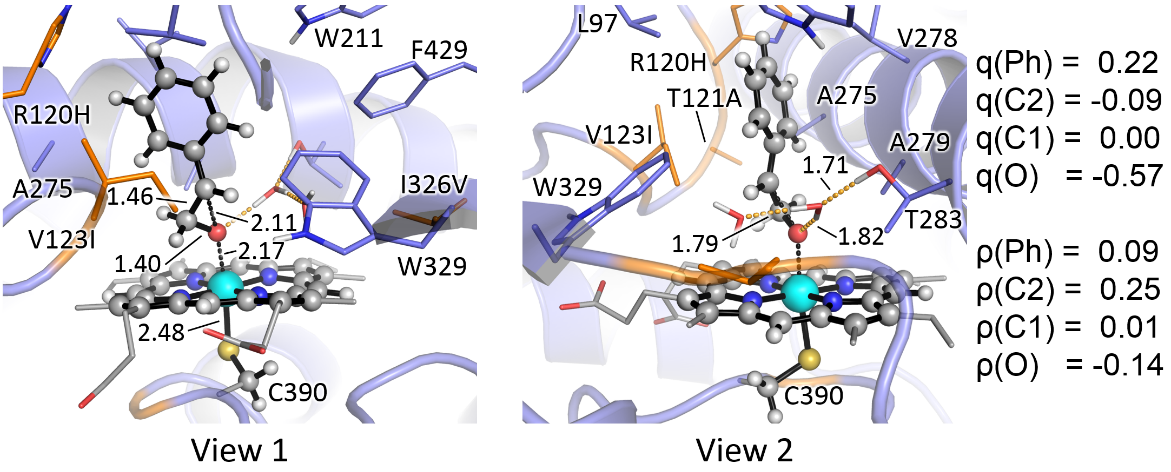

aMOx-**TS2 pro-S**[q]
ΔG‡= 10.7 (ΔE‡= 9.4)

∠O-C1-C2-C(Ph) = 127.2°

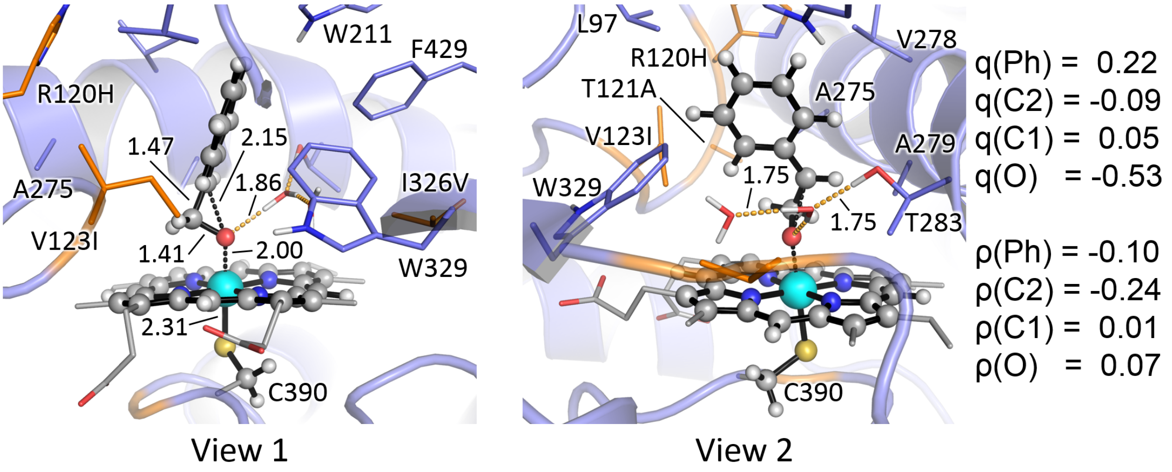

aMOx-**TS2 pro-R**[d]
ΔG‡= 14.9 (ΔE‡= 9.9)

∠O-C1-C2-C(Ph) = -98.1°

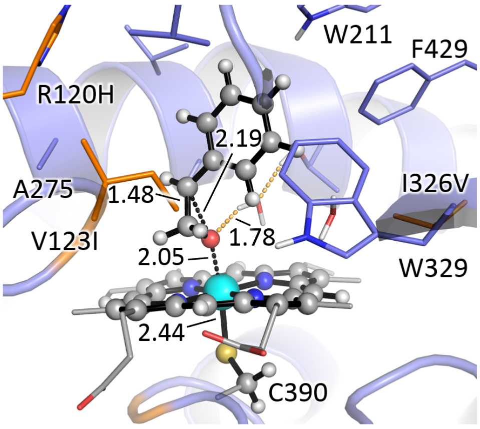

View 1

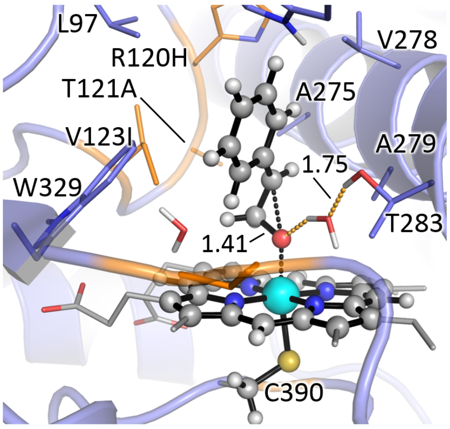

View 2

q(Ph) = 0.18
q(C2) = -0.20
q(C1) = 0.08
q(O) = -0.56

ρ(Ph) = 0.16
ρ(C2) = 0.42
ρ(C1) = 0.00
ρ(O) = -0.12

aMOx-**TS2 pro-R**[q]
$\Delta G^{\ddagger}$= 24.7 ($\Delta E^{\ddagger}$= 20.7)

∠O-C1-C2-C(Ph) = -89.0°

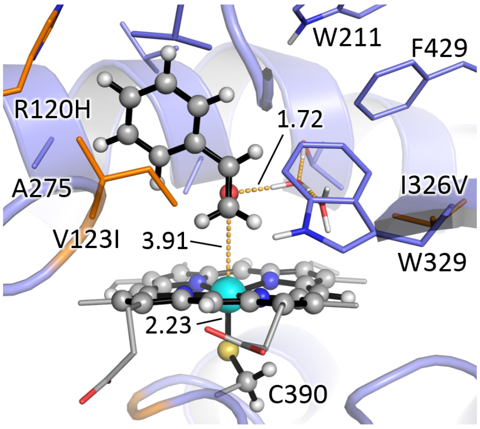

View 1

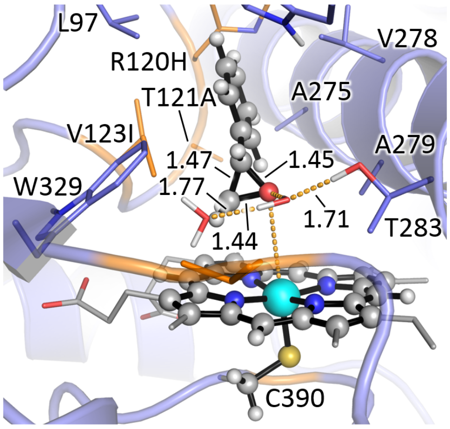

View 2

aMOx-**2-S**[d]
ΔG_r= -17.8 (ΔE_r= -20.0)

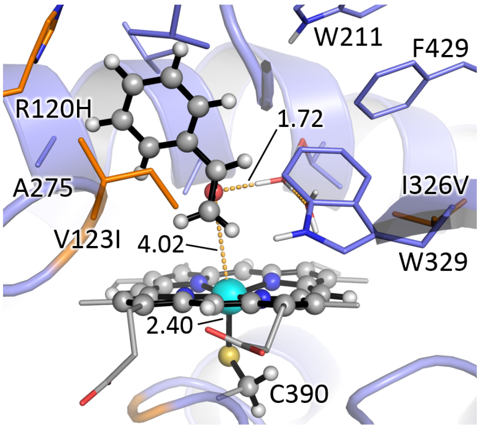

View 1

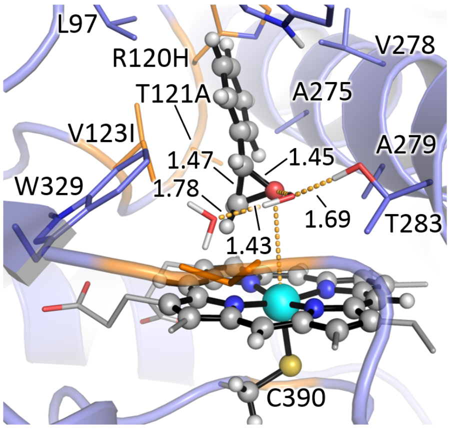

View 2

aMOx-**2-S**[q]
ΔG_r= -17.1 (ΔE_r= -18.6)

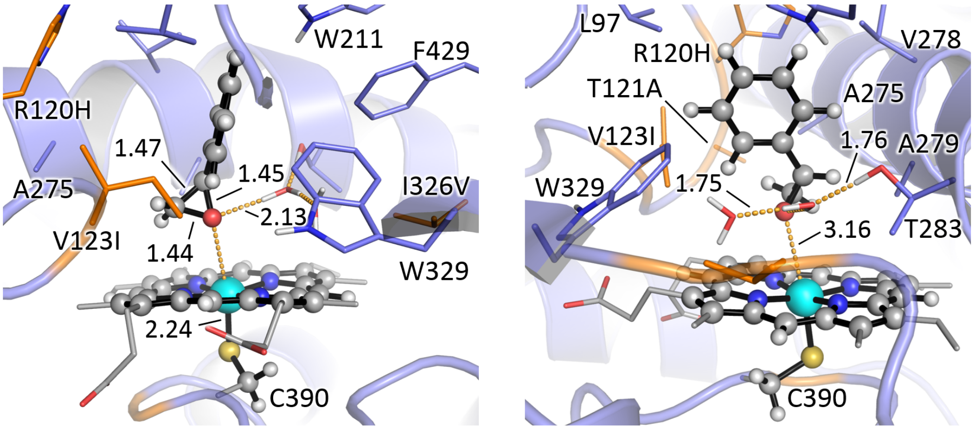

View 1 View 2

aMOx-**2-R**[d]

ΔG_r= -8.4 (ΔE_r= -12.8)

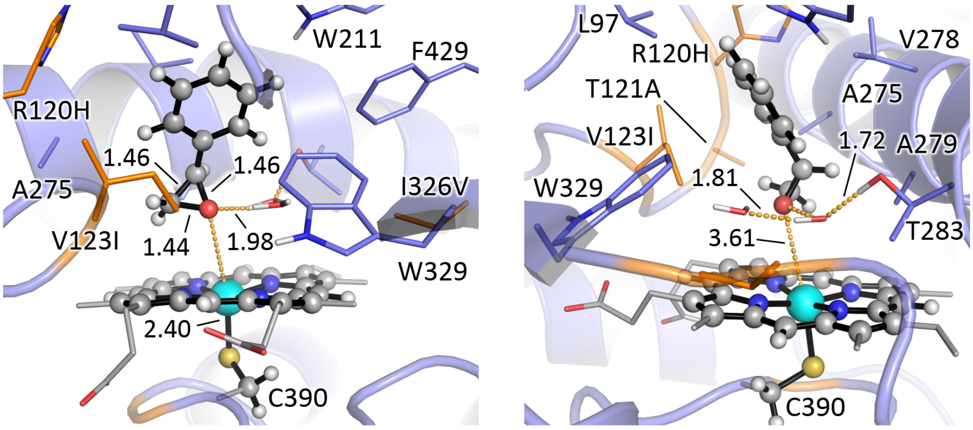

View 1 View 2

aMOx-**2-R**[q]

ΔG_r= -16.6 (ΔE_r= -18.4)

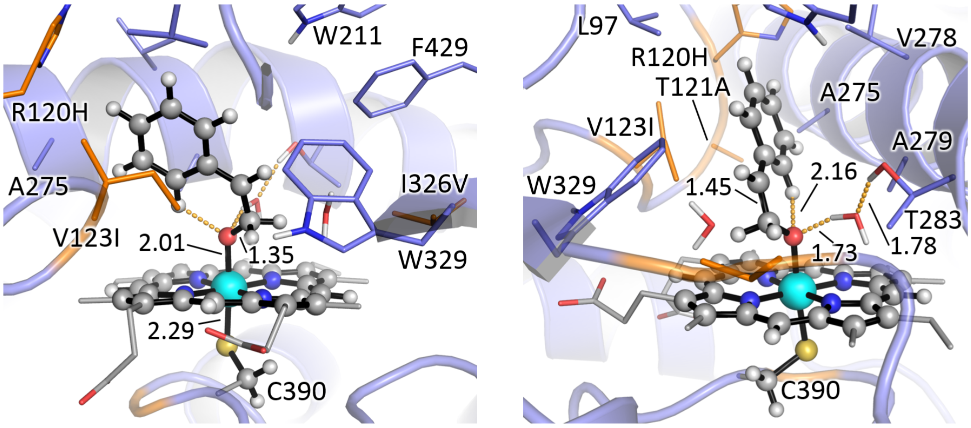

q(Ph) = 0.33
q(C2) = -0.08
q(C1) = 0.08
q(O) = -0.63

ρ(Ph) = 0.00
ρ(C2) = -0.01
ρ(C1) = 0.01
ρ(O) = 0.01

View 1 View 2

aMOx-**Int2**[d]

$\Delta\Delta G$= 1.9 ($\Delta\Delta E$= 1.7)

∠O-C1-C2-C(Ph) = 15.3°
∠H-C1-C2-C(Ph) = -107.8°

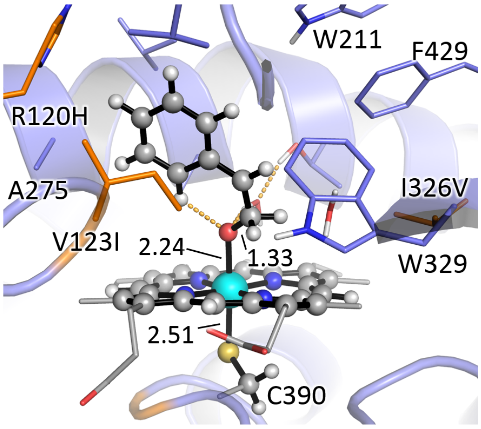

View 1

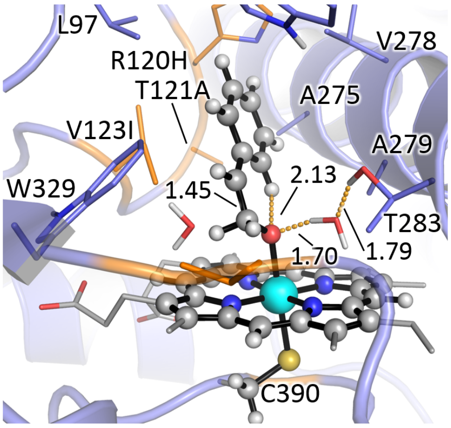

View 2

q(Ph) = 0.32
q(C2) = -0.08
q(C1) = 0.09
q(O) = -0.69

ρ(Ph) = 0.00
ρ(C2) = 0.00
ρ(C1) = 0.01
ρ(O) = 0.06

aMOx-**Int2**^{q}
ΔΔG= 9.1 (ΔΔE= 10.8)

∠O-C1-C2-C(Ph) = 11.4º
∠H-C1-C2-C(Ph) = -113.0º

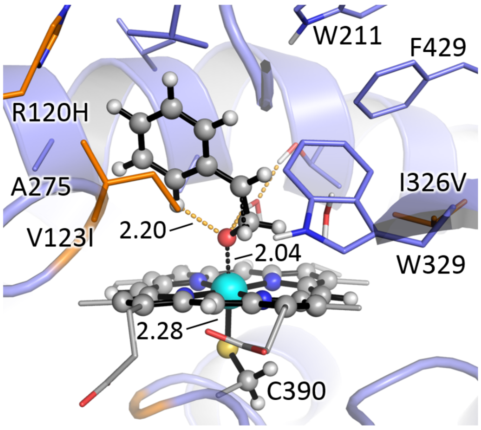

View 1

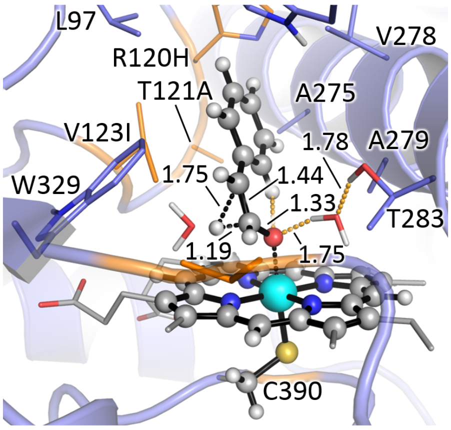

View 2

q(Ph) = 0.29
q(C2) = -0.06
q(C1) = 0.02
q(O) = -0.62

ρ(Ph) = 0.00
ρ(C2) = 0.00
ρ(C1) = 0.00
ρ(O) = 0.00

aMOx-**TS3-cis-re**^{d}
ΔG‡= -0.2 (ΔE‡= -0.2)

∠H-C1-C2-C(Ph) = -99.8º

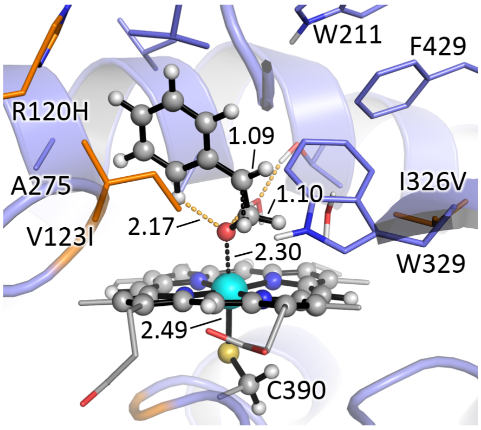

View 1

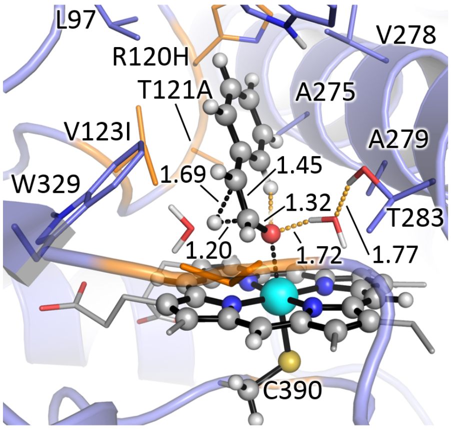

View 2

q(Ph) = 0.26
q(C2) = -0.08
q(C1) = 0.03
q(O) = -0.67

ρ(Ph) = 0.00
ρ(C2) = 0.00
ρ(C1) = 0.00
ρ(O) = 0.04

aMOx-**TS3-cis-re**^{q}
ΔG‡= -1.5 (ΔE‡= -1.8)

∠H-C1-C2-C(Ph) = -99.7º

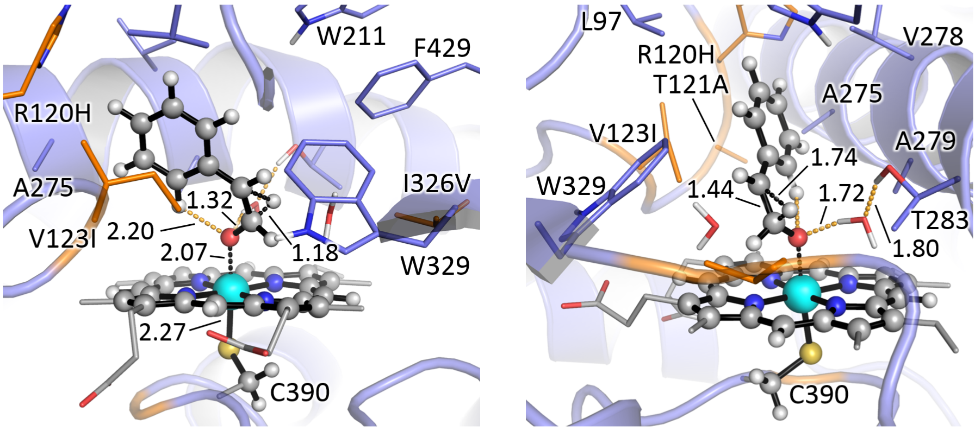

View 1 View 2

q(Ph) = 0.32
q(C2) = -0.17
q(C1) = 0.12
q(O) = -0.62

ρ(Ph) = 0.00
ρ(C2) = 0.00
ρ(C1) = 0.00
ρ(O) = 0.00

aMOx-**TS3-trans-si**^{d}

$\Delta G^{\ddagger}$= 2.1 ($\Delta E^{\ddagger}$= 2.2)

∠H-C1-C2-C(Ph) = 110.1º

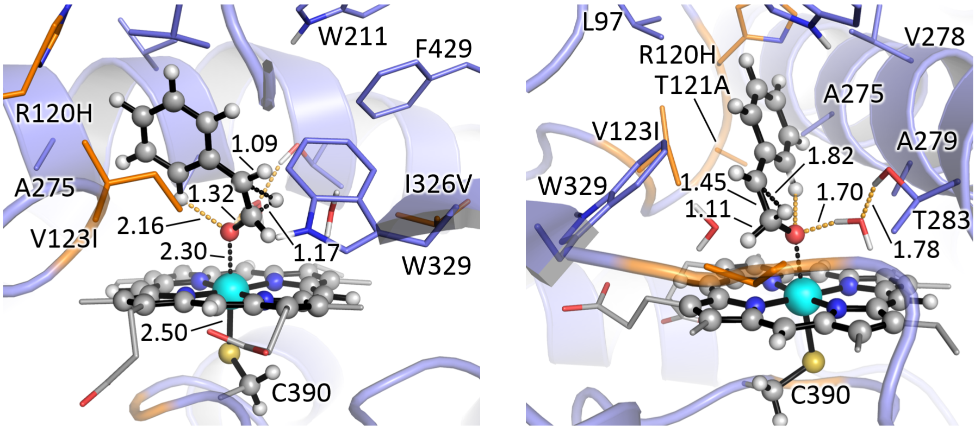

View 1 View 2

q(Ph) = 0.31
q(C2) = -0.17
q(C1) = 0.16
q(O) = -0.68

ρ(Ph) = 0.00
ρ(C2) = 0.00
ρ(C1) = 0.01
ρ(O) = 0.04

aMOx-**TS3-trans-si**^{q}

$\Delta G^{\ddagger}$= 0.8 ($\Delta E^{\ddagger}$= 0.5)

∠H-C1-C2-C(Ph) = 115.0º

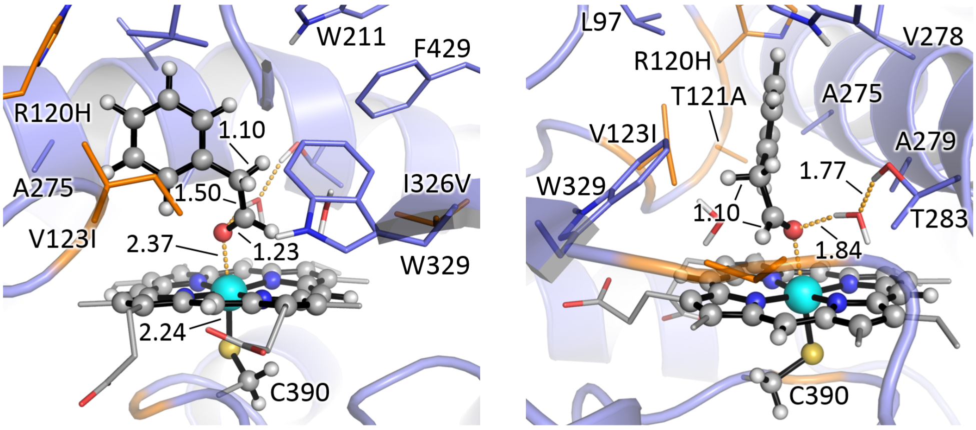

View 1 View 2

aMOx-**3**^{d}

ΔG_r= -41.5 (ΔE_r= -41.9)

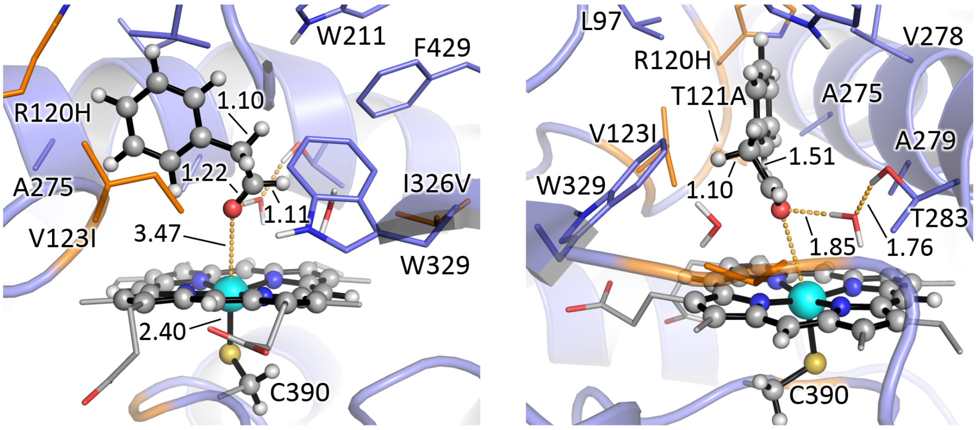

View 1 View 2

aMOx-**3**[q]

ΔG$_r$= -50.0 (ΔE$_r$= -50.9)

D)

D.1)

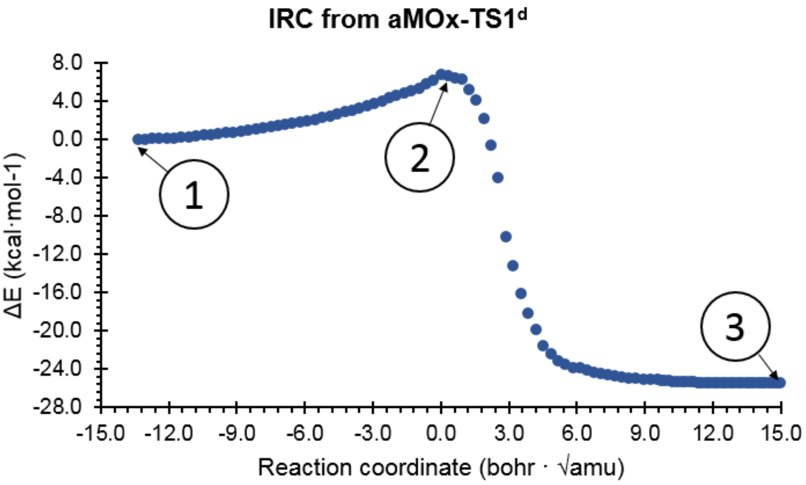

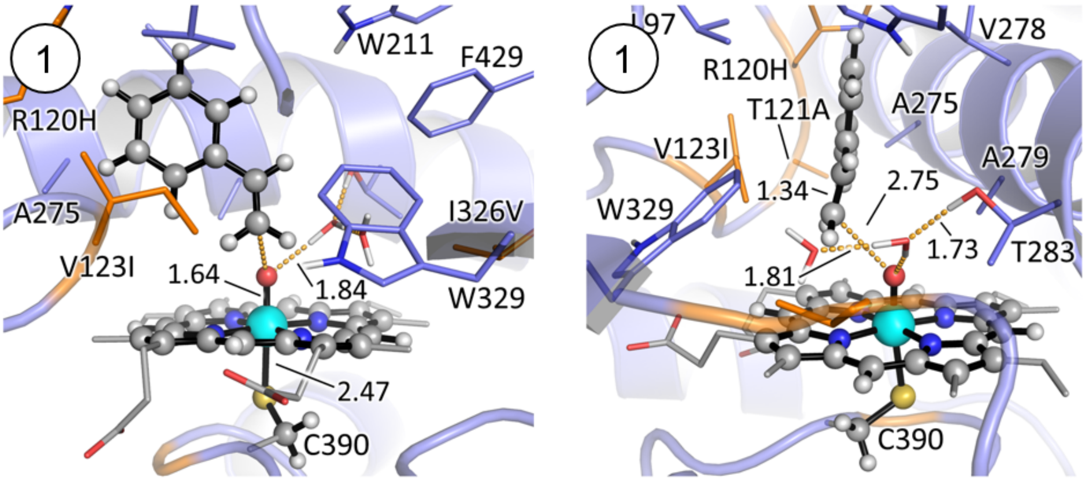

View 1 View 2

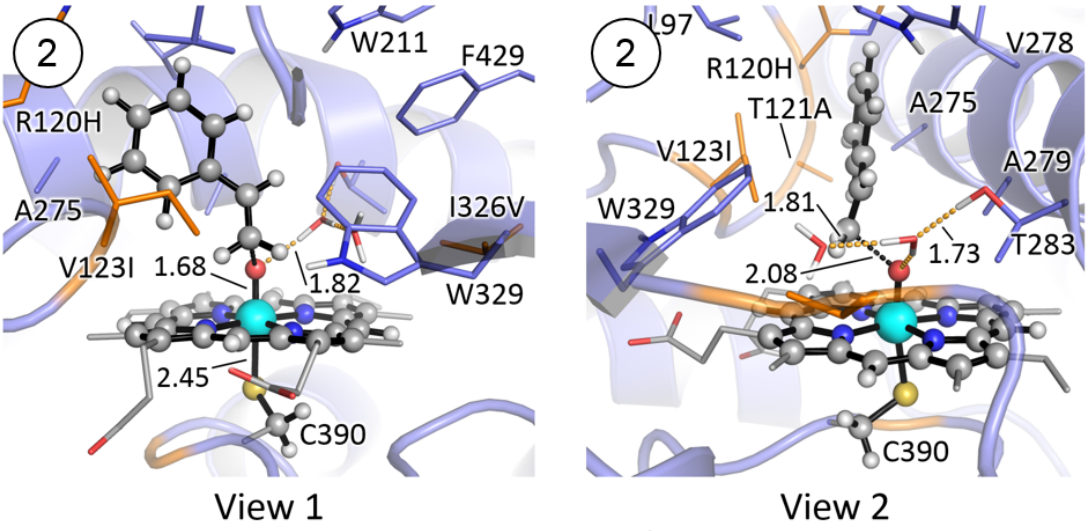

aMOx-**TS1**[d]

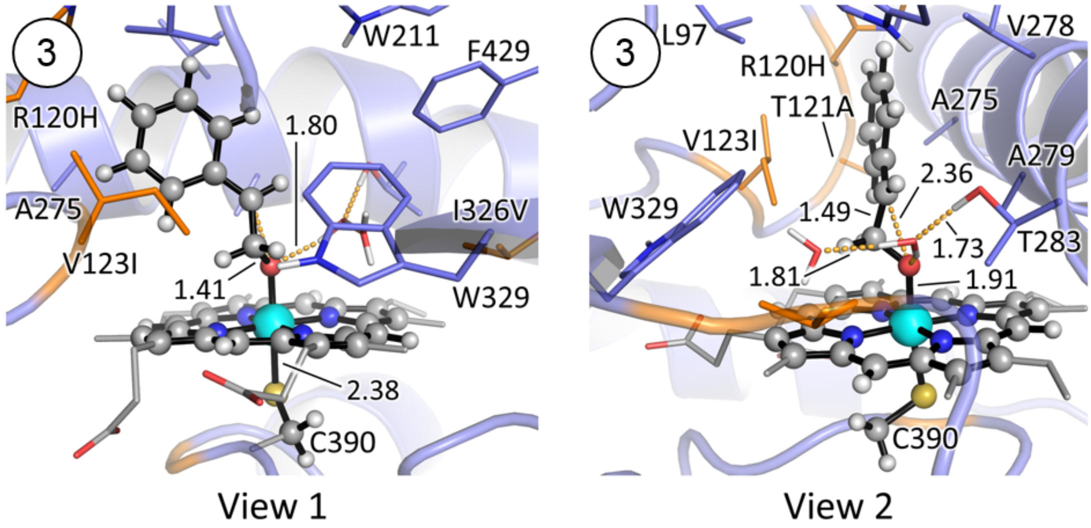

D.2)

IRC from aMOx-TS1[q]

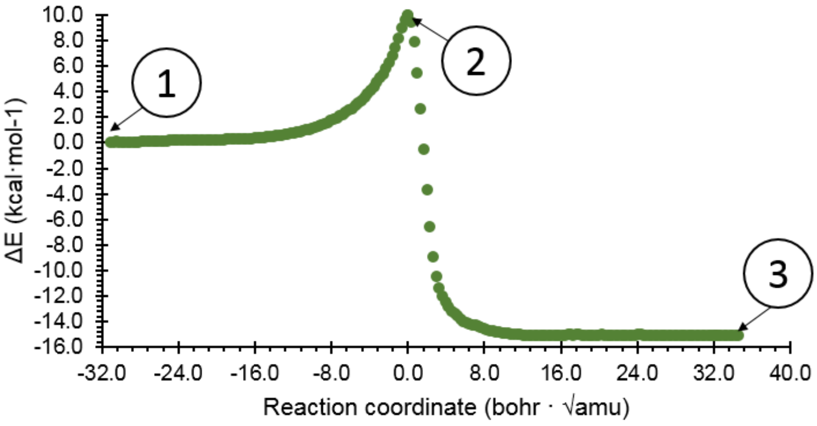

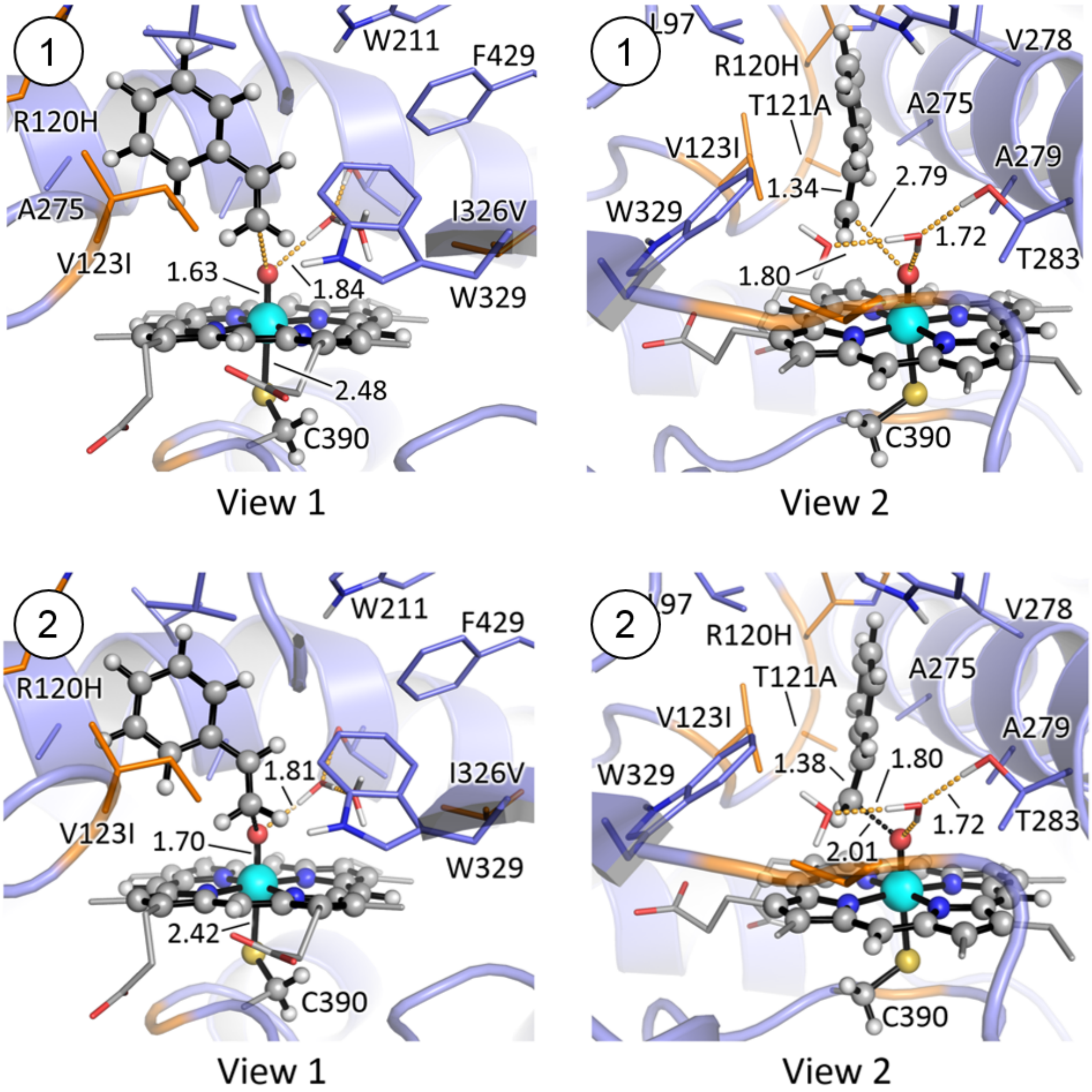
1
W211
F429
R120H
A275
V123I
I326V
1.63
1.84
W329
2.48
C390
View 1
1
L97
V278
R120H
T121A
A275
V123I
A279
W329
1.34
2.79
1.72
T283
1.80
C390
View 2
2
W211
F429
R120H
1.81
I326V
V123I
1.70
W329
2.42
C390
View 1
2
V278
R120H
T121A
A275
V123I
A279
W329
1.38
1.80
T283
1.72
2.01
C390
View 2

aMOx-**TS1**q

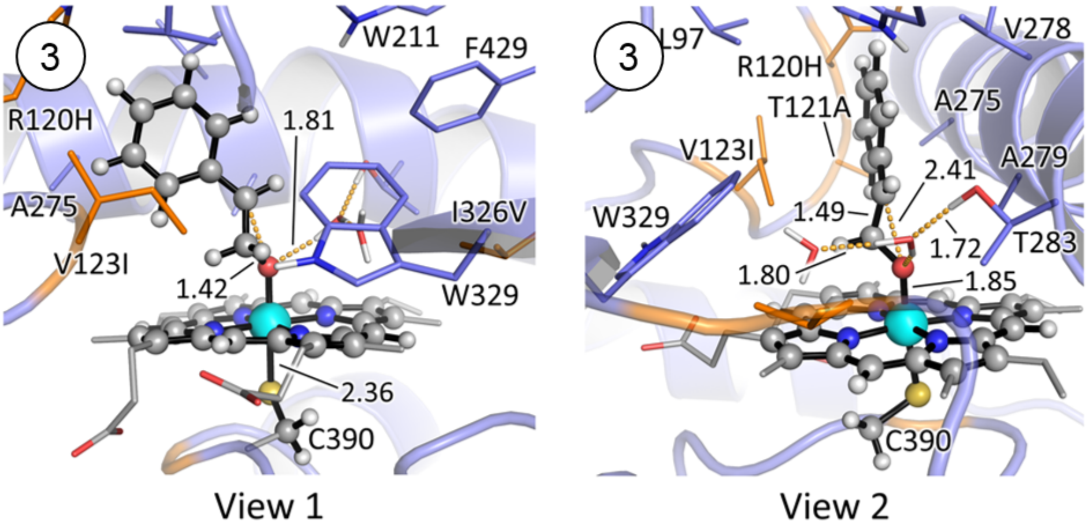
3
W211
F429
R120H
1.81
A275
I326V
V123I
1.42
W329
2.36
C390
View 1
3
L97
V278
R120H
T121A
A275
V123I
A279
2.41
W329
1.49
1.72
T283
1.80
1.85
C390
View 2

E)

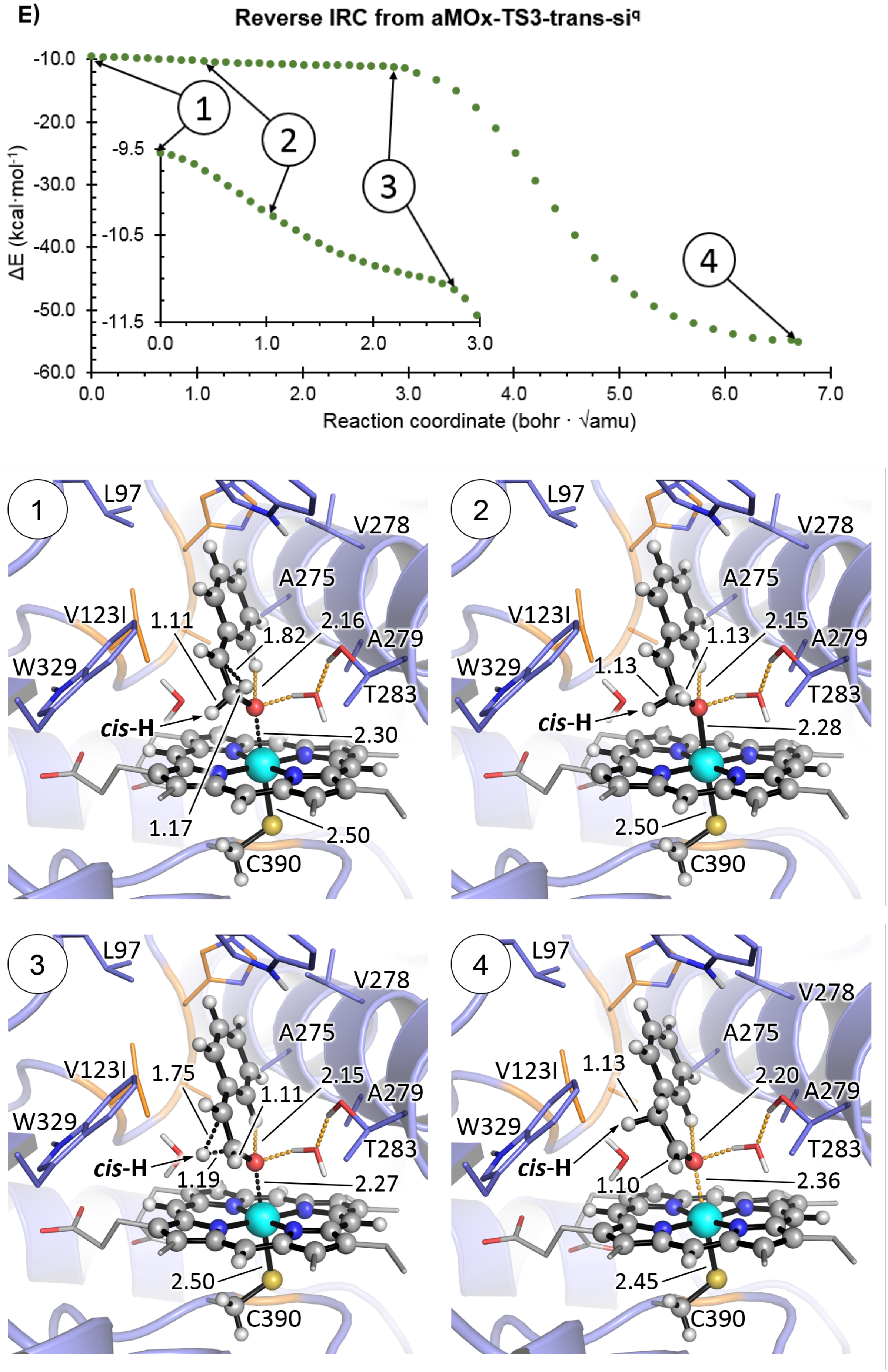
Reverse IRC from aMOx-TS3-trans-si^q
ΔE (kcal·mol⁻¹)
Reaction coordinate (bohr · √amu)
-10.0
-20.0
-30.0
-40.0
-50.0
-60.0
0.0
1.0
2.0
3.0
4.0
5.0
6.0
7.0
-9.5
-10.5
-11.5
1
2
3
4
L97
V278
A275
V123I
W329
A279
T283
cis-H
C390
1.11
1.82
2.16
2.30
1.17
2.50
1.13
2.15
2.28
1.75
1.11
1.19
2.27
1.10
2.20
2.36
2.45

The near attack conformation of styrene considered here corresponds to the major NAC binding pose characterized from MD simulations, with styrene bound exposing the *re* face to the iron-oxo species (see **Figure S11**). This particular binding mode would lead to the formation of the experimentally observed *S*-epoxide product as side product, considering no radical epimerization.

For the reaction occurring in aMOx active site, the covalent radical intermediate aMOx-**Int1** formed from a *re* face NAC in the quartet and doublet electronic states can be optimized as a minimum on the potential energy surface (PES). This is similar to what it is found for $P450_{LA1}$ modelled reaction, and it is indicating that the enzyme active site can stabilize these reactive intermediates due to confinement, as compared to the free-enzyme model system (see **Figure S1**), where the radical intermediate in the doublet electronic state could not be optimized.

The optimized radical intermediates in aMOx active site (aMOx-**Int1**) have a slightly distorted geometry, as compared to the free-enzyme optimized **Int1^{q}** geometry, which stabilizes the radical intermediate and makes it less reactive. This geometric distortion is induced by the interactions occurring with surrounding residues in the active site (see **Figure S20**), and strongly disfavors the epoxide ring closing through aMOx-**TS2** in both electronic states (doublet and quartet). aMOx-**Int1^{q}** is ca. 4 kcal·mol^{-1} more stable than aMOx-**Int1^{d}**.

Epoxide ring closing transition state (aMOx-**TS2**) could be optimized for both electronic states as opposite to the $P450_{LA1}$ catalyzed reaction (**Figures S14** and **S15**), where epoxide ring closing is found to be barrierless from the radical intermediate in the doublet electronic state, and in contrast to the enzyme-free reaction (**Figure S1**), where epoxidation in the doublet state is found to be asynchronous but concerted from first TS1. Significant energy barriers for aMOx-**TS2** transition states are found for each electronic state. In addition, aMOx-**TS2** transition states are higher in energy than the carbocation intermediates aMOx-**Int2** in both doublet and quartet electronic state.

Carbocation formation (aMOx-**Int2**) from radical intermediate aMOx-**Int1** is energetically disfavored in the two studied PES (doublet and quartet). This is caused by the geometric steric control imposed by aMOx active site (see also **Figure S20**), that stabilizes the radical intermediate over the carbocation. A conformational change of aMOx-**Int1** is required to form aMOx-**Int2**, which involves the rotation of the methylene group covalently attached to the O-atom to ensure effective stabilization of the carbocation intermediate due to stereoelectronic effects. The doublet aMOx-**Int2** is more stable than the quartet, suggesting that carbonyl formation might be occurring through this aMOx-**Int2^{d}** intermediate.

The steric conformational control imposed by aMOx active site to the radical intermediate (**aMOx-Int1**) largely disfavors the epoxide forming transition state, which is intrinsically favored due to the existing *dynamic match* between TS1 and TS2. Consequently, the steric control due to confinement in aMOx active site allows the thermal equilibration of the **aMOx-Int1** radical intermediate, overriding the intrinsic *dynamic* preferences, and preferentially following the lowest in energy carbonyl formation pathway through the generation of the carbocation intermediate aMOx-**Int2**.

Once carbocation intermediate is formed (aMOx-**Int2**), hydride migration occurs selectively for the *cis* hydrogen atom though the *re* face, via a barrierless aMOx-**TS3-cis-re** transition state in both the doublet and quartet PES. On the other hand, transition states for the migration of the *trans* hydrogen atom through the *re* face, via **TS3-trans-si** transition states, are ca. $\Delta\Delta G = 2$

kcal·mol^{-1} higher in energy than the corresponding **TS3-cis-re** in the doublet and quartet electronic states. This is due to the preorganized geometry of aMOx-**Int2** intermediates when formed in aMOx active site. Within this geometry, induced by steric restraints imposed by surrounding active site residues, *cis*-H atom is well aligned with the empty *p* orbital on the carbocation C atom for an effective migration from the *re* face of the intermediate (see also **Figure S21**). On the other hand, from this conformation, *trans*-H atom is not well aligned for migrating. Conformational control enables the enantioselective 1,2-hydride migration as corroborated by experiments with deuterated styrene substrates (see **Figures S33** and **S34**).

Due to its high reactivity, aMOx-**Int2^q** and aMOx-**TS3-cis-re^q** in the quartet electronic state could only be optimized applying a set of geometrical restraints (using Gaussian *Modredundant* routine).

aMOx-**Int2^q** structure was optimized with C2–*cis*-H, C2–*trans*-H, and *cis*-H–*trans*-H distances frozen (using the reference values from optimized structure in the doublet state), in order to avoid spontaneous migration during optimization. Subsequent full frequency calculation showed that the optimized aMOx-**Int2^q** structure has all the frequencies positive.

aMOx-**TS3-cis-re^q** structure was optimized with C2–*cis*-H, C1–*cis*-H and O–C1 distances frozen (using the values from optimized structure in the doublet state). Subsequent full frequency calculation showed that the optimized aMOx-**TS3-cis-re^q** structure has only one imaginary frequency corresponding to the H-migration coordinate.

Intrinsic reaction coordinate (IRC) calculations starting from the fully optimized aMOx-**TS3-trans-si^q** support the high reactivity of carbocation intermediate aMOx-**Int2^q** in the quartet electronic state. From the reverse IRC calculated starting from the fully optimized aMOx-**TS3-trans-si^q** (**Figure S19-D)**, a geometry similar to the optimized aMOx-**Int2^q** structure (see earlier) is explored, however it is not considered as a minimum on the PES by the algorithm. Consequently, the IRC calculation proceeds, and it leads to the spontaneous migration of the *cis*-H through a geometry similar to the aMOx-**TS3-cis-re^q** optimized structure, to finally form the phenyl aldehyde product (**3**) with an *R*-configuration. This is indicating that, in the quartet electronic state, 1,2-hydride migration involving the *cis*-H atom from the *re* face is highly favored as compared to the trans-H migration from the *si* face once the carbocation intermediate aMOx-**Int2^q** is formed.

Finally, the alternative pro-*R* epoxidation aMOx-**TS2** transition states, leading to *R*-epoxide product from radical intermediate aMOx-**Int1**, were also optimized using QM/MM calculations. Calculations show that energy barriers associated to pro-*R* epoxidation aMOx-**TS2** for each electronic state, are much higher than those for the pro-*S* epoxide formation. This is in line with the active site reshaping characterized from MD simulations, which already indicated that pro-*S* epoxidation (*re* face) catalytically relevant binding modes are favored in aMOx active site, being the pro-*R* (*si* face) ones disfavored due to sterics. These QM/MM results are also in line with the high *S*-epoxide enantioselectivity experimentally observed.

Figure S20: QM/MM optimized structures of key intermediates formed in aMOx variant active site: **A**) covalent radical intermediate (aMOx-**Int1^{q}**); and **B**) covalent carbocation intermediate (aMOx-**Int2^{d}**). Lowest in energy electronic states are considered (see **Figure S19**).

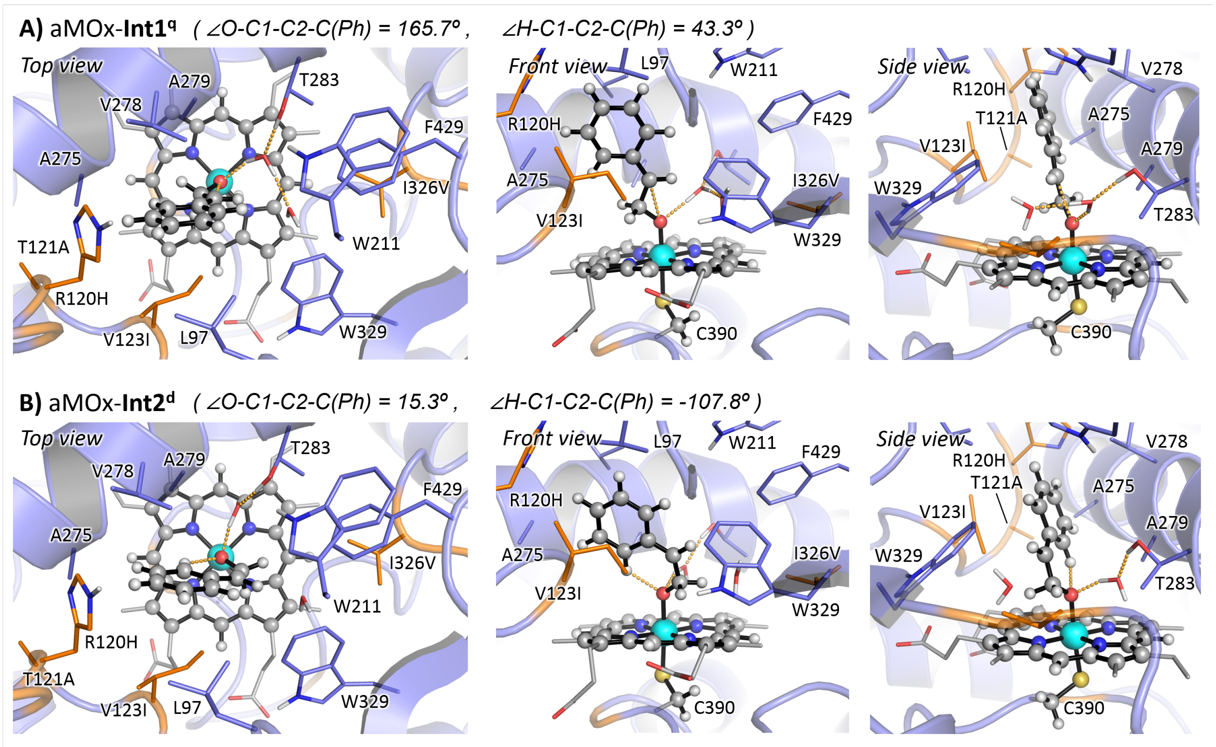

Radical intermediate optimized in aMOx active site (aMOx-**Int1**) has the phenyl ring oriented in an anti-periplanar conformation with respect the O–Fe group, as described by the ∠(O-C1-C2-C(Ph)) dihedral angle (∠(O-C1-C2-C(Ph)) = 165.7º). This is due to steric restrictions imposed by active site residues L97, W211, V278, A279, W329, and F429.

For an effective epoxide ring closing, the radical localized on the C2 atom *p* orbital has to be syn-periplanar to the O–Fe group allowing an efficient orbital overlap leading to the C2–O bond formation. This is described by the value of ∠(O-C1-C2-C(Ph)) angle in the ideal enzyme-free epoxide forming **TS2** (∠(O-C1-C2-C(Ph)) is ca. 93º (see **Figure 2** in the main text, and **Figure S1**). The ∠(O-C1-C2-C(Ph)) dihedral angle value in the QM/MM optimized aMOx-**TS2^{d}** is ca. 127º, which describes a large geometric distortion from the ideal enzyme-free structure, and that causes a dramatic increase of the activation barrier for the epoxide ring closing in aMOx active site (see also discussions in **Figure S19**). Consequently, geometric control due to confinement in aMOx active site stabilizes the radical intermediate in a conformation that highly disfavors epoxidation.

Covalent carbocation intermediate (aMOx-**Int2**) conformation in aMOx is also distorted due to confinement as compared to the optimal one characterized from enzyme-free model calculations (**Figure 2** in the main text, and **Figure S1**). Within this conformation, stereoelectronic stabilization of the carbocation intermediate can effectively occur, but there exists a slight distortion of aMOx-**Int2** along the ∠(Fe-O-C1-C2) dihedral angle (∠(Fe-O-C1-C2) = -161.9º) as compared to the free-enzyme system ((∠(Fe-O-C1-C2) = -178.5º). This geometric distortion preorganizes *cis*-H for a preferential 1,2-hydride migration from the *re* face of the intermediate by aligning it with the empty *p* orbital of the carbocation C center

(∠(*cis*-H-C1-C2-C(Ph)) = -107.8º , ∠(*trans*-H-C1-C2-C(Ph)) = 151.2º see also discussions in **Figure S21**).

QM/MM optimized aMOx-**Int1** and aMOx-**Int2** structures have the phenyl ring of the intermediate tightly packed by hydrophobic L97, V123I, W211, V278, A279, W329, and F429 active site residues, occupying the same position in aMOx active site. The main difference between the two intermediate structures is the positioning of the methylene-O group in the active site pocket. In the radical aMOx-**Int1**, the methylene-O group is oriented to the left-handed side of the cavity (from a front view perspective), while it is oriented to the right-handed side in the optimized carbocation aMOx-**Int2** structure, directly pointing to the I326V position. It is important to highlight that original I326 residue is mutated in aMOx to a less bulkier valine (I326V), which makes more space for positioning the methylidene group at this position, and thus contributing to the geometric control and preorganization of aMOx-**Int2** for an enantioselective 1,2-hydride migration.

Figure S21: Active site packing occurring in aMOx QM/MM optimized key transition states: **A)** aMOx-**TS2** ; and **B)** aMOx-**TS3**. Only lowest in energy electronic states are considered here (see **Figure S19**). For each QM/MM optimized structure two different orientations (side and top view) are shown, using two different representation models with and without space-filling model for key active site residues (L97, W211, V278, A279, W329, and F429).

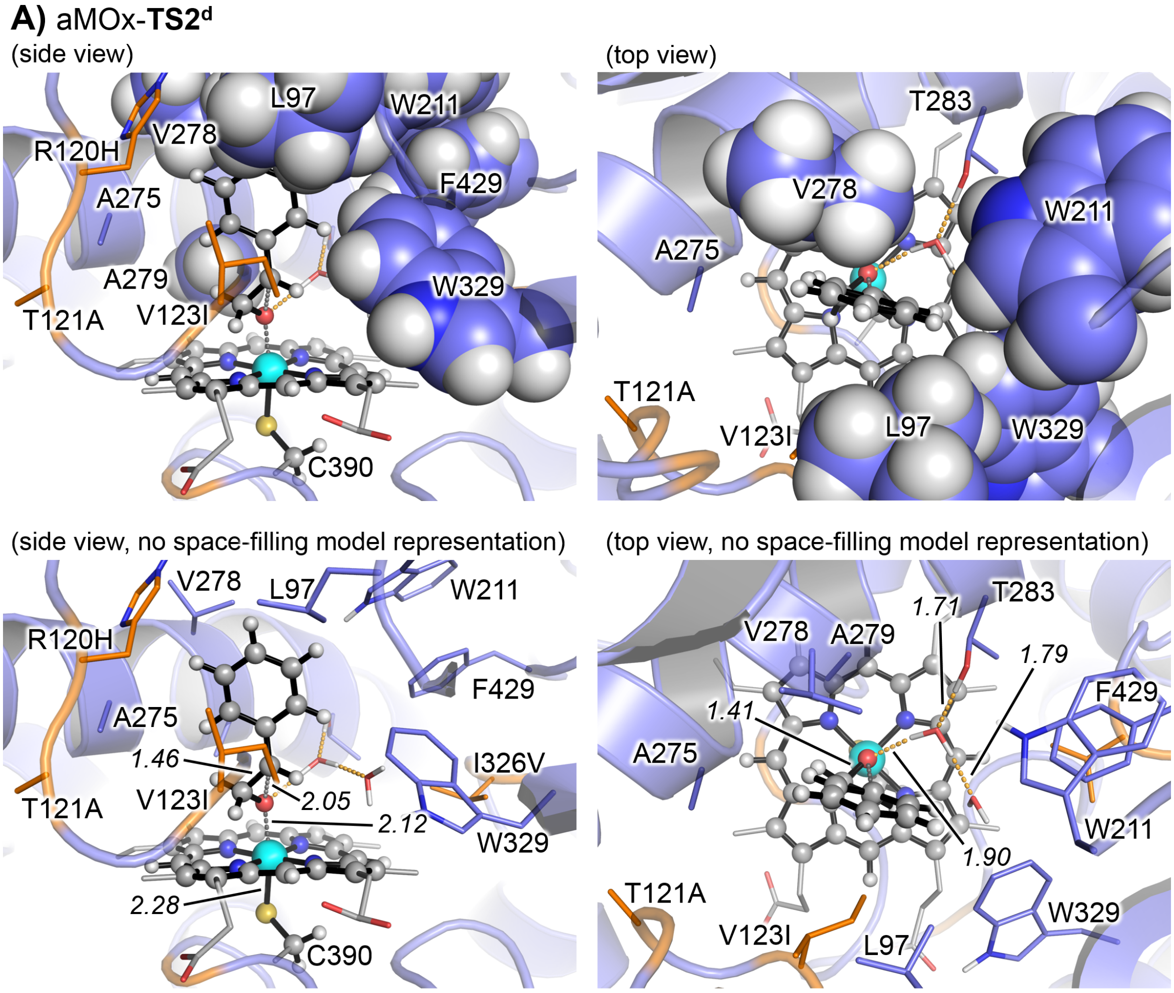

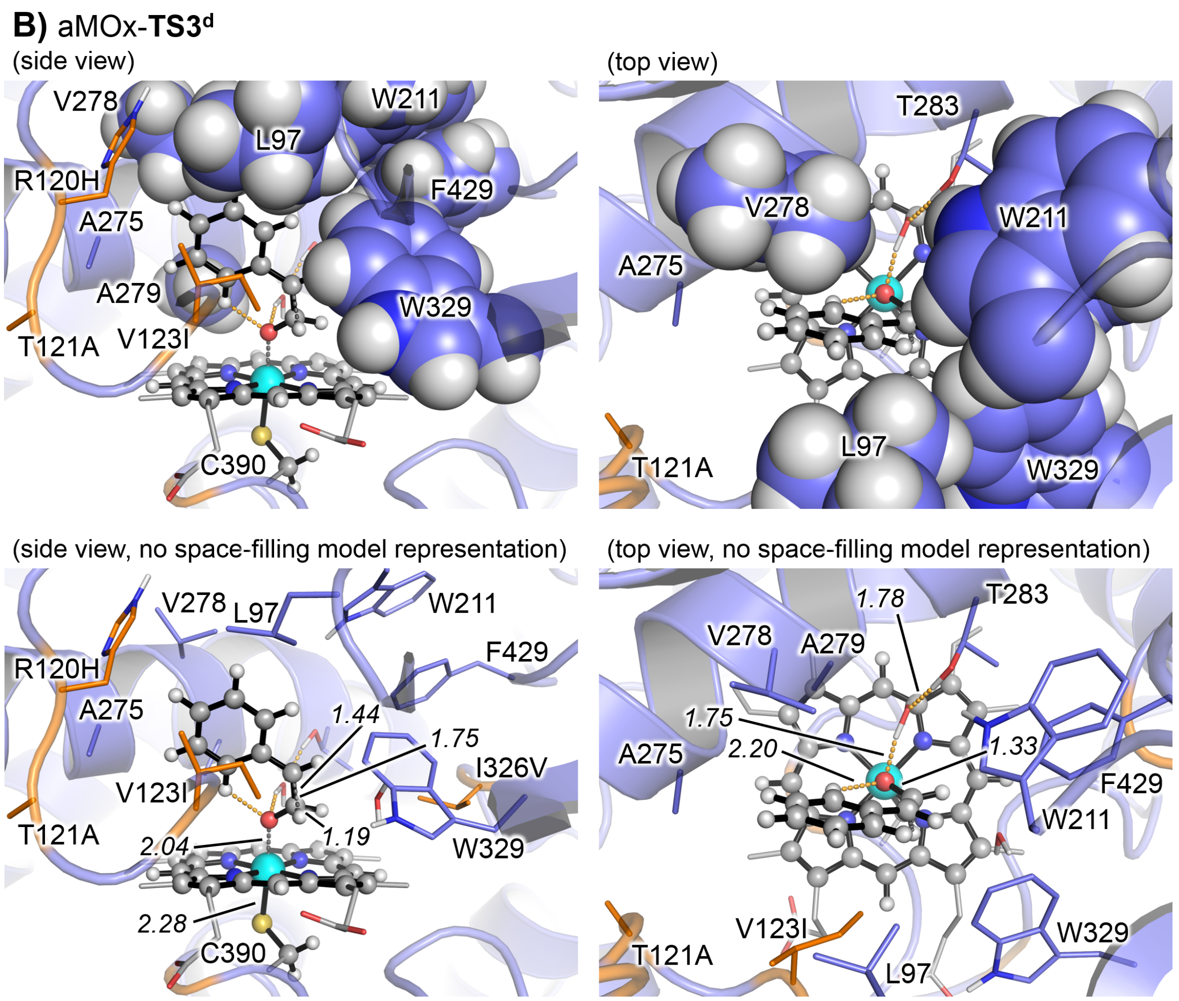

aMOx active site packing significantly increases the activation barrier of aMOx-**TS2** (doublet and quartet), mainly due to steric interactions with L97, W211, V278, W329, and F429 residues (see discussion in **Figure S19**).

Similar interactions occurring in the optimized aMOx-**TS3** (in both doublet and quartet electronic states). These interactions favor a slight distortion of aMOx-**Int2** along the ∠(Fe-O-C1-C2) dihedral angle (∠(Fe-O-C1-C2) = -161.9º, **Figure S20**) as compared to the free-enzyme system ((∠(Fe-O-C1-C2) = -178.5º, **Figure S1**) . This geometric distortion preorganizes *cis*-H for a preferential 1,2-hydride migration over *trans*-H, by aligning it with the empty *p* orbital of the carbocation C center (see discussion in **Figure S19**).

Figure S22: QM/MM calculations were used to study the rotation of the C1–C2 bond in the covalent radical intermediate formed in the aMOx active site, for both doublet (q) and quartet (q) electronic states (aMOx-**Int1^{d}** and aMOx-**Int1^{q}**, **Figure S19**).

A) QM/MM relaxed scan calculations were carried out along the rotation of ∠(O-C1-C2-C(Ph)) dihedral angle starting from the optimized radical intermediate species in the doublet and quartet states (aMOx-**Int1^{d}** ∠(O-C1-C2-C(Ph) ca. -166.1° ; and aMOx-**Int1^{q}** ∠(O-C1-C2-C(Ph) ca. -165.7°). Calculations were carried out using the mechanical embedding (ME) approach and at the (U)B3LYP/6-31G(d)+SDD(Fe):Amber FF14SB level.

B) Single point calculations were performed on the optimized points along the relaxed scan coordinate reported in **A**), but using an electrostatic embedding (EE) scheme and at (U)B3LYP/ Def2TZVP:Amber FF14SB level.

C) QM/MM optimized rotation transition states (**TS-rotation^{q}** and **TS-rotation^{d}**) in both doublet and quartet electronic states. They were optimized starting from the highest in energy point on the relaxed scan coordinates (**A**). Mulliken charges (*q*) and spin densities (ρ) for the phenyl group (sum of all C and H atoms), C2 benzylic position, C1 and O are given in a.u. These transition states connect the corresponding radical intermediate aMOx-**Int1** and a aMOx-**Int2**-like geometry with radical character.

D) QM/MM computed relative stabilities in terms of electronic energy at the QM region (ΔE_{QM}), QM/MM ONIOM electronic energy (ΔE), enthalpy (ΔH), and Gibbs energy (ΔG) for the transition state associated to C1-C2 rotation. Energy values were obtained at the (U)B3LYP/Def2TZVP:AmberFF14SB//(U)B3LYP/631G(d)+SDD(Fe):Amber FF14SB level. Doublet (d) and quartet (q) electronic states were considered, and all energies are referred considering the lowest in energy aMOx-**Int1^{q}** structure as zero.

Energies, distances, Mulliken charges and spin density values are given in kcal· mol^{-1}, Angstrom (Å), and a.u., respectively.

A)

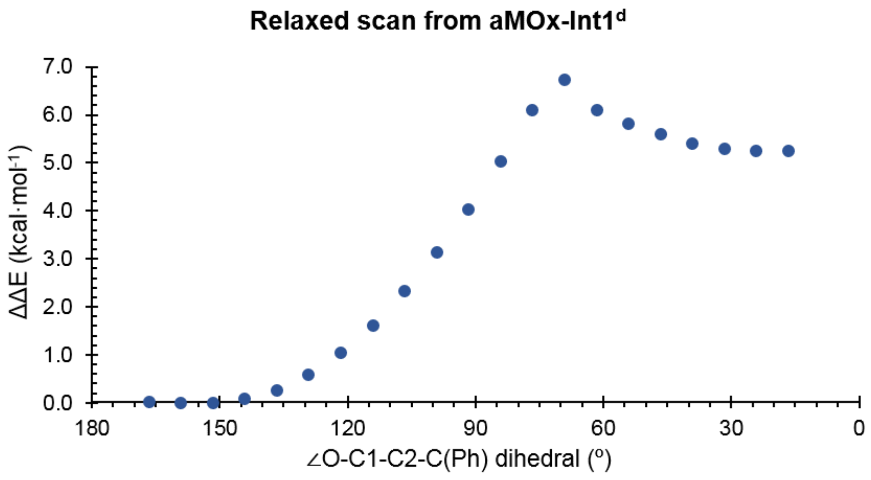

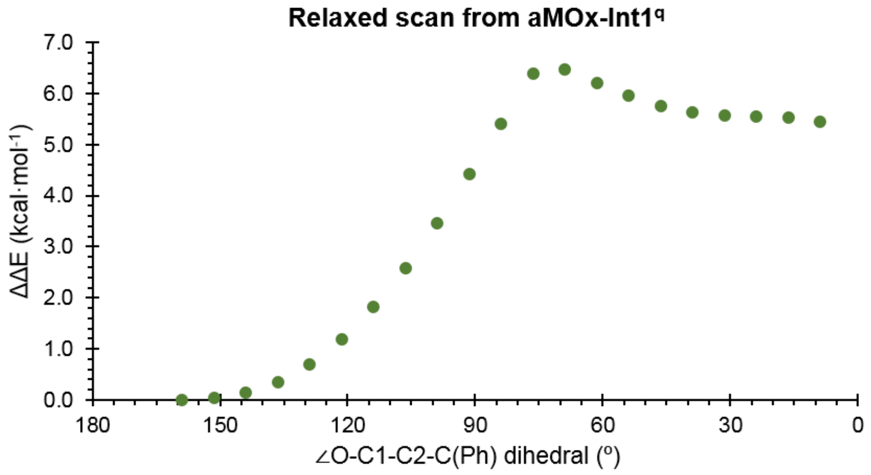
Relaxed scan from aMOx-Int1q
ΔΔE (kcal·mol-1)
7.0
6.0
5.0
4.0
3.0
2.0
1.0
0.0
180
150
120
90
60
30
0
∠O-C1-C2-C(Ph) dihedral (°)

B)

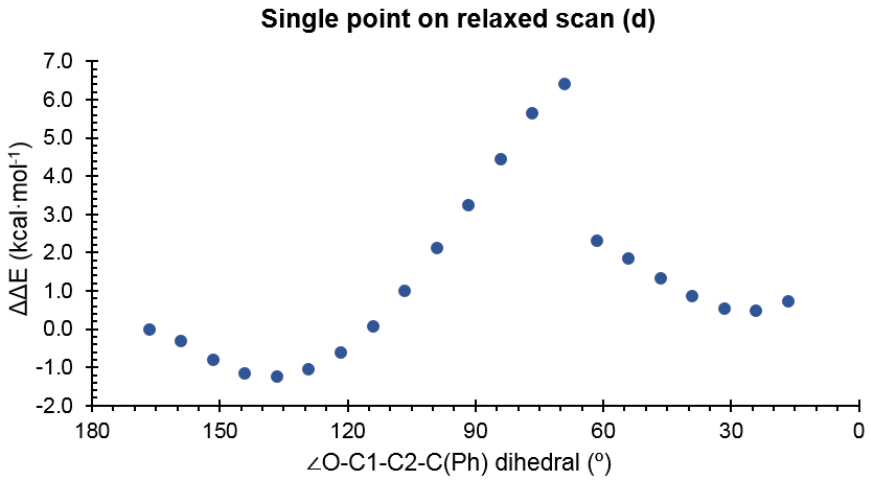
Single point on relaxed scan (d)
ΔΔE (kcal·mol-1)
7.0
6.0
5.0
4.0
3.0
2.0
1.0
0.0
-1.0
-2.0
180
150
120
90
60
30
0
∠O-C1-C2-C(Ph) dihedral (°)

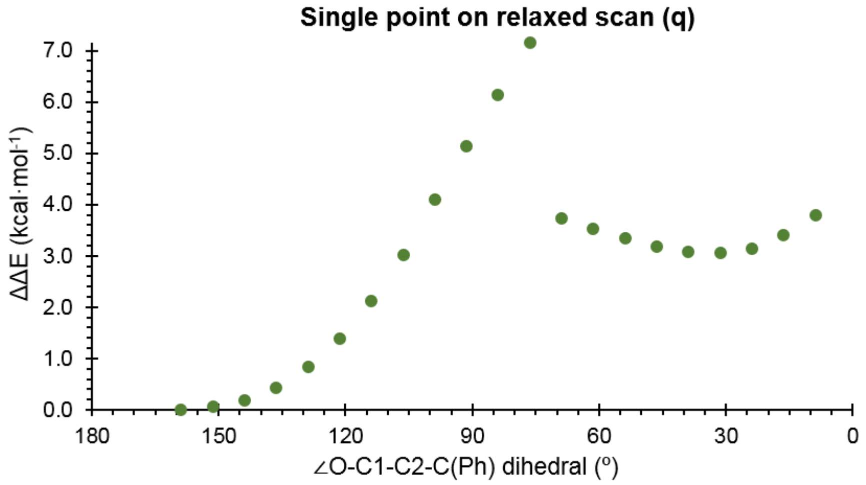
Single point on relaxed scan (q)
ΔΔE (kcal·mol-1)
7.0
6.0
5.0
4.0
3.0
2.0
1.0
0.0
180
150
120
90
60
30
0
∠O-C1-C2-C(Ph) dihedral (°)

C)

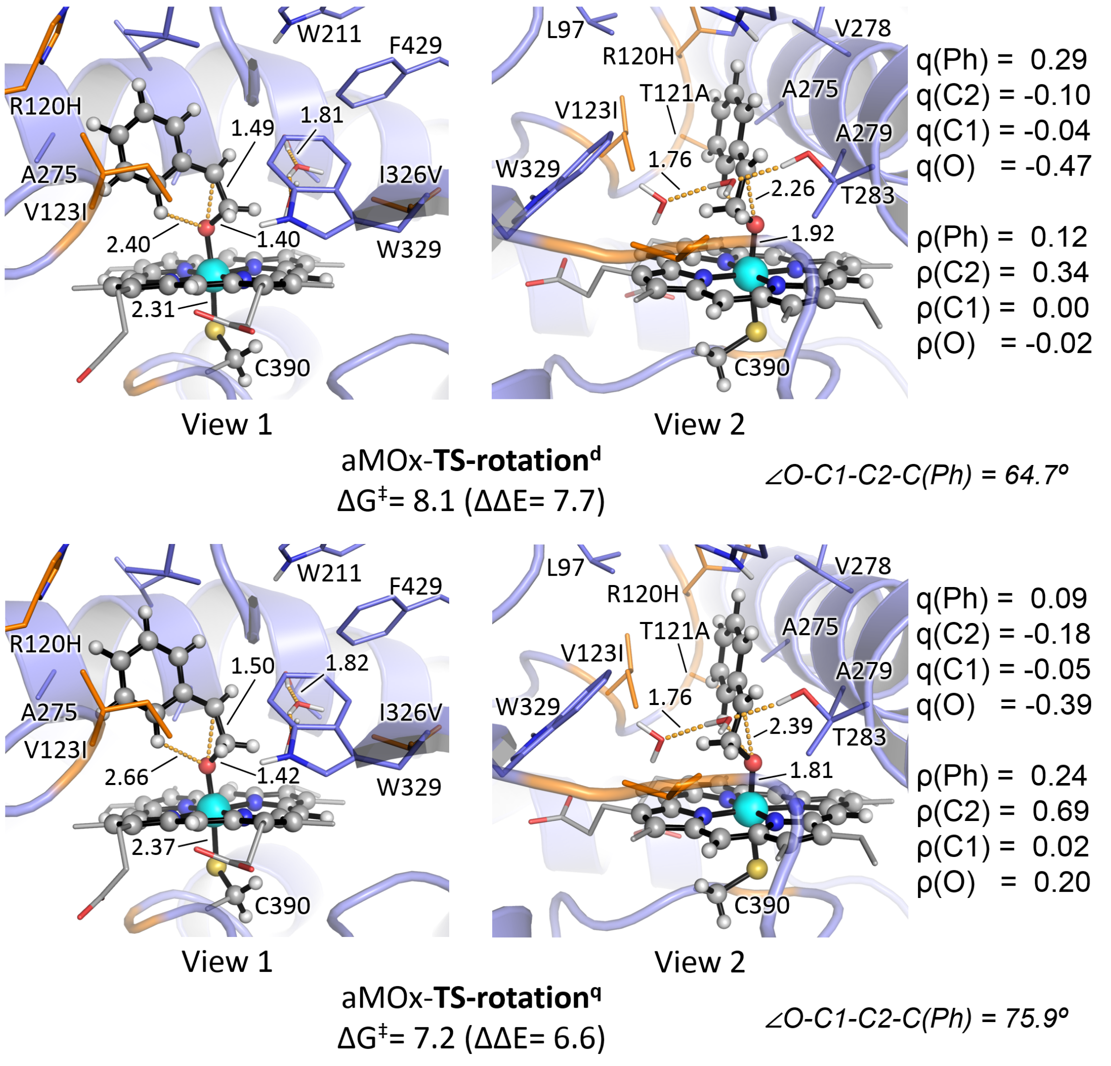

D)

Structure	Electronic State	$\Delta\Delta E_{QM}$	ΔΔE	ΔΔH	ΔΔG
aMOx-**Int1** [a]	doublet (d)	-0.6	3.7	3.2	4.0
	quartet (q)	0.0	0.0	0.0	0.0
aMOx-**TS2** [a] (pro-S)	doublet (d)	4.2	7.2	7.6	9.7
	quartet (q)	3.8	9.4	9.3	10.7
aMOx-**TS-rotation**	doublet (d)	10.7	11.4	10.6	12.1
	quartet (q)	8.4	6.6	5.6	7.2
aMOx-**Int2** [a]	doublet (d)	-10.0	5.4	4.7	5.9
	quartet (q)	-8.0	10.8	9.5	9.1

[a] From **Figure S19**

Focusing on the lowest in energy electronic state, quartet, QM/MM relaxed scan calculations indicated the existence of a significant energy barrier for the rotational conformational change that connects aMOx-**Int1**[q] and an aMOx-**Int2**-like geometry, around ca. 7 kcal·mol^{-1} in electronic energy. Starting from the highest in energy point on the relaxed scan rotational

coordinate (∠O-C1-C2-C(Ph) ca. 70º, ΔE ca. 7.0 kcal·mol^{-1}), a rotational transition state aMOx-**TS-rotation**[q] was fully optimized, with a ΔG‡ = 7.2 kcal·mol^{-1} (∠O-C1-C2-C(Ph) = 75.9º). This is a significant rotational barrier as compared to what was found for the enzyme-free system (see **Figure S2**) and the wildtype LA1 system (see **Figure S18**), which is attributed to the higher confinement and packing that the radical intermediate has in the evolved aMOx active site. Although higher, this rotational barrier that converts **aMOx-Int1** to a **aMOx-Int2**-like geometry is significantly lower (ca. 3.5 kcal·mol^{-1}lower) than the epoxide forming barrier **aMOx-TS2** (ΔG‡ = 10.7 kcal·mol^{-1}, see **Figure S19**).

Consequently, these new results further support the conclusion that a strong conformational control over the radical reactive intermediate is responsible for the carbonyl formation over the epoxide formation promoted by the laboratory evolved aMOx enzyme active site.

Figure S23: QM/MM exploration of aMOx variant catalyzed oxidation of styrene (**1**) substrate. QM/MM calculations were carried out starting from a MD-relaxed structure of the covalent intermediate (**Int1**) formed in aMOx active site (**Figure S13-B**), that mimics the minor explored, catalytically relevant, binding pose of styrene substrate characterized from restrained-MD simulations (green marker selected snapshot in **Figure S11)**. This corresponds to a styrene binding pose with its *si* face exposed to the iron-oxo species (pro-*R* epoxidation).

A) QM/MM computed relative stabilities in terms of electronic energy at the QM region (ΔE_{QM}), QM/MM ONIOM electronic energy (ΔE), enthalpy (ΔH), and Gibbs energy (ΔG) for the different optimized species. Energy values were obtained at the (U)B3LYP/Def2TZVP: AmberFF14SB//(U)B3LYP/6-31G(d)+SDD(Fe):AmberFF14SB level, with the same MM parameters used in MD simulations. An Electrostatic Embedding was used (see computational details). Doublet (d) and quartet (q) electronic states were considered, and all energies are referred considering the lowest in energy aMOx-$\mathbf{1}^q$ structure as zero.

B) QM/MM calculated Gibbs free energy profile. Relative Gibbs free energies (ΔG) and electronic energies (ΔE, in parenthesis) are reported.

C) QM/MM optimized structures. Atoms included in the QM region are shown in ball-and-stick representation, and residues in the MM region are shown in sticks. Mutated residues are highlighted in orange. Mulliken charges (*q*) and spin density (ρ) values for the phenyl group (Ph, sum of all C and H atoms), C2 benzylic position, C1 and O, are reported.

Energies, distances, and Mulliken charges and spin density values are given in kcal·mol^{-1}, Angstrom (Å), and a.u., respectively.

A)

Structure	Electronic State	ΔE_{QM}	ΔE	ΔH	ΔG
aMOx-**1**	doublet (d)	-1.3	-0.2	-0.1	0.4
	quartet (q)	0.0	0.0	0.0	0.0
aMOx-**TS1**	doublet (d)	14.9	29.3	28.7	30.4
	quartet (q)	17.3	25.8	24.8	25.6
aMOx-**Int1**	doublet (d)	-7.6	7.8	7.8	9.2
	quartet (q)	-7.2	4.6	5.3	6.5
aMOx-**TS2**	doublet (d)	-4.5	10.2	10.2	12.8
	quartet (q)	-9.0	7.4	7.5	9.4
aMOx-**2-R**	doublet (d)	-35.5	-20.2	-18.4	-17.4
	quartet (q)	-45.8	-25.5	-23.7	-23.0
aMOx-**Int2**	doublet (d)	-7.5	1.5	1.1	3.2
	quartet (q)	-12.5	3.2	2.2	2.9
aMOx-**TS3-cis-si**	doublet (d)	-7.3	2.2	0.9	3.1
	quartet (q)	-13.5	3.2	1.6	2.7
aMOx-**TS3-trans-re**	doublet (d)	-6.4	1.9	0.6	2.8
	quartet (q)	-13.2	2.9	1.3	2.1
aMOx-**3**	doublet (d)	-55.2	-48.7	-47.1	-47.4
	quartet (q)	-66.6	-53.3	-52.0	-53.9

B)

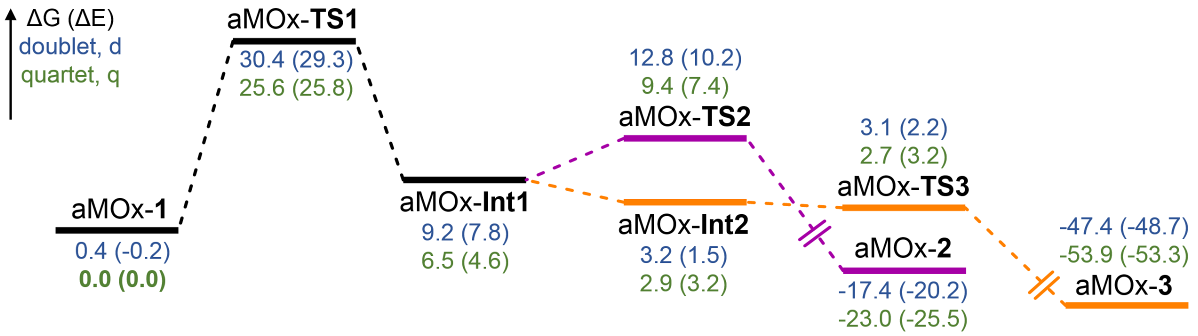

C)

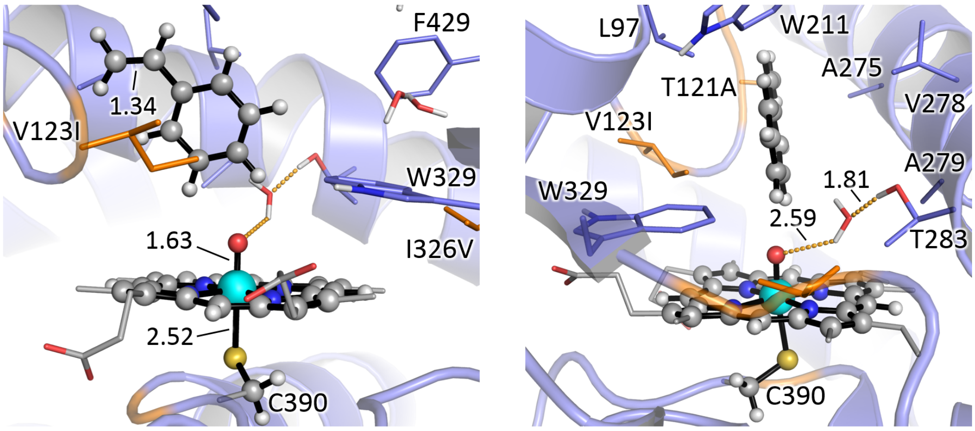

View 1 View 2

aMOx-**1**[d]

ΔG= 0.4 (ΔE= -0.2)

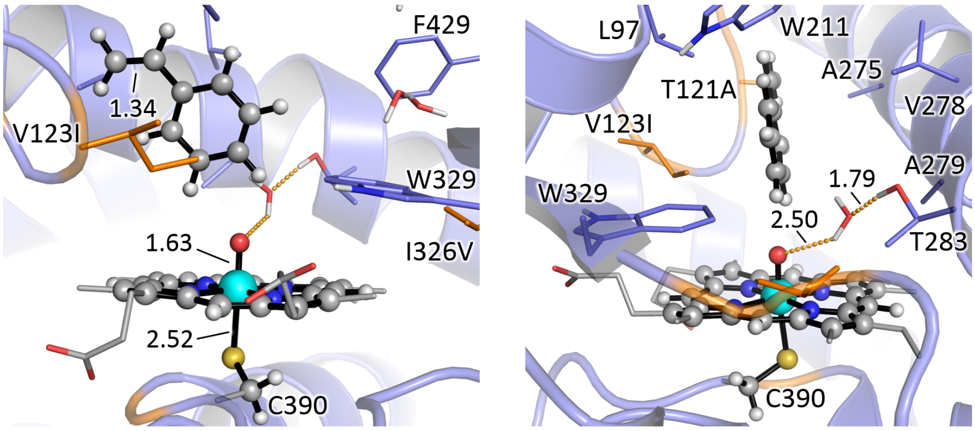

View 1 View 2

aMOx-**1**[q]

ΔG= 0.0 (ΔE= 0.0)

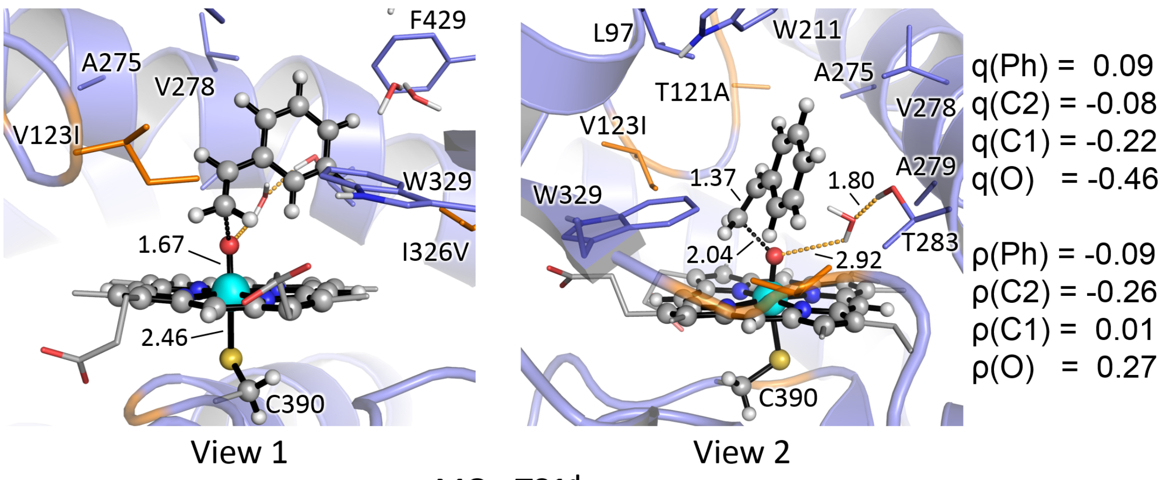

aMOx-**TS1**[d]
$\Delta G^{\ddagger}$= 30.0 ($\Delta E^{\ddagger}$= 29.5)

|∠Fe-O-C1-C2| = 176.1°

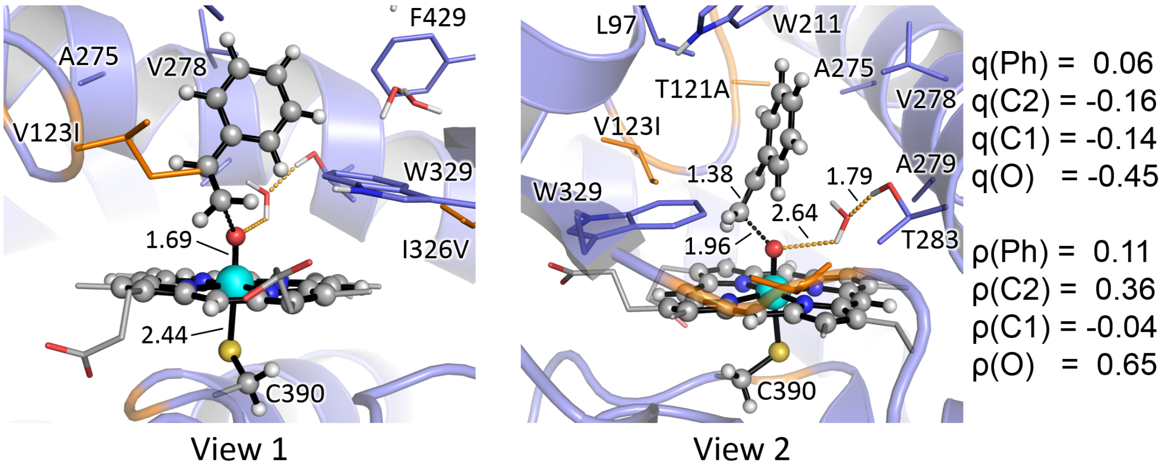

aMOx-**TS1**[q]
$\Delta G^{\ddagger}$= 25.8 ($\Delta E^{\ddagger}$= 25.6)

|∠Fe-O-C1-C2| = 148.2°

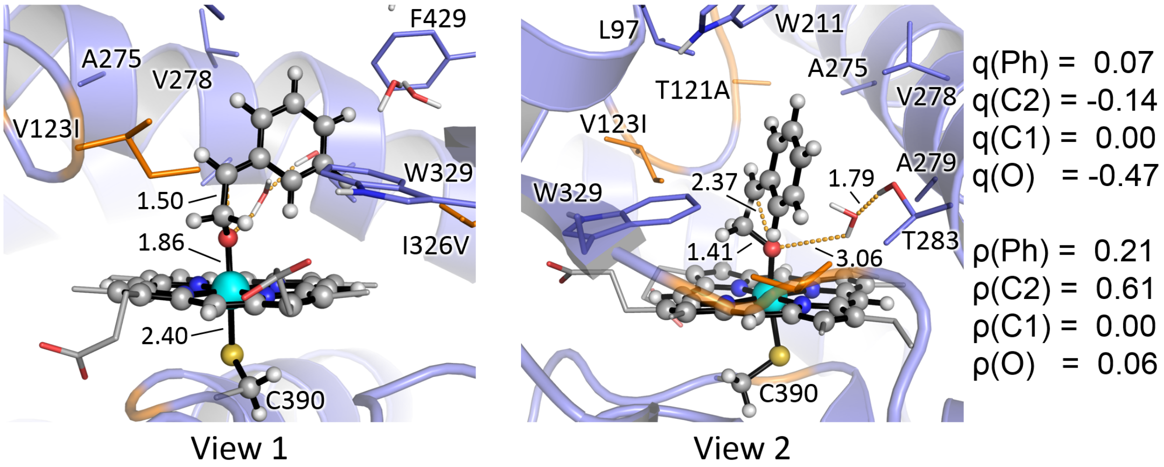

aMOx-**Int1**[d]
ΔG_r= 8.8 (ΔE_r= 8.0)
$\Delta\Delta G$= 2.7 ($\Delta\Delta E$= 3.2)

∠O-C1-C2-C(Ph) = -60.6°
∠H-C1-C2-C(Ph) = 60.7°

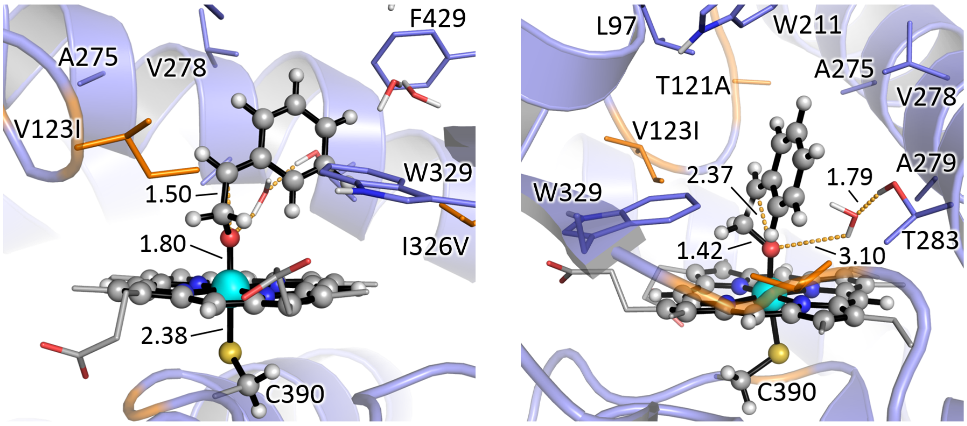

View 1 View 2

q(Ph) = 0.04
q(C2) = -0.17
q(C1) = -0.02
q(O) = -0.40

ρ(Ph) = 0.25
ρ(C2) = 0.71
ρ(C1) = 0.02
ρ(O) = 0.19

aMOx-**Int1**[q]
ΔG_r= 6.5 (ΔE_r= 4.6)
$\Delta\Delta G$= 0.0 ($\Delta\Delta E$= 0.0)

∠O-C1-C2-C(Ph) = -59.2º
∠H-C1-C2-C(Ph) = 60.8º

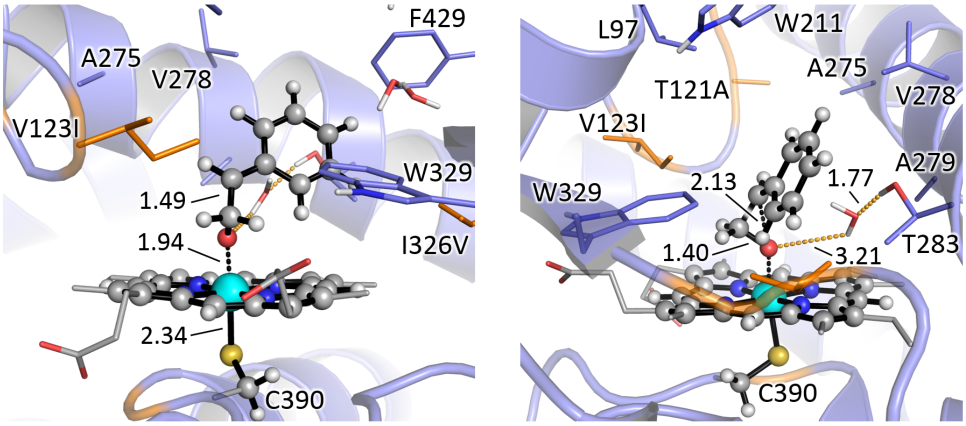

View 1 View 2

q(Ph) = 0.11
q(C2) = -0.11
q(C1) = -0.05
q(O) = -0.40

ρ(Ph) = 0.18
ρ(C2) = 0.54
ρ(C1) = 0.00
ρ(O) = -0.07

aMOx-**TS2**[d]
$\Delta G^‡$= 3.6 ($\Delta E^‡$= 2.4)

∠O-C1-C2-C(Ph) = -80.4º

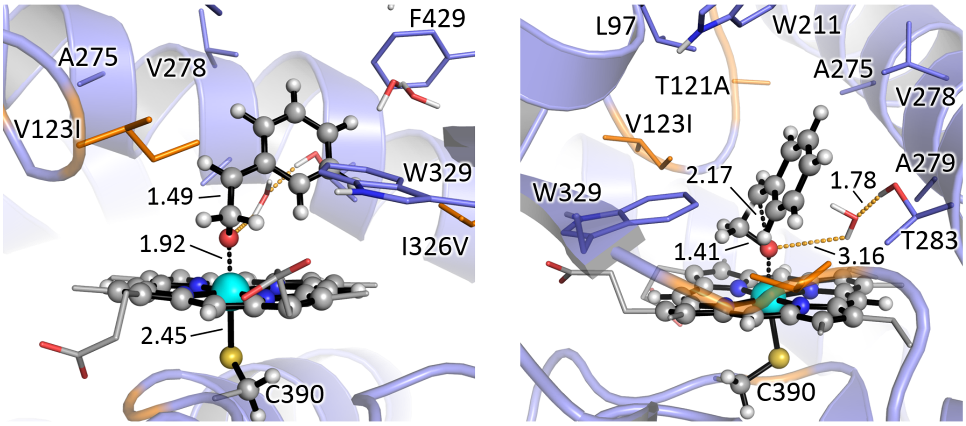

View 1 View 2

q(Ph) = 0.23
q(C2) = -0.06
q(C1) = -0.05
q(O) = -0.42

ρ(Ph) = -0.02
ρ(C2) = -0.06
ρ(C1) = 0.01
ρ(O) = 0.04

aMOx-**TS2**[q]
$\Delta G^‡$= 2.9 ($\Delta E^‡$= 2.7)

∠O-C1-C2-C(Ph) = -77.7º

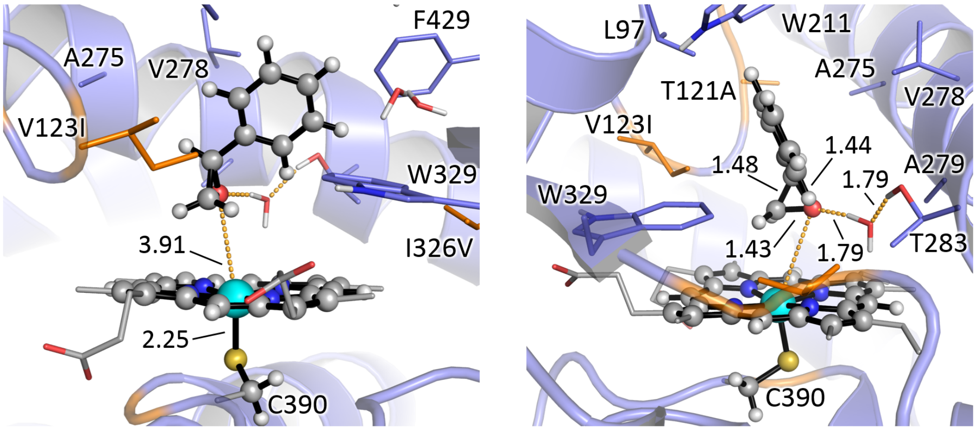

View 1 View 2

aMOx-**2-R**[d]

ΔG_r= -26.6 (ΔE_r= -28.0)

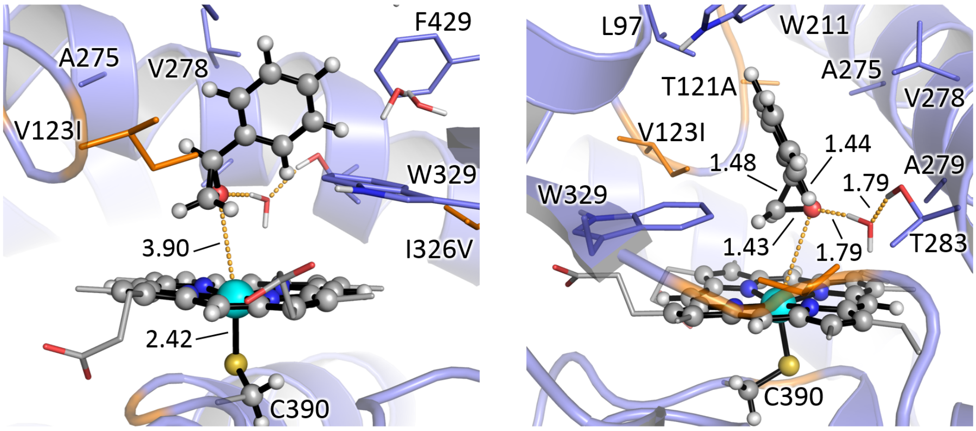

View 1 View 2

aMOx-**2-R**[q]

ΔG_r= -29.5 (ΔE_r= -30.1)

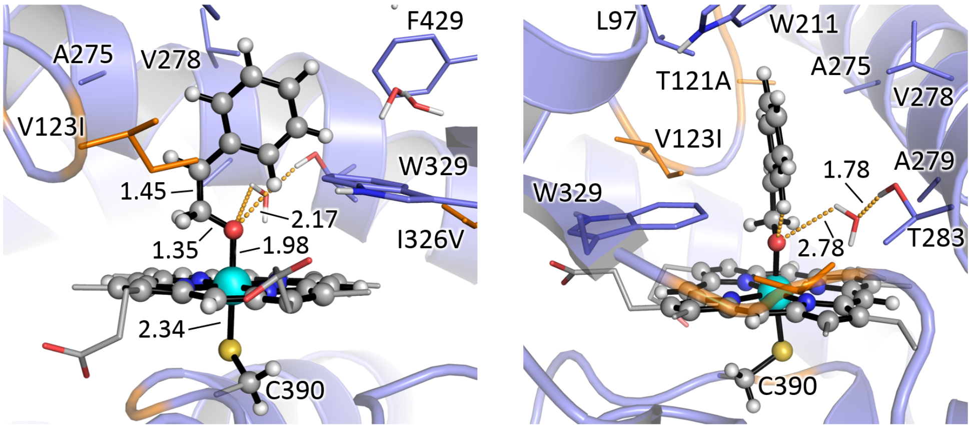

q(Ph) = 0.24
q(C2) = -0.12
q(C1) = 0.05
q(O) = -0.52

ρ(Ph) = 0.03
ρ(C2) = 0.06
ρ(C1) = 0.00
ρ(O) = -0.02

View 1 View 2

aMOx-**Int2**[d]

$\Delta\Delta G$= -6.0 ($\Delta\Delta E$= -6.3)

∠O-C1-C2-C(Ph) = -1.1°

∠H-C1-C2-C(Ph) = 126.5°

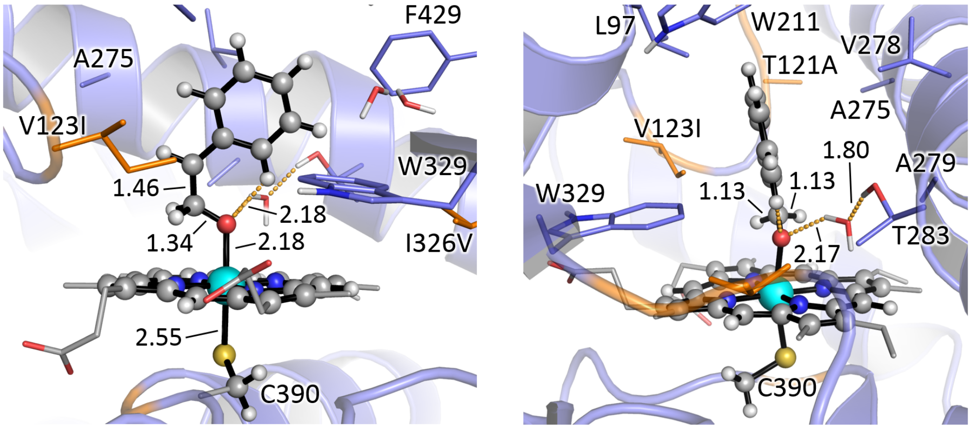

q(Ph) = 0.26
q(C2) = -0.10
q(C1) = 0.06
q(O) = -0.61

ρ(Ph) = 0.05
ρ(C2) = 0.15
ρ(C1) = -0.02
ρ(O) = 0.02

View 1 View 2

aMOx-**Int2**[q]
ΔΔG= -3.6 (ΔΔE= -1.5)

∠O-C1-C2-C(Ph) = -1.4º
∠H-C1-C2-C(Ph) = 126.0º

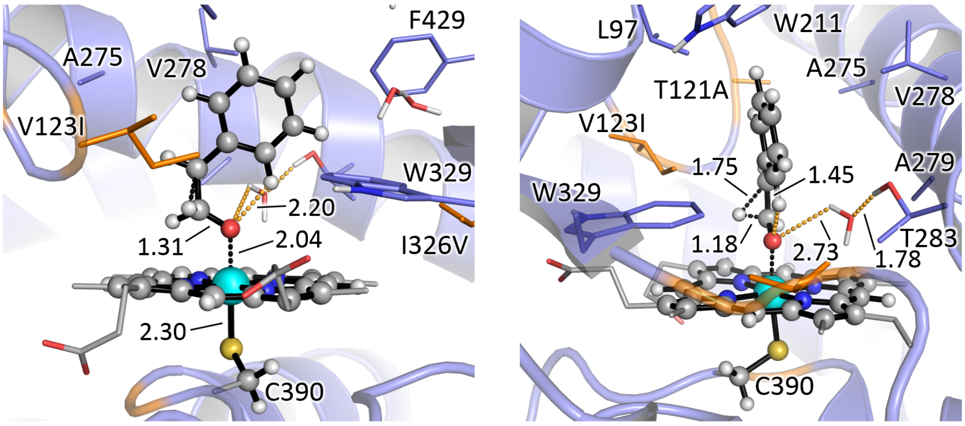

q(Ph) = 0.27
q(C2) = -0.13
q(C1) = 0.03
q(O) = -0.51

ρ(Ph) = -0.01
ρ(C2) = -0.02
ρ(C1) = 0.01
ρ(O) = 0.00

View 1 View 2

aMOx-**TS3-cis-si**[d]
$\Delta G^{\ddagger}$= -0.1 ($\Delta E^{\ddagger}$= -0.6)

∠H-C1-C2-C(Ph) = 106.0º

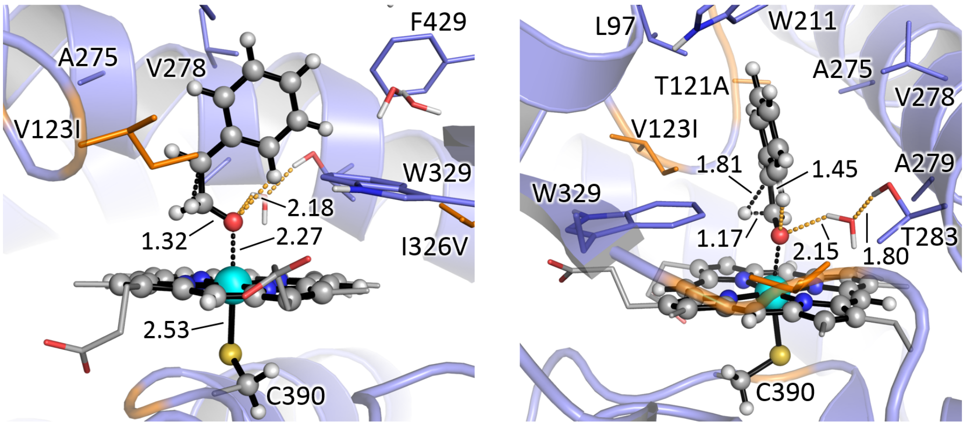

q(Ph) = 0.27
q(C2) = -0.09
q(C1) = 0.04
q(O) = -0.61

ρ(Ph) = 0.01
ρ(C2) = 0.02
ρ(C1) = 0.00
ρ(O) = 0.04

View 1 View 2

aMOx-**TS3-cis-si**[q]
$\Delta G^{\ddagger}$= -0.2 ($\Delta E^{\ddagger}$= 0.1)

∠H-C1-C2-C(Ph) = 109.2º

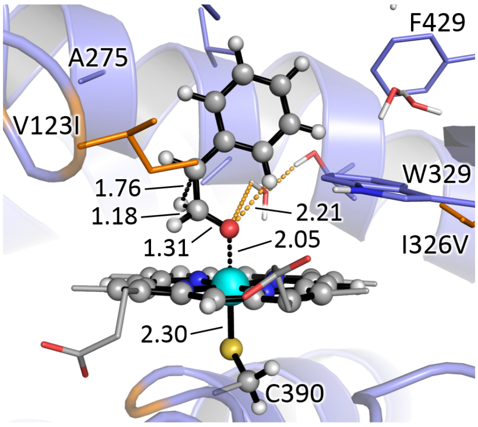

View 1

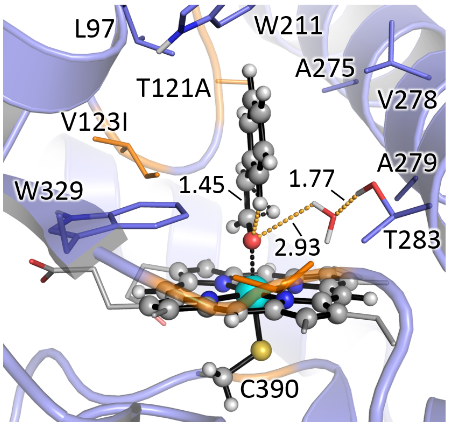

View 2

q(Ph) = 0.22
q(C2) = -0.14
q(C1) = 0.08
q(O) = -0.49

ρ(Ph) = -0.01
ρ(C2) = -0.03
ρ(C1) = 0.01
ρ(O) = 0.01

aMOx-**TS3-trans-re**[d]
ΔG‡= -0.4 (ΔE‡= 0.4)

∠H-C1-C2-C(Ph) = -107.6°

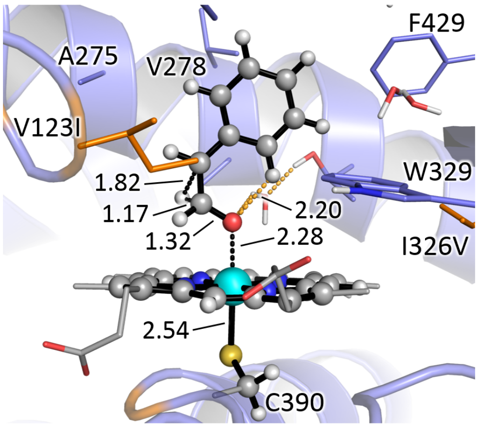

View 1

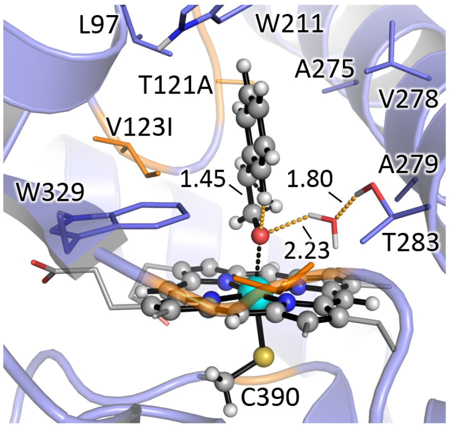

View 2

q(Ph) = 0.26
q(C2) = -0.11
q(C1) = 0.08
q(O) = -0.59

ρ(Ph) = 0.00
ρ(C2) = 0.01
ρ(C1) = 0.00
ρ(O) = 0.05

aMOx-**TS3-trans-re**[q]
ΔG‡= -0.8 (ΔE‡= -0.2)

∠H-C1-C2-C(Ph) = -111.1°

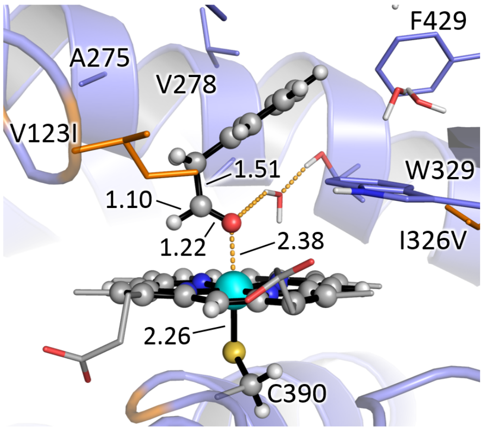

View 1

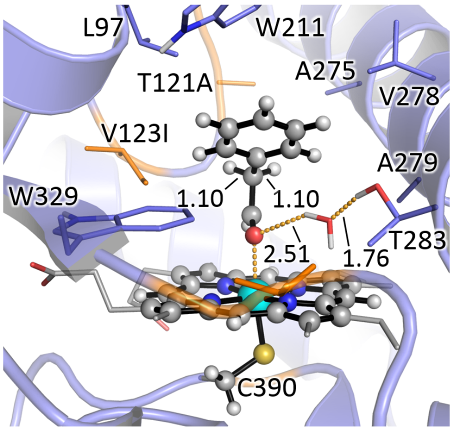

View 2

aMOx-**3**[d]
ΔG_r= -50.6 (ΔE_r= -50.3)

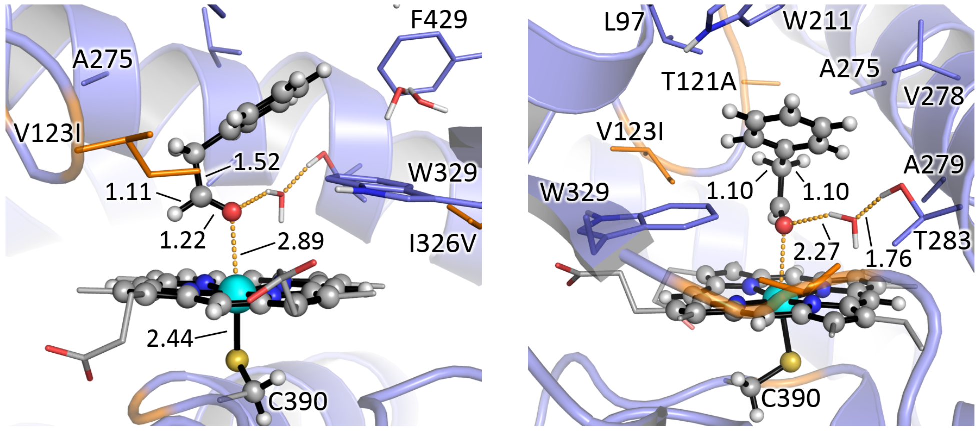

View 1 View 2

aMOx-**3**^{q}

ΔG_r= -56.8 (ΔE_r= -56.4)

QM/MM calculations were also carried out considering a near attack conformation of styrene exposing the *re* face to the iron-oxo species (see **Figure S11**). This corresponds to the minor, less favorable, catalytically relevant binding mode of styrene characterized from restrained-MD simulations, which would lead to the minor *R*-epoxide as a side product.

QM/MM calculations showed that the substrate is not well stabilized by aMOx active site residues in this *si* face relative binding pose. During the optimization procedure, starting from a NAC conformation in which styrene is close to the iron-oxo, styrene is displaced far from the iron-oxo active species (**Figure S13-B**). The rate-limiting C1–O bond formation **TS1** was also calculated, becoming highly disfavored (activation barriers, $\Delta G^{‡}$, higher than ca. 25 kcal·mol^{-1} for both electronic states). Calculated activation barriers are much higher than the equivalent ones calculated for the preferential styrene NAC binding mode (*re* face oriented, **Figure S19**). These QM/MM results are in line with the preferential *re* face oriented catalytically relevant binding mode of styrene in aMOx active site, characterized by substrate-restrained MD simulations (see **Figure S11**). Due to active site shape, styrene *re* face binding modes are much more stable than *si* face ones, which are much less reactive.

Therefore, QM/MM calculations together with MD simulations carried out suggest that reactivity of styrene through *si* face binding modes in aMOx active site is highly disfavored and not expected.

Figure S24: QM/MM optimized structures corresponding to the lowest in energy calculated reaction pathways in **A)** P450$_{LA1}$ and **B)** aMOx. See **Figures S15** and **S19** for a complete description of the calculated mechanisms. Energy values are given in kcal·mol^{-1}.

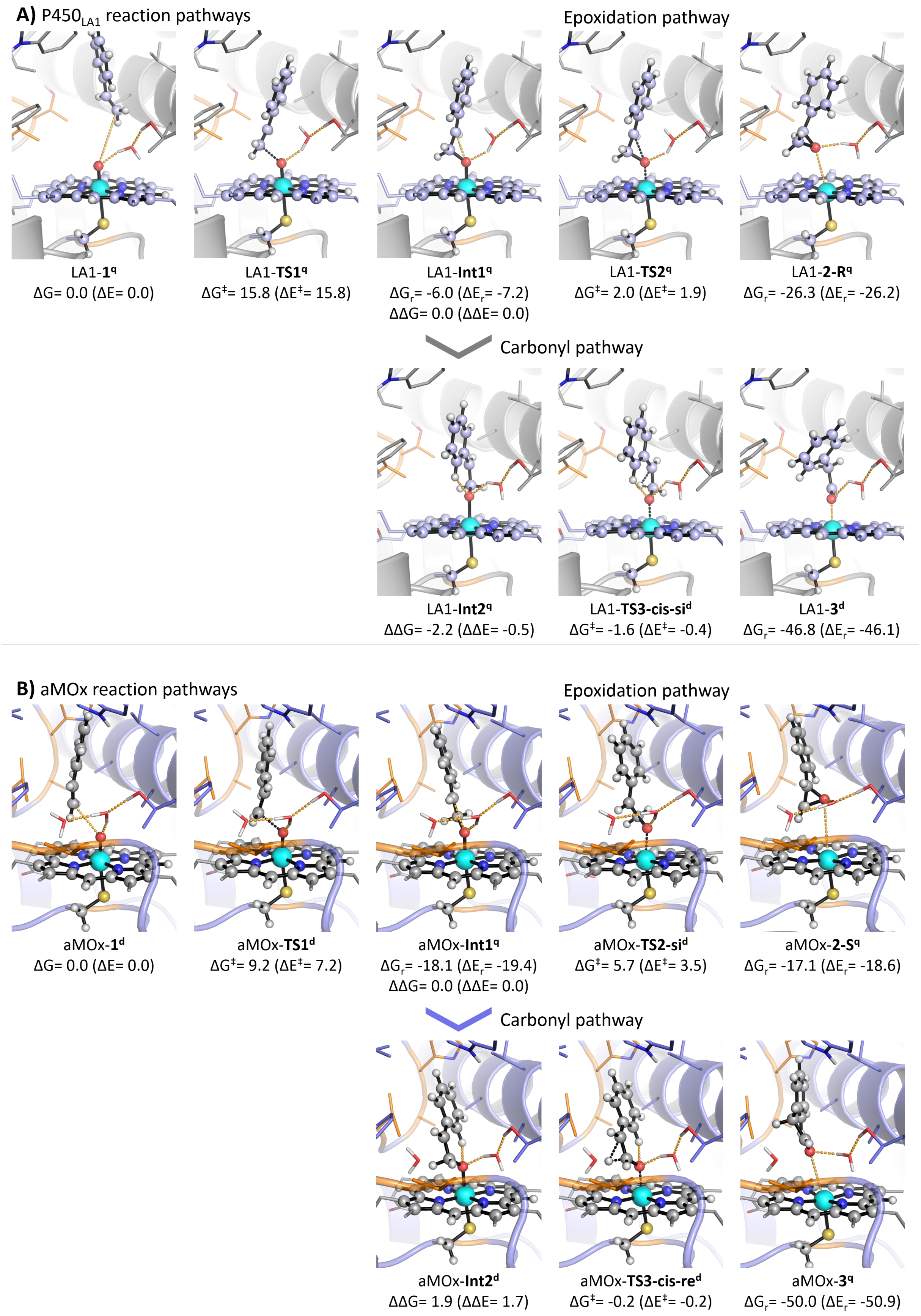

Table S2: Predicted lifetimes for the reactive radical intermediate from the enzyme-free DFT truncated model and QM/MM calculations, based on the Transition State Theory model. For comparison, the lifetimes of the radical intermediates estimated from QCT simulations are also reported. The equations used to obtain the statistic intermediate lifetime are also given.

DFT free-enzyme model	Energy barrier (ΔG‡)	Predicted lifetime (fs)
**Int1**^{q} to **TS2**^{q}	2.7	$1.1\cdot10^4$
**Int1**^{q} to **TS-rotation**^{q}	2.0	$3.3\cdot10^3$

QM/MM calculations	Energy barrier (ΔG‡)	Predicted lifetime (fs)
LA1-**Int1**^{q} to LA1-**TS2**^{q}	2.0	$3.3\cdot10^3$
aMOx-**Int1**^{q} to aMOx-**TS2**^{q}	10.7	$7.8\cdot10^9$
aMOx-**Int1**^{q} to aMOx-**TS-rotation**^{q}	7.2	$2.1\cdot10^7$
aMOx-**Int1**^{d} to aMOx-**TS2**^{d} *	5.7 (9.7 from aMOx-**Int1**^{q})	$1.7\cdot10^6$ ($1.4\cdot10^9$)
aMOx-**Int1**^{d} to aMOx-**TS-rotation**^{d} *	8.1 (12.1 from aMOx-**Int1**^{q})	$9.7\cdot10^7$ ($8.3\cdot10^{10}$)

* Higher in energy pathway. aMOx-**Int**^{d} is 4.0 kcal·mol^{-1} higher than aMOx-**Int**^{q}.

QCT simulations	Estimated lifetime (fs)
15 trajectories (d)	$9.2\cdot10^1$
11 trajectories (q)	$2.7\cdot10^2$

$$t_{1/2} = \frac{\ln(2)}{k} \quad (1)$$

Where:

$t_{1/2}$ stands for the half-life time of a first order reaction,
and k is the TST rate constant (as defined in eq. 2):

$$k = \frac{k_B T}{h} e^{-\Delta G^{\ddagger}/RT} \quad (2)$$

Where:

k_B is the Boltzmann constant ($1.380649\cdot10^{-23}$ J·K^{-1}·mol^{-1}),
T is the temperature at 298.15 K,
h is the Plank constant ($6.62607015\cdot10^{-34}$ J·s),
$\Delta G^{\ddagger}$ is the computed free energy barrier in kcal·mol^{-1}, and
R is the ideal gas constant ($1.987204259\cdot10^{-3}$ kcal· K^{-1}·mol^{-1}).

Intermediate lifetime obtained from QCT simulations are calculated as the average value of the timegap measured between the formation of the first C1-O bond and the second C2-O bond in reactive, epoxide forming, QCT trajectories.

Nonstatistical intermediates and non-IRC reaction pathways are well established for a variety of organic reactions,[53–56] especially those involving post-transition state bifurcations.[57,58] However, they are still relatively unexplored in organometallic reactions.[59–66] Recent contributions in this field by Ess and coworkers[67] have highlighted the necessity to use direct dynamics trajectory QCT simulations to consider atomic motion in organometallic reactions involving shallow intermediates, to properly understand organometallic reaction mechanisms and selectivities. Nevertheless, and because the nonstatistical dynamic effects on organometallic intermediates and their potential evolution toward non-IRC pathways are highly system dependent (shape of the PES, weight of the atoms involved, geometric requirements, energy acquired by the intermediate when it is formed, etc.), it is not trivial and very challenging to propose a set of rules that allow us to predict, a priori, when these dynamic effects will determine the selectivity or the reaction mechanism. Further work in this direction is still required.

VI. Local Electric Field characterization in the active site cavity and its impact on the reactive intermediates

Figure S25: Estimation of the Local Electric Field (LEF) generated in enzyme active sites. The LEF in wildtype P450$_{LA1}$ and aMOx variant were calculated using TITAN 2.0, and using the QM/MM optimized key radical intermediates (**Int1**^{q}) (find more details in the Computational Methods section).

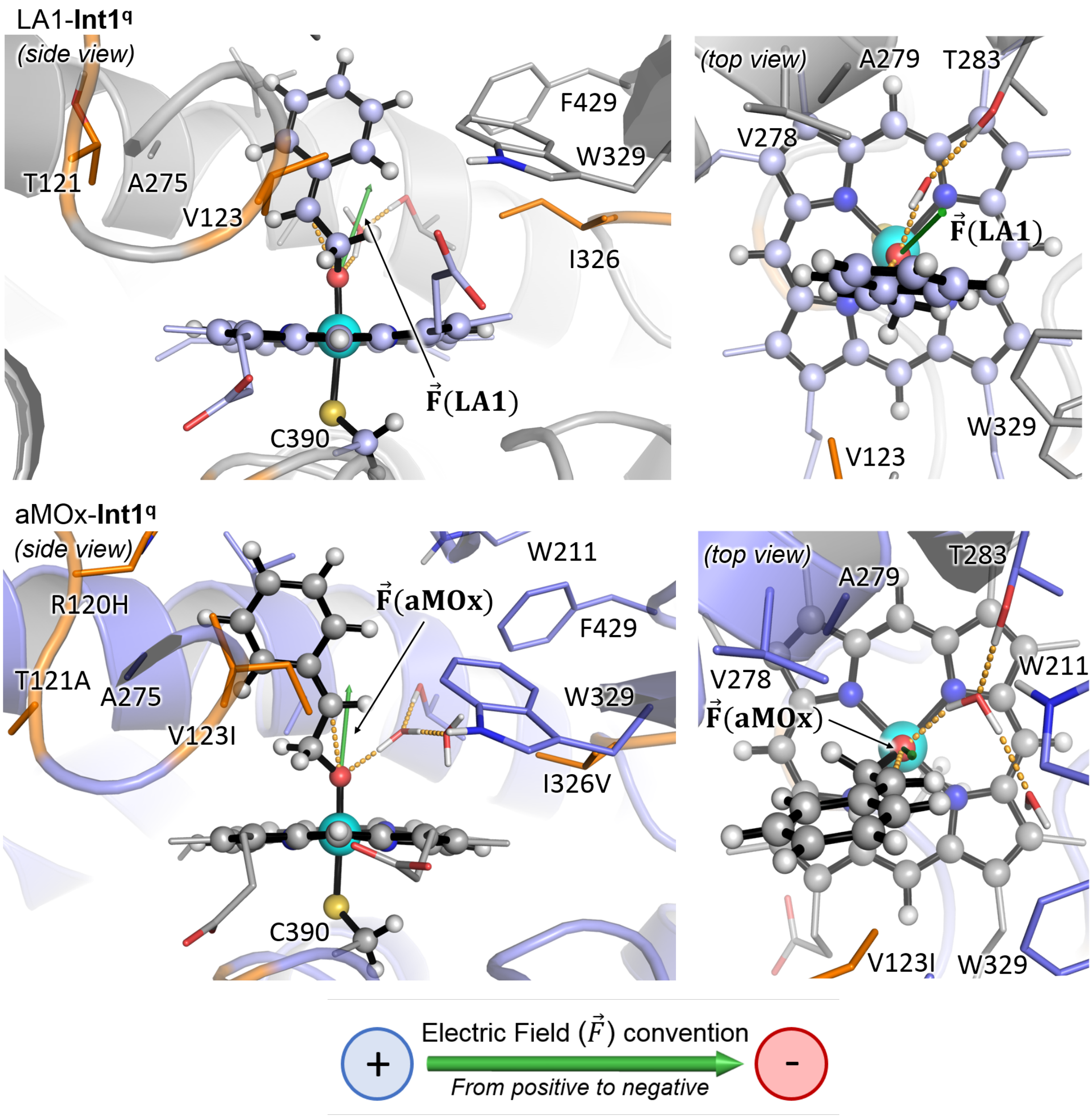

	Electric field components (a.u.)		
	F_x	F_y	F_z
$\vec{F}(LA1)$	0.00090	0.00470	0.00040
$\vec{F}(aMOx)$	-0.00020	0.00420	-0.00030

The electric field convention used is that the direction of the field is from positive to negative (i.e. a free positive charge will follow the direction of the electric vector). This is the general convention despite Gaussian package uses the opposite (i.e. negative to positive).

Interestingly, calculations showed that the local electric field (LEF) generated in the active sites of wildtype $P450_{LA1}$ ($\vec{F}(LA1)$) and aMOx variant ($\vec{F}(aMOx)$) are very similar, and almost equivalent, in both direction and strength.

$P450_{LA1}$ $\vec{F}(LA1)$ exhibits a large projection along the Fe-O bond despite the local electric field is not completely perpendicular to the heme cofactor. On the other hand, the local electric field generated in aMOx active site ($\vec{F}(aMOx)$) is almost perpendicular to the heme (see top view representations) and has a similar strength as the one generated in $P450_{LA1}$. In both cases, the LEF follows the Fe to O direction. Therefore, these calculations showed that evolution did not significantly altered the LEF exerted by the protein scaffold.

The direction of the LEF in the active site of these enzymes is relevant because it stabilizes the formation of the carbocation intermediate (see **Figures S26** to **S29**).

These results support the conclusion that the preexisting electrostatic preorganization of the wildtype $P450_{LA1}$ active site favors the formation of the carbocation intermediate. This electrostatic preorganization is required, together with the strong conformational control achieved by the aMOx variant active site upon evolution, to direct the reaction toward the carbonyl formation pathway.

Figure S26: Impact of the local electric field characterized in aMOx active site (**Figure S25**) on stabilization of key intermediates.

A) DFT optimized quartet radical intermediate (**Int1^q**) and doublet and quartet carbocation intermediates (**Int2^d** and **Int2^q**) in the absence and in the presence of an oriented electric field (EF) in terms of electronic energy (ΔE), enthalpy (ΔH), and quasi-harmonic corrected Gibbs energy (ΔG). Energy values were obtained at the (U)B3LYP/Def2TZVP/PCM (dichloromethane)//(U)B3LYP/631G(d)+SDD(Fe)/PCM (dichloromethane) level. Radical intermediate **Int1^q** in the absence of EF was used as energy reference. An EF strength equivalent to the one generated by the aMOx active site ($\vec{F}(aMOx) = (F_x, F_y, F_z) = (-0.00020, 0.00420, -0.00030)$ a.u., **Figure S25**) has been used in the calculations.

B) Optimized geometries for the stationary points reported in **A**). Mulliken charges (*q*) and spin densities (ρ) for the phenyl group (sum of all C and H atoms), C2 benzylic position, C1, O and Fe are reported.

Distances, energies and Mulliken charges and spin density values are given in Å, kcal·mol^{-1} and a.u., respectively.

A)

Structure	Electronic State	Electric Field	ΔΔE	ΔΔH	ΔΔG
**Int1** [a]	quartet (q)	No field	0.0	0.0	0.0
**Int2** [a]	doublet (d)	No field	-4.2	-4.7	-3.8
	quartet (q)	No field	3.7	2.6	0.8
**Int1-EF**	quartet (q)	$\vec{F}(aMOx)$	-6.9	-6.9	-7.0
**Int2-EF**	doublet (d)	$\vec{F}(aMOx)$	-25.2	-25.7	-24.8
	quartet (q)	$\vec{F}(aMOx)$	-19.1	-20.2	-21.9

[a] From **Figure S1**

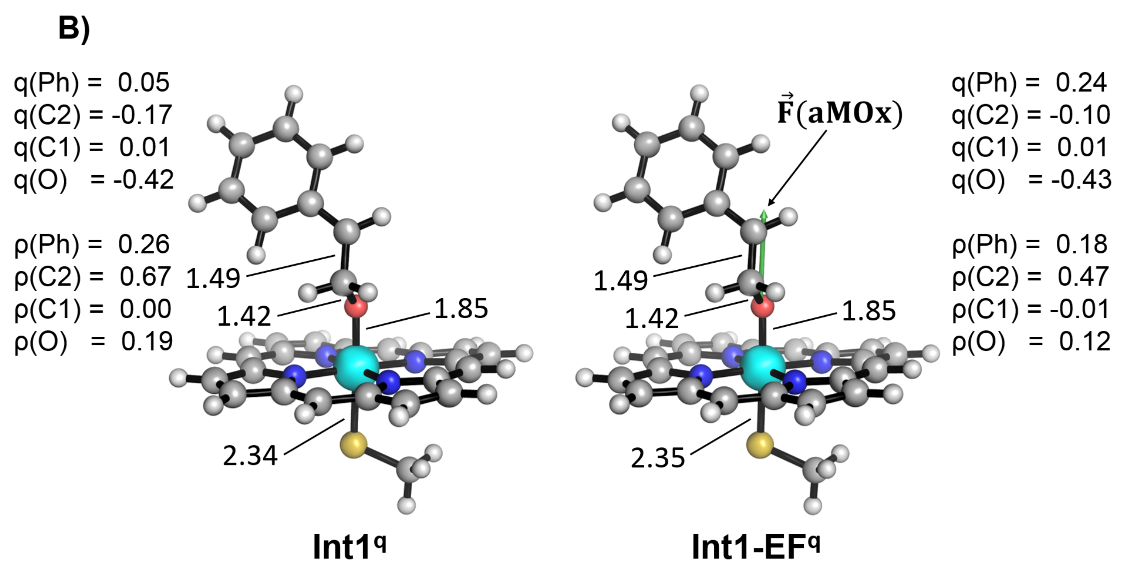

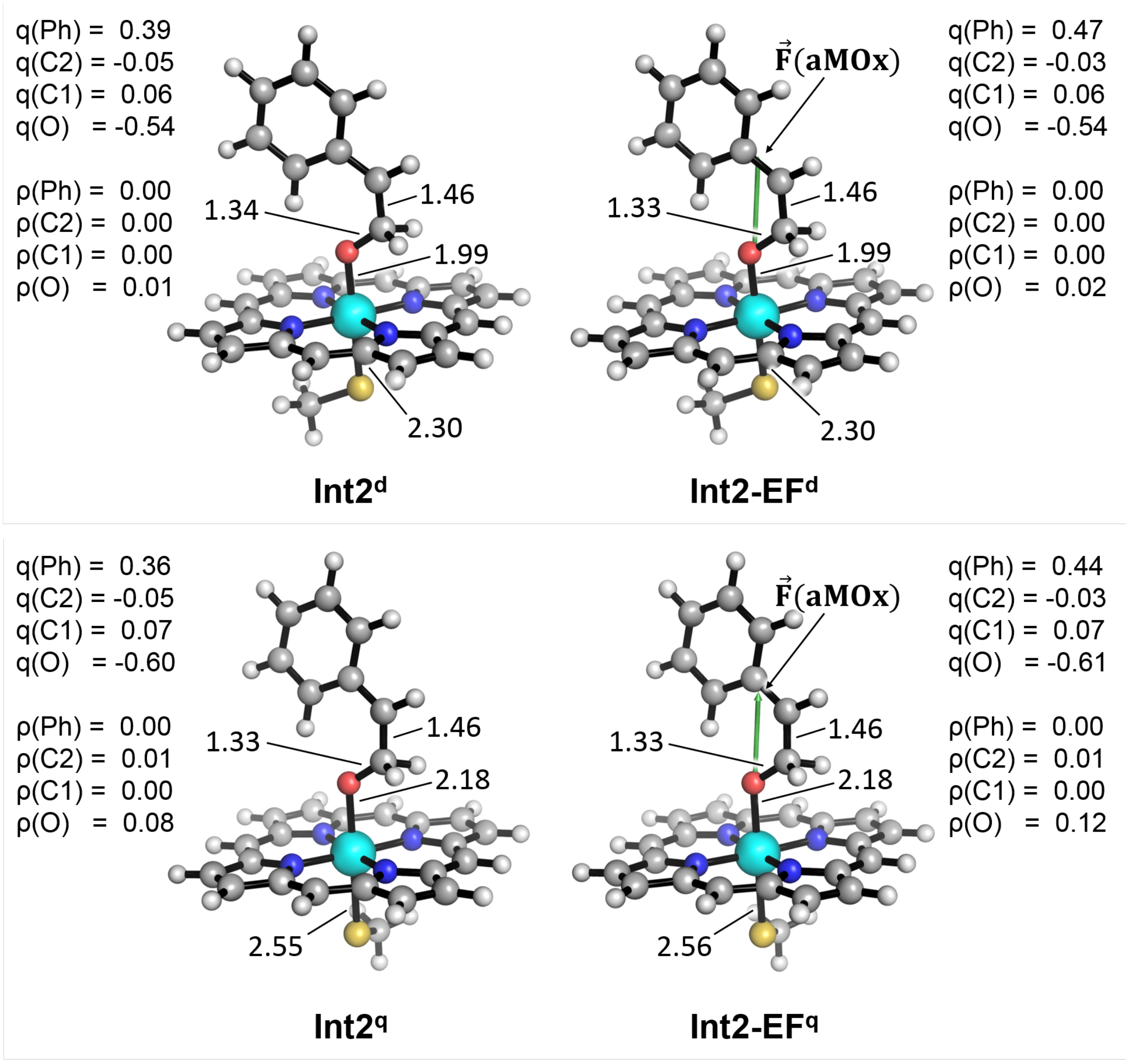

Reoptimization of the reactive intermediates in the presence of an oriented electric field with the same intensity and direction than $\vec{F}(aMOx)$ lead to almost identical geometries. Nevertheless, there are important differences regarding their stabilization.

Both radical (**Int1**) and carbocation (**Int2**) intermediates are stabilized by the presence of the electric field. However, carbocation **Int2** is significantly more stabilized than **Int1**. This stronger stabilization is induced by the orientation and direction of the electric field (oriented almost parallel to the Fe-O bond, following the Fe to O direction), which favors the accumulation of positive charge on the aromatic ring of the carbocation intermediate.

Equivalent conclusions are obtained from DFT calculations without considering the polarization effects introduced by the use of the implicit solvation model (see **Figure S27**).

In summary, calculations indicated that the generated LEF in aMOx (and $P450_{LA1}$) active site due to electrostatic preorganization largely stabilizes the carbocation **Int2** as compared to the radical intermediate **Int1**, favoring the formation of the carbocation intermediate in the enzymatic framework.

Figure S27: Impact of the local electric field characterized in aMOx active site (**Figure S25**) on stabilization of key intermediates, without considering polarization by implicit solvent model.

A) DFT optimized quartet radical intermediate (**Int1^q**) and doublet and quartet carbocation intermediates (**Int2^d** and **Int2^q**) in gas phase (no implicit solvent model) and in the presence of an oriented electric field (EF) in terms of electronic energy (ΔE), enthalpy (ΔH), and quasi-harmonic corrected Gibbs energy (ΔG). Energy values were obtained at the (U)B3LYP/Def2TZVP//(U)B3LYP/6-31G(d)+SDD(Fe) level. Radical intermediate **Int1^q** optimized in the gas phase and in the absence of EF was used as energy reference. An EF strength equivalent to the one generated by the aMOx active site ($\vec{F}(aMOx) = (F_x, F_y, F_z) = (-0.00020, 0.0420, -0.00030)$ a.u., **Figure S25**) has been used in the calculations.

B) Optimized geometries for the stationary points reported in **A**). Mulliken charges (*q*) and spin densities (ρ) for the phenyl group (sum of all C and H atoms), C2 benzylic position, C1, O and Fe are reported.

Distances, energies and Mulliken charges and spin density values are given in Å, kcal·mol^{-1} and a.u., respectively.

A)

Structure	Electronic State	Electric Field	ΔΔE	ΔΔH	ΔΔG
**Int1** (gas phase)	quartet (q)	No field	0.0	0.0	0.0
**Int2** (gas phase)	doublet (d)	No field	0.6	-0.5	0.2
**Int1-EF** (gas phase)	quartet (q)	$\vec{F}(aMOx)$	-3.3	-3.3	-3.3
**Int2-EF** (gas phase)	doublet (d)	$\vec{F}(aMOx)$	-12.6	-13.7	-13.0
	quartet (q)	$\vec{F}(aMOx)$	0.6	-0.6	-3.6

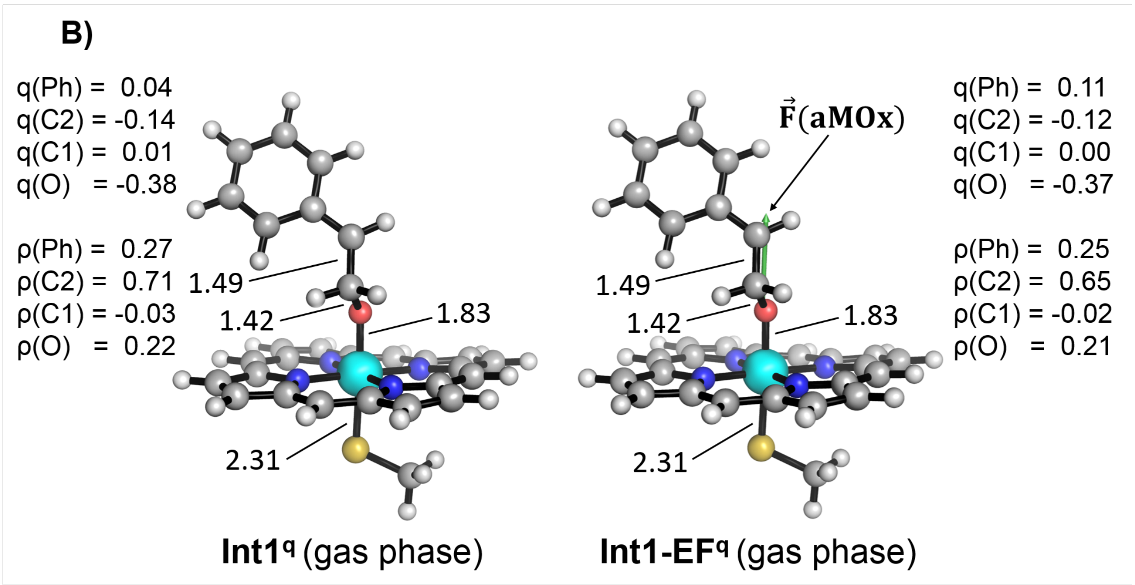

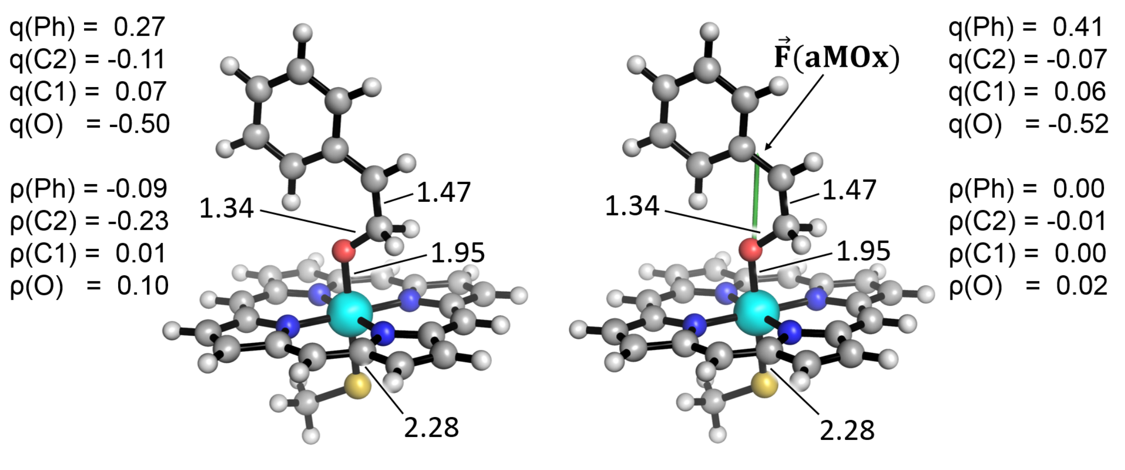

Int2^{d} (gas phase) **Int2-EF^{d}** (gas phase)

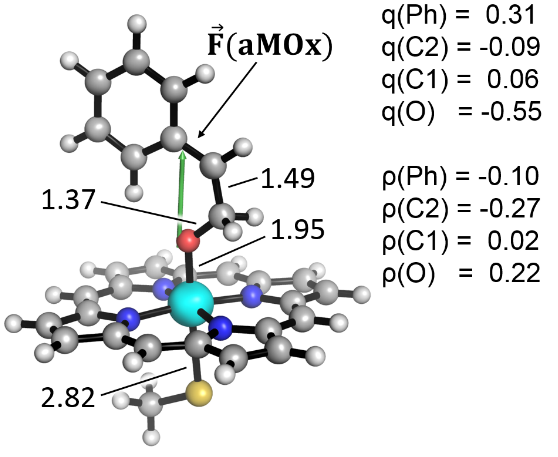

Int2-EF^{q} (gas phase)

Figure S28: Study of the impact of the local electric field strength on the stabilization of key intermediates.

A) Electric field components (in a.u.) of different electric fields with the same orientation and direction than $\vec{F}(aMOx)$, but with different intensities that have been used.

Calculations considering polarization from implicit solvent model:

B) Single point calculations at the (U)B3LYP/Def2TZVP/PCM(dichloromethane) level in the presence of oriented electric fields as described in **A)**. The DFT optimized structures of the radical and carbocation intermediates in the absence of electric field have been used, respectively. ΔΔE electronic energies are given in kcal·mol^{-1}. Radical intermediate **Int1^{q}** in the absence of EF was used as energy reference.

C) Mulliken charges and spin density values (in a.u.) obtained from **Int1^{q}** single point calculations with the different electric fields studied. A figure showing the orientation and direction of the applied electric fields is also shown.

D) Mulliken charges and spin density values (in a.u.) obtained from **Int2^{d}** single point calculations with the different electric fields studied. A figure showing the orientation and direction of the applied electric fields is also shown.

E) Mulliken charges and spin density values (in a.u.) obtained from **Int2^{q}** single point calculations with the different electric fields studied. A figure showing the orientation and direction of the applied electric fields is also shown.

Gas phase calculations:

F) Single point calculations at the (U)B3LYP/Def2TZVP level (gas phase) in the presence of oriented electric fields as described in **A)**. The DFT optimized structures of the radical and carbocation intermediates in the gas phase and in the absence of electric field have been used, respectively. ΔΔE electronic energies are given in kcal·mol^{-1}. Radical intermediate **Int1^{q}** (gas phase) in the absence of EF was used as energy reference.

G) Mulliken charges and spin density values (in a.u.) obtained from **Int1^{q}** (gas phase) single point calculations with the different electric fields studied. A figure showing the orientation and direction of the applied electric fields is also shown.

H) Mulliken charges and spin density values (in a.u.) obtained from **Int2^{d}** (gas phase) single point calculations with the different electric fields studied. A figure showing the orientation and direction of the applied electric fields is also shown.

A)

Electric field intensity	Electric field components (a.u.)		
	F_x	F_y	F_z
$\vec{F}(aMOx)$ x2	-0.00040	0.00840	-0.00060
$\vec{F}(aMOx)$ x1	-0.00020	0.00420	-0.00030
$\vec{F}(aMOx)$ x0.5	-0.00010	0.00210	-0.00015
No field	0.00000	0.00000	0.00000
- $\vec{F}(aMOx)$ x0.5	0.00010	-0.00210	0.00015
- $\vec{F}(aMOx)$ x1	0.00020	-0.00420	0.00030
- $\vec{F}(aMOx)$ x2	0.00040	-0.00840	0.00060

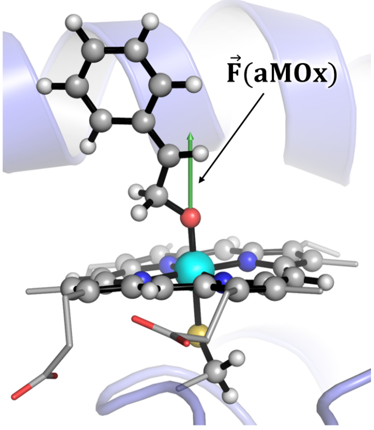

aMOx-**Int1q**

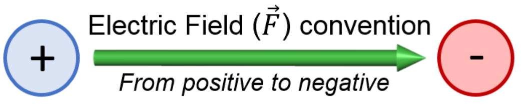

The electric field convention is that the direction of the field is from positive to negative (i.e. a free positive charge will follow the direction of the electric vector). This is the general convention despite Gaussian package uses the opposite (i.e. negative to positive).

Calculations considering polarization from implicit solvent model:

B)

Electric field intensity	ΔΔE (kcal·mol^{-1})		
	Int1q	Int2d	Int2q
$\vec{F}(aMOx)$ x2	-31.2	-52.7	-48.5
$\vec{F}(aMOx)$ x1	-6.8	-25.0	-19.0
$\vec{F}(aMOx)$ x0.5	-1.9	-13.7	-6.8
No field	0.0	-4.2	3.7
- $\vec{F}(aMOx)$ x0.5	-0.2	3.5	12.2
- $\vec{F}(aMOx)$ x1	-2.2	14.8	7.6
- $\vec{F}(aMOx)$ x2	-12.6	-11.6	2.9

Values highlighted in gray indicate a change in the electronic configuration as compared to the electronic configuration in the absence of electric field. For example: a radical configuration when a carbocation structure is expected.

C)

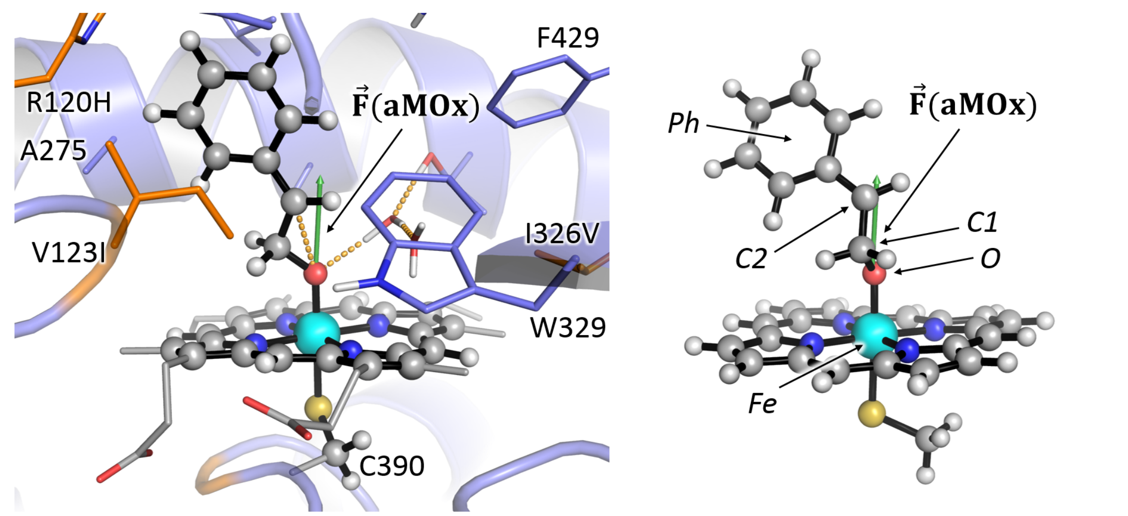

aMOx-**Int1q** **Int1q**

Electric field intensity	Int1q Mulliken Charges (q)				
	Ph	C2	C1	O	Fe
$\vec{F}(aMOx)$ x2	0.54	0.00	0.01	-0.48	-0.03
$\vec{F}(aMOx)$ x1	0.24	-0.11	0.01	-0.42	-0.14
$\vec{F}(aMOx)$ x0.5	0.10	-0.15	0.01	-0.41	-0.18
No field	0.05	-0.17	0.01	-0.42	-0.20
- $\vec{F}(aMOx)$ x0.5	0.01	-0.17	0.02	-0.43	-0.21
- $\vec{F}(aMOx)$ x1	-0.01	-0.18	0.02	-0.45	-0.22
- $\vec{F}(aMOx)$ x2	-0.08	-0.20	0.04	-0.47	-0.21

Electric field intensity	Int1q Spin Densities (ρ)				
	Ph	C2	C1	O	Fe
$\vec{F}(aMOx)$ x2	0.06	0.09	0.00	0.20	2.55
$\vec{F}(aMOx)$ x1	0.18	0.48	-0.01	0.12	2.26
$\vec{F}(aMOx)$ x0.5	0.24	0.66	-0.01	0.17	2.06
No field	0.26	0.67	0.00	-0.19	1.99
- $\vec{F}(aMOx)$ x0.5	0.27	0.67	0.00	0.19	1.97
- $\vec{F}(aMOx)$ x1	0.27	0.67	0.00	0.19	1.96
- $\vec{F}(aMOx)$ x2	0.28	0.69	-0.04	0.13	1.20

Values highlighted in gray indicate a change in the electronic configuration as compared to the electronic configuration in the absence of electric field. For example: a radical configuration when a carbocation structure is expected.

D)

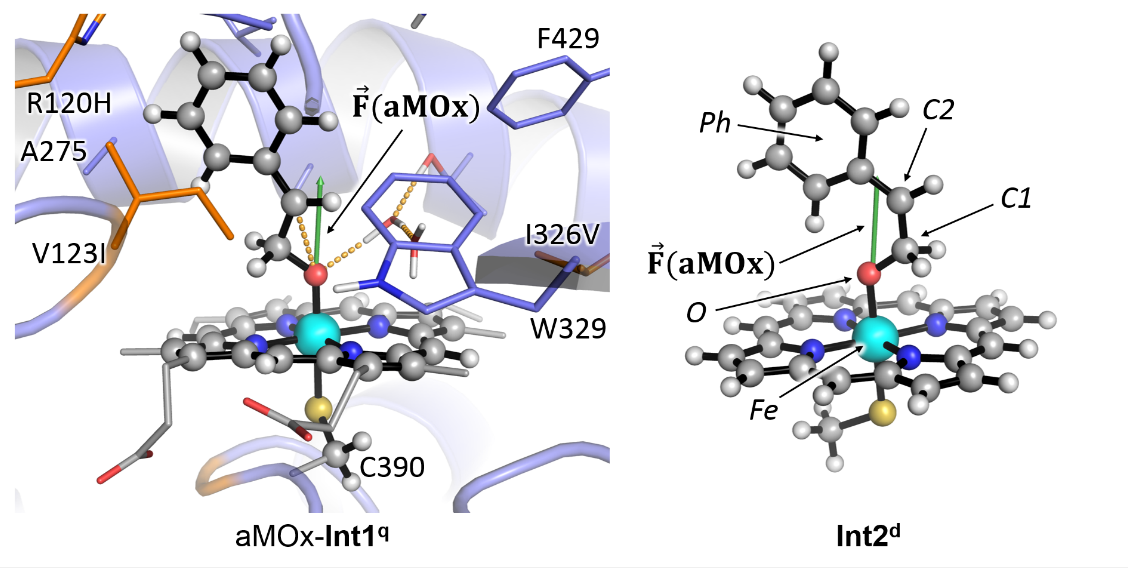

Electric field intensity	Int2d Mulliken Charges (q)				
	Ph	C2	C1	O	Fe
$\vec{F}(aMOx)$ x2	0.54	-0.03	0.06	-0.53	-0.06
$\vec{F}(aMOx)$ x1	0.47	-0.03	0.06	-0.54	-0.07
$\vec{F}(aMOx)$ x0.5	0.43	-0.04	0.06	-0.54	-0.08
No field	0.39	-0.05	0.06	-0.54	-0.08
- $\vec{F}(aMOx)$ x0.5	0.32	-0.07	0.06	-0.53	-0.09
- $\vec{F}(aMOx)$ x1	0.17	-0.13	0.06	-0.52	-0.09
- $\vec{F}(aMOx)$ x2	-0.18	-0.20	0.09	-0.55	-0.13

Electric field intensity	Int2d Spin Densities (ρ)				
	Ph	C2	C1	O	Fe
$\vec{F}(aMOx)$ x2	0.00	0.00	0.00	0.04	1.03
$\vec{F}(aMOx)$ x1	0.00	0.00	0.00	0.02	1.04
$\vec{F}(aMOx)$ x0.5	0.00	0.01	0.00	0.02	1.04
No field	0.00	0.00	0.00	0.01	1.03
- $\vec{F}(aMOx)$ x0.5	-0.02	-0.06	0.00	0.03	1.01
- $\vec{F}(aMOx)$ x1	0.02	0.01	0.00	0.02	1.23
- $\vec{F}(aMOx)$ x2	0.36	0.57	-0.04	0.05	1.09

Values highlighted in gray indicate a change in the electronic configuration as compared to the electronic configuration in the absence of electric field. For example: a radical configuration when a carbocation structure is expected.

E)

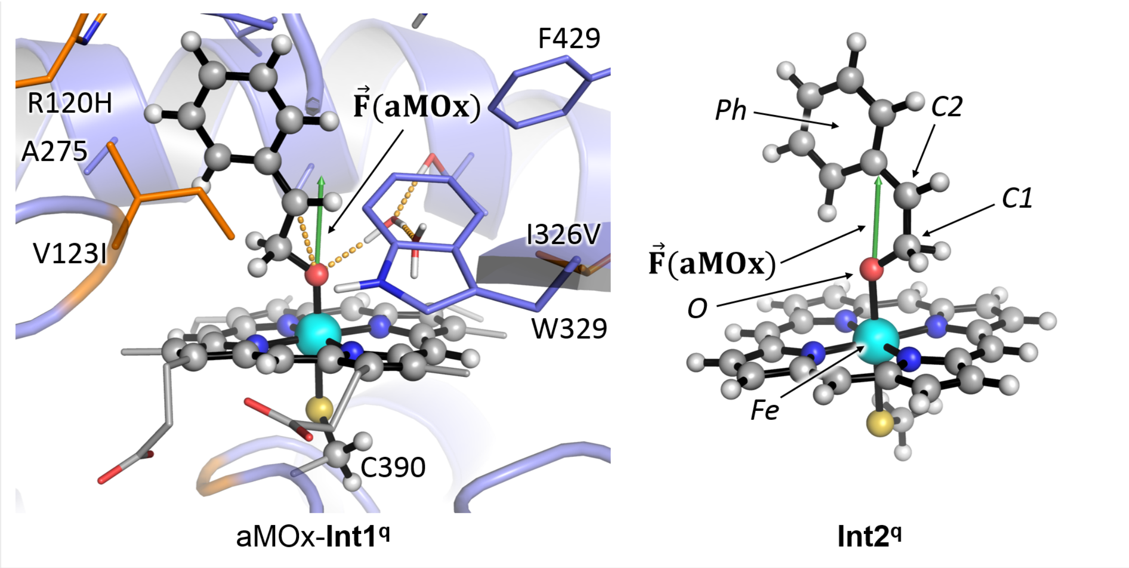

Electric field intensity	Int2q Mulliken Charges (q)				
	Ph	C2	C1	O	Fe
$\vec{F}(aMOx)$ x2	0.51	-0.02	0.07	-0.60	0.10
$\vec{F}(aMOx)$ $x1$	0.44	-0.03	0.07	-0.61	0.09
$\vec{F}(aMOx)$ x0.5	0.40	-0.04	0.07	-0.61	0.08
No field	0.36	-0.05	0.07	-0.60	0.07
- $\vec{F}(aMOx)$ x0.5	0.20	-0.10	0.08	-0.59	0.03
- $\vec{F}(aMOx)$ x1	-0.14	-0.19	0.10	-0.60	-0.06
- $\vec{F}(aMOx)$ x2	-0.22	-0.20	0.10	-0.61	-0.05

Electric field intensity	Int2q Spin Densities (ρ)				
	Ph	C2	C1	O	Fe
$\vec{F}(aMOx)$ x2	0.00	0.01	-0.01	0.17	2.74
$\vec{F}(aMOx)$ $x1$	0.00	0.01	0.00	0.12	2.75
$\vec{F}(aMOx)$ x0.5	0.00	0.01	0.00	0.10	2.74
No field	0.00	0.01	0.00	0.08	2.72
- $\vec{F}(aMOx)$ x0.5	0.11	0.24	-0.01	0.01	2.69
- $\vec{F}(aMOx)$ x1	0.34	0.61	-0.05	0.16	2.63
- $\vec{F}(aMOx)$ x2	0.36	0.53	-0.04	0.11	2.80

Values highlighted in gray indicate a change in the electronic configuration as compared to the electronic configuration in the absence of electric field. For example: a radical configuration when a carbocation structure is expected.

Gas phase calculations:

F)

**Electric field intensity**	**ΔΔE (kcal·mol^{-1})**	
	**Int1^q** (gas phase)	**Int2^d** (gas phase)
$\vec{F}(aMOx)$ x2	-13.6	-32.8
$\vec{F}(aMOx)$ $x1$	-3.3	-12.6
$\vec{F}(aMOx)$ x0.5	-0.9	-4.9
No field	0.0	0.6
- $\vec{F}(aMOx)$ x0.5	-0.4	3.3
- $\vec{F}(aMOx)$ x1	-2.1	2.4
- $\vec{F}(aMOx)$ x2	-9.3	-7.8

Values highlighted in gray indicate a change in the electronic configuration as compared to the electronic configuration in the absence of electric field. For example: a radical configuration when a carbocation structure is expected.

G)

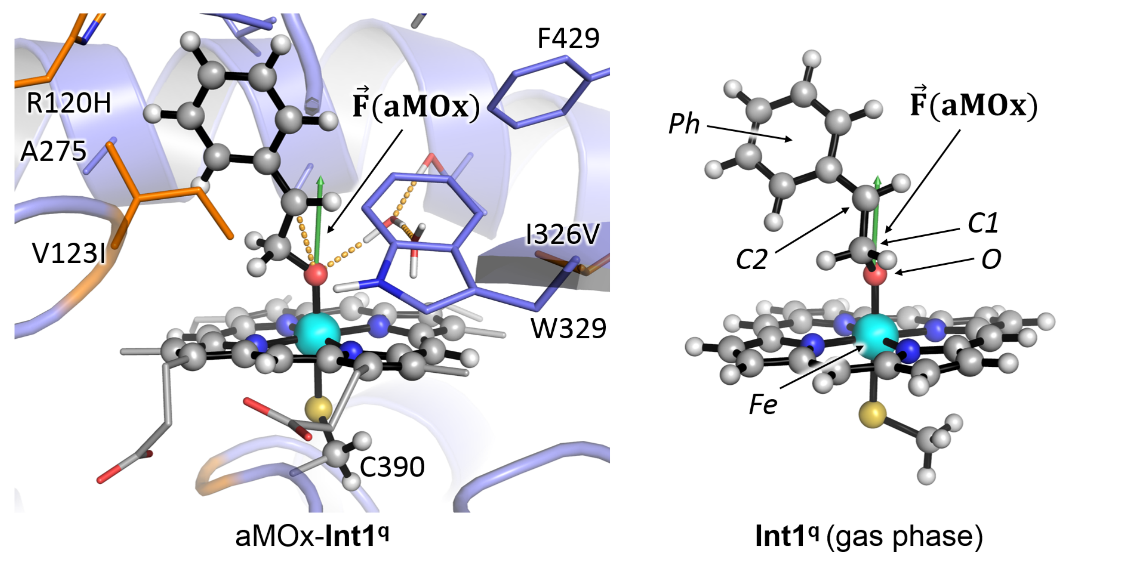

Electric field intensity	Int1q (gas phase) Mulliken Charges (q)				
	Ph	C2	C1	O	Fe
$\vec{F}(aMOx)$ x2	0.25	-0.09	0.00	-0.39	-0.17
$\vec{F}(aMOx)$ x1	0.11	-0.12	0.00	-0.37	-0.21
$\vec{F}(aMOx)$ x0.5	0.07	-0.13	0.00	-0.37	-0.22
No field	0.04	-0.14	0.01	-0.38	-0.23
- $\vec{F}(aMOx)$ x0.5	0.02	-0.14	0.01	-0.39	-0.23
- $\vec{F}(aMOx)$ x1	-0.01	-0.14	0.02	-0.40	-0.24
- $\vec{F}(aMOx)$ x2	-0.05	-0.14	0.03	-0.42	-0.25

Electric field intensity	Int1q (gas phase) Spin Densities (ρ)				
	Ph	C2	C1	O	Fe
$\vec{F}(aMOx)$ x2	0.19	0.48	-0.01	0.15	2.18
$\vec{F}(aMOx)$ x1	0.25	0.65	-0.02	0.21	1.96
$\vec{F}(aMOx)$ x0.5	0.26	0.69	-0.03	0.22	1.90
No field	0.27	0.71	-0.03	0.22	1.86
- $\vec{F}(aMOx)$ x0.5	0.27	0.72	-0.03	0.21	1.84
- $\vec{F}(aMOx)$ x1	0.28	0.72	-0.03	0.21	1.81
- $\vec{F}(aMOx)$ x2	0.28	0.71	-0.04	0.18	1.64

Values highlighted in gray indicate a change in the electronic configuration as compared to the electronic configuration in the absence of electric field. For example: a radical configuration when a carbocation structure is expected.

H)

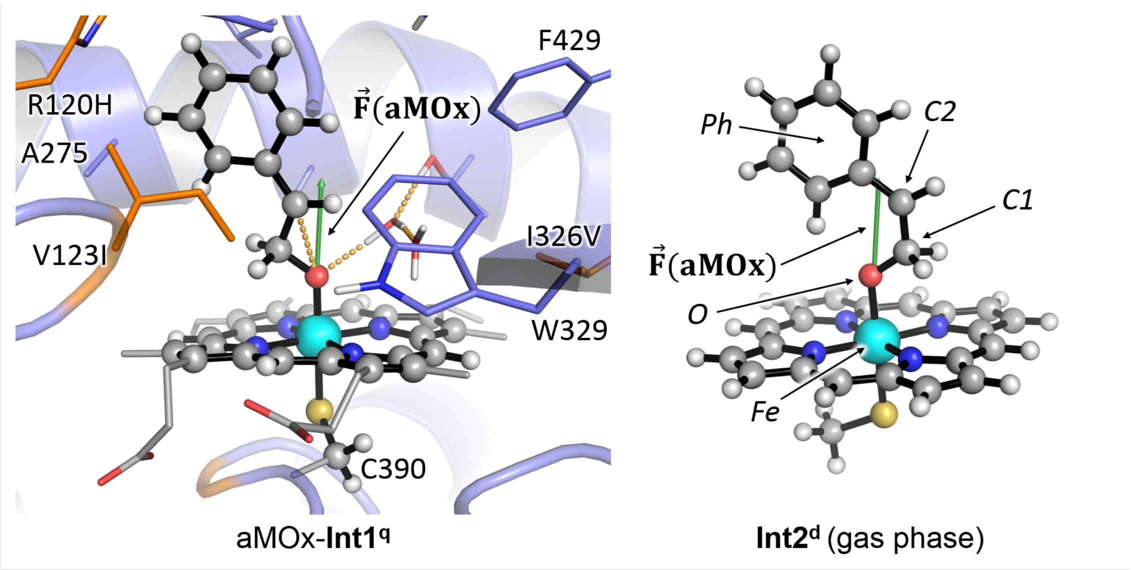

**Electric field intensity**	**Int2**[d] (gas phase) **Mulliken Charges (q)**				
	Ph	C2	C1	O	Fe
$\vec{F}(aMOx)$ x2	0.48	-0.06	0.06	-0.52	-0.11
$\vec{F}(aMOx)$ x1	0.41	-0.07	0.06	-0.52	-0.11
$\vec{F}(aMOx)$ x0.5	0.36	-0.09	0.06	-0.51	-0.12
No field	0.27	-0.11	0.07	-0.50	-0.12
- $\vec{F}(aMOx)$ x0.5	0.18	-0.13	0.07	-0.50	-0.13
- $\vec{F}(aMOx)$ x1	-0.04	-0.19	0.09	-0.49	-0.18
- $\vec{F}(aMOx)$ x2	-0.09	-0.19	0.09	-0.51	-0.18

**Electric field intensity**	**Int2**[d] (gas phase) **Spin Densities (ρ)**				
	Ph	C2	C1	O	Fe
$\vec{F}(aMOx)$ x2	0.00	0.00	0.00	0.02	1.01
$\vec{F}(aMOx)$ x1	0.00	-0.01	0.00	0.02	0.99
$\vec{F}(aMOx)$ x0.5	-0.03	-0.09	0.00	0.05	0.97
No field	-0.09	-0.23	0.01	0.10	0.95
- $\vec{F}(aMOx)$ x0.5	-0.15	-0.36	0.02	0.12	0.93
- $\vec{F}(aMOx)$ x1	-0.31	-0.65	0.04	0.12	1.98
- $\vec{F}(aMOx)$ x2	-0.33	-0.63	0.04	0.08	2.03

Values highlighted in gray indicate a change in the electronic configuration as compared to the electronic configuration in the absence of electric field. For example: a radical configuration when a carbocation structure is expected.

Figure S29: Study of the impact of the local electric field direction on the stabilization of key intermediates.

A) Electric field components (in a.u.) of the different fields orthogonal to $\vec{F}(aMOx)$ but with the same strength that have been used:

Calculations considering polarization from implicit solvent model:

B) Single point calculations at the (U)B3LYP/Def2TZVP/PCM(dichloromethane) in the presence of oriented electric fields as described in **A**. The DFT optimized structures of the radical and carbocation intermediates in the absence of electric field have been used, respectively. ΔΔE electronic energies are given in kcal·mol^{-1}. Radical intermediate **Int1^{q}** in the absence of EF was used as energy reference.

C) Mulliken charges and spin density values (in a.u.) obtained from **Int1^{q}** single point calculations with the different fields orthogonal to $\vec{F}(aMOx)$ but with the same intensity. A figure showing the orientation and direction of the applied electric fields is also shown.

D) Mulliken charges and spin density values (in a.u.) obtained from **Int2^{d}** single point calculations with the different fields orthogonal to $\vec{F}(aMOx)$ but with the same intensity. A figure showing the orientation and direction of the applied electric fields is also shown.

E) Mulliken charges and spin density values (in a.u.) obtained from **Int2^{q}** single point calculations with the different fields orthogonal to $\vec{F}(aMOx)$ but with the same intensity. A figure showing the orientation and direction of the applied electric fields is also shown.

Gas Phase calculations:

F) Single point calculations at the (U)B3LYP/Def2TZVP level (gas phase) in the presence of oriented electric fields as described in **A.** The DFT optimized structures of the radical and carbocation intermediates in the gas phase and in the absence of electric field have been used, respectively. ΔΔE electronic energies are given in kcal·mol^{-1}. Radical intermediate **Int1^{q}** (gas phase) in the absence of EF was used as energy reference.

G) Mulliken charges and spin density values (in a.u.) obtained from **Int1^{q}** (gas phase) single point calculations with the different fields orthogonal to $\vec{F}(aMOx)$ but with the same intensity. A figure showing the orientation and direction of the applied electric fields is also shown.

H) Mulliken charges and spin density values (in a.u.) obtained from **Int2^{d}** (gas phase) single point calculations with the different fields orthogonal to $\vec{F}(aMOx)$ but with the same intensity. A figure showing the orientation and direction of the applied electric fields is also shown.

A)

Electric field directions	Electric field components (a.u.)		
	F_x	F_y	F_z
No field	0.00000	0.00000	0.00000
$\vec{F_i} = \vec{F}(aMOx)$	-0.00020	0.00420	-0.00030
$-\vec{F_i} = -\vec{F}(aMOx)$	0.00020	-0.00420	0.00030
$\vec{F_j}$	0.00421	0.00020	-0.00001
$-\vec{F_j}$	-0.00421	-0.00020	0.00001
$\vec{F_k}$	0.00000	0.00030	0.00420
$-\vec{F_k}$	0.00000	-0.00030	-0.00420

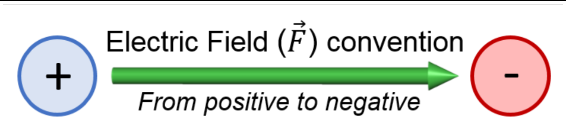

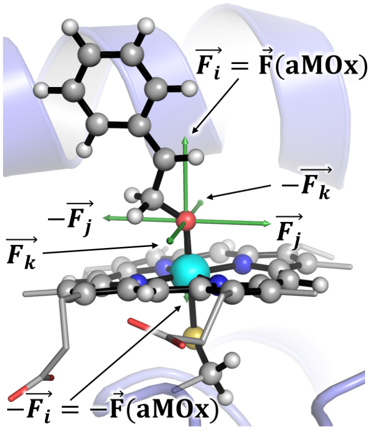

aMOx-**Int1**q

The electric field convention is that the direction of the field is from positive to negative (i.e. a free positive charge will follow the direction of the electric vector). This is the general convention despite Gaussian package uses the opposite (i.e. negative to positive).

Calculations considering polarization from implicit solvent model:

B)

Electric field directions	ΔΔE (kcal·mol^{-1})		
	Int1q	Int2d	Int2q
No field	0.0	-4.1	3.7
$\vec{F_i} = \vec{F}(aMOx)$	-6.7	-24.9	-18.9
$-\vec{F_i} = -\vec{F}(aMOx)$	-2.2	14.8	7.6
$\vec{F_j}$	-6.3	-6.8	-1.0
$-\vec{F_j}$	-4.2	-11.6	-1.7
$\vec{F_k}$	-5.5	-9.2	1.0
$-\vec{F_k}$	-3.7	-8.0	-3.0

Values highlighted in gray indicate a change in the electronic configuration as compared to the electronic configuration in the absence of electric field. For example: a radical configuration when a carbocation structure is expected.

C)

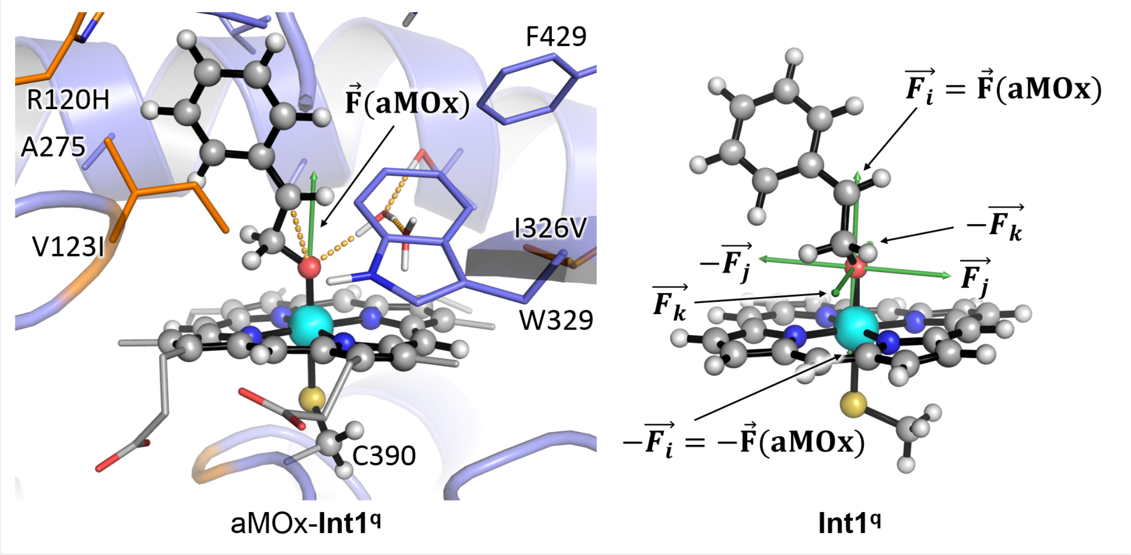

Electric field directions	Int1q Mulliken Charges (q)				
	Ph	C2	C1	O	Fe
No field	0.05	-0.17	0.01	-0.42	-0.20
$\vec{F_i} = \vec{F}(aMOx)$	0.24	-0.11	0.01	-0.42	-0.14
$-\vec{F_i} = -\vec{F}(aMOx)$	-0.01	-0.18	0.02	-0.45	-0.22
$\vec{F_j}$	-0.01	-0.16	0.01	-0.42	-0.20
$-\vec{F_j}$	0.12	-0.17	0.02	-0.42	-0.20
$\vec{F_k}$	0.03	-0.16	0.00	-0.43	-0.21
$-\vec{F_k}$	0.07	-0.18	0.02	-0.41	-0.19

Electric field directions	Int1q Spin Densities (ρ)				
	Ph	C2	C1	O	Fe
No field	0.26	0.67	0.00	0.19	1.99
$\vec{F_i} = \vec{F}(aMOx)$	0.18	0.48	-0.01	0.12	2.26
$-\vec{F_i} = -\vec{F}(aMOx)$	0.27	0.67	0.00	0.19	1.96
$\vec{F_j}$	0.27	0.68	0.00	0.20	1.97
$-\vec{F_j}$	0.25	0.67	0.00	0.17	2.03
$\vec{F_k}$	0.26	0.68	0.00	0.19	1.98
$-\vec{F_k}$	0.26	0.67	0.00	0.18	2.00

D)

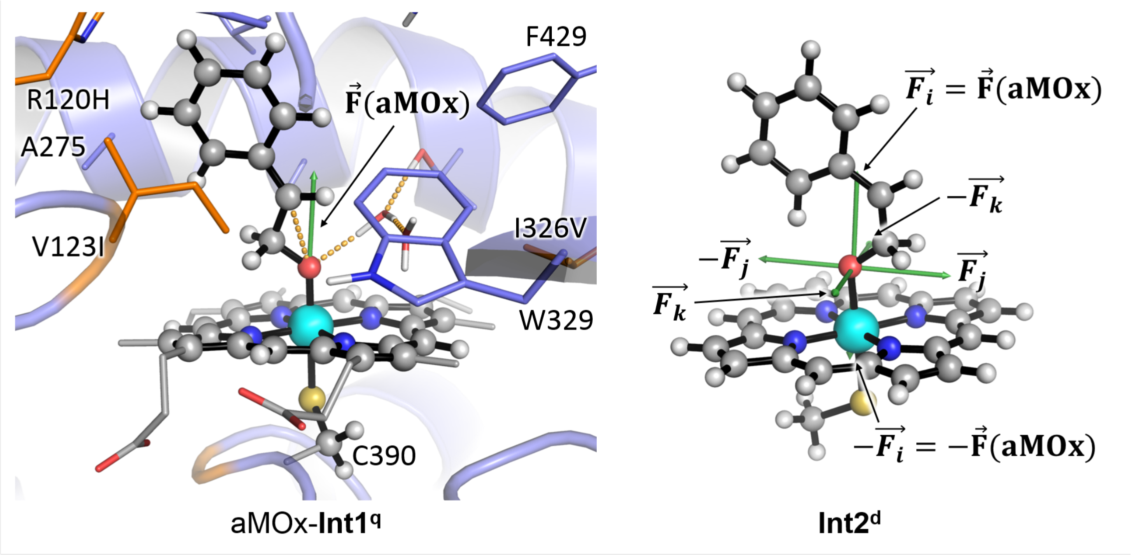

Electric field directions	Int2[d] Mulliken Charges (q)				
	Ph	C2	C1	O	Fe
No field	0.39	-0.05	0.06	-0.54	-0.08
$\vec{F_i} = \vec{F}(aMOx)$	0.47	-0.03	0.06	-0.54	-0.07
$-\vec{F_i} = -\vec{F}(aMOx)$	0.17	-0.13	0.06	-0.52	-0.09
$\vec{F_j}$	0.33	-0.05	0.05	-0.54	-0.08
$-\vec{F_j}$	0.45	-0.04	0.07	-0.53	-0.08
$\vec{F_k}$	0.39	-0.05	0.06	-0.54	-0.08
$-\vec{F_k}$	0.39	-0.05	0.06	-0.54	-0.08

Electric field directions	Int2[d] Spin Densities (ρ)				
	Ph	C2	C1	O	Fe
No field	0.00	0.00	0.00	0.01	1.03
$\vec{F_i} = \vec{F}(aMOx)$	0.00	0.00	0.00	0.02	1.04
$-\vec{F_i} = -\vec{F}(aMOx)$	0.02	0.01	0.00	0.02	1.23
$\vec{F_j}$	0.00	0.00	0.00	0.01	1.03
$-\vec{F_j}$	0.00	0.00	0.00	0.01	1.03
$\vec{F_k}$	0.00	0.00	0.00	0.01	1.03
$-\vec{F_k}$	0.00	0.00	0.00	0.01	1.03

E)

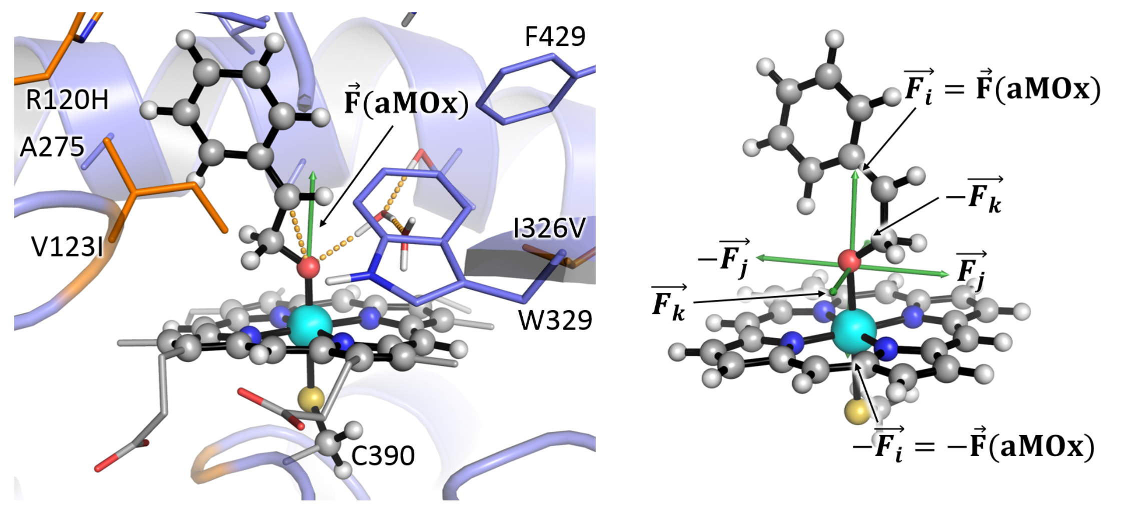

aMOx-**Int1**q **Int2**q

Electric field directions	Int2q Mulliken Charges (q)				
	Ph	C2	C1	O	Fe
No field	0.36	-0.05	0.07	-0.60	0.07
$\vec{F_i} = \vec{F}(aMOx)$	0.44	-0.03	0.07	-0.61	0.09
$-\vec{F_i} = -\vec{F}(aMOx)$	-0.14	-0.19	0.10	-0.60	-0.06
$\vec{F_j}$	0.31	-0.05	0.06	-0.60	0.07
$-\vec{F_j}$	0.41	-0.04	0.08	-0.59	0.07
$\vec{F_k}$	0.34	-0.05	0.07	-0.60	0.07
$-\vec{F_k}$	0.38	-0.05	0.07	-0.60	0.08

Electric field directions	Int2q Spin Densities (ρ)				
	Ph	C2	C1	O	Fe
No field	0.00	0.01	0.00	0.08	2.72
$\vec{F_i} = \vec{F}(aMOx)$	0.00	0.01	0.00	0.12	2.75
$-\vec{F_i} = -\vec{F}(aMOx)$	0.34	0.61	-0.05	0.16	2.62
$\vec{F_j}$	0.00	0.01	0.00	0.07	2.72
$-\vec{F_j}$	0.00	0.00	0.00	0.08	2.73
$\vec{F_k}$	0.00	0.00	0.00	0.08	2.73
$-\vec{F_k}$	0.00	0.01	0.00	0.08	2.72

Values highlighted in gray indicate a change in the electronic configuration as compared to the electronic configuration in the absence of electric field. For example: a radical configuration when a carbocation structure is expected.

Gas phase calculations:

F)

Electric field directions	ΔΔE ($kcal \cdot mol^{-1}$)	
	**Int1^{q}** (gas phase)	**Int2^{d}** (gas phase)
No field	0.0	0.6
$\vec{F_i} = \vec{F}(aMOx)$	-3.3	-12.6
$-\vec{F_i} = -\vec{F}(aMOx)$	-2.1	2.4
$\vec{F_j}$	-2.5	-3.8
$-\vec{F_j}$	-4.1	-1.4
$\vec{F_k}$	-2.5	-2.0
$-\vec{F_k}$	-3.3	-2.5

Values highlighted in gray indicate a change in the electronic configuration as compared to the electronic configuration in the absence of electric field. For example: a radical configuration when a carbocation structure is expected.

G)

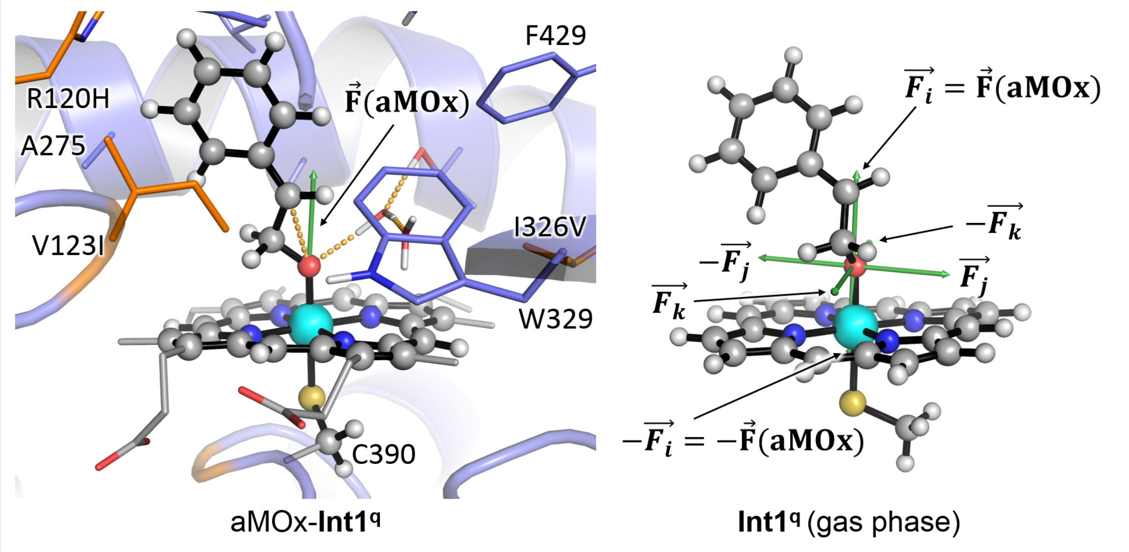

**Electric field directions**	**Int1q** (gas phase) **Mulliken Charges (q)**				
	Ph	C2	C1	O	Fe
No field	0.04	-0.14	0.01	-0.38	-0.23
$\vec{F_i} = \vec{F}(aMOx)$	0.11	-0.12	0.00	-0.37	-0.21
$-\vec{F_i} = -\vec{F}(aMOx)$	-0.01	-0.14	0.02	-0.40	-0.24
$\vec{F_j}$	0.08	-0.14	0.01	-0.38	-0.23
$-\vec{F_j}$	0.00	-0.13	0.00	-0.39	-0.23
$\vec{F_k}$	0.05	-0.14	0.02	-0.38	-0.22
$-\vec{F_k}$	0.03	-0.13	0.00	-0.39	-0.23

**Electric field directions**	**Intq** (gas phase) **Spin Densities (ρ)**				
	Ph	C2	C1	O	Fe
No field	0.27	0.71	-0.03	0.22	1.86
$\vec{F_i} = \vec{F}(aMOx)$	0.25	0.65	-0.02	0.21	1.96
$-\vec{F_i} = -\vec{F}(aMOx)$	0.28	0.72	-0.03	0.21	1.81
$\vec{F_j}$	0.26	0.70	-0.03	0.22	1.88
$-\vec{F_j}$	0.27	0.71	-0.03	0.22	1.85
$\vec{F_k}$	0.27	0.71	-0.03	0.22	1.87
$-\vec{F_k}$	0.27	0.71	-0.03	0.22	1.85

H)

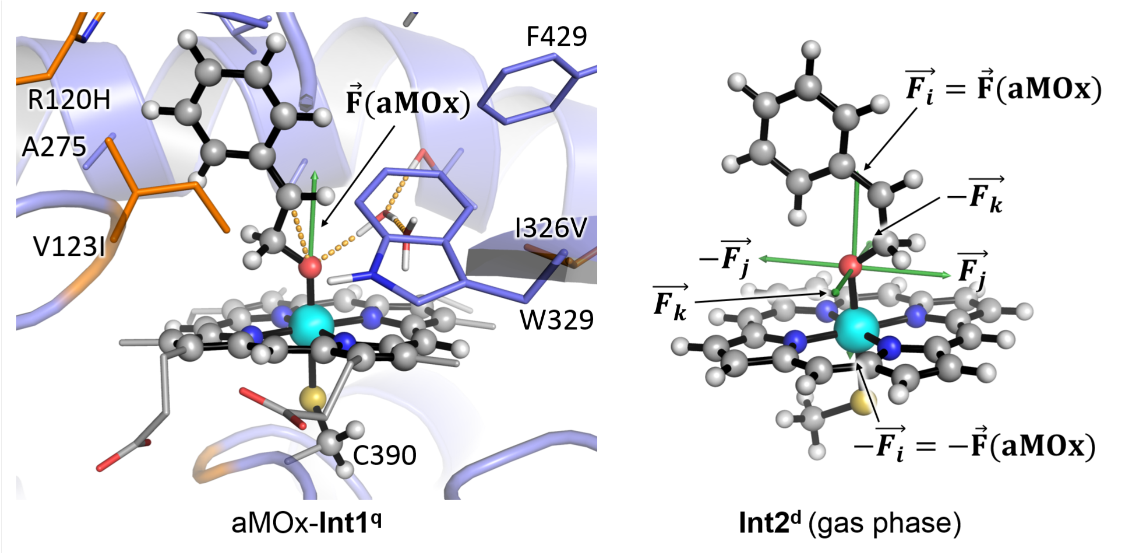

Electric field directions	Int2^d (gas phase) Mulliken Charges (q)				
	Ph	C2	C1	O	Fe
No field	0.27	-0.11	0.07	-0.50	-0.12
$\vec{F_i} = \vec{F}(aMOx)$	0.41	-0.07	0.06	-0.52	-0.11
$-\vec{F_i} = -\vec{F}(aMOx)$	-0.04	-0.19	0.09	-0.49	-0.18
$\vec{F_j}$	0.32	-0.10	0.08	-0.50	-0.12
$-\vec{F_j}$	0.22	-0.12	0.06	-0.50	-0.13
$\vec{F_k}$	0.27	-0.11	0.07	-0.50	-0.12
$-\vec{F_k}$	0.27	-0.11	0.07	-0.50	-0.12

Electric field directions	Int2^d (gas phase) Spin Densities (ρ)				
	Ph	C2	C1	O	Fe
No field	-0.09	-0.23	0.01	0.10	0.95
$\vec{F_i} = \vec{F}(aMOx)$	0.00	-0.01	0.00	0.02	0.99
$-\vec{F_i} = -\vec{F}(aMOx)$	-0.31	-0.65	0.04	0.12	1.98
$\vec{F_j}$	-0.07	-0.20	0.01	0.08	0.95
$-\vec{F_j}$	-0.11	-0.27	0.01	0.10	0.94
$\vec{F_k}$	-0.10	-0.24	0.01	0.10	0.95
$-\vec{F_k}$	-0.09	-0.23	0.01	0.09	0.95

Values highlighted in gray indicate a change in the electronic configuration as compared to the electronic configuration in the absence of electric field. For example: a radical configuration when a carbocation structure is expected.

DFT calculations using the computational truncated model have been carried out in order to assess the impact that the LEF generated by the protein has on the stabilization of the key intermediates involved in the studied reactions. Calculations showed that generated LEF in aMOx active site has the suitable orientation, direction and strength to favor the formation and stabilization of the carbocation **Int2** (**Figures S25**, **S28** and **S29**). Other orientations and directions of the electric field would lead to much less or insignificant stabilization of the carbocation intermediate **Int2** as compared to radical **Int1**

These results support the conclusion that aMOx and $P450_{LA1}$ scaffolds generate an optimal electrostatic preorganization and local electric field that favors the formation of carbocation **Int2**.

VII. Absolute energies of the characterized stationary points

Table S3: Energies and thermochemistry parameters (at T = 298.15 K and P = 1 atm) of all DFT optimized stationary points reported in **Figures S1** to **S2** and **S4** and **Table S1**: Electronic energies (E), enthalpy (H), free energy (G), quasi harmonic corrected free energy (G-qh), electronic energies from high level single point calculations (E(SP)), and imaginary frequencies for the TS. All energies are given in a.u.

Structure	Electronic State	E	H	G	G-qh	E(SP)	Imag. Freq.
**[1 + Cpd I]**	doublet (d)	-1935.265125	-1934.780477	-1934.880918	-1934.878482	-3075.828461	-
(reactant complex)	quartet (q)	-1935.264817	-1934.780147	-1934.881164	-1934.878701	-3075.828095	-
**TS1**	doublet (d)	-1935.242306	-1934.759298	-1934.855056	-1934.853430	-3075.813350	907.8i
(conformer 1)	quartet (q)	-1935.243414	-1934.760303	-1934.854566	-1934.853497	-3075.814598	461.1i
**TS1**	doublet (d)	-1935.242243	-1934.759322	-1934.856449	-1934.854333	-3075.811944	649.7i
(conformer 2)	quartet (q)	-1935.242758	-1934.759627	-1934.855291	-1934.853638	-3075.811734	496.2i
**Int1**	quartet (q)	-1935.281328	-1934.796395	-1934.891310	-1934.890105	-3075.851040	-
**TS-rotation**	quartet (q)	-1935.276546	-1934.793228	-1934.885900	-1934.885140	-3075.847996	44.3i
**TS2**	quartet (q)	-1935.279376	-1934.795513	-1934.890321	-1934.889208	-3075.845657	265.9i
**[2 + Fe(III)-Porph]**	doublet (d)	-1935.325727	-1934.838700	-1934.932404	-1934.931468	-3075.894010	-
(product complex)	quartet (q)	-1935.325022	-1934.838378	-1934.940629	-1934.937327	-3075.886509	-
	doublet (d)	-1935.290374	-1934.806235	-1934.899050	-1934.898554	-3075.857663	-
**Int2**	MECP	-1935.277517	-1934.797501	-1934.891096	-1934.890811	-3075.846824[a]	No stationary
	quartet (q)	-1935.277506	-1934.794285	-1934.891641	-1934.890895	-3075.845175	-
**TS3**	doublet (d)	-1935.288331	-1934.805889	-1934.898489	-1934.897724	-3075.856393	520.8i
	quartet (q)	-1935.277177	-1934.795399	-1934.891983	-1934.890975	-3075.845409	293.5i
**[3 + Fe(III)-Porph]**	doublet (d)	-1935.361687	-1934.875234	-1934.970606	-1934.969210	-3075.926939	-
(product complex)	quartet (q)	-1935.359022	-1934.873030	-1934.975805	-1934.972938	-3075.923585	-

[a]E(SP) energy value of **Int2^{MECP}** was obtained at the quartet electronic state on the optimized MECP structure.

Table S4: Energies and thermochemistry parameters (at T = 298.15 K and P = 1 atm) of DFT key intermediate models which include water, an oriented external electric field, and/or in gas phase conditions as reported in **Figures S9**, **S26**, and **S27**: Electronic energies (E), enthalpy (H), free energy (G), quasi harmonic corrected free energy (G-qh), electronic energies from high level single point calculations (E(SP)), and imaginary frequencies for the TS. All energies are given in a.u.

Structure	Electronic State	E	H	G	G-qh	E(SP)	Imag. Freq.
**Int1-wat**	quartet (q)	-2011.707699	-2011.195223	-2011.298351	-2011.296838	-3152.335645	-
**Int2-wat**	doublet (d)	-2011.715644	-2011.203764	-2011.303542	-2011.302938	-3152.342817	-
	quartet (d)	-2011.705827	-2011.194723	-2011.299252	-2011.298181	-3152.336261	-
**Int1-EF**	quartet (q)	-1935.281356	-1934.796473	-1934.891700	-1934.890313	-3075.861966	-
**Int2-EF**	doublet (d)	-1935.290312	-1934.806200	-1934.898890	-1934.898493	-3075.891142	-
	quartet (d)	-1935.277521	-1934.794305	-1934.891691	-1934.890820	-3075.881490	-
**Int1** (gas phase)	quartet (q)	-1935.266178	-1934.781378	-1934.877347	-1934.875499	-3075.838272	-
**Int2** (gas phase)	doublet (d)	-1935.267514	-1934.784403	-1934.878430	-1934.877433	-3075.837333	-
**Int1-EF** (gas phase)	quartet (q)	-1935.266178	-1934.781378	-1934.877340	-1934.875497	-3075.843462	-
**Int1-EF** (gas phase)	doublet (d)	-1935.267514	-1934.784402	-1934.878430	-1934.877433	-3075.858397	-
	quartet (d)	-1935.254937	-1934.772121	-1934.873096	-1934.870984	-3075.837275	-

Table S5: Energies and thermochemistry parameters (at T = 298.15 K and P = 1 atm) of all QM/MM optimized stationary points reported in **Figure S14**: QM Electronic energies (E_{QM}), MM energies (E_{MM}), enthalpy corrections (H correction), free energy corrections (G correction), QM electronic energies from high level single point calculations (E_{QM}(SP)), and imaginary frequencies for the TS. All energies are given in a.u.

Structure	Electronic State	E_{QM}	E_{MM}	H correction	G correction	E_{QM}(SP)	Imag. Freq.
LA1-**1**	doublet (d)	-1935.351349	-33.084589	21.514788	19.219301	-3075.754223	-
	quartet (q)	-1935.351338	-33.084660	21.514809	19.219092	-3075.754211	-
LA1-**TS1**	doublet (d)	-1935.330082	-33.078188	21.513777	19.221848	-3075.732727	1176.5i
	quartet (q)	-1935.329990	-33.078586	21.512806	19.219680	-3075.730383	536.6i
LA1-**Int1**	doublet (d)	-1935.371117	-33.076224	21.514896	19.222621	-3075.763804	-
	quartet (q)	-1935.361684	-33.082500	21.515726	19.220808	-3075.763805	-
LA1-**TS2**	quartet (q)	-1935.365557	-33.077532	21.515204	19.221040	-3075.765857	246.6i
LA1-**2-S**	doublet (d)	-1935.400827	-33.073524	21.518979	19.228222	-3075.798238	-
	quartet (q)	-1935.413905	-33.064068	21.518725	19.225065	-3075.811396	-
LA1-**Int2**	doublet (d)	-1935.368357	-33.076648	21.515114	19.223893	-3075.768582	-
	quartet (q)	-1935.378882	-33.065326	21.514123	19.220382	-3075.778619	-
LA1-**TS3-cis-re**	doublet (d)	-1935.368594	-33.075916	21.513368	19.222331	-3075.762461	568.4i
	quartet (q)	-1935.376298	-33.068129	21.512809	19.219646	-3075.776528	252.9i
LA1-**TS3-trans-si**	doublet (d)	-1935.363562	-33.077474	21.513879	19.224325	-3075.765706	609.0i
	quartet (q)	-1935.374502	-33.068434	21.512948	19.219898	-3075.775666	365.1i
LA1-**3**	doublet (d)	-1935.440131	-33.082048	21.517279	19.223487	-3075.840844	-
	quartet (q)	-1935.455678	-33.073811	21.517330	19.221186	-3075.856313	-

Table S6: Energies and thermochemistry parameters (at T = 298.15 K and P = 1 atm) of all QM/MM optimized stationary points reported in **Figures S15** and **S18**: QM Electronic energies (E_{QM}), MM energies (E_{MM}), enthalpy corrections (H correction), free energy corrections (G correction), QM electronic energies from high level single point calculations (E_{QM}(SP)), and imaginary frequencies for the TS. All energies are given in a.u.

Structure	Electronic State	E_{QM}	E_{MM}	H correction	G correction	E_{QM}(SP)	Imag. Freq.
LA1-**1**	doublet (d)	-1935.332059	-33.746423	21.075534	18.825776	-3075.735388	-
	quartet (q)	-1935.332095	-33.746318	21.075581	18.825418	-3075.735286	-
LA1-**TS1**	doublet (d)	-1935.315493	-33.738259	21.073208	18.825083	-3075.717371	565.6i
	quartet (q)	-1935.312160	-33.742797	21.073874	18.825508	-3075.713645	536.6i
LA1-**Int1**	doublet (d)	-1935.356247	-33.737708	21.075330	18.828193	-3075.752882	-
	quartet (q)	-1935.348563	-33.742400	21.075907	18.827339	-3075.750662	-
LA1-**TS2**	quartet (q)	-1935.352368	-33.737562	21.075357	18.827551	-3075.752516	232.7i
LA1-**2-R**	doublet (d)	-1935.389901	-33.736014	21.078196	18.829823	-3075.788047	-
	quartet (q)	-1935.402571	-33.733395	21.077863	18.827183	-3075.801398	-
LA1-**Int2**	doublet (d)	-1935.367946	-33.730257	21.074889	18.829591	-3075.767176	-
	quartet (q)	-1935.375375	-33.719533	21.073837	18.824622	-3075.774333	-
LA1-**TS3-cis-si**	doublet (d)	-1935.365349	-33.730285	21.072931	18.826421	-3075.766575	662.5i
	quartet (q)	-1935.373458	-33.720084	21.072514	18.824221	-3075.773553	475.8i
LA1-**TS3-trans-re**	doublet (d)	-1935.362167	-33.732088	21.073130	18.825918	-3075.764348	547.3i
	quartet (q)	-1935.372045	-33.720877	21.072808	18.824431	-3075.772187	359.4i
LA1-**3**	doublet (d)	-1935.438046	-33.733051	21.077289	18.828491	-3075.837861	-
	quartet (q)	-1935.451814	-33.722890	21.076884	18.826072	-3075.850967	-

Table S7: Energies and thermochemistry parameters (at T = 298.15 K and P = 1 atm) of all QM/MM optimized stationary points reported in **Figures S19** and **S22**: QM Electronic energies (E_{QM}), MM energies (E_{MM}), enthalpy corrections (H correction), free energy corrections (G correction), QM electronic energies from high level single point calculations (E_{QM}(SP)), and imaginary frequencies for the TS. All energies are given in a.u.

Structure	Electronic State	E_{QM}	E_{MM}	H correction	G correction	E_{QM}(SP)	Imag. Freq.
aMOx-**1**	doublet (d)	-1935.333357	-32.712510	21.216497	18.962179	-3075.737987	-
	quartet (q)	-1935.333224	-32.712667	21.216535	18.962108	-3075.737716	-
aMOx-**TS1**	doublet (d)	-1935.311884	-32.721937	21.215266	18.965431	-3075.717137	730.71i
	quartet (q)	-1935.308749	-32.724638	21.215219	18.965361	-3075.713160	562.80i
aMOx-**Int1**	doublet (d)	-1935.353642	-32.722520	21.215587	18.964836	-3075.753075	-
	quartet (q)	-1935.346585	-32.729377	21.216309	18.964322	-3075.752112	-
aMOx-**TS-rotation**	doublet (d)	-1935.332542	-32.728321	21.215027	18.965502	-3075.735061	198.9i
	quartet (q)	-1935.333718	-32.732248	21.214739	18.965276	-3075.738736	28.5i
aMOx-**TS2 pro-S**	doublet (d)	-1935.343453	-32.724584	21.216917	18.968298	-3075.745461	165.36i
	quartet (q)	-1935.344953	-32.720409	21.216191	18.966326	-3075.746106	204.84i
aMOx-**TS2 pro-R**	doublet (d)	-1935.345426	-32.713461	21.218015	18.972809	-3075.746322	272.29i
	quartet (q)	-1935.355773	-32.691483	21.217486	18.970633	-3075.757005	283.45i
aMOx-**2-S**	doublet (d)	-1935.384645	-32.719154	21.219332	18.968274	-3075.788313	-
	quartet (q)	-1935.396595	-32.711121	21.219612	18.966711	-3075.799978	-
aMOx-**2-R**	doublet (d)	-1935.374758	-32.718887	21.220610	18.971738	-3075.777062	-
	quartet (q)	-1935.391825	-32.716978	21.218939	18.967188	-3075.793889	-
aMOx-**Int2**	doublet (d)	-1935.365568	-32.704801	21.215140	18.965195	-3075.768084	-
	quartet (q)	-1935.363783	-32.699454	21.214301	18.961688	-3075.764836	- [a]
aMOx-**TS3-cis-re**	doublet (d)	-1935.364189	-32.705604	21.213734	18.965050	-3075.767524	467.16i
	quartet (q)	-1935.364236	-32.700888	21.213009	18.962149	-3075.766227	656.28i [b]
aMOx-**TS3-trans-si**	doublet (d)	-1935.361163	-32.704075	21.214167	18.965147	-3075.765368	518.92i
	quartet (q)	-1935.361838	-32.699253	21.213483	18.962155	-3075.764167	378.06i
aMOx-**3**	doublet (d)	-1935.427515	-32.709415	21.217914	18.965831	-3075.830264	-
	quartet (q)	-1935.433494	-32.709659	21.217860	18.963220	-3075.835787	-

[a] aMOx-**Int2^{q}** was optimized with C2–*cis*-H, C2–*trans*-H, and *cis*-H–*trans*-H distances frozen. Distance values are taken from optimized structure in the doublet state. Frequency calculation showed that the optimized structure has all frequencies positive.

[b] aMOx-**TS3-cis-re^{q}** was optimized with C2–*cis*-H, C1–*cis*-H and O–C1 distances frozen. Distance values are taken from optimized structure in the doublet state. Frequency calculation showed that all frequencies of the optimized structure are positive except one, which corresponds to the H-migration coordinate.

Table S8: Energies and thermochemistry parameters (at T = 298.15 K and P = 1 atm) of all QM/MM optimized stationary points reported in **Figure S23**: QM Electronic energies (E_{QM}), MM energies (E_{MM}), enthalpy corrections (H correction), free energy corrections (G correction), QM electronic energies from high level single point calculations (E_{QM}(SP)), and imaginary frequencies for the TS. All energies are given in a.u.

Structure	Electronic State	E_{QM}	E_{MM}	H correction	G correction	E_{QM}(SP)	Imag. Freq.
aMOx-**1**	doublet (d)	-1935.338494	-34.059275	18.960519	16.933275	-3075.741695	-
	quartet (q)	-1935.336423	-34.061060	18.960343	16.932280	-3075.739559	-
aMOx-**TS1**	doublet (d)	-1935.313335	-34.038172	18.959442	16.934153	-3075.715825	815.70i
	quartet (q)	-1935.309333	-34.047519	18.958746	16.931954	-3075.712011	644.22i
aMOx-**Int1**	doublet (d)	-1935.355270	-34.036612	18.960335	16.934571	-3075.751600	-
	quartet (q)	-1935.348978	-34.042221	18.961458	16.935269	-3075.751028	-
aMOx-**TS2**	doublet (d)	-1935.351443	-34.037669	18.960346	16.936449	-3075.746717	298.38i
	quartet (q)	-1935.353640	-34.035004	18.960589	16.935521	-3075.753894	291.80i
aMOx-**2-R**	doublet (d)	-1935.394311	-34.036759	18.963203	16.936829	-3075.796103	-
	quartet (q)	-1935.411262	-34.028680	18.963239	16.936302	-3075.812572	-
aMOx-**Int2**	doublet (d)	-1935.350515	-34.046698	18.959657	16.934921	-3075.751475	-
	quartet (q)	-1935.359256	-34.036149	18.958777	16.931831	-3075.759424	-
aMOx-**TS3-cis-si**	doublet (d)	-1935.348892	-34.045948	18.958300	16.933753	-3075.751202	594.37i
	quartet (q)	-1935.359837	-34.034443	18.957697	16.931455	-3075.761026	422.01i
aMOx-**TS3-trans-re**	doublet (d)	-1935.347017	-34.047826	18.958259	16.933640	-3075.749718	568.28i
	quartet (q)	-1935.358925	-34.035410	18.957669	16.930973	-3075.760527	391.82i
aMOx-**3**	doublet (d)	-1935.426242	-34.050773	18.962880	16.934412	-3075.827505	-
	quartet (q)	-1935.444105	-34.039749	18.962439	16.931293	-3075.845771	-

B. Experimental section

I. Materials and Methods

(A) Chemicals and Enzymes: All solvents, buffer components and chemicals were purchased form commercial suppliers (Merck, Sigma-Aldrich, Alfa Aesar, Fisher Scientific, Carl Roth). Lysozyme and DNase I were purchased from Carl Roth and GoldBio respectively.

(B) NMR spectroscopy: ^{1}H and ^{13}C NMR spectra were recorded on a Bruker Avance spectrometer working at a frequency of 500 MHz (protons) using $CDCl_3$ as solvents. Chemical shifts (δ) are given in ppm and referenced to the residual solvent peak or tetramethylsilane. Coupling constants (Hz) and signal multiplicity (s = singlet, d = doublet, dt = doublet of triplets, t = triplet, m = multiplet) are as well noted in the conventional form.

(C) GC-MS analysis: Gas chromatography-mass spectrometry was carried out on an Agilent GC 7820A instrument coupled with an Agilent 5977B Series mass selective detector (MSD). An Agilent HP-5MS UI column (30 m × 250 µm × 0.25 µm) was operated with hydrogen as carrier gas (40 cm s^{-1}). Injector temperature: 250°C. Split mode with a split ratio of 1:50. Electron ionization of the analyte with 70 eV acceleration voltage. Temperature profile: 90°C for 2 min, 5°C/min to 100°C, 10°C/min to 130°C, 50°C/min to 200 min, hold 0.5 min.

(D) Chiral GC analysis: Chiral gas chromatography analysis was performed on a Shimadzu GC-2010 instrument equipped with a flame ionization detector (FID) using an Agilent CP-Chirasil-Dex CB column (30 m × 250 µm × 0.25 µm, CP7502) with hydrogen as carrier gas (40 cm s^{-1} linear velocity). Injector temperature: 220°C. Split mode with a split ratio of 1:20, 1 µL injection volume. Detector temperature: 250°C. Temperature profile: 70°C for 2 min, 4°C/min to 102°C, 10°C/min to 140°C, 30°C/min to 190 min, hold 4 min.

(E) DNA sequences: Gene and amino acid sequence of phenylacetaldehyde reductase from *Rhodococcus* Sp., ST-10 (PAR-ADH) cloned into a pET22b(+) vector containing a C-terminal 6×His-tag.[68]

PAR-ADH

```
ATGAAGGCCATCCAGTACACTCGCATTGGCGCTGAGCCTGAACTGACTGAGATCCCAAAGCCCGAGCCCGGGCCCGGAGAAGT
CCTTCTTGAAGTGACAGCAGCCGGAGTTTGTCACTCTGATGATTTTATTATGAGCTTGCCAGAAGAACAGTATACCTACGGGT
TACCCTTGACATTAGGACACGAAGGAGCAGGGAAGGTCGCAGCCGTGGGTGAGGGCGTTGAGGGATTAGATATCGGCACGAAC
GTCGTAGTGTACGGCCCGTGGGGCTGTGGAAATTGCTGGCACTGTTCTCAGGGTCTTGAGAACTATTGTAGTCGCGCACAGGA
GTTAGGAATTAACCCTCCAGGATTGGGAGCTCCAGGGGCATTGGCCGAGTTTATGATCGTAGATAGTCCCCGCCATTTAGTGC
CTATTGGAGACTTAGACCCAGTTAAAACTGTTCCATTGACCGACGCGGGTCTTACACCTTATCACGCTATTAAACGTAGCTTA
CCCAAGCTTCGTGGCGGATCGTATGCGGTGGTTATTGGCACGGGCGGCCTTGGCCATGTCGCCATCCAATTATTACGTCACTT
GTCGGCGGCCACTGTCATTGCCTTGGACGTTAGTGCGGATAAACTTGAATTAGCTACGAAAGTCGGGGCCCATGAGGTTGTGT
TAAGCGATAAAGATGCTGCCGAAAATGTACGTAAGATCACAGGATCACAGGGAGCTGCACTTGTCTTAGATTTCGTCGGATAT
CAGCCCACAATCGATACCGCGATGGCCGTGGCCGGTGTCGGTAGCGATGTCACCATTGTGGGCATTGGGGACGGACAAGCCCA
CGCAAAAGTCGGATTCTTTCAAAGCCCCTACGAAGCTTCTGTAACCGTTCCTTACTGGGGTGCACGCAACGAATTAATCGAAC
```

TGATCGATTTAGCCCACGCCGGGATCTTCGACATTTCGGTAGAAACTTTCTCTTTGGATAACGGTGCAGAGGCGTACCGTCGC
TTGGCGGCGGGTACGCTGAGTGGCCGTGCCGTGGTGGTTCCGGGTTTGCTCGAGCACCATCACCACCATCACTGA

MKAIQYTRIGAEPELTEIPKPEPGPGEVLLEVTAAGVCHSDDFIMSLPEEQYTYGLPLTLGHEGAGKVAAVGEGVEGLDIGTN
VVVYGPWGCGNCWHCSQGLENYCSRAQELGINPPGLGAPGALAEFMIVDSPRHLVPIGDLDPVKTVPLTDAGLTPYHAIKRSL
PKLRGGSYAVVIGTGGLGHVAIQLLRHLSAATVIALDVSADKLELATKVGAHEVVLSDKDAAENVRKITGSQGAALVLDFVGY
QPTIDTAMAVAGVGSDVTIVGIGDGQAHAKVGFFQSPYEASVTVPYWGARNELIELIDLAHAGIFDISVETFSLDNGAEAYRR
LAAGTLSGRAVVVPGLLEHHHHHH

In preliminary work, $P450_{LA1}$ was cloned into a pET22b(+) vector containing a C-terminal 6×His-tag.[69] Gene and amino acid sequence of cytochrome $P450_{LA1}$ monooxygenase (Uniprot ID: A0P0F6) as well as P7 and anti-Markovnikov oxygenase (aMOx) are given:

$P450_{LA1}$

ATGGAACGCACTGCAAATCCAGCGGACGTTCCGGCTGGTGGTAAATCTTCTGAAGGCAAGGCGGGTACTCCACCGGCTGCTGA
AGCTCAATGCCCTTTCAGCAAAATGGCAGCAGATTTCGACGCTTTCGCAGGCCCATATCAGGCTGATCCGGCAGAAGCGCTGC
GTTGGTCTCGTGACCAGCTGCCGGTTTTCTATTCCCCGAACCTGGGTTACTGGGTGGTTTCTCGTTACGATGATATCAAAGCT
GTTTTTCGTGACAACATCCTGTTCAGCCCGCGTAACGCTCTGGAAAAAATTACTCCGGCAACCCCGGAAGCGATGGAGGTCCT
GAAAGGTTATGGTTACGCAATGAACCGTACCATGGTTAACGAAGACGAACCAGTTCACATGGAACGTCGTCGTGCACTGATGG
GCCACTTCCTGCCGGACAATCTGGAAGCTCGTCAGGAGATGGTACGCCGCCTGACCCGCGAAAAAATCGATGCATTCATCGAT
TCCGGTCGCGTGGATCTGGTGGAAGCCATGCTGTATGAGGTTCCGCTGAACGTTGCTCTGCACTTCCTGGGCGTTCCGGAGGA
TGACATTGCCATTCTGAAAAACTTTTCTGTCGCACACAGCGTCAACACCTGGGGTAAACCGACCGATGAGCAGCAGGTTGCGA
TCGCACACGACGTTGGTCAGTTCTGGAACTATGCTGGTAAAATCATCGAAAAAATGCGCAAGGAACCGGACGGTACCGGTTGG
ATGCACGAAACCATCCGTAAAAACGCAGAAATGCCGGATATTGTTCCGGATTCTTATGTTCACTCCATGATGATGGCGATCAT
CGTTGCGGCACACGAGACCACCAGCCTGGCCTCTGCAGGTATGTTTAAAACCCTGCTGACTCACCGTCAGGCTTGGCAGGATA
TCTGCGAGGACCCGTCCCTGATTCCGAACGCAGTTGAGGAGTGTCTGCGTTATAGCGGCTCCATCGTGGCATGGCGTCGTCAA
GCTACGGCTGCCACCCGTATCGGTGGTGTCGACATCCCGGAAGGTGCTAAACTGCTGATCGTTCAAGCATCTGGTAATCAGGA
TGAGCGTCACTTCGAGGATGGTGACAAATTTGACATCTACCGCGATAACGCGGTGGACCACCTGACCTTTGGCTACGGTTCTC
ATCAGTGTATGGGCAAAAACATTGCCCGTATGGAGATGCGCATCTTTCTGGAAGAAATGACTCGTCGCCTGCCTCACCTGCAA
CTGGCGGAACAGGAATTCACTTACCTGTCCAACACCAGCTTTCGTGGTCCGGATCATGTGTGGGTCGAATGGGATCCGGAAAA
AAACCCTGAACGTGCCGATCCGAGCCTGGCTAACGGCAACCACCGTTTTCCAGTTGGTGCCCCAGCCCGCCGTGATATTGCAC
GTAAAATTCGCATCAAAACTGTTCGTCGTGAAGCCGACGGCATCCTGGGCCTGACCATTGAGGATGCAAAGGGTCGTTCTCTG
CCACGTTGGTCCGCTGGTGCGCACATCGAAGTGTGTGTTGACGGCTTCGACCGTAAATATTCCCTGTGTGGTCGTGCGGACTC
CCGTGACTATGATATCGCAGTTCTGCTGGAAGAAGGCGGTCGCGGTGGTAGCCGTCGTATTCACGAAGTGGCTGCCGAGGGCC
TGGAGCTGCGCCTGCGTGGTCCTTCTAACCTGTTCCGCCTGGACGAACAGGCGCGTTCCTATGTTCTGATTGCGGGTGGCATT
GGTATCACCCCGATCCTGGCAATGGCGGACCACCTGAAAGCCCTGGGTCGTGACTACACCATCCACTACTGCGGTCGTTCTCG
TCGTTCTATGGCATTCCTGGACCGTCTGCAGGCAGACCATGGCGAGCGTCTGTCTGTGCATGCTGGCGATGAGAACCGTCACG
CCGAGCTGGCTGGTATTGTTGCCTCCCTGCCGGAAGGTGGCCAGATTTACGCATGTGGTCCGGAACGTATGATCAGCGAGCTG
GAAGATCTGACCGCCCGTCTGCCACATGGCACCCTGCATTTCGAGCACTTCAGCGCTCAGGAAACTGCCCTGGACCCGTCTAA
AGAAAACGCATTCCAGGTAGAACTGAAAGATTCCGGTCTGACTCTGGAGGTGGCTGCGAACGTTACCCTGCTGGATGCACTGC
TGGCGTCTGGTATCGATATCTCTTGTGACTGCCGTGAAGGCCTGTGTGGCTCTTGCGAGGTAGAAGTCCTGGAGGGCGAGATC
GACCACCGCGACGTGGTACTGACTCGCACCGAACGTGCGGAAAACCGTCGCATGATGTCTTGCTGTTCCCGCTCTGTAAAAGG
CGGTAAGCTGAAACTGGCACTGCTCGAGCACCACCACCACCACCACTGA

MERTANPADVPAGGKSSEGKAGTPPAAEAQCPFSKMAADFDAFAGPYQADPAEALRWSRDQLPVFYSPNLGYWVVSRYDDIKA
VFRDNILFSPRNALEKITPATPEAMEVLKGYGYAMNRTMVNEDEPVHMERRRALMGHFLPDNLEARQEMVRRLTREKIDAFID
SGRVDLVEAMLYEVPLNVALHFLGVPEDDIAILKNFSVAHSVNTWGKPTDEQQVAIAHDVGQFWNYAGKIIEKMRKEPDGTGW
MHETIRKNAEMPDIVPDSYVHSMMMAIIVAAHETTSLASAGMFKTLLTHRQAWQDICEDPSLIPNAVEECLRYSGSIVAWRRQ
ATAATRIGGVDIPEGAKLLIVQASGNQDERHFEDGDKFDIYRDNAVDHLTFGYGSHQCMGKNIARMEMRIFLEEMTRRLPHLQ
LAEQEFTYLSNTSFRGPDHVWVEWDPEKNPERADPSLANGNHRFPVGAPARRDIARKIRIKTVRREADGILGLTIEDAKGRSL
PRWSAGAHIEVCVDGFDRKYSLCGRADSRDYDIAVLLEEGGRGGSRRIHEVAAEGLELRLRGPSNLFRLDEQARSYVLIAGGI
GITPILAMADHLKALGRDYTIHYCGRSRRSMAFLDRLQADHGERLSVHAGDENRHAELAGIVASLPEGGQIYACGPERMISEL
EDLTARLPHGTLHFEHFSAQETALDPSKENAFQVELKDSGLTLEVAANVTLLDALLASGIDISCDCREGLCGSCEVEVLEGEI
DHRDVVLTRTERAENRRMMSCCSRSVKGGKLKLALLEHHHHHH

P450$_{LA1}$-P7

ATGGAACGCACTGCAAATCCAGCGGACGTTCCGGCTGGTGGTAAATCTTCTGAAGGCAAGGCGGGTACTCCACCGGCTGCTGA
AGCTCAATGCCCTTTCAGCAAAATGGCAGCAGATTTCGACGCTTTCGCAGGCCCATATCAGGCTGATCCGGCAGAAGCGCTGC
GTTGGTCTCGTGACCAGCTGCCGGTTTTCTATTCCCCGAACCTGGGTTACTGGGTGGTTTCTCGTTACGATGATATCAAAGCT
GTTTTTCGTGACAACATCCTGTTCAGCCCGCGTAACGCTCTGGAAAAAATTACTCCGGCAACCCCGGAAGCGATGGAGGTCCT
GAAAGGTTATGGTTACGCAATGAACCGTGCCATGGTTAACGAAGACGAACCAGTTCACATGGAACGTCGTCGTGCACTGATGG
GCCACTTCCTGCCGGACAATCTGGAAGCTCGTCAGGAGATGGTACGCCGCCTGACCCGCGAAAAAATCGATGCATTCATCGAT
TCCGGTCGCGTGGATCTGGTGGAAGCCATGCTGTATGAGGTTCCGCTGAACGTTGCTCTGCACTTCCTGGGCGTTCCGGAGGA
TGACATTGCCATTCTGAAAAAGTTTTCTGTCGCACACAGCGTCAGCACCTGGGGTAAACCGACCGATGAGCAGCAGGTTGCGA
TCGCACACGACGTTGGTCAGTTCTGGAACTATGCTGGTAAAATCATCGAAAAAATGCGCAAGGAACCGGACGGTACCGGTTGG
ATGCACGAAACCATCCGTAAAAACGCAGAAATGCCGGATATTGTTCCGGATTCTTATGTTCACTCCATGATGATGGCGATCAT
CGTTGCGGCACACGAGACCACCAGCCTGGCCTCTGCAGGTATGTTTAAAACCCTGCTGACTCACCGTCAGGCTTGGCAGGATA
TCTGCGAGGACCCGTCCCTGATTCCGAACGCAGTTGAGGAGTGTCTGCGTTATAGCGGCTCCATCGTGGCATGGCGTCGTCAA
GCTACGGCTGCCACCCGTATCGGTGGTGTCGACATCCCGGAAGGTGCTAAACTGCTGATCGTTCAAGCATCTGGTAATCAGGA
TGAGCGTCACTTCGAGGATGGTGACAAATTTGACATCTACCGCGATAACGCGGTGGACCACCTGACCTTTGGCCACGGTTCTC
ATCAGTGTATGGGCAAAAACATTGCCCGTATGGAGATGCGCATCTTTCTGGAAGAAATGACTCGTCGCCTGCCTCACCTGCAA
CTGGCGGGACAGGAATTCACTTACCTGTCCAACACCAGCTTTCGTGGTCCGGATCATGTGTGGGTCGAATGGGATCCGGAAAA
AAACCCTGAACGTGCCGATCCGAGCCTGGCTAACGGCAACCACCGTTTTCCAGTTGGTGCCCCAGCCCGCCGTGATATTGCAC
GTAAAATTCGCATCAAAACTGTTCGTCGTGAAGCCGACGGCATCCTGGGCCTGACCATTGAGGATGCAAAGGGTCGTTCTCTG
CCACGTTGGTCCGCTGGTGCGCACATCGAAGTGTGTGTTGACGGCTTCGACCGTAAATATTCCCTGTGTGGTCGTGCGGACTC
CCGTGACTATGATATCGCAGTTCTGCTGGAAGAAGGCGGTCGCGGTGGTAGCCGTCGTATTCACGAAGTGGCTGCCGAGGGCC
TGGAGCTGCGCCTGCGTGGTCCTTCTAACCTGTTCCGCCTGGACGAACAGGCGCGTTCCTATGTTCTGATTGCGGGTGGCATT
GGTATCACCCCGATCCTGGCAATGGCGGACCACCTGAAAGCCCTGGGTCGTGACTACACCATCCACTACTGCGGTCGTTCTCG
TCGTTCTATGGCATTCCTGGACCGTCTGCAGGCAGACCATGGCGAGCGTCTGTCTGTGCATGCTGGCGATGAGAACCGTCACG
CCGAGCTGGCTGGTATTGTTGCCTCCCTGCCGGAAGGTGGCCAGATTTACGCATGTGGTCCGGAACGTATGATCAGCGAGCTG
GAAGATCTGACCGCCCGTCTGCCACATGGCACCCTGCATTTCGAGCACTTCAGCGCTCAGGAAACTGCCCTGGACCCGTCTAA
AGAAAACGCATTCCAGGTAGAACTGAAAGATTCCGGTCTGACTCTGGAGGTGGCTGCGAACGTTACCCTGCTGGATGCACTGC
TGGCGTCTGGTATCGATATCTCTTGTGACTGCCGTGAAGGCCTGTGTGGCTCTTGCGAGGTAGAAGTCCTGGAGGGCGAGATC
GACCACCGCGACGTGGTACTGACTCGCACCGAACGTGCGGAAAACCGTCGCATGATGTCTTGCTGTTCCCGCTCTGTAAAAGG
CGGTAAGCTGAAACTGGCACTGCTCGAGCACCACCACCACCACCACTGA

MERTANPADVPAGGKSSEGKAGTPPAAEAQCPFSKMAADFDAFAGPYQADPAEALRWSRDQLPVFYSPNLGYWVVSRYDDIKA
VFRDNILFSPRNALEKITPATPEAMEVLKGYGYAMNR**A**MVNEDEPVHMERRRALMGHFLPDNLEARQEMVRRLTREKIDAFID
SGRVDLVEAMLYEVPLNVALHFLGVPEDDIAILK**K**FSVAHSV**S**TWGKPTDEQQVAIAHDVGQFWNYAGKIIEKMRKEPDGTGW
MHETIRKNAEMPDIVPDSYVHSMMMAIIVAAHETTSLASAGMFKTLLTHRQAWQDICEDPSLIPNAVEECLRYSGSIVAWRRQ
ATAATRIGGVDIPEGAKLLIVQASGNQDERHFEDGDKFDIYRDNAVDHLTFG**H**GSHQCMGKNIARMEMRIFLEEMTRRLPHLQ
LA**G**QEFTYLSNTSFRGPDHVWVEWDPEKNPERADPSLANGNHRFPVGAPARRDIARKIRIKTVRREADGILGLTIEDAKGRSL
PRWSAGAHIEVCVDGFDRKYSLCGRADSRDYDIAVLLEEGGRGGSRRIHEVAAEGLELRLRGPSNLFRLDEQARSYVLIAGGI
GITPILAMADHLKALGRDYTIHYCGRSRRSMAFLDRLQADHGERLSVHAGDENRHAELAGIVASLPEGGQIYACGPERMISEL
EDLTARLPHGTLHFEHFSAQETALDPSKENAFQVELKDSGLTLEVAANVTLLDALLASGIDISCDCREGLCGSCEVEVLEGEI
DHRDVVLTRTERAENRRMMSCCSRSVKGGKLKLALLEHHHHHH

P450$_{LA1}$-P7 has the following mutations compared to P450$_{LA1}$: T121A, N201K, N209S, Y385H, E418G

aMOx

ATGGAGCGCACTGCAAATCCAGCGGACGTTCCGGCTGGTGGTAAATCTTCTGAAGGCAAGGCGGGTACTCCACCGGCTGCTGA
AGCTCAATGCCCTTTCAGCAAAATGGCAGCAGATTTCGACGCTTTCGCAGGCCCATATCAGGCTGATCCGGCAGAAGCGCTGC
GTTGGTCTCGTGACCAGCTGCCGGTTTTCTATTCCCCGAACCTGGGTTACTGGGTGGTTTCTCGTTACGATGATATCAAAGCT
GTTTTTCGTGACAACATCCTGTTCAGCCCGCGTAACGCTCTGGAAAAAATCACTCCGCTGACCCCGGAAGCGATGGAGGTCCT
GAAAGGTTATGGTTACGCACTGAACCATGCCATGATTAACGAAGACGAACCAGTTCACATGGAACGTCGTCGTGCACTGATGG
GCCACTTCCTGCCGGACAATCTGGAAGCTCGTCAGGAGATGGTACGCCGCCTGACCCGCGAAAAAATCGATGCATTCATCGAT
TCCGGTCGCGTGGATCTGGTGGAAGCCATGCTGTATGAGGTTCCACTGAACGTTGCCCTGCACTTCCTGGGCGTTCCGGAGGA
TGACATTGCCATTCTGAAAAAGTTTTCTGTCGCACACAGCGTCAGCACCTGGGGTAAACCGACCGATGAGCAGCAGGTTGCGA
TCGCACACGACGTTGGTCAGTTCTGGAACTATGCTGGTAAAATCATCGAAAAAATGCGCAAGGAACCGGACGGTACCGGTTGG
ATGCACGAAACCATCCGTAAAAACGCAGAAATGCCGGATATTGTCCCGGATTCTTATGTTCACTCCATGATGATGGCGATCAT
CGTTGCGGCACACGAGACCACCAGCCTGGCCTCTGCAGGTATGTTTAAAACCCTGCTGACTCACCGTCAGGCTTGGCAGGATA
TCTGCGAGGACCCGTCTCTGATTCCGAACGCAGTTGAGGAGTGTCTGCGTTATAGCGGCTCCGTTATGGCATGGCGTCGTCAA
GCTACGGCTGCCACCCGTATCGGTGGTGTCGACATCCCGGAAGGTGCTAAACTGCTGATCGTTCAAGCATCTGGTAATCAGGA
TGAGCGTCACTTCGAGGATGGTGACAAATTTGACATCTACCGCGATAACGCGGTGGACCACCTGACCTTTGGCGTGGGTTCTC
ACCAGTGTCTGGGCAAAAACATTGCCCGTATGGAGATGCGCATCTTTCTGGAAGAAATGACTCGTCGCCTGCCTCACCTGCAA
CTGGCGGGACAGGAATTCACTTACCTGTCCAACACCAGCTTTCGTGGTCCGGATCATGTGTGGGTCGAATGGGATCCGGAAAA
AAACCCTGAACGTGCCGATCCGAGCCTGGCTAACGGCAACCACCGTTTTCCAGTTGGTGCCCCAGCCCGCCGTGATATTGCAC
GTAAAATTCGCATCAAAACTGTTCGTCGTGAAGCCGACGGCATCCTGGGCCTGACCATTGAGGATGCAAAGGGTCGTTCTCTG
CCACGTTGGTCCGCTGGTGCGCACATCGAAGTGTGTGTTGACGGCTTCGACCGTAAATATTCCCTGTGTGGTCGTGCGGACTC
CCGTGACTATGATATCGCAGTTCTGCTGGAAGAAGGCGGTCGCGGTGGTAGCCGTCGTATTCACGAAGTGGCTGCCGAGGGCC
TGGAGCTGCGCCTGCGTGGTCCTTCTAACCTGTTCCGCCTGGACGAACAGGCGCGTTCCTATGTTCTGATTGCGGGTGGCATT
GGTATCACCCCGATCCTGGCAATGGCGGACCACCTGAAAGCCCTGGGTCGTGACTACACCATCCACTACTGCGGTCGTTCTCG
TCGTTCTATGGCATTCCTGGACCGTCTGCAGGCAGACCATGGCGAGCGTCTGTCTGTGCATGCTGGCGATGAGAACCGTCACG
CCGAGCTGGCTGGTATTGTTGCCTCCCTGCCGGAAGGTGGCCAGATTTACGCATGTGGTCCGGAACGTATGATCAGCGAGCTG
GAAGATCTGACCGCCCGTCTGCCACATGGCACCCTGCATTTCGAGCACTTCAGCGCTCAGGAAACTGCCCTGGACCCGTCTAA
AGAAAACGCATTCCAGGTAGAACTGAAAGAT
TCCGGTCTGACTCTGGAGGTGGCTGCGAACGTTACCCTGCTGGATGCACTGCTGGCGTCTGGTATCGATATCTCTTGTGACTG
CCGTGAAGGCCTGTGTGGCTCTTGCGAGGTAGAAGTCCTGGAGGGCGAGATCGACCACCGCGACGTGGTACTGACTCGCACCG
AACGTGCGGAAAACCGTCGCATGATGTCTTGCTGTTCCCGCTCTGTAAAAGGCGGTAAGCTGAAACTGGCACTGCTCGAGCAC
CACCACCACCACCACTGA

MERTANPADVPAGGKSSEGKAGTPPAAEAQCPFSKMAADFDAFAGPYQADPAEALRWSRDQLPVFYSPNLGYWVVSRYDDIKA
VFRDNILFSPRNALEKITP**L**TPEAMEVLKGYGYA**L**N**HA**M**I**NEDEPVHMERRRALMGHFLPDNLEARQEMVRRLTREKIDAFID
SGRVDLVEAMLYEVPLNVALHFLGVPEDDIAILK**K**FSVAHSV**S**TWGKPTDEQQVAIAHDVGQFWNYAGKIIEKMRKEPDGTGW
MHETIRKNAEMPDIVPDSYVHSMMMAIIVAAHETTSLASAGMFKTLLTHRQAWQDICEDPSLIPNAVEECLRYSGS**VM**AWRRQ
ATAATRIGGVDIPEGAKLLIVQASGNQDERHFEDGDKFDIYRDNAVDHLTFG**V**GSHQC**L**GKNIARMEMRIFLEEMTRRLPHLQ
LA**G**QEFTYLSNTSFRGPDHVWVEWDPEKNPERADPSLANGNHRFPVGAPARRDIARKIRIKTVRREADGILGLTIEDAKGRSL
PRWSAGAHIEVCVDGFDRKYSLCGRADSRDYDIAVLLEEGGRGGSRRIHEVAAEGLELRLRGPSNLFRLDEQARSYVLIAGGI
GITPILAMADHLKALGRDYTIHYCGRSRRSMAFLDRLQADHGERLSVHAGDENRHAELAGIVASLPEGGQIYACGPERMISEL
EDLTARLPHGTLHFEHFSAQETALDPSKENAFQVELKDSGLTLEVAANVTLLDALLASGIDISCDCREGLCGSCEVEVLEGEI
DHRDVVLTRTERAENRRMMSCCSRSVKGGKLKLALLEHHHHHH

aMOx has the following mutations compared to $P450_{LA1}$: A103L, M118L, R120H, T121A, V123I, N201K, N209S, I326V, V327M, Y385V, M391L, E418G

II. General procedures

(A) Large scale expression of P450 variants and PAR-ADH

E. coli BL21(DE3) cells were transformed with plasmid DNA encoding P450 variants or PAR-ADH and grown overnight in 5 mL LB medium (100 µg mL^{-1} ampicillin final concentration) at 37ºC, 180 rpm. Expression cultures were inoculated with 4 mL of preculture into 400 mL TB medium (100 µg mL^{-1} ampicillin final concentration) in a 2 L flask with baffles and incubated for 2 – 3 h at 37ºC and 100 rpm until an OD_{600} = 0.6 - 0.8 was reached. The flask was cooled on ice for 10 min before expression was induced (0.2 mM IPTG, final concentration). In addition, aminolaevulinic acid (0.5 mM, final concentration) was added for the expression of P450 variants. Induced cells were shaken for 20 h at 25ºC, 100 rpm. The cells were harvested (3220×g, 10 min, 4ºC) and stored at -20°C.

(B) Expression of $P450_{LA1}$ variants in 24-deepwell plates

The expression of P450 variants was also carried out in 24-deepwell plates for easier handling. Individual *E. coli* BL21(DE3) transformants containing plasmid DNA encoding P450 variants were cultivated in 4 mL/well of TB medium (100 µg mL^{-1} ampicillin, final concentration) for 20 h at 37°C, 250 rpm using humidity control. Expression cultures were inoculated with 300 µL of preculture into 3.6 mL of TB medium (100 µg mL^{-1} ampicillin, final concentration). The cultures were incubated for 4 h at 37ºC, 250 rpm, then cooled on ice for 10 min before induction with induction master mix (100 µL, 0.2 mM IPTG, 0.5 mM aminolaevulinic acid, final concentration). Induced cells were shaken for 20 h at 25ºC, 250 rpm using humidity control. The cells were harvested (3220×g, 10 min, 4ºC) and stored at -20°C for three days.

(C) Biotransformations

Styrene or isotopically labeled β-*d*-styrene was converted in an enzyme cascade employing the P450 variant together with an alcohol dehydrogenase (PAR-ADH) to convert styrene directly to 2-phenylethanol and for cofactor regeneration. Since aldehydes are prone to side reactions in buffered systems, we chose to prevent aldehyde accumulation by direct reduction to the corresponding alcohol with an alcohol dehydrogenase. *E. coli* BL21(DE3) cells were lysed with lysis buffer (3 mL/g wet cell pellet, 0.1 M NaH_2PO_4, pH 8.0, 0.15 M NaCl, 2% glycerol by weight, 1.0 mg/mL lysozyme, 0.2 mg/mL DNase I) for 4 h on ice followed by centrifugation (10 min, 20238×g, 4°C). The supernatant was used as lysate in biotransformations. For biotransformations, 100 µL of lysate were mixed with 584 µL NADH buffer solution (0.1 M NaH_2PO_4, pH 8.0, 0.15 M NaCl, 2% glycerol by weight, 5 mM NADH, final concentration) and 100 µL PAR-ADH lysate (prepared following the identic lysing protocol as described above) and 1% isopropanol for NADH cofactor regeneration. To start the reaction, 16 µL substrate stock in 1:1 DMSO/isopropanol (5 mM final substrate concentration, 1% isopropanol

for cofactor regeneration) were added in 2 mL screw-top glass vials. The biotransformation was stopped and extracted with 800 µL MTBE. The organic phase was analyzed using GC-MS or chiral GC.

Note: Instead of lysate, purified P450 enzyme (as prepared in chapter (D)) can also be used for biotransformations. A final enzyme concentration of 2 – 5 µM was typically used.

(D) ÄKTA purification of P450 variants

E. coli BL21(DE3) cells were lysed with lysis buffer (3 mL/g wet cell pellet, 0.05 M TRIS, pH 8.0, 0.05 M NaCl, 10% glycerol by weight, 10 mM imidazole, 1.0 mg/mL lysozyme, 0.2 mg/mL DNase I) for 4 h on ice using a Dounce homogenizer followed by centrifugation (35 min, 20,000×g, 4°C). The supernatant was filtered using a syringe filter (0.45 µm pore size, PES) before loading the IMAC column. The protein (containing a C-terminal 6×His-tag) was purified by loading the lysate on a nickel NTA column (5 mL HisTrap HP, GE Healthcare, Piscataway, NJ) using an ÄKTA purifier. The column was washed with 5 column volumes buffer A (0.05 M TRIS, pH 8.0, 0.05 M NaCl, 10% glycerol by weight, 10 mM imidazole) and the protein was eluted using a linear gradient from 100% buffer A to 100% buffer B (0.05 M TRIS, pH 8.0, 0.05 M NaCl, 10% glycerol by weight, 250 mM imidazole) over 7.5 column volumes. The combined fractions were dialyzed (2 L overnight, 2 L for 4 h, 0.05 M TRIS, pH 8.0, 0.05 M NaCl, 10% glycerol by weight, MWCO = 6 - 8 kDa) after purification. 200 µL aliquots were frozen using liquid nitrogen and stored at -20°C. The concentration of purified P450 enzymes was determined from ferrous carbon monoxide binding difference spectra using a previously reported protocol.[70]

(E) Determination of dissociation constants

A solution of styrene in DMSO (1 µL) was added to a buffered solution of purified $P450_{LA1}$ or aMOx (199 µL, 1 µM final concentration in 0.05 M TRIS, pH 8.0, 0.05 M NaCl, 10% glycerol by weight) to reach the following final concentrations: 0, 50, 100, 250, 500 and 1000 µM. UV/Vis spectra were recorded from 325 to 525 nm in 1 nm increments before and after addition of substrate and the difference spectra were determined by subtracting the enzyme reference spectra from the substrate-bound spectra. The absorbance differences between λ_{max} and λ_{min} were calculated and plotted against the substrate concentrations. The dissociation constants were determined by fitting the data to a binding isotherm model using the following formula:

$$\Delta Abs = ([styrene] \times \Delta Abs_{max})/([styrene] + K_d).$$

(F) Determination of kinetic parameters

For the determination of initial velocities, 100 µL of purified $P450_{LA1}$ (5.2 - 5.8 µM) or aMOx (2.3 - 2.4 µM) were mixed with 584 µL NADH buffer solution (0.1 M NaH_2PO_4, pH 8.0, 0.15 M NaCl, 2% glycerol by weight, 5 mM NADH, final concentration) and 100 µL PAR-ADH lysate (60 U/mL). To start the reaction, 16 µL styrene stock solution in 1:1 DMSO/isopropanol were added to reach the following final substrate concentrations: 50, 100, 250, 500, 750, 1000, 1500 and 2000 µM. The biotransformations were stopped after 0, 10, 15 and 60 minutes by extracting with 800 µL MTBE (containing 100 µM 3-phenylpropanol as internal standard). Each reaction was typically performed in duplicates. The organic phase was analyzed using GC-MS. The determined initial velocities were plotted against the substrate concentrations and the kinetic parameters were determined by fitting the data to the Michaelis-Menten equation.

(G) NADH consumption and H_2O_2 formation

To compare the NADH consumption and peroxide formation to product formation, 100 µL of purified $P450_{LA1}$ (4.5 µM) or aMOx (2.5 µM) were mixed with 29 µL NADH buffer solution (0.1 M NaH_2PO_4, pH 8.0, 0.15 M NaCl, 2% glycerol by weight, 250 µM NADH, final concentration) and either 655 µL lysis buffer (0.1 M NaH_2PO_4, pH 8.0, 0.15 M NaCl, 2% glycerol by weight) or 555 µL lysis buffer and 100 µL PAR-ADH lysate (60 U/mL). The reactions were started by adding 16 µL styrene stock solution (12.5 mM in 1:1 DMSO:isopropanol). After 15 minutes the absorption at 340 nm was measured to determine NADH consumption. 50 µL of the same reaction was used to determine the peroxide concentration using the Pierce™ quantitative peroxide assay kit (Thermo Fisher). The other reaction was stopped by extracting with 800 µL MTBE (containing 100 µM 3-phenylpropanol as internal standard) and the organic phase was analyzed using GC-MS.

III. Chemical synthesis of *d*-labeled styrenes

Reaction:

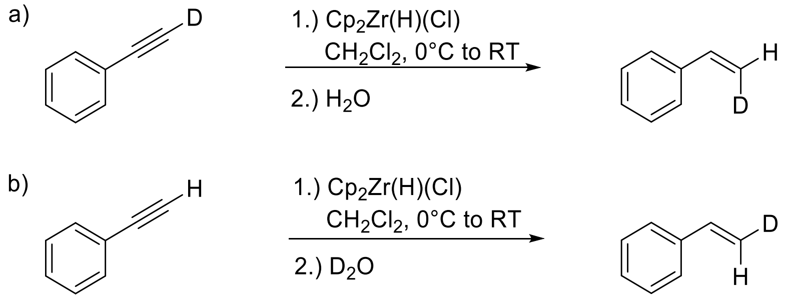

Scheme S1: Synthesis of a) *cis*-styrene-β-*d* and b) *trans*-styrene-β-*d* by hydrozirconation/hydrolysis according to literature.[71,72]

Under a nitrogen atmosphere, zirconocene hydrochloride (2.09 g, 7.7 mmol) was added in two equal portions to a solution of anhydrous dichloromethane (20 mL) and cooled to 0°C. After the addition of a) phenylacetylene-d_1 (784 µL, 7.0 mmol) or b) phenylacetylene (784 µL, 7.0 mmol), the reaction mixtures were kept in the dark, slowly warmed up to room temperature and stirred for 2 h. Either a) H_2O (1.0 mL) or b) D_2O (1.0 mL) was added and the reaction mixtures were stirred for further 2 h. The resulting mixtures were dried using $MgSO_4$, filtered and concentrated *in vacuo* to ca. 5 mL each (rotary evaporator without heating). Pentane (10 mL) was added and the resulting suspensions were passed through a pad of silica using pentane as eluent. The filtrate was concentrated *in vacuo* (rotary evaporator without heating) and distilled using a bulb to bulb distillation. Product fractions for both a) *cis*-styrene-β-*d* and b) *trans*-styrene-β-*d* were isolated as colorless liquid (ca. 200 µL each) showing >95% *d*-incorporation and 2 - 3% residual pentane from NMR analysis.

a) *cis*-Styrene-β-*d*

^1H NMR (500 MHz, $CDCl_3$) δ = 7.42–7.40 (m, 2H), 7.34–7.30 (m, 2H), 7.26–7.24 (m, 1H), 6.72 (dt, *J* = 10.8 Hz, 2.6 Hz, 1H), 5.22 (d, *J* = 11.0 Hz, 1H) ppm.

^{13}C NMR (125 MHz, $CDCl_3$) δ = 137.6, 136.8, 128.5, 127.8, 126.2, 113.5 (t, *J* = 23.6 Hz) ppm.

GC-MS *m/z*: 105, 104, 79, 78.

b) *trans*-Styrene-β-*d*

^1H NMR (500 MHz, $CDCl_3$) δ = 7.42–7.40 (m, 2H), 7.34–7.30 (m, 2H), 7.26–7.24 (m, 1H), 6.71 (d, *J* = 17.6 Hz, 1H), 5.73 (d, *J* = 17.6 Hz, 1H) ppm.

^{13}C NMR (125 MHz, $CDCl_3$) δ = 137.6, 136.8, 128.5, 127.8, 126.2, 113.5 (t, *J* = 24.6 Hz) ppm.

GC-MS *m/z*: 105, 104, 79, 78.

^{1}H NMR spectra:

cis-Styrene-β-*d* (residue fraction)

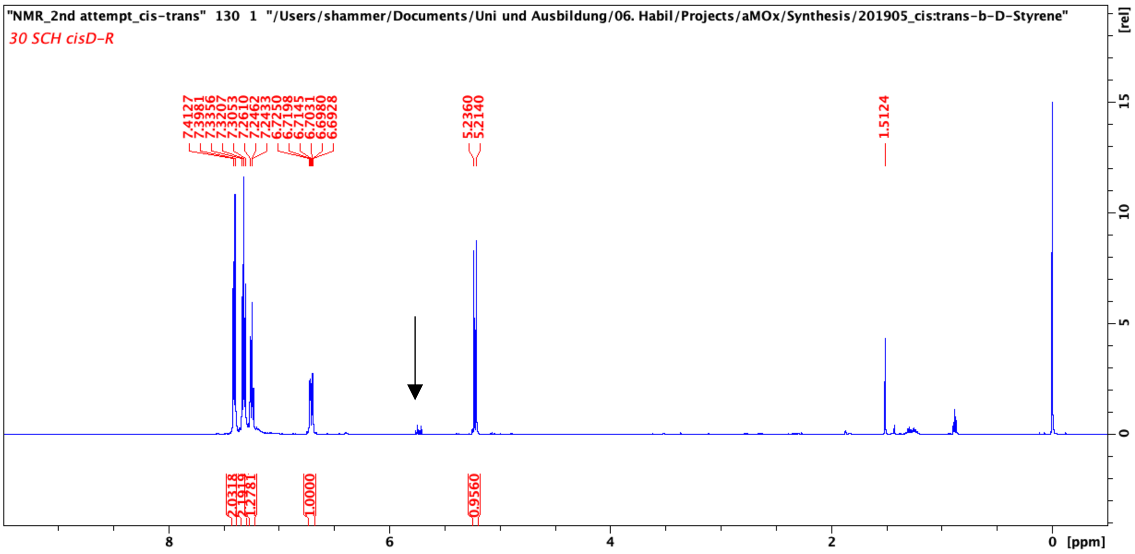

trans-Styrene-β-*d* (fraction 3)

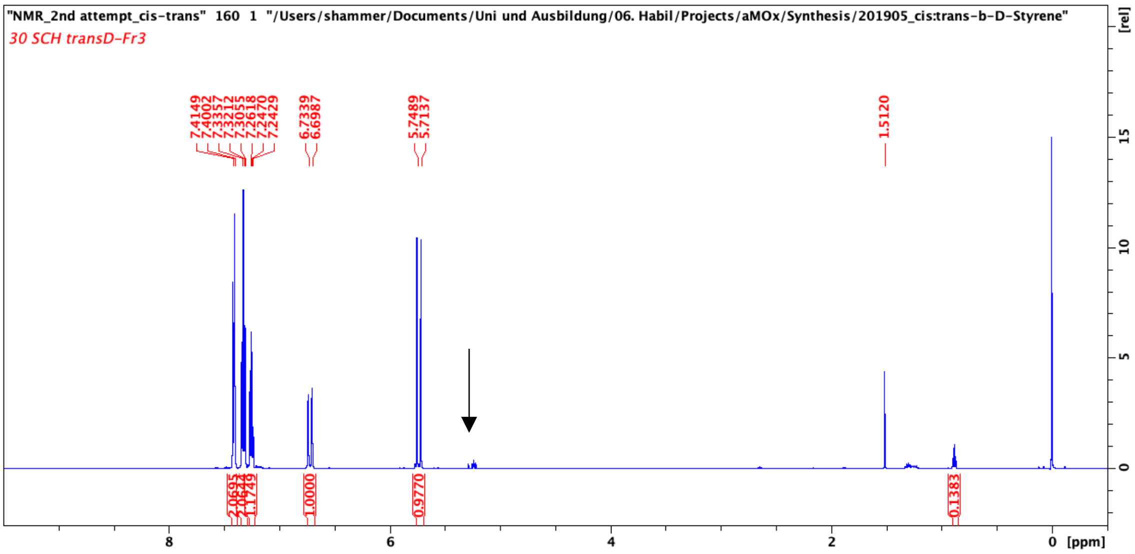

Styrene (commercial)

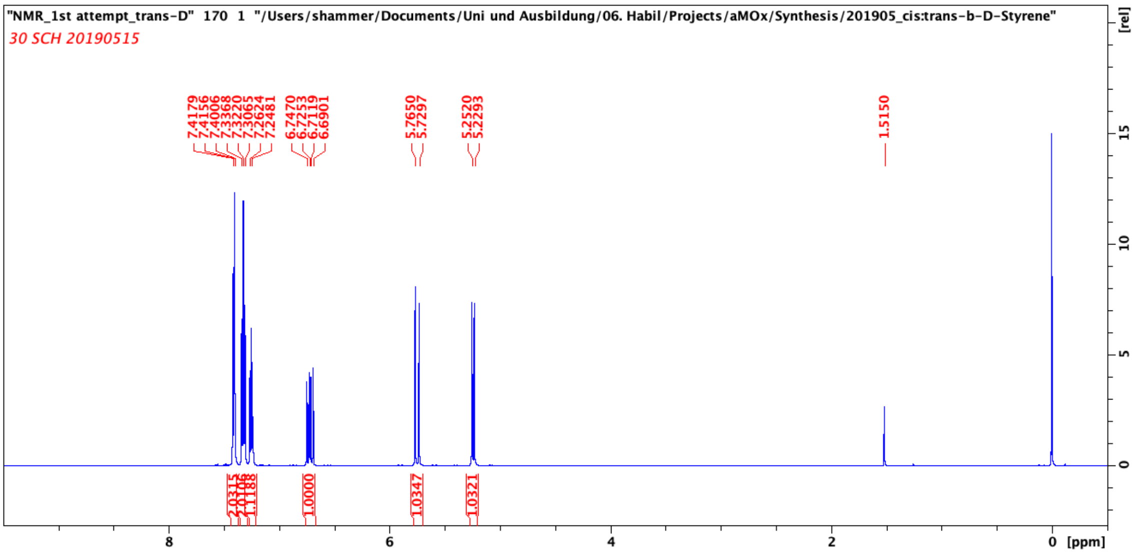

^{13}C NMR spectra:

cis-Styrene-β-*d* (residue fraction)

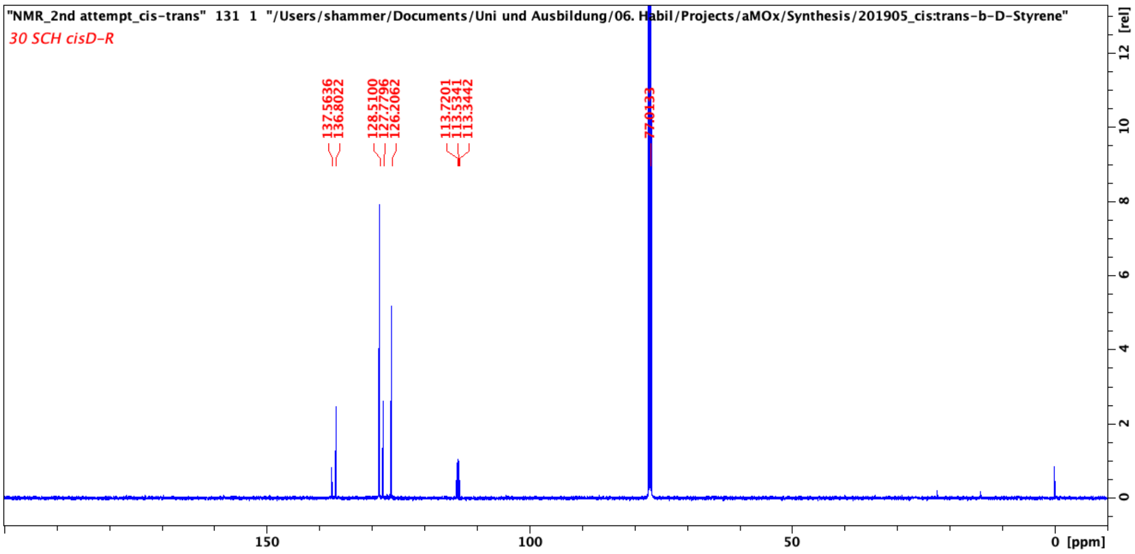

trans-Styrene-β-*d* (fraction 3)

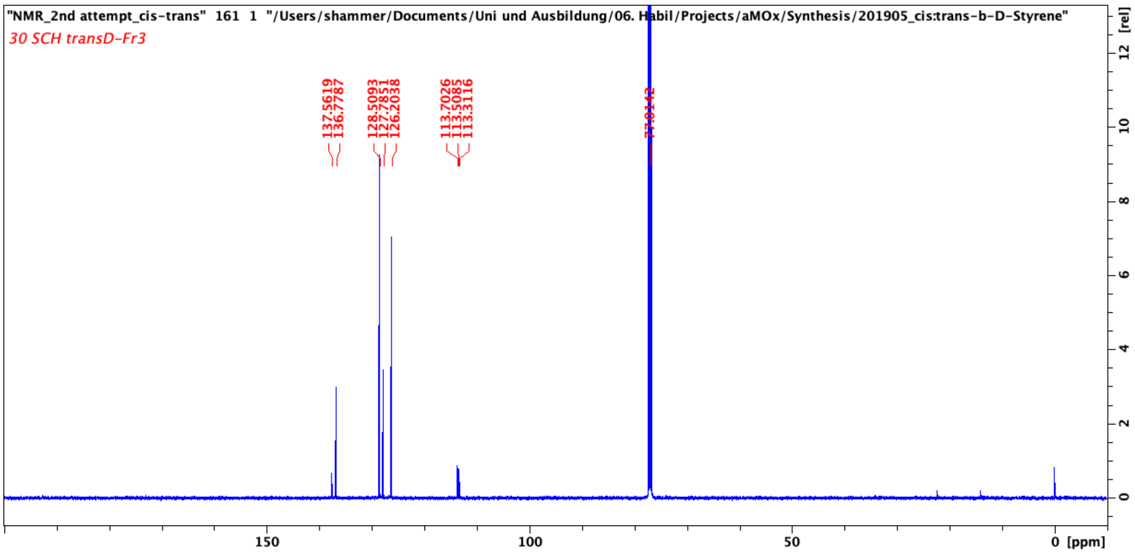

Styrene (commercial)

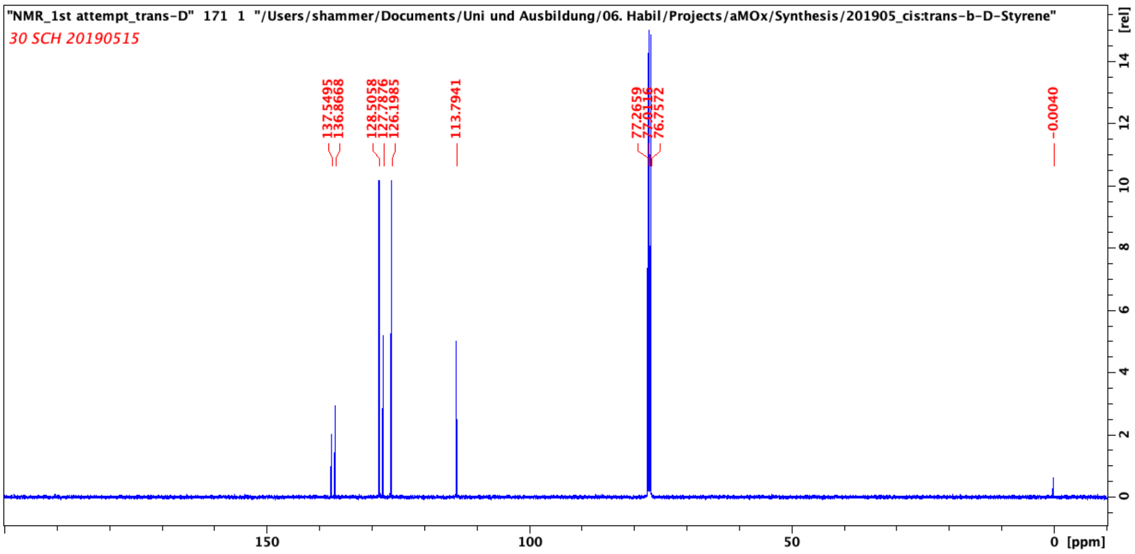

IV. Determination of the migration tendency (*cis* vs. *trans*)

Question: Does aMOx differentiate between these enantiotopic hydrogen atoms (blue)?

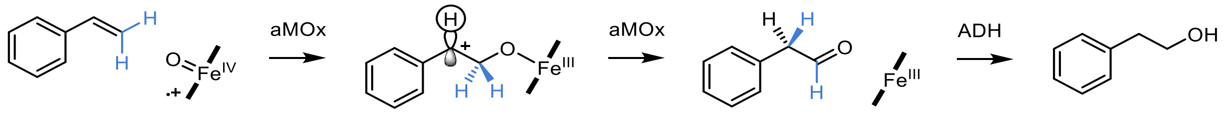

Approach: Conversion of isotopically labeled substrates (*cis*-styrene-β-*d* and *trans*-styrene-β-*d*) and MS-analysis of the reduced products. The fragmentation pattern provides information about the deuteride migration to the neighboring (benzylic) carbon atom.

Figure S30: Rationale behind the experiment of using MS fragmentation patterns to determine the position of the deuterium in the resulting product. An increase in mass (*m/z* = +1) for the benzylic cleavage and McLafferty rearrangement fragments provides information about the deuteride migration.

Bioconversion of unlabeled styrene:

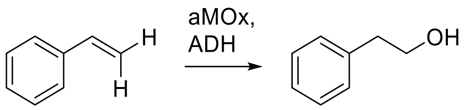

- Molecular ion peak *m/z* = 122
- After benzylic cleavage: *m/z* = 91
- McLafferty rearrangement product: *m/z* = 92

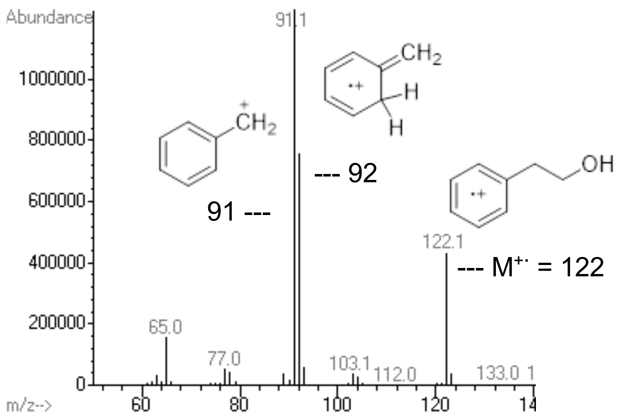

Mass fragmentations observed for 2-phenylethanol:

Benzylic cleavage:[73]

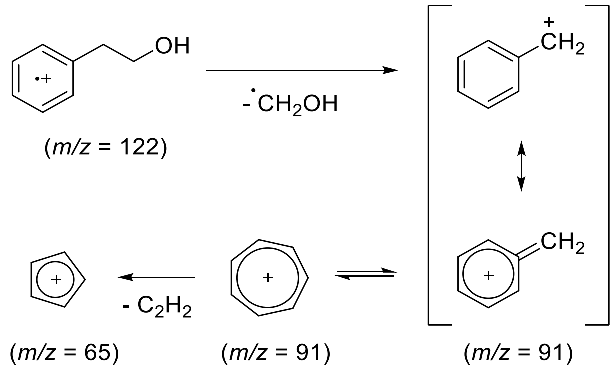

McLafferty rearrangement:

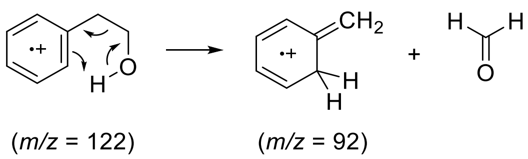

Figure S31: Deuteride migration regioselectivity evidence from MS fragmentation of deuterium labeled anti-Markovnikov products from bioconversions.

Bioconversion of *trans*-styrene-β-*d*:

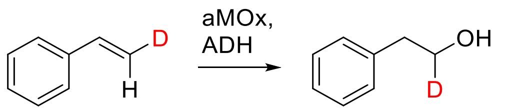

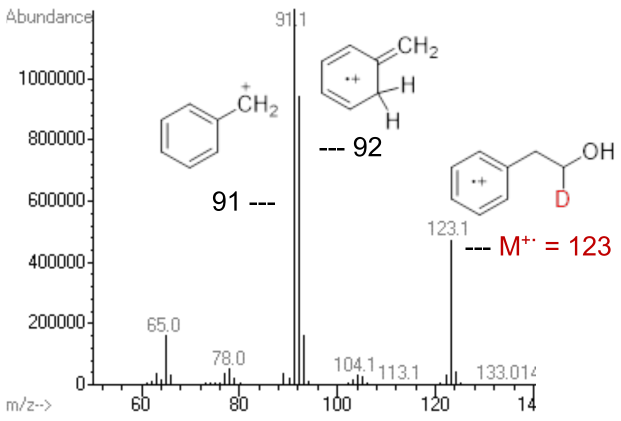

- Molecular ion peak *m/z* = 123 confirms the *d*-label of the product.
- After benzylic cleavage: *m/z* = 91
- McLafferty rearrangement product: *m/z* = 92

→ *d*-label remains untouched if in *trans*-position

Bioconversion of *cis*-styrene-β-*d*:

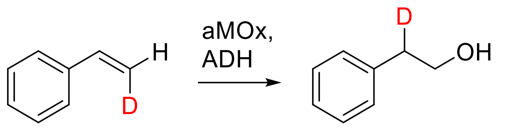

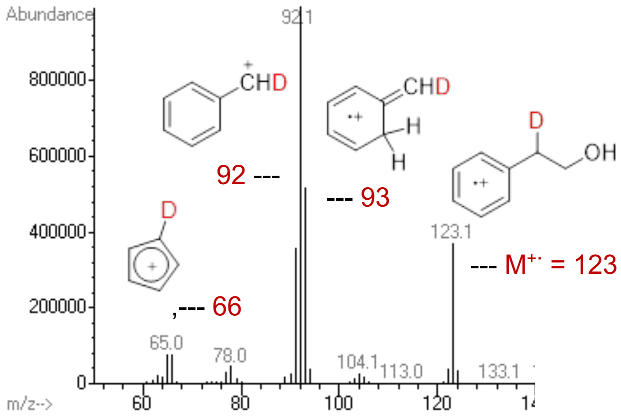

- Molecular ion peak *m/z* = 123 confirms the *d*-label of the product.
- After benzylic cleavage: *m/z* = 92
- McLafferty rearrangement product: *m/z* = 93

→ *d*-label migrates if in *cis*-position

aMOx differentiates between enantiotopic hydrogen atoms → *cis*-selective hydride migration

Both isotopically labeled isomers of styrene are converted to *d*-labeled 2-phenylethanol (molecular ion peak *m/z* = 123) but the mass spectra differ clearly with regard to the fragmentation pattern. In the case of *trans*-styrene-β-*d* as substrate, the fragmentation pattern is similar to that of unlabeled 2-phenylethanol. The ion count of *m/z* = 91 results from a benzylic cleavage product whereas *m/z* = 92 derives from a McLafferty rearrangement product. However, if *cis*-styrene-β-*d* is used as substrate, the ion count of these most prominent fragmentation products increases by one, which indicates that a deuterium atom must have migrated to the benzylic position during the aMOx-catalyzed bioconversion. A residual ion

count of m/z = 91 might suggest, that small quantities of the *trans*-hydride migrated or it originates from remaining quantities of non-labeled substrate.

This experiment confirms, that a high level of catalyst-control provided by aMOx enables the regioselective hydride migration exclusively for the hydride/deuteride in *cis*-position of the styrene substrate and thereby discriminates between enantiotopic hydrogen atoms. Yet, this finding does not provide any information about the expected enantioselectivity of this transformation.

v. MS spectra from isotopic labeling experiment

Table S9: MS spectra of GC separated 2-phenylethanol or 2-phenylethan-2-*d*-1-ol after biotransformation of styrene, *cis*-styrene-β-*d* and *trans*-styrene-β-*d* using $P450_{LA1}$, $P450_{LA1}$-P7 and aMOx. $P450_{LA1}$ was *cis*-selective from the beginning and maintained the selectivity throughout the evolution. This can be derived from identical fragmentation patterns for the product 2-phenylethan-2-*d*-1-ol for $P450_{LA1}$, $P450_{LA1}$-P7 and aMOx. Blue circle: proton, m/z = ±0; red circle: deuterium, m/z = +1.

Directed Evolution

	$P450_{LA1}$	P7	aMOx
**styrene**	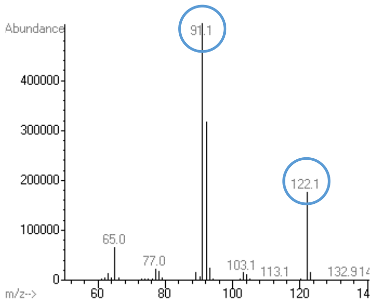	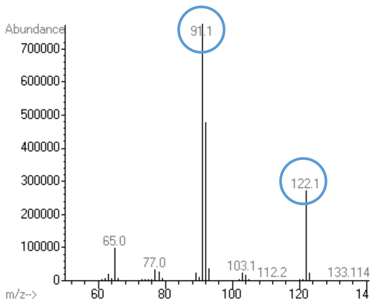	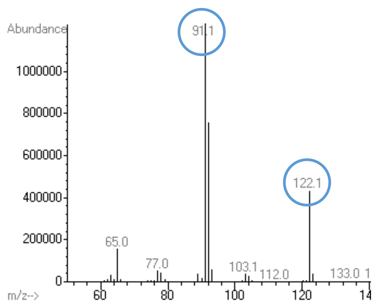
trans*-styrene-β-*d	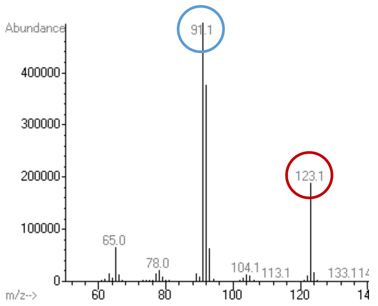	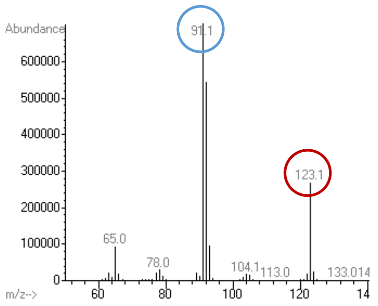	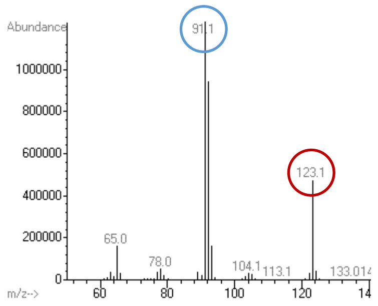
cis*-styrene-β-*d	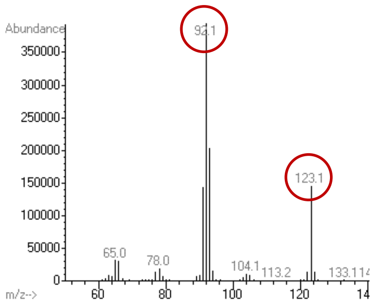	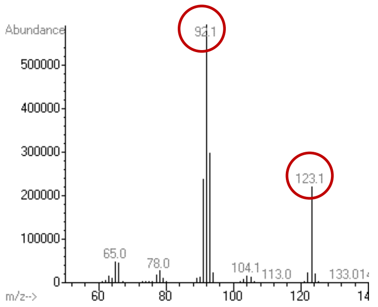	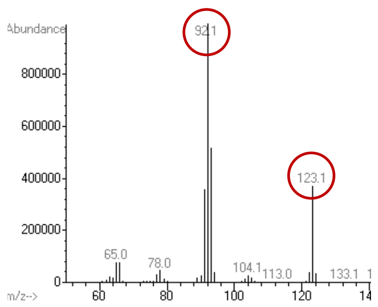

Table S10: MS spectra of GC separated 2-phenyloxirane or 2-phenyloxirane-3-*d* after biotransformation of styrene, *cis*-styrene-β-*d* and *trans*-styrene-β-*d* using P450$_{LA1}$, P450$_{LA1}$-P7 and aMOx. For the isotopically labeled epoxidation product, the fragmentation pattern is identical for both scenarios as expected. Blue circle: proton, *m/z* = ±0; red circle: deuterium, m/z = +1.

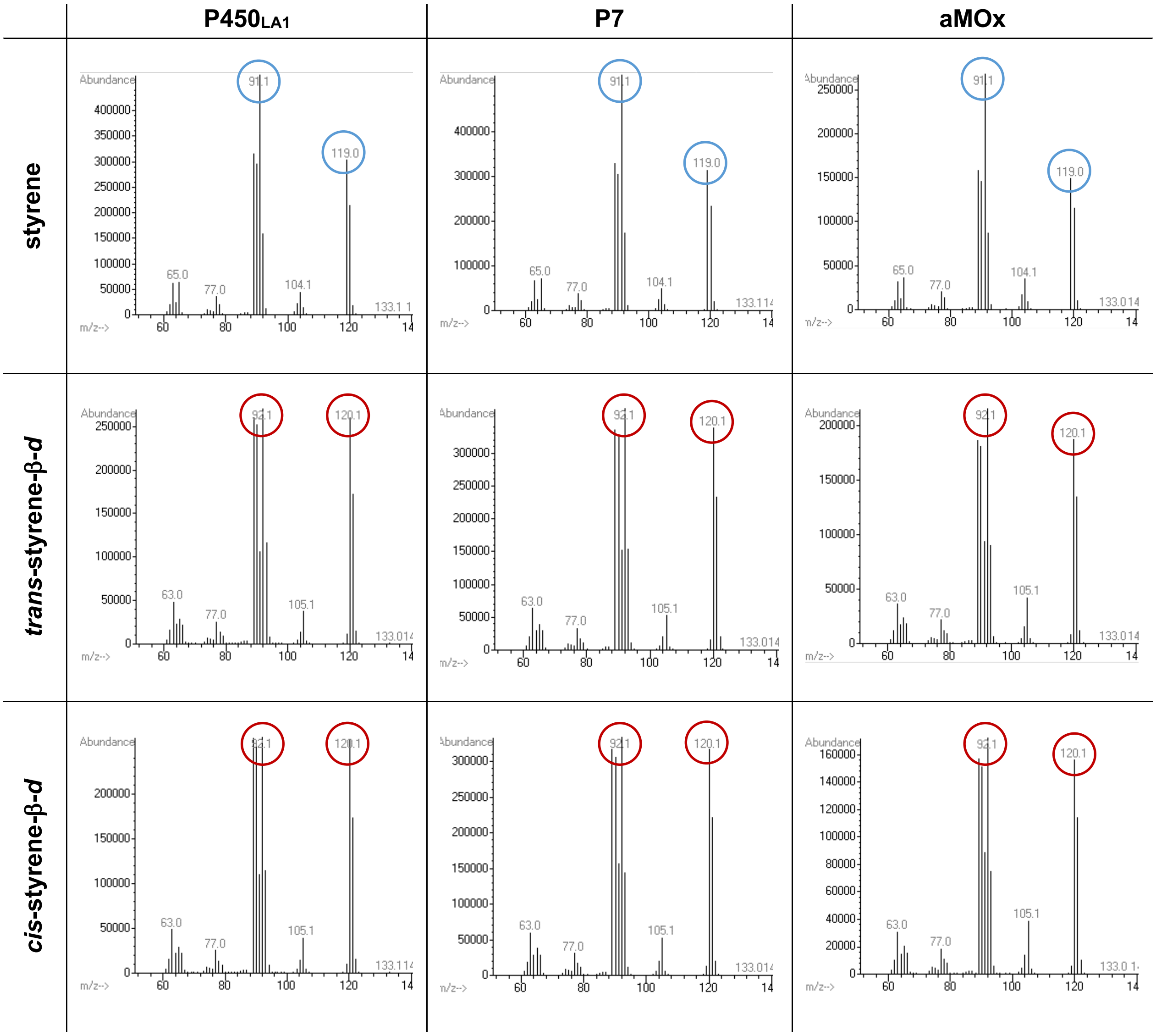

Figure S32: GC-MS chromatogram after biotransformation of *cis*-styrene-β-*d* with P450$_{LA1}$ (black), P450$_{LA1}$-P7 (red) and aMOx (blue) combined with PAR-ADH.

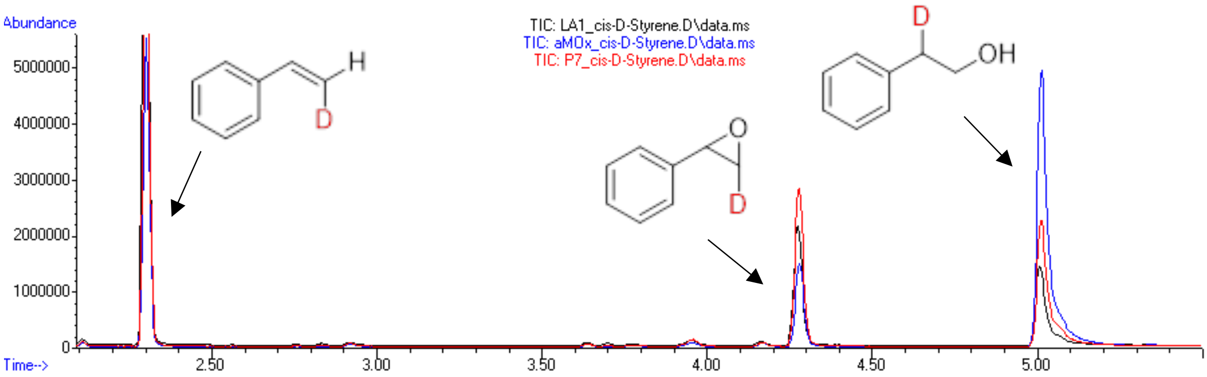

VI. Chemical and enzymatic synthesis of isotopically labeled 2-phenylethan-2-*d*-1-ol

Reactions:

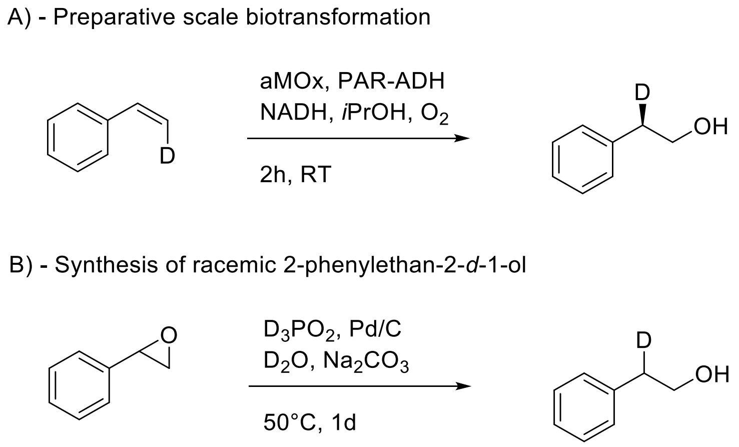

Scheme 2: A) Bioconversion of *cis*-styrene-β-*d* with aMOx and PAR-ADH on preparative scale, B) chemical synthesis of racemic 2-phenylethan-2-*d*-1-ol .

Setup A – Preparative scale bioconversion of *cis*-styrene-β-*d* with aMOx and PAR-ADH

Cell free aMOx lysate (37 mL, 2.5 µM final conc., 0.0625 mol% catalyst) and cell free PAR-ADH lysate (25 mL) were mixed in a 500 mL Schott flask together with NADH (2 mM final conc., 20 mL of a 200 mM stock solution) and *cis*-Styrene-β-*d* (0.4 mmol, 2 mM final conc., 2 mL of a 200 mM stock solution in isopropanol) in a reaction buffer system (116 mL, 100 mM sodium phosphate, 150 mM NaCl, 2% glycerol, pH 8.0). The reaction mixture was incubated for 2 h at room temperature (180 rpm). After extraction with MTBE (3 × 100 mL), the phases were separated by centrifugation. The combined organic extracts were washed with water (100 mL) and brine (100 mL) and dried over $MgSO_4$, filtered, concentrated and purified by column chromatography (10 - 20% ethyl acetate/cyclohexane). By removing the epoxidation product, 20.0 mg (40.7%) of predominantly (*R*)-2-phenylethan-2-*d*-1-ol were isolated.

(*R*)-2-phenylethan-2-*d*-1-ol from biotransformation: ^{1}H NMR (500 MHz, $CDCl_3$) δ = 7.32–7.29 (m, 2H), 7.24–7.21 (m, 3H), 3.85 (br d, J = 6.4 Hz, 2H), 2.86 (m, 1H) ppm. ^{13}C NMR (125 MHz, $CDCl_3$) δ = 138.4, 129.0, 128.6, 126.5, 63.6, 38.8 (t, J = 19.5 Hz) ppm.

^{1}H NMR (500 MHz, $CDCl_3$)

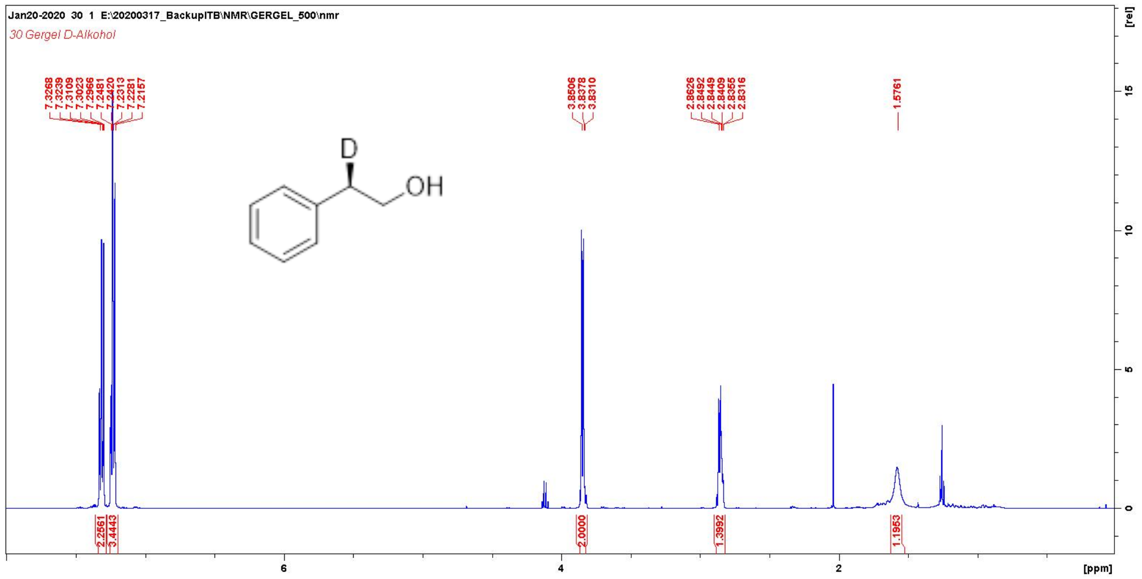

^{13}C NMR (125 MHz, $CDCl_3$)

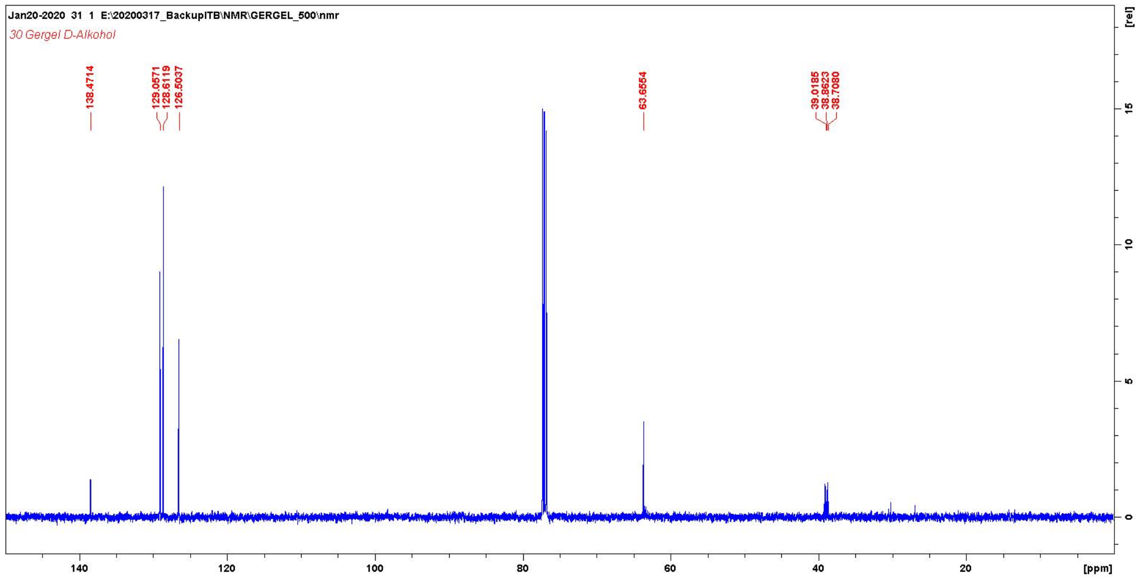

Setup B – Chemical synthesis of racemic 2-phenylethan-2-*d*-1-ol

Racemic 2-phenylethan-2-*d*-1-ol was synthesized according to literature.[74] A solution of D_3PO_2 (6.00 mmol, 3 eq.) in D_2O (12 mL) was added to a mixture of styrene oxide (0.24 g, 2.00 mmol, 1 eq.), sodium carbonate (0.64 g, 6.00 mmol, 3 eq.) and 10% Pd/C (35.4 mg). The mixture was stirred over night at 50°C. After cooling, the mixture was acidified with aqueous HCl (1 M), taken up in dichloromethane (100 mL) and filtered through celite. The organic phase was separated, dried over $MgSO_4$, filtered and concentrated to yield 2-phenylethan-2-*d*-1-ol (49%, 120 mg, 0.97 mmol).

(*rac*)-2-phenylethan-2-*d*-1-ol: ^{1}H NMR (500 MHz, $CDCl_3$) δ = 7.33–7.30 (m, 2H), 7.25–7.22 (m, 3H), 3.86 (br d, *J* = 6.4 Hz, 2H), 2.86 (m, 1H) ppm. ^{13}C NMR (125 MHz, $CDCl_3$) δ = 138.4, 129.0, 128.6, 126.5, 63.6, 38.8 (t, *J* = 19.5 Hz) ppm.

^{1}H NMR (500 MHz, CDCl3)

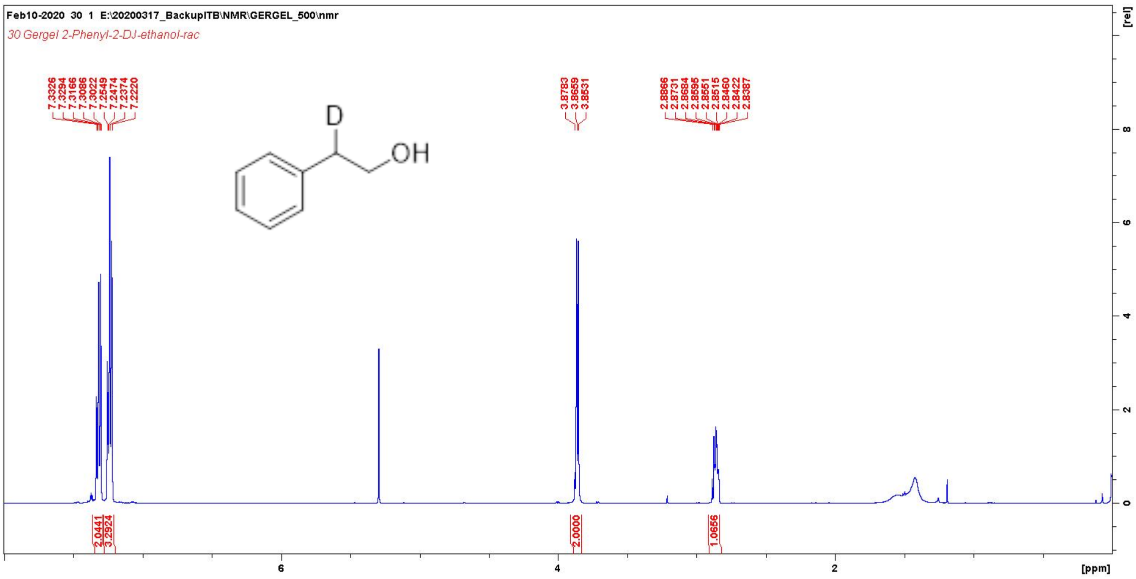

^{13}C NMR (125 MHz, $CDCl_3$)

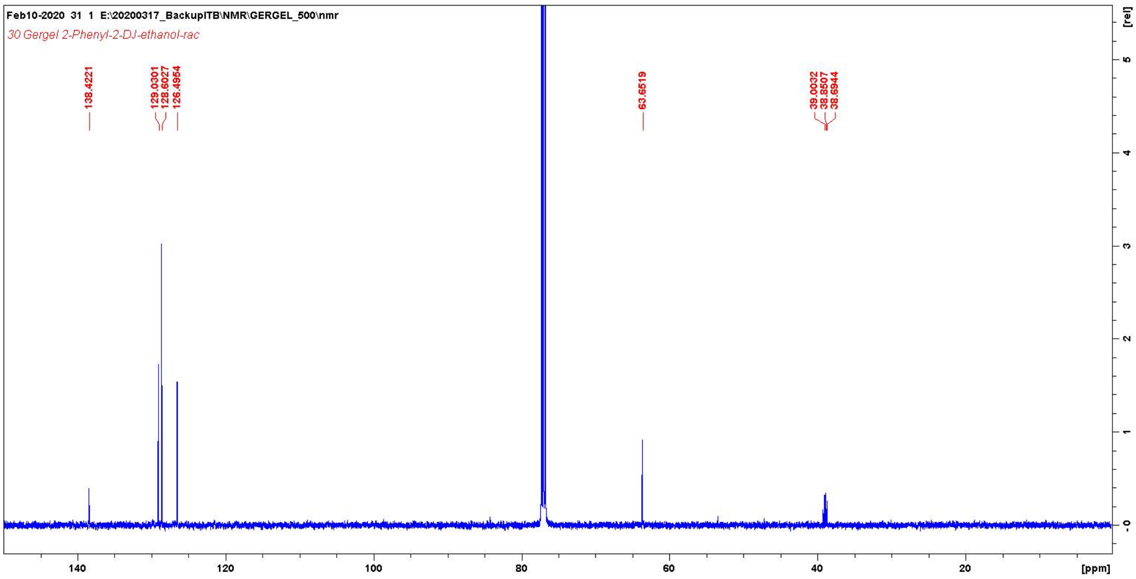

VII. NMR-analysis to determine the enantioselectivity

Question: Is the hydride migration controlled by aMOx also enantioselective?

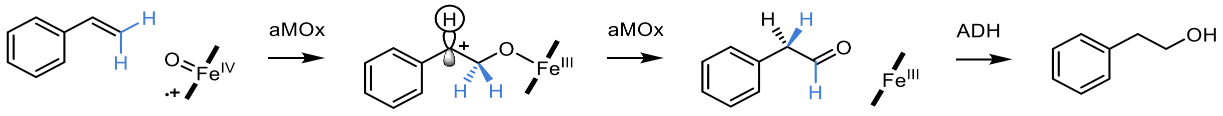

Approach: *cis*-styrene-β-*d* was converted by aMOx and PAR-ADH on preparative scale and the anti-Markovnikov product was isolated and purified after biotransformation (**Section B VI**). The obtained enantioenriched 2-phenylethan-2-*d*-1-ol was esterified with (*S*)-(+)-MTPA-Cl in order to undergo a Mosher ester analysis for the determination of the absolute configuration of the formed *d*-labeled stereogenic center. For comparison, also the mosher esters of (*rac*)-2-phenylethan-2-*d*-1-ol and 2-phenylethanol were synthesized. Although the chemical shifts of generated diastereomeric compounds using the Mosher ester method should differ significantly, this is not the case for compounds with deuterium stereocenters. As shown in **Figure S33-A** (red), the signals of the benzylic protons of the racemic mixture overlap fully and prevent a separated consideration of the diastereomers. For this reason, the spectra have been simplified by decoupling by irradiation of the methylene protons.[75,76] Two baseline separated broad peaks for both diastereomers are the result (**Figure S33-B**, red).

Mosher ester derivatization[75–77]

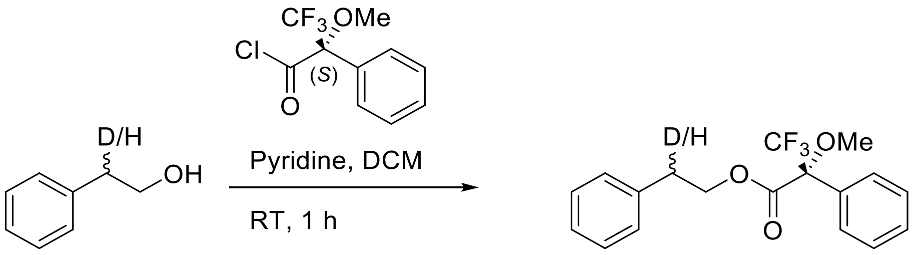

Scheme S3: Mosher ester derivatization of (*R*)-2-phenylethan-2-d-1-ol, (*rac*)-2-phenylethan-2-*d*-1-ol and 2-phenylethanol.

(*R*)-2-phenylethyl-2-*d* (*R*)-3,3,3-trifluoro-2-methoxy-2-phenylpropanoate:

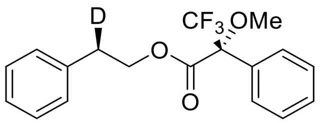

(*S*)-(+)-MTPA-Cl (27.9 µL, 0.15 mmol, 1.9 eq.) was added to a mixture of (*R*)-2-phenylethan-2-*d*-1-ol (9.66 mg, 0.08 mmol, 1 eq.) and pyridine (19.6 µL, 24.3 mmol, 3.1 eq.) in DCM (1 mL) and stirred for 1 h at room temperature. The reaction mixture was directly purified by prep-TLC on silica gel (9:1 cyclohexane/ethyl acetate). Scratching off the spot with the highest retention factor (R_f), extraction with DMC, filtering and concentrating *in vacuo* yielded the (*R*)-*d*-mosher ester (94%, 25.2 mg, 0.07 mmol) as yellow oil.

^{1}H NMR (500 MHz, $CDCl_3$) δ = 7.45–7.43 (m, 2H), 7.42–7.35 (m, 3H), 7.31–7.28 (m, 2H), 7.26–7.24 (m, 1H), 7.21–7.19 (m, 2H), 4.55 (d, *J* = 6.8 Hz, 2H), 3.47 (s, 3H), 3.02 (t, *J* = 6.5 Hz, 1H) ppm.

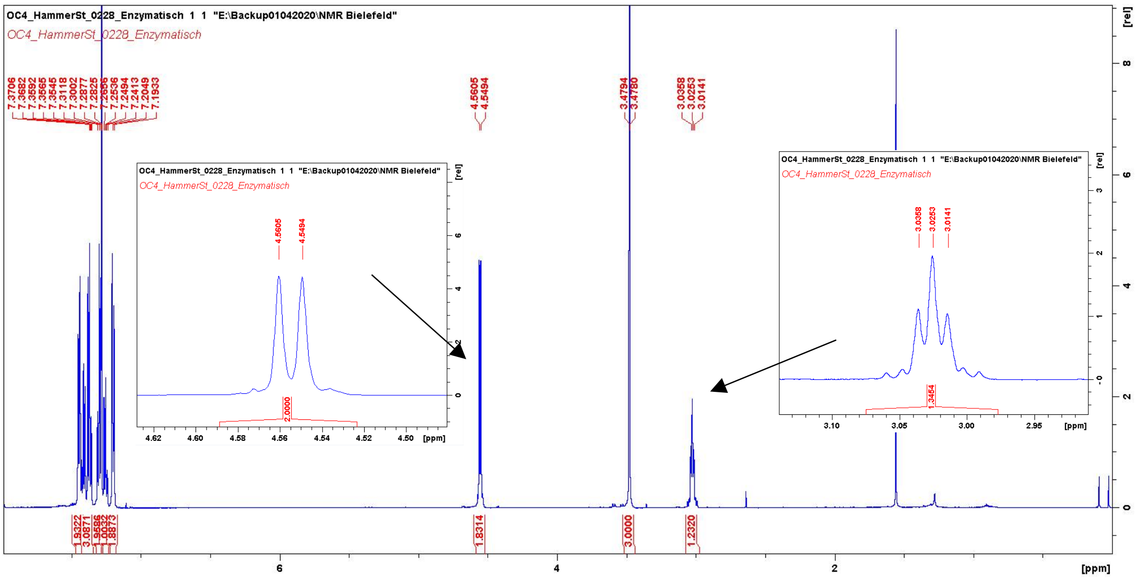

^{1}H NMR (500 MHz, $CDCl_3$) with decoupling by irradiation of the methylene protons (δ = 4.52) using the Bruker zghd.2 standard pulse program.

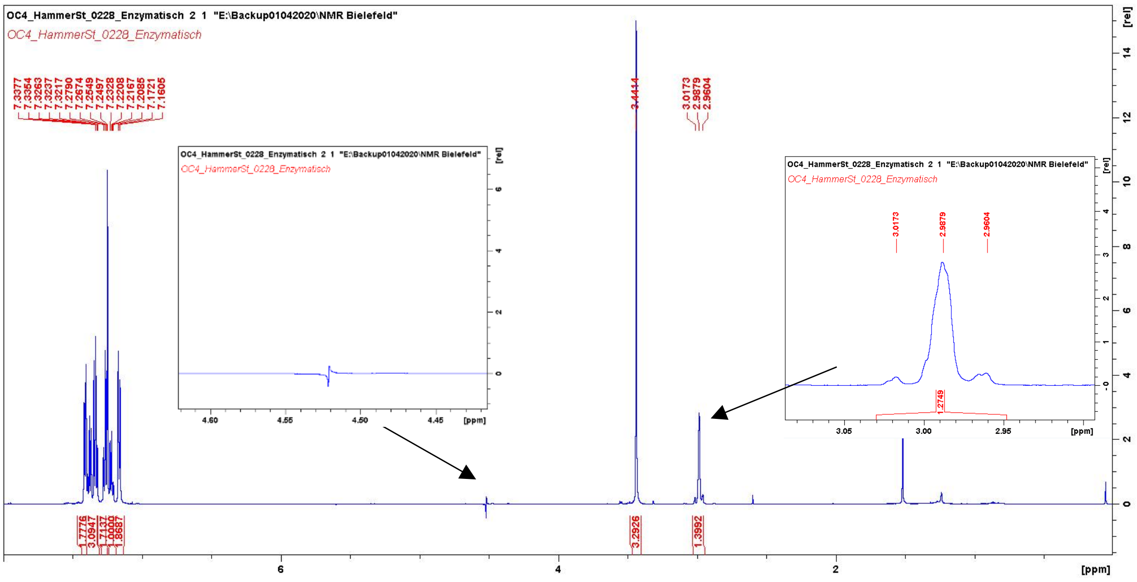

(*rac*)-2-phenylethyl-2-*d* (2*R*)-3,3,3-trifluoro-2-methoxy-2-phenylpropanoate:

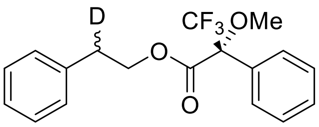

(*S*)-(+)-MTPA-Cl (28.8 µL, 0.15 mmol, 1.9 eq.) was added to a mixture of (*rac*)-2-phenylethan-2-*d*-1-ol (10.0 mg, 0.08 mmol, 1 eq.) and pyridine (20.3 µL, 25.1 mmol, 3.1 eq.) in DCM (1 mL) and stirred for 1 h at room temperature. The reaction mixture was directly purified by prep-TLC on silica gel (9:1 cyclohexane/ethyl acetate). Scratching off the spot with the highest retention factor (R_f), extraction with DMC, filtering and concentrating *in vacuo* yielded the (*rac*)-*d*-Mosher ester (89%, 24.4 mg, 0.07 mmol) as yellow oil.

^{1}H NMR (500 MHz, $CDCl_3$) δ = 7.45–7.43 (m, 2H), 7.42–7.35 (m, 3H), 7.31–7.28 (m, 2H), 7.26–7.24 (m, 1H), 7.21–7.19 (m, 2H), 4.55 (d, *J* = 6.8 Hz, 2H), 3.47 (s, 3H), 3.05–2.98 (m, 1H) ppm.

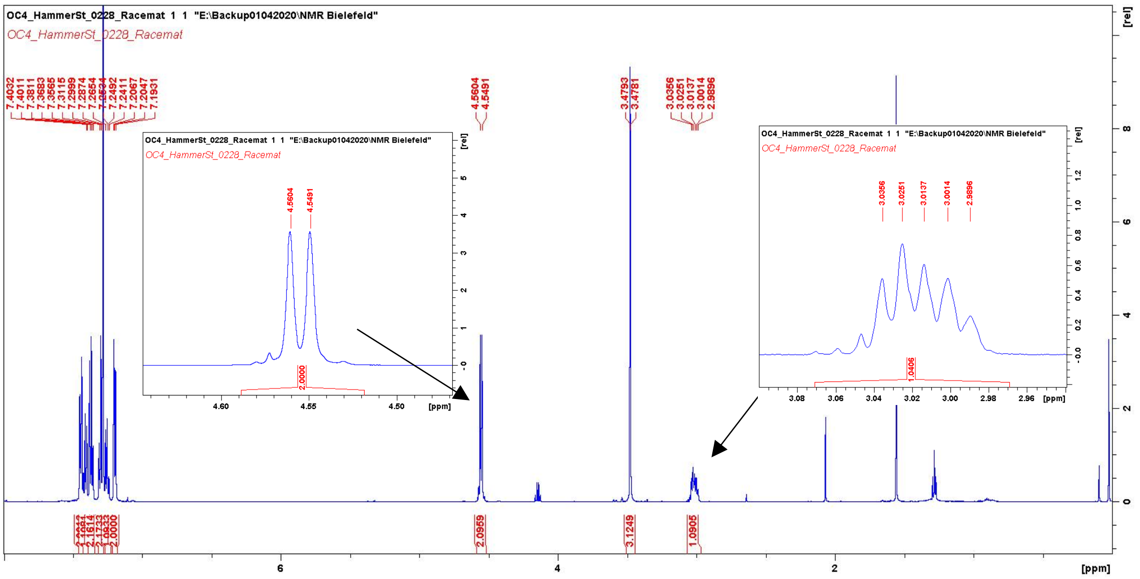

^{1}H NMR (500 MHz, $CDCl_3$) with decoupling by irradiation of the methylene protons (δ = 4.52) using the Bruker zghd.2 standard pulse program.

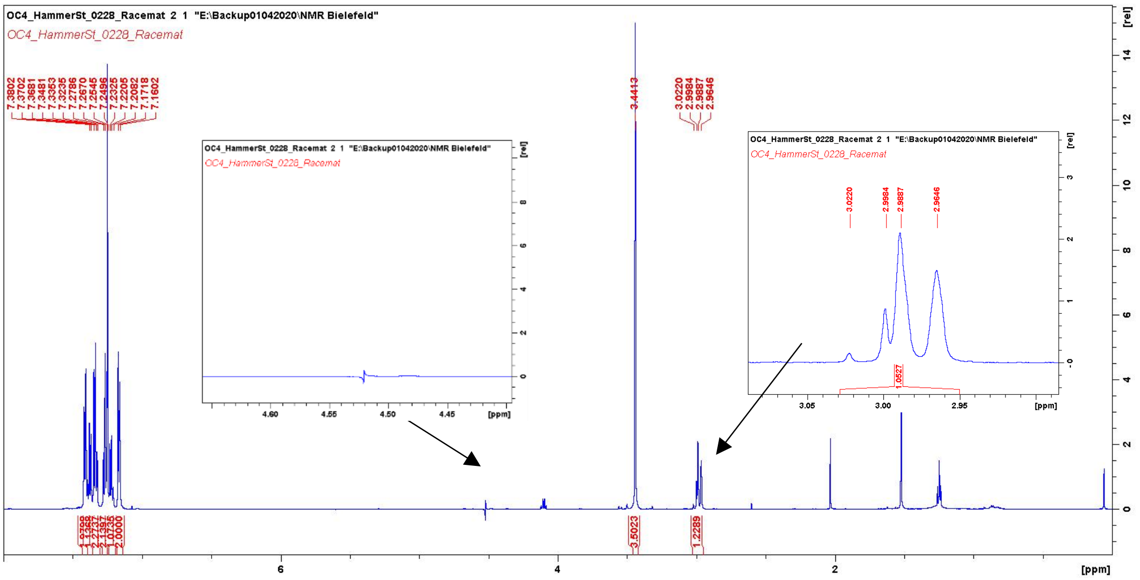

Phenethyl (*R*)-3,3,3-trifuoro-2-methoxy-2-phenylpropanoate:

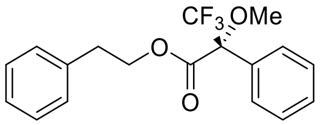

(*S*)-(+)-MTPA-Cl (14.4 µL, 0.08 mmol, 1.9 eq.) was added to a mixture of 2-phenylethanol (5.00 mg, 0.04 mmol, 1 eq.) and pyridine (10.1 µL, 12.6 mmol, 3.1 eq.) in DCM (1 mL) and stirred for 1 h at room temperature. The reaction mixture was directly purified by prep-TLC on silica gel (9:1 cyclohexane/ethyl acetate). Scratching off the spot with the highest retention factor (R_f), extraction with DMC, filtering and concentrating *in vacuo* yielded the mosher ester (>99%, 14.21 mg, 0.04 mmol) as yellow oil.

^{1}H NMR (500 MHz, $CDCl_3$) δ = 7.45–7.43 (m, 2H), 7.42–7.35 (m, 3H), 7.31–7.28 (m, 2H), 7.26–7.24 (m, 1H), 7.21–7.19 (m, 2H), 4.56 (t, *J* = 6.9 Hz, 2H), 3.47 (s, 3H), 3.07–2.99 (m, 2H) ppm.

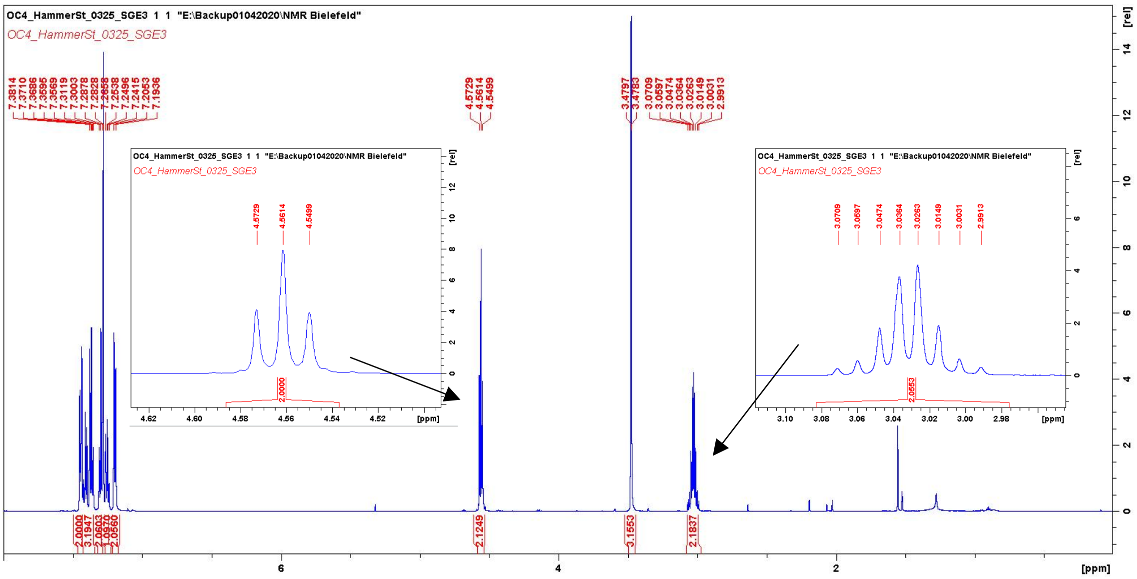

^{1}H NMR (500 MHz, $CDCl_3$) with decoupling by irradiation of the methylene protons (δ = 4.52) using the Bruker zghd.2 standard pulse program.

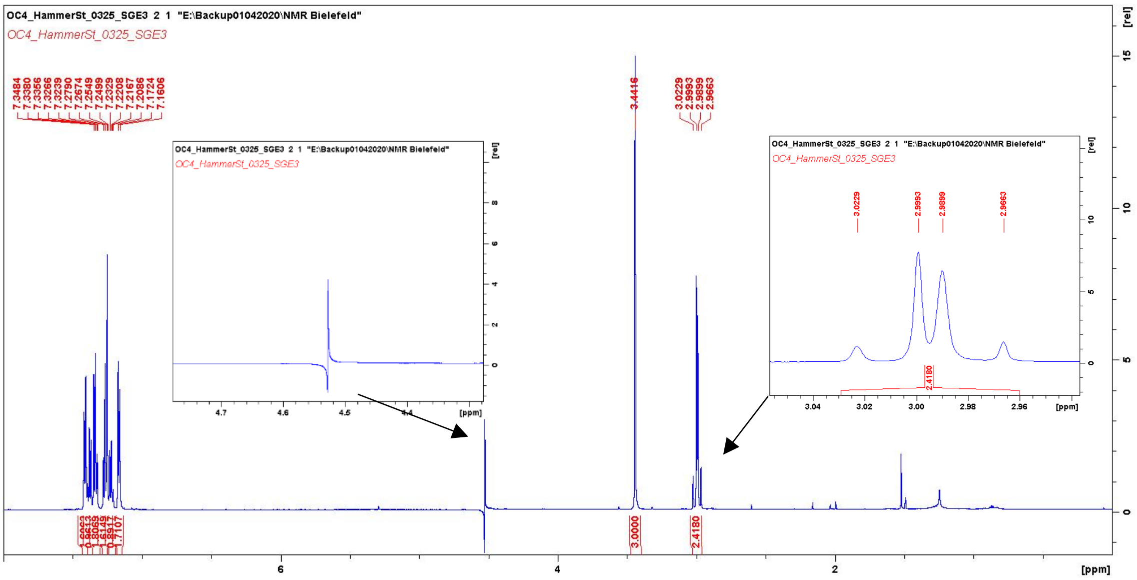

Figure S33: Hydride migration enantioselectivity evidence from NMR analysis.

A – Benzylic protons

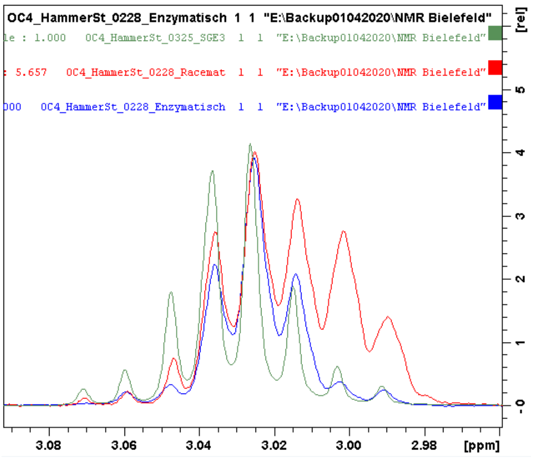

B – Benzylic protons after decoupling

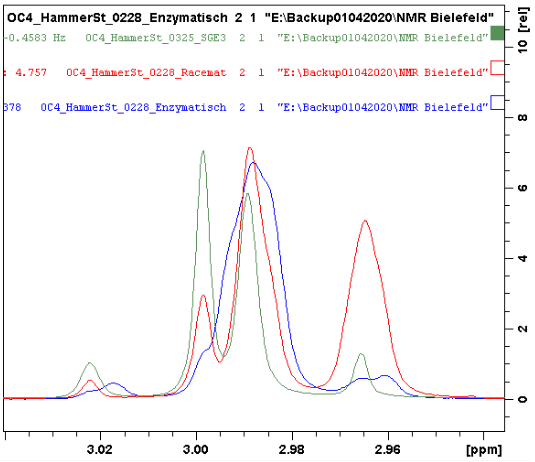

(A) Overlap of proton NMR spectra showing the signal of β-deuterated (*R*)-enantioenriched (blue) and racemic (red) 2-phenylethanol as well as the benzylic protons of non-deuterated 2-phenylethanol (green). (B) Overlaped spectra after decoupling by irradiation of the methylene protons (δ = 4.52).

C – Biotransformation

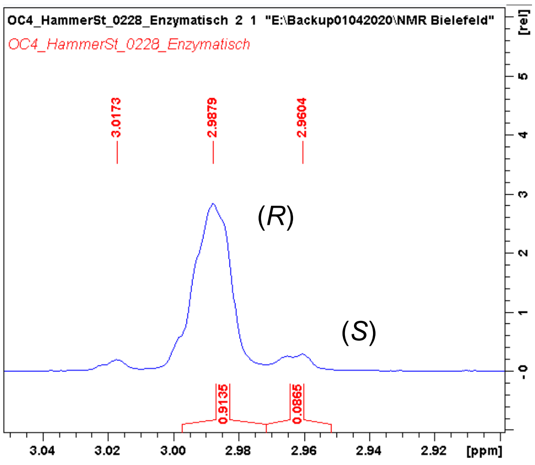

D – Racemate

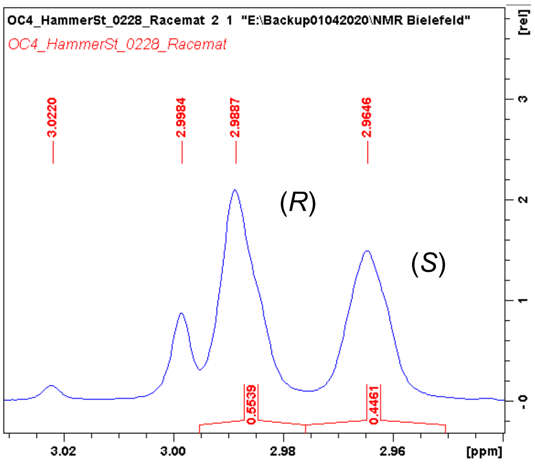

91(*R*):9(*S*)

55(*R*):45(*S*)

Both the sample from the biotransformation (C) and the synthesized racemate (D) contain considerable amounts of the undeuterated compound (compare to green in Fig. A + B). It is believed that this species loses its deuterium label through keto-enol tautomerism of the aldehyde before being reduced to the corresponding alcohol. This impurity was neglected as far as possible in the integration of both signals. Comparison with literature data[75,76] reveals that the aMOx-catalyzed deuteride migration yields predominatly the *R*-enantiomer with an enantiomeric ratio of *de* = >90(*R*):10(*S*). This confirms that aMOx provides a high level of enantiocontrol over the hydride/deuteride migration.

Figure S34: Hydride migration enantioselectivity for styrene and α-methylstyrene.

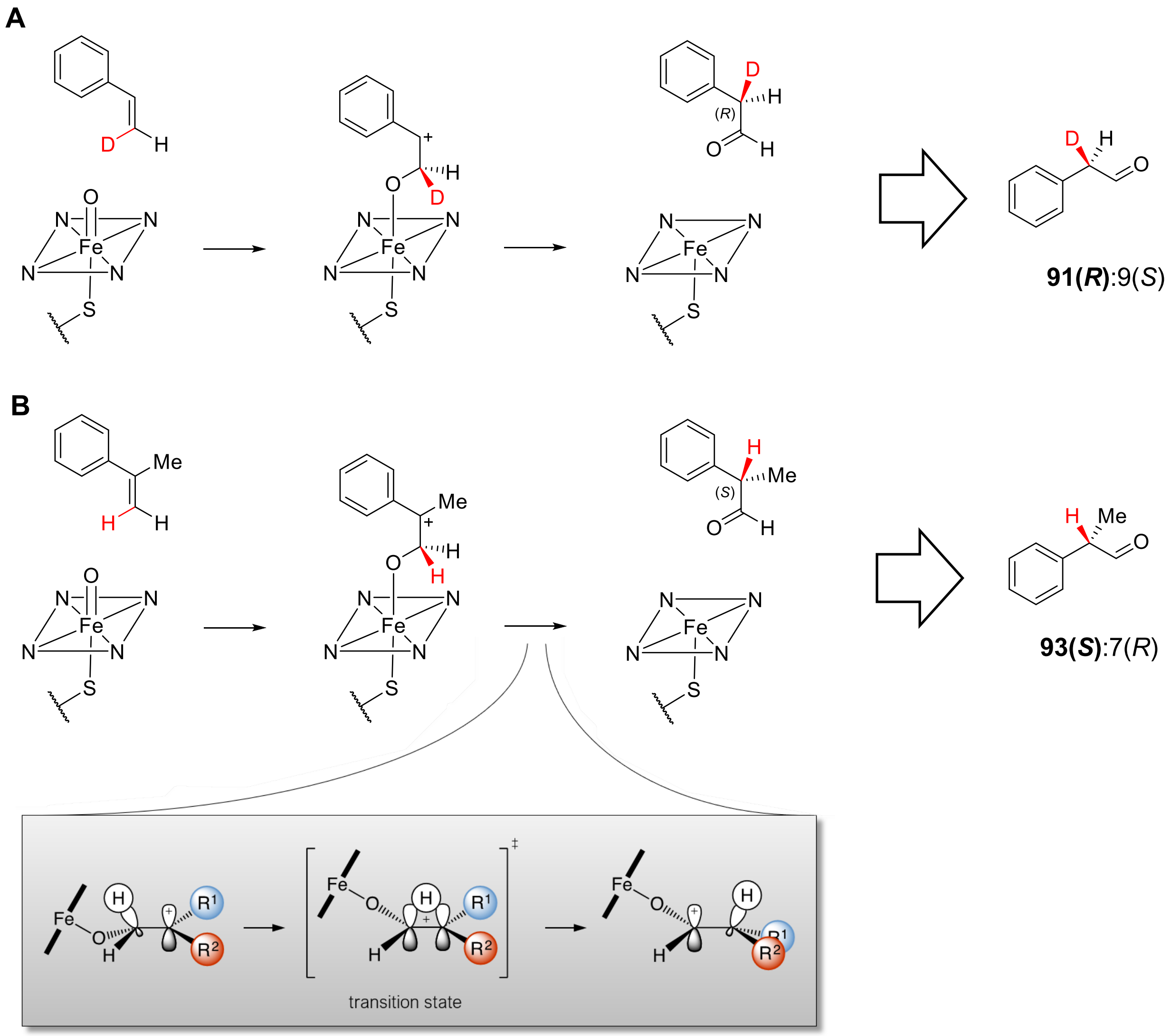

Enantioselectivity was determined in different ways for styrene (**A**) (**Figure S33** and **Figure S35**), and α-methylstyrene (**B**), see previous paper[69]), but in both cases the same selectivity was determined. The hydride/deuteride migration occurs selectively on the *re*-side after proper positioning of the substrate in the active site (see **Figure S21**). This also confirms the postulated orbital alignment (grey box), where only the *cis*-substituent is able to migrate because of the favored orbital overlap of the empty p_z-orbital.

VIII. Enantioselectivity in epoxidation as a function of evolution

Figure S35: Enantioselectivity in the epoxidation as a function of evolution.

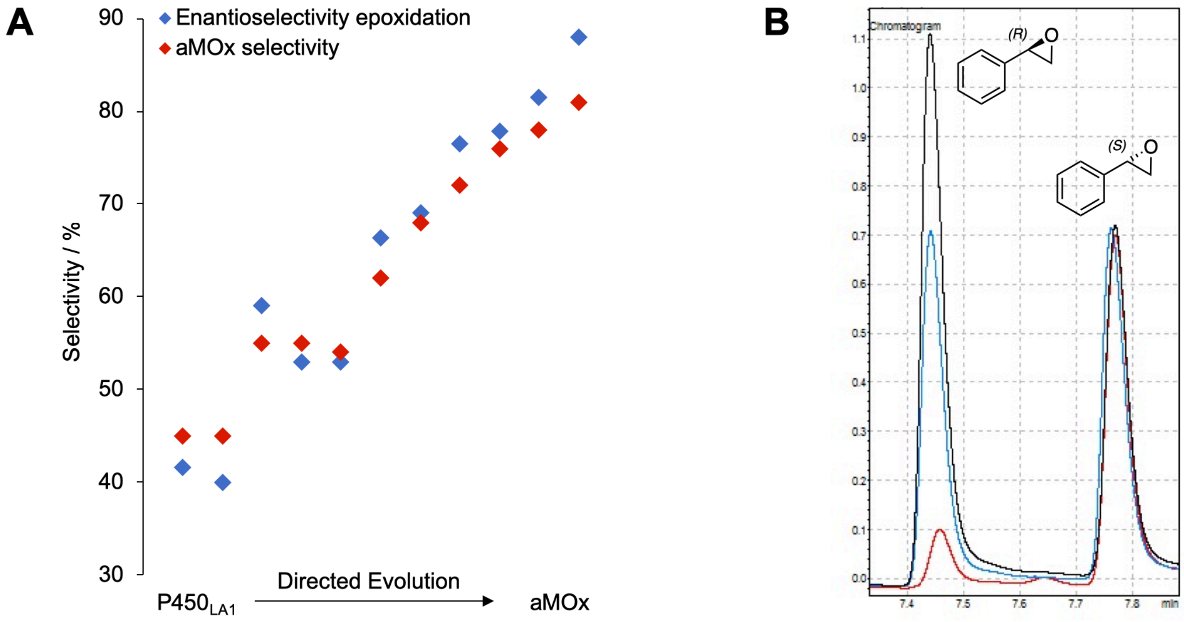

(A) Enantioselectivity in the epoxidation as a function of directed evolution. Interestingly, the enantioselectivity in epoxidation changes during evolution stepwise in analogy to the aMOx selectivity. While wildtype $P450_{LA1}$ generates preferably the (*R*)-enantiomer (60:40 of *R*:*S*), epoxidation gets more and more (*S*)-selective during directed evolution and reaches a ratio of 14:86 (*R*:*S*) for aMOx. (B) Chiral-GC chromatograms of selected variants: $P450_{LA1}$ (black), P7 (blue) and aMOx (red).

IX. UV/Vis spectroscopic analysis of substrate binding

Figure S36: UV/Vis spectroscopic analysis of substrate binding.

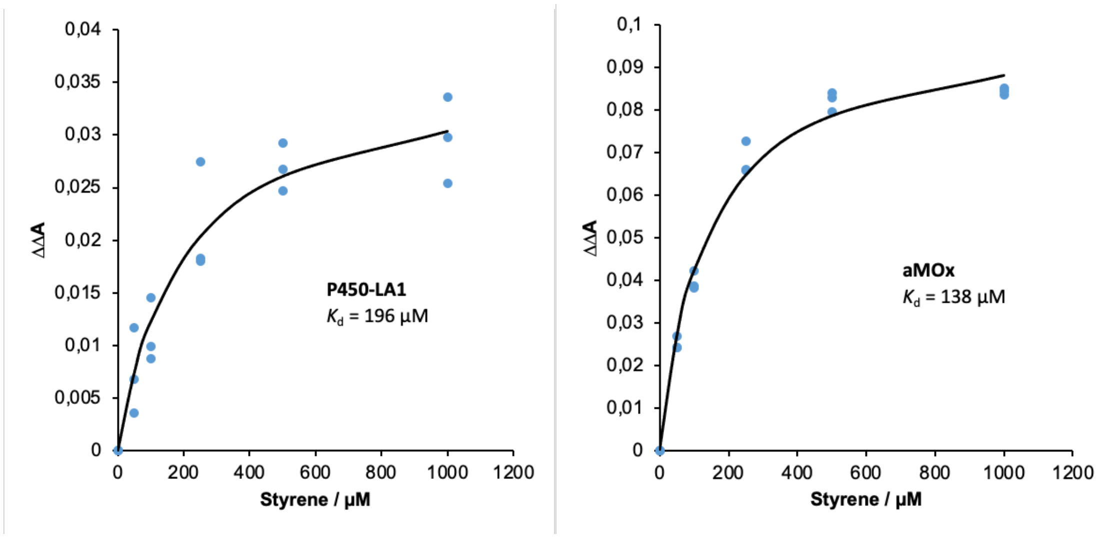

The dissociation constant of styrene was determined by UV/Vis difference spectroscopy at different substrate concentrations. The obtained difference spectra support type I binding in which heme bound water is displaced by productive substrate binding. The determined dissociation constant decreases from 196 µM for $P450_{LA1}$ to 136 µM for aMOx during the directed evolution, which supports the optimization of the active site for styrene binding (see general procedures in the experimental section of the SI details). Please consider that this data should be treated with caution, as styrene has a solubility limit of approximately 2 mM under these conditions.

X. Michealis-Menten kinetics

Figure S37: Michealis-Menten kinetics.

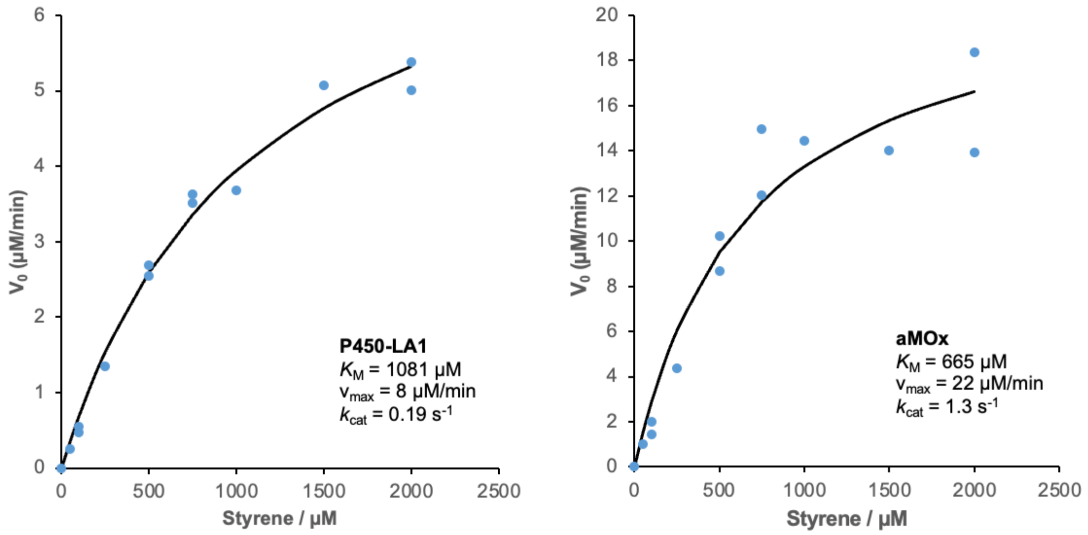

The kinetic parameters for the $P450_{LA1}$ and aMOx catalyzed reaction with styrene were calculated by determining the initial velocities at different substrate concentrations (see general procedures in the experimental section of the SI details). During the directed evolution K_M decreased 1.6-fold while k_{cat} increased 6.8-fold. The resulting catalytic efficiency of aMOx (2.0×10^3 M^{-1} s^{-1}) is one order of magnitude higher than the efficiency of $P450_{LA1}$. The decrease of K_M during evolution is in accordance with improved substrate binding to aMOx. Please consider that this data should be treated with caution, as styrene has a solubility limit of approximately 2 mM under these conditions.

XI. Coupling efficiencies

Figure S38: Coupling efficiencies.

Enzyme	Coupling efficiency / %	Uncoupling to H_2O_2 / %
$P450_{LA1}$	6.5	3.9
aMOx	26	4.6

The coupling efficiencies of $P450_{LA1}$ and aMOx were determined using styrene as the substrate by relating NADH consumption to product formation. During directed evolution the coupling efficiency increases significantly, which might result from improved substrate binding and contribute to the increased k_{cat}. However, uncoupling via the peroxide shunt (H_2O_2 formation) is not observed, suggesting that the active site is not water accessible but that there might be uncoupling via the oxidase shunt.

C. Optimized structures (cartesian coordinates) of characterized stationary points

Table S11: Cartesian coordinates (xyz, in Å) of all DFT optimized stationary points reported in **Figures S1** to **S2** and **S4** and **Tables S1** and **S3**.

[1 + Cpd I]^{d} (reactant complex)

Fe	0.925842	0.031903	0.092113
N	2.200204	1.434344	-0.612806
N	1.787169	-1.325214	-1.140866
N	0.340850	1.299830	1.543398
N	-0.089762	-1.455600	0.997032
C	2.305031	2.738653	-0.188337
C	1.466910	-2.661108	-1.251631
C	3.021851	1.329378	-1.707520
C	2.671545	-1.065028	-2.169977
C	0.698712	2.616762	1.682202
C	-0.171768	-2.764106	0.593083
C	-0.588404	1.058601	2.523849
C	-0.972050	-1.330102	2.045880
C	3.223675	3.466172	-1.029127
C	2.187965	-3.257993	-2.349027
C	3.663459	2.592839	-1.976598
C	2.928294	-2.268934	-2.920061
C	-0.018438	3.219866	2.778481
C	-1.117622	-3.484511	1.409538
C	-0.821802	2.251349	3.299848
C	-1.615348	-2.592995	2.311750
H	3.483677	4.508482	-0.896901
H	2.116834	-4.299424	-2.635174
H	4.362404	2.766385	-2.784548
H	3.594152	-2.329687	-3.771173
H	0.091970	4.249566	3.093081
H	-1.353011	-4.534843	1.295834
H	-1.509244	2.317915	4.133125
H	-2.345189	-2.757680	3.093819
C	1.619418	3.289160	0.884994
C	0.559795	-3.333630	-0.445662
C	-1.207541	-0.163792	2.759689
C	3.233569	0.169888	-2.446247
H	1.804184	4.334430	1.111636
H	0.395424	-4.386608	-0.651228
H	-1.927780	-0.210823	3.570267
H	3.909266	0.233951	-3.293077
O	-0.273820	0.412030	-0.939553
S	3.086033	-0.660948	1.416508
C	2.863795	-2.314049	2.140818
H	2.718821	-3.048239	1.339124
H	3.771120	-2.583282	2.689729
H	1.997387	-2.346332	2.805774
C	-2.835347	2.715130	-1.968226
H	-2.012674	2.089900	-1.629435
H	-2.571710	3.693430	-2.360866
C	-4.117877	2.327193	-1.918868
H	-4.880112	3.018254	-2.280656
C	-4.646320	1.044337	-1.421544
C	-6.036221	0.829517	-1.442977
C	-3.822715	0.015607	-0.921592
C	-6.591440	-0.365317	-0.982074
H	-6.686989	1.612400	-1.826568
C	-4.377517	-1.176578	-0.460696
H	-2.744140	0.146489	-0.893718
C	-5.763181	-1.374499	-0.487650
H	-7.668856	-0.506295	-1.009855
H	-3.724948	-1.956275	-0.075764
H	-6.190228	-2.306651	-0.127282

[1 + Cpd I]^{q} (reactant complex)

Fe	0.930808	0.030444	0.086757
N	2.201381	1.436738	-0.614668
N	1.795317	-1.321802	-1.139979
N	0.340788	1.298696	1.541680
N	-0.087283	-1.458064	0.990200
C	2.302868	2.741260	-0.188420
C	1.481845	-2.660472	-1.247895
C	3.027397	1.333998	-1.705953
C	2.687605	-1.062100	-2.163321
C	0.693660	2.616521	1.679415
C	-0.167715	-2.766072	0.586119
C	-0.591122	1.055646	2.518638
C	-0.971550	-1.333165	2.037792
C	3.222588	3.470749	-1.026341
C	2.213543	-3.257558	-2.337696
C	3.667127	2.598773	-1.972822
C	2.954198	-2.267588	-2.906746
C	-0.029319	3.219224	2.772267
C	-1.115793	-3.487269	1.399316
C	-0.831230	2.248760	3.292333
C	-1.615467	-2.596417	2.301103
H	3.480390	4.513418	-0.892641
H	2.148366	-4.300138	-2.621036
H	4.368537	2.773915	-2.778279
H	3.626296	-2.328682	-3.752910
H	0.076244	4.249892	3.085346
H	-1.350676	-4.537581	1.284431
H	-1.522197	2.314362	4.122778
H	-2.346906	-2.761779	3.081549
C	1.614871	3.290456	0.883730
C	0.570643	-3.334635	-0.448515
C	-1.208386	-0.168138	2.752955
C	3.245849	0.173744	-2.441631
H	1.797264	4.336166	1.110250
H	0.410522	-4.388170	-0.654488
H	-1.930192	-0.216659	3.562050
H	3.925602	0.237468	-3.285205
O	-0.280527	0.422111	-0.926806
S	3.080868	-0.653952	1.421238
C	2.859468	-2.306158	2.147841
H	2.717523	-3.042045	1.347158
H	3.766006	-2.572984	2.699228
H	1.991634	-2.338524	2.810890
C	-2.853405	2.720371	-1.955814
H	-2.029067	2.098189	-1.615564
H	-2.592960	3.700665	-2.345548
C	-4.134304	2.326479	-1.911514
H	-4.898417	3.014820	-2.274511
C	-4.658648	1.040298	-1.418488
C	-6.047421	0.818990	-1.445865
C	-3.832250	0.014587	-0.916961
C	-6.598848	-0.379270	-0.989337
H	-6.700311	1.599479	-1.830708
C	-4.383255	-1.181032	-0.460440
H	-2.754453	0.150479	-0.884416
C	-5.767839	-1.385427	-0.493376
H	-7.675463	-0.525256	-1.021683
H	-3.728568	-1.958307	-0.074225
H	-6.191938	-2.320219	-0.136382

TS1^d (conformer 1)

Fe	0.840080	-0.018140	-0.127900
N	1.902920	-1.619920	0.485880
N	2.110990	1.210400	0.864800
N	-0.307180	-1.228350	-1.251300
N	-0.065620	1.598590	-0.927640
C	1.676300	-2.938600	0.175080
C	2.054240	2.579640	0.934500
C	2.967060	-1.611260	1.355070
C	3.130430	0.827250	1.701660
C	-0.240180	-2.598440	-1.336230
C	0.168900	2.916540	-0.618770
C	-1.328410	-0.846260	-2.088400
C	-1.130980	1.594740	-1.792430
C	2.623540	-3.782220	0.861880
C	3.070820	3.075240	1.833600
C	3.422740	-2.958730	1.595500
C	3.737130	1.988490	2.309940
C	-1.256180	-3.092770	-2.234010
C	-0.762840	3.765460	-1.321720
C	-1.931950	-2.005650	-2.699690
C	-1.568990	2.945520	-2.050860
H	2.656970	-4.861130	0.780540
H	3.236060	4.121640	2.056160
H	4.250330	-3.220130	2.242450
H	4.565210	1.954370	3.006330
H	-1.410220	-4.137760	-2.470870
H	-0.782220	4.845520	-1.251560
H	-2.756290	-1.970870	-3.400470
H	-2.390000	3.210300	-2.704780
C	0.677520	-3.402230	-0.672500
C	1.154400	3.381050	0.242840
C	-1.721010	0.461580	-2.338740
C	3.533820	-0.480640	1.931210
H	0.614330	-4.473670	-0.833920
H	1.223430	4.454110	0.391040
H	-2.552630	0.611020	-3.019920
H	4.365270	-0.633640	2.612020
O	-0.167600	0.003640	1.206860
S	2.510850	0.027620	-1.966100
C	2.688310	1.730250	-2.613370
H	3.016160	2.420220	-1.830690
H	3.458710	1.702400	-3.391900
H	1.757460	2.098920	-3.051730
C	-1.435170	-1.457210	2.034720
H	-1.408630	-0.886190	2.954830
H	-0.672220	-2.216770	1.922990
C	-2.551520	-1.492250	1.236420
H	-2.561800	-2.202440	0.412670
C	-3.720210	-0.642250	1.342690
C	-4.794160	-0.846000	0.445440
C	-3.845680	0.387670	2.305420
C	-5.945320	-0.066560	0.511870
H	-4.711470	-1.629160	-0.303900
C	-4.995220	1.165900	2.367100
H	-3.034110	0.579730	3.000000
C	-6.051870	0.942640	1.473900
H	-6.759300	-0.243060	-0.185650
H	-5.072330	1.953070	3.111910
H	-6.948480	1.553560	1.527640

TS1^q (conformer 1)

Fe	0.909060	0.076890	-0.111470
N	1.911340	-1.537000	0.605170
N	2.121670	1.295410	0.918100
N	-0.233420	-1.175470	-1.251730
N	0.051580	1.657860	-0.991960
C	1.669110	-2.860930	0.321650
C	2.124450	2.667530	0.900430
C	2.921590	-1.523430	1.531150
C	3.098780	0.924750	1.811260
C	-0.185140	-2.548700	-1.279930
C	0.326780	2.983250	-0.756540
C	-1.240580	-0.810200	-2.104960
C	-1.007380	1.639290	-1.870780
C	2.560650	-3.701050	1.084070
C	3.119440	3.177720	1.815880
C	3.337560	-2.871310	1.835340
C	3.721080	2.097390	2.382760
C	-1.194360	-3.061550	-2.177520
C	-0.567000	3.820850	-1.519390
C	-1.850650	-1.982830	-2.688850
C	-1.396930	2.987840	-2.205980
H	2.574290	-4.782550	1.038810
H	3.317180	4.228640	1.984220
H	4.122650	-3.129760	2.534470
H	4.518540	2.073820	3.114440
H	-1.360550	-4.112020	-2.379780
H	-0.550920	4.903180	-1.510820
H	-2.667050	-1.963000	-3.399510
H	-2.203170	3.243510	-2.881720
C	0.695460	-3.336710	-0.549860
C	1.298850	3.460490	0.114270
C	-1.613090	0.501140	-2.387090
C	3.473160	-0.379490	2.101800
H	0.617250	-4.413000	-0.667970
H	1.408530	4.536960	0.199450
H	-2.436550	0.647530	-3.079060
H	4.270950	-0.519430	2.824500
O	-0.218450	0.064360	1.164200
S	2.499540	-0.179240	-1.919190
C	1.844290	0.591070	-3.438920
H	1.658440	1.658630	-3.299530
H	2.583920	0.446530	-4.232640
H	0.912210	0.104580	-3.743580
C	-1.397930	-1.390130	1.929370
H	-1.369130	-0.868180	2.878480
H	-0.643550	-2.153380	1.789810
C	-2.556520	-1.444930	1.182260
H	-2.585400	-2.136810	0.343500
C	-3.738370	-0.625030	1.359280
C	-4.857410	-0.849080	0.523500
C	-3.837700	0.395120	2.335530
C	-6.022850	-0.100890	0.661580
H	-4.798010	-1.624380	-0.236530
C	-5.002520	1.142410	2.469140
H	-2.993040	0.605460	2.984090
C	-6.102570	0.898850	1.636450
H	-6.869810	-0.294530	0.008950
H	-5.056370	1.922210	3.223990
H	-7.010300	1.485570	1.745680

TS1^d (conformer 2)

Fe	0.885360	0.043199	0.099993
N	1.559433	-1.836812	-0.189529
N	-0.321097	-0.621111	1.565542
N	2.277761	0.731103	-1.195651
N	0.359215	1.945454	0.536335
C	2.501791	-2.242588	-1.102472
C	-1.215571	0.125004	2.294830
C	1.156383	-2.972717	0.469484
C	-0.477677	-1.921968	1.986640
C	3.111492	-0.021470	-1.984575
C	-0.637838	2.346151	1.391239
C	2.499147	2.040326	-1.545762
C	0.835987	3.088813	-0.057634
C	2.685261	-3.672246	-1.027741
C	-1.953087	-0.724990	3.196649
C	1.854561	-4.124490	-0.048254
C	-1.491404	-1.993649	3.009712
C	3.886076	0.836112	-2.849910
C	-0.789581	3.781442	1.340588
C	3.508770	2.114878	-2.575147
C	0.126892	4.241704	0.445739
H	3.373993	-4.235551	-1.644298
H	-2.715029	-0.377347	3.882522
H	1.715652	-5.137488	0.307114
H	-1.798101	-2.904897	3.507040
H	4.619694	0.485642	-3.564579
H	-1.506751	4.343880	1.924675
H	3.865445	3.035604	-3.018792
H	0.318660	5.261683	0.138108
C	3.215364	-1.406104	-1.952022
C	-1.380596	1.502539	2.206534
C	1.837901	3.141175	-1.016859
C	0.211970	-3.019558	1.486908
H	3.927041	-1.869991	-2.627554
H	-2.134847	1.956356	2.841449
H	2.114547	4.119395	-1.397025
H	-0.013032	-3.990060	1.917645
O	-0.274910	0.002954	-1.113254
S	2.720083	0.374360	1.762805
C	1.996566	0.758329	3.397759
H	1.393366	-0.068184	3.781474
H	2.830384	0.937862	4.085597
H	1.385029	1.664177	3.357753
C	-1.503137	-1.604496	-1.683603
H	-1.604906	-1.926306	-0.655173
H	-0.674566	-2.030514	-2.235002
C	-2.539980	-1.010461	-2.360220
H	-2.412567	-0.833246	-3.426834
C	-3.790730	-0.544803	-1.795252
C	-4.762920	0.013435	-2.657394
C	-4.097834	-0.628210	-0.415589
C	-5.986714	0.460990	-2.169483
H	-4.542980	0.088659	-3.719419
C	-5.321009	-0.178785	0.068196
H	-3.372036	-1.041897	0.277001
C	-6.272683	0.366347	-0.803526
H	-6.718518	0.884672	-2.851539
H	-5.536727	-0.249757	1.130739
H	-7.226769	0.716022	-0.419467

TS1^q (conformer 2)

Fe	0.905660	0.118410	0.064450
N	1.859090	-1.456900	-0.795850
N	0.323100	-1.103470	1.589560
N	1.614210	1.332610	-1.366010
N	0.138360	1.691420	1.034240
C	2.542770	-1.445620	-1.983430
C	-0.446670	-0.752850	2.667620
C	1.887830	-2.756840	-0.349420
C	0.561850	-2.452920	1.711390
C	2.325690	0.963880	-2.482810
C	-0.618100	1.664230	2.184060
C	1.450680	2.692190	-1.451140
C	0.167980	3.004850	0.630340
C	3.022720	-2.770640	-2.295320
C	-0.703750	-1.909050	3.494840
C	2.617340	-3.583930	-1.280380
C	-0.074850	-2.962200	2.902930
C	2.605510	2.123120	-3.298740
C	-1.064860	2.995920	2.515300
C	2.066950	3.195210	-2.657130
C	-0.572810	3.827490	1.556180
H	3.594520	-3.025540	-3.178470
H	-1.285730	-1.895550	4.407560
H	2.785610	-4.646190	-1.157380
H	-0.035280	-3.994210	3.227410
H	3.152050	2.098580	-4.232860
H	-1.672930	3.242600	3.376280
H	2.075720	4.236140	-2.954060
H	-0.694910	4.899000	1.462980
C	2.755720	-0.322330	-2.777520
C	-0.900310	0.535070	2.941230
C	0.788790	3.479760	-0.518120
C	1.287340	-3.225300	0.813960
H	3.317630	-0.461520	-3.695800
H	-1.507480	0.671490	3.830740
H	0.733520	4.546320	-0.711780
H	1.389960	-4.282980	1.035760
O	-0.520590	-0.146670	-0.817310
S	2.990280	0.247280	1.316410
C	2.690020	1.058640	2.924130
H	1.971990	0.483180	3.516950
H	3.640610	1.084530	3.466490
H	2.312900	2.076190	2.797410
C	-1.705010	-1.812300	-0.887670
H	-1.829100	-1.804540	0.187770
H	-0.886600	-2.413350	-1.262780
C	-2.743060	-1.484530	-1.733000
H	-2.611760	-1.680640	-2.795870
C	-3.991070	-0.847970	-1.363220
C	-4.968850	-0.625990	-2.360840
C	-4.290220	-0.437680	-0.041660
C	-6.189310	-0.031430	-2.055090
H	-4.755680	-0.930300	-3.382700
C	-5.509570	0.158430	0.259590
H	-3.557930	-0.580380	0.746910
C	-6.467090	0.363600	-0.742360
H	-6.924830	0.126570	-2.839050
H	-5.718080	0.468800	1.279890
H	-7.418340	0.829410	-0.501010

Int1[q]

Fe	-0.858760	0.051050	-0.261280
N	-1.698730	-1.461430	0.758260
N	-1.796770	1.373990	0.927230
N	0.029670	-1.266320	-1.498350
N	-0.055290	1.572500	-1.321930
C	-1.613840	-2.802880	0.462860
C	-1.778360	2.743050	0.812510
C	-2.451020	-1.364110	1.905670
C	-2.527700	1.090250	2.057490
C	-0.127850	-2.634000	-1.492610
C	-0.279370	2.913990	-1.132140
C	0.902570	-0.981670	-2.521220
C	0.836120	1.473100	-2.361780
C	-2.336930	-3.567930	1.447020
C	-2.531340	3.336990	1.889620
C	-2.845710	-2.678540	2.345980
C	-2.985990	2.314250	2.666550
C	0.669010	-3.223710	-2.538460
C	0.491650	3.681740	-2.080530
C	1.314390	-2.202100	-3.168810
C	1.189500	2.789680	-2.836070
H	-2.424890	-4.646820	1.441150
H	-2.671180	4.401870	2.024690
H	-3.442770	-2.875850	3.227100
H	-3.581950	2.364350	3.568820
H	0.722100	-4.285180	-2.743940
H	0.491620	4.762670	-2.138140
H	2.002480	-2.250640	-4.003060
H	1.878590	2.984210	-3.647880
C	-0.902260	-3.355120	-0.594160
C	-1.095070	3.467540	-0.155500
C	1.302920	0.289060	-2.915130
C	-2.820650	-0.180670	2.532160
H	-0.927350	-4.434050	-0.706600
H	-1.174310	4.549160	-0.121010
H	2.001300	0.360870	-3.742460
H	-3.415030	-0.255440	3.436880
O	0.600750	0.072070	0.867820
S	-2.655000	0.057530	-1.768110
C	-4.219680	0.026400	-0.821820
H	-4.291690	-0.870980	-0.202310
H	-5.033450	0.016090	-1.554280
H	-4.322050	0.912680	-0.191080
C	1.335590	-1.055730	1.326600
H	0.683700	-1.708220	1.925580
H	1.699190	-1.646720	0.476310
C	2.456950	-0.538830	2.168530
H	2.206200	-0.262590	3.190880
C	3.774470	-0.260250	1.726060
C	4.727820	0.268160	2.647150
C	4.218730	-0.494840	0.390870
C	6.033600	0.533300	2.262400
H	4.412680	0.459830	3.669980
C	5.527480	-0.222710	0.016160
H	3.522310	-0.880330	-0.346840
C	6.446070	0.289530	0.943870
H	6.738550	0.933630	2.986210
H	5.840520	-0.406840	-1.008270
H	7.468360	0.499230	0.642610

TS-rotation[q]

Fe	-0.818545	0.021998	0.235611
N	-1.559340	0.822289	-1.445827
N	-0.153907	1.832191	0.805652
N	-1.547776	-1.777683	-0.288990
N	-0.139202	-0.770631	1.966761
C	-2.325188	0.184767	-2.396194
C	0.452985	2.151777	1.996815
C	-1.381770	2.111778	-1.893368
C	-0.155159	2.982725	0.051720
C	-2.321253	-2.061288	-1.392144
C	0.456656	-0.093657	3.003080
C	-1.389861	-2.964293	0.387406
C	-0.164524	-2.093679	2.334070
C	-2.641160	1.098453	-3.464020
C	0.830885	3.543233	1.998097
C	-2.047637	2.287137	-3.158794
C	0.464575	4.054913	0.789693
C	-2.652954	-3.463263	-1.411384
C	0.814918	-1.016256	4.053071
C	-2.068128	-4.023974	-0.315755
C	0.439024	-2.256229	3.634602
H	-3.233260	0.843026	-4.333411
H	1.323752	4.043005	2.822063
H	-2.056731	3.210922	-3.722951
H	0.588822	5.063480	0.416902
H	-3.251232	-3.940492	-2.176856
H	1.299103	-0.732522	4.978680
H	-2.090980	-5.056619	0.007850
H	0.544964	-3.204115	4.146411
C	-2.700778	-1.151708	-2.368788
C	0.720610	1.268247	3.034166
C	-0.724661	-3.126461	1.595438
C	-0.705188	3.117036	-1.215386
H	-3.308126	-1.519552	-3.189174
H	1.206392	1.665948	3.919160
H	-0.678449	-4.126643	2.013394
H	-0.639168	4.089090	-1.692684
O	0.805221	-0.324685	-0.564006
S	-2.843161	0.388328	1.325608
C	-3.424134	2.078974	0.946909
H	-3.600608	2.210390	-0.123145
H	-4.371565	2.206769	1.480946
H	-2.715343	2.832778	1.296971
C	1.006833	-0.920919	-1.813556
H	0.461452	-0.361774	-2.597618
H	0.562785	-1.935022	-1.827754
C	2.442201	-1.036942	-2.243043
H	2.538088	-1.497901	-3.226627
C	3.649787	-0.668765	-1.607115
C	4.883220	-0.923806	-2.288315
C	3.742044	-0.052774	-0.319397
C	6.106770	-0.592044	-1.727870
H	4.847854	-1.390758	-3.270090
C	4.975693	0.273445	0.228254
H	2.827925	0.154425	0.221572
C	6.168328	0.010991	-0.461926
H	7.022892	-0.801484	-2.274755
H	5.013744	0.740747	1.209629
H	7.127431	0.270921	-0.022833

TS2[q]

Fe	-0.896700	0.015870	0.204660
N	-0.197160	1.506020	1.369820
N	-0.008930	-1.350200	1.394030
N	-1.800620	1.385470	-0.973660
N	-1.615590	-1.472190	-0.948120
C	-0.417020	2.855520	1.218380
C	0.005980	-2.713580	1.223570
C	0.575550	1.365520	2.499740
C	0.750780	-1.093090	2.510410
C	-1.802830	2.750540	-0.813220
C	-1.382770	-2.818950	-0.805310
C	-2.565610	1.126640	-2.086800
C	-2.393970	-1.332440	-2.073050
C	0.239880	3.582470	2.275290
C	0.786630	-3.332440	2.267010
C	0.855390	2.660100	3.067920
C	1.246760	-2.328590	3.064910
C	-2.582220	3.368110	-1.857450
C	-2.040090	-3.547420	-1.862240
C	-3.054310	2.362330	-2.646320
C	-2.668250	-2.626660	-2.646230
H	0.220760	4.659530	2.382410
H	0.951470	-4.398410	2.359120
H	1.445580	2.823390	3.960590
H	1.868440	-2.398640	3.948340
H	-2.737950	4.434780	-1.957050
H	-2.011860	-4.623730	-1.974890
H	-3.678640	2.431720	-3.527940
H	-3.262100	-2.790020	-3.536440
C	-1.160990	3.444690	0.204250
C	-0.628830	-3.407260	0.201900
C	-2.843730	-0.131020	-2.605120
C	1.023490	0.164090	3.033150
H	-1.251420	4.525990	0.209980
H	-0.529950	-4.487800	0.190630
H	-3.459340	-0.178890	-3.497540
H	1.634280	0.211880	3.928880
O	0.683200	0.058050	-0.898830
S	-2.895130	0.005420	1.579040
C	-3.353900	-1.734120	1.925910
H	-2.557150	-2.256650	2.461850
H	-4.249570	-1.720670	2.555870
H	-3.581780	-2.277530	1.005020
C	1.374070	1.220460	-1.311550
H	1.726890	1.816150	-0.460430
H	0.744540	1.865560	-1.941270
C	2.481860	0.613280	-2.098640
H	2.220140	0.269700	-3.097130
C	3.775280	0.284780	-1.621190
C	4.687400	-0.382250	-2.489680
C	4.229050	0.615100	-0.311460
C	5.976950	-0.685440	-2.079320
H	4.356770	-0.647530	-3.490490
C	5.518990	0.300700	0.091170
H	3.555580	1.106340	0.382810
C	6.401210	-0.346300	-0.786590
H	6.657070	-1.190230	-2.759470
H	5.846460	0.556570	1.094920
H	7.409830	-0.587480	-0.463840

[2 + Fe(III)-Porph][d] (product complex)

Fe	0.894420	-0.030930	0.306260
N	2.157270	-0.273150	-1.249610
N	0.530300	-2.005410	0.349690
N	1.103830	1.970990	0.109900
N	-0.532560	0.239260	1.696320
C	2.857320	0.696490	-1.929620
C	-0.366050	-2.679490	1.145720
C	2.584940	-1.474930	-1.763070
C	1.174280	-2.967550	-0.390810
C	1.949020	2.651760	-0.737670
C	-1.290200	-0.724420	2.322480
C	0.532920	2.928100	0.918070
C	-0.892320	1.434590	2.274940
C	3.732440	0.092210	-2.905540
C	-0.282360	-4.100010	0.903070
C	3.565780	-1.255160	-2.800230
C	0.677020	-4.279280	-0.046730
C	1.892870	4.068440	-0.470440
C	-2.150830	-0.121290	3.311150
C	1.017240	4.239640	0.558800
C	-1.900280	1.217280	3.285540
H	4.383450	0.642870	-3.572760
H	-0.887940	-4.846160	1.401830
H	4.050680	-2.041710	-3.364620
H	1.022290	-5.203870	-0.492020
H	2.459310	4.818760	-1.007470
H	-2.848320	-0.666440	3.934410
H	0.714100	5.160450	1.041130
H	-2.351100	2.000020	3.882570
C	2.760110	2.063870	-1.701110
C	-1.226010	-2.087420	2.062070
C	-0.392440	2.684270	1.926230
C	2.137070	-2.728330	-1.364440
H	3.378790	2.720010	-2.305430
H	-1.883170	-2.739300	2.629230
H	-0.766960	3.541740	2.476900
H	2.560850	-3.591840	-1.868010
O	-0.775470	0.009740	-1.232260
S	2.587090	-0.114610	1.764530
C	2.109640	-1.188610	3.169600
H	1.906320	-2.210200	2.839390
H	2.956060	-1.202910	3.864030
H	1.232090	-0.797780	3.690380
C	-0.832840	0.858250	-2.399760
H	-1.103300	0.331780	-3.312480
H	-0.023310	1.578200	-2.478400
C	-1.833380	1.006850	-1.326180
H	-1.689260	1.838830	-0.641090
C	-3.229870	0.499350	-1.433580
C	-4.283950	1.305860	-0.984750
C	-3.516500	-0.755020	-1.991810
C	-5.606430	0.874480	-1.106430
H	-4.068310	2.274790	-0.540790
C	-4.837310	-1.187980	-2.105700
H	-2.702980	-1.394770	-2.321280
C	-5.886180	-0.373390	-1.667480
H	-6.415180	1.510330	-0.757070
H	-5.048530	-2.163450	-2.535210
H	-6.914330	-0.712610	-1.758290

[2 + Fe(III)-Porph][q] (product complex)

Fe	1.226120	-0.033950	0.375830
N	1.850440	1.301530	-0.998890
N	1.771290	-1.519680	-0.869290
N	0.238210	1.452870	1.325340
N	0.162180	-1.369190	1.456700
C	1.778650	2.677470	-0.909030
C	1.619530	-2.874810	-0.656670
C	2.579750	1.043680	-2.141880
C	2.510210	-1.408430	-2.029320
C	0.386990	2.808580	1.111880
C	0.228490	-2.744070	1.363500
C	-0.563200	1.341150	2.443130
C	-0.632740	-1.111710	2.553770
C	2.475980	3.288420	-2.009400
C	2.277070	-3.622120	-1.696000
C	2.970810	2.275270	-2.775230
C	2.827370	-2.712850	-2.548720
C	-0.335760	3.555280	2.107230
C	-0.537480	-3.356140	2.417140
C	-0.927010	2.645300	2.931590
C	-1.073380	-2.343450	3.154530
H	2.558890	4.356280	-2.165870
H	2.296420	-4.702650	-1.755990
H	3.545560	2.339040	-3.690300
H	3.393960	-2.891840	-3.453450
H	-0.379630	4.635890	2.151920
H	-0.646600	-4.424140	2.555170
H	-1.555970	2.823910	3.794220
H	-1.713400	-2.406900	4.025180
C	1.110240	3.385690	0.078820
C	0.914770	-3.451930	0.388760
C	-0.971200	0.148870	3.023180
C	2.894540	-0.217130	-2.626480
H	1.139740	4.469210	0.028370
H	0.879570	-4.535410	0.434830
H	-1.603200	0.206340	3.903330
H	3.476700	-0.275420	-3.540320
O	-1.874830	-0.175460	-1.788150
S	3.165780	-0.026310	1.808870
C	4.629640	-0.061440	0.696100
H	4.655200	0.821720	0.051820
H	5.532880	-0.071030	1.313680
H	4.624700	-0.957430	0.069300
C	-2.089430	1.217220	-2.062230
H	-2.654060	1.425270	-2.970460
H	-1.233840	1.864000	-1.873320
C	-2.772940	0.559380	-0.930920
H	-2.369230	0.745770	0.063900
C	-4.217420	0.188080	-0.972110
C	-5.052650	0.545700	0.094520
C	-4.761490	-0.501750	-2.064500
C	-6.413740	0.235260	0.063270
H	-4.635320	1.069690	0.951510
C	-6.120400	-0.816030	-2.093120
H	-4.109980	-0.803360	-2.879320
C	-6.951650	-0.445700	-1.031460
H	-7.051100	0.519820	0.896140
H	-6.530740	-1.354890	-2.943020
H	-8.009620	-0.692230	-1.054860

Int2[d]

Fe	-0.927160	0.120630	0.025080
N	-0.512630	1.506470	-1.384430
N	-0.370280	1.443620	1.446370
N	-1.425250	-1.232390	-1.400640
N	-1.283320	-1.295810	1.430080
C	-0.650200	1.344980	-2.738650
C	-0.373470	1.221470	2.798870
C	-0.025850	2.775120	-1.196430
C	0.097610	2.720380	1.267690
C	-1.455750	-0.998750	-2.752880
C	-1.176430	-1.123220	2.787370
C	-1.870530	-2.520630	-1.223740
C	-1.744330	-2.576830	1.241170
C	-0.247430	2.552340	-3.427530
C	0.097890	2.397940	3.497660
C	0.142270	3.439100	-2.470380
C	0.392830	3.327340	2.547080
C	-1.922370	-2.176470	-3.450050
C	-1.569930	-2.333280	3.474350
C	-2.178780	-3.120500	-2.502000
C	-1.920730	-3.235180	2.515550
H	-0.264180	2.683070	-4.502470
H	0.188050	2.480470	4.573740
H	0.510870	4.449700	-2.595390
H	0.774350	4.332130	2.679980
H	-2.034350	-2.251900	-4.524600
H	-1.572250	-2.457590	4.550170
H	-2.545710	-4.130420	-2.636750
H	-2.271770	-4.251920	2.640960
C	-1.092810	0.190270	-3.378300
C	-0.752050	0.037860	3.426500
C	-2.016490	-3.156610	0.005410
C	0.266220	3.348080	0.037390
H	-1.159360	0.219440	-4.461930
H	-0.710090	0.017820	4.511560
H	-2.381630	-4.179190	0.000760
H	0.646770	4.365160	0.040850
O	0.982590	-0.419980	-0.078810
S	-3.137380	0.745370	0.157140
C	-3.260140	2.574990	0.189130
H	-2.837600	3.018550	-0.716740
H	-4.320790	2.840770	0.248040
H	-2.745340	2.993930	1.058270
C	1.333530	-1.715820	-0.137010
H	0.880030	-2.275810	-0.998680
H	0.928690	-2.338350	0.707680
C	2.737690	-2.116240	-0.180990
H	2.877920	-3.195750	-0.235190
C	3.899400	-1.325820	-0.157690
C	5.170850	-1.973740	-0.209470
C	3.845040	0.099160	-0.082700
C	6.335390	-1.229650	-0.187370
H	5.209580	-3.057600	-0.266410
C	5.023630	0.829570	-0.060450
H	2.870570	0.570400	-0.043630
C	6.260050	0.171720	-0.112670
H	7.302170	-1.720570	-0.226970
H	4.990250	1.912770	-0.002740
H	7.177020	0.753870	-0.095130

Int2^{MECP}

Fe	0.958688	0.150999	-0.040172
N	1.029034	-1.070447	1.573453
N	0.336814	1.671864	1.137362
N	1.483027	-1.403118	-1.234206
N	0.811162	1.344303	-1.664009
C	1.425850	-2.386964	1.593607
C	0.061700	2.957998	0.742528
C	0.725005	-0.737738	2.871398
C	0.120644	1.628566	2.493288
C	1.822772	-2.673054	-0.829621
C	0.478641	2.677504	-1.676023
C	1.635729	-1.378230	-2.598654
C	1.051115	0.993783	-2.970042
C	1.372359	-2.896646	2.942786
C	-0.335860	3.750167	1.882141
C	0.934538	-1.875623	3.733795
C	-0.302604	2.925232	2.966851
C	2.192580	-3.471871	-1.973587
C	0.515235	3.182939	-3.027632
C	2.073276	-2.670341	-3.070035
C	0.866751	2.138573	-3.829891
H	1.635700	-3.906776	3.230156
H	-0.604198	4.798188	1.839587
H	0.767359	-1.876255	4.803583
H	-0.536267	3.157624	3.998270
H	2.501088	-4.508650	-1.926589
H	0.294843	4.204915	-3.308556
H	2.265278	-2.914204	-4.107334
H	0.995773	2.127508	-4.904794
C	1.803208	-3.134971	0.481751
C	0.133819	3.435366	-0.562217
C	1.427641	-0.269832	-3.413525
C	0.291396	0.511439	3.304472
H	2.095543	-4.166740	0.651606
H	-0.110467	4.480399	-0.725700
H	1.588807	-0.396868	-4.479564
H	0.086955	0.628563	4.364279
O	-1.142966	-0.382399	-0.295331
S	3.425225	0.786121	0.217688
C	3.740119	1.038020	2.012963
H	3.550199	0.120720	2.579725
H	4.785713	1.326629	2.163737
H	3.101601	1.829196	2.418951
C	-1.557980	-1.649066	-0.288467
H	-1.205726	-2.245883	0.607716
H	-1.113925	-2.294549	-1.095161
C	-2.973175	-2.021627	-0.287036
H	-3.141563	-3.098575	-0.301368
C	-4.111317	-1.196082	-0.237004
C	-5.404239	-1.798318	-0.206323
C	-4.002280	0.226449	-0.217497
C	-6.539327	-1.010571	-0.157659
H	-5.483872	-2.881601	-0.221713
C	-5.151896	1.001815	-0.167818
H	-3.008655	0.657258	-0.240472
C	-6.411508	0.388929	-0.138013
H	-7.523749	-1.466389	-0.134079
H	-5.077268	2.084436	-0.152021
H	-7.305740	1.004588	-0.099050

Int2^{q}

Fe	0.959110	0.150820	-0.039790
N	1.029140	-1.070380	1.573690
N	0.337130	1.671720	1.137660
N	1.483430	-1.402940	-1.234250
N	0.811600	1.344110	-1.664090
C	1.425900	-2.386740	1.593660
C	0.061910	2.957680	0.742590
C	0.725130	-0.737650	2.871510
C	0.120880	1.628490	2.493480
C	1.822880	-2.672780	-0.829660
C	0.478860	2.677240	-1.675970
C	1.635920	-1.378100	-2.598650
C	1.051380	0.993710	-2.970090
C	1.372350	-2.896520	2.942790
C	-0.335770	3.749930	1.882080
C	0.934610	-1.875570	3.733870
C	-0.302560	2.925250	2.966890
C	2.192440	-3.471750	-1.973580
C	0.515240	3.182670	-3.027600
C	2.073210	-2.670310	-3.070050
C	0.866730	2.138540	-3.829950
H	1.635690	-3.906700	3.230050
H	-0.604150	4.798010	1.839410
H	0.767420	-1.876210	4.803650
H	-0.536270	3.157660	3.998290
H	2.500900	-4.508560	-1.926550
H	0.294730	4.204770	-3.308480
H	2.265150	-2.914230	-4.107370
H	0.995610	2.127450	-4.904910
C	1.803240	-3.134730	0.481770
C	0.134020	3.434880	-0.562190
C	1.427720	-0.269820	-3.413600
C	0.291520	0.511530	3.304590
H	2.095400	-4.166390	0.651550
H	-0.110420	4.479870	-0.725670
H	1.588680	-0.396940	-4.479570
H	0.087000	0.628590	4.364320
O	-1.140590	-0.382360	-0.294680
S	3.418110	0.784630	0.218210
C	3.738240	1.037530	2.012220
H	3.549950	0.120530	2.579980
H	4.784360	1.325960	2.159970
H	3.101270	1.829370	2.419280
C	-1.556760	-1.648520	-0.288260
H	-1.205310	-2.245810	0.607970
H	-1.113330	-2.294260	-1.095070
C	-2.972390	-2.020690	-0.286970
H	-3.140490	-3.097560	-0.301430
C	-4.110820	-1.195850	-0.236900
C	-5.403970	-1.798330	-0.206220
C	-4.002280	0.226650	-0.217410
C	-6.539120	-1.010590	-0.157550
H	-5.483740	-2.881550	-0.221580
C	-5.151820	1.001840	-0.167740
H	-3.008540	0.657480	-0.240390
C	-6.411420	0.388960	-0.137910
H	-7.523570	-1.466390	-0.133930
H	-5.077260	2.084520	-0.151950
H	-7.305670	1.004550	-0.098940

TS3^{d}

Fe	0.895670	0.181770	0.233150
N	1.845810	-0.780910	-1.266590
N	0.754200	-1.575580	1.220610
N	0.951380	1.921870	-0.798600
N	-0.127950	1.130590	1.699750
C	2.317140	-0.205430	-2.421260
C	0.184690	-1.760430	2.457040
C	2.194700	-2.109960	-1.331370
C	1.239260	-2.803660	0.834930
C	1.540390	2.112740	-2.021790
C	-0.573090	0.563050	2.865610
C	0.448140	3.140170	-0.421560
C	-0.487200	2.452380	1.755110
C	2.979230	-1.198130	-3.236440
C	0.308610	-3.143040	2.858780
C	2.901930	-2.379460	-2.561790
C	0.960790	-3.790380	1.852420
C	1.403110	3.494090	-2.431040
C	-1.232890	1.557970	3.683760
C	0.724850	4.131560	-1.437660
C	-1.180130	2.730470	2.993830
H	3.436570	-0.999390	-4.197730
H	-0.061040	-3.548050	3.792640
H	3.283000	-3.350090	-2.853860
H	1.237810	-4.835400	1.789970
H	1.782080	3.901730	-3.359980
H	-1.673440	1.364090	4.653870
H	0.430570	5.172100	-1.379840
H	-1.567400	3.700700	3.279080
C	2.176310	1.132600	-2.777310
C	-0.431190	-0.774310	3.222400
C	-0.224700	3.397110	0.768300
C	1.908070	-3.061840	-0.357400
H	2.599810	1.438030	-3.729560
H	-0.837260	-1.076490	4.183290
H	-0.566070	4.413070	0.942540
H	2.241020	-4.079710	-0.536610
O	-0.901540	-0.147670	-0.635970
S	2.846590	0.769730	1.270890
C	3.975370	-0.674080	1.331410
H	4.210060	-1.038890	0.327930
H	4.903630	-0.350750	1.813900
H	3.544920	-1.493760	1.913180
C	-1.220620	-1.323150	-1.139910
H	-1.454430	-2.149650	-0.323000
H	-0.447020	-1.853990	-1.722520
C	-2.558460	-1.683080	-1.557240
H	-2.618280	-2.668090	-2.018120
C	-3.785220	-1.002940	-1.323130
C	-4.995810	-1.648290	-1.691870
C	-3.833400	0.307850	-0.776930
C	-6.212090	-1.005300	-1.524130
H	-4.957470	-2.649050	-2.112950
C	-5.059680	0.937640	-0.608950
H	-2.903410	0.786140	-0.495890
C	-6.243180	0.286870	-0.980220
H	-7.135460	-1.498940	-1.809660
H	-5.100960	1.938290	-0.190270
H	-7.197200	0.788840	-0.846350

TS3^{q}

Fe	0.986040	0.145460	-0.013110
N	0.947910	-1.099940	1.582740
N	0.314460	1.654410	1.149360
N	1.536530	-1.401970	-1.200660
N	0.905520	1.353880	-1.631590
C	1.321290	-2.423660	1.604660
C	0.065480	2.946970	0.756360
C	0.590050	-0.778340	2.870200
C	0.038880	1.599980	2.494500
C	1.838750	-2.682190	-0.797530
C	0.578950	2.688360	-1.645010
C	1.755180	-1.362470	-2.556080
C	1.201020	1.015610	-2.929200
C	1.194520	-2.949200	2.942410
C	-0.373540	3.730910	1.885780
C	0.738330	-1.930500	3.725860
C	-0.392860	2.895400	2.962650
C	2.254840	-3.470590	-1.932190
C	0.675300	3.206780	-2.988690
C	2.200070	-2.653460	-3.022250
C	1.058350	2.169320	-3.785070
H	1.426510	-3.967460	3.228150
H	-0.632140	4.781380	1.842150
H	0.520410	-1.941460	4.786400
H	-0.669320	3.119270	3.985290
H	2.546150	-4.512250	-1.884090
H	0.470240	4.232320	-3.268670
H	2.439050	-2.886740	-4.052190
H	1.234180	2.167960	-4.853390
C	1.744150	-3.162580	0.504190
C	0.192950	3.437160	-0.539010
C	1.593090	-0.244480	-3.367890
C	0.158440	0.472470	3.299710
H	2.012180	-4.200800	0.673610
H	-0.038610	4.485200	-0.701360
H	1.802710	-0.361770	-4.426460
H	-0.091680	0.580480	4.350520
O	-1.136560	-0.358820	-0.393350
S	3.425680	0.745370	0.353100
C	3.653060	1.038170	2.155240
H	3.421080	0.138040	2.733630
H	4.694340	1.315260	2.350470
H	3.007830	1.848940	2.508100
C	-1.575600	-1.602090	-0.422480
H	-1.568420	-2.129000	0.618860
H	-0.959660	-2.335290	-0.987540
C	-2.981840	-1.974000	-0.506800
H	-3.146220	-3.048960	-0.573940
C	-4.127070	-1.151240	-0.380650
C	-5.412860	-1.760310	-0.355030
C	-4.026430	0.266880	-0.319390
C	-6.554410	-0.980370	-0.276990
H	-5.488440	-2.843040	-0.403170
C	-5.179930	1.035130	-0.236560
H	-3.037550	0.708610	-0.333420
C	-6.436770	0.417070	-0.216070
H	-7.535120	-1.444840	-0.260900
H	-5.108580	2.117240	-0.188640
H	-7.333760	1.026570	-0.152270

[3 + Fe(III)-Porph]^{d} (product complex)

Fe	-0.893930	-0.069950	0.368810
N	-1.805120	0.834540	-1.180670
N	-0.206480	1.717560	0.978230
N	-1.411150	-1.878780	-0.367250
N	0.186890	-0.998780	1.797180
C	-2.610520	0.230360	-2.118980
C	0.550120	1.956310	2.100220
C	-1.827730	2.179800	-1.473930
C	-0.428940	2.946820	0.402990
C	-2.275630	-2.112910	-1.411700
C	0.882100	-0.385880	2.811080
C	-1.083620	-3.116620	0.135800
C	0.312230	-2.349380	2.015470
C	-3.149510	1.217700	-3.023400
C	0.804690	3.371290	2.238220
C	-2.657870	2.425510	-2.628020
C	0.203330	3.985110	1.181450
C	-2.486780	-3.531760	-1.576450
C	1.461160	-1.376190	3.689630
C	-1.742320	-4.154320	-0.620640
C	1.112460	-2.594740	3.192440
H	-3.809950	0.996490	-3.852450
H	1.380050	3.816950	3.039870
H	-2.834220	3.401350	-3.062700
H	0.178910	5.039460	0.936560
H	-3.121510	-3.975320	-2.333280
H	2.059660	-1.148780	4.562830
H	-1.641500	-5.214860	-0.427320
H	1.361680	-3.577150	3.573270
C	-2.844270	-1.136800	-2.222010
C	1.043990	0.985770	2.964420
C	-0.265940	-3.344210	1.236170
C	-1.172570	3.168620	-0.749930
H	-3.504370	-1.470640	-3.016710
H	1.628980	1.326640	3.813140
H	-0.090500	-4.375450	1.526600
H	-1.273040	4.193370	-1.094090
O	0.952130	-0.169410	-0.847930
S	-2.697400	-0.041110	1.692210
C	-3.494110	1.604670	1.575540
H	-3.836110	1.810650	0.558190
H	-4.360850	1.591100	2.244430
H	-2.814680	2.399230	1.893640
C	1.024510	-0.509360	-2.020190
H	2.134440	0.074600	-3.699070
H	0.112070	-0.805440	-2.562430
C	2.304560	-0.557030	-2.813870
H	2.377870	-1.581140	-3.209990
C	3.555090	-0.153390	-2.069660
C	4.322630	-1.111020	-1.393770
C	3.962490	1.186820	-2.034980
C	5.472150	-0.737330	-0.694470
H	4.021410	-2.155760	-1.418130
C	5.111540	1.563450	-1.336750
H	3.379590	1.939330	-2.561080
C	5.869590	0.601610	-0.664260
H	6.058130	-1.492410	-0.177510
H	5.415790	2.606490	-1.321670
H	6.765710	0.893030	-0.123300

[3 + Fe(III)-Porph]^{q} (product complex)

Fe	1.166030	0.029840	-0.251240
N	1.332920	1.191290	1.387700
N	0.312800	1.570320	-1.227660
N	1.605350	-1.587210	0.879350
N	0.597960	-1.206120	-1.741730
C	1.852670	0.824680	2.611460
C	-0.153880	1.568590	-2.524610
C	1.081090	2.543530	1.479300
C	0.193050	2.871590	-0.789810
C	2.093910	-1.582740	2.169430
C	0.097170	-0.836380	-2.971410
C	1.644370	-2.902680	0.470480
C	0.767570	-2.571640	-1.803100
C	1.932050	1.967990	3.482930
C	-0.569570	2.892890	-2.908100
C	1.449830	3.033190	2.782270
C	-0.358020	3.700430	-1.830980
C	2.439030	-2.919820	2.577240
C	-0.048380	-1.991210	-3.819600
C	2.157420	-3.738780	1.524640
C	0.364980	-3.067990	-3.094070
H	2.303190	1.940550	4.499460
H	-0.977620	3.151540	-3.876760
H	1.344950	4.061450	3.104000
H	-0.554260	4.760450	-1.732550
H	2.839440	-3.181390	3.548310
H	-0.426570	-1.964480	-4.833500
H	2.280360	-4.811870	1.452310
H	0.398260	-4.108960	-3.389150
C	2.221560	-0.462090	2.977810
C	-0.244630	0.454680	-3.346960
C	1.255460	-3.369290	-0.777530
C	0.548200	3.333040	0.469820
H	2.620360	-0.605720	3.976840
H	-0.631870	0.600860	-4.350030
H	1.341200	-4.434600	-0.965660
H	0.400410	4.387340	0.680110
O	-1.512920	-0.401030	0.658070
S	3.429670	0.333850	-1.083100
C	3.796240	2.131440	-0.956830
H	3.733740	2.473600	0.080070
H	4.812940	2.306190	-1.322190
H	3.101000	2.717920	-1.564100
C	-1.790320	-1.064140	1.638580
H	-3.190110	-0.950830	3.207410
H	-0.986100	-1.548770	2.226900
C	-3.190720	-1.305340	2.165510
H	-3.310470	-2.396490	2.236710
C	-4.302190	-0.673610	1.362870
C	-4.940010	-1.388170	0.340120
C	-4.706440	0.644130	1.616620
C	-5.957360	-0.799760	-0.414280
H	-4.640610	-2.413460	0.134880
C	-5.723040	1.235800	0.864400
H	-4.224320	1.209490	2.410890
C	-6.351840	0.514830	-0.153980
H	-6.443260	-1.369490	-1.201870
H	-6.025860	2.257770	1.076250
H	-7.145430	0.972990	-0.737940

Table S12: Cartesian coordinates (xyz, in Å) of all DFT optimized stationary points reported in **Figures S9**, **S26**, and **S27** and **Table S4**.

Int1-wat^{q}

Fe	0.894581	0.093474	-0.334507
N	1.364184	1.709752	0.758225
N	2.172986	-1.010108	0.745098
N	-0.329335	1.216760	-1.474326
N	0.452889	-1.519593	-1.459750
C	0.959362	3.006341	0.531564
C	2.517393	-2.324391	0.527858
C	2.148124	1.741611	1.888569
C	2.839864	-0.617814	1.884761
C	-0.500581	2.580405	-1.402925
C	1.030943	-2.762863	-1.380263
C	-1.155581	0.774037	-2.478468
C	-0.484904	-1.592880	-2.460797
C	1.504824	3.873552	1.544169
C	3.431044	-2.767115	1.549661
C	2.229369	3.089200	2.391656
C	3.618290	-1.716167	2.398983
C	-1.460742	3.005999	-2.389931
C	0.437281	-3.645958	-2.354764
C	-1.874984	1.886239	-3.047866
C	-0.510110	-2.924942	-3.015320
H	1.336727	4.941792	1.591449
H	3.852470	-3.762527	1.605282
H	2.784217	3.381539	3.273895
H	4.233112	-1.668557	3.288590
H	-1.773269	4.031561	-2.538836
H	0.716199	-4.682692	-2.492313
H	-2.593058	1.803276	-3.853576
H	-1.167520	-3.243553	-3.814030
C	0.110918	3.423841	-0.485146
C	2.012903	-3.139989	-0.476055
C	-1.256626	-0.537297	-2.925377
C	2.812527	0.653907	2.440412
H	-0.122491	4.481936	-0.541786
H	2.376715	-4.160946	-0.523946
H	-1.955897	-0.741173	-3.729433
H	3.393489	0.822622	3.341088
O	-0.531373	-0.351138	0.806513
S	2.577075	0.622803	-1.881606
C	4.129878	0.937742	-0.969872
H	4.016706	1.767007	-0.267261
H	4.883307	1.208740	-1.716894
H	4.466763	0.047451	-0.433782
C	-1.396771	0.585748	1.445642
H	-0.844658	1.119110	2.235111
H	-1.744139	1.335763	0.727478
C	-2.524464	-0.190341	2.041262
H	-2.256369	-0.840719	2.870766
C	-3.857415	-0.254226	1.566532
C	-4.799183	-1.082751	2.248695
C	-4.333377	0.482169	0.440816
C	-6.121811	-1.160841	1.840250
H	-4.459844	-1.657268	3.106932
C	-5.659117	0.393538	0.039065
H	-3.650724	1.114111	-0.117929
C	-6.564555	-0.422924	0.732193
H	-6.816429	-1.798743	2.380221
H	-5.996161	0.962224	-0.823627
H	-7.600324	-0.485827	0.411272
O	-0.140915	-2.497023	2.598456
H	0.750592	-2.321060	2.935359
H	-0.263462	-1.804584	1.912529

Int2-wat^{d}

Fe	0.993823	0.008747	0.229941
N	0.868403	-2.007552	0.205176
N	0.007323	0.023659	1.987724
N	1.891829	-0.006926	-1.584184
N	1.057575	2.029526	0.210090
C	1.412692	-2.846801	-0.740853
C	-0.312277	1.135564	2.725274
C	0.288846	-2.822570	1.149036
C	-0.475945	-1.052430	2.688178
C	2.281612	-1.120695	-2.287135
C	0.594427	2.860260	1.198500
C	2.310615	1.074309	-2.320262
C	1.582979	2.842473	-0.761542
C	1.165409	-4.224625	-0.382962
C	-1.013112	0.748897	3.929814
C	0.464548	-4.209242	0.785615
C	-1.117598	-0.608704	3.905292
C	2.950455	-0.730057	-3.508121
C	0.827482	4.241137	0.836722
C	2.965636	0.631394	-3.529798
C	1.441043	4.229978	-0.381127
H	1.489099	-5.079199	-0.963929
H	-1.373431	1.440393	4.681252
H	0.097022	-5.048667	1.362580
H	-1.579455	-1.264217	4.632985
H	3.350230	-1.421464	-4.239388
H	0.551711	5.093286	1.445257
H	3.381259	1.290433	-4.281721
H	1.772627	5.070509	-0.977747
C	2.069718	-2.440188	-1.898136
C	-0.036795	2.451988	2.369509
C	2.163359	2.405264	-1.948164
C	-0.356814	-2.383340	2.299424
H	2.439543	-3.218559	-2.558774
H	-0.355388	3.228914	3.058058
H	2.543596	3.161228	-2.628367
H	-0.777824	-3.137679	2.957270
O	-0.842580	0.146430	-0.690500
S	3.028528	0.024879	1.259386
C	3.101629	-1.334655	2.487305
H	3.021293	-2.313453	2.006909
H	4.069112	-1.270641	2.996105
H	2.306794	-1.239324	3.231901
C	-1.190135	-0.739866	-1.644701
H	-0.902815	-1.799950	-1.383910
H	-0.628540	-0.629351	-2.604139
C	-2.585048	-0.961457	-2.018700
H	-2.696647	-1.640052	-2.864632
C	-3.772249	-0.497434	-1.426765
C	-5.016499	-0.974256	-1.940802
C	-3.775809	0.408245	-0.323306
C	-6.209367	-0.571840	-1.370786
H	-5.010951	-1.662102	-2.781199
C	-4.981700	0.798409	0.238889
H	-2.829132	0.780599	0.042693
C	-6.189932	0.313454	-0.279705
H	-7.154773	-0.934975	-1.760078
H	-4.991422	1.486207	1.078193
H	-7.128708	0.630181	0.165764
O	-1.639015	2.689854	-1.478199
H	-1.277411	1.820375	-1.187249
H	-1.057791	3.331520	-1.042676

Int2-wat[q]

S	-2.609997	0.001226	-2.196524
C	-2.902608	-1.754142	-2.658856
H	-3.627397	-1.797008	-3.478566
H	-3.303851	-2.322095	-1.813571
N	0.208990	-1.420080	-1.139627
N	0.175458	1.446914	-1.256456
N	-1.986881	1.498260	0.592770
C	1.061533	-3.476702	-1.751320
N	-1.937809	-1.367627	0.737071
C	1.779177	-2.528059	-2.417977
C	0.083930	-2.776739	-0.953806
C	-0.834812	-3.393841	-0.111026
C	1.241421	-1.247018	-2.029198
C	1.714704	-0.022382	-2.490563
C	1.212622	1.222501	-2.131157
C	1.708917	2.478393	-2.641371
C	0.955964	3.461129	-2.071678
C	-0.001055	2.808072	-1.210610
C	-0.963290	3.471093	-0.454655
C	-1.885207	2.851064	0.384518
C	-2.867386	3.550319	1.178037
C	-3.561297	2.603758	1.870067
C	-3.007628	1.324043	1.497901
C	-3.457781	0.104182	1.988329
C	-2.959545	-1.143313	1.627126
C	-3.453939	-2.398543	2.139310
C	-1.775851	-2.730709	0.672307
C	-2.720430	-3.383230	1.546940
H	-0.817946	-4.478090	-0.061090
H	2.538416	-0.041770	-3.197282
H	-0.998865	4.553670	-0.525896
H	-4.273394	0.126311	2.704224
Fe	-0.918289	0.038722	-0.303679
C	1.001187	-0.912753	2.071941
C	2.426121	-1.252610	2.181228
C	3.555404	-0.615137	1.641962
C	4.840540	-1.196653	1.870359
C	3.459951	0.582015	0.868991
C	5.975952	-0.614300	1.340483
C	4.611295	1.156225	0.351012
C	5.859391	0.562274	0.579976
H	0.701560	-0.874880	3.158420
H	0.536234	-1.893660	1.793257
H	2.611047	-2.139600	2.788209
H	4.910706	-2.105158	2.461506
H	2.486231	1.030996	0.728985
H	6.951699	-1.057385	1.510344
H	4.544880	2.068341	-0.233232
H	6.753925	1.020264	0.167422
O	0.561299	0.130263	1.350778
O	1.003044	2.672116	2.248462
H	0.814482	1.738986	1.981695
H	0.575428	3.181597	1.543532
H	-1.977567	-2.236497	-2.989842
H	-4.258879	-2.496391	2.856695
H	-2.799534	-4.454978	1.678505
H	1.171766	-4.553267	-1.785352
H	2.599002	-2.666901	-3.111371
H	2.523246	2.576860	-3.348044
H	1.026555	4.532304	-2.212164
H	-2.993230	4.625473	1.194032
H	-4.375760	2.741552	2.569782

Int1-EF[q]

Fe	37.815127	32.293075	49.178314
N	39.030701	32.679041	50.729149
N	39.403914	32.233763	47.946541
N	36.226527	32.286398	50.416643
N	36.596965	31.845482	47.629697
C	38.672868	32.771737	52.054931
C	39.397009	31.934817	46.605459
C	40.382216	32.929517	50.674573
C	40.702862	32.555455	48.264270
C	36.250716	32.424840	51.786150
C	36.975510	31.588342	46.334733
C	34.906420	32.130529	50.067176
C	35.226777	31.756212	47.656359
C	39.830766	33.081804	52.854572
C	40.729616	32.056579	46.067485
C	40.886620	33.189984	51.999250
C	41.536291	32.450594	47.092245
C	34.909869	32.357508	52.309902
C	35.811043	31.326071	45.523464
C	34.076909	32.184983	51.245080
C	34.727420	31.438609	46.340126
H	39.816909	33.206955	53.929686
H	40.991815	31.872088	45.033592
H	41.920223	33.416858	52.227348
H	42.599824	32.651668	47.076695
H	34.656964	32.440476	53.359160
H	35.840150	31.096069	44.466100
H	32.998420	32.091848	51.239160
H	33.680178	31.315630	46.095370
C	37.385426	32.635916	52.557188
C	38.276395	31.610769	45.852117
C	34.429156	31.906403	48.782006
C	41.164610	32.898388	49.527378
H	37.252703	32.733318	53.629667
H	38.425216	31.395149	44.799138
H	33.356503	31.802309	48.656176
H	42.221050	33.121979	49.633380
O	37.607067	34.093340	48.820253
S	37.988854	29.989629	49.590443
C	39.750240	29.565660	49.839991
H	40.169836	30.098061	50.697141
H	39.792700	28.489468	50.037167
H	40.343459	29.786047	48.949283
C	37.368794	35.121605	49.773208
H	38.220349	35.194068	50.465476
H	36.478713	34.889912	50.371895
C	37.215626	36.400269	49.015395
H	38.135584	36.877663	48.683469
C	35.994034	36.980262	48.590831
C	36.015879	38.197637	47.846403
C	34.715976	36.416991	48.881645
C	34.843556	38.812097	47.432438
H	36.977814	38.645075	47.608327
C	33.549100	37.038979	48.458906
H	34.653026	35.483204	49.431031
C	33.599266	38.239345	47.735393
H	34.890854	39.740299	46.869138
H	32.587709	36.587752	48.690143
H	32.681511	38.720030	47.409086

Int2-EF^{d}

Fe	37.838692	32.153782	49.152729
N	39.414766	32.349049	47.892329
N	36.600805	31.943414	47.571878
N	39.068951	32.453198	50.734455
N	36.255685	32.059857	50.409326
C	40.733424	32.514858	48.243092
C	35.241883	31.748463	47.612056
C	39.381496	32.305498	46.520085
C	36.950682	31.958895	46.243094
C	40.437284	32.588806	50.696216
C	34.943888	31.846878	50.061937
C	38.713127	32.492472	52.060364
C	36.280494	32.166365	51.778292
C	41.554286	32.578875	47.057292
C	34.720525	31.629376	46.271442
C	40.716017	32.451798	45.989473
C	35.779173	31.762515	45.422889
C	40.956039	32.720612	52.036248
C	34.117972	31.816748	51.245881
C	39.887311	32.663763	52.881224
C	34.945976	32.017402	52.309403
H	42.629585	32.705541	47.059073
H	33.677592	31.468930	46.028778
H	40.962934	32.451624	44.935199
H	35.783922	31.731825	44.340563
H	42.003759	32.840830	52.281548
H	33.046297	31.662557	51.244793
H	39.878657	32.726844	53.962104
H	34.694292	32.060227	53.361618
C	41.216132	32.619343	49.544385
C	34.465647	31.695279	48.765429
C	37.417227	32.370459	52.552789
C	38.237958	32.132888	45.746289
H	42.287426	32.741388	49.670414
H	33.399303	31.533938	48.642010
H	37.285419	32.423547	53.628963
H	38.362466	32.118454	44.667971
O	37.519074	34.307310	48.999064
S	38.223581	29.635837	49.374751
C	38.433766	28.962428	47.675252
H	39.309482	29.398730	47.183554
H	38.572087	27.877238	47.725846
H	37.555065	29.169828	47.056155
C	38.501945	35.185493	49.198000
H	39.430167	34.989616	48.581201
H	38.974656	35.137736	50.218430
C	38.341514	36.623230	48.974721
H	39.237710	37.202222	49.198253
C	37.222855	37.332937	48.501926
C	37.315307	38.748158	48.344127
C	36.000012	36.672564	48.178587
C	36.230612	39.470912	47.883273
H	38.247468	39.248255	48.590912
C	34.921799	37.413316	47.716540
H	35.956700	35.597546	48.305713
C	35.034854	38.802133	47.569748
H	36.298688	40.547155	47.762859
H	33.988610	36.917972	47.467919
H	34.184810	39.373294	47.207340

Int2-EF^{q}

Fe	37.825787	32.230751	49.223926
N	36.544800	32.077060	47.670084
N	36.261835	32.050872	50.489698
N	39.390755	32.476180	47.959374
N	39.107845	32.448991	50.778821
C	36.883174	32.106954	46.342420
C	36.329364	32.058466	51.858638
C	35.181490	31.933752	47.716149
C	34.934743	31.912503	50.171292
C	39.328004	32.431738	46.589198
C	38.774119	32.380964	52.108338
C	40.717462	32.642616	48.277671
C	40.471057	32.618295	50.733672
C	35.697890	31.969404	45.523313
C	35.004903	31.911511	52.423302
C	34.641386	31.864653	46.376111
C	34.139127	31.823458	51.375889
C	40.650979	32.585990	46.026149
C	39.958551	32.522572	52.925732
C	41.512448	32.718298	47.073136
C	41.010719	32.671143	52.073336
H	35.695165	31.960058	44.440382
H	34.786842	31.885447	53.483800
H	33.590720	31.750267	46.139778
H	33.062493	31.708704	51.396839
H	40.872470	32.588833	44.966051
H	39.964706	32.507237	54.008577
H	42.586976	32.850863	47.051095
H	42.059276	32.801175	52.310761
C	38.170038	32.264964	45.835616
C	37.489638	32.204997	52.614041
C	41.228471	32.714785	49.570399
C	34.421103	31.862157	48.879205
H	38.280011	32.259609	44.755125
H	37.382765	32.181326	53.694563
H	42.300969	32.844773	49.679302
H	33.346655	31.748266	48.770215
O	37.464882	34.184959	49.199051
S	38.244468	29.969411	49.243662
C	36.669302	29.049770	49.052698
H	36.186749	29.279252	48.098498
H	36.898240	27.979353	49.081854
H	35.972386	29.281462	49.862923
C	38.461731	35.075583	49.335439
H	39.305275	34.932975	48.603864
H	39.041663	34.973876	50.291071
C	38.211777	36.511527	49.236653
H	39.105630	37.117313	49.385025
C	37.016280	37.200923	48.971194
C	37.045440	38.627427	48.915044
C	35.780572	36.519223	48.754561
C	35.890107	39.339690	48.653701
H	37.986912	39.143202	49.080422
C	34.631492	37.249883	48.492357
H	35.781186	35.437212	48.800762
C	34.684207	38.649532	48.442368
H	35.910504	40.423655	48.610846
H	33.688842	36.738546	48.325698
H	33.777992	39.212218	48.236759

Int1^{q} (gas phase)

Fe	37.828897	32.282386	49.162436
N	39.025379	32.688085	50.726940
N	39.435531	32.197778	47.955463
N	36.220645	32.311320	50.379506
N	36.627824	31.836880	47.600426
C	38.649747	32.793509	52.045400
C	39.443253	31.884988	46.620022
C	40.377297	32.929953	50.687774
C	40.728613	32.522369	48.285519
C	36.228124	32.457740	51.746351
C	37.021858	31.563487	46.316837
C	34.906681	32.167091	50.011604
C	35.258910	31.772508	47.607334
C	39.798146	33.105156	52.858284
C	40.783290	31.995302	46.096607
C	40.865672	33.200063	52.016842
C	41.577554	32.400192	47.125502
C	34.879249	32.407680	52.252216
C	35.865697	31.313852	45.487798
C	34.060266	32.237231	51.177214
C	34.772982	31.452527	46.285282
H	39.771681	33.239920	53.932154
H	41.058444	31.798077	45.068306
H	41.897143	33.423477	52.258328
H	42.641732	32.599321	47.119460
H	34.611376	32.499489	53.297109
H	35.906923	31.074896	44.432754
H	32.981029	32.153544	51.157775
H	33.727651	31.346398	46.024542
C	37.354514	32.668029	52.529720
C	38.329951	31.562610	45.855562
C	34.447763	31.941976	48.719830
C	41.173487	32.880573	49.550229
H	37.207814	32.773872	53.599938
H	38.492024	31.334036	44.806987
H	33.375535	31.851713	48.577875
H	42.229622	33.102000	49.667381
O	37.634818	34.061470	48.771262
S	37.958899	30.019043	49.611166
C	39.711643	29.566574	49.853656
H	40.150037	30.109699	50.695061
H	39.733287	28.494577	50.075579
H	40.301549	29.754616	48.953023
C	37.440807	35.127444	49.685029
H	38.333495	35.238094	50.320183
H	36.594325	34.914322	50.350883
C	37.223408	36.374230	48.889457
H	38.110363	36.810940	48.434469
C	35.974567	36.964389	48.574957
C	35.936884	38.151585	47.784870
C	34.724872	36.443470	49.022811
C	34.739579	38.777922	47.476075
H	36.875871	38.566198	47.425752
C	33.532057	37.077367	48.705226
H	34.704333	35.529854	49.607925
C	33.525062	38.248198	47.934731
H	34.743923	39.682928	46.873825
H	32.592937	36.657036	49.056588
H	32.586616	38.738571	47.691505

Int2^{d} (gas phase)

Fe	37.833797	32.252468	49.188805
N	36.572247	32.065792	47.621178
N	36.252861	32.114853	50.439915
N	39.414494	32.434662	47.937126
N	39.094940	32.484859	50.755466
C	36.926453	32.057388	46.298313
C	36.302090	32.153534	51.808010
C	35.207069	31.953527	47.654293
C	34.929260	31.996174	50.106125
C	39.370422	32.350117	46.569080
C	38.745644	32.447235	52.081070
C	40.739964	32.590080	48.269897
C	40.462032	32.633274	50.722534
C	35.748676	31.923779	45.468334
C	34.967983	32.043823	52.357544
C	34.681259	31.862114	46.309814
C	34.115658	31.948994	51.301235
C	40.702850	32.468464	46.021725
C	39.922333	32.589359	52.908234
C	41.551483	32.619854	47.075684
C	40.985870	32.707314	52.066280
H	35.758053	31.887415	44.386032
H	34.734871	32.044849	53.415108
H	33.631416	31.763279	46.062889
H	33.036808	31.854612	51.310299
H	40.939869	32.435322	44.965527
H	39.916943	32.593552	53.991184
H	42.628380	32.733105	47.064152
H	42.033371	32.824764	52.314526
C	38.221214	32.180119	45.804676
C	37.453907	32.298828	52.574368
C	41.233891	32.689587	49.566635
C	34.432220	31.925810	48.809013
H	38.345661	32.142113	44.726341
H	37.333740	32.298565	53.653851
H	42.307224	32.803421	49.686256
H	33.357010	31.829540	48.688877
O	37.496223	34.176361	49.115662
S	38.258678	30.009844	49.276875
C	36.690250	29.090193	49.115410
H	36.203686	29.315037	48.161621
H	36.914093	28.020077	49.159927
H	36.003506	29.346584	49.927605
C	38.505878	35.058956	49.218273
H	39.312454	34.894428	48.452942
H	39.112400	34.925133	50.154976
C	38.234249	36.498798	49.159466
H	39.127003	37.118023	49.253590
C	37.006279	37.171417	49.001282
C	36.994091	38.598843	48.973606
C	35.767361	36.474052	48.866863
C	35.808403	39.292995	48.820061
H	37.934380	39.134991	49.075451
C	34.586742	37.188778	48.713482
H	35.788353	35.391010	48.889076
C	34.599815	38.588257	48.689404
H	35.809189	40.378906	48.800236
H	33.646244	36.655197	48.611462
H	33.669117	39.136200	48.568783

Int1-EF^{q} (gas phase)

Fe	37.828903	32.282375	49.163044
N	39.025757	32.689228	50.726971
N	39.435268	32.196735	47.955788
N	36.220924	32.312355	50.380444
N	36.627464	31.835718	47.601646
C	38.650421	32.795681	52.045433
C	39.442680	31.882896	46.620590
C	40.377678	32.930996	50.687317
C	40.728439	32.521530	48.285301
C	36.228710	32.459839	51.747173
C	37.021206	31.561287	46.318188
C	34.906868	32.167953	50.012936
C	35.258549	31.771458	47.608902
C	39.799019	33.107891	52.857822
C	40.782608	31.992741	46.096790
C	40.866360	33.202103	52.016071
C	41.577118	32.398407	47.125195
C	34.879940	32.410290	52.253368
C	35.864845	31.311085	45.489597
C	34.060711	32.239075	51.178673
C	34.772312	31.450472	46.287209
H	39.772800	33.243488	53.931595
H	41.057526	31.794701	45.068584
H	41.897896	33.425659	52.257153
H	42.641300	32.597488	47.118755
H	34.612301	32.502937	53.298244
H	35.905823	31.071299	44.434731
H	32.981461	32.155472	51.159531
H	33.726917	31.344225	46.026775
C	37.355289	32.670650	52.530132
C	38.329197	31.559971	45.856628
C	34.447655	31.941868	48.721436
C	41.173611	32.880699	49.549633
H	37.208828	32.777332	53.600304
H	38.491028	31.330566	44.808197
H	33.375389	31.851595	48.579781
H	42.229782	33.102173	49.666374
O	37.634850	34.061169	48.770531
S	37.958889	30.019348	49.613503
C	39.711670	29.566996	49.855961
H	40.150276	30.110767	50.696838
H	39.733322	28.495173	50.078720
H	40.301384	29.754307	48.955048
C	37.441051	35.127857	49.683510
H	38.333923	35.239040	50.318314
H	36.594776	34.915237	50.349777
C	37.223392	36.373978	48.886971
H	38.110165	36.810062	48.431026
C	35.974495	36.964119	48.572678
C	35.936615	38.150551	47.781448
C	34.724949	36.443954	49.021816
C	34.739277	38.776878	47.472775
H	36.875481	38.564575	47.421341
C	33.532093	37.077831	48.704342
H	34.704536	35.530926	49.607853
C	33.524910	38.247906	47.932697
H	34.743469	39.681290	46.869629
H	32.593086	36.658081	49.056698
H	32.586433	38.738270	47.689564

Int2-EF^{d} (gas phase)

Fe	37.833820	32.252491	49.188766
N	36.572292	32.065933	47.621098
N	36.252857	32.114780	50.439835
N	39.414543	32.434734	47.937126
N	39.094948	32.484692	50.755459
C	36.926521	32.057659	46.298241
C	36.302063	32.153381	51.807930
C	35.207111	31.953686	47.654182
C	34.929260	31.996153	50.106014
C	39.370491	32.350334	46.569069
C	38.745621	32.446999	52.081055
C	40.740017	32.590084	48.269928
C	40.462044	32.633072	50.722565
C	35.748756	31.924143	45.468227
C	34.967945	32.043671	52.357433
C	34.681320	31.862406	46.309689
C	34.115635	31.948923	51.301105
C	40.702930	32.468704	46.021744
C	39.922298	32.589043	52.908249
C	41.551552	32.619961	47.075729
C	40.985859	32.707008	52.066322
H	35.758148	31.887894	44.385921
H	34.734814	32.044651	53.414993
H	33.631481	31.763613	46.062737
H	33.036785	31.854576	51.310143
H	40.939960	32.435677	44.965542
H	39.916885	32.593178	53.991199
H	42.628452	32.733189	47.064220
H	42.033356	32.824429	52.314594
C	38.221287	32.180435	45.804630
C	37.453873	32.298595	52.574318
C	41.233925	32.689457	49.566681
C	34.432243	31.925884	48.808887
H	38.345749	32.142544	44.726295
H	37.333683	32.298275	53.653797
H	42.307259	32.803261	49.686329
H	33.357033	31.829651	48.688721
O	37.496265	34.176350	49.115791
S	38.258690	30.009798	49.276600
C	36.690243	29.090168	49.115208
H	36.203579	29.315125	48.161499
H	36.914078	28.020044	49.159580
H	36.003590	29.346485	49.927502
C	38.505909	35.058971	49.218445
H	39.312366	34.894627	48.452934
H	39.112553	34.925022	50.155025
C	38.234200	36.498814	49.159729
H	39.126919	37.118073	49.253948
C	37.006222	37.171391	49.001476
C	36.993992	38.598820	48.973858
C	35.767326	36.473995	48.866970
C	35.808292	39.292942	48.820278
H	37.934258	39.134991	49.075775
C	34.586700	37.188690	48.713547
H	35.788349	35.390949	48.889141
C	34.599731	38.588173	48.689529
H	35.809048	40.378857	48.800503
H	33.646221	36.655087	48.611462
H	33.669025	39.136093	48.568882

Int2-EF[q] (gas phase)

Fe	38.049469	32.396069	48.953751
N	39.704124	32.417152	47.784206
N	36.902794	32.050266	47.325008
N	39.229431	32.488159	50.600986
N	36.428101	32.124866	50.134754
C	41.005718	32.568298	48.196655
C	35.538925	31.894007	47.293575
C	39.740273	32.342392	46.414082
C	37.322369	32.038052	46.016994
C	40.596119	32.628334	50.631535
C	35.128582	31.957254	49.725910
C	38.812977	32.486984	51.909206
C	36.395592	32.186275	51.506760
C	41.885742	32.593552	47.053654
C	35.087051	31.772734	45.928986
C	41.101265	32.453239	45.948818
C	36.192627	31.861454	45.137772
C	41.052120	32.719608	51.997005
C	34.252811	31.903551	50.871731
C	39.946869	32.632141	52.788773
C	35.038250	32.045559	51.975315
H	42.961971	32.699409	47.107117
H	34.054729	31.637152	45.631638
H	41.401226	32.421467	44.908970
H	36.254318	31.814142	44.057865
H	42.087231	32.830425	52.294678
H	33.179031	31.773663	50.820782
H	39.888626	32.657368	53.869610
H	34.742340	32.055908	53.016727
C	41.427299	32.669437	49.517948
C	34.706074	31.851402	48.405708
C	37.497051	32.355167	52.336582
C	38.636993	32.171993	45.586460
H	42.492714	32.780903	49.694405
H	33.642673	31.721128	48.229607
H	37.315891	32.377010	53.406780
H	38.817074	32.134628	44.516495
O	37.805618	34.333149	48.862415
S	38.555542	29.627615	49.176769
C	37.011414	28.768589	48.744736
H	36.710865	29.036337	47.725517
H	37.124573	27.683764	48.820091
H	36.212387	29.097200	49.419601
C	38.762135	35.295818	49.029224
H	39.622612	35.151901	48.331684
H	39.249626	35.227146	50.031937
C	38.360462	36.722290	48.875641
H	39.187836	37.419468	49.016899
C	37.105469	37.290199	48.583382
C	36.992592	38.713512	48.495213
C	35.922215	36.516003	48.368183
C	35.782101	39.321320	48.213371
H	37.881859	39.318783	48.655319
C	34.717220	37.143291	48.087036
H	36.002140	35.438305	48.431572
C	34.633766	38.540131	48.007034
H	35.720337	40.404636	48.151672
H	33.826908	36.540756	47.926208
H	33.683731	39.018841	47.785442

Table S13: Cartesian coordinates (xyz, in Å) of the QM atoms of all QM/MM optimized stationary points reported in **Figure S14** and **Table S5**.

LA1-**1**^{d}

S	39.799000	27.538000	45.405998
C	40.945000	26.624001	46.512001
H	41.685001	26.086000	45.910000
H	41.467999	27.320999	47.167000
N	37.698002	28.681000	47.366001
N	38.041000	29.816999	44.755001
N	40.674999	30.653999	45.481998
C	36.441002	27.570999	48.958000
N	40.316002	29.495001	48.073002
C	35.629002	27.798000	47.897999
C	37.735001	28.121000	48.622002
C	38.846001	28.104000	49.464001
C	36.417999	28.476999	46.893002
C	35.986000	28.820000	45.618999
C	36.749001	29.400999	44.598000
C	36.293999	29.587999	43.233002
C	37.366001	30.152000	42.589001
C	38.439999	30.291000	43.534000
C	39.695000	30.841000	43.257999
C	40.728001	31.042000	44.165001
C	41.991001	31.694000	43.847000
C	42.669998	31.679001	45.035999
C	41.848999	31.054001	46.035999
C	42.205002	30.888000	47.384998
C	41.490002	30.187000	48.334000
C	41.883999	29.986000	49.710999
C	40.014999	28.834999	49.235001
C	41.014000	29.101000	50.247002
H	38.780998	27.542000	50.389999
H	34.959000	28.569000	45.375000
H	39.869999	31.164000	42.237000
H	43.152000	31.311001	47.701000
Fe	39.134998	29.757999	46.459999
C	38.924000	33.422001	50.333000
C	38.355000	34.568001	49.943001
C	37.233002	34.723999	49.002998
C	36.758999	33.672001	48.191002
C	36.598000	35.973999	48.902000
C	35.696999	33.875999	47.311001
C	35.521000	36.167999	48.037998
C	35.070000	35.123001	47.231998
H	39.737000	33.421001	51.050999
H	38.598999	32.452999	49.966000
H	38.726002	35.505001	50.362000
H	37.237000	32.698002	48.224998
H	36.953999	36.801998	49.514000
H	35.358002	33.062000	46.675999
H	35.040001	37.141998	47.995998
H	34.245998	35.275002	46.541000
O	38.562000	31.174999	47.028000

LA1-**1**^{q}

S	39.804001	27.525000	45.408001
C	40.951000	26.608000	46.511002
H	41.688999	26.069000	45.909000
H	41.474998	27.302999	47.166000
N	37.705002	28.670000	47.365002
N	38.050999	29.799999	44.752998
N	40.688999	30.629999	45.480000
C	36.444000	27.559999	48.956001
N	40.324001	29.474001	48.074001
C	35.632999	27.790001	47.896000
C	37.740002	28.107000	48.620998
C	38.849998	28.087999	49.464001
C	36.424000	28.469000	46.890999
C	35.993000	28.813000	45.618000
C	36.757999	29.391001	44.597000
C	36.304001	29.580000	43.230999
C	37.377998	30.139000	42.587002
C	38.452999	30.273001	43.530998
C	39.709999	30.820000	43.256001
C	40.742001	31.020000	44.162998
C	42.004002	31.674000	43.848000
C	42.683998	31.658001	45.036999
C	41.861000	31.031000	46.035999
C	42.216000	30.864000	47.386002
C	41.498001	30.167000	48.334000
C	41.888000	29.969000	49.713001
C	40.020000	28.816999	49.236000
C	41.016998	29.084000	50.249001
H	38.783001	27.525999	50.389999
H	34.965000	28.565001	45.374001
H	39.886002	31.143000	42.236000
H	43.161999	31.287001	47.702999
Fe	39.146000	29.733000	46.458000
C	38.951000	33.459000	50.297001
C	38.387001	34.605999	49.900002
C	37.276001	34.759998	48.945999
C	36.827999	33.710999	48.116001
C	36.622002	36.000000	48.855999
C	35.768002	33.907001	47.231998
C	35.549000	36.187000	47.986000
C	35.119999	35.145000	47.164001
H	39.754002	33.457001	51.026001
H	38.631001	32.491001	49.924000
H	38.749001	35.542999	50.327999
H	37.320999	32.743999	48.144001
H	36.957001	36.825001	49.483002
H	35.445000	33.095001	46.587002
H	35.050999	37.153999	47.953999
H	34.297001	35.290001	46.471001
O	38.585999	31.159000	47.019001

LA1-**TS1**[d]

S	39.855999	27.641001	45.418999
C	40.987000	26.707001	46.525002
H	41.728001	26.177999	45.917999
H	41.507000	27.391001	47.194000
N	37.699001	28.715000	47.407001
N	38.047001	29.839001	44.785999
N	40.674000	30.674000	45.513000
C	36.445999	27.580999	48.980999
N	40.306999	29.506001	48.104000
C	35.636002	27.816999	47.919998
C	37.741001	28.128000	48.655998
C	38.848999	28.096001	49.499001
C	36.424000	28.504000	46.919998
C	35.988998	28.840000	45.646000
C	36.750999	29.430000	44.634998
C	36.289001	29.643000	43.273998
C	37.358002	30.212000	42.632000
C	38.443001	30.333000	43.568001
C	39.693001	30.879000	43.286999
C	40.723000	31.070999	44.199001
C	41.987999	31.726000	43.888000
C	42.668999	31.702000	45.074001
C	41.853001	31.068001	46.075001
C	42.212002	30.889999	47.411999
C	41.487000	30.184999	48.356998
C	41.887001	29.971001	49.729000
C	40.012001	28.820999	49.266998
C	41.020000	29.082001	50.266998
H	38.784000	27.525000	50.417000
H	34.965000	28.579000	45.397999
H	39.865002	31.212999	42.270000
H	43.166000	31.294001	47.730000
Fe	39.145000	29.754000	46.493999
C	37.801998	31.650000	48.818001
C	38.220001	32.911999	49.174000
C	37.535000	34.147999	48.860001
C	36.436001	34.217999	47.967999
C	37.959999	35.344002	49.484001
C	35.777000	35.421001	47.744999
C	37.284000	36.539001	49.272999
C	36.187000	36.584000	48.408001
H	38.292000	30.797001	49.259998
H	36.807999	31.476000	48.423000
H	39.139000	33.011002	49.745998
H	36.109001	33.327000	47.441002
H	38.820999	35.318001	50.146000
H	34.941002	35.457001	47.049999
H	37.611000	37.443001	49.778000
H	35.665001	37.522999	48.249001
O	38.577000	31.257999	46.966999

LA1-**TS1**[q]

S	39.820000	27.584999	45.431999
C	40.957001	26.653000	46.530998
H	41.695000	26.122999	45.921001
H	41.480000	27.341000	47.195000
N	37.727001	28.705000	47.396000
N	38.068001	29.858999	44.782001
N	40.692001	30.684000	45.497002
C	36.477001	27.575001	48.980999
N	40.347000	29.504999	48.094002
C	35.655998	27.827999	47.931999
C	37.772999	28.115999	48.640999
C	38.893002	28.082001	49.473000
C	36.444000	28.518000	46.931000
C	36.007000	28.879000	45.660000
C	36.769001	29.452999	44.637001
C	36.306999	29.639000	43.271000
C	37.381001	30.184000	42.616001
C	38.464001	30.319000	43.553001
C	39.720001	30.860001	43.271000
C	40.750999	31.068001	44.181000
C	42.013000	31.726000	43.868999
C	42.688000	31.714001	45.060001
C	41.866001	31.084000	46.060001
C	42.223000	30.912001	47.403999
C	41.513000	30.198999	48.355000
C	41.915001	29.990000	49.730000
C	40.056999	28.822001	49.249001
C	41.054001	29.091000	50.262001
H	38.834000	27.504000	50.389000
H	34.977001	28.636000	45.418999
H	39.896000	31.177000	42.248001
H	43.172001	31.330999	47.720001
Fe	39.174999	29.761000	46.459000
C	37.852001	31.617001	48.830002
C	38.265999	32.874001	49.216000
C	37.584999	34.117001	48.909000
C	36.492001	34.198002	48.012001
C	38.008999	35.308998	49.541000
C	35.838001	35.405998	47.791000
C	37.339001	36.507999	49.332001
C	36.247002	36.563999	48.460999
H	38.325001	30.760000	49.283001
H	36.848000	31.455999	48.455002
H	39.187000	32.966999	49.786999
H	36.164001	33.311001	47.478001
H	38.867001	35.278000	50.207001
H	35.006001	35.446999	47.091999
H	37.667999	37.407001	49.845001
H	35.730999	37.507000	48.301998
O	38.602001	31.274000	47.009998

LA1-**Int1**^{d}

S	39.867001	27.629000	45.409000
C	40.985001	26.681000	46.519001
H	41.719002	26.142000	45.911999
H	41.514000	27.363001	47.183998
N	37.709000	28.679001	47.417000
N	38.040001	29.850000	44.794998
N	40.653999	30.683001	45.514999
C	36.445999	27.556000	48.997002
N	40.305000	29.482000	48.119999
C	35.634998	27.799000	47.938999
C	37.744999	28.094000	48.665001
C	38.859001	28.061001	49.504002
C	36.429001	28.482000	46.938999
C	35.995998	28.837000	45.667999
C	36.747002	29.436001	44.647999
C	36.272999	29.646999	43.291000
C	37.335999	30.219000	42.641998
C	38.423000	30.341999	43.575001
C	39.675999	30.893000	43.294998
C	40.712002	31.083000	44.202999
C	41.979000	31.732000	43.891998
C	42.658001	31.701000	45.080002
C	41.833000	31.066999	46.075001
C	42.189999	30.881001	47.419998
C	41.479000	30.171000	48.370998
C	41.886002	29.965000	49.744999
C	40.021999	28.801001	49.279999
C	41.025002	29.072001	50.285999
H	38.798000	27.483999	50.421001
H	34.969002	28.580999	45.423000
H	39.846001	31.226999	42.277000
H	43.141998	31.291000	47.735001
Fe	39.165001	29.687000	46.476002
C	37.868999	31.500000	48.311001
C	38.244999	32.832001	48.866001
C	37.484001	34.022999	48.792000
C	36.228001	34.105999	48.116001
C	37.970001	35.207001	49.424999
C	35.511002	35.292000	48.092999
C	37.234001	36.380001	49.410000
C	36.000000	36.432999	48.745998
H	38.169998	30.733000	49.033001
H	36.780998	31.407000	48.175999
H	39.233002	32.908001	49.313999
H	35.840000	33.237000	47.594002
H	38.930000	35.174999	49.932999
H	34.563999	35.340000	47.561001
H	37.615002	37.265999	49.910000
H	35.431999	37.359001	48.729000
O	38.537998	31.344999	47.070999

LA1-**Int1**^{q}

S	39.824001	27.646999	45.483002
C	40.964001	26.707001	46.567001
H	41.707001	26.195999	45.948002
H	41.478001	27.385000	47.248001
N	37.672001	28.724001	47.446999
N	38.023998	29.837999	44.837002
N	40.638000	30.684999	45.550999
C	36.431000	27.587000	49.022999
N	40.314999	29.445999	48.115002
C	35.611000	27.841000	47.971001
C	37.727001	28.122000	48.693001
C	38.838001	28.065001	49.525002
C	36.390999	28.527000	46.969002
C	35.946999	28.864000	45.699001
C	36.717999	29.440001	44.691002
C	36.259998	29.653000	43.332001
C	37.331001	30.209000	42.683998
C	38.422001	30.330000	43.612000
C	39.661999	30.881001	43.320000
C	40.688999	31.075001	44.235001
C	41.952999	31.728001	43.925999
C	42.639000	31.701000	45.111000
C	41.827999	31.066000	46.112999
C	42.201000	30.872999	47.438999
C	41.484001	30.145000	48.372002
C	41.877998	29.933001	49.743000
C	40.006001	28.775000	49.285000
C	41.012001	29.040001	50.280998
H	38.771000	27.495001	50.444000
H	34.921001	28.611000	45.453999
H	39.832001	31.211000	42.301998
H	43.154999	31.275999	47.757000
Fe	39.139999	29.698000	46.513000
C	37.910000	31.507999	48.415001
C	38.191002	32.905998	48.849998
C	37.337002	34.026001	48.699001
C	36.061001	33.963001	48.063000
C	37.749001	35.290001	49.221001
C	35.248001	35.084000	47.987000
C	36.923000	36.400002	49.146000
C	35.664001	36.304001	48.537998
H	38.278999	30.811001	49.174999
H	36.839001	31.316999	48.276001
H	39.172001	33.088001	49.282001
H	35.723999	33.032001	47.619999
H	38.724998	35.369999	49.693001
H	34.283001	35.019001	47.490002
H	37.254002	37.349998	49.556999
H	35.016998	37.176998	48.488998
O	38.603001	31.313999	47.181000

LA1-**TS2**^{q}

S	39.833000	27.587000	45.437000
C	40.973999	26.658001	46.534000
H	41.717999	26.127001	45.931999
H	41.487999	27.350000	47.201000
N	37.673000	28.729000	47.474998
N	37.994999	29.885000	44.879002
N	40.613998	30.719000	45.577000
C	36.443001	27.589001	49.057999
N	40.328999	29.438000	48.129002
C	35.610001	27.861000	48.021000
C	37.740002	28.113001	48.714001
C	38.860001	28.045000	49.534000
C	36.383999	28.548000	47.014000
C	35.926998	28.900999	45.752998
C	36.689999	29.483000	44.741001
C	36.223000	29.691999	43.383999
C	37.290001	30.247999	42.728001
C	38.384998	30.374001	43.650002
C	39.625000	30.924000	43.352001
C	40.659000	31.114000	44.261002
C	41.924999	31.757000	43.944000
C	42.619999	31.721001	45.125000
C	41.813999	31.087999	46.130001
C	42.199001	30.879999	47.452000
C	41.493000	30.143000	48.386002
C	41.890999	29.930000	49.756001
C	40.026001	28.761000	49.294998
C	41.029999	29.032000	50.293999
H	38.800999	27.465000	50.448002
H	34.897999	28.653000	45.516998
H	39.790001	31.254000	42.333000
H	43.155998	31.281000	47.764000
Fe	39.140999	29.693001	46.526001
C	37.858002	31.455999	48.528000
C	38.193001	32.846001	48.915001
C	37.398998	34.001999	48.691002
C	36.146999	33.963001	48.013000
C	37.839001	35.252998	49.212002
C	35.373001	35.108002	47.895000
C	37.043999	36.382999	49.112000
C	35.806999	36.316002	48.459000
H	38.222000	30.745001	49.276001
H	36.785000	31.285999	48.375000
H	39.180000	32.999001	49.345001
H	35.805000	33.035999	47.564999
H	38.801998	35.306999	49.714001
H	34.426998	35.069000	47.361000
H	37.379002	37.325001	49.536999
H	35.188000	37.206001	48.382999
O	38.590000	31.388000	47.317001

LA1-**2-S**^{d}

S	39.679001	27.391001	45.354000
C	40.853001	26.617001	46.535000
H	41.653999	26.139999	45.962002
H	41.287998	27.361000	47.201000
N	37.228001	28.393999	46.907001
N	37.848999	29.504000	44.373001
N	40.352001	30.455999	45.415001
C	35.903999	27.122999	48.320999
N	39.733002	29.311001	47.946999
C	35.257999	27.204000	47.125999
C	37.133999	27.867001	48.182999
C	38.066002	28.023001	49.202999
C	36.089001	27.982000	46.237000
C	35.804001	28.268999	44.908001
C	36.623001	28.986000	44.036999
C	36.306999	29.238001	42.640999
C	37.390999	29.919001	42.159000
C	38.344002	30.082001	43.226002
C	39.573002	30.722000	43.109001
C	40.507000	30.900000	44.123001
C	41.792000	31.562000	43.933998
C	42.397999	31.466999	45.158001
C	41.509998	30.796000	46.070000
C	41.787998	30.539000	47.409000
C	40.955002	29.860001	48.285000
C	41.249001	29.596001	49.675999
C	39.272999	28.697001	49.097000
C	40.223000	28.870001	50.178001
H	37.825001	27.577000	50.154999
H	34.868999	27.888000	44.508999
H	39.830002	31.108000	42.129002
H	42.737999	30.889999	47.796001
Fe	38.828999	29.326000	46.137001
C	36.521999	31.546000	46.651001
C	37.198002	31.514000	47.956001
C	37.637001	32.723000	48.706001
C	37.221001	34.015999	48.356998
C	38.540001	32.556000	49.769001
C	37.727001	35.125000	49.039001
C	39.043999	33.662998	50.446999
C	38.646000	34.950001	50.074001
H	35.898998	30.707001	46.361000
H	36.297001	32.493000	46.167000
H	37.081001	30.607000	48.539001
H	36.502998	34.167999	47.556000
H	38.859001	31.558001	50.053001
H	37.401001	36.124001	48.757000
H	39.751999	33.530998	51.257999
H	39.051998	35.818001	50.587002
O	37.939999	31.249001	46.706001

LA1-**2-S**[q]

S	39.793999	27.214001	45.262001
C	40.924999	26.459999	46.499001
H	41.735001	25.945000	45.976002
H	41.352001	27.229000	47.141998
N	37.261002	28.421000	46.924999
N	37.884998	29.534000	44.380001
N	40.369999	30.499001	45.407001
C	35.932999	27.150000	48.337002
N	39.755001	29.337999	47.933998
C	35.290001	27.233000	47.140999
C	37.161999	27.896000	48.200001
C	38.097000	28.046000	49.215000
C	36.123001	28.011999	46.254002
C	35.840000	28.292999	44.924999
C	36.658001	29.007000	44.049999
C	36.340000	29.249001	42.657001
C	37.416000	29.945000	42.169998
C	38.368999	30.120001	43.229000
C	39.587002	30.775999	43.105000
C	40.520000	30.955000	44.118000
C	41.803001	31.615999	43.929001
C	42.414001	31.513000	45.150002
C	41.529999	30.837999	46.061001
C	41.814999	30.570999	47.396000
C	40.985001	29.886999	48.266998
C	41.283001	29.615999	49.651001
C	39.300999	28.716999	49.094002
C	40.258999	28.884001	50.159000
H	37.862000	27.601000	50.168999
H	34.908001	27.907000	44.525002
H	39.839001	31.166000	42.125999
H	42.766998	30.917999	47.782001
Fe	38.883999	29.312000	46.124001
C	36.334999	31.739000	46.712002
C	37.078999	31.705999	47.980000
C	37.521000	32.911999	48.736000
C	37.091000	34.208000	48.415001
C	38.463001	32.737999	49.762001
C	37.617001	35.311001	49.090000
C	38.991001	33.840000	50.431999
C	38.574001	35.129002	50.090000
H	35.728001	30.882000	46.435001
H	36.035999	32.686001	46.268002
H	37.021999	30.785999	48.555000
H	36.346001	34.365002	47.639000
H	38.794998	31.739000	50.023998
H	37.278000	36.312000	48.831001
H	39.730000	33.700001	51.214001
H	38.993999	35.993000	50.598999
O	37.757999	31.493999	46.696999

LA1-**Int2**[d]

S	39.813000	27.705999	45.506001
C	40.952999	26.736000	46.577000
H	41.680000	26.209999	45.949001
H	41.487999	27.406000	47.247002
N	37.752998	28.826000	47.541000
N	38.061001	29.980000	44.910999
N	40.720001	30.702000	45.566002
C	36.504002	27.698999	49.125000
N	40.411999	29.500000	48.168999
C	35.671001	27.999001	48.099998
C	37.810001	28.212000	48.771000
C	38.937000	28.131001	49.589001
C	36.459000	28.677999	47.089001
C	35.998001	29.046000	45.833000
C	36.757000	29.601000	44.794998
C	36.271999	29.781000	43.431999
C	37.345001	30.285000	42.748001
C	38.452999	30.410999	43.665001
C	39.710999	30.913000	43.349998
C	40.762001	31.090000	44.251999
C	42.028999	31.738001	43.928001
C	42.721001	31.716999	45.112000
C	41.912998	31.084999	46.120998
C	42.289001	30.899000	47.452000
C	41.589001	30.166000	48.408001
C	42.000999	29.962999	49.785999
C	40.109001	28.827999	49.345001
C	41.131001	29.086000	50.335999
H	38.873001	27.554001	50.502998
H	34.959000	28.819000	45.612999
H	39.881001	31.219000	42.324001
H	43.250000	31.297001	47.755001
Fe	39.237000	29.729000	46.548000
C	39.054001	31.948999	48.452999
C	38.395000	33.105999	49.040001
C	37.251999	33.799000	48.583000
C	36.481998	33.335999	47.474998
C	36.839001	34.984001	49.258999
C	35.340000	34.023998	47.090000
C	35.707001	35.666000	48.851002
C	34.952999	35.182999	47.770000
H	40.148998	32.216999	48.487999
H	39.054001	31.173000	49.264000
H	38.879002	33.492001	49.937000
H	36.805000	32.441002	46.958000
H	37.431000	35.355999	50.091000
H	34.761002	33.674000	46.243000
H	35.404999	36.581001	49.353001
H	34.063999	35.723999	47.455002
O	38.661999	31.486000	47.243000

LA1-**Int2**[q]

S	39.835999	27.554001	45.382999
C	40.979000	26.643000	46.502998
H	41.716999	26.075001	45.929001
H	41.504002	27.354000	47.143002
N	37.709000	28.900999	47.485001
N	38.047001	30.021000	44.862999
N	40.717999	30.740000	45.519001
C	36.472000	27.756001	49.066002
N	40.384998	29.566000	48.112000
C	35.634998	28.063000	48.043999
C	37.773998	28.275000	48.715000
C	38.897999	28.195000	49.528000
C	36.417000	28.747999	47.035999
C	35.960999	29.110001	45.775002
C	36.735001	29.643999	44.742001
C	36.271999	29.804001	43.375000
C	37.355999	30.292000	42.693001
C	38.455002	30.430000	43.612000
C	39.716999	30.924000	43.296001
C	40.765999	31.106001	44.195999
C	42.041000	31.733999	43.867001
C	42.730999	31.719999	45.050999
C	41.917000	31.113001	46.069000
C	42.292999	30.934999	47.398998
C	41.573002	30.230000	48.354000
C	41.971001	30.020000	49.729000
C	40.075001	28.896000	49.284000
C	41.091999	29.145000	50.276001
H	38.840000	27.611000	50.439999
H	34.924999	28.882000	45.547001
H	39.893002	31.212000	42.264999
H	43.258999	31.320999	47.702999
Fe	39.216999	29.778999	46.487000
C	38.830002	32.030998	48.587002
C	38.016998	32.942001	49.374001
C	36.813999	33.592999	49.009998
C	36.301998	33.520000	47.685001
C	36.101002	34.341999	49.987999
C	35.130001	34.192001	47.359001
C	34.923000	34.986000	49.654999
C	34.442001	34.917000	48.337002
H	39.898998	32.292000	48.813999
H	38.764999	31.132000	49.284000
H	38.380001	33.098999	50.389999
H	36.849998	32.938999	46.953999
H	36.488998	34.398998	51.000999
H	34.754002	34.158001	46.341999
H	34.372002	35.551998	50.402000
H	33.523998	35.439999	48.077999
O	38.588001	31.733000	47.310001

LA1-**TS3-cis-re**[d]

S	39.832001	27.760000	45.498001
C	40.953999	26.778000	46.576000
H	41.678001	26.250000	45.948002
H	41.492001	27.443001	47.250000
N	37.756001	28.841999	47.547001
N	38.050999	30.018000	44.931000
N	40.703999	30.760000	45.584000
C	36.519001	27.702999	49.133999
N	40.426998	29.514999	48.166000
C	35.687000	27.982000	48.101002
C	37.816002	28.235001	48.786999
C	38.942001	28.174999	49.602001
C	36.466000	28.671000	47.092999
C	35.999001	29.039000	45.837002
C	36.749001	29.618999	44.811001
C	36.261002	29.809000	43.452999
C	37.326000	30.336000	42.772999
C	38.432999	30.466000	43.688999
C	39.685001	30.983000	43.370998
C	40.740002	31.152000	44.264999
C	42.013000	31.778999	43.930000
C	42.719002	31.737000	45.105000
C	41.914001	31.112000	46.119999
C	42.313999	30.903999	47.442001
C	41.613998	30.186001	48.396999
C	42.021999	29.987000	49.775002
C	40.125000	28.862000	49.341000
C	41.144001	29.120001	50.332001
H	38.882000	27.606001	50.521999
H	34.965000	28.796000	45.612999
H	39.848000	31.298000	42.346001
H	43.279999	31.295000	47.737000
Fe	39.238998	29.749001	46.558998
C	38.777000	31.771000	48.672001
C	37.969002	32.693001	49.426998
C	36.758999	33.346001	49.043999
C	36.269001	33.305000	47.713001
C	36.058998	34.106998	50.016998
C	35.129002	34.026001	47.375999
C	34.916000	34.808998	49.671001
C	34.456001	34.772999	48.347000
H	39.804001	31.698000	49.070999
H	38.248001	30.993000	49.396999
H	38.306000	32.834999	50.453999
H	36.792999	32.705002	46.980000
H	36.433998	34.139999	51.036999
H	34.769001	34.014000	46.352001
H	34.381001	35.391998	50.415001
H	33.566002	35.337002	48.075001
O	38.603001	31.514000	47.386002

LA1-**TS3-cis-re**[q]

S	39.803001	27.565001	45.410999
C	40.959000	26.649000	46.514999
H	41.698002	26.093000	45.930000
H	41.480999	27.355000	47.160000
N	37.700001	28.908001	47.542000
N	38.014000	30.052000	44.932999
N	40.682999	30.778999	45.576000
C	36.477001	27.747999	49.124001
N	40.387001	29.556000	48.147999
C	35.630001	28.066999	48.112999
C	37.775002	28.270000	48.764999
C	38.908001	28.181999	49.566002
C	36.402000	28.760000	47.105000
C	35.935001	29.131001	45.851002
C	36.702000	29.671000	44.817001
C	36.230999	29.834000	43.453999
C	37.310001	30.327999	42.768002
C	38.411999	30.468000	43.681000
C	39.667999	30.972000	43.359001
C	40.721001	31.153000	44.252998
C	41.995998	31.774000	43.916000
C	42.699001	31.745001	45.091999
C	41.894001	31.136000	46.112999
C	42.285999	30.940001	47.437000
C	41.576000	30.225000	48.388000
C	41.979000	30.010000	49.759998
C	40.084999	28.882999	49.316002
C	41.102001	29.131001	50.306000
H	38.856998	27.590000	50.473000
H	34.896999	28.905001	45.630001
H	39.835999	31.266001	42.328999
H	43.254002	31.325001	47.735001
Fe	39.202000	29.785999	46.541000
C	38.758999	31.973000	48.765999
C	37.942001	32.875000	49.554001
C	36.736000	33.526001	49.176998
C	36.258999	33.495998	47.838001
C	36.001999	34.250000	50.155998
C	35.098999	34.182999	47.501999
C	34.839001	34.916000	49.811001
C	34.390999	34.887001	48.480999
H	39.817001	32.023998	49.111000
H	38.421001	31.098000	49.452000
H	38.270000	32.990002	50.588001
H	36.818001	32.929001	47.105000
H	36.365002	34.275002	51.180000
H	34.747002	34.176998	46.474998
H	34.275002	35.466000	50.560001
H	33.483002	35.423000	48.214001
O	38.574001	31.736000	47.477001

LA1-**TS3-trans-si**[d]

S	39.845001	27.722000	45.493000
C	40.966000	26.740000	46.573002
H	41.692001	26.214001	45.944000
H	41.501999	27.405001	47.248001
N	37.754002	28.764000	47.537998
N	38.036999	29.955999	44.928001
N	40.673000	30.740000	45.597000
C	36.516998	27.642000	49.132999
N	40.409000	29.473000	48.172001
C	35.683998	27.916000	48.101002
C	37.816002	28.164000	48.778999
C	38.943001	28.098000	49.594002
C	36.464001	28.596001	47.085999
C	35.994999	28.958000	45.830002
C	36.740002	29.546000	44.804001
C	36.250000	29.743999	43.448002
C	37.307999	30.292999	42.775002
C	38.412998	30.424000	43.691002
C	39.660000	30.959000	43.381001
C	40.710999	31.135000	44.279999
C	41.977001	31.778999	43.952999
C	42.676998	31.746000	45.132000
C	41.875000	31.111000	46.141998
C	42.266998	30.909000	47.466999
C	41.580002	30.160000	48.414001
C	41.993000	29.954000	49.789001
C	40.115002	28.798000	49.342999
C	41.131001	29.066000	50.337002
H	38.883999	27.525999	50.512001
H	34.962002	28.709999	45.606998
H	39.820999	31.284000	42.359001
H	43.228001	31.309999	47.768002
Fe	39.223999	29.690001	46.556999
C	38.908001	31.865999	48.550999
C	38.383999	33.058998	49.167999
C	37.473000	34.007000	48.606998
C	36.632000	33.659000	47.516998
C	37.333000	35.282001	49.212002
C	35.660000	34.551998	47.081001
C	36.375999	36.172001	48.751999
C	35.530998	35.799999	47.698002
H	39.890999	32.539001	48.537998
H	39.224998	31.110001	49.287998
H	38.821999	33.294998	50.138000
H	36.747002	32.686001	47.053001
H	37.987000	35.560001	50.034000
H	35.006001	34.286999	46.256001
H	36.282001	37.155998	49.203999
H	34.763000	36.490002	47.361000
O	38.563000	31.452999	47.348000

LA1-**TS3-trans-si**[q]

S	39.789001	27.563999	45.417999
C	40.949001	26.648001	46.516998
H	41.688000	26.094999	45.929001
H	41.472000	27.354000	47.162998
N	37.693001	28.906000	47.558998
N	37.997002	30.052000	44.952000
N	40.665001	30.780001	45.591999
C	36.473000	27.747999	49.144001
N	40.374001	29.556000	48.160999
C	35.619999	28.077999	48.141998
C	37.772999	28.261000	48.778999
C	38.910000	28.158001	49.570999
C	36.390999	28.768000	47.130001
C	35.917999	29.143000	45.880001
C	36.682999	29.676001	44.841000
C	36.209000	29.837999	43.480000
C	37.289001	30.323999	42.787998
C	38.393002	30.464001	43.698002
C	39.650002	30.965000	43.373001
C	40.702999	31.150000	44.266998
C	41.976002	31.773001	43.929001
C	42.679001	31.749001	45.105999
C	41.875000	31.139999	46.129002
C	42.266998	30.948000	47.452999
C	41.561001	30.226999	48.402000
C	41.970001	30.003000	49.770000
C	40.081001	28.865000	49.324001
C	41.101002	29.114000	50.313000
H	38.862999	27.558001	50.473000
H	34.877998	28.922001	45.665001
H	39.817001	31.256001	42.341999
H	43.235001	31.333000	47.750000
Fe	39.189999	29.778000	46.554001
C	38.685001	32.011002	48.768002
C	37.784000	32.841999	49.547001
C	36.686001	33.623001	49.092999
C	36.250999	33.582001	47.741001
C	35.965000	34.412998	50.028999
C	35.138000	34.317001	47.351002
C	34.841999	35.118999	49.632000
C	34.433998	35.077000	48.290001
H	39.520000	32.793999	49.000999
H	39.064999	31.181999	49.401001
H	38.060001	32.932999	50.598000
H	36.807999	32.974998	47.037998
H	36.296001	34.448002	51.063000
H	34.818001	34.301998	46.313999
H	34.279999	35.710999	50.348999
H	33.561001	35.647999	47.980999
O	38.549000	31.761000	47.480000

LA1-**3**[d]

S	39.813000	27.684999	45.473000
C	40.935001	26.742001	46.578999
H	41.671001	26.219000	45.960999
H	41.458000	27.420000	47.251999
N	37.692001	28.813000	47.462002
N	38.035999	29.889000	44.827999
N	40.648998	30.702999	45.540001
C	36.445999	27.669001	49.042000
N	40.323002	29.528999	48.136002
C	35.630001	27.912001	47.984001
C	37.738998	28.216000	48.715000
C	38.848999	28.166000	49.550999
C	36.415001	28.591999	46.979000
C	35.973999	28.909000	45.702000
C	36.738998	29.476999	44.681999
C	36.276001	29.658001	43.314999
C	37.347000	30.200001	42.655998
C	38.433998	30.343000	43.590000
C	39.681999	30.881001	43.297001
C	40.712002	31.077000	44.213001
C	41.981998	31.716000	43.898998
C	42.666000	31.691999	45.087002
C	41.849998	31.076000	46.095001
C	42.226002	30.896999	47.425999
C	41.507999	30.197001	48.380001
C	41.912998	29.995001	49.756001
C	40.022999	28.865000	49.308998
C	41.040001	29.120001	50.305000
H	38.778999	27.599001	50.472000
H	34.949001	28.643000	45.459999
H	39.862000	31.190001	42.273998
H	43.188000	31.290001	47.730000
Fe	39.209999	29.627001	46.464001
C	38.198002	31.888000	48.449001
C	38.148998	33.278999	49.026001
C	36.730999	33.834999	48.941002
C	36.162998	34.091999	47.685001
C	35.985001	34.110001	50.094002
C	34.874001	34.615002	47.584000
C	34.695999	34.637001	49.993000
C	34.137001	34.889000	48.738998
H	37.820999	31.058001	49.066002
H	38.478001	33.235001	50.070999
H	38.841000	33.917999	48.466000
H	36.735001	33.903000	46.780998
H	36.417999	33.923000	51.073002
H	34.453999	34.812000	46.602001
H	34.127998	34.863998	50.891998
H	33.133999	35.305000	48.665001
O	38.622002	31.656000	47.321999

LA1-**3q**

S	39.868000	27.517000	45.381001
C	40.995998	26.613001	46.516998
H	41.736000	26.048000	45.943001
H	41.514999	27.320999	47.162998
N	37.693001	28.856001	47.507000
N	38.016998	29.966000	44.905998
N	40.645000	30.754000	45.582001
C	36.455002	27.725000	49.102001
N	40.334000	29.546000	48.138000
C	35.612999	28.033001	48.083000
C	37.756001	28.232000	48.743000
C	38.882000	28.142000	49.550999
C	36.390999	28.702000	47.067001
C	35.930000	29.049000	45.806999
C	36.702000	29.587000	44.778000
C	36.235001	29.766001	43.418999
C	37.313000	30.275000	42.741001
C	38.410999	30.407000	43.657001
C	39.660000	30.931999	43.347000
C	40.695999	31.129000	44.252998
C	41.964001	31.761999	43.928001
C	42.660000	31.738001	45.110001
C	41.853001	31.124001	46.123001
C	42.237999	30.934999	47.448002
C	41.520000	30.223000	48.389999
C	41.923000	30.004999	49.757999
C	40.043999	28.858000	49.306000
C	41.056999	29.112000	50.297001
H	38.828999	27.552000	50.458000
H	34.893002	28.823000	45.584000
H	39.831001	31.233999	42.320000
H	43.202000	31.323999	47.752998
Fe	39.228001	29.608999	46.473999
C	38.206001	32.301998	48.813999
C	38.095001	33.730000	49.303001
C	36.652000	34.196999	49.150002
C	36.106998	34.360001	47.867001
C	35.845001	34.458000	50.264000
C	34.786999	34.775002	47.702999
C	34.523998	34.880001	50.102001
C	33.991001	35.037998	48.820999
H	37.911999	31.506001	49.519001
H	38.407001	33.778999	50.352001
H	38.763000	34.360001	48.703999
H	36.723000	34.171001	46.991001
H	36.257000	34.341999	51.263000
H	34.387001	34.897999	46.700001
H	33.910999	35.097000	50.973000
H	32.963001	35.372002	48.699001
O	38.558998	32.009998	47.683998

Table S14: Cartesian coordinates (xyz, in Å) of the QM atoms of all QM/MM optimized stationary points reported in **Figures S15** and **S18**, and **Table S6**.

LA1-**1**^{d}

S	37.410000	28.740999	45.063999
C	38.047001	27.455999	46.196999
H	38.554001	26.677999	45.615002
H	38.762001	27.886999	46.896999
N	35.740002	30.410999	47.035999
N	36.544998	31.473000	44.495998
N	39.296001	31.382000	45.328999
C	34.129002	29.643000	48.520000
N	38.469002	30.287001	47.831001
C	33.477001	30.187000	47.459999
C	35.542999	29.823999	48.269001
C	36.557999	29.483000	49.153000
C	34.484001	30.658001	46.523998
C	34.244999	31.216000	45.271999
C	35.192001	31.514999	44.282001
C	34.890999	31.895000	42.907001
C	36.118000	32.074001	42.327000
C	37.136002	31.829000	43.310001
C	38.511002	31.978001	43.099998
C	39.521000	31.809999	44.042999
C	40.939999	32.053001	43.799000
C	41.539001	31.740000	44.991001
C	40.519001	31.341000	45.923000
C	40.754002	30.964001	47.251999
C	39.805000	30.500999	48.137001
C	40.067001	30.108999	49.501999
C	37.916000	29.750999	48.964001
C	38.919998	29.589001	49.987999
H	36.269001	29.033001	50.094002
H	33.208000	31.410000	45.018002
H	38.814999	32.283001	42.104000
H	41.778000	31.004000	47.606998
Fe	37.493999	31.000999	46.227001
C	35.056999	36.337002	46.481998
C	34.415001	37.011002	47.443001
C	34.919998	38.178001	48.181000
C	36.291000	38.462002	48.297001
C	34.005001	39.055000	48.789001
C	36.726002	39.595001	48.979000
C	34.438000	40.182999	49.484001
C	35.804001	40.457001	49.583000
H	36.056999	36.601002	46.148998
H	34.594002	35.490002	45.986000
H	33.403000	36.710999	47.714001
H	37.020000	37.792000	47.849998
H	32.939999	38.855999	48.688999
H	37.791000	39.804001	49.025002
H	33.719002	40.859001	49.938999
H	36.134998	41.341000	50.120998
O	37.373001	32.484001	46.884998

LA1-**1**^{q}

S	37.410999	28.746000	45.069000
C	38.049000	27.457001	46.196999
H	38.553001	26.679001	45.612000
H	38.766998	27.884001	46.896999
N	35.744999	30.413000	47.049999
N	36.542999	31.473000	44.507999
N	39.298000	31.374001	45.331001
C	34.137001	29.643999	48.535999
N	38.474998	30.281000	47.837002
C	33.483002	30.194000	47.480999
C	35.550999	29.820999	48.280998
C	36.567001	29.476000	49.161999
C	34.487999	30.665001	46.542999
C	34.245998	31.228001	45.293999
C	35.189999	31.524000	44.299999
C	34.884998	31.905001	42.925999
C	36.111000	32.076000	42.341000
C	37.131001	31.825001	43.320000
C	38.507000	31.969000	43.105000
C	39.520000	31.802000	44.043999
C	40.938000	32.048000	43.798000
C	41.539001	31.736000	44.988998
C	40.521000	31.334999	45.923000
C	40.758999	30.957001	47.251999
C	39.812000	30.496000	48.139999
C	40.077000	30.107000	49.505001
C	37.924999	29.745001	48.970001
C	38.931000	29.586000	49.993000
H	36.279999	29.023001	50.102001
H	33.209000	31.427999	45.046001
H	38.807999	32.271999	42.106998
H	41.784000	30.996000	47.604000
Fe	37.493999	30.990999	46.235001
C	35.044998	36.342999	46.474998
C	34.403000	37.015999	47.437000
C	34.910000	38.181000	48.178001
C	36.282001	38.459999	48.297001
C	33.995998	39.058998	48.785000
C	36.717999	39.591000	48.980999
C	34.431000	40.185001	49.483002
C	35.797001	40.455002	49.584999
H	36.047001	36.605999	46.145000
H	34.581001	35.499001	45.974998
H	33.389999	36.717999	47.703999
H	37.008999	37.789001	47.851002
H	32.930000	38.863998	48.682999
H	37.784000	39.797001	49.029999
H	33.713001	40.862000	49.937000
H	36.130001	41.337002	50.124001
O	37.394001	32.479000	46.887001

LA1-**TS1**^{d}

S	37.463001	28.834999	45.062000
C	38.091000	27.542000	46.195999
H	38.601002	26.767000	45.612999
H	38.804001	27.966999	46.902000
N	35.708000	30.455999	47.105000
N	36.499001	31.542000	44.551998
N	39.250000	31.466999	45.374001
C	34.097000	29.649000	48.570000
N	38.422001	30.339001	47.881001
C	33.445000	30.201000	47.513000
C	35.509998	29.834999	48.323002
C	36.530998	29.482000	49.201000
C	34.452000	30.690001	46.587002
C	34.208000	31.263000	45.339001
C	35.145000	31.597000	44.353001
C	34.833000	32.043999	42.999001
C	36.056000	32.249001	42.419998
C	37.081001	31.950001	43.382999
C	38.453999	32.098000	43.165001
C	39.471001	31.905001	44.095001
C	40.891998	32.127998	43.838001
C	41.495998	31.795000	45.021000
C	40.476002	31.402000	45.959000
C	40.709999	31.007000	47.279999
C	39.762001	30.542000	48.172001
C	40.041000	30.132000	49.528000
C	37.883999	29.768999	49.011002
C	38.900002	29.601999	50.021000
H	36.249001	29.004000	50.130001
H	33.167999	31.448999	45.091999
H	38.750999	32.428001	42.174999
H	41.737000	31.027000	47.629002
Fe	37.453999	31.030001	46.271999
C	36.735001	33.188999	48.660000
C	35.880001	34.244999	48.436001
C	36.195000	35.658001	48.523998
C	37.442001	36.146999	48.984001
C	35.202000	36.606998	48.182999
C	37.669998	37.513000	49.101002
C	35.435001	37.972000	48.304001
C	36.671001	38.433998	48.765999
H	37.730999	33.345001	49.057999
H	36.312000	32.210999	48.824001
H	34.861000	34.015999	48.125999
H	38.230000	35.453999	49.258999
H	34.237000	36.255001	47.826000
H	38.624001	37.874001	49.473999
H	34.653999	38.681999	48.041000
H	36.855999	39.500000	48.866001
O	37.382000	32.637001	46.846001

LA1-**TS1**^{q}

S	37.415001	28.857000	45.152000
C	38.050999	27.545000	46.251999
H	38.567001	26.794001	45.643002
H	38.757999	27.958000	46.970001
N	35.724998	30.468000	47.094002
N	36.537998	31.549000	44.554001
N	39.271000	31.483000	45.380001
C	34.125999	29.648001	48.556000
N	38.469002	30.322001	47.859001
C	33.470001	30.204000	47.501999
C	35.537998	29.839001	48.306999
C	36.561001	29.485001	49.182999
C	34.473000	30.702000	46.577999
C	34.230999	31.273001	45.328999
C	35.175999	31.601999	44.355999
C	34.869999	32.044998	43.000000
C	36.091999	32.249001	42.421001
C	37.119999	31.955999	43.381001
C	38.487999	32.109001	43.159000
C	39.493999	31.919001	44.097000
C	40.916000	32.141998	43.847000
C	41.520000	31.809999	45.028000
C	40.507000	31.412001	45.970001
C	40.751999	31.009001	47.277000
C	39.798000	30.532000	48.162998
C	40.066002	30.125999	49.520000
C	37.909000	29.764000	48.991001
C	38.918999	29.598000	50.006001
H	36.277000	29.009001	50.112000
H	33.193001	31.455999	45.074001
H	38.786999	32.437000	42.169998
H	41.778999	31.025000	47.624001
Fe	37.504002	31.041000	46.245998
C	36.557999	33.175999	48.639999
C	35.784000	34.279999	48.340000
C	36.167999	35.668999	48.472000
C	37.415001	36.085999	49.001999
C	35.243999	36.674000	48.096001
C	37.709999	37.436001	49.145000
C	35.543999	38.021999	48.247002
C	36.779999	38.411999	48.770000
H	37.514999	33.278999	49.139000
H	36.071999	32.221001	48.751999
H	34.787998	34.105999	47.938000
H	38.152000	35.348999	49.304001
H	34.282001	36.377998	47.686001
H	38.664001	37.738998	49.566002
H	34.813999	38.775002	47.958000
H	37.018002	39.466999	48.884998
O	37.407001	32.589001	46.931000

LA1-**Int1**^{d}

S	37.481426	28.859138	45.092632
C	38.096478	27.555204	46.222675
H	38.611534	26.789221	45.632706
H	38.805431	27.973267	46.937679
N	35.679283	30.425083	47.108547
N	36.464275	31.534065	44.542538
N	39.198261	31.477249	45.361603
C	34.074295	29.628021	48.580532
N	38.388268	30.344263	47.884617
C	33.419292	30.167957	47.517502
C	35.486290	29.816101	48.330559
C	36.510292	29.468185	49.210594
C	34.423279	30.649992	46.585514
C	34.177277	31.210949	45.331493
C	35.110279	31.559978	44.345505
C	34.786289	32.003922	42.991486
C	36.004284	32.236988	42.411510
C	37.036282	31.955070	43.372540
C	38.406277	32.125145	43.154572
C	39.421265	31.927231	44.085598
C	40.842258	32.154312	43.832626
C	41.446251	31.810373	45.011650
C	40.427250	31.408339	45.947636
C	40.669247	31.010386	47.264648
C	39.726254	30.546350	48.165638
C	40.016243	30.138403	49.520657
C	37.857277	29.766260	49.018616
C	38.880264	29.605349	50.021645
H	36.232300	28.988192	50.138596
H	33.135277	31.378878	45.080463
H	38.701279	32.465141	42.167568
H	41.698235	31.026457	47.605671
Fe	37.417274	30.951164	46.232578
C	36.968102	33.008190	48.257511
C	36.010036	34.145126	48.158463
C	36.242947	35.509148	48.447433
C	37.454903	35.988235	49.034451
C	35.230900	36.471077	48.145382
C	37.630817	37.340256	49.286419
C	35.417809	37.817097	48.409355
C	36.620770	38.262184	48.976376
H	37.878075	33.262257	48.819527
H	36.486137	32.172173	48.775524
H	35.060062	33.900059	47.685444
H	38.250942	35.293293	49.272488
H	34.302929	36.129013	47.694370
H	38.556786	37.693321	49.729431
H	34.634773	38.533047	48.169319
H	36.771706	39.321201	49.166351
O	37.266151	32.701172	46.909527

LA1-**Int1**^{q}

S	37.448002	28.834000	45.123001
C	38.076000	27.545000	46.256001
H	38.608002	26.791000	45.666000
H	38.770000	27.974001	46.978001
N	35.619999	30.422001	47.046001
N	36.452999	31.454000	44.490002
N	39.179001	31.448999	45.362000
C	34.009998	29.622000	48.498001
N	38.334999	30.320999	47.842999
C	33.366001	30.136999	47.415001
C	35.421001	29.825001	48.276001
C	36.424000	29.495001	49.178001
C	34.374001	30.620001	46.492001
C	34.136002	31.142000	45.223999
C	35.095001	31.481001	44.269001
C	34.807999	31.924999	42.911999
C	36.036999	32.167000	42.359001
C	37.054001	31.889999	43.332001
C	38.417000	32.077000	43.129002
C	39.409000	31.889999	44.082001
C	40.831001	32.120998	43.848999
C	41.426998	31.784000	45.035000
C	40.408001	31.382000	45.966000
C	40.641998	30.987000	47.273998
C	39.674999	30.521000	48.144001
C	39.931999	30.111000	49.497002
C	37.768002	29.766001	48.983002
C	38.780998	29.593000	49.987000
H	36.131001	29.045000	50.115002
H	33.098999	31.294001	44.945999
H	38.724998	32.422001	42.148998
H	41.664001	31.004000	47.636002
Fe	37.383999	30.944000	46.215000
C	36.803001	33.023998	48.188999
C	35.924999	34.220001	48.032001
C	36.228001	35.563999	48.355999
C	37.443001	35.973000	48.987000
C	35.276001	36.583000	48.047001
C	37.678001	37.310001	49.271000
C	35.521000	37.911999	48.346001
C	36.727001	38.289001	48.952000
H	37.700001	33.224998	48.785999
H	36.250000	32.216999	48.678001
H	34.980000	34.035999	47.525002
H	38.198002	35.237000	49.238998
H	34.344002	36.298000	47.563999
H	38.608002	37.605000	49.749001
H	34.778000	38.667999	48.102001
H	36.924999	39.335999	49.167999
O	37.188000	32.639000	46.868000

LA1-**TS2**^{q}

S	37.442001	28.778000	45.063999
C	38.082001	27.507999	46.216999
H	38.616001	26.737000	45.650002
H	38.772999	27.957001	46.930000
N	35.606998	30.410000	47.061001
N	36.426998	31.497000	44.515999
N	39.154999	31.490000	45.373001
C	34.005001	29.605000	48.521000
N	38.328999	30.327999	47.847000
C	33.354000	30.132999	47.449001
C	35.416000	29.802999	48.285000
C	36.429001	29.465000	49.175999
C	34.356998	30.621000	46.520000
C	34.113998	31.160000	45.261002
C	35.068001	31.514000	44.303001
C	34.769001	31.959999	42.948002
C	35.993999	32.210999	42.388000
C	37.018002	31.938000	43.355999
C	38.381001	32.126999	43.146999
C	39.379002	31.936001	44.092999
C	40.799999	32.162998	43.853001
C	41.402000	31.820000	45.034000
C	40.388000	31.416000	45.970001
C	40.629002	31.013000	47.275002
C	39.667999	30.535000	48.146999
C	39.931000	30.120001	49.497002
C	37.770000	29.753000	48.981998
C	38.785999	29.587999	49.986000
H	36.144001	28.996000	50.108002
H	33.076000	31.311001	44.987999
H	38.682999	32.474998	42.166000
H	41.653000	31.028999	47.631001
Fe	37.375000	30.937000	46.212002
C	36.848999	32.967999	48.320000
C	35.999001	34.167999	48.143002
C	36.346001	35.523998	48.375000
C	37.555000	35.918999	49.019001
C	35.431000	36.546001	47.987999
C	37.820000	37.258999	49.256001
C	35.705002	37.880001	48.238998
C	36.903999	38.245998	48.866001
H	37.766998	33.145000	48.893002
H	36.296001	32.151001	48.792000
H	35.041000	33.980000	47.662998
H	38.277000	35.168999	49.323002
H	34.507999	36.264000	47.490002
H	38.740002	37.549999	49.752998
H	34.992001	38.646000	47.943001
H	37.124001	39.294998	49.044998
O	37.119999	32.715000	46.951000

LA1-**2-R**^{d}

S	37.526001	28.874001	45.118000
C	38.096001	27.551001	46.247002
H	38.589001	26.780001	45.644001
H	38.818001	27.945000	46.960999
N	35.792000	30.514000	47.167999
N	36.560001	31.570000	44.611000
N	39.282001	31.476999	45.408001
C	34.193001	29.704000	48.643002
N	38.505001	30.316000	47.898998
C	33.534000	30.249001	47.584000
C	35.602001	29.889000	48.390999
C	36.617001	29.506001	49.257999
C	34.528999	30.725000	46.645000
C	34.264000	31.257000	45.387001
C	35.200001	31.590000	44.408001
C	34.879002	32.007000	43.046001
C	36.094002	32.227001	42.459999
C	37.130001	31.964001	43.421001
C	38.493000	32.122002	43.187000
C	39.505001	31.912001	44.118000
C	40.924000	32.125000	43.859001
C	41.535000	31.775999	45.033001
C	40.528999	31.379999	45.980999
C	40.791000	30.966999	47.280998
C	39.847000	30.502001	48.178001
C	40.130001	30.104000	49.536999
C	37.964001	29.757000	49.043999
C	38.988998	29.587000	50.042999
H	36.334999	29.027000	50.186001
H	33.220001	31.410999	45.133999
H	38.786999	32.451000	42.196999
H	41.824001	30.966000	47.610001
Fe	37.533001	30.839001	46.234001
C	37.709000	33.244999	48.868000
C	36.398998	33.629002	48.319000
C	35.993999	35.055000	48.153999
C	36.933998	36.069000	47.917000
C	34.645000	35.403000	48.306999
C	36.534000	37.401001	47.853001
C	34.247002	36.738998	48.257999
C	35.189999	37.742001	48.033001
H	38.428001	34.022999	49.118000
H	37.817001	32.312000	49.409000
H	35.578999	32.924999	48.435001
H	37.983002	35.827000	47.772999
H	33.901001	34.627998	48.472000
H	37.276001	38.174999	47.672001
H	33.201000	37.000000	48.395000
H	34.880001	38.784000	48.000000
O	37.452999	33.103001	47.452999

LA1-**2-R**^{q}

S	37.487999	28.700001	44.988998
C	38.099998	27.445000	46.176998
H	38.598999	26.632999	45.638000
H	38.813999	27.895000	46.868000
N	35.778000	30.590000	47.124001
N	36.556000	31.645000	44.591000
N	39.282001	31.521999	45.370998
C	34.174999	29.767000	48.591999
N	38.488998	30.375999	47.841999
C	33.518002	30.358000	47.556999
C	35.584999	29.940001	48.334000
C	36.597000	29.542000	49.192001
C	34.514999	30.844000	46.625000
C	34.249001	31.406000	45.382999
C	35.189999	31.716000	44.401001
C	34.876999	32.152000	43.049999
C	36.095001	32.333000	42.451000
C	37.131001	32.035999	43.396000
C	38.493000	32.174000	43.152000
C	39.502998	31.962000	44.082001
C	40.921001	32.171001	43.821999
C	41.534000	31.822001	44.997002
C	40.529999	31.429001	45.945000
C	40.789001	31.018999	47.244999
C	39.834999	30.562000	48.132999
C	40.105000	30.156000	49.487000
C	37.941002	29.809999	48.984001
C	38.958000	29.632999	49.983002
H	36.317001	29.048000	50.112999
H	33.209000	31.594999	45.141998
H	38.785000	32.501999	42.160999
H	41.820000	31.011999	47.577999
Fe	37.526001	30.823999	46.152000
C	38.166000	33.578999	49.432999
C	36.860001	34.171001	49.090000
C	36.654999	35.645000	48.986000
C	37.640999	36.491001	48.457001
C	35.458000	36.206001	49.449001
C	37.439999	37.869999	48.417000
C	35.261002	37.587002	49.418999
C	36.255001	38.423000	48.909000
H	39.014999	34.241001	49.592999
H	38.212002	32.618999	49.941002
H	35.967999	33.601002	49.339001
H	38.561001	36.073002	48.061001
H	34.674000	35.562000	49.838001
H	38.207001	38.516998	47.999001
H	34.331001	38.013000	49.790001
H	36.106998	39.500000	48.896999
O	37.675999	33.529999	48.082001

LA1-**Int2**^{d}

S	37.299000	28.745001	44.976002
C	38.063999	27.582001	46.175999
H	38.660000	26.841999	45.630001
H	38.710999	28.108999	46.874001
N	35.174999	30.400999	46.542999
N	36.240002	31.375000	44.076000
N	38.891998	31.417000	45.234001
C	33.441002	29.643999	47.869999
N	37.823002	30.357000	47.662998
C	32.896000	30.061001	46.695000
C	34.868000	29.875000	47.776001
C	35.775002	29.615999	48.806000
C	33.979000	30.516001	45.847000
C	33.868999	30.978001	44.541000
C	34.923000	31.351999	43.693001
C	34.773998	31.788000	42.305000
C	36.049000	32.070999	41.900002
C	36.956001	31.823999	42.990002
C	38.328999	32.037998	42.939999
C	39.237000	31.851999	43.981998
C	40.679001	32.070999	43.868000
C	41.169998	31.719000	45.096001
C	40.067001	31.325001	45.937000
C	40.181999	30.903000	47.255001
C	39.137001	30.455000	48.056000
C	39.285999	29.990999	49.417000
C	37.141998	29.841000	48.750000
C	38.064999	29.589001	49.839001
H	35.373001	29.202000	49.719002
H	32.866001	31.073000	44.139999
H	38.729000	32.388000	41.994999
H	41.174999	30.900000	47.689999
Fe	37.025002	30.871000	45.882000
C	35.597000	33.293999	46.452000
C	35.435001	34.613998	47.053001
C	36.286999	35.282001	47.957001
C	37.397999	34.634998	48.584999
C	36.051998	36.662998	48.230999
C	38.227001	35.353001	49.433998
C	36.914001	37.372002	49.047001
C	38.005001	36.720001	49.645000
H	34.743999	32.685001	46.876999
H	35.242001	33.390999	45.397999
H	34.550999	35.160000	46.719002
H	37.570999	33.587002	48.370998
H	35.208000	37.160999	47.761002
H	39.066002	34.862000	49.917000
H	36.757999	38.433998	49.216000
H	38.688000	37.292000	50.265999
O	36.790001	32.665001	46.585999

LA1-**Int2**[q]

S	37.299000	28.598000	44.873001
C	38.007000	27.452999	46.124001
H	38.571999	26.653000	45.632999
H	38.678001	27.993000	46.792999
N	35.228001	30.469000	46.605000
N	36.299000	31.431000	44.110001
N	38.957001	31.438999	45.223999
C	33.493999	29.726999	47.942001
N	37.884998	30.368999	47.660000
C	32.950001	30.159000	46.771000
C	34.922001	29.940001	47.838001
C	35.839001	29.655001	48.846001
C	34.036999	30.601999	45.918999
C	33.924999	31.056000	44.606998
C	34.973000	31.399000	43.744999
C	34.814999	31.792000	42.348999
C	36.085999	32.066002	41.922001
C	37.005001	31.849001	43.004002
C	38.380001	32.055000	42.931000
C	39.297001	31.862000	43.963001
C	40.737999	32.058998	43.839001
C	41.234001	31.704000	45.068001
C	40.136002	31.326000	45.918999
C	40.254002	30.899000	47.236000
C	39.209999	30.458000	48.037998
C	39.362000	29.995001	49.395000
C	37.209000	29.856001	48.757999
C	38.141998	29.594000	49.827999
H	35.449001	29.247999	49.766998
H	32.919998	31.153999	44.209999
H	38.771000	32.390999	41.978001
H	41.249001	30.882999	47.664001
Fe	37.091999	30.913000	45.904999
C	35.609001	33.522999	46.547001
C	35.403999	34.841999	47.139999
C	36.275002	35.558998	47.985001
C	37.459000	34.965000	48.521999
C	35.978001	36.918999	48.298000
C	38.294998	35.713001	49.338001
C	36.845001	37.660000	49.077999
C	38.004002	37.056000	49.596001
H	34.742001	32.924999	46.966000
H	35.235001	33.627998	45.492001
H	34.470001	35.331001	46.860001
H	37.669998	33.931000	48.279999
H	35.077000	37.373001	47.891998
H	39.188999	35.264999	49.758999
H	36.639000	38.707001	49.284000
H	38.687000	37.648998	50.198002
O	36.789001	32.901001	46.654999

LA1-**TS3-cis-re**[d]

S	37.238998	28.731001	44.957001
C	37.987000	27.563999	46.160999
H	38.561001	26.805000	45.617001
H	38.653999	28.082001	46.848000
N	35.146999	30.398001	46.543999
N	36.195000	31.351000	44.068001
N	38.856998	31.382999	45.208000
C	33.421001	29.677000	47.903999
N	37.799999	30.348000	47.651001
C	32.872002	30.070000	46.722000
C	34.846001	29.900999	47.797001
C	35.755001	29.650000	48.823002
C	33.952999	30.500000	45.859001
C	33.831001	30.927999	44.537998
C	34.875000	31.292000	43.681000
C	34.723999	31.698999	42.286999
C	35.993999	32.005001	41.880001
C	36.903999	31.791000	42.973999
C	38.277000	32.016998	42.917999
C	39.192001	31.826000	43.952000
C	40.632000	32.049000	43.833000
C	41.131001	31.687000	45.056999
C	40.035999	31.287001	45.902000
C	40.161999	30.865999	47.222000
C	39.123001	30.434999	48.030998
C	39.276001	29.983999	49.396000
C	37.125999	29.854000	48.749001
C	38.055000	29.600000	49.832001
H	35.355999	29.261999	49.747002
H	32.825001	31.002001	44.138000
H	38.671001	32.368999	41.971001
H	41.159000	30.860001	47.646999
Fe	36.993999	30.832001	45.863998
C	35.660999	33.368999	46.379002
C	35.455002	34.682999	46.939999
C	36.245998	35.348000	47.923000
C	37.380001	34.741001	48.532001
C	35.943001	36.703999	48.216000
C	38.187000	35.486000	49.382000
C	36.778999	37.444000	49.036999
C	37.903000	36.835999	49.613998
H	34.776001	33.123001	47.143002
H	35.132999	33.216000	45.422001
H	34.563000	35.195000	46.580002
H	37.594002	33.700001	48.324001
H	35.075001	37.171001	47.756001
H	39.050999	35.027000	49.848999
H	36.570999	38.493999	49.223000
H	38.564999	37.428001	50.240002
O	36.764999	32.685001	46.595001

LA1-**TS3-cis-re**[q]

S	37.312000	28.577999	44.855999
C	38.011002	27.441000	46.119999
H	38.575001	26.636999	45.634998
H	38.681999	27.983000	46.786999
N	35.205002	30.433001	46.587002
N	36.264999	31.400999	44.095001
N	38.921001	31.427999	45.214001
C	33.472000	29.707001	47.935001
N	37.855000	30.361000	47.651001
C	32.928001	30.122999	46.758999
C	34.898998	29.920000	47.830002
C	35.813000	29.646000	48.841999
C	34.014999	30.556999	45.902000
C	33.896000	30.996000	44.584999
C	34.939999	31.347000	43.723000
C	34.782001	31.740000	42.328999
C	36.049999	32.037998	41.907001
C	36.967999	31.830999	42.992001
C	38.341000	32.054001	42.923000
C	39.257999	31.862000	43.952999
C	40.698002	32.067001	43.831001
C	41.195999	31.705999	45.056999
C	40.101002	31.319000	45.905998
C	40.223000	30.889999	47.223000
C	39.182999	30.450001	48.026001
C	39.337002	29.990000	49.383999
C	37.181999	29.850000	48.752998
C	38.117001	29.591999	49.820999
H	35.423000	29.246000	49.764999
H	32.890999	31.080000	44.188000
H	38.730999	32.398998	41.972000
H	41.220001	30.875999	47.648998
Fe	37.063000	30.862000	45.882999
C	35.632999	33.591000	46.459999
C	35.437000	34.923000	47.005001
C	36.256001	35.598999	47.946999
C	37.418999	34.997002	48.507999
C	35.952000	36.951000	48.264999
C	38.248001	35.741001	49.337002
C	36.807999	37.688999	49.063999
C	37.958000	37.084000	49.595001
H	34.752998	33.287998	47.185001
H	35.112000	33.469002	45.487999
H	34.525002	35.422001	46.675999
H	37.629002	33.960999	48.278999
H	35.062000	37.412998	47.842999
H	39.133999	35.286999	49.768002
H	36.598999	38.735001	49.271000
H	38.633999	37.672001	50.208000
O	36.742001	32.908001	46.643002

LA1-**TS3-trans-si**[d]

S	37.316002	28.707001	44.931000
C	37.976002	27.514999	46.160999
H	38.540001	26.746000	45.619999
H	38.650002	28.014000	46.855000
N	35.172001	30.285000	46.554001
N	36.193001	31.319000	44.066002
N	38.827000	31.393000	45.196999
C	33.437000	29.597000	47.919998
N	37.792999	30.295000	47.653999
C	32.895000	29.965000	46.728001
C	34.862000	29.818001	47.818001
C	35.76698	29.589001	48.848999
C	33.980999	30.382999	45.862999
C	33.849998	30.812000	44.540001
C	34.877998	31.215000	43.682999
C	34.706001	31.624001	42.291000
C	35.962002	31.975000	41.881001
C	36.882000	31.787001	42.971001
C	38.248001	32.049999	42.914001
C	39.162998	31.857000	43.946999
C	40.599998	32.083000	43.830002
C	41.099998	31.702000	45.049000
C	40.007000	31.285000	45.888000
C	40.143002	30.843000	47.202000
C	39.117001	30.396999	48.019001
C	39.289001	29.952999	49.387001
C	37.134998	29.802999	48.764000
C	38.076000	29.565001	49.839001
H	35.370998	29.211000	49.779999
H	32.841000	30.865999	44.146000
H	38.634998	32.418999	41.971001
H	41.143002	30.840000	47.618999
Fe	36.988998	30.774000	45.862999
C	35.608002	33.331001	46.403000
C	35.366001	34.604000	47.055000
C	36.265999	35.323002	47.893002
C	37.344002	34.668999	48.553001
C	36.069000	36.716000	48.096001
C	38.194000	35.397999	49.376999
C	36.938000	37.431999	48.901001
C	38.002998	36.773998	49.535999
H	34.679001	32.800999	46.125000
H	35.722000	34.036999	45.460999
H	34.457001	35.111000	46.733002
H	37.483002	33.605999	48.402000
H	35.243999	37.216999	47.596001
H	39.014000	34.902000	49.883999
H	36.806000	38.502998	49.032001
H	38.688000	37.348999	50.153999
O	36.682999	32.612000	46.644001

LA1-**TS3-trans-si**[q]

S	37.320000	28.580000	44.855999
C	38.018002	27.438999	46.118000
H	38.582001	26.636000	45.631001
H	38.688000	27.979000	46.786999
N	35.201000	30.403000	46.591999
N	36.254002	31.414000	44.116001
N	38.908001	31.447001	45.234001
C	33.469002	29.680000	47.943001
N	37.849998	30.344999	47.660000
C	32.924000	30.093000	46.766998
C	34.896000	29.893999	47.837002
C	35.811001	29.622999	48.849998
C	34.009998	30.528000	45.910000
C	33.889000	30.976999	44.595001
C	34.931000	31.343000	43.740002
C	34.771999	31.731001	42.344002
C	36.039001	32.040001	41.924999
C	36.955002	31.846001	43.013000
C	38.327000	32.077999	42.944000
C	39.243999	31.886000	43.973999
C	40.683998	32.088001	43.848999
C	41.183998	31.719999	45.073002
C	40.090000	31.330999	45.922001
C	40.215000	30.891001	47.235001
C	39.176998	30.441000	48.036999
C	39.333000	29.979000	49.393002
C	37.179001	29.830999	48.761002
C	38.113998	29.577000	49.830002
H	35.421001	29.223000	49.772999
H	32.882999	31.055000	44.196999
H	38.715000	32.424000	41.993000
H	41.212002	30.877001	47.660000
Fe	37.055000	30.860001	45.898998
C	35.589001	33.542000	46.624001
C	35.335999	34.800999	47.310001
C	36.258999	35.554001	48.080002
C	37.430000	34.949001	48.618000
C	36.001999	36.928001	48.340000
C	38.305000	35.705002	49.388000
C	36.900002	37.674999	49.081001
C	38.050999	37.063000	49.603001
H	34.659000	32.945000	46.514000
H	35.521000	34.161999	45.636002
H	34.377998	35.264999	47.071999
H	37.611000	33.900002	48.418999
H	35.106998	37.390999	47.931000
H	39.193001	35.249001	49.810001
H	36.722000	38.734001	49.252998
H	38.754002	37.658001	50.178001
O	36.720001	32.877998	46.743999

LA1-**3**[d]

S	37.415001	28.669001	44.886002
C	38.023998	27.506001	46.161999
H	38.588001	26.721001	45.646000
H	38.692001	28.010000	46.858002
N	35.173000	30.214001	46.476002
N	36.201000	31.228001	43.986000
N	38.806999	31.398001	45.141998
C	33.430000	29.544001	47.845001
N	37.769001	30.312000	47.598000
C	32.898998	29.850000	46.630001
C	34.848999	29.788000	47.754002
C	35.744999	29.608000	48.801998
C	33.987000	30.256001	45.764000
C	33.863998	30.657000	44.433998
C	34.889000	31.099001	43.594002
C	34.709999	31.542999	42.215000
C	35.957001	31.944000	41.820000
C	36.879002	31.747999	42.904999
C	38.237000	32.047001	42.856998
C	39.148998	31.863001	43.890999
C	40.584000	32.099998	43.783001
C	41.081001	31.716999	45.001999
C	39.987999	31.292999	45.835999
C	40.122002	30.854000	47.148998
C	39.094002	30.415001	47.966000
C	39.264999	29.982000	49.334999
C	37.111000	29.827000	48.716999
C	38.050999	29.599001	49.792999
H	35.346001	29.256001	49.742001
H	32.859001	30.681000	44.028000
H	38.618999	32.438999	41.921001
H	41.120998	30.858000	47.570000
Fe	36.992001	30.698000	45.776001
C	35.466999	33.240002	46.492001
C	35.161999	34.527000	47.210999
C	36.313999	35.130001	47.978001
C	36.971001	34.389000	48.973000
C	36.751999	36.429001	47.695000
C	38.057999	34.930000	49.655998
C	37.827000	36.980999	48.396000
C	38.484001	36.230999	49.370998
H	34.304001	34.292999	47.865002
H	34.637001	32.783001	45.935001
H	34.757999	35.228001	46.463001
H	36.641998	33.381001	49.205002
H	36.254002	37.011002	46.922001
H	38.574001	34.337002	50.403000
H	38.150002	37.994999	48.169998
H	39.330002	36.658001	49.903000
O	36.558998	32.686001	46.526001

LA1-**3q**

S	37.397999	28.493999	44.804001
C	38.056000	27.382000	46.106998
H	38.611000	26.559999	45.640999
H	38.727001	27.924000	46.771999
N	35.205002	30.319000	46.506001
N	36.258999	31.292999	44.028999
N	38.879002	31.407000	45.175999
C	33.460999	29.632000	47.867001
N	37.820000	30.333000	47.598999
C	32.924999	30.004999	46.673000
C	34.884998	29.846001	47.766998
C	35.786999	29.607000	48.796001
C	34.014000	30.417000	45.813000
C	33.895000	30.837999	44.491001
C	34.936001	31.218000	43.644001
C	34.775002	31.636000	42.258999
C	36.036999	31.978001	41.853001
C	36.952999	31.771000	42.937000
C	38.317001	32.034000	42.880001
C	39.223999	31.856001	43.917999
C	40.659000	32.088001	43.810001
C	41.153999	31.724001	45.035999
C	40.060001	31.315001	45.873001
C	40.187000	30.882999	47.187000
C	39.148998	30.434999	47.985001
C	39.303001	29.985001	49.341999
C	37.153000	29.823000	48.709999
C	38.084000	29.582001	49.780998
H	35.393002	29.226999	49.726002
H	32.890999	30.903000	44.087002
H	38.705002	32.403999	41.938000
H	41.181999	30.886000	47.618000
Fe	37.058998	30.688999	45.785000
C	35.382000	33.544998	46.771000
C	35.101002	34.757000	47.630001
C	36.308998	35.327000	48.334999
C	37.089001	34.511002	49.167999
C	36.681999	36.661999	48.146999
C	38.236000	35.014000	49.777000
C	37.817001	37.175999	48.778000
C	38.599998	36.351002	49.584999
H	34.312000	34.445000	48.334999
H	34.499001	33.084000	46.291000
H	34.619999	35.507000	46.983002
H	36.808998	33.474998	49.327999
H	36.084000	37.304001	47.502998
H	38.847000	34.367001	50.396999
H	38.091000	38.217999	48.629002
H	39.493000	36.747002	50.060001
O	36.486000	33.056000	46.610001

Table S15: Cartesian coordinates (xyz, in Å) of the QM atoms of all QM/MM optimized stationary points reported in **Figures S19** and **S22,** and **Table S7**.

aMOx-**1**^{d}

S	38.126999	29.646000	49.098999
C	38.959000	29.124001	50.644001
H	39.398998	28.132000	50.486000
H	39.751999	29.818001	50.922001
N	36.367001	32.073002	50.259998
N	36.743000	31.622999	47.455002
N	39.578999	32.029999	47.777000
C	35.002998	32.442001	52.105000
N	39.172001	32.409000	50.591000
C	34.195000	32.078999	51.082001
C	36.349998	32.442001	51.581001
C	37.473999	32.778000	52.330002
C	35.040001	31.862000	49.922001
C	34.609001	31.535000	48.646999
C	35.393002	31.447001	47.481998
C	34.866001	31.198000	46.147999
C	35.960999	31.278999	45.327999
C	37.120998	31.528999	46.139000
C	38.422001	31.669001	45.661999
C	39.573002	31.884001	46.419998
C	40.922001	31.903999	45.870998
C	41.724998	32.046001	46.970001
C	40.889999	32.133999	48.141998
C	41.340000	32.323002	49.453999
C	40.549000	32.509998	50.577999
C	41.023998	32.915001	51.888000
C	38.785000	32.749001	51.863998
C	39.933998	33.083000	52.673000
H	37.316002	33.089001	53.355000
H	33.540001	31.388000	48.523998
H	38.557999	31.576000	44.590000
H	42.411999	32.368999	49.603001
Fe	37.948002	32.120998	49.002998
C	36.832001	35.960999	50.279999
C	37.174999	37.234001	50.064999
C	36.268002	38.330002	49.716999
C	36.818001	39.519001	49.214001
C	34.868999	38.252998	49.859001
C	36.001999	40.585999	48.855999
C	34.053001	39.321999	49.505001
C	34.617001	40.497002	49.000000
H	37.580002	35.222000	50.525002
H	35.807999	35.605999	50.201000
H	38.228001	37.506001	50.105999
H	37.893002	39.589001	49.069000
H	34.417999	37.348000	50.255001
H	36.462002	41.473999	48.428001
H	32.976002	39.245998	49.612999
H	33.973999	41.324001	48.710999
O	37.758999	33.714001	48.723000

aMOx-**1**^{q}

S	38.119999	29.658001	49.104000
C	38.956001	29.132999	50.645000
H	39.396000	28.141001	50.483002
H	39.748001	29.826000	50.924000
N	36.359001	32.071999	50.261002
N	36.736000	31.625999	47.457001
N	39.570999	32.036999	47.778000
C	34.994999	32.443001	52.106998
N	39.165001	32.410999	50.591999
C	34.187000	32.078999	51.084000
C	36.342999	32.442001	51.582001
C	37.466000	32.779999	52.330002
C	35.032001	31.860001	49.924000
C	34.602001	31.533001	48.648998
C	35.386002	31.445999	47.484001
C	34.859001	31.195000	46.150002
C	35.953999	31.278999	45.330002
C	37.112999	31.533001	46.139999
C	38.414001	31.676001	45.662998
C	39.564999	31.892000	46.419998
C	40.914001	31.910000	45.872002
C	41.716999	32.049000	46.972000
C	40.882000	32.137001	48.144001
C	41.333000	32.320999	49.455002
C	40.542000	32.511002	50.580002
C	41.016998	32.916000	51.889999
C	38.778000	32.751999	51.865002
C	39.926998	33.085999	52.674000
H	37.307999	33.091999	53.355999
H	33.533001	31.385000	48.526001
H	38.549999	31.583000	44.591000
H	42.403999	32.365002	49.605000
Fe	37.939999	32.117001	49.004002
C	36.869999	35.979000	50.298000
C	37.202999	37.251999	50.074001
C	36.287998	38.341000	49.720001
C	36.830002	39.530998	49.210999
C	34.889999	38.255001	49.865002
C	36.007000	40.591000	48.849998
C	34.067001	39.318001	49.507000
C	34.622002	40.493999	48.995998
H	37.625000	35.247002	50.547001
H	35.848999	35.616001	50.224998
H	38.254002	37.533001	50.110001
H	37.903999	39.605999	49.063999
H	34.445000	37.349998	50.265999
H	36.460999	41.480000	48.416000
H	32.990002	39.237000	49.616001
H	33.973999	41.316002	48.703999
O	37.751999	33.714001	48.737999

aMOx-**TS1**^{d}

S	38.063000	29.976999	49.179001
C	38.916000	29.389999	50.689999
H	39.379002	28.422001	50.465000
H	39.695999	30.084999	51.000999
N	36.352001	32.377998	50.375000
N	36.720001	31.936001	47.574001
N	39.556999	32.300999	47.893002
C	35.002998	32.632999	52.245998
N	39.150002	32.647999	50.708000
C	34.193001	32.313999	51.207001
C	36.348000	32.668999	51.723000
C	37.469002	32.958000	52.484001
C	35.030998	32.150002	50.035999
C	34.588001	31.820000	48.764000
C	35.368999	31.733999	47.608002
C	34.851002	31.436001	46.279999
C	35.945000	31.514999	45.459999
C	37.103001	31.805000	46.261002
C	38.398998	31.931000	45.775002
C	39.544998	32.139000	46.535000
C	40.896000	32.105000	45.991001
C	41.702000	32.217999	47.090000
C	40.873001	32.348000	48.264000
C	41.327000	32.507000	49.571999
C	40.532001	32.702999	50.692001
C	41.014999	33.046001	52.014999
C	38.771000	32.933998	52.004002
C	39.931999	33.209000	52.812000
H	37.320000	33.207001	53.526001
H	33.521000	31.653000	48.650002
H	38.535000	31.808001	44.707001
H	42.397999	32.507999	49.730000
Fe	37.926998	32.421001	49.137001
C	37.547001	35.554001	50.217999
C	37.355999	36.778999	49.623001
C	36.098999	37.485001	49.526001
C	36.115002	38.838001	49.116001
C	34.862000	36.923000	49.924000
C	34.958000	39.605999	49.155998
C	33.714001	37.701000	49.969002
C	33.755001	39.047001	49.590000
H	38.535999	35.263000	50.535999
H	36.727001	35.037998	50.695000
H	38.224998	37.317001	49.247002
H	37.053001	39.276001	48.779999
H	34.806999	35.877998	50.203999
H	34.985001	40.650002	48.854000
H	32.778999	37.265999	50.306000
H	32.858002	39.652000	49.615002
O	37.786999	34.061001	48.796001

aMOx-**TS1**^{q}

S	37.995998	30.016001	49.245998
C	38.855999	29.421000	50.743999
H	39.332001	28.462999	50.499001
H	39.624001	30.122999	51.064999
N	36.355000	32.388000	50.346001
N	36.734001	31.978001	47.542999
N	39.556999	32.334000	47.853001
C	35.021000	32.623001	52.230000
N	39.176998	32.639999	50.680000
C	34.202999	32.326000	51.192001
C	36.362999	32.658001	51.695999
C	37.491001	32.931999	52.453999
C	35.035999	32.180000	50.014000
C	34.591000	31.875000	48.733002
C	35.375000	31.781000	47.582001
C	34.855999	31.473000	46.257000
C	35.948002	31.537001	45.433998
C	37.109001	31.830000	46.228001
C	38.403000	31.951000	45.737000
C	39.549000	32.165001	46.494999
C	40.900002	32.129002	45.952000
C	41.705002	32.248001	47.050999
C	40.876999	32.380001	48.224998
C	41.339001	32.535999	49.529999
C	40.551998	32.717999	50.660999
C	41.037998	33.063999	51.983002
C	38.796001	32.918999	51.973000
C	39.953999	33.206001	52.784000
H	37.346001	33.171001	53.499001
H	33.522999	31.722000	48.612000
H	38.536999	31.822001	44.668999
H	42.410999	32.540001	49.680000
Fe	37.943001	32.428001	49.076000
C	37.535000	35.535000	50.271999
C	37.355000	36.777000	49.695000
C	36.101002	37.480999	49.571999
C	36.123001	38.833000	49.155998
C	34.855000	36.924000	49.952000
C	34.966000	39.602001	49.168999
C	33.708000	37.702999	49.972000
C	33.754002	39.046001	49.584999
H	38.513000	35.264000	50.638000
H	36.703999	35.021000	50.730999
H	38.233002	37.323002	49.355000
H	37.066002	39.270000	48.834999
H	34.792999	35.881001	50.240002
H	34.999001	40.643002	48.862000
H	32.766998	37.270000	50.298000
H	32.855999	39.652000	49.590000
O	37.816002	34.110001	48.875999

aMOx-**Int1**^{d}

S	37.983002	29.915001	49.196999
C	38.891998	29.327000	50.680000
H	39.348000	28.356001	50.449001
H	39.674999	30.025999	50.971001
N	36.247002	32.271000	50.437000
N	36.584000	31.898001	47.640999
N	39.445000	32.305000	47.936001
C	34.924000	32.547001	52.326000
N	39.077999	32.584000	50.750000
C	34.098000	32.217999	51.305000
C	36.262001	32.576000	51.783001
C	37.395000	32.891998	52.516998
C	34.917999	32.049000	50.124001
C	34.465000	31.743000	48.848000
C	35.238998	31.663000	47.679001
C	34.722000	31.327999	46.362999
C	35.816002	31.403999	45.539001
C	36.966000	31.740000	46.333000
C	38.265999	31.886999	45.841999
C	39.419998	32.132999	46.578999
C	40.764000	32.122002	46.011002
C	41.582001	32.256001	47.099998
C	40.761002	32.369999	48.282001
C	41.236000	32.525002	49.591000
C	40.456001	32.686001	50.723999
C	40.945000	33.060001	52.040001
C	38.702999	32.888000	52.029999
C	39.861000	33.207001	52.837002
H	37.252998	33.158001	53.556999
H	33.397999	31.576000	48.737999
H	38.396000	31.743999	44.775002
H	42.310001	32.550999	49.730000
Fe	37.820000	32.304001	49.189999
C	36.543999	34.907001	49.316002
C	36.893002	36.337002	49.493000
C	36.028999	37.452999	49.548000
C	36.608002	38.741001	49.735001
C	34.605999	37.365002	49.500999
C	35.817001	39.865002	49.893002
C	33.827000	38.497002	49.689999
C	34.421001	39.750999	49.886002
H	36.087002	34.556999	50.262001
H	35.733002	34.786999	48.574001
H	37.948002	36.537998	49.654999
H	37.691002	38.830002	49.738998
H	34.131001	36.405998	49.325001
H	36.284000	40.837002	50.030998
H	32.743999	38.426998	49.688000
H	33.798000	40.631001	50.020000
O	37.689999	34.172001	49.002998

aMOx-**Int1**^{q}

S	38.008999	30.009001	49.249001
C	38.858002	29.431000	50.757999
H	39.344002	28.479000	50.508999
H	39.618999	30.138000	51.084999
N	36.237999	32.259998	50.446999
N	36.583000	31.855000	47.651001
N	39.402000	32.363998	47.938000
C	34.910000	32.527000	52.321999
N	39.033001	32.615002	50.754002
C	34.099998	32.140999	51.305000
C	36.243999	32.597000	51.789001
C	37.356998	32.950001	52.529999
C	34.921001	31.976999	50.127998
C	34.469002	31.622000	48.866001
C	35.240002	31.583000	47.702999
C	34.726002	31.273001	46.381001
C	35.806000	31.409000	45.548000
C	36.959000	31.753000	46.332001
C	38.237999	31.948999	45.826000
C	39.379002	32.198002	46.577000
C	40.726002	32.188000	46.021999
C	41.541000	32.299999	47.115002
C	40.724998	32.407001	48.298000
C	41.202999	32.536999	49.597000
C	40.411999	32.706001	50.723000
C	40.896000	33.076000	52.036999
C	38.652000	32.936001	52.042000
C	39.814999	33.240002	52.837002
H	37.209000	33.228001	53.564999
H	33.408001	31.419001	48.763000
H	38.368000	31.834000	44.756001
H	42.276001	32.549000	49.741001
Fe	37.786999	32.337002	49.206001
C	36.473999	34.946999	49.384998
C	36.896000	36.379002	49.452999
C	36.041000	37.500999	49.541000
C	36.627998	38.792000	49.695999
C	34.616001	37.430000	49.535000
C	35.848000	39.921001	49.866001
C	33.847000	38.570999	49.717999
C	34.449001	39.825001	49.889000
H	36.118999	34.631001	50.375999
H	35.624001	34.803001	48.700001
H	37.959000	36.564999	49.561001
H	37.712002	38.877998	49.671001
H	34.125000	36.473999	49.384998
H	36.328999	40.888000	49.988998
H	32.763000	38.505001	49.728001
H	33.837002	40.712002	50.025002
O	37.566002	34.138000	48.978001

aMOx-**TS-rotation**^{d}

S	38.002087	29.767460	49.151737
C	38.881031	29.270708	50.681366
H	39.357658	28.299742	50.500542
H	39.645802	29.994646	50.959263
N	36.149788	32.089813	50.227253
N	36.553005	31.585133	47.448318
N	39.380875	32.108219	47.791580
C	34.782070	32.438728	52.069244
N	38.949913	32.556328	50.567970
C	33.985497	32.037300	51.049252
C	36.130329	32.475979	51.548988
C	37.233990	32.858528	52.298576
C	34.836983	31.824940	49.892067
C	34.414009	31.452093	48.621765
C	35.207973	31.364742	47.469563
C	34.702572	31.064125	46.133030
C	35.804462	31.154913	45.324821
C	36.950817	31.464493	46.143135
C	38.252396	31.630722	45.673695
C	39.385506	31.911085	46.435947
C	40.741020	31.949356	45.898773
C	41.533642	32.134293	47.001095
C	40.691849	32.236359	48.166290
C	41.138969	32.452579	49.472820
C	40.327915	32.655556	50.578671
C	40.783756	33.059685	51.897205
C	38.543522	32.874420	51.845570
C	39.682541	33.221180	52.669197
H	37.062206	33.156807	53.324265
H	33.350780	31.264929	48.501083
H	38.407200	31.492109	44.609188
H	42.210350	32.495922	49.633202
Fe	37.745632	32.059376	49.007801
C	38.158058	34.909389	49.017178
C	37.491215	36.132545	48.501152
C	36.211067	36.595428	48.870773
C	35.824718	37.909016	48.478939
C	35.318401	35.831535	49.678776
C	34.639046	38.457699	48.938923
C	34.126453	36.384560	50.114941
C	33.791267	37.699951	49.758801
H	39.179211	34.830502	48.626945
H	38.223427	34.951035	50.117760
H	38.021320	36.735939	47.761806
H	36.496693	38.485798	47.847569
H	35.578995	34.804268	49.899158
H	34.355698	39.470322	48.662613
H	33.444656	35.797256	50.721790
H	32.854218	38.137398	50.093941
O	37.336555	33.878475	48.551254

aMOx-**TS-rotation**^{q}

S	38.036873	29.827530	49.166027
C	38.870251	29.326113	50.713818
H	39.360943	28.363853	50.525654
H	39.625584	30.052069	51.012276
N	36.165062	32.127254	50.276329
N	36.573402	31.636469	47.490681
N	39.383217	32.190262	47.801476
C	34.799297	32.477364	52.112701
N	38.951649	32.562336	50.587475
C	34.009026	32.051483	51.098866
C	36.144714	32.531620	51.592892
C	37.245686	32.918392	52.335285
C	34.859192	31.841261	49.941658
C	34.435589	31.449663	48.679203
C	35.225414	31.395325	47.527508
C	34.718021	31.116426	46.194111
C	35.807915	31.250830	45.372551
C	36.959496	31.560108	46.173443
C	38.245766	31.754633	45.681408
C	39.379372	32.014240	46.443272
C	40.734638	32.029003	45.906700
C	41.532192	32.175854	47.009731
C	40.697212	32.276527	48.179058
C	41.151009	32.450829	49.483364
C	40.335728	32.644997	50.586056
C	40.793442	33.034172	51.900330
C	38.549210	32.898689	51.869061
C	39.696342	33.216255	52.678734
H	37.083271	33.228088	53.359058
H	33.375702	31.248243	48.561295
H	38.386318	31.644817	44.612404
H	42.221584	32.476990	49.646786
Fe	37.746407	32.176376	49.036381
C	38.020893	35.041275	49.193455
C	37.467663	36.290241	48.582417
C	36.220959	36.873589	48.926540
C	35.927715	38.205055	48.517101
C	35.244987	36.194614	49.709492
C	34.752838	38.829140	48.908726
C	34.065266	36.824123	50.080318
C	33.813103	38.147278	49.693748
H	39.091408	34.959606	48.986275
H	37.886127	35.069736	50.284485
H	38.088745	36.824703	47.866375
H	36.662792	38.738304	47.917690
H	35.420902	35.162857	49.988731
H	34.554886	39.856533	48.608200
H	33.327724	36.284786	50.667645
H	32.888412	38.643456	49.975128
O	37.356064	33.901646	48.666840

aMOx-**TS2 pro-S**^{d}

S	38.119999	29.905001	49.175999
C	38.909000	29.371000	50.737999
H	39.390999	28.407000	50.535999
H	39.667999	30.083000	51.058998
N	36.241001	32.074001	50.395000
N	36.581001	31.752001	47.567001
N	39.366001	32.342999	47.859001
C	34.882999	32.366001	52.255001
N	38.995998	32.639999	50.674999
C	34.091000	31.933001	51.243000
C	36.221001	32.451000	51.729000
C	37.316002	32.889000	52.452999
C	34.931000	31.756001	50.075001
C	34.493000	31.419001	48.799000
C	35.250000	31.441999	47.622002
C	34.717999	31.171000	46.290001
C	35.785000	31.360001	45.452000
C	36.938999	31.698000	46.242001
C	38.217999	31.919001	45.742001
C	39.358002	32.175999	46.498001
C	40.708000	32.176998	45.950001
C	41.514000	32.305000	47.048000
C	40.687000	32.410000	48.223999
C	41.160000	32.567001	49.527000
C	40.372002	32.743000	50.654999
C	40.854000	33.124001	51.972000
C	38.615002	32.938999	51.963001
C	39.768002	33.276001	52.765999
H	37.157001	33.176998	53.484001
H	33.436001	31.191999	48.695000
H	38.352001	31.819000	44.669998
H	42.234001	32.587002	49.668999
Fe	37.785999	32.161999	49.137001
C	36.092999	34.707001	49.376999
C	36.807999	35.922001	49.740002
C	36.456001	37.257999	49.368000
C	37.301998	38.327000	49.748001
C	35.151001	37.553001	48.896999
C	36.828999	39.632000	49.749001
C	34.675999	38.856998	48.931999
C	35.504002	39.891998	49.377998
H	35.728001	34.141998	50.233002
H	35.257999	34.848000	48.680000
H	37.661999	35.783001	50.393002
H	38.318001	38.102001	50.053001
H	34.500000	36.757000	48.555000
H	37.477001	40.446999	50.058998
H	33.653999	39.076000	48.632999
H	35.105000	40.901001	49.435001
O	37.243999	34.180000	48.759998

aMOx-**TS2 pro-S**^{q}

S	38.095001	29.742001	49.130001
C	38.931000	29.233999	50.681000
H	39.400002	28.254999	50.526001
H	39.700001	29.954000	50.963001
N	36.231998	32.131001	50.376999
N	36.591000	31.825001	47.560001
N	39.401001	32.365002	47.865002
C	34.889000	32.401001	52.247002
N	39.025002	32.598000	50.680000
C	34.084999	32.016998	51.223999
C	36.228001	32.467999	51.720001
C	37.334000	32.855999	52.456001
C	34.917000	31.858999	50.049999
C	34.474998	31.545000	48.769001
C	35.248001	31.551001	47.604000
C	34.730000	31.291000	46.271000
C	35.808998	31.457001	45.441002
C	36.963001	31.768000	46.237999
C	38.248001	31.968000	45.743000
C	39.388000	32.203999	46.502998
C	40.737999	32.193001	45.957001
C	41.547001	32.311001	47.055000
C	40.723999	32.417000	48.231998
C	41.196999	32.555000	49.535000
C	40.402000	32.713001	50.660999
C	40.872002	33.091000	51.978001
C	38.632999	32.895000	51.969002
C	39.780998	33.233002	52.771000
H	37.179001	33.125999	53.492001
H	33.416000	31.339001	48.655998
H	38.382999	31.870001	44.672001
H	42.270000	32.574001	49.681000
Fe	37.805000	32.205002	49.125000
C	36.210999	34.875000	49.522999
C	36.827000	36.188999	49.716999
C	36.314999	37.460999	49.327999
C	37.084000	38.615002	49.623001
C	34.965000	37.630001	48.921001
C	36.502998	39.874001	49.611000
C	34.388000	38.891998	48.930000
C	35.145000	40.011002	49.294998
H	35.932999	34.401001	50.469002
H	35.320999	34.889999	48.877998
H	37.764999	36.174999	50.259998
H	38.132000	38.493000	49.879002
H	34.369999	36.768002	48.643002
H	37.095001	40.748001	49.863998
H	33.341000	39.022999	48.672001
H	34.669998	40.987999	49.325001
O	37.337002	34.313999	48.902000

aMOx-**TS2 pro-R**[d]

S	37.990002	29.936001	49.219002
C	38.888000	29.361000	50.709999
H	39.354000	28.396000	50.477001
H	39.661999	30.066000	51.009998
N	36.254002	32.276001	50.389000
N	36.592999	31.840000	47.615002
N	39.448002	32.266998	47.919998
C	34.926998	32.535999	52.275002
N	39.071999	32.625000	50.716000
C	34.111000	32.146999	51.266998
C	36.259998	32.616001	51.724998
C	37.382000	32.970001	52.462002
C	34.930000	31.982000	50.085999
C	34.485001	31.605000	48.830002
C	35.258999	31.538000	47.662998
C	34.757000	31.146000	46.355000
C	35.848000	31.239000	45.528999
C	36.985001	31.645000	46.312000
C	38.278999	31.809000	45.820999
C	39.426998	32.070999	46.564999
C	40.772999	32.053001	46.007000
C	41.588001	32.196999	47.098000
C	40.766998	32.332001	48.275002
C	41.237000	32.509998	49.578999
C	40.448002	32.710999	50.700001
C	40.931000	33.102001	52.014999
C	38.687000	32.959000	51.990002
C	39.844002	33.277000	52.799999
H	37.229000	33.248001	53.498001
H	33.425999	31.381001	48.734001
H	38.418999	31.642000	44.758999
H	42.310001	32.528000	49.724998
Fe	37.831001	32.237000	49.174999
C	36.417999	34.817001	49.061001
C	36.826000	36.047001	48.359001
C	37.109001	37.313999	48.925999
C	37.459999	38.390999	48.071999
C	37.018002	37.558998	50.319000
C	37.661999	39.662998	48.584999
C	37.243999	38.827999	50.826000
C	37.554001	39.887001	49.963001
H	35.993000	34.992001	50.056999
H	35.694000	34.238998	48.473999
H	37.051998	35.935001	47.306000
H	37.550999	38.209999	47.005001
H	36.798000	36.733002	50.978001
H	37.910000	40.479000	47.914001
H	37.193001	39.008999	51.895000
H	37.717999	40.882000	50.369999
O	37.698002	34.228001	49.105999

aMOx-**TS2 pro-R**[q]

S	38.020000	29.660000	49.159000
C	38.876999	29.173000	50.701000
H	39.363998	28.205000	50.535000
H	39.634998	29.905001	50.980000
N	36.113998	31.985001	50.382999
N	36.456001	31.562000	47.567001
N	39.250999	32.187000	47.824001
C	34.778999	32.316002	52.242001
N	38.886002	32.508999	50.637001
C	33.972000	31.885000	51.240002
C	36.112999	32.374001	51.708000
C	37.220001	32.784000	52.431999
C	34.799000	31.690001	50.069000
C	34.355000	31.316999	48.806000
C	35.118999	31.274000	47.636002
C	34.595001	30.941999	46.320000
C	35.666000	31.083000	45.474998
C	36.820999	31.452999	46.247002
C	38.095001	31.667999	45.728001
C	39.236000	31.971001	46.465000
C	40.583000	31.997000	45.911999
C	41.394001	32.160999	47.005001
C	40.577000	32.269001	48.183998
C	41.055000	32.426998	49.480999
C	40.265999	32.597000	50.606998
C	40.757000	32.952999	51.919998
C	38.513000	32.812000	51.932999
C	39.678001	33.127998	52.722000
H	37.073002	33.076000	53.463001
H	33.296001	31.100000	48.709999
H	38.223000	31.534000	44.660999
H	42.129002	32.450001	49.622002
Fe	37.654999	32.076000	49.111000
C	36.282001	34.743999	49.721001
C	36.129002	35.986000	48.935001
C	37.009998	37.092999	49.014000
C	36.664001	38.316002	48.379002
C	38.216000	37.030998	49.758999
C	37.459999	39.439999	48.539001
C	39.015999	38.152000	49.882999
C	38.637001	39.362999	49.292000
H	36.768002	34.910999	50.688000
H	35.313999	34.262001	49.886002
H	35.304001	36.043999	48.229000
H	35.751999	38.363998	47.790001
H	38.539001	36.091999	50.189999
H	37.181999	40.377998	48.063999
H	39.952000	38.076000	50.419998
H	39.278000	40.234001	49.395000
O	37.105000	34.033001	48.819000

aMOx-**2-S**^{d}

S	38.115002	29.764999	49.139999
C	38.897999	29.246000	50.709000
H	39.351002	28.261999	50.534000
H	39.680000	29.943001	51.007000
N	36.360001	32.272999	50.272999
N	36.716999	31.757999	47.514999
N	39.504002	32.221001	47.832001
C	35.004002	32.567001	52.146999
N	39.133999	32.549000	50.624001
C	34.196999	32.194000	51.123001
C	36.344002	32.604000	51.623001
C	37.455002	32.909000	52.386002
C	35.032001	31.997999	49.959000
C	34.588001	31.611000	48.706001
C	35.365002	31.528000	47.549000
C	34.841000	31.237000	46.222000
C	35.928001	31.337999	45.395000
C	37.084000	31.639999	46.192001
C	38.372002	31.806999	45.701000
C	39.505001	32.054001	46.464001
C	40.851002	32.062000	45.919998
C	41.657001	32.185001	47.019001
C	40.833000	32.284000	48.195000
C	41.305000	32.435001	49.495998
C	40.516998	32.624001	50.617001
C	40.997002	32.998001	51.932999
C	38.756001	32.868999	51.916000
C	39.911999	33.172001	52.723000
H	37.299999	33.179001	53.423000
H	33.523998	31.417999	48.603001
H	38.513000	31.688000	44.632000
H	42.377998	32.450001	49.640999
Fe	37.945999	31.992001	49.108002
C	37.744999	35.776001	50.410000
C	37.637001	37.099998	49.777000
C	36.370998	37.875999	49.768002
C	36.417000	39.258999	49.978001
C	35.129002	37.247002	49.613998
C	35.237999	40.000999	50.060001
C	33.956001	37.987999	49.712002
C	34.004002	39.366001	49.931999
H	38.688999	35.448002	50.830002
H	36.855999	35.324001	50.841000
H	38.541000	37.709999	49.721001
H	37.381001	39.756001	50.075001
H	35.085999	36.182999	49.404999
H	35.278999	41.074001	50.223999
H	32.999001	37.491001	49.620998
H	33.088001	39.943001	49.981998
O	37.705002	35.889999	48.980000

aMOx-**2-S**^{q}

S	38.125999	29.540001	49.124001
C	38.936001	29.063999	50.698002
H	39.387001	28.072001	50.582001
H	39.715000	29.777000	50.965000
N	36.362999	32.221001	50.292000
N	36.728001	31.757000	47.523998
N	39.530998	32.191002	47.838001
C	35.013000	32.515999	52.166000
N	39.148998	32.507999	50.627998
C	34.201000	32.174999	51.133999
C	36.355000	32.535000	51.644001
C	37.467999	32.833000	52.405998
C	35.034000	31.985001	49.969002
C	34.584999	31.628000	48.707001
C	35.368999	31.547001	47.556000
C	34.853001	31.259001	46.230000
C	35.945999	31.340000	45.407001
C	37.103001	31.629999	46.202000
C	38.391998	31.781000	45.709000
C	39.525002	32.029999	46.472000
C	40.869999	32.051998	45.924000
C	41.679001	32.192001	47.019001
C	40.858002	32.278999	48.196999
C	41.330002	32.435001	49.495998
C	40.534000	32.605999	50.616001
C	41.006001	32.983002	51.930000
C	38.765999	32.814999	51.924999
C	39.917999	33.133999	52.724998
H	37.317001	33.092999	53.445000
H	33.520000	31.451000	48.597000
H	38.532001	31.659000	44.641998
H	42.402000	32.464001	49.640999
Fe	37.959000	31.931000	49.109001
C	37.028999	35.523998	50.195999
C	37.165001	36.924999	49.777000
C	36.062000	37.915001	49.808998
C	36.394001	39.256001	50.025002
C	34.714001	37.553001	49.674000
C	35.396999	40.221001	50.131001
C	33.719002	38.518002	49.803001
C	34.058998	39.853001	50.029999
H	37.894001	35.009998	50.601002
H	36.060001	35.153999	50.518002
H	38.153000	37.376999	49.865002
H	37.441002	39.543999	50.102001
H	34.446999	36.523998	49.457001
H	35.657001	41.261002	50.299000
H	32.672001	38.247002	49.733002
H	33.289001	40.606998	50.112999
O	37.099998	35.849998	48.801998

aMOx-**2-R**^{d}

S	38.125000	29.778000	49.152000
C	38.907001	29.280001	50.727001
H	39.369999	28.299999	50.558998
H	39.680000	29.987000	51.022999
N	36.308998	32.216999	50.285000
N	36.673000	31.714001	47.523998
N	39.458000	32.222000	47.834000
C	34.950001	32.515999	52.153000
N	39.077999	32.589001	50.617001
C	34.151001	32.104000	51.139000
C	36.286999	32.587002	51.622002
C	37.390999	32.958000	52.367001
C	34.990002	31.906000	49.977001
C	34.550999	31.504000	48.724998
C	35.328999	31.436001	47.564999
C	34.814999	31.099001	46.247002
C	35.900002	31.207001	45.417000
C	37.046001	31.569000	46.205002
C	38.332001	31.754000	45.710999
C	39.462002	32.030998	46.469002
C	40.810001	32.035000	45.928001
C	41.612999	32.175999	47.028999
C	40.785000	32.291000	48.200001
C	41.254002	32.459999	49.500999
C	40.459000	32.667000	50.613998
C	40.933998	33.067001	51.925999
C	38.692001	32.938000	51.896000
C	39.844002	33.257999	52.705002
H	37.231998	33.250999	53.396000
H	33.493000	31.284000	48.625999
H	38.477001	31.610001	44.646000
H	42.326000	32.478001	49.650002
Fe	37.897999	32.002998	49.112000
C	35.728001	35.272999	49.188000
C	36.722000	36.174000	48.583000
C	36.997002	37.541000	49.101002
C	37.125999	38.612000	48.213001
C	37.132000	37.765999	50.473000
C	37.355000	39.900002	48.695000
C	37.337002	39.054001	50.956001
C	37.449001	40.125999	50.068001
H	35.176998	35.613998	50.063000
H	35.230000	34.522999	48.580002
H	36.946999	36.042000	47.526001
H	37.062000	38.433998	47.143002
H	37.103001	36.915001	51.139000
H	37.474998	40.722000	47.993999
H	37.424999	39.230000	52.025002
H	37.618000	41.129002	50.452999
O	37.129002	35.055000	49.410999

aMOx-**2-R**^{q}

S	38.104000	29.500000	49.118000
C	38.918999	29.068001	50.700001
H	39.379002	28.077999	50.605999
H	39.689999	29.795000	50.953999
N	36.241001	32.182999	50.212002
N	36.653999	31.614000	47.470001
N	39.446999	32.111000	47.803001
C	34.872002	32.499001	52.066002
N	39.021000	32.528999	50.564999
C	34.077000	32.098000	51.042999
C	36.214001	32.551998	51.546001
C	37.313999	32.904999	52.304001
C	34.924000	31.891001	49.888000
C	34.500000	31.472000	48.636002
C	35.300999	31.358999	47.497002
C	34.813999	30.982000	46.183998
C	35.919998	31.044001	45.372002
C	37.055000	31.417000	46.162998
C	38.346001	31.591000	45.679001
C	39.462002	31.906000	46.443001
C	40.813999	31.945000	45.910999
C	41.604000	32.124001	47.014999
C	40.764999	32.228001	48.178001
C	41.219002	32.419998	49.479000
C	40.404999	32.619999	50.578999
C	40.855999	33.019001	51.893002
C	38.618000	32.875000	51.845001
C	39.757000	33.198002	52.661999
H	37.148998	33.198002	53.333000
H	33.443001	31.252001	48.523998
H	38.505001	31.426001	44.619999
H	42.290001	32.453999	49.638000
Fe	37.862999	31.884001	49.063000
C	35.515999	35.426998	49.224998
C	36.358002	36.424000	48.561001
C	36.548000	37.820000	49.049999
C	35.512001	38.542999	49.648998
C	37.803001	38.423000	48.880001
C	35.737000	39.841999	50.106998
C	38.035999	39.708000	49.360001
C	37.005001	40.411999	49.983002
H	34.980000	35.693001	50.133999
H	35.077999	34.616001	48.650002
H	36.562000	36.293999	47.498001
H	34.523998	38.099998	49.743000
H	38.598999	37.887001	48.375000
H	34.924000	40.407001	50.556000
H	39.015999	40.160999	49.237999
H	37.194000	41.409000	50.368999
O	36.945999	35.365002	49.376999

aMOx-**Int2**^{d}

S	38.043999	29.962999	49.243000
C	38.897999	29.344999	50.743000
H	39.342999	28.371000	50.506001
H	39.686001	30.028000	51.056000
N	36.376999	32.317001	50.459000
N	36.722000	31.927000	47.645000
N	39.563999	32.257000	47.963001
C	35.042000	32.603001	52.334000
N	39.200001	32.550999	50.794998
C	34.224998	32.284000	51.303001
C	36.384998	32.629002	51.801998
C	37.511002	32.919998	52.555000
C	35.057999	32.108002	50.126999
C	34.599998	31.788000	48.854000
C	35.374001	31.698000	47.694000
C	34.851002	31.344000	46.377998
C	35.941002	31.391001	45.553001
C	37.099998	31.733000	46.341999
C	38.396999	31.846001	45.848000
C	39.548000	32.070000	46.604000
C	40.897999	32.063000	46.051998
C	41.708000	32.205002	47.146000
C	40.886002	32.319000	48.327999
C	41.355000	32.472000	49.631001
C	40.570000	32.631001	50.769001
C	41.063000	32.993999	52.089001
C	38.817001	32.875000	52.083000
C	39.984001	33.167000	52.887001
H	37.362999	33.187000	53.592999
H	33.532001	31.620001	48.748001
H	38.534000	31.688999	44.784000
H	42.426998	32.502998	49.773998
Fe	37.958000	32.247002	49.236000
C	38.527000	35.112000	49.775002
C	38.122002	36.492001	49.948002
C	36.852001	37.085999	49.743999
C	36.682999	38.470001	50.029999
C	35.729000	36.331001	49.306999
C	35.443001	39.070000	49.894001
C	34.484001	36.939999	49.209999
C	34.337002	38.301998	49.494999
H	39.608002	35.098000	49.512001
H	38.629002	34.855000	50.882999
H	38.904999	37.131001	50.354000
H	37.542999	39.055000	50.347000
H	35.873001	35.283001	49.071999
H	35.319000	40.131001	50.098999
H	33.632000	36.358002	48.887001
H	33.362999	38.778000	49.397999
O	37.759998	34.241001	49.084999

aMOx-**Int2**^{q}

S	38.067001	29.702999	49.168999
C	38.923000	29.159000	50.699001
H	39.368999	28.169001	50.544998
H	39.710999	29.862000	50.971001
N	36.347000	32.300999	50.400002
N	36.707001	31.900000	47.598999
N	39.556000	32.236000	47.905998
C	35.019001	32.588001	52.279999
N	39.181000	32.528999	50.731998
C	34.198002	32.268002	51.251999
C	36.361000	32.610001	51.743999
C	37.487999	32.901001	52.493000
C	35.026001	32.092999	50.074001
C	34.570999	31.773001	48.799999
C	35.353001	31.681000	47.646000
C	34.838001	31.332001	46.331001
C	35.933998	31.372000	45.507000
C	37.091000	31.704000	46.292999
C	38.388000	31.813000	45.796001
C	39.539001	32.040001	46.547001
C	40.889000	32.032001	45.997002
C	41.699001	32.180000	47.091000
C	40.879002	32.299000	48.271000
C	41.347000	32.453999	49.574001
C	40.556000	32.616001	50.706001
C	41.040001	32.985001	52.021000
C	38.793999	32.855999	52.016998
C	39.957001	33.153000	52.819000
H	37.344002	33.166000	53.532001
H	33.504002	31.606001	48.688999
H	38.521999	31.653999	44.731998
H	42.417999	32.484001	49.719002
Fe	37.939999	32.207001	49.174000
C	38.486000	35.305000	49.787998
C	38.112000	36.692001	49.998001
C	36.859001	37.313000	49.770000
C	36.692001	38.693001	50.075001
C	35.743999	36.576000	49.285000
C	35.460999	39.304001	49.915001
C	34.507000	37.198002	49.160000
C	34.362000	38.554001	49.466000
H	39.564999	35.314999	49.500999
H	38.641998	35.034000	50.890999
H	38.897999	37.299999	50.445000
H	37.546001	39.264999	50.430000
H	35.891998	35.529999	49.041000
H	35.338001	40.362000	50.136002
H	33.660000	36.627998	48.801998
H	33.394001	39.037998	49.351002
O	37.719002	34.438999	49.124001

aMOx-**TS3-cis-re**[d]

S	38.061001	29.948999	49.231998
C	38.901001	29.343000	50.743000
H	39.348999	28.368000	50.513000
H	39.687000	30.027000	51.056999
N	36.374001	32.297001	50.438000
N	36.722000	31.898001	47.629002
N	39.558998	32.255001	47.946999
C	35.035999	32.591999	52.310001
N	39.193001	32.548000	50.776001
C	34.222000	32.249001	51.284000
C	36.377998	32.629002	51.777000
C	37.500999	32.944000	52.522999
C	35.056000	32.069000	50.110001
C	34.601002	31.738001	48.838001
C	35.375000	31.660000	47.676998
C	34.854000	31.313000	46.359001
C	35.943001	31.375999	45.533001
C	37.099998	31.719000	46.323002
C	38.396000	31.846001	45.828999
C	39.544998	32.070999	46.585999
C	40.894001	32.064999	46.035000
C	41.702999	32.201000	47.132000
C	40.881001	32.312000	48.313000
C	41.352001	32.458000	49.618000
C	40.567001	32.622002	50.751999
C	41.056999	32.991001	52.070999
C	38.810001	32.887001	52.054001
C	39.976002	33.178001	52.862999
H	37.351002	33.224998	53.556999
H	33.535000	31.561001	48.731998
H	38.533001	31.697001	44.764000
H	42.424000	32.487000	49.759998
Fe	37.955002	32.222000	49.219002
C	38.480999	35.111000	49.755001
C	38.103001	36.486000	49.963001
C	36.826000	37.098999	49.792000
C	36.683998	38.477001	50.101002
C	35.696999	36.380001	49.323002
C	35.463001	39.112999	49.943001
C	34.470001	37.021999	49.207001
C	34.348000	38.382000	49.507999
H	39.575001	34.985001	49.683998
H	38.366001	35.058998	50.936001
H	38.895000	37.096001	50.394001
H	37.549999	39.036999	50.446999
H	35.813000	35.332001	49.077999
H	35.362999	40.174000	50.160999
H	33.612000	36.464001	48.858002
H	33.387001	38.882000	49.396999
O	37.723999	34.245998	49.090000

aMOx-**TS3-cis-re**[q]

S	38.074001	29.705999	49.162998
C	38.924999	29.167999	50.699001
H	39.373001	28.179001	50.546001
H	39.710999	29.872999	50.972000
N	36.334000	32.297001	50.393002
N	36.699001	31.899000	47.595001
N	39.541000	32.264999	47.905998
C	35.001999	32.582001	52.269001
N	39.160999	32.547001	50.726002
C	34.185001	32.241001	51.243999
C	36.342999	32.619999	51.733002
C	37.466999	32.930000	52.480000
C	35.014000	32.070000	50.067001
C	34.563999	31.745001	48.794998
C	35.346001	31.670000	47.639000
C	34.832001	31.340000	46.320000
C	35.925999	31.405001	45.494999
C	37.082001	31.730000	46.285000
C	38.376999	31.858999	45.789001
C	39.526001	32.082001	46.542999
C	40.875999	32.071999	45.995998
C	41.685001	32.205002	47.094002
C	40.863998	32.317001	48.272999
C	41.332001	32.457001	49.577999
C	40.540001	32.620998	50.706001
C	41.020000	32.981998	52.023998
C	38.772999	32.876999	52.007999
C	39.935001	33.165001	52.814999
H	37.319000	33.201000	53.516998
H	33.499001	31.568001	48.681999
H	38.512001	31.715000	44.723000
H	42.403999	32.477001	49.726002
Fe	37.928001	32.191002	49.173000
C	38.426998	35.341999	49.763000
C	38.083000	36.726002	49.997002
C	36.808998	37.359001	49.831001
C	36.675999	38.733002	50.150002
C	35.681999	36.658001	49.334999
C	35.463001	39.383999	49.976002
C	34.464001	37.313999	49.199001
C	34.348999	38.671001	49.512001
H	39.516998	35.174999	49.722000
H	38.290001	35.347000	50.955002
H	38.888000	37.313999	50.437000
H	37.540001	39.283001	50.514000
H	35.794998	35.610001	49.084999
H	35.370998	40.444000	50.202999
H	33.608002	36.766998	48.827999
H	33.395000	39.180000	49.389999
O	37.647999	34.478001	49.138000

aMOx-**TS3-trans-si**^{d}

S	38.073002	29.952000	49.242001
C	38.905998	29.340000	50.752998
H	39.348999	28.363001	50.521999
H	39.695000	30.020000	51.070000
N	36.408001	32.324001	50.445000
N	36.754002	31.907000	47.633999
N	39.592999	32.234001	47.955002
C	35.070999	32.612999	52.319000
N	39.224998	32.535000	50.791000
C	34.252998	32.301998	51.285000
C	36.415001	32.631001	51.789001
C	37.542000	32.902000	52.549000
C	35.087002	32.117001	50.112000
C	34.630001	31.787001	48.841999
C	35.404999	31.683001	47.683998
C	34.882999	31.316999	46.372002
C	35.976002	31.351000	45.547001
C	37.132999	31.697001	46.332001
C	38.431999	31.802999	45.839001
C	39.580002	32.035999	46.595001
C	40.929001	32.035999	46.046001
C	41.737000	32.191002	47.140999
C	40.914001	32.306999	48.320999
C	41.382999	32.465000	49.625999
C	40.602001	32.616001	50.763000
C	41.091999	32.979000	52.083000
C	38.846001	32.841999	52.081001
C	40.011002	33.133999	52.883999
H	37.393002	33.164001	53.588001
H	33.562000	31.618999	48.737000
H	38.570000	31.636000	44.777000
H	42.455002	32.505001	49.764999
Fe	37.990002	32.222000	49.227001
C	38.537998	35.155998	49.723999
C	38.085999	36.481998	50.075001
C	36.848999	37.117001	49.758999
C	36.696999	38.502998	50.027000
C	35.731998	36.375000	49.299000
C	35.467999	39.119999	49.859001
C	34.500000	37.001999	49.158001
C	34.363998	38.367001	49.431000
H	39.273998	35.770000	49.029999
H	39.285000	34.765999	50.430000
H	38.848999	37.088001	50.562000
H	37.555000	39.076000	50.367001
H	35.862000	35.323002	49.077000
H	35.355000	40.182999	50.056999
H	33.647999	36.429001	48.817001
H	33.398998	38.855000	49.307999
O	37.793999	34.277000	49.071999

aMOx-**TS3-trans-si**^{q}

S	38.069000	29.701000	49.167999
C	38.923000	29.155001	50.699001
H	39.366001	28.164000	50.542999
H	39.712002	29.856001	50.971001
N	36.352001	32.308998	50.405998
N	36.713001	31.907000	47.606998
N	39.558998	32.238998	47.917999
C	35.021999	32.591000	52.285000
N	39.181000	32.518002	50.745998
C	34.201000	32.278999	51.254002
C	36.365002	32.608002	51.751999
C	37.492001	32.879002	52.507999
C	35.028999	32.105000	50.077000
C	34.574001	31.787001	48.804001
C	35.358002	31.691000	47.651001
C	34.844002	31.343000	46.337002
C	35.941002	31.381001	45.514000
C	37.098000	31.709999	46.299999
C	38.394001	31.819000	45.805000
C	39.543999	32.046001	46.557999
C	40.893002	32.041000	46.008999
C	41.702000	32.188000	47.104000
C	40.882000	32.303001	48.284000
C	41.348999	32.453999	49.588001
C	40.558998	32.605999	50.720001
C	41.041000	32.967999	52.035999
C	38.796001	32.826000	52.035999
C	39.958000	33.123001	52.837002
H	37.346001	33.137001	53.549000
H	33.507000	31.622000	48.692001
H	38.528999	31.660000	44.741001
H	42.419998	32.486000	49.733002
Fe	37.945000	32.193001	49.186001
C	38.480000	35.348000	49.801998
C	38.056999	36.691002	50.146000
C	36.840000	37.346001	49.810001
C	36.681000	38.727001	50.102001
C	35.735001	36.618000	49.296001
C	35.459999	39.349998	49.908001
C	34.508999	37.250999	49.130001
C	34.367001	38.609001	49.430000
H	39.328999	35.841000	49.168999
H	39.132000	34.938000	50.599998
H	38.819000	37.277000	50.658001
H	37.528999	39.292000	50.481998
H	35.877998	35.570999	49.056000
H	35.341000	40.409000	50.125000
H	33.667000	36.688000	48.750999
H	33.407001	39.101002	49.289001
O	37.737999	34.484001	49.134998

aMOx-**3**^{d}

S	38.098000	29.900999	49.195000
C	38.896000	29.322001	50.735001
H	39.341999	28.344000	50.519001
H	39.681999	30.004999	51.051998
N	36.348000	32.258999	50.417999
N	36.682999	31.851000	47.618999
N	39.492001	32.290001	47.914001
C	35.009998	32.561001	52.292000
N	39.144001	32.549000	50.738998
C	34.195000	32.203999	51.270000
C	36.348999	32.595001	51.758999
C	37.470001	32.910999	52.507000
C	35.025002	32.012001	50.098999
C	34.570000	31.659000	48.835999
C	35.338001	31.596001	47.671001
C	34.808998	31.271999	46.353001
C	35.889000	31.372000	45.515999
C	37.048000	31.709999	46.299999
C	38.333000	31.871000	45.794998
C	39.480999	32.108002	46.547001
C	40.826000	32.105999	45.995998
C	41.638000	32.234001	47.092999
C	40.821999	32.338001	48.273998
C	41.300999	32.474998	49.575001
C	40.521999	32.636002	50.710999
C	41.014999	33.012001	52.022999
C	38.771000	32.869999	52.028999
C	39.937000	33.178001	52.825001
H	37.321999	33.187000	53.542999
H	33.505001	31.472000	48.736000
H	38.465000	31.740999	44.726002
H	42.374001	32.500999	49.710999
Fe	37.915001	32.130001	49.203999
C	38.279999	35.265999	49.771999
C	37.995998	36.719002	50.022999
C	36.619999	37.299000	49.773998
C	36.519001	38.647999	49.404999
C	35.439999	36.576000	50.000000
C	35.273998	39.269001	49.298000
C	34.195999	37.198002	49.889000
C	34.105000	38.548000	49.546001
H	39.273998	34.932999	50.095001
H	38.316002	36.896000	51.062000
H	38.758999	37.257000	49.433998
H	37.423000	39.219002	49.201000
H	35.486000	35.527000	50.263000
H	35.216000	40.319000	49.020000
H	33.292999	36.626999	50.077000
H	33.136002	39.035000	49.464001
O	37.529999	34.464001	49.222000

aMOx-**3**^{q}

S	38.094002	29.641001	49.119999
C	38.912998	29.157000	50.688000
H	39.375999	28.173000	50.556999
H	39.686001	29.877001	50.959999
N	36.342999	32.338001	50.235001
N	36.720001	31.851000	47.471001
N	39.525002	32.265999	47.785999
C	34.995998	32.597000	52.115002
N	39.131001	32.598000	50.577999
C	34.188000	32.241001	51.087002
C	36.334999	32.657001	51.583000
C	37.445999	32.969002	52.340000
C	35.020000	32.076000	49.916000
C	34.574001	31.718000	48.653000
C	35.360001	31.639000	47.502998
C	34.847000	31.337999	46.180000
C	35.942001	31.409000	45.356998
C	37.098999	31.705000	46.150002
C	38.389999	31.837000	45.657001
C	39.528000	32.074001	46.418999
C	40.876999	32.069000	45.883999
C	41.680000	32.210999	46.987000
C	40.853001	32.326000	48.157001
C	41.316002	32.480999	49.459999
C	40.514999	32.669998	50.571999
C	40.988998	33.037998	51.887001
C	38.745998	32.929001	51.865002
C	39.901001	33.220001	52.674000
H	37.293999	33.237000	53.377998
H	33.511002	31.532000	48.542000
H	38.529999	31.702000	44.591000
H	42.387001	32.495998	49.613998
Fe	37.941002	32.035999	49.066002
C	38.445999	36.492001	49.591999
C	37.951000	37.782001	50.209999
C	36.494999	38.158001	50.005001
C	36.165001	39.509998	49.841999
C	35.462002	37.209999	50.001999
C	34.837002	39.910000	49.691002
C	34.137001	37.610001	49.834999
C	33.818001	38.958000	49.680000
H	39.498001	36.507000	49.254002
H	38.175999	37.675999	51.283001
H	38.599998	38.592999	49.853001
H	36.957001	40.257999	49.831001
H	35.699001	36.160999	50.112999
H	34.595001	40.964001	49.570000
H	33.352001	36.861000	49.819000
H	32.789001	39.266998	49.537998
O	37.783001	35.473999	49.483002

Table S16. Cartesian coordinates (xyz, in Å) of the QM atoms of all QM/MM optimized stationary points reported in **Figure S23** and **Table S8**.

aMOx-**1**^{d}

S	43.490002	26.382000	45.424000
C	45.077000	25.757999	46.101002
H	45.776001	25.563999	45.278999
H	45.521000	26.493000	46.771000
N	41.655998	25.926001	48.097000
N	40.735001	27.379000	45.799000
N	42.868999	29.278999	46.181000
C	41.341999	24.334999	49.757999
N	43.800999	27.760000	48.424999
C	40.356998	24.153000	48.848000
C	42.175999	25.402000	49.250999
C	43.376999	25.802000	49.837002
C	40.532001	25.165001	47.823002
C	39.696999	25.375000	46.740002
C	39.778999	26.409000	45.787998
C	38.875000	26.563000	44.659000
C	39.340000	27.670000	43.997002
C	40.490002	28.172001	44.702000
C	41.256001	29.278999	44.344002
C	42.354000	29.804001	45.027000
C	43.112999	30.959999	44.584000
C	44.097000	31.094999	45.527000
C	43.937000	30.059999	46.511002
C	44.755001	29.870001	47.633999
C	44.685001	28.816000	48.528000
C	45.549000	28.632000	49.674999
C	44.112999	26.922001	49.466999
C	45.235001	27.436001	50.222000
H	43.750000	25.212999	50.665001
H	38.874001	24.677999	46.620998
H	40.980000	29.792000	43.430000
H	45.542999	30.594999	47.799999
Fe	42.202999	27.643999	47.192001
C	34.708000	28.843000	51.984001
C	35.619999	29.818001	52.088001
C	36.873001	29.966000	51.330002
C	37.699001	31.075001	51.589001
C	37.297001	29.044001	50.354000
C	38.919998	31.238001	50.935001
C	38.519001	29.198000	49.705002
C	39.342999	30.290001	50.001999
H	34.812000	28.000999	51.305000
H	33.813999	28.858999	52.598000
H	35.425999	30.613001	52.807999
H	37.376999	31.813000	52.321999
H	36.661999	28.200001	50.104000
H	39.537998	32.104000	51.162998
H	38.844002	28.466999	48.972000
H	40.308998	30.379000	49.514999
O	41.265999	28.552000	48.168999

aMOx-**1**^{q}

S	43.445999	26.336000	45.437000
C	45.028000	25.709000	46.118999
H	45.734001	25.531000	45.299999
H	45.465000	26.438999	46.799999
N	41.627998	25.995001	48.069000
N	40.737999	27.433001	45.754002
N	42.893002	29.309000	46.130001
C	41.321999	24.388000	49.715000
N	43.794998	27.805000	48.393002
C	40.332001	24.216999	48.807999
C	42.153999	25.459000	49.216000
C	43.358002	25.848000	49.799000
C	40.502998	25.237000	47.791000
C	39.669998	25.450001	46.706001
C	39.770000	26.474001	45.747002
C	38.879002	26.625999	44.606998
C	39.365002	27.719000	43.936001
C	40.514000	28.214001	44.645000
C	41.294998	29.309000	44.280998
C	42.388000	29.832001	44.970001
C	43.150002	30.988001	44.533001
C	44.123001	31.127001	45.487000
C	43.955002	30.094000	46.471001
C	44.761002	29.910000	47.604000
C	44.681999	28.858999	48.500000
C	45.542000	28.669001	49.651001
C	44.101002	26.965000	49.432999
C	45.224998	27.469999	50.191002
H	43.729000	25.257000	50.625999
H	38.841999	24.760000	46.588001
H	41.029999	29.816000	43.359001
H	45.544998	30.636000	47.777000
Fe	42.200001	27.693001	47.150002
C	34.719002	28.844000	51.974998
C	35.631001	29.818001	52.087002
C	36.884998	29.974001	51.332001
C	37.707001	31.083000	51.598999
C	37.311001	29.058001	50.352001
C	38.929001	31.252001	50.948002
C	38.533001	29.218000	49.705002
C	39.355000	30.311001	50.009998
H	34.823002	28.007999	51.289001
H	33.824001	28.855000	52.587002
H	35.435001	30.608000	52.813999
H	37.382000	31.816000	52.334999
H	36.678001	28.214001	50.096001
H	39.543999	32.118000	51.181999
H	38.861000	28.493999	48.966999
H	40.320999	30.405001	49.522999
O	41.276001	28.631001	48.112000

aMOx-**TS1**[d]

S	43.455002	26.298000	45.442001
C	45.035999	25.690001	46.139000
H	45.745998	25.531000	45.319000
H	45.458000	26.420000	46.828999
N	41.618999	25.797001	48.078999
N	40.710999	27.195000	45.737999
N	42.785999	29.153000	46.151001
C	41.341000	24.188999	49.726002
N	43.691002	27.680000	48.431999
C	40.355000	24.003000	48.813999
C	42.155998	25.274000	49.236000
C	43.333000	25.698000	49.837002
C	40.514999	25.016001	47.792000
C	39.676998	25.200001	46.701000
C	39.747002	26.226000	45.754002
C	38.814999	26.398001	44.648998
C	39.270000	27.504999	43.981998
C	40.442001	28.002001	44.660000
C	41.183998	29.125000	44.299999
C	42.259998	29.670000	44.995998
C	42.986000	30.858999	44.574001
C	43.958000	31.016001	45.523998
C	43.827999	29.965000	46.498001
C	44.644001	29.796000	47.618999
C	44.574001	28.746000	48.516998
C	45.445000	28.570000	49.658001
C	44.035000	26.834999	49.469002
C	45.154999	27.367001	50.208000
H	43.716999	25.121000	50.667999
H	38.865002	24.490999	46.588001
H	40.897999	29.635000	43.388000
H	45.431999	30.521999	47.779999
Fe	42.146999	27.489000	47.157001
C	40.661999	28.569000	49.974998
C	39.723000	29.568001	50.061001
C	40.012001	30.975000	49.874001
C	39.105000	31.962999	50.318001
C	41.195999	31.398001	49.220001
C	39.383999	33.314999	50.146999
C	41.453999	32.750000	49.029999
C	40.556999	33.713001	49.499001
H	41.716000	28.783001	50.098000
H	40.362999	27.538000	50.120998
H	38.678001	29.301001	50.216999
H	38.188000	31.658001	50.819000
H	41.881001	30.656000	48.831001
H	38.683998	34.061001	50.515999
H	42.352001	33.063999	48.505001
H	40.775002	34.759998	49.334999
O	41.043999	28.434999	47.973999

aMOx-**TS1**[q]

S	43.327999	26.358999	45.568001
C	44.925999	25.764000	46.230000
H	45.626999	25.646000	45.395000
H	45.341999	26.481001	46.938000
N	41.519001	26.025000	48.102001
N	40.652000	27.448999	45.768002
N	42.757000	29.349001	46.166000
C	41.276001	24.368000	49.708000
N	43.667000	27.865999	48.432999
C	40.275002	24.205000	48.808998
C	42.078999	25.469000	49.230000
C	43.271000	25.875000	49.817001
C	40.417000	25.250999	47.814999
C	39.577999	25.461000	46.727001
C	39.680000	26.479000	45.773998
C	38.790001	26.627001	44.633999
C	39.275002	27.712000	43.951000
C	40.424000	28.219999	44.653000
C	41.193001	29.320999	44.283001
C	42.268002	29.855000	44.986000
C	43.037998	31.007999	44.549000
C	44.007000	31.152000	45.505001
C	43.834000	30.132000	46.504002
C	44.644001	29.957001	47.625000
C	44.560001	28.910000	48.529999
C	45.435001	28.711000	49.667000
C	43.987999	27.009001	49.464001
C	45.118999	27.511000	50.209999
H	43.662998	25.275999	50.627998
H	38.757999	24.761999	46.604000
H	40.933998	29.816999	43.355000
H	45.448002	30.667999	47.778000
Fe	42.088001	27.738001	47.152000
C	40.508999	28.551001	49.980999
C	39.236000	29.066000	50.152000
C	38.928001	30.466999	50.366001
C	37.745998	30.834000	51.048000
C	39.787998	31.502001	49.931000
C	37.464001	32.169998	51.321999
C	39.491001	32.837002	50.186001
C	38.332001	33.179001	50.889999
H	41.374001	29.138000	50.266998
H	40.637001	27.478001	50.009998
H	38.389999	28.379999	50.143002
H	37.074001	30.055000	51.404999
H	40.678001	31.249001	49.362000
H	36.574001	32.430000	51.894001
H	40.160000	33.615002	49.828999
H	38.105000	34.220001	51.101002
O	41.081001	28.708000	48.108002

aMOx-**Int1**^{d}

S	43.484001	26.322001	45.464001
C	45.069000	25.694000	46.138000
H	45.766998	25.521999	45.311001
H	45.507000	26.421000	46.820999
N	41.657001	25.754999	48.099998
N	40.688000	27.195999	45.783001
N	42.752998	29.148001	46.181999
C	41.366001	24.159000	49.762001
N	43.709999	27.655001	48.459999
C	40.375999	23.973000	48.855999
C	42.187000	25.233000	49.252998
C	43.375000	25.664000	49.848000
C	40.540001	24.983000	47.827000
C	39.692001	25.184000	46.749001
C	39.741001	26.221001	45.799000
C	38.793999	26.378000	44.702000
C	39.229000	27.493000	44.033001
C	40.397999	28.000999	44.708000
C	41.133999	29.129999	44.354000
C	42.223999	29.674999	45.037998
C	42.955002	30.857000	44.605999
C	43.941002	31.002001	45.546001
C	43.808998	29.947001	46.515999
C	44.637001	29.767000	47.632999
C	44.583000	28.722000	48.542000
C	45.462002	28.561001	49.683998
C	44.067001	26.813999	49.493000
C	45.181000	27.360001	50.237999
H	43.766998	25.080000	50.672001
H	38.877998	24.475000	46.641998
H	40.837002	29.648001	43.449001
H	45.426998	30.495001	47.783001
Fe	42.186001	27.438000	47.140999
C	40.825001	28.334000	49.534000
C	39.862999	29.382999	50.000999
C	40.061001	30.775000	49.834999
C	39.159000	31.712999	50.415001
C	41.187000	31.291000	49.123001
C	39.382000	33.077999	50.312000
C	41.387001	32.658001	49.014000
C	40.493999	33.557999	49.609001
H	41.793999	28.483000	50.044998
H	40.450001	27.346001	49.835999
H	38.953999	29.068001	50.513000
H	38.294998	31.344000	50.965000
H	41.868000	30.596001	48.651001
H	38.692001	33.778000	50.777000
H	42.238998	33.041000	48.459999
H	40.673000	34.620998	49.512001
O	40.973999	28.429001	48.138000

aMOx-**Int1**^{q}

S	43.424999	26.301001	45.518002
C	45.018002	25.702000	46.188000
H	45.716999	25.568001	45.355000
H	45.437000	26.422001	46.889999
N	41.620998	25.799999	48.112999
N	40.706001	27.202999	45.786999
N	42.769001	29.164000	46.198002
C	41.351002	24.193001	49.757999
N	43.719002	27.658001	48.433998
C	40.362999	24.006001	48.847000
C	42.167999	25.273001	49.269001
C	43.348999	25.687000	49.862999
C	40.515999	25.016001	47.825001
C	39.679001	25.190001	46.734001
C	39.749001	26.222000	45.798000
C	38.819000	26.393999	44.695000
C	39.264000	27.510000	44.035000
C	40.428001	28.018000	44.714001
C	41.150002	29.152000	44.358002
C	42.233002	29.684999	45.049000
C	42.964001	30.868999	44.623001
C	43.950001	31.017000	45.561001
C	43.828999	29.964001	46.533001
C	44.660999	29.790001	47.636002
C	44.592999	28.732000	48.523998
C	45.458000	28.556999	49.666000
C	44.051998	26.815001	49.484001
C	45.167000	27.354000	50.219002
H	43.731998	25.112000	50.695000
H	38.870998	24.476000	46.618000
H	40.855999	29.669001	43.452999
H	45.451000	30.513000	47.792999
Fe	42.174999	27.464001	47.169998
C	40.799999	28.372999	49.556000
C	39.835999	29.448999	49.950001
C	40.077000	30.834999	49.772999
C	39.155998	31.799999	50.276001
C	41.247002	31.323000	49.115002
C	39.396000	33.159000	50.141998
C	41.465000	32.686001	48.973999
C	40.549000	33.611000	49.487000
H	41.749001	28.483999	50.106998
H	40.384998	27.389000	49.804001
H	38.890999	29.153999	50.402000
H	38.257999	31.455000	50.785999
H	41.957001	30.618000	48.706001
H	38.685001	33.875999	50.547001
H	42.351002	33.042000	48.457001
H	40.738998	34.668999	49.359001
O	41.055000	28.468000	48.166000

aMOx-**TS2**^{d}

S	43.500999	26.295000	45.451000
C	45.071999	25.646999	46.132999
H	45.766998	25.472000	45.305000
H	45.514999	26.368999	46.818001
N	41.692001	25.747999	48.083000
N	40.715000	27.191999	45.778999
N	42.779999	29.129999	46.166000
C	41.391998	24.172001	49.762001
N	43.771999	27.622000	48.412998
C	40.401001	23.986000	48.858002
C	42.226002	25.232000	49.245998
C	43.415001	25.652000	49.830002
C	40.571999	24.982000	47.818001
C	39.723000	25.172001	46.737000
C	39.770000	26.207001	45.793999
C	38.818001	26.367001	44.702000
C	39.245998	27.485001	44.035000
C	40.417000	27.996000	44.706001
C	41.141998	29.132000	44.348999
C	42.234001	29.667999	45.029999
C	42.953999	30.858999	44.605999
C	43.949001	30.997000	45.536999
C	43.837002	29.930000	46.495998
C	44.681000	29.749001	47.598999
C	44.639999	28.695999	48.498001
C	45.512001	28.539000	49.646000
C	44.119999	26.788000	49.453999
C	45.230999	27.337000	50.201000
H	43.798000	25.079000	50.665001
H	38.911999	24.459999	46.631001
H	40.838001	29.653999	43.450001
H	45.467999	30.480000	47.748001
Fe	42.252998	27.396000	47.090000
C	41.091000	28.455999	49.612999
C	40.046001	29.503000	49.745998
C	40.266998	30.891001	49.536999
C	39.207001	31.815001	49.744999
C	41.528999	31.399000	49.113998
C	39.405998	33.176998	49.569000
C	41.702999	32.757999	48.909000
C	40.653999	33.653000	49.148998
H	42.071999	28.771999	49.991001
H	40.807999	27.518999	50.111000
H	39.015999	29.180000	49.886002
H	38.233002	31.441999	50.053001
H	42.339001	30.712000	48.910999
H	38.584999	33.868999	49.738998
H	42.657001	33.138000	48.556000
H	40.807999	34.709999	48.972000
O	41.014000	28.382999	48.215000

aMOx-**TS2**^{q}

S	43.543999	26.249001	45.382000
C	45.111000	25.624001	46.094002
H	45.830002	25.434999	45.290001
H	45.533001	26.358999	46.778999
N	41.683998	25.747999	48.084000
N	40.743000	27.177000	45.778000
N	42.813999	29.128000	46.175999
C	41.388000	24.174999	49.757999
N	43.787998	27.608000	48.396999
C	40.395000	23.990999	48.853001
C	42.223999	25.232000	49.248001
C	43.410999	25.642000	49.834000
C	40.562000	24.983999	47.814999
C	39.717999	25.164000	46.730000
C	39.784000	26.198000	45.794998
C	38.842999	26.372999	44.702000
C	39.284000	27.490000	44.039001
C	40.455002	27.993999	44.709000
C	41.178001	29.129000	44.352001
C	42.264999	29.660000	45.037998
C	42.985001	30.851999	44.618999
C	43.977001	30.996000	45.550999
C	43.870998	29.931999	46.513000
C	44.710999	29.752001	47.609001
C	44.654999	28.688000	48.492001
C	45.513000	28.518000	49.639999
C	44.118999	26.767000	49.450001
C	45.227001	27.313000	50.192001
H	43.790001	25.070999	50.672001
H	38.904999	24.455999	46.620998
H	40.875999	29.650999	43.452000
H	45.499001	30.479000	47.766998
Fe	42.255001	27.403000	47.116001
C	41.074001	28.481001	49.616001
C	40.019001	29.514999	49.771000
C	40.235001	30.906000	49.558998
C	39.186001	31.833000	49.804001
C	41.485001	31.413000	49.099998
C	39.383999	33.195000	49.629002
C	41.659000	32.773998	48.902000
C	40.620998	33.671001	49.175999
H	42.047001	28.791000	50.015999
H	40.789001	27.525999	50.074001
H	38.993999	29.184999	49.928001
H	38.222000	31.462000	50.144001
H	42.287998	30.726999	48.865002
H	38.571999	33.888000	49.828999
H	42.605000	33.152000	48.523998
H	40.773998	34.728001	49.000000
O	41.056000	28.424000	48.208000

aMOx-**2-R**^{d}

S	43.544998	26.356001	45.398998
C	45.080002	25.691000	46.141998
H	45.794998	25.500999	45.333000
H	45.514000	26.413000	46.831001
N	41.643002	25.937000	48.098000
N	40.758999	27.320999	45.779999
N	42.799999	29.240000	46.221001
C	41.341000	24.325001	49.750999
N	43.758999	27.738001	48.435001
C	40.362999	24.138000	48.828999
C	42.169998	25.395000	49.264000
C	43.360001	25.782000	49.860001
C	40.542999	25.136000	47.801998
C	39.726002	25.302000	46.695000
C	39.804001	26.334000	45.761002
C	38.886002	26.497999	44.645000
C	39.334000	27.614000	43.991001
C	40.487000	28.121000	44.689999
C	41.223999	29.246000	44.339001
C	42.296001	29.771999	45.049999
C	43.048000	30.937000	44.625000
C	44.027000	31.073000	45.571999
C	43.881001	30.028999	46.547001
C	44.721001	29.847000	47.644001
C	44.653999	28.792000	48.532001
C	45.521000	28.608999	49.675999
C	44.087002	26.896000	49.485001
C	45.212002	27.412001	50.227001
H	43.730000	25.195000	50.689999
H	38.924000	24.583000	46.567001
H	40.959000	29.750999	43.417000
H	45.515999	30.566000	47.794998
Fe	42.382999	27.426001	47.005001
C	39.655998	28.628000	49.091999
C	38.669998	29.698999	49.338001
C	39.022999	30.959000	50.057999
C	38.372002	31.261999	51.262001
C	39.991001	31.844999	49.568001
C	38.686001	32.426998	51.959999
C	40.301998	33.012001	50.268002
C	39.652000	33.308998	51.466000
H	40.681999	28.775000	49.424999
H	39.324001	27.597000	48.997002
H	37.622002	29.400999	49.410000
H	37.626999	30.573000	51.659000
H	40.497002	31.628000	48.634998
H	38.173000	32.651001	52.890999
H	41.042999	33.699001	49.868999
H	39.889999	34.219002	52.009998
O	39.231998	29.492001	48.030998

aMOx-**2-R**^{q}

S	43.645000	26.278000	45.279999
C	45.167999	25.629999	46.070000
H	45.912998	25.399000	45.300999
H	45.583000	26.372999	46.750000
N	41.627998	25.931000	48.098999
N	40.742001	27.336000	45.799999
N	42.806999	29.249001	46.220001
C	41.334999	24.323000	49.751999
N	43.750000	27.740999	48.425999
C	40.346001	24.143999	48.840000
C	42.167000	25.386000	49.257000
C	43.361000	25.768000	49.842999
C	40.515999	25.146999	47.817001
C	39.687000	25.323000	46.721001
C	39.775002	26.355000	45.789001
C	38.866001	26.521999	44.671001
C	39.327999	27.631001	44.009998
C	40.480000	28.136000	44.703999
C	41.223000	29.254000	44.344002
C	42.299000	29.777000	45.051998
C	43.049000	30.943001	44.625999
C	44.027000	31.084999	45.575001
C	43.882999	30.044001	46.551998
C	44.716999	29.862000	47.648998
C	44.644001	28.799000	48.529999
C	45.507999	28.606001	49.667999
C	44.081001	26.886999	49.470001
C	45.202999	27.402000	50.210999
H	43.737999	25.177999	50.667000
H	38.877998	24.613001	46.596001
H	40.959000	29.756001	43.419998
H	45.513000	30.577999	47.806000
Fe	42.377998	27.423000	46.990002
C	39.646000	28.625999	49.105999
C	38.667000	29.705999	49.334999
C	39.021999	30.965000	50.056000
C	38.372002	31.266001	51.261002
C	39.991001	31.850000	49.568001
C	38.688000	32.430000	51.962002
C	40.303001	33.015999	50.269001
C	39.653999	33.312000	51.467999
H	40.668999	28.764000	49.452000
H	39.306000	27.597000	49.009998
H	37.615002	29.417000	49.394001
H	37.625999	30.577999	51.658001
H	40.497002	31.632999	48.633999
H	38.174000	32.653999	52.893002
H	41.044998	33.702999	49.870998
H	39.893002	34.221001	52.013000
O	39.243999	29.490000	48.036999

aMOx-**Int2**^{d}

S	43.283001	26.370001	45.618999
C	44.904999	25.811001	46.272999
H	45.608002	25.701000	45.438999
H	45.307999	26.531000	46.983002
N	41.428001	25.969000	48.169998
N	40.557999	27.372000	45.837002
N	42.675999	29.299000	46.251999
C	41.202999	24.305000	49.768002
N	43.580002	27.820000	48.514000
C	40.219002	24.114000	48.854000
C	41.993000	25.422001	49.304001
C	43.174000	25.841000	49.902000
C	40.349998	25.155001	47.855000
C	39.518002	25.354000	46.764000
C	39.605999	26.393000	45.820000
C	38.714001	26.545000	44.679001
C	39.188000	27.648001	44.016998
C	40.327999	28.163000	44.735001
C	41.084999	29.278000	44.383999
C	42.174000	29.806000	45.082001
C	42.945000	30.959000	44.632999
C	43.925999	31.098000	45.578999
C	43.758999	30.075001	46.576000
C	44.582001	29.895000	47.688999
C	44.490002	28.851000	48.595001
C	45.376999	28.646000	49.724998
C	43.897999	26.966999	49.542999
C	45.049000	27.455000	50.277000
H	43.560001	25.247000	50.719002
H	38.716000	24.636999	46.624001
H	40.821999	29.783001	43.462002
H	45.396000	30.596001	47.831001
Fe	42.056999	27.608000	47.181999
C	39.743999	28.216999	48.776001
C	38.869999	28.980000	49.653000
C	39.012001	30.297001	50.147999
C	38.077999	30.778000	51.113998
C	40.051998	31.170000	49.714001
C	38.191002	32.060001	51.624001
C	40.131001	32.466000	50.212002
C	39.212002	32.911999	51.167000
H	39.930000	27.259001	49.341999
H	39.049000	27.788000	48.000000
H	37.974998	28.438000	49.959999
H	37.290001	30.115000	51.465000
H	40.779999	30.791000	49.004002
H	37.500000	32.410999	52.387001
H	40.910000	33.137001	49.862999
H	39.285000	33.921001	51.564999
O	40.879002	28.749001	48.284000

aMOx-**Int2**^{q}

S	43.397999	26.334999	45.428001
C	44.967999	25.733999	46.173000
H	45.361000	26.475000	46.869999
H	45.715000	25.563999	45.389000
N	41.402000	26.098000	48.145000
N	40.595001	27.466000	45.749001
N	42.715000	29.379000	46.186001
C	41.161999	24.417000	49.723000
N	43.571999	27.900000	48.477001
C	40.186001	24.238001	48.797001
C	41.959999	25.530001	49.277000
C	43.146000	25.924000	49.874001
C	40.328999	25.285999	47.806000
C	39.521000	25.474001	46.694000
C	39.632999	26.495001	45.737000
C	38.757999	26.643000	44.583000
C	39.247002	27.737000	43.916000
C	40.382999	28.249001	44.639999
C	41.153999	29.356001	44.287998
C	42.236000	29.877001	45.000000
C	43.025002	31.016001	44.556000
C	43.993999	31.153000	45.514999
C	43.803001	30.143000	46.520000
C	44.609001	29.962999	47.645000
C	44.499001	28.924000	48.554001
C	45.375999	28.705000	49.683998
C	43.882999	27.039000	49.509998
C	45.037998	27.514000	50.236000
H	43.527000	25.322001	50.687000
H	38.721001	24.757000	46.547001
H	40.911999	29.851000	43.355000
H	45.430000	30.655001	47.792000
Fe	42.081001	27.704000	47.132999
C	39.618000	28.448000	48.789001
C	38.825001	29.163000	49.783001
C	38.981998	30.479000	50.278000
C	38.120998	30.936001	51.319000
C	39.967999	31.372000	49.768002
C	38.242001	32.222000	51.817001
C	40.064999	32.666000	50.266998
C	39.209999	33.092999	51.287998
H	39.900002	27.502001	49.341000
H	38.849998	27.986000	48.104000
H	37.998001	28.582001	50.192001
H	37.377998	30.254999	51.728001
H	40.644001	31.009001	49.004002
H	37.597000	32.560001	52.625000
H	40.807999	33.348999	49.866001
H	39.294998	34.102001	51.683998
O	40.659000	29.004000	48.157001

aMOx-**TS3-cis-si**[d]

S	43.366001	26.431999	45.576000
C	44.929001	25.795000	46.289001
H	45.646000	25.664000	45.470001
H	45.340000	26.506001	47.004002
N	41.476002	25.947001	48.105999
N	40.584999	27.368999	45.764000
N	42.652000	29.302999	46.202999
C	41.195000	24.320999	49.737000
N	43.577999	27.804001	48.478001
C	40.238998	24.115000	48.796001
C	42.000000	25.424999	49.276001
C	43.165001	25.848000	49.895000
C	40.408001	25.136000	47.778999
C	39.592999	25.313000	46.664001
C	39.658001	26.360001	45.734001
C	38.752998	26.516001	44.604000
C	39.188999	27.646000	43.963001
C	40.323002	28.174000	44.680000
C	41.056999	29.309000	44.340000
C	42.146999	29.827000	45.037998
C	42.922001	30.980000	44.599998
C	43.909000	31.101000	45.543999
C	43.743000	30.066000	46.529999
C	44.574001	29.877001	47.636002
C	44.486000	28.837999	48.547001
C	45.370998	28.645000	49.685001
C	43.894001	26.965000	49.522999
C	45.042999	27.462000	50.251999
H	43.532001	25.270000	50.731998
H	38.806999	24.580999	46.514999
H	40.780998	29.826000	43.428001
H	45.390999	30.576000	47.772999
Fe	42.091999	27.576000	47.110001
C	39.749001	28.195999	48.792999
C	38.849998	28.929001	49.658001
C	39.007000	30.238001	50.205002
C	38.096001	30.686001	51.199001
C	40.004002	31.132999	49.734001
C	38.180000	31.978001	51.695000
C	40.063000	32.433998	50.226002
C	39.159000	32.856998	51.205002
H	39.966000	27.580000	49.776001
H	39.210999	27.413000	48.223999
H	37.979000	28.357000	49.976002
H	37.334999	30.004000	51.573002
H	40.724998	30.775000	49.007000
H	37.498001	32.313000	52.473000
H	40.813000	33.124001	49.851002
H	39.215000	33.869999	51.596001
O	40.834000	28.709000	48.256001

aMOx-**TS3-cis-si**[q]

S	43.435001	26.327999	45.391998
C	44.992001	25.719999	46.157001
H	45.744999	25.537001	45.382000
H	45.382999	26.464001	46.852001
N	41.411999	26.075001	48.111000
N	40.602001	27.447001	45.727001
N	42.717999	29.361000	46.167000
C	41.153999	24.413000	49.708000
N	43.575001	27.882000	48.455002
C	40.183998	24.228001	48.776001
C	41.957001	25.521000	49.256001
C	43.139000	25.915001	49.861000
C	40.339001	25.264999	47.777000
C	39.535999	25.443001	46.657001
C	39.644001	26.468000	45.708000
C	38.765999	26.624001	44.558998
C	39.245998	27.728001	43.902000
C	40.382000	28.239000	44.625999
C	41.146000	29.351999	44.278999
C	42.230999	29.868000	44.987999
C	43.015999	31.011999	44.549000
C	43.988998	31.143999	45.505001
C	43.805000	30.127001	46.504002
C	44.613998	29.945000	47.625000
C	44.500999	28.907000	48.533001
C	45.372002	28.691000	49.668999
C	43.880001	27.025000	49.494999
C	45.030998	27.502001	50.223999
H	43.511002	25.319000	50.681999
H	38.738998	24.723000	46.507999
H	40.897999	29.853001	43.351002
H	45.435001	30.636999	47.772999
Fe	42.106998	27.662001	47.088001
C	39.597000	28.492001	48.757000
C	38.759998	29.190001	49.716000
C	38.957001	30.479000	50.285999
C	38.105999	30.909000	51.341000
C	39.942001	31.375000	49.791000
C	38.229000	32.186001	51.863998
C	40.048000	32.658001	50.319000
C	39.196999	33.064999	51.349998
H	39.884998	27.774000	49.634998
H	38.987999	27.768999	48.167999
H	37.916000	28.605000	50.083000
H	37.360001	30.223000	51.737000
H	40.615002	31.028000	49.016998
H	37.585999	32.509998	52.678001
H	40.791000	33.347000	49.929001
H	39.285999	34.066002	51.763000
O	40.641998	29.021000	48.159000

aMOx-**TS3-trans-re**[d]

S	43.353001	26.431000	45.599998
C	44.929001	25.795000	46.285000
H	45.633999	25.670000	45.453999
H	45.348999	26.503000	46.998001
N	41.522999	25.884001	48.154999
N	40.563999	27.336000	45.858002
N	42.624001	29.278999	46.273998
C	41.270000	24.247999	49.778999
N	43.604000	27.764000	48.515999
C	40.300999	24.045000	48.851002
C	42.063999	25.357000	49.312000
C	43.229000	25.792999	49.924999
C	40.445999	25.070000	47.838001
C	39.605999	25.259001	46.749001
C	39.643002	26.320999	45.834999
C	38.724998	26.472000	44.715000
C	39.151001	27.601999	44.067001
C	40.287998	28.139000	44.775002
C	41.011002	29.277000	44.426998
C	42.106998	29.799999	45.113998
C	42.875999	30.952999	44.664001
C	43.872002	31.077999	45.597000
C	43.719002	30.044001	46.587002
C	44.564999	29.856001	47.680000
C	44.497002	28.809999	48.585999
C	45.387001	28.629000	49.720001
C	43.936001	26.926001	49.557999
C	45.074001	27.443001	50.287998
H	43.613998	25.211000	50.750999
H	38.820999	24.524000	46.606998
H	40.724998	29.792000	43.518002
H	45.377998	30.561001	47.812000
Fe	42.099998	27.534000	47.176998
C	39.771999	28.112000	48.915001
C	38.879002	28.820000	49.806000
C	38.891998	30.195000	50.183998
C	37.886002	30.670000	51.066002
C	39.910999	31.087999	49.759998
C	37.908001	31.982000	51.514000
C	39.902000	32.410999	50.191002
C	38.910000	32.858002	51.070000
H	39.785999	27.025999	49.125999
H	38.834000	28.124001	48.192001
H	38.037998	28.221001	50.153000
H	37.112000	29.988001	51.410999
H	40.695000	30.712000	49.110001
H	37.159000	32.332001	52.221001
H	40.671001	33.098999	49.852001
H	38.917000	33.887001	51.421001
O	40.860001	28.629000	48.394001

aMOx-**TS3-trans-re**[q]

S	43.436001	26.333000	45.395000
C	44.997002	25.722000	46.153000
H	45.745998	25.539000	45.374001
H	45.390999	26.465000	46.847000
N	41.439999	26.028000	48.123001
N	40.588001	27.429001	45.772999
N	42.695999	29.350000	46.205002
C	41.189999	24.365999	49.720001
N	43.584999	27.856001	48.471001
C	40.215000	24.180000	48.793999
C	41.988998	25.474001	49.264000
C	43.167999	25.879999	49.869999
C	40.361000	25.218000	47.793999
C	39.544998	25.405001	46.688000
C	39.637001	26.443001	45.751999
C	38.751999	26.599001	44.608002
C	39.222000	27.709000	43.952999
C	40.356998	28.224001	44.674000
C	41.112999	29.341999	44.326000
C	42.202000	29.858000	45.028999
C	42.985001	31.002001	44.584999
C	43.964001	31.134001	45.535000
C	43.785999	30.115000	46.534000
C	44.604000	29.933001	47.647999
C	44.500000	28.889000	48.553001
C	45.372002	28.680000	49.688000
C	43.896000	27.000000	49.508999
C	45.037998	27.490000	50.243000
H	43.548000	25.283001	50.687000
H	38.750999	24.681999	46.539001
H	40.858002	29.844999	43.401001
H	45.421001	30.628000	47.793999
Fe	42.106998	27.638000	47.117001
C	39.632999	28.410999	48.908001
C	38.824001	29.082001	49.909000
C	38.862999	30.441999	50.320999
C	37.930000	30.895000	51.292999
C	39.842999	31.348000	49.833000
C	37.980000	32.201000	51.755001
C	39.868999	32.660000	50.292999
C	38.944000	33.088001	51.249001
H	39.730000	27.330999	49.159000
H	38.664001	28.327000	48.257000
H	38.023998	28.465000	50.321999
H	37.191002	30.200001	51.687000
H	40.569000	30.986000	49.116001
H	37.282001	32.537998	52.518002
H	40.610001	33.355999	49.910000
H	38.972000	34.112000	51.612999
O	40.652000	28.957001	48.279999

aMOx-**3**^{d}

S	43.349998	26.447001	45.590000
C	44.918999	25.802999	46.280998
H	45.623001	25.691999	45.448002
H	45.338001	26.504999	47.000999
N	41.522999	25.940001	48.157001
N	40.577999	27.371000	45.863998
N	42.620998	29.316999	46.289001
C	41.299999	24.268000	49.755001
N	43.598000	27.809999	48.520000
C	40.328999	24.069000	48.827999
C	42.076000	25.395000	49.305000
C	43.243000	25.825001	49.914001
C	40.462002	25.108999	47.830002
C	39.632999	25.281000	46.729000
C	39.669998	26.341999	45.820000
C	38.759998	26.489000	44.694000
C	39.174999	27.629999	44.058998
C	40.299000	28.174000	44.777000
C	41.015999	29.315001	44.432999
C	42.108002	29.839001	45.122002
C	42.876999	30.988001	44.672001
C	43.875999	31.112000	45.604000
C	43.721001	30.083000	46.595001
C	44.569000	29.898001	47.686001
C	44.498001	28.855000	48.591000
C	45.388000	28.669001	49.721001
C	43.938999	26.966000	49.556999
C	45.075001	27.481001	50.285999
H	43.640999	25.230000	50.723999
H	38.860001	24.535000	46.580002
H	40.731998	29.827000	43.521000
H	45.382000	30.603001	47.817001
Fe	42.141998	27.544001	47.148998
C	39.646000	28.278999	48.995998
C	38.589001	28.985001	49.820000
C	38.830002	30.426001	50.209000
C	39.502998	30.742001	51.398998
C	38.367001	31.476999	49.404999
C	39.716999	32.069000	51.772999
C	38.598000	32.806999	49.765999
C	39.268002	33.108002	50.952999
H	38.438999	28.351999	50.709000
H	39.417999	27.222000	48.786999
H	37.653999	28.878000	49.245998
H	39.861000	29.945000	52.042000
H	37.824001	31.261000	48.486000
H	40.237000	32.298000	52.700001
H	38.251999	33.608002	49.118000
H	39.435001	34.139999	51.247002
O	40.675999	28.775000	48.567001

aMOx-**3**^{q}

S	43.534000	26.327999	45.349998
C	45.077000	25.694000	46.113998
H	45.819000	25.495001	45.334000
H	45.484001	26.431999	46.805000
N	41.550999	25.955999	48.133999
N	40.639999	27.370001	45.839001
N	42.702999	29.306000	46.259998
C	41.313999	24.297001	49.743999
N	43.660000	27.794001	48.466000
C	40.326000	24.115000	48.832001
C	42.111000	25.400999	49.273998
C	43.296001	25.804001	49.863998
C	40.464001	25.148001	47.831001
C	39.629002	25.323999	46.737999
C	39.695999	26.370001	45.816002
C	38.786999	26.531000	44.696999
C	39.229000	27.656000	44.048000
C	40.369999	28.177000	44.750999
C	41.097000	29.308001	44.401001
C	42.182999	29.830999	45.097000
C	42.938999	30.992001	44.659000
C	43.931999	31.125000	45.596001
C	43.789001	30.090000	46.577999
C	44.629002	29.906000	47.672001
C	44.554001	28.851000	48.560001
C	45.425999	28.658001	49.695000
C	43.995998	26.940001	49.504002
C	45.118999	27.457001	50.243000
H	43.693001	25.205000	50.672001
H	38.837002	24.597000	46.602001
H	40.817001	29.820000	43.488998
H	45.432999	30.617001	47.819000
Fe	42.249001	27.500000	47.068001
C	39.347000	28.457001	49.070999
C	38.252998	29.134001	49.882999
C	38.537998	30.563000	50.278000
C	39.145000	30.853001	51.508999
C	38.242001	31.625000	49.412998
C	39.462002	32.165001	51.862000
C	38.567001	32.938999	49.757999
C	39.176998	33.214001	50.984001
H	38.074001	28.497999	50.762001
H	39.186001	27.377001	48.896000
H	37.334000	29.052000	49.278999
H	39.369999	30.045000	52.199001
H	37.758999	31.427999	48.457001
H	39.937000	32.374001	52.818001
H	38.342999	33.745998	49.064999
H	39.422001	34.234001	51.264000
O	40.330002	29.011999	48.619999

D. References

(1) Frisch, M. J.; Trucks, G. W.; Schlegel, H. B.; Scuseria, G. E.; Robb, M. a.; Cheeseman, J. R.; Scalmani, G.; Barone, V.; Petersson, G. a.; Nakatsuji, H.; Li, X.; Caricato, M.; Marenich, a. V.; Bloino, J.; Janesko, B. G.; Gomperts, R.; Mennucci, B.; Hratchian, H. P.; Ortiz, J. V.; Izmaylov, a. F.; Sonnenberg, J. L.; Williams; Ding, F.; Lipparini, F.; Egidi, F.; Goings, J.; Peng, B.; Petrone, A.; Henderson, T.; Ranasinghe, D.; Zakrzewski, V. G.; Gao, J.; Rega, N.; Zheng, G.; Liang, W.; Hada, M.; Ehara, M.; Toyota, K.; Fukuda, R.; Hasegawa, J.; Ishida, M.; Nakajima, T.; Honda, Y.; Kitao, O.; Nakai, H.; Vreven, T.; Throssell, K.; Montgomery Jr., J. a.; Peralta, J. E.; Ogliaro, F.; Bearpark, M. J.; Heyd, J. J.; Brothers, E. N.; Kudin, K. N.; Staroverov, V. N.; Keith, T. a.; Kobayashi, R.; Normand, J.; Raghavachari, K.; Rendell, a. P.; Burant, J. C.; Iyengar, S. S.; Tomasi, J.; Cossi, M.; Millam, J. M.; Klene, M.; Adamo, C.; Cammi, R.; Ochterski, J. W.; Martin, R. L.; Morokuma, K.; Farkas, O.; Foresman, J. B.; Fox, D. J. Gaussian 09. 2009, p Gaussian 09, Revision D.01, Gaussian, Inc., Wallin.

(2) Lee, C.; Yang, W.; Parr, R. G. Development of the Colle-Salvetti Correlation-Energy Formula into a Functional of the Electron Density. *Phys. Rev. B* **1988**, *37* (2), 785–789. https://doi.org/10.1103/PhysRevB.37.785.

(3) Becke, A. D. Density-functional Thermochemistry. III. The Role of Exact Exchange. *J. Chem. Phys.* **1993**, *98* (7), 5648–5652. https://doi.org/10.1063/1.464913.

(4) Becke, A. D. Density-Functional Exchange-Energy Approximation with Correct Asymptotic Behavior. *Phys. Rev. A* **1988**, *38* (6), 3098–3100. https://doi.org/10.1103/PhysRevA.38.3098.

(5) Bootsma, A.; N.; Wheeler, S. Popular Integration Grids Can Result in Large Errors in DFT-Computed Free Energies. **2019**. https://doi.org/10.26434/CHEMRXIV.8864204.V5.

(6) Barone, V.; Cossi, M. Quantum Calculation of Molecular Energies and Energy Gradients in Solution by a Conductor Solvent Model. *J. Phys. Chem. A* **1998**, *102* (11), 1995–2001. https://doi.org/10.1021/jp9716997.

(7) Cossi, M.; Rega, N.; Scalmani, G.; Barone, V. Energies, Structures, and Electronic Properties of Molecules in Solution with the C-PCM Solvation Model. *J. Comput. Chem.* **2003**, *24* (6), 669–681. https://doi.org/10.1002/jcc.10189.

(8) Schutz, C. N.; Warshel, A. What Are the Dielectric "Constants" of Proteins and How to Validate Electrostatic Models? *Proteins Struct. Funct. Genet.* **2001**, *44* (4), 400–417. https://doi.org/10.1002/prot.1106.

(9) Li, L.; Li, C.; Zhang, Z.; Alexov, E. On the Dielectric "Constant" of Proteins: Smooth Dielectric Function for Macromolecular Modeling and Its Implementation in DelPhi. *J. Chem. Theory Comput.* **2013**, *9* (4), 2126–2136. https://doi.org/10.1021/ct400065j.

(10) Warshel, A.; Papazyan, A. Electrostatic Effects in Macromolecules: Fundamental Concepts and Practical Modeling. *Curr. Opin. Struct. Biol.* **1998**, *8*, 211–217.

(11) Ribeiro, R. F.; Marenich, A. V.; Cramer, C. J.; Truhlar, D. G. Use of Solution-Phase Vibrational Frequencies in Continuum Models for the Free Energy of Solvation. *J. Phys. Chem. B* **2011**, *115* (49), 14556–14562. https://doi.org/10.1021/jp205508z.

(12) Zhao, Y.; Truhlar, D. G. Computational Characterization and Modeling of Buckyball Tweezers: Density Functional Study of Concave–Convex Π⋯π Interactions. *Phys. Chem. Chem. Phys.* **2008**, *10* (19), 2813. https://doi.org/10.1039/b717744e.

(13) Funes-Ardoiz, I.; Paton, R. S. GoodVibes: GoodVibes v1.0.1. August 2016. https://doi.org/10.5281/ZENODO.60811.

(14) Grimme, S.; Ehrlich, S.; Goerigk, L. Effect of the Damping Function in Dispersion Corrected Density Functional Theory. *J. Comput. Chem.* **2011**, *32* (7), 1456–1465. https://doi.org/10.1002/jcc.21759.

(15) Shaik, S.; Cohen, S.; Wang, Y.; Chen, H.; Kumar, D.; Thiel, W. P450 Enzymes: Their

Structure, Reactivity, and Selectivity - Modeled by QM/MM Calculations. *Chem. Rev.* **2010**, *110* (2), 949–1017. https://doi.org/10.1021/cr900121s.
(16) Radon, M.; Broclawik, E. Peculiarities of the Electronic Structure of Cytochrome P450 Compound I: CASPT2 and DFT Modeling. *J. Chem. Theory Comput.* **2007**, *3*, 728–734.
(17) Chen, H.; Song, J.; Lai, W.; Wu, W.; Shaik, S. Multiple Low-Lying States for Compound I of P450cam and Chloroperoxidase Revealed from Multireference Ab Initio QM/MM Calculations. *J. Chem. Theory Comput.* **2010**, *6* (3), 940–953. https://doi.org/10.1021/ct9006234.
(18) Chen, H.; Lai, W.; Shaik, S. Multireference and Multiconfiguration Ab Initio Methods in Heme-Related Systems: What Have We Learned so Far? *J. Phys. Chem. B* **2011**, *115* (8), 1727–1742. https://doi.org/10.1021/jp110016u.
(19) Legault, C. Y. CYLview, 1.0b. Université de Sherbrooke 2009, p Université de Sherbrooke.
(20) The PyMOL Molecular Graphics System, Version 2.0. p Schrödinger, LLC.
(21) Harvey, J. N.; Aschi, M.; Schwarz, H.; Koch, W. The Singlet and Triplet States of Phenyl Cation. A Hybrid Approach for Locating Minimum Energy Crossing Points between Non-Interacting Potential Energy Surfaces. *Theor. Chem. Acc.* **1998**, *99* (2), 95–99. https://doi.org/10.1007/s002140050309.
(22) Rodríguez-Guerra, J. Jaimergp/Easymecp: V0.3.2. **2020**. https://doi.org/10.5281/ZENODO.4293422.
(23) Kurouchi, H.; Singleton, D. A. Labelling and Determination of the Energy in Reactive Intermediates in Solution Enabled by Energy-Dependent Reaction Selectivity. *Nat. Chem.* **2018**, *10* (2), 237–241. https://doi.org/10.1038/NCHEM.2907.
(24) Yang, Z.; Houk, K. N. The Dynamics of Chemical Reactions: Atomistic Visualizations of Organic Reactions, and Homage to van 't Hoff. *Chem. Eur. J.* **2018**, *24* (16), 3916–3924. https://doi.org/10.1002/chem.201706032.
(25) Gonzalez-James, O. M.; Kwan, E. E.; Singleton, D. A. Entropic Intermediates and Hidden Rate-Limiting Steps in Seemingly Concerted Cycloadditions. Observation, Prediction, and Origin of an Isotope Effect on Recrossing. *J. Am. Chem. Soc.* **2012**, *134* (4), 1914–1917. https://doi.org/10.1021/ja208779k.
(26) Kelly, K. K.; Hirschi, J. S.; Singleton, D. A. Newtonian Kinetic Isotope Effects . Observation, Prediction, and Origin of Heavy-Atom Dynamic Isotope Effects. *J. Am. Chem. Soc* **2009**, *131*, 8382–8383.
(27) Wang, Z.; Hirschi, J. S.; Singleton, D. A. Recrossing and Dynamic Matching Effects on Selectivity in a Diels-Alder Reaction. *Angew. Chemie - Int. Ed.* **2009**, *48* (48), 9156–9159. https://doi.org/10.1002/anie.200903293.
(28) Waterhouse, A.; Bertoni, M.; Bienert, S.; Studer, G.; Tauriello, G.; Gumienny, R.; Heer, F. T.; De Beer, T. A. P.; Rempfer, C.; Bordoli, L.; Lepore, R.; Schwede, T. SWISS-MODEL: Homology Modelling of Protein Structures and Complexes. *Nucleic Acids Res.* **2018**, *46* (W1), W296–W303. https://doi.org/10.1093/nar/gky427.
(29) Roe, D. R.; Cheatham, T. E. PTRAJ and CPPTRAJ: Software for Processing and Analysis of Molecular Dynamics Trajectory Data. *J. Chem. Theory Comput.* **2013**, *9* (7), 3084–3095. https://doi.org/10.1021/ct400341p.
(30) Richter, F.; Leaver-Fay, A.; Khare, S. D.; Bjelic, S.; Baker, D. De Novo Enzyme Design Using Rosetta3. *PLoS One* **2011**, *6* (5), 1–12. https://doi.org/10.1371/journal.pone.0019230.
(31) Case, D. A.; Ben-Shalom, I. Y.; Brozell, S. R.; Cerutti, D. S.; T.E. Cheatham, I.; Cruzeiro, V. W. D.; Darden, T. A.; Duke, R. E.; Ghoreishi, D.; Gilson, M. K.; Gohlke, H.; Goetz, A. W.; Greene, D.; Harris, R.; Homeyer, N.; Huang, Y.; Izadi, S.; Kovalenko, A.; Kurtzman, T.; Lee, T. S.; LeGrand, S.; Li, P.; Lin, C.; Liu, J.; Luchko, T.; Luo, R.; Mermelstein, D. J.; Merz, K. M.; Miao, Y.; Monard, G.; Nguyen, C.; Nguyen, H.;

Omelyan, I.; Onufriev, A.; Pan, F.; Qi, R.; Roe, D. R.; Roitberg, A.; Sagui, C.; Schott-Verdugo; Shen, S.; Simmerling, J. C. L.; Smith, J.; SalomonFerrer, R.; Swails, J.; Walker, R. C.; Wang, J.; Wei, H.; Wolf, R. M.; Wu, X.; Xiao, L.; York, D. M.; Kollman, P. A. AMBER 2018. University of California, San Francisco.
(32) Cruzeiro, V. W. D.; Amaral, M. S.; Roitberg, A. E. Redox Potential Replica Exchange Molecular Dynamics at Constant PH in AMBER: Implementation and Validation. *J. Chem. Phys.* **2018**, *149* (7), 072338. https://doi.org/10.1063/1.5027379.
(33) Shahrokh, K.; Orendt, A.; Yost, G. S.; Cheatham, T. E. Quantum Mechanically Derived AMBER-Compatible Heme Parameters for Various States of the Cytochrome P450 Catalytic Cycle. *J. Comput. Chem.* **2012**, *33* (2), 119–133. https://doi.org/10.1002/jcc.21922.
(34) Jorgensen, W. L.; Chandrasekhar, J.; Madura, J. D.; Impey, R. W.; Klein, M. L. Comparison of Simple Potential Functions for Simulating Liquid Water. *J. Chem. Phys.* **1998**, *79* (2), 926. https://doi.org/10.1063/1.445869.
(35) Maier, J. A.; Martinez, C.; Kasavajhala, K.; Wickstrom, L.; Hauser, K. E.; Simmerling, C. Ff14SB: Improving the Accuracy of Protein Side Chain and Backbone Parameters from Ff99SB. *J. Chem. Theory Comput.* **2015**, *11* (8), 3696–3713. https://doi.org/10.1021/acs.jctc.5b00255.
(36) Darden, T.; York, D.; Pedersen, L. Particle Mesh Ewald: An N·log(N) Method for Ewald Sums in Large Systems. *J. Chem. Phys.* **1998**, *98* (12), 10089. https://doi.org/10.1063/1.464397.
(37) Wagner, J. R.; Sørensen, J.; Hensley, N.; Wong, C.; Zhu, C.; Perison, T.; Amaro, R. E. POVME 3.0: Software for Mapping Binding Pocket Flexibility. *J. Chem. Theory Comput.* **2017**, *13* (9), 4584–4592. https://doi.org/10.1021/acs.jctc.7b00500.
(38) Humphrey, W.; Dalke, A.; Schulten, K. VMD: Visual Molecular Dynamics. *J. Mol. Graph.* **1996**, *14* (1), 33–38. https://doi.org/10.1016/0263-7855(96)00018-5.
(39) Li, P.; Merz, K. M. MCPB.Py: A Python Based Metal Center Parameter Builder. *J. Chem. Inf. Model.* **2016**, *56* (4), 599–604. https://doi.org/10.1021/acs.jcim.5b00674.
(40) Wang, J.; Wolf, R. M.; Caldwell, J. W.; Kollman, P. A.; Case, D. A. Development and Testing of a General Amber Force Field. *J. Comput. Chem.* **2004**, *25* (9), 1157–1174. https://doi.org/10.1002/jcc.20035.
(41) Bayly, C. I.; Cieplak, P.; Cornell, W.; Kollman, P. A. A Well-Behaved Electrostatic Potential Based Method Using Charge Restraints for Deriving Atomic Charges: The RESP Model. *J. Phys. Chem.* **1993**, *97* (40), 10269–10280. https://doi.org/10.1021/j100142a004.
(42) Besler, B. H.; Merz, K. M.; Kollman, P. A. Atomic Charges Derived from Semiempirical Methods. *J. Comput. Chem.* **1990**, *11* (4), 431–439. https://doi.org/10.1002/jcc.540110404.
(43) Singh, U. C.; Kollman, P. A. An Approach to Computing Electrostatic Charges for Molecules. *J. Comput. Chem.* **1984**, *5* (2), 129–145. https://doi.org/10.1002/jcc.540050204.
(44) Trott, O.; Olson, A. J. AutoDock Vina: Improving the Speed and Accuracy of Docking with a New Scoring Function, Efficient Optimization, and Multithreading. *J. Comput. Chem.* **2009**, *31* (2), NA-NA. https://doi.org/10.1002/jcc.21334.
(45) Li, P.; Merz, K. M. MCPB.Py: A Python Based Metal Center Parameter Builder. *J. Chem. Inf. Model.* **2016**, *56* (4), 599–604. https://doi.org/10.1021/acs.jcim.5b00674.
(46) Dapprich, S.; Komáromi, I.; Byun, K. S.; Morokuma, K.; Frisch, M. J. A New ONIOM Implementation in Gaussian98. Part I. The Calculation of Energies, Gradients, Vibrational Frequencies and Electric Field Derivatives. *J. Mol. Struct. THEOCHEM* **1999**, *461–462*, 1–21. https://doi.org/10.1016/S0166-1280(98)00475-8.
(47) Vreven, T.; Byun, K. S.; Komáromi, I.; Dapprich, S.; John A. Montgomery, J.;

Morokuma, K.; Frisch, M. J. Combining Quantum Mechanics Methods with Molecular Mechanics Methods in ONIOM. *J. Chem. Theory Comput.* **2006**, *2*, 815–826. https://doi.org/10.2223/jped.1687.

(48) Vreven, T.; Morokuma, K.; Farkas, Ö.; Schlegel, H. B.; Frisch, M. J. Geometry Optimization with QM/MM, ONIOM, and Other Combined Methods. I. Microiterations and Constraints. *J. Comput. Chem.* **2003**, *24* (6), 760–769. https://doi.org/10.1002/jcc.10156.

(49) S. Fernandes, H.; Ramos, M. J.; M. F. S. A. Cerqueira, N. MolUP: A VMD Plugin to Handle QM and ONIOM Calculations Using the Gaussian Software. *J. Comput. Chem.* **2018**, *39* (19), 1344–1353. https://doi.org/10.1002/jcc.25189.

(50) Stuyver, T.; Huang, J.; Mallick, D.; Danovich, D.; Shaik, S. TITAN: A Code for Modeling and Generating Electric Fields—Features and Applications to Enzymatic Reactivity. *J. Comput. Chem.* **2020**, *41* (1), 74–82. https://doi.org/10.1002/jcc.26072.

(51) Bím, D.; Alexandrova, A. N. Local Electric Fields As a Natural Switch of Heme-Iron Protein Reactivity. *ACS Catal.* **2021**, *11*, 6534–6546. https://doi.org/10.1021/acscatal.1c00687.

(52) Shaik, S.; Ramanan, R.; Danovich, D.; Mandal, D. Structure and Reactivity/Selectivity Control by Oriented-External Electric Fields. *Chem. Soc. Rev.* **2018**, *47* (14), 5125–5145. https://doi.org/10.1039/c8cs00354h.

(53) Carpenter, B. K. Dynamic Behavior of Organic Reactive Intermediates. *Angew. Chemie Int. Ed.* **1998**, *37*, 3340–3350. https://doi.org/10.1002/(sici)1521-3773(19981231)37:24<3340::aid-anie3340>3.0.co;2-1.

(54) Carpenter, B. K. Energy Disposition in Reactive Intermediates. *Chemical Reviews*. 2013, pp 7265–7286. https://doi.org/10.1021/cr300511u.

(55) López, J. G.; Vayner, G.; Lourderaj, U.; Addepalli, S. V; Kato, S.; DeJong, W. A.; Windus, T. L.; Hase, W. L. A Direct Dynamics Trajectory Study of F- + CH3OOH Reactive Collisions Reveals a Major Non-IRC Reaction Path. *J. Am. Chem. Soc.* **2007**, *129* (32), 9976–9985. https://doi.org/10.1021/ja0717360.

(56) Biswas, B.; Singleton, D. A. Controlling Selectivity by Controlling the Path of Trajectories. *J. Am. Chem. Soc.* **2015**, *137* (45), 14244–14247. https://doi.org/10.1021/jacs.5b08635.

(57) Ess, D. H.; Wheeler, S. E.; Iafe, R. G.; Xu, L.; Çelebi-Ölçüm, N.; Houk, K. N. Bifurcations on Potential Energy Surfaces of Organic Reactions. *Angew. Chem. Int. Ed.* **2008**, *47* (40), 7592–7601. https://doi.org/10.1002/anie.200800918.

(58) Hare, S. R.; Tantillo, D. J. Post-Transition State Bifurcations Gain Momentum-Current State of the Field. *Pure Appl. Chem.* **2017**, *89* (6), 679–698. https://doi.org/10.1515/pac-2017-0104.

(59) Carlsen, R.; Wohlgemuth, N.; Carlson, L.; Ess, D. H. Dynamical Mechanism May Avoid High-Oxidation State Ir(V)-H Intermediate and Coordination Complex in Alkane and Arene C-H Activation by Cationic Ir(III) Phosphine. *J. Am. Chem. Soc.* **2018**, *140* (35), 11039–11045. https://doi.org/10.1021/jacs.8b05238.

(60) Rowley, C. N.; Woo, T. K. A Path Sampling Study of Ru-Hydride-Catalyzed H2 Hydrogenation of Ethylene. *J. Am. Chem. Soc.* **2008**, *130* (23), 7218–7219. https://doi.org/10.1021/ja802219a.

(61) Hare, S. R.; Tantillo, D. J. Cryptic Post-Transition State Bifurcations That Reduce the Efficiency of Lactone-Forming Rh-Carbenoid C-H Insertions. *Chem. Sci.* **2017**, *8* (2), 1442–1449. https://doi.org/10.1039/c6sc03745c.

(62) Ye, L.; Wang, Y.; Aue, D. H.; Zhang, L. Experimental and Computational Evidence for Gold Vinylidenes: Generation from Terminal Alkynes via a Bifurcation Pathway and Facile C-H Insertions. *J. Am. Chem. Soc.* **2012**, *134* (1), 31–34. https://doi.org/10.1021/ja2091992.

(63) Zhang, L.; Wang, Y.; Yao, Z. J.; Wang, S.; Yu, Z. X. Kinetic or Dynamic Control on a Bifurcating Potential Energy Surface? An Experimental and DFT Study of Gold-Catalyzed Ring Expansion and Spirocyclization of 2-Propargyl-β-Tetrahydrocarbolines. *J. Am. Chem. Soc.* **2015**, *137* (41), 13290–13300. https://doi.org/10.1021/jacs.5b05971.

(64) Noey, E. L.; Wang, X.; Houk, K. N. Selective Gold(I)-Catalyzed Formation of Tetracyclic Indolines: A Single Transition Structure and Bifurcations Lead to Multiple Products. *J. Org. Chem.* **2011**, *76* (9), 3477–3483. https://doi.org/10.1021/jo200556f.

(65) Yang, B.; Schouten, A.; Ess, D. H. Direct Dynamics Trajectories Reveal Nonstatistical Coordination Intermediates and Demonstrate That σ and ?-Coordination Are Not Required for Rhenium(I)-Mediated Ethylene C-H Activation. *J. Am. Chem. Soc.* **2021**, *143* (22), 8367–8374. https://doi.org/10.1021/jacs.1c01709.

(66) Teynor, M. S.; Scott, W.; Ess, D. H. Catalysis with a Skip: Dynamically Coupled Addition, Proton Transfer, and Elimination during Au- and Pd-Catalyzed Diol Cyclizations. *ACS Catal.* **2021**, *11* (16), 10179–10189. https://doi.org/10.1021/acscatal.1c02408.

(67) Ess, D. H. Quasiclassical Direct Dynamics Trajectory Simulations of Organometallic Reactions. *Acc. Chem. Res.* **2021**, *54* (23), 4410–4422. https://doi.org/10.1021/acs.accounts.1c00575.

(68) Wang, J.-C.; Sakakibara, M.; Liu, J.-Q.; Dairi, T.; Itoh, N. Cloning, Sequence Analysis, and Expression in Escherichia Coli of the Gene Encoding Phenylacetaldehyde Reductase from Styrene-Assimilating Corynebacterium Sp. Strain ST-10. *Appl. Microbiol. Biotechnol.* **1999**, *52* (3), 386–392. https://doi.org/10.1007/s002530051536.

(69) Hammer, S. C.; Kubik, G.; Watkins, E.; Huang, S.; Minges, H.; Arnold, F. H. Anti-Markovnikov Alkene Oxidation by Metal-Oxo–Mediated Enzyme Catalysis. *Science* **2017**, *358* (6360), 215–218. https://doi.org/10.1126/science.aao1482.

(70) Guengerich, F. P.; Martin, M. V; Sohl, C. D.; Cheng, Q. Measurement of Cytochrome P450 and NADPH–Cytochrome P450 Reductase. *Nat. Protoc.* **2009**, *4* (9), 1245–1251. https://doi.org/10.1038/nprot.2009.121.

(71) Wei, Y.; Tinoco, A.; Steck, V.; Fasan, R.; Zhang, Y. Cyclopropanations via Heme Carbenes: Basic Mechanism and Effects of Carbene Substituent, Protein Axial Ligand, and Porphyrin Substitution. *J. Am. Chem. Soc.* **2018**, *140* (5), 1649–1662. https://doi.org/10.1021/jacs.7b09171.

(72) Ball, L. T.; Lloyd-Jones, G. C.; Russell, C. A. Gold-Catalysed Oxyarylation of Styrenes and Mono- and Gem-Disubstituted Olefins Facilitated by an Iodine(III) Oxidant. *Chem. Eur. J* **2012**, *18* (10), 2931–2937. https://doi.org/10.1002/chem.201103061.

(73) Hesse, M.; Meier, H.; Zeeh, B. *Spektroskopische Methoden in Der Organischen Chemie*; Georg Thieme Verlag: Stuttgart, 2012.

(74) Oba, M. A Convenient Method for Palladium-Catalyzed Reductive Deuteration of Organic Substrates Using Deuterated Hypophosphite in D2O. *J. Label. Compd. Radiopharm.* **2015**, *58* (5), 215–219. https://doi.org/10.1002/jlcr.3277.

(75) Mandrelli, F.; Blond, A.; James, T.; Kim, H.; List, B. Deracemizing A-branched Carboxylic Acids by Catalytic Asymmetric Protonation of Bis-silyl Ketene Acetals with Water or Methanol. *Angew. Chem. Int. Ed.* **2019**, *58* (33), 11479–11482. https://doi.org/10.1002/anie.201905623.

(76) Matoishi, K.; Hanzawa, S.; Kakidani, H.; Suzuki, M.; Sugai, T.; Ohta, H. The First Synthesis of Both Enantiomers of [α-2H]Phenylacetic Acid in High Enantiomeric Excess. *Chem. Commun.* **2000**, 1519–1520. https://doi.org/10.1039/b003941l.

(77) Hoye, T. R.; Jeffrey, C. S.; Shao, F. Mosher Ester Analysis for the Determination of Absolute Configuration of Stereogenic (Chiral) Carbinol Carbons. *Nat. Protoc.* **2007**, *2* (10), 2451–2458. https://doi.org/10.1038/nprot.2007.354.
